# Testing DNA Barcode Performance in 1000 Species of European Lepidoptera: Large Geographic Distances Have Small Genetic Impacts

**DOI:** 10.1371/journal.pone.0115774

**Published:** 2014-12-26

**Authors:** Peter Huemer, Marko Mutanen, Kristina M. Sefc, Paul D. N. Hebert

**Affiliations:** 1 Tiroler Landesmuseen-Betriebsges.m.b.H., Naturwissenschaftliche Sammlungen, Innsbruck, Austria; 2 Biodiversity Unit, Department of Biology, University of Oulu, Oulu, Finland; 3 University of Graz, Institute of Zoology, Graz, Austria; 4 Biodiversity Institute of Ontario, University of Guelph, Guelph, ON, Canada; University of Veterinary Medicine Hanover, Germany

## Abstract

This study examines the performance of DNA barcodes (mt cytochrome *c* oxidase 1 gene) in the identification of 1004 species of Lepidoptera shared by two localities (Finland, Austria) that are 1600 km apart. Maximum intraspecific distances for the pooled data were less than 2% for 880 species (87.6%), while deeper divergence was detected in 124 species. Despite such variation, the overall DNA barcode library possessed diagnostic COI sequences for 98.8% of the taxa. Because a reference library based on Finnish specimens was highly effective in identifying specimens from Austria, we conclude that barcode libraries based on regional sampling can often be effective for a much larger area. Moreover, dispersal ability (poor, good) and distribution patterns (disjunct, fragmented, continuous, migratory) had little impact on levels of intraspecific geographic divergence. Furthermore, the present study revealed that, despite the intensity of past taxonomic work on European Lepidoptera, nearly 20% of the species shared by Austria and Finland require further work to clarify their status. Particularly discordant BIN (Barcode Index Number) cases should be checked to ascertain possible explanatory factors such as incorrect taxonomy, hybridization, introgression, and *Wolbachia* infections.

## Introduction

Animal DNA barcodes are based on a 648 base pair segment near the 5′ terminus of the mitochondrial COI gene (cytochrome *c* oxidase 1). Over the last decade DNA barcoding [1], [2] has quickly gained adoption because of its effectiveness for species identification in many different groups [2], [3], [4], [5]. It has also become widely used in taxonomy as reflected, for example, by national and regional DNA barcoding initiatives in European nations including Austria, Finland, Germany, Norway and Switzerland, efforts contributing to the International Barcode of Life Project (www.ibol.org) which is building barcode coverage for all multicellular species. Although more than 3.4M barcode sequences from more than 350K species of animals, plants and fungi are currently available on BOLD (Barcode of Life Data Systems - www.boldsystems.org), it remains critical to understand the level of barcode coverage required to develop an effective identification system.

Early studies [6] indicated that DNA barcode libraries require comprehensive coverage of known species to enable the identification of newly collected specimens. However, there is also a need to consider the impacts of intraspecific variation on the capacity of barcode libraries to deliver identifications. Since the patterning of DNA barcodes varies among species on local, regional and continental scales, it is not possible to set a standard number of specimens which must be analysed to ensure a functional reference library, especially since sequence distance to the nearest neighbour species varies. However, work on two genera of aquatic beetles has suggested that a detailed understanding of intra- and inter-specific variation is needed to guide species identifications, requiring the analysis of tens of specimens across the range of each species [7]. If these results reflect a general situation, they indicate that the development of an effective DNA barcode reference library will require extensive parameterization.

Lepidoptera have seen more intensive DNA barcode analysis than any other insect order; the 800K records from 78K species currently on BOLD provide coverage for nearly half the world's known species [8]. A comparatively well-established taxonomy, coupled with numerous museum vouchers for most species [9], and a strong taxonomic community mean that lepidopterans are well positioned to provide insights into the parameterization needed to develop an effective DNA barcode reference library. Local and regional barcode studies on Lepidoptera have revealed high identification success [10], [11], [12], but few investigations have examined the extent of geographic variation in DNA barcode sequences for a large assemblage of species. Prior studies on this topic have targeted particular taxonomic groups [13], [14], [15], [16], [17] or species with disjunct arctic-alpine distributions [18]. As a consequence, the present study provides the first test of barcode performance based on analysing a high fraction of the species shared by two distant localities, Finland and Vorarlberg, a province in Austria. It examines 1004 species of Lepidoptera from 58 families, roughly 40% of the species at each locality and a majority of their shared species. It tests the success in identifying one barcode sequence per species from Austria using a larger reference set from Finland. Situations where the reference samples derive from a distant location to newly collected specimens are not uncommon. Our test thus deliberately presumes that there is no data from specimens collected at closer locations, although this is not true for some of the species included in this study.

This study also investigates the effects of this expanded geographic sampling on barcode performance in species with different dispersal abilities and varying distributional patterns. Finally, the concordance between morphological species concepts and those based on COI data is tested by comparing the congruence between current taxonomy and the sequence clusters (BINs) recognized by the Barcode Index Number System [19].

## Materials and Methods

### Study areas and sampling

**Figure 1:**
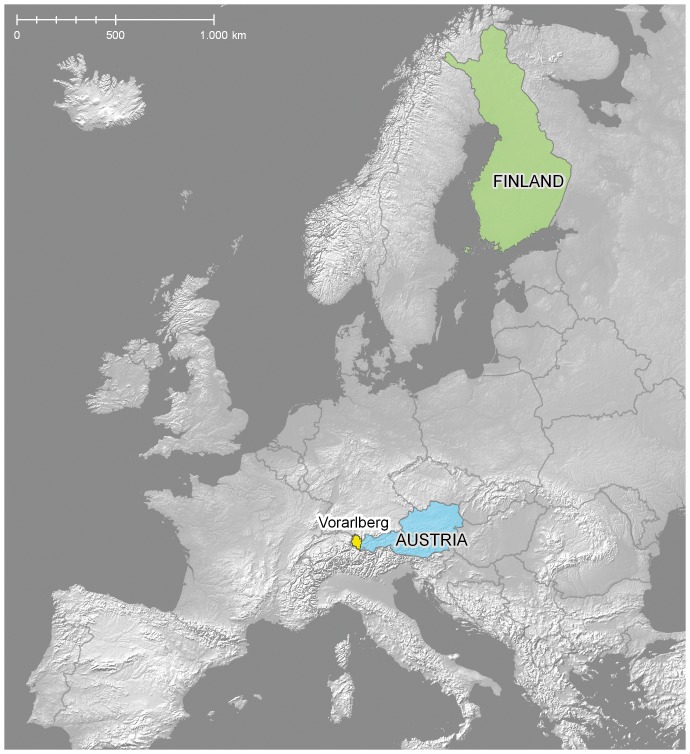
Map showing study areas Finland and Austria/Vorarlberg. Digital base map from Natural Earth. Free vector and raster map data @ http://www.naturalearthdata.com/.

Specimens were analysed from two European localities, Finland and Vorarlberg, the westernmost province of Austria (Fig. 1). Samples from these localities are referenced in later sections of this paper by the acronyms FI and AT with FI+AT representing the pooled sample of Finnish and Austrian specimens. The two localities are separated by a minimum linear distance of 1600 km, but since the Baltic Sea acts as a major dispersal barrier, the shortest land distance (2100 km) provides a more accurate measure of their geographic separation. Vorarlberg and Finland share a considerable number of species of Lepidoptera, reflecting the presence of arctic-alpine, forest, and grassland habitats in both regions. The Finnish fauna includes roughly 2600 species [20] whereas Vorarlberg hosts about 2400 species [21]. An almost complete barcode library for the Lepidoptera of Finland has been assembled with three to four specimens of most species to provide a sense of intraspecific divergence on a national scale. The sampling strategy for Vorarlberg aimed to analyse just one specimen of most species to test the differentiation between these two well-separated regions and keeping in mind one of the major goals of our study - species assignment for a barcode from Austria based upon a reference set from Finland. Specimens for both projects were either collected freshly or were selected from museum collections made in recent decades. No permissions are required to collect Lepidoptera in Finland outside of nature reserves (including private land, except house sites). Endangered species can be collected legally for all species except those specifically protected against collecting. Collecting permits were issued by the Finnish Centre for Economic Development, Transport, and the Environment to MM under permissions VARELY/441/07.01/2012 and LAPELY/275/07.01/2012. The collection of most Lepidoptera is prohibited except for pest species in Vorarlberg. PH obtained the necessary collecting permits for protected species sequenced in this study from the regional museum inatura, Erlebnis Naturschau Dornbirn, Austria through his function as permanent scientific associate of this institution. Information regarding the institutions hosting each specimen, sample and process IDs are available in S1 Appendix. Further details, including voucher images, specimen data including GPS coordinates and collection details are publicly available in BOLD and can be accessed under dx.doi.org/10.5883/DS-FVLEP. This dataset provides barcode coverage for 1004 morphologically defined species from both countries.

### Sequencing

Tissue samples (a dried leg or part of it) from 5777 specimens (4536 Finland, 1241 Austria) belonging to 1004 species were submitted to the Canadian Centre for DNA Barcoding (CCDB, Biodiversity Institute of Ontario, University of Guelph) for sequence analysis. PCR amplification and DNA sequencing was performed at the CCDB following standard high-throughput protocols [22] that can be accessed at http://www.dnabarcoding.ca/pa/ge/research/protocols. Sequences and trace files can be accessed in the public dataset “Finland and Austria: Vorarlberg Lepidoptera” in the Barcode of Life Data Systems BOLD [23]. All data are available from the BOLD database, DOI: dataset dx.doi.org/10.5883/DS-FVLEP. Furthermore all sequences were deposited in Genbank prior to submission, and the accession numbers are provided in S1 Appendix.

### Data Analyses

Pairwise genetic distances between specimens were calculated using the Kimura 2 Parameter (K2P) model, employing the analytical tools on BOLD 3.0. Distances between species are reported as minimum pairwise distances, while intraspecific variation is reported as mean and maximum pairwise distances with maximum intraspecific distance for a species referring to the complete sample (FI+AT). A neighbor-joining (NJ) tree was constructed in MEGA5 [24] using the K2P distance metric to screen for possible misidentifications, contamination events, or other errors (S3 Appendix).

To test the potential impact of differing distributional patterns on intraspecific variation in COI sequences, each species was assigned to one of four categories based on knowledge of its range. The distribution categories included: 1) continuous: species present in all countries situated between Austria and Finland (Germany, Czech Republic, Poland, at least two of the three Baltic states Lithuania, Latvia and Estonia and north-western Russia) and in at least half of the provinces in Germany and Poland with a continuous land connection on a provincial scale between the study areas; 2) fragmented: species present in countries situated between Austria and Finland (Germany, Czech Republic, Poland and at least one of the Baltic states) but with a patchy distribution in these nations; 3) disjunct: species separated by a distribution gap of more than 500 km from Finland to Austria, often involving their absence from either Germany, Czech Republic or Poland; 4) migratory: species regularly migrating over long distances but without overwintering populations in Finland and only exceptionally in Austria. Checklists for species distributions in Germany, Czech Republic, Poland, Latvia, Lithuania, Estonia, and Russia [25], [26], [27], [28], [29], [30], [31] were used to assign each species to a category. Uneven faunistic knowledge for these countries, particularly for microlepidoptera but not for butterflies and skippers [32], has undoubtedly led to some misassignments, with more species included in the disjunct and fragmented categories than would be the case if better data were available.

We also tested the influence of dispersal capacity on the extent of intraspecific barcode variation. Because quantitative data on dispersal capacity are lacking for most species, the classification into two categories (poor or good dispersers) was made at a family level and was limited to 18 of the 58 families, selecting those whose component taxa are likely to show relatively uniform dispersal behaviour (although dispersal capacity undoubtedly varies among species within these families). Families with small species and slow fliers were generally considered poor dispersers while those with larger size and faster flight over longer distances were classed as high dispersers. Although species of Lasiocampidae are large and robust, they were assigned as poor dispersers due to the sedentary lifestyle, especially of females, which should enhance regional divergence of maternally inherited mtDNA.

The effects of distribution and dispersal capacity on divergence were analysed using general linear mixed models in the R package nlme [33]. To account for potential phylogenetic correlations, nested random effects (genus within family) were included in the models. Heteroscedasticity among distribution and dispersal categories was modelled with the varIdent function. Log transformations were applied to the ratio between maximum intraspecific variation and NN distance (effect of distribution) and maximum geographic distance (effect of dispersal capacity). In all cases, statistical significance was established by likelihood ratio tests (LRT), comparing models including and excluding the variable of interest. Statistical analyses and graphs were carried out in R version 3.1.0 [34].

The Barcode Index Number system (BIN) assigns each sequence to a barcode sequence cluster through the application of a Refined Single Linkage (RESL) algorithm, which initially clusters sequences by 2.2% minimum divergence and then refines boundaries by Markov clustering followed by application of the Silhouette criterion [35].

Taxonomy and nomenclature of families, genera and species follows Fauna Europaea (http://www.faunaeur.org/). Further details to specimens see S1 Appendix and S2 Appendix.

## Results

### Overall performance of DNA barcodes

**Figure 2:**
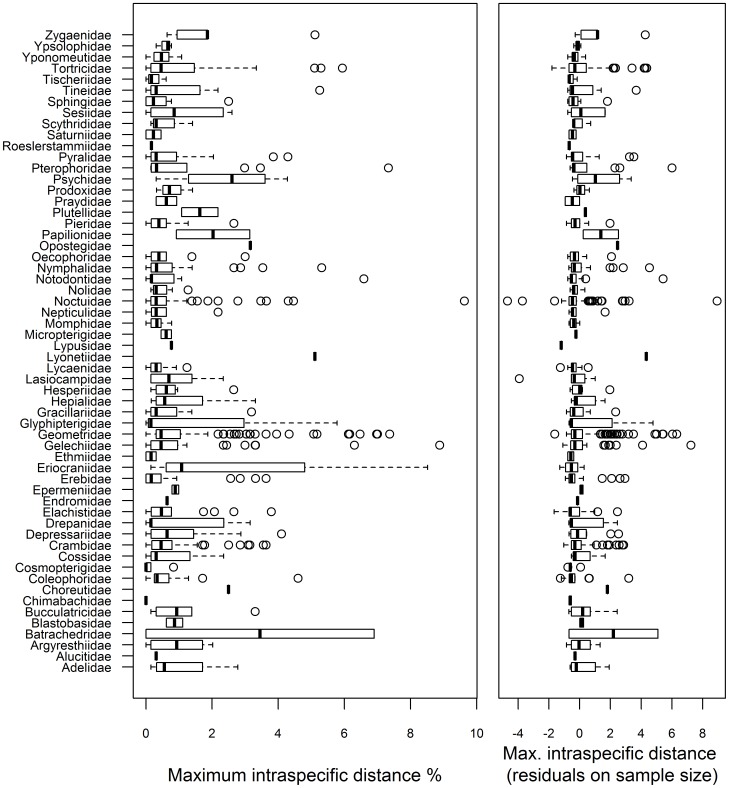
Maximum intraspecific distance for the COI barcode region for the species in each of the 58 families. Left panel: Distributions of maximum intraspecific genetic distance values for each family. Right panel: Residuals from a regression of intraspecific distance against sample size per species, accounting for differences in sample size among species.

**Figure 3:**
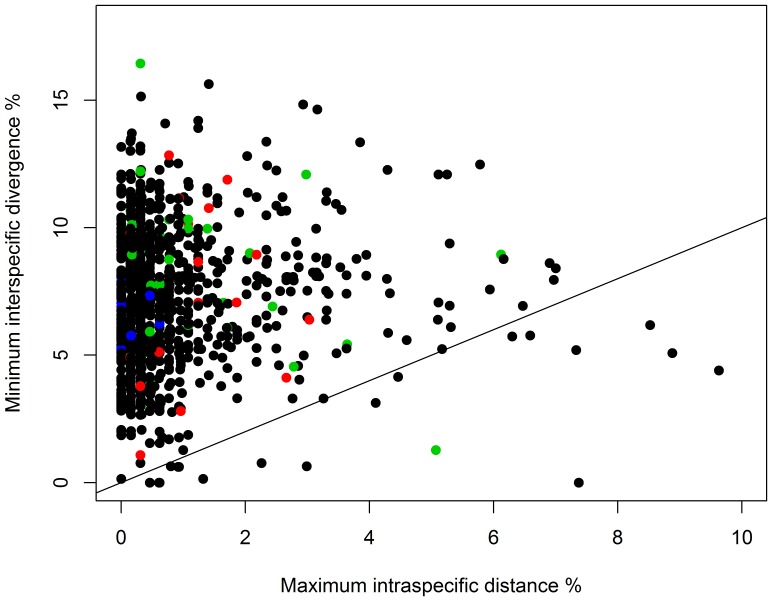
Maximum intraspecific distance (K2P) for COI plotted against minimum distances to the nearest neighbour (NN) species for the combined Austrian and Finnish data (5777 specimens, 1004 species). The diagonal line represents cases where the maximum intraspecific distance equals the minimum distance to the NN. Colours represent different distribution categories (black: continuous; green: fragmented, red: disjunct, blue: migratory).

A full barcode sequence (658 bp) was recovered from 4925 of the 5577 specimens and sequences >500 bp were obtained from all but two specimens (both >474 bp) providing coverage for 1004 species from both localities. Inspection of these data indicated that 992 of the 1004 species (98.8%) possessed a diagnostic COI sequence, irrespective of their geographic origin. Maximum intraspecific distances varied considerably within each family (Fig. 2), but averaged just 1.08%. In fact, specimens of 162 species showed no divergence, while maximum distances were <1% in 773 species (77.0%) and <2% in 880 species (87.6%). Higher intraspecific variation (>2%) was observed in 124 species (12.3%) with a maximum distance of 9.6%, but many of these cases likely reflect overlooked species (see below). DNA barcodes failed to discriminate 12 species which either shared COI sequences or where sequences were intermingled (see below), but some of these cases may represent overlooked synonymies (Fig. 2). The mean minimum distance to the nearest neighbour (NN) species was 7.17%, nearly seven times higher than the mean intraspecific variation (Fig. 3). NN distances varied from a maximum of 16.44% to 0% in the few cases of barcode overlap or barcode-sharing. It was <2% in only 25 of the 1004 species (2.49%) and <1% in just 12 species. All species with low or no interspecific divergence are closely related taxa which are morphologically similar.

### Influence of increased sampling on distance to Nearest Neighbour

We examined the impact of sample size on minimum distance to the Nearest Neighbour (NN).

**Figure 4:**
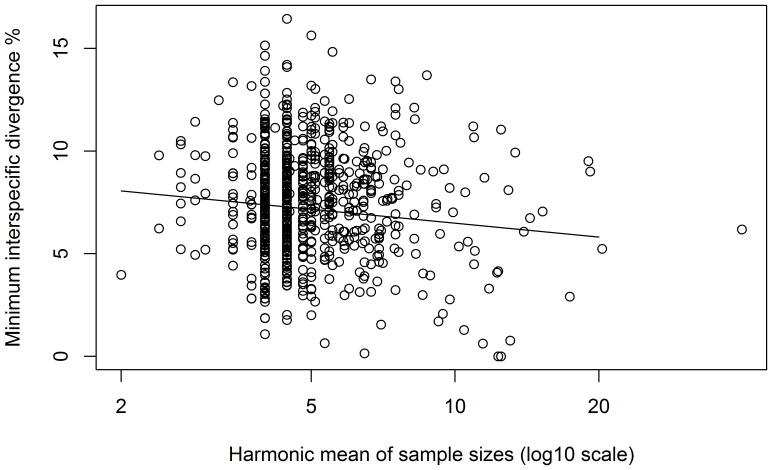
Decrease in the minimum distance (K2P) to nearest neighbour (NN) species with increasing sample size. To account for the impact of small sample sizes, the harmonic mean of the sample size for the focus species and its NN was calculated.

To account for the disproportionate impact of small sample sizes for both the target species and its NN on the minimum distance between species, the harmonic mean of the two sample sizes was calculated. The distance to the NN decreased significantly with increasing sample size (log harmonic mean, base  = 10) (linear regression, β = −2.24, p = 0.0002), but the presence of a barcode gap to the NN was ordinarily unaffected (Fig. 4).

### Influence of increased geographical sampling on intra- and interspecific divergence by distribution types

**Figure 5:**
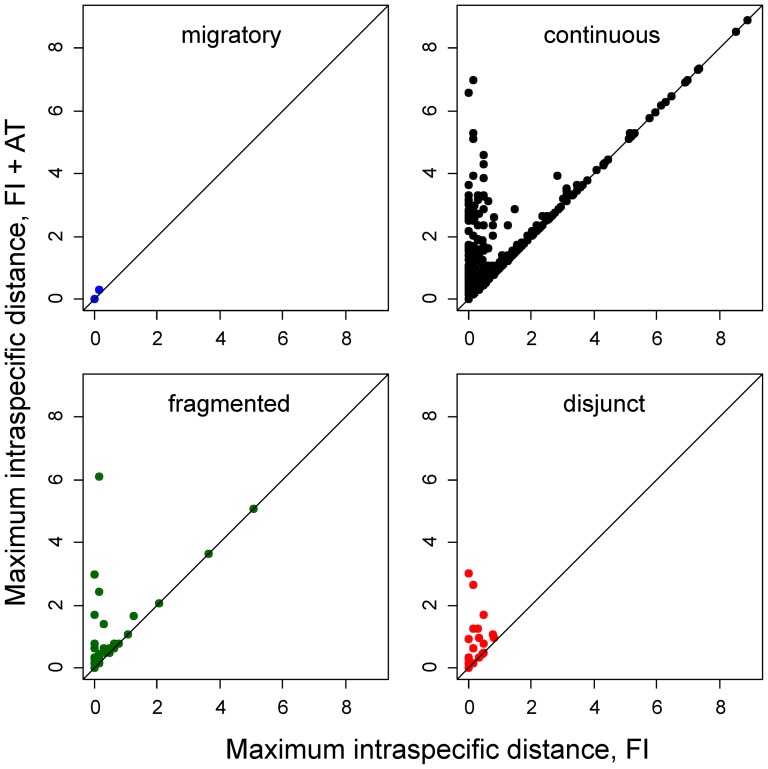
Effect of increased geographic sampling on maximum intraspecific distance among species in the four distribution categories. Maximum intraspecific distance values for the Finnish samples (FI) are plotted against maximum intraspecific distance values after addition of the Austrian samples (FI + AT).

The effect of increased geographic sampling on intraspecific divergence was determined separately for species in the four distribution categories. Increased geographic sampling increased the maximum intraspecific distance (from mean ± SE 0.53±0.033 for FI to 0.83±0.039 for FI+AT; t = −5.86, df = 1977, p-value  = 5.274e-09), particularly for species with fragmented and disjunct distributions although the effect was also evident for species with continuous distributions. Migratory species formed the sole exception (Fig. 5).

**Figure 6:**
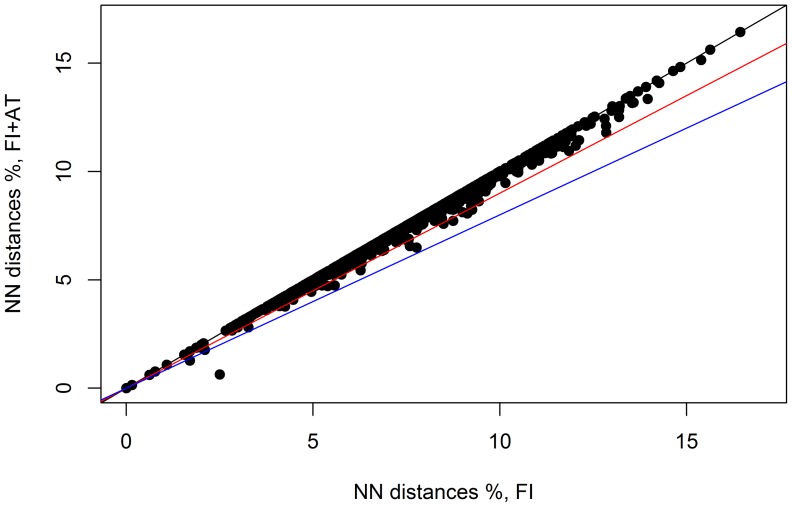
Effect of increased geographical sampling on distance to the nearest neighbor (NN) species. The minimum distance to the NN combining specimens from Austria and Finland (FI + AT) is plotted against the minimum distance to the NN for the Finnish samples (FI). Colored lines mark 0% (black), 10% (red) and 20% (blue) reduction of NN distance due to increased geographic sampling. The outlier point is *Anacampsis blattariella* and *A. populella.*

**Figure 7:**
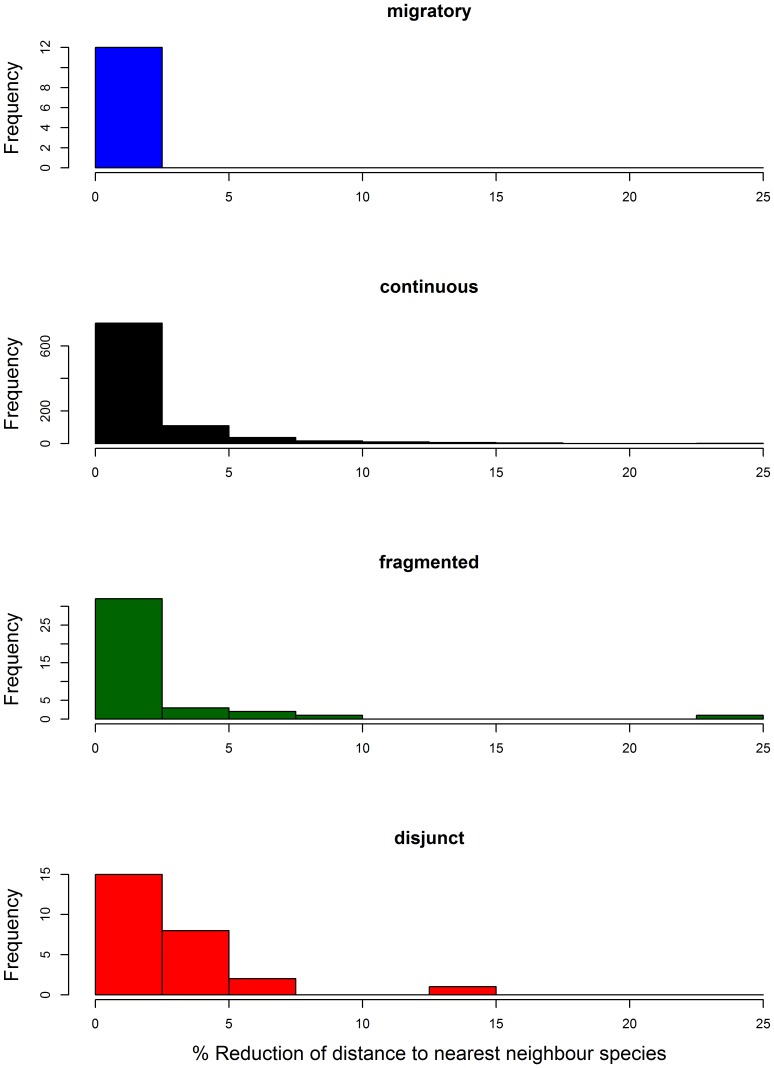
Effect of increased geographical sampling on nearest neighbor (NN) distance for species in each of four distribution categories. Histograms show the % reduction in minimum NN distance after addition of the Austrian samples. This figure excludes one species pair (*Anacampsis blattariella* and *A. populella*) in the continuous distribution category that showed a 75% reduction in NN distance with the addition of the Austrian samples.

The reduction of NN distances due to the expansion of geographic coverage (by combining the AT and FI data) was generally less than 10% (Fig. 6). NN distances for FI + AT were not significantly lower than those for FI alone (mean ± SE 7.27±0.084 for FI and 6.83±0.083 for FI + AT; t = −0.82, df = 2002, p-value  = 0.41). These shifts were largely irrespective of the distribution categories although the decrease in NN distance was most evident for species with disjunct distributions (Fig. 7). However, the NN species for FI was different in 78 cases from the NN species in the FI + AT sample.

### Effect of distribution type and dispersal capacity on intraspecific barcode variation

**Figure 8:**
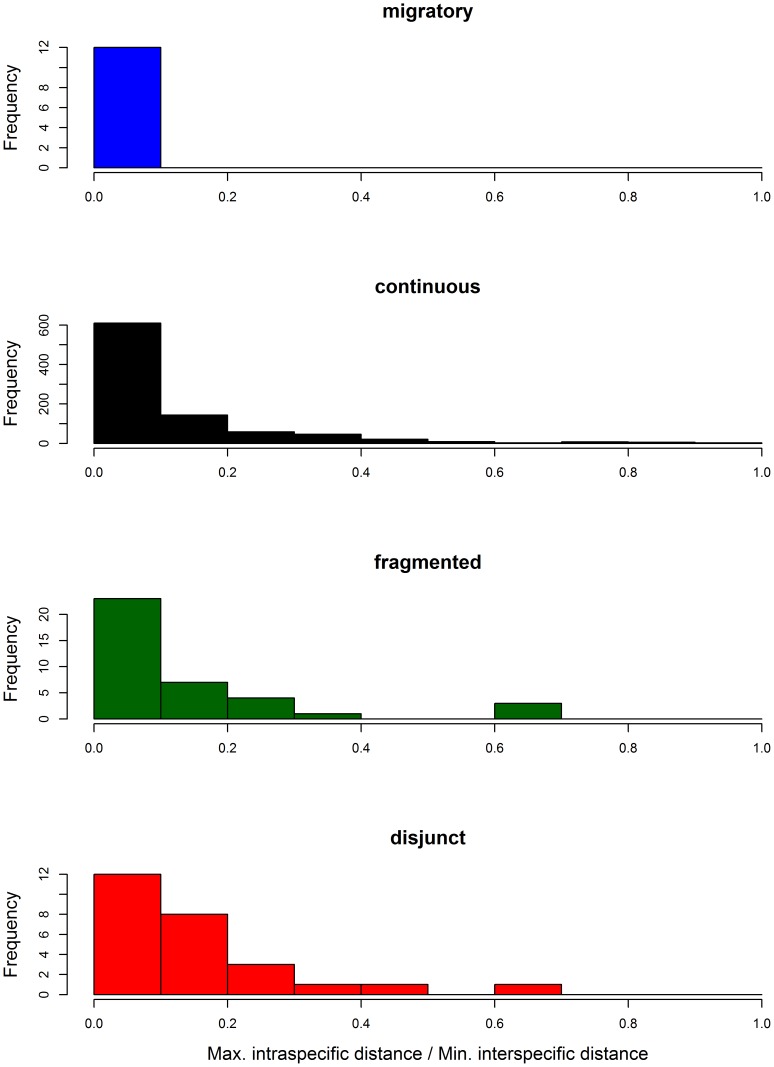
Ratios between maximum intraspecific distance and minimum distance to nearest neighbour (NN) species across distribution types for Lepidoptera from Austria and Finland. Two species pairs with a NN distance of 0% were excluded as well as 15 species whose maximum intraspecific distance was larger than the minimum NN distance.

Distribution category had a significant impact on the ratio between maximum intraspecific variation and NN distance (LRT, model including distribution versus null model: χ^2^ = 52.02, df = 6, p<0.001). However, this was only due to the difference between the migratory species and those in the other categories (p-values in pairwise contrasts <0.01). No significant differences were apparent among species with continuous, fragmented and disjunct distributions (all p>0.05; Fig. 8).

**Figure 9:**
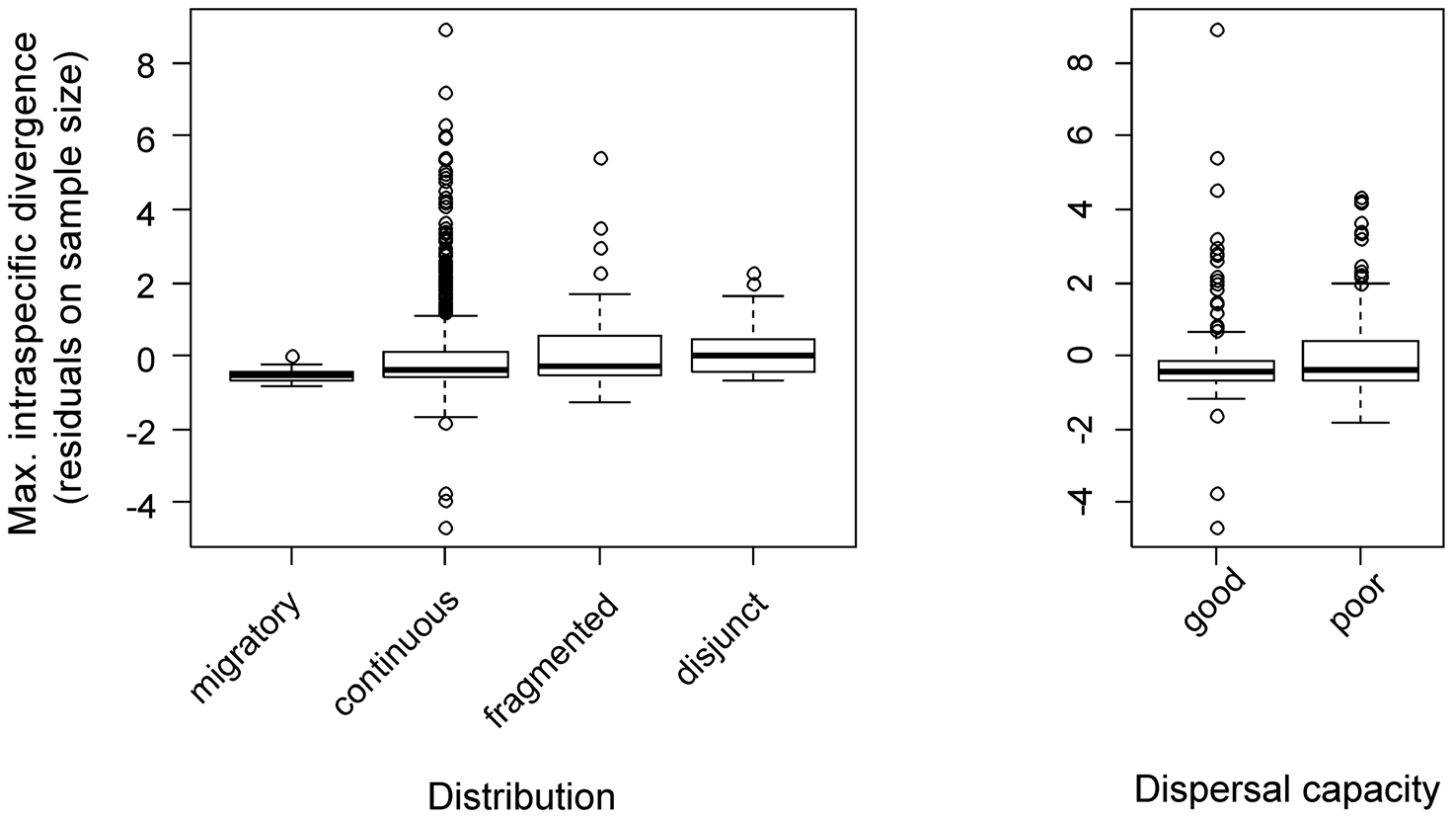
Intraspecific distances in relation to distribution and dispersal capacity. Boxplots show the residuals from a regression of maximum intraspecific distance (FI + AT) against sample size. Residuals are plotted to account for among-species differences in sample size, and thus allow comparisons between distribution categories (left panel) and dispersal capacities (right panel).

Maximum intraspecific distances were lower for migratory species (0.13±0.007) than for species with continuous (0.83±0.039), fragmented (1.08±0.044) or disjunct (0.91±0.026) distributions. There was also a significant effect of distribution category on maximum intraspecific distance (LRT, χ^2^ = 37.72, df = 6, p<0.0001), again reflecting the contrast between migratory species and those in the other distribution categories (p-values in pairwise contrasts <0.01). Species with continuous, fragmented and disjunct distributions showed no significant differences in maximum intraspecific divergence (all p>0.05). To account for the significant effect of variation in sample size per species on intraspecific barcode variation, sample size was included as a co-variate in these analyses (Fig. 9).

**Figure 10:**
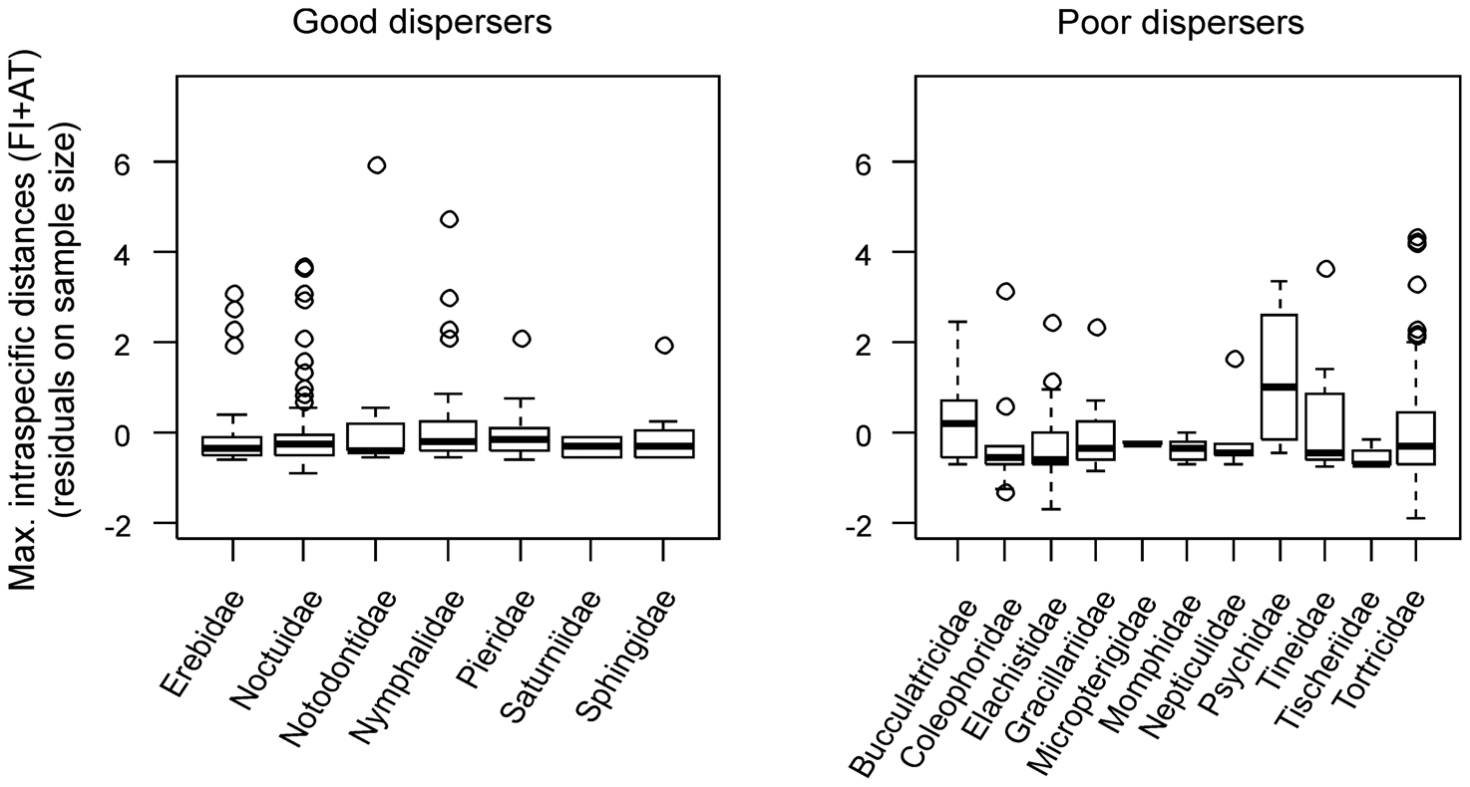
Maximum intraspecific divergence compared across families with good and poor dispersal. Boxplots show the residuals from a regression of maximum intraspecific distance between Austrian and Finnish samples against sample size. Residuals are plotted to account for among-species differences in sample size, and thus allow comparisons between families.

Maximum intraspecific variation (mean ± SE) was less in species with high dispersal (0.54+/−0.058) than those with poor dispersal (0.84+/−0.058) ability. The difference was significant (LRT, χ^2^ = 5.66, df = 1, p = 0.017, sample size included as covariate; Fig. 9) although there was considerable variation among families within the groups (Fig. 10).

### Congruence between BINs and current species taxonomy

**Figure 11:**
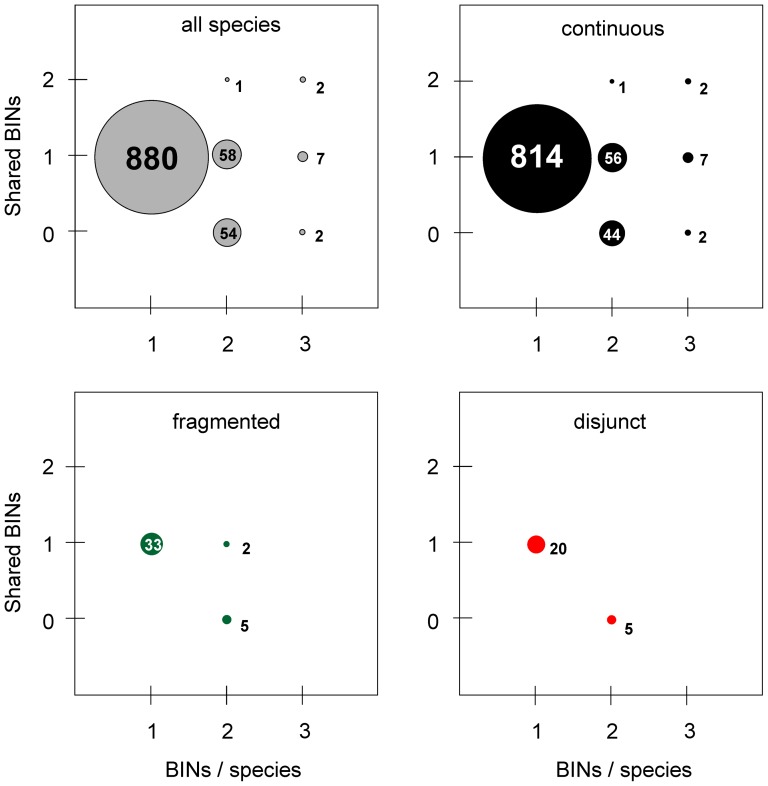
BIN assignment in relation to species taxonomy and distribution category. Circles represent species assigned to one, two or three BINs, of which none, one or two are shared between Finland and Austria. Circle area is proportional to the number of species (number in or beside circle). BIN assignments are shown for all species, and separately for different distribution types. The 12 migratory species were each assigned to one BIN per species (not plotted).

The 5777 sequences obtained in this study were assigned to 1137 BINs. Most species (864, 86.1%) were assigned to a distinct BIN whose member specimens included only representatives of that species, indicating a perfect correspondence between BIN and species boundaries. Another 16 species (1.5%) were assigned to 8 BINs, each comprised of a pair of closely allied species with very similar or identical barcodes (Fig. 11). As such, these taxa represented cases of BIN sharing. Specimens in the remaining species (124; 12.4%) were involved in BIN splits with their member specimens assigned to two or three BINs, producing a total of 263 BINs. Approximately half of these species (56) involved cases where specimens from Austria and Finland were assigned to different BINs, while the other 68 species showed the co-occurrence of two and sometimes three BINs in a single region (Fig. 11). Similar patterns were observed when species with continuous, fragmented and disjunct distributions were analysed separately (Fig. 11). A single species pair (*Anacampsis blattariella* (Hübner, 1796)/*A. populella* (Clerck, 1759)) involved a case of BIN mixture (Ratnasingham & Hebert 2013) where two BINs were detected, but members of one species shared a BIN assignment with the other.

### Deep intraspecific divergence and barcode-sharing

Deep intraspecific divergences (>2%) were detected in 124 of the 1004 (12.3%) species examined in this study. In fact, divergence exceeded 4% in 33 (3.3%) species with representatives in 17 different families (Batrachedridae - 1, Coleophoridae - 1, Elachistidae -1, Eriocraniidae - 1, Gelechiidae - 2, Geometridae - 9, Glyphipterigidae - 1, Lyonetiidae - 1, Noctuidae - 4, Notodontidae - 1, Nymphalidae - 1, Psychidae - 1, Pterophoridae - 1, Pyralidae - 1, Tineidae - 1, Tortricidae - 5, and Zygaenidae - 1). Although maximum intraspecific distances in these taxa ranged from 3.6% to 9.6%, Finnish specimens showed <1% in nine of these species, indicating the possibility of allopatric species.

## Discussion

This study has shown that nearly all species of Lepidoptera (992 of 1004, 98.8%) shared by Finland and Austria possess diagnostic barcodes, confirming results reported in other regions. For example, DNA barcodes distinguished 97% of more than 1000 species from Costa Rica [36] and 99% of 957 species of butterflies and macromoths from southern Germany [37]. Studies on a larger geographical scale have revealed slightly lower resolution with barcode sequences discriminating 93% of 219 species from selected subfamilies of European Geometridae [13] and 90% of 1554 species of Canadian Noctuoidea [17]. Importantly, barcode identification in the present study was highly successful despite the unbalanced comparison between a small test sample of 1–2 specimen per species (Austria) with a larger reference sample (Finland), which might have been expected to biase the results. The present study also revealed that the Barcode Index Number system performed strongly in recognizing sequence clusters (BINs) coincident with currently recognized species boundaries. In fact, 86.1% of the species were assigned to a unique BIN, a higher value than that (70%) reported for Canadian species of Noctuoidea [17]). Although more intensive sampling in our study area, particularly within Austria, and expansion of work across the complete range of each species might reveal further BIN splits, the strong match between BIN assignments and species boundaries affirms that this approach can establish species boundaries that coincide closely with those that would result from detailed taxonomic study.

All efforts to assess the utility of DNA barcodes for species identification involve evaluation of the correspondence between assignments based on DNA barcodes and those arising from taxonomy. Cases of discordance can reflect the inability of DNA barcodes to discriminate certain species or they can result from flaws in the current taxonomy or in its application. Consequently, species sharing barcodes and species with deep intraspecific variation require detailed investigation to understand the responsible factor(s).

This study found just six species pairs which either shared barcodes or possessed very little divergence. While such situations can arise through mitochondrial introgression or incomplete lineage sorting [38], they can also reflect unrecognized synonymy. Our study revealed just one undisputed species pair with regular barcode sharing (*Scoparia ambigualis* (Treitschke, 1829)/*S. basistrigalis* Knaggs, 1866). Two other morphologically distinct species pairs (*Perizoma hydrata* (Treitschke, 1829)*/P. affinitata* (Stephens, 1831), *Apotomis capreana* (Hübner, 1817)/*A. sauciana* (Frölich, 1828)) possessed closely similar barcodes (0.15% and 0.62% interspecific distance respectively), but were separable suggesting that they represent young species pairs although introgression may occur in *Perizoma* [37]). Two other cases of barcode overlap (*Aethes rubigana* (Treitschke, 1830)/*A. cnicana* (Westwood, 1854), *Thera obeliscata* (Hübner, 1787)/*T. variata* (Denis & Schiffermüller, 1775)) involved closely related species pairs. *Thera* is one of the most difficult genera in European Lepidoptera from a taxonomic perspective because of its lack of reliable diagnostic characters. Hausmann et al [37]) also found that *Thera obeliscata* and *T. variata* showed barcode divergence, but that several other species in this genus shared barcodes. To further complicate the situation, specimens of *T. obeliscata* in this study belonged to two barcode clusters with more than 7% divergence, suggesting a cryptic species or introgression from another taxon. The final case of barcode congruence involved a single Austrian specimen of *Anacampsis blattariella* with a barcode sequence close (but not identical) to that of *Anacampsis populella*, a species from which the other specimens showed marked COI divergence.

In contrast to the rarity of barcode sharing, many of the species (124; 12.3%) in this study showed high intraspecific variation (2.0%–9.6%), a greater incidence than in Canadian Noctuoidea (7.7%; [17] or in Bavarian butterflies and macromoths (3.3%; [13]). Because these deep splits can complicate identifications, especially in groups with low NN distances, it is important to consider their origins. Cases of deep intraspecific sequence divergence can arise as a result of *Wolbachia* infections [39] which can provoke selective sweeps of the mitochondrial genome [40], [41]. They can also involve ancestral polymorphisms, often reflecting secondary contact between phylogeographic lineages. Other cases can arise through taxonomic problems such as misidentified specimens or the oversight of cryptic taxa.

Some cases of deep divergence detected in this study were easily resolved because they were found, upon closer inspection, to reflect misidentifications. For example, *Tholera cespitis* (Denis & Schiffermüller, 1775) showed a maximum intraspecific variation of 1.29% within Finland, but 9.63% between two specimens from Austria. Subsequent morphological study revealed that the sequence outlier was actually another species (*Xestia xanthographa* (Denis & Schiffermüller, 1775)) so this case was excluded from the final analysis. However, most other cases of deep divergence involved situations of taxonomic complexity. For example, Finnish specimens of *Neofaculta infernella* (Herrich-Schäffer, 1854) showed up to 8.8% sequence distance, likely reflecting their inclusion of *N. taigana* Ponomarenko, 1998, a species widespread in Siberia and North America [18]. Because this complex includes several other cryptic taxa, final resolution requires a detailed taxonomic investigation of the entire group. Several other cases of deep divergence also involved cryptic diversity. Specimens of *Epiblema grandaevana* (Lienig & Zeller, 1846) from Finland and Austria had a maximum distance of 4.94% versus just 0.61% for Finnish samples. Because the two barcode lineages possess clearly different genitalia, they have been recognized as different species (Segerer et al. in prep.). Specimens of *Euchalcia variabilis* (Piller, 1783) from Finland not only possessed 4.42% sequence divergence from the Austrian sample, but they were morphologically similar to *E. variabilis uralensis* (Eversmann, 1842), a subspecies (or species) described from eastern Russia. Similarly, specimens of *Notodonta dromedarius* (Linnaeus, 1767) showed 6.59% maximum distance between Finland and Austria, a fact correlated with remarkable variation in male genitalia [42]. Another species with deep divergence, *Melitaea athalia* (Rottemburg, 1775), occurred within the contact zone in Austria for *M. athalia athalia* and *M. athalia celadussa* Fruhstorfer, 1910, taxa often viewed as separate species [43]. In a similar fashion, *Eriocrania semipurpurella* (Stephens, 1835) likely involves a complex of cryptic species (Kozlov et al. in prep.). Based on these six examples, it is likely that a considerable fraction of the “species” found to possess deep intraspecific COI divergence in this study actually represent a species pair or trio. Their prevalence in European Lepidoptera, a group which has seen intensive taxonomic study, sends a clear warning of the need for cautious interpretation of barcode data in other groups because, if treated as intraspecific variation, these cases can inflate apparent levels of geographic variation. For example, nearly half the deep intraspecific divergences detected in this study involved differences between populations from Austria and Finland. If many of these cases reflect overlooked taxa, the extent of geographic divergence between Austrian and Finnish populations has been overestimated.

The present study had the primary goal of providing a better understanding of the complications introduced by geographic variation in barcode sequences. The need for this work arose from the differing conclusions reached through prior studies [14] found that increased geographic sampling for 353 species of butterflies in Central Asia did not significantly reduce the performance of DNA barcodes as a tool for species identification. Similarly, [16] observed that while transcontinental sampling generally narrows the barcoding gap (distance to NN) in spiders, this effect was slight and did not hamper reliable identification of species included in the analysis. By contrast, [7] found that broad geographic sampling compromised identification success for two of three genera of diving beetles in Europe. Their results reflected the fact that 23 of 53 species in these genera showed more than 1% intraspecific divergence, sufficient variation to complicate species identifications because NN distances were low, averaging just 3.8%. By contrast, the present study has shown that regional variation in barcode sequences rarely compromised the identification of Lepidoptera species in central and northern Europe. This outcome reflected the fact that most species (77%) showed an intraspecific divergence of less than 1% while NN distances averaged more than 7.0%. Intraspecific distance did increase with geographic distance, but the rise was too small to reduce the effectiveness of DNA barcodes for species identification given the high NN distances. Species distribution patterns also had limited influence on the level of geographic divergence although migratory taxa showed no regional variation, while those with discontinuous distributions did [18]. However, the level of divergence was small, suggesting that gene flow was ordinarily sufficient to prevent local mitochondrial differentiation. There was also a correlation between estimated dispersal capacity and divergence, but this effect was also small, likely indicating that even families classed as poor dispersers are exposed to substantial gene flow. For example, although species of Gracillariidae were rated as poor dispersers because of their small size, certain invasive species in this family, such as *Phyllonorycter issikii* (Kumata, 1963) and *Cameraria ohridella* Deschka & Dimic, 1986, have dispersed across Europe within a decade [44]. Because of this limited geographical variation, a barcode library based on Finnish specimens was highly effective in identifying conspecific individuals from Austria. The decreased identification success with increased geographic sampling encountered by [7] was likely partially due to the fact that diving beetles occupy freshwater habits whose discontinuous nature ensures population fragmentation, favouring high intraspecific differentiation. Secondly, species in the two genera (*Agabus*, *Ilybius*) that were studied appear to have unusually small NN distances in comparison with most other European beetles [45]. As such, these beetles represent a situation in which low NN distances coupled with high intraspecific divergences create the need for an extensively parameterized barcode reference library. The present study indicates that similar complexity is uncommon in the Lepidoptera of central and northern Europe. As such, this study supports the conclusion that barcode reference libraries based on the analysis of just a few specimens of each species will be highly effective. However, the analysis of more specimens often revealed overlooked species, supporting the value of large-scale investigations, especially those that survey diverse regions.

## Supporting Information

S1 Appendix **Accession numbers and BINs.** List of species names, sample-IDs, process-IDs (from BOLD database), GenBank Accession numbers, BINs, collection locality, and Institution storing vouchers.
(PDF)

S2 Appendix **List of families and species, barcoded material per country, barcode gap analysis (intraspecific variation and distance to nearest neighbor), numbers of BINs and shared BINs, distribution pattern and dispersal capacity.**
(PDF)

S3 Appendix **Neighbor Joining Tree.** Neighbor Joining Tree (BOLD-Aligner, Kimura 2 parameter) for 5777 barcoded specimens (>500 bp) representing 1004 species and 1137 BINs.
(PDF)
