# Supplementary material for: Testing DNA Barcode Performance in 1000 Species of European Lepidoptera: Large Geographic Distances Have Small Genetic Impacts
Source: PLoS One. 2014 Dec 26;9(12):e115774. doi: 10.1371/journal.pone.0115774 (PMC4277373; doi:10.1371/journal.pone.0115774)
Supplement: S1 Appendix — Accession numbers and BINs. List of species names, sample-IDs, process-IDs (from BOLD database), GenBank Accession numbers, BINs, collection locality, and Institution storing vouchers. (PDF) [file pone.0115774.s001.pdf]

Appendix S1: Accession numbers and BINs

List of species names, sample-IDs, process-IDs (from BOLD database), GenBank Accession numbers, BINs, collection locality, and Institution storing vouchers.

Species	Sample ID	Process ID	GenBank	BIN	Country	Institution
Abraxas sylvata	MM01459	LEFIA400-10	HM386742	BOLD:AAC2560	Finland	University of Oulu
Abraxas sylvata	TLMF Lep 08122	PHLAV303-12	KM572089	BOLD:AAC2560	Austria	inatura, Dornbirn
Abraxas sylvata	MM01460	LEFIA401-10	HM386743	BOLD:AAC2560	Finland	University of Oulu
Abraxas sylvata	MM10848	LEFIJ165-10	KM573065	BOLD:AAC2560	Finland	University of Oulu
Abraxas sylvata	MM01282	LEFIA249-10	HM386593	BOLD:AAC2560	Finland	University of Oulu
Abraxas sylvata	MM01283	LEFIA250-10	HM386594	BOLD:AAC2560	Finland	University of Oulu
Abrostola triplasia	MM05143	LEFIC870-10	HM872688	BOLD:AAC8312	Finland	University of Oulu
Abrostola triplasia	MM01244	LEFIA215-10	HM396558	BOLD:AAC8312	Finland	University of Oulu
Abrostola triplasia	TLMF Lep 08199	PHLAV380-12	KM572924	BOLD:AAC8312	Austria	inatura, Dornbirn
Abrostola triplasia	MM01245	LEFIA216-10	HM396559	BOLD:ACE9950	Finland	University of Oulu
Acasis viretata	MM10485	LEFIF045-10	HM874756	BOLD:AAC2783	Finland	University of Oulu
Acasis viretata	MM03876	LEFIC356-10	HM872199	BOLD:AAC2783	Finland	University of Oulu
Acasis viretata	MM04670	LEFIC690-10	HM872511	BOLD:AAC2783	Finland	University of Oulu
Acasis viretata	TLMF Lep 07885	PHLAV066-12	KM572782	BOLD:AAC2783	Austria	inatura, Dornbirn
Achlya flavicornis	TLMF Lep 12459	LEATC477-13	KM573038	BOLD:AAD6559	Austria	Tiroler Landesmuseum Ferdinandeum
Achlya flavicornis	TLMF Lep 09499	LEATA082-13	KM572922	BOLD:AAD6559	Austria	Tiroler Landesmuseum Ferdinandeum
Achlya flavicornis	MM00413	LEFIB101-10	HM871008	BOLD:AAD6559	Finland	University of Oulu
Achlya flavicornis	MM01520	LEFIA455-10	HM386795	BOLD:AAD6559	Finland	University of Oulu
Achlya flavicornis	MM10869	LEFIF207-10	HM874901	BOLD:ABX5484	Finland	University of Oulu
Achlya flavicornis	MM01519	LEFIA959-10	GU828665	BOLD:ABX5484	Finland	University of Oulu
Acleris bergmanniana	MM08940	LEFIE393-10	HM874117	BOLD:AAE3614	Finland	University of Oulu
Acleris bergmanniana	MM02005	LEFIB463-10	HM871362	BOLD:AAE3614	Finland	University of Oulu
Acleris bergmanniana	MM02997	LEFIB898-10	HM871775	BOLD:AAE3614	Finland	University of Oulu
Acleris bergmanniana	TLMF Lep 08398	PHLAH579-12	KM572096	BOLD:AAE3614	Austria	inatura, Dornbirn

Acleris ferrugana	MM04204	LEFIC498-10	HM872323	BOLD:AAC3487	Finland	University of Oulu
Acleris ferrugana	MM05306	LEFIC943-10	HM872758	BOLD:AAC3487	Finland	University of Oulu
Acleris ferrugana	TLMF Lep 07932	PHLAV113-12	KM572192	BOLD:AAC3487	Austria	inatura, Dornbirn
Acleris ferrugana	MM05305	LEFIC942-10	HM872757	BOLD:AAC3487	Finland	University of Oulu
Acleris forsskaleana	TLMF Lep 08232	PHLAH413-12	KM572947	BOLD:AAA8796	Austria	inatura, Dornbirn
Acleris forsskaleana	MM02021	LEFIB470-10	HM871369	BOLD:AAA8796	Finland	University of Oulu
Acleris forsskaleana	MM13267	LEFIF875-10	HM875557	BOLD:AAA8796	Finland	University of Oulu
Acleris forsskaleana	MM05001	LEFIC819-10	HM872638	BOLD:AAA8796	Finland	University of Oulu
Acleris hastiana	TLMF Lep 10031	LEATA424-13	KM572565	BOLD:AAA9796	Austria	inatura, Dornbirn
Acleris hastiana	MM11959	LEFIF459-10	HM875144	BOLD:AAA9796	Finland	University of Oulu
Acleris hastiana	MM20722	LEEUA663-11	KM572585	BOLD:AAA9796	Finland	University of Oulu
Acleris hastiana	MM00402	LEFIB093-10	HM871000	BOLD:AAA9796	Finland	University of Oulu
Acleris hastiana	MM00428	LEFIB108-10	HM871015	BOLD:AAA9796	Finland	University of Oulu
Acleris hastiana	MM08206	LEFIE071-10	HM873818	BOLD:AAA9796	Finland	University of Oulu
Acleris hastiana	MM08234	LEFIE078-10	HM873825	BOLD:AAA9796	Finland	University of Oulu
Acleris hastiana	MM00460	LEFIB112-10	HM871019	BOLD:AAA9796	Finland	University of Oulu
Acleris hastiana	MM10283	LEFIE918-10	HM874635	BOLD:AAA9796	Finland	University of Oulu
Acleris hastiana	MM19856	LEEUA448-11	KM573463	BOLD:AAA9796	Finland	University of Oulu
Acleris laterana	MM03483	LEFIC139-10	HM871985	BOLD:AAM1977	Finland	University of Oulu
Acleris laterana	TLMF Lep 08454	PHLAH635-12	KM573124	BOLD:AAM1977	Austria	inatura, Dornbirn
Acleris laterana	MM02029	LEFIB475-10	HM871374	BOLD:AAM1977	Finland	University of Oulu
Acleris laterana	MM00762	LEFIB277-10	HM871179	BOLD:AAM1977	Finland	University of Oulu
Acleris laterana	MM13787	LEFIG095-10	HM875774	BOLD:AAM1977	Finland	University of Oulu
Acleris laterana	MM04832	LEFIC735-10	HM872556	BOLD:AAM1977	Finland	University of Oulu
Acleris maccana	TLMF Lep 02971	PHLAC936-10	JF860404	BOLD:AAA8391	Austria	Tiroler Landesmuseum Ferdinandeum
Acleris maccana	TLMF Lep 02841	PHLAC806-10	JF860323	BOLD:AAA8391	Austria	Tiroler Landesmuseum Ferdinandeum
Acleris maccana	TLMF Lep 02842	PHLAC807-10	JF860324	BOLD:AAA8391	Austria	Tiroler Landesmuseum Ferdinandeum
Acleris maccana	MM13882	LEFIG100-10	HM875780	BOLD:AAA8391	Finland	University of Oulu
Acleris maccana	MM08197	LEFIE066-10	HM873813	BOLD:AAA8391	Finland	University of Oulu
Acleris maccana	MM08298	LEFIE110-10	HM873858	BOLD:AAA8391	Finland	University of Oulu
Acleris maccana	MM08762	LEFIE343-10	HM874067	BOLD:AAA8391	Finland	University of Oulu

Acleris maccana	MM17981	LEFIK406-10	JX034630	BOLD:AAA8391	Finland	University of Oulu
Acleris schalleriana	MM07000	LEFID867-10	HM873624	BOLD:ABZ5454	Finland	University of Oulu
Acleris schalleriana	MM06998	LEFID865-10	HM873622	BOLD:ABZ5454	Finland	University of Oulu
Acleris schalleriana	MM06999	LEFID866-10	HM873623	BOLD:ABZ5454	Finland	University of Oulu
Acleris schalleriana	TLMF Lep 08092	PHLAV273-12	KM572375	BOLD:ABZ5454	Austria	inatura, Dornbirn
Acleris schalleriana	TLMF Lep 07931	PHLAV112-12	KM573244	BOLD:ABZ5454	Austria	inatura, Dornbirn
Acleris shepherdana	MM05363	LEFIC971-10	HM872786	BOLD:AAM2761	Finland	University of Oulu
Acleris shepherdana	TLMF Lep 08418	PHLAH599-12	KM573179	BOLD:AAM2761	Austria	inatura, Dornbirn
Acleris shepherdana	MM08976	LEFIE407-10	HM874131	BOLD:AAM2761	Finland	University of Oulu
Acleris umbrana	MM08930	LEFIE389-10	HM874113	BOLD:AAE7430	Finland	University of Oulu
Acleris umbrana	TLMF Lep 12532	LEATC550-13	KM572894	BOLD:AAE7430	Austria	Tiroler Landesmuseum Ferdinandeum
Acleris umbrana	MM02032	LEFIB476-10	HM871375	BOLD:AAE7430	Finland	University of Oulu
Acleris umbrana	MM00624	LEFIB193-10	HM871097	BOLD:AAE7430	Finland	University of Oulu
Acleris variegana	TLMF Lep 12533	LEATC551-13	KM572262	BOLD:AAB2294	Austria	inatura, Dornbirn
Acleris variegana	MM13178	LEFIF835-10	HM875518	BOLD:ACE3007	Finland	University of Oulu
Acleris variegana	MM13177	LEFIF834-10	HM875517	BOLD:ACE3007	Finland	University of Oulu
Acompsia cinerella	TLMF Lep 00903	PHLAB103-10	HM381475	BOLD:AAD0078	Austria	Tiroler Landesmuseum Ferdinandeum
Acompsia cinerella	TLMF Lep 08103	PHLAV284-12	KM572568	BOLD:AAD0078	Austria	inatura, Dornbirn
Acompsia cinerella	MM05048	LEFIC841-10	HM872659	BOLD:AAD0078	Finland	University of Oulu
Acompsia cinerella	MM13521	LEFIF951-10	HM875632	BOLD:AAD0078	Finland	University of Oulu
Acompsia cinerella	MM02545	LEFIB725-10	HM871603	BOLD:AAD0078	Finland	University of Oulu
Acompsia cinerella	TLMF Lep 00907	PHLAB107-10	HM381479	BOLD:AAD0078	Austria	Tiroler Landesmuseum Ferdinandeum
Acrobasis advenella	MM02362	LEFIB621-10	HM871500	BOLD:AAC0978	Finland	University of Oulu
Acrobasis advenella	MM13009	LEFIF778-10	HM875462	BOLD:AAC0978	Finland	University of Oulu
Acrobasis advenella	TLMF Lep 08471	PHLAH652-12	KM572231	BOLD:AAC0978	Austria	inatura, Dornbirn
Acrobasis advenella	MM03492	LEFIC147-10	HM871993	BOLD:AAC0978	Finland	University of Oulu
Acrobasis advenella	MM20752	LEFIJ1424-12	KM573235	BOLD:AAC0978	Finland	University of Oulu
Acronicta alni	MM23152	LEFIJ1960-13	KM572202	BOLD:AAC3196	Finland	University of Oulu
Acronicta alni	MM23151	LEFIJ1959-13	KM572106	BOLD:AAC3196	Finland	University of Oulu
Acronicta alni	MM22909	LEFIJ1622-13	KM572247	BOLD:AAC3196	Finland	University of Oulu
Acronicta alni	MM22910	LEFIJ1623-13	KM572012	BOLD:AAC3196	Finland	University of Oulu

Acronicta alni	MM22911	LEFIJ1624-13	KM572987	BOLD:AAC3196	Finland	University of Oulu
Acronicta alni	MM22912	LEFIJ1625-13	KM572849	BOLD:AAC3196	Finland	University of Oulu
Acronicta alni	MM22913	LEFIJ1626-13	KM573084	BOLD:AAC3196	Finland	University of Oulu
Acronicta alni	MM22914	LEFIJ1627-13	KM573282	BOLD:AAC3196	Finland	University of Oulu
Acronicta alni	MM18529	LEFII102-10	KM572450	BOLD:AAC3196	Finland	University of Oulu
Acronicta alni	TLMF Lep 04629	PHLAE314-11	JN261552	BOLD:AAC3196	Austria	Tiroler Landesmuseum Ferdinandeum
Acronicta alni	MM23153	LEFIJ1961-13	KM572579	BOLD:AAC3196	Finland	University of Oulu
Acronicta alni	MM23150	LEFIJ1958-13	KM573330	BOLD:AAC3196	Finland	University of Oulu
Acronicta alni	MM11171	LEFIF350-10	HM875035	BOLD:AAC3196	Finland	University of Oulu
Acronicta alni	MM04232	LEFIC519-10	HM872342	BOLD:AAC3196	Finland	University of Oulu
Acronicta auricoma	MM04363	LEFIC581-10	HM872402	BOLD:ACF2281	Finland	University of Oulu
Acronicta auricoma	MM08162	LEFIE050-10	HM873798	BOLD:ACF2281	Finland	University of Oulu
Acronicta auricoma	MM06306	LEFID393-10	HM873190	BOLD:ACF2281	Finland	University of Oulu
Acronicta auricoma	TLMF Lep 04628	PHLAE313-11	JN261551	BOLD:ACF2281	Austria	Tiroler Landesmuseum Ferdinandeum
Acronicta cuspis	MM07390	LEFID926-10	HM873676	BOLD:AAF6047	Finland	University of Oulu
Acronicta cuspis	MM18530	LEFII103-10	JF853342	BOLD:AAF6047	Finland	University of Oulu
Acronicta cuspis	TLMF Lep 10000	LEATA393-13	KM573166	BOLD:AAF6047	Austria	inatura, Dornbirn
Acronicta cuspis	MM06741	LEFID692-10	HM873453	BOLD:AAF6047	Finland	University of Oulu
Acronicta euphorbiae	MM09126	LEFIE431-10	HM874155	BOLD:AAC6993	Finland	University of Oulu
Acronicta euphorbiae	MM09125	LEFIE430-10	HM874154	BOLD:AAC6993	Finland	University of Oulu
Acronicta euphorbiae	TLMF Lep 07576	PHLAG897-12	KM573135	BOLD:AAC6993	Austria	inatura, Dornbirn
Acronicta leporina	MM10910	LEFIF214-10	HQ963162	BOLD:AAC2384	Finland	University of Oulu
Acronicta leporina	MM01525	LEFIA460-10	HM386800	BOLD:AAC2384	Finland	University of Oulu
Acronicta leporina	TLMF Lep 04664	PHLAE349-11	JN261553	BOLD:AAC2384	Austria	Tiroler Landesmuseum Ferdinandeum
Acronicta leporina	MM00856	LEFIB322-10	HM871222	BOLD:AAC2384	Finland	University of Oulu
Acronicta leporina	MM02754	LEFIB817-10	HM871694	BOLD:AAC2384	Finland	University of Oulu
Acronicta psi	MM01526	LEFIA461-10	HM386801	BOLD:AAB3300	Finland	University of Oulu
Acronicta psi	TLMF Lep 04665	PHLAE350-11	JN261554	BOLD:AAB3300	Austria	Tiroler Landesmuseum Ferdinandeum
Acronicta psi	MM12604	LEFIF661-10	HM875345	BOLD:AAB3300	Finland	University of Oulu
Acronicta psi	MM03858	LEFIC344-10	HM872187	BOLD:AAB3300	Finland	University of Oulu
Acronicta psi	MM01544	LEFIA472-10	HM386812	BOLD:AAB3300	Finland	University of Oulu

Acronicta psi	MM04539	LEFIC610-10	HM872431	BOLD:AAB3300	Finland	University of Oulu
Acronicta rumicis	MM04657	LEFIC684-10	HM872505	BOLD:AAC2556	Finland	University of Oulu
Acronicta rumicis	MM01535	LEFIA464-10	HM386804	BOLD:AAC2556	Finland	University of Oulu
Acronicta rumicis	MM03386	LEFIC086-10	HM871954	BOLD:AAC2556	Finland	University of Oulu
Acronicta rumicis	TLMF Lep 07855	PHLAV036-12	KM572108	BOLD:AAC2556	Austria	inatura, Dornbirn
Acronicta rumicis	MM01529	LEFIA965-10	GU828666	BOLD:AAC2556	Finland	University of Oulu
Adaina microdactyla	MM05682	LEFID073-10	HM872884	BOLD:AAD2831	Finland	University of Oulu
Adaina microdactyla	TLMF Lep 08451	PHLAH632-12	KM572553	BOLD:AAD2831	Austria	inatura, Dornbirn
Adaina microdactyla	MM11165	LEFIF346-10	HM875031	BOLD:AAD2831	Finland	University of Oulu
Adaina microdactyla	TLMF Lep 09198	PHLAI636-13	KM572330	BOLD:AAD2831	Austria	Tiroler Landesmuseum Ferdinandeum
Adoxophyes orana	BIOUG04118-B11	GMFIH299-12	KM573373	BOLD:AAD8062	Finland	Biodiversity Institute of Ontario
Adoxophyes orana	MM14376	LEFIG371-10	HM876048	BOLD:AAD8062	Finland	University of Oulu
Adoxophyes orana	BIOUG04118-E07	GMFIQ250-13	KM573388	BOLD:AAD8062	Finland	Biodiversity Institute of Ontario
Adoxophyes orana	BIOUG04118-F06	GMFIS065-13	KM572896	BOLD:AAD8062	Finland	Biodiversity Institute of Ontario
Adoxophyes orana	BIOUG04118-F07	GMFIS066-13	KM572335	BOLD:AAD8062	Finland	Biodiversity Institute of Ontario
Adoxophyes orana	BIOUG04118-C07	GMFII251-12	KM572497	BOLD:AAD8062	Finland	Biodiversity Institute of Ontario
Adoxophyes orana	MM02980	LEFIB892-10	HM871769	BOLD:AAD8062	Finland	University of Oulu
Adoxophyes orana	MM02007	LEFIB464-10	HM871363	BOLD:AAD8062	Finland	University of Oulu
Adoxophyes orana	BIOUG04118-B10	GMFIH298-12	KM572053	BOLD:AAD8062	Finland	Biodiversity Institute of Ontario
Adoxophyes orana	TLMF Lep 08012	PHLAV193-12	KM573184	BOLD:AAD8062	Austria	inatura, Dornbirn
Adoxophyes orana	BIOUG04118-E08	GMFIQ251-13	KM572230	BOLD:AAD8062	Finland	Biodiversity Institute of Ontario
Adscita statices	TLMF Lep 10013	LEATA406-13	KM572158	BOLD:AAD5110	Austria	inatura, Dornbirn
Adscita statices	MM06560	LEFID566-10	HM873331	BOLD:AAD5110	Finland	University of Oulu
Adscita statices	MM06561	LEFID567-10	HM873332	BOLD:AAD5110	Finland	University of Oulu
Adscita statices	MM18630	LEFIL320-10	JN277134	BOLD:AAD5110	Finland	University of Oulu
Adscita statices	MM00313	LEFIB041-10	HM870950	BOLD:AAD5110	Finland	University of Oulu
Adscita statices	MM00312	LEFIA967-10	GU828630	BOLD:AAD5110	Finland	University of Oulu
Adscita statices	MM17364	LEFIJ739-10	JF853797	BOLD:AAD5110	Finland	University of Oulu
Aethes cnicana	MM14221	LEFIG277-10	HM875956	BOLD:AAC2885	Finland	University of Oulu
Aethes cnicana	MM02988	LEFIJ050-10	KM572438	BOLD:AAC2885	Finland	University of Oulu
Aethes cnicana	MM01070	LEFIJ033-10	KM572294	BOLD:AAC2885	Finland	University of Oulu

Aethes cnicana	MM18274	LEFIK699-10	JF854264	BOLD:AAC2885	Finland	University of Oulu
Aethes cnicana	MM02987	LEFIB895-10	HM871772	BOLD:AAC2885	Finland	University of Oulu
Aethes cnicana	MM05509	LEFIJ072-10	JF853423	BOLD:AAC2885	Finland	University of Oulu
Aethes cnicana	MM02986	LEFIB894-10	HM871771	BOLD:AAC2885	Finland	University of Oulu
Aethes cnicana	MM01069	LEFIJ032-10	KM572502	BOLD:AAC2885	Finland	University of Oulu
Aethes cnicana	MM06710	LEFID671-10	HM873432	BOLD:AAC2885	Finland	University of Oulu
Aethes cnicana	MM21121	LEFIJ1261-11	KM573370	BOLD:AAC2885	Finland	University of Oulu
Aethes cnicana	MM18273	LEFIK698-10	JF854263	BOLD:AAC2885	Finland	University of Oulu
Aethes cnicana	MM01072	LEFIA134-10	HM396481	BOLD:AAC2885	Finland	University of Oulu
Aethes cnicana	MM01068	LEFIA969-10	GU828656	BOLD:AAC2885	Finland	University of Oulu
Aethes cnicana	TLMF Lep 07684	PHLAH245-12	KM571982	BOLD:AAC2885	Austria	Tiroler Landesmuseum Ferdinandeum
Aethes cnicana	TLMF Lep 07683	PHLAH244-12	KM573634	BOLD:AAC2885	Austria	Tiroler Landesmuseum Ferdinandeum
Aethes cnicana	TLMF Lep 07720	PHLAH281-12	KM573366	BOLD:AAC2885	Austria	Tiroler Landesmuseum Ferdinandeum
Aethes hartmanniana	MM06142	LEFID271-10	HM873069	BOLD:AAC6718	Finland	University of Oulu
Aethes hartmanniana	MM17278	LEFIJ653-10	JF853748	BOLD:AAC6718	Finland	University of Oulu
Aethes hartmanniana	MM05974	LEFID185-10	HM872991	BOLD:AAC6718	Finland	University of Oulu
Aethes hartmanniana	TLMF Lep 08081	PHLAV262-12	KM572144	BOLD:AAC6718	Austria	inatura, Dornbirn
Aethes hartmanniana	MM14121	LEFIG224-10	HM875904	BOLD:AAC6718	Finland	University of Oulu
Aethes rubigana	MM18271	LEFIK696-10	JF854261	BOLD:AAC2885	Finland	University of Oulu
Aethes rubigana	MM05200	LEFIJ070-10	KM573157	BOLD:AAC2885	Finland	University of Oulu
Aethes rubigana	MM18272	LEFIK697-10	JF854262	BOLD:AAC2885	Finland	University of Oulu
Aethes rubigana	MM12324	LEFIF569-10	HM875254	BOLD:AAC2885	Finland	University of Oulu
Aethes rubigana	TLMF Lep 07682	PHLAH243-12	KM573002	BOLD:AAC2885	Austria	Tiroler Landesmuseum Ferdinandeum
Aethes rubigana	MM08971	LEFIJ129-10	KM573080	BOLD:AAC2885	Finland	University of Oulu
Aethes rubigana	MM05199	LEFIC894-10	HM872711	BOLD:AAC2885	Finland	University of Oulu
Aethes rubigana	MM08969	LEFIE404-10	HM874128	BOLD:AAC2885	Finland	University of Oulu
Aethes rubigana	MM06813	LEFID752-10	HM873509	BOLD:AAC2885	Finland	University of Oulu
Aethes rubigana	MM18270	LEFIK695-10	JF854260	BOLD:AAC2885	Finland	University of Oulu
Aethes rubigana	MM08970	LEFIJ128-10	KM573310	BOLD:AAC2885	Finland	University of Oulu
Aethes rutilana	MM08053	LEFIE002-10	HM873751	BOLD:AAC1488	Finland	University of Oulu
Aethes rutilana	MM11838	LEFIF423-10	HM875108	BOLD:AAC1488	Finland	University of Oulu

Aethes rutilana	MM14379	LEFIG373-10	HM876050	BOLD:AAC1488	Finland	University of Oulu
Aethes rutilana	TLMF Lep 10043	LEATA436-13	KM571999	BOLD:AAC1488	Austria	inatura, Dornbirn
Aethes rutilana	MM11837	LEFIF422-10	HM875107	BOLD:AAC1488	Finland	University of Oulu
Aethes rutilana	MM14588	LEFIG504-10	HM876178	BOLD:AAC1488	Finland	University of Oulu
Aethes rutilana	MM18275	LEFIK700-10	JF854265	BOLD:AAC1488	Finland	University of Oulu
Aethes rutilana	MM18276	LEFIK701-10	JF854266	BOLD:AAC1488	Finland	University of Oulu
Aethes rutilana	MM03430	LEFIC106-10	HQ570297	BOLD:AAC1488	Finland	University of Oulu
Aethes rutilana	MM09745	LEFIA868-10	HM387006	BOLD:AAC1488	Finland	University of Oulu
Aethes rutilana	MM00075	LEFIA004-10	HM396354	BOLD:AAC1488	Finland	University of Oulu
Aethes smeathmanniana	MM13141	LEFIJ1423-12	KM572964	BOLD:AAB1945	Finland	University of Oulu
Aethes smeathmanniana	MM02094	LEFIB504-10	HM871398	BOLD:AAB1945	Finland	University of Oulu
Aethes smeathmanniana	TLMF Lep 09981	PHLAW184-13	KM573562	BOLD:AAB1945	Austria	inatura, Dornbirn
Aethes smeathmanniana	MM13297	LEFIF890-10	HM875572	BOLD:AAB1945	Finland	University of Oulu
Aethes smeathmanniana	MM06319	LEFID400-10	HM873197	BOLD:AAB1945	Finland	University of Oulu
Agapeta hamana	MM06820	LEFID757-10	HM873514	BOLD:AAB9919	Finland	University of Oulu
Agapeta hamana	TLMF Lep 09163	PHLAI601-13	KM572797	BOLD:AAB9919	Austria	Tiroler Landesmuseum Ferdinandeum
Agapeta hamana	MM11780	LEFIF409-10	HM875094	BOLD:AAB9919	Finland	University of Oulu
Agapeta zoegana	MM18268	LEFIK693-10	JF854258	BOLD:AAA6573	Finland	University of Oulu
Agapeta zoegana	MM09584	LEFIE636-10	HM874359	BOLD:AAA6573	Finland	University of Oulu
Agapeta zoegana	MM06900	LEFID813-10	HM873570	BOLD:AAA6573	Finland	University of Oulu
Agapeta zoegana	MM13171	LEFIF830-10	HM875513	BOLD:AAA6573	Finland	University of Oulu
Agapeta zoegana	MM12372	LEFIF577-10	HM875261	BOLD:AAA6573	Finland	University of Oulu
Agapeta zoegana	TLMF Lep 08455	PHLAH636-12	KM572129	BOLD:AAA6575	Austria	inatura, Dornbirn
Aglais io	TLMF Lep 09820	PHLAW023-13	KM573283	BOLD:AAB4921	Austria	Tiroler Landesmuseum Ferdinandeum
Aglais io	MM17153	LEFIJ528-10	JF853647	BOLD:AAB4921	Finland	University of Oulu
Aglais io	MM00383	LEFIB081-10	HM870990	BOLD:AAB4921	Finland	University of Oulu
Aglais io	MM17152	LEFIJ527-10	JF853646	BOLD:AAB4921	Finland	University of Oulu
Aglais urticae	MM17154	LEFIJ529-10	JF853648	BOLD:AAA9652	Finland	University of Oulu
Aglais urticae	TLMF Lep 08563	PHLAH744-12	KM571986	BOLD:AAA9652	Austria	inatura, Dornbirn
Aglais urticae	MM00382	LEFIB080-10	HM870989	BOLD:AAA9652	Finland	University of Oulu
Aglais urticae	MM00381	LEFIB079-10	HM870988	BOLD:AAA9652	Finland	University of Oulu

Aglia tau	MM00321	LEFIA973-10	GU828632	BOLD:AAB6586	Finland	University of Oulu
Aglia tau	MM00017	LEFIB006-10	HM870919	BOLD:AAB6586	Finland	University of Oulu
Aglia tau	TLMF Lep 07823	PHLAV004-12	KM572708	BOLD:AAB6586	Austria	inatura, Dornbirn
Aglia tau	MM10715	LEFIF167-10	HM874861	BOLD:AAB6586	Finland	University of Oulu
Agnathosia mendicella	MM15523	LEFIG659-10	HM876320	BOLD:AAJ7521	Finland	University of Oulu
Agnathosia mendicella	TLMF Lep 08476	PHLAH657-12	KM572586	BOLD:AAJ7521	Austria	inatura, Dornbirn
Agnathosia mendicella	MM18069	LEFIK494-10	JF854101	BOLD:AAJ7521	Finland	University of Oulu
Agnathosia mendicella	MM08638	LEFIA975-10	GU828825	BOLD:AAJ7521	Finland	University of Oulu
Agnathosia mendicella	MM21145	LEFIJ1285-11	KM573416	BOLD:AAJ7521	Finland	University of Oulu
Agonopterix angelicella	MM23128	LEFIJ1936-13	KM573232	BOLD:AAE3381	Finland	University of Oulu
Agonopterix angelicella	MM08211	LEFIE072-10	HM873819	BOLD:AAE3381	Finland	University of Oulu
Agonopterix angelicella	MM23132	LEFIJ1940-13	KM572533	BOLD:AAE3381	Finland	University of Oulu
Agonopterix angelicella	MM22949	LEFIJ1662-13	KM573458	BOLD:AAE3381	Finland	University of Oulu
Agonopterix angelicella	MM23131	LEFIJ1939-13	KM573665	BOLD:AAE3381	Finland	University of Oulu
Agonopterix angelicella	MM02196	LEFIB557-10	HM871439	BOLD:AAE3381	Finland	University of Oulu
Agonopterix angelicella	MM23130	LEFIJ1938-13	KM572822	BOLD:AAE3381	Finland	University of Oulu
Agonopterix angelicella	MM22950	LEFIJ1663-13	KM573577	BOLD:AAE3381	Finland	University of Oulu
Agonopterix angelicella	MM23129	LEFIJ1937-13	KM573321	BOLD:AAE3381	Finland	University of Oulu
Agonopterix angelicella	TLMF Lep 04507	PHLAE382-11	KM573182	BOLD:AAE3381	Austria	Tiroler Landesmuseum Ferdinandeum
Agonopterix angelicella	MM03053	LEFIB921-10	HM871798	BOLD:AAE3381	Finland	University of Oulu
Agonopterix angelicella	MM23124	LEFIJ1932-13	KM572426	BOLD:AAE3381	Finland	University of Oulu
Agonopterix angelicella	MM23133	LEFIJ1941-13	KM572723	BOLD:AAE3381	Finland	University of Oulu
Agonopterix angelicella	MM23125	LEFIJ1933-13	KM572759	BOLD:ABZ4758	Finland	University of Oulu
Agonopterix angelicella	MM23127	LEFIJ1935-13	KM573371	BOLD:ACE0304	Finland	University of Oulu
Agonopterix arenella	MM13348	LEFIF919-10	HM875601	BOLD:AAC6982	Finland	University of Oulu
Agonopterix arenella	CNCLEP00020426	LNEL230-06	KM572554	BOLD:AAC6982	Finland	Canadian National Collection of Insects, Arachnids and Nematodes
Agonopterix arenella	MM04061	LEFIC471-10	HM872304	BOLD:AAC6982	Finland	University of Oulu
Agonopterix arenella	TLMF Lep 07997	PHLAV178-12	KM573205	BOLD:AAC6982	Austria	inatura, Dornbirn
Agonopterix arenella	MM02191	LEFIB556-10	HQ570276	BOLD:AAC6982	Finland	University of Oulu
Agonopterix astrantiae	MM18146	LEFIK571-10	JF854162	BOLD:AAJ1481	Finland	University of Oulu

Agonopterix astrantiae	MM03748	LEFIC283-10	HM872127	BOLD:AAJ1481	Finland	University of Oulu
Agonopterix astrantiae	TLMF Lep 05638	PHLAF468-11	KM572898	BOLD:AAJ1481	Austria	Tiroler Landesmuseum Ferdinandeum
Agonopterix astrantiae	MM03749	LEFIC284-10	HM872128	BOLD:AAJ1481	Finland	University of Oulu
Agonopterix conterminella	MM18889	LEFIL591-10	JF854676	BOLD:AAE7213	Finland	University of Oulu
Agonopterix conterminella	MM02198	LEFIB558-10	HM871440	BOLD:AAE7213	Finland	University of Oulu
Agonopterix conterminella	MM18888	LEFIL590-10	JF854675	BOLD:AAE7213	Finland	University of Oulu
Agonopterix conterminella	MM18890	LEFIL592-10	JF854677	BOLD:AAE7213	Finland	University of Oulu
Agonopterix conterminella	MM18891	LEFIL593-10	JF854678	BOLD:AAE7213	Finland	University of Oulu
Agonopterix conterminella	MM17544	LEFIJ919-10	JF853898	BOLD:AAE7213	Finland	University of Oulu
Agonopterix conterminella	MM17547	LEFIJ922-10	KF808674	BOLD:AAE7213	Finland	University of Oulu
Agonopterix conterminella	MM10277	LEFIE913-10	HM874630	BOLD:AAE7213	Finland	University of Oulu
Agonopterix conterminella	MM15555	LEFIG691-10	HM876348	BOLD:AAE7213	Finland	University of Oulu
Agonopterix conterminella	MM00768	LEFIB280-10	HM871182	BOLD:AAE7213	Finland	University of Oulu
Agonopterix conterminella	TLMF Lep 08446	PHLAH627-12	KF808517	BOLD:AAE7213	Austria	inatura, Dornbirn
Agonopterix heracliana	TLMF Lep 07957	PHLAV138-12	KM573215	BOLD:AAC7415	Austria	inatura, Dornbirn
Agonopterix heracliana	MM01086	LEFIA138-10	HM396484	BOLD:ACF1608	Finland	University of Oulu
Agonopterix heracliana	MM00364	LEFIA976-10	JF818718	BOLD:ACF1608	Finland	University of Oulu
Agonopterix heracliana	MM00418	LEFIB105-10	HM871012	BOLD:ACF1608	Finland	University of Oulu
Agonopterix heracliana	MM01087	LEFIA139-10	HM396485	BOLD:ACF1608	Finland	University of Oulu
Agonopterix heracliana	MM00911	LEFIB359-10	HM871259	BOLD:ACF1608	Finland	University of Oulu
Agonopterix kaekeritziana	MM18997	LEFIL699-10	KM573635	BOLD:AAF7198	Finland	University of Oulu
Agonopterix kaekeritziana	MM14764	LEFIG579-10	HM876250	BOLD:AAF7198	Finland	University of Oulu
Agonopterix kaekeritziana	MM02681	LEFIB783-10	HM871660	BOLD:AAF7198	Finland	University of Oulu
Agonopterix kaekeritziana	MM03648	LEFIC240-10	HM872084	BOLD:AAF7198	Finland	University of Oulu
Agonopterix kaekeritziana	TLMF Lep 07993	PHLAV174-12	KM573607	BOLD:AAF7198	Austria	inatura, Dornbirn
Agonopterix liturosa	TLMF Lep 05639	PHLAF469-11	KM572087	BOLD:AAE7191	Austria	Tiroler Landesmuseum Ferdinandeum
Agonopterix liturosa	MM06815	LEFID754-10	HM873511	BOLD:AAE7191	Finland	University of Oulu
Agonopterix liturosa	MM02201	LEFIB559-10	HM871441	BOLD:AAE7191	Finland	University of Oulu
Agonopterix liturosa	TLMF Lep 07995	PHLAV176-12	KF808563	BOLD:AAE7191	Austria	inatura, Dornbirn
Agonopterix liturosa	MM13226	LEFIF858-10	HM875540	BOLD:AAE7191	Finland	University of Oulu
Agonopterix ocellana	MM00504	LEFIB135-10	HM871041	BOLD:AAF7176	Finland	University of Oulu

Agonopterix ocellana	TLMF Lep 07987	PHLAV168-12	KM572704	BOLD:AAF7176	Austria	inatura, Dornbirn
Agonopterix ocellana	MM00461	LEFIB113-10	HM871020	BOLD:AAF7176	Finland	University of Oulu
Agonopterix ocellana	MM18145	LEFIK570-10	JF854161	BOLD:AAF7176	Finland	University of Oulu
Agriopis marginaria	MM07217	LEFID898-10	HM873648	BOLD:AAC0355	Finland	University of Oulu
Agriopis marginaria	TLMF Lep 08857	PHLAI362-13	KM572244	BOLD:AAC0355	Austria	Tiroler Landesmuseum Ferdinandeum
Agriopis marginaria	MM17858	LEFIK283-10	JF853948	BOLD:AAC0355	Finland	University of Oulu
Agriphila inquinatella	MM13032	LEFIF790-10	HM875474	BOLD:ACN8815	Finland	University of Oulu
Agriphila inquinatella	MM22627	LEFIJ1611-12	KM572064	BOLD:ACN8815	Finland	University of Helsinki
Agriphila inquinatella	TLMF Lep 08730	PHLAH926-12	KM572252	BOLD:ACN8815	Austria	Tiroler Landesmuseum Ferdinandeum
Agriphila inquinatella	MM04957	LEFIC799-10	HM872618	BOLD:ACN8815	Finland	University of Oulu
Agriphila inquinatella	MM01886	LEFIB414-10	HM871313	BOLD:ACN8815	Finland	University of Oulu
Agriphila straminella	TLMF Lep 08470	PHLAH651-12	KM572258	BOLD:AAC0267	Austria	inatura, Dornbirn
Agriphila straminella	MM13031	LEFIF789-10	HM875473	BOLD:AAC0267	Finland	University of Oulu
Agriphila straminella	MM02948	LEFIB876-10	HM871753	BOLD:AAC0268	Finland	University of Oulu
Agriphila straminella	MM01913	LEFIB428-10	HM871327	BOLD:AAC0268	Finland	University of Oulu
Agriphila straminella	MM11806	LEFIJ179-10	KM572343	BOLD:AAC0268	Finland	University of Oulu
Agriphila straminella	MM08487	LEFIJ123-10	KM572310	BOLD:AAC0268	Finland	University of Oulu
Agriphila tristella	MM04963	LEFIC803-10	HM872622	BOLD:AAB9062	Finland	University of Oulu
Agriphila tristella	TLMF Lep 08732	PHLAH928-12	KM572304	BOLD:AAB9062	Austria	Tiroler Landesmuseum Ferdinandeum
Agriphila tristella	MM01912	LEFIB427-10	HM871326	BOLD:AAB9062	Finland	University of Oulu
Agriphila tristella	MM14676	LEFIG538-10	HM876211	BOLD:AAB9062	Finland	University of Oulu
Agrochola circellaris	TLMF Lep 06144	PHLSA689-11	KM572539	BOLD:AAC7613	Austria	Tiroler Landesmuseum Ferdinandeum
Agrochola circellaris	MM06994	LEFID862-10	HM873619	BOLD:AAC7613	Finland	University of Oulu
Agrochola circellaris	MM04842	LEFIC739-10	HM872560	BOLD:AAC7613	Finland	University of Oulu
Agrochola circellaris	MM18040	LEFIK465-10	KM572644	BOLD:AAC7613	Finland	University of Oulu
Agrochola helvola	MM12649	LEFIF672-10	HM875356	BOLD:AAD6457	Finland	University of Oulu
Agrochola helvola	MM08205	LEFIE070-10	HM873817	BOLD:AAD6457	Finland	University of Oulu
Agrochola helvola	MM07738	LEFID942-10	HM873692	BOLD:AAD6457	Finland	University of Oulu
Agrochola helvola	TLMF Lep 09863	PHLAW066-13	KM573162	BOLD:AAD6457	Austria	Tiroler Landesmuseum Ferdinandeum
Agrochola litura	MM12647	LEFIF671-10	HM875355	BOLD:AAC8167	Finland	University of Oulu
Agrochola litura	MM04843	LEFIC740-10	HM872561	BOLD:AAC8167	Finland	University of Oulu

Agrochola litura	MM07739	LEFID943-10	HM873693	BOLD:AAC8167	Finland	University of Oulu
Agrochola litura	TLMF Lep 06196	PHLSA741-11	KM572030	BOLD:AAC8167	Austria	Tiroler Landesmuseum Ferdinandeum
Agrochola lota	MM18041	LEFIK466-10	JF854079	BOLD:AAC0283	Finland	University of Oulu
Agrochola lota	MM03181	LEFIB987-10	HM871859	BOLD:AAC0283	Finland	University of Oulu
Agrochola lota	MM02694	LEFIB788-10	HM871665	BOLD:AAC0283	Finland	University of Oulu
Agrochola lota	TLMF Lep 06143	PHLSA688-11	KM573351	BOLD:AAC0283	Austria	Tiroler Landesmuseum Ferdinandeum
Agrochola macilenta	MM04642	LEFIC675-10	HM872496	BOLD:AAC1555	Finland	University of Oulu
Agrochola macilenta	MM03183	LEFIB989-10	HM871861	BOLD:AAC1555	Finland	University of Oulu
Agrochola macilenta	TLMF Lep 06145	PHLSA690-11	KM573581	BOLD:AAC1555	Austria	Tiroler Landesmuseum Ferdinandeum
Agrochola macilenta	MM03182	LEFIB988-10	HM871860	BOLD:AAC1555	Finland	University of Oulu
Agrochola nitida	TLMF Lep 08759	PHLAI264-13	KM572348	BOLD:AAJ1527	Austria	Tiroler Landesmuseum Ferdinandeum
Agrochola nitida	MM18905	LEFIL607-10	KM572474	BOLD:AAJ1527	Finland	University of Oulu
Agrochola nitida	MM18906	LEFIL608-10	KM571960	BOLD:AAJ1527	Finland	University of Oulu
Agrochola nitida	MM15950	LEFIJ350-10	JF853553	BOLD:AAJ1527	Finland	University of Oulu
Agrotis clavis	MM09495	LEFIE587-10	HM874310	BOLD:AAC2793	Finland	University of Oulu
Agrotis clavis	MM01656	LEFIA562-10	KM573319	BOLD:AAC2793	Finland	University of Oulu
Agrotis clavis	MM04575	LEFIC633-10	HM872454	BOLD:AAC2793	Finland	University of Oulu
Agrotis clavis	TLMF Lep 08490	PHLAH671-12	KM573076	BOLD:AAC2793	Austria	inatura, Dornbirn
Agrotis exclamationis	MM18832	LEFIL534-10	KM573623	BOLD:AAB9113	Finland	University of Oulu
Agrotis exclamationis	MM01657	LEFIA563-10	KM572302	BOLD:AAB9113	Finland	University of Oulu
Agrotis exclamationis	TLMF Lep 07903	PHLAV084-12	KM572288	BOLD:AAB9113	Austria	inatura, Dornbirn
Agrotis exclamationis	MM06642	LEFID624-10	HM873389	BOLD:AAB9113	Finland	University of Oulu
Agrotis exclamationis	MM04568	LEFIC626-10	HM872447	BOLD:AAB9113	Finland	University of Oulu
Agrotis exclamationis	TLMF Lep 08146	PHLAV327-12	KM573507	BOLD:AAB9113	Austria	inatura, Dornbirn
Agrotis ipsilon	MM18000	LEFIK425-10	JF854054	BOLD:AAA3364	Finland	University of Oulu
Agrotis ipsilon	MM17212	LEFIJ587-10	KM573001	BOLD:AAA3364	Finland	University of Oulu
Agrotis ipsilon	MM04210	LEFIC502-10	HM872327	BOLD:AAA3364	Finland	University of Oulu
Agrotis ipsilon	TLMF Lep 07833	PHLAV014-12	KM572047	BOLD:AAA3364	Austria	inatura, Dornbirn
Agrotis segetum	MM11111	LEFIF321-10	HM875006	BOLD:AAC3884	Finland	University of Oulu
Agrotis segetum	TLMF Lep 09862	PHLAW065-13	KM572518	BOLD:AAC3884	Austria	Tiroler Landesmuseum Ferdinandeum
Agrotis segetum	MM15905	LEFIH041-10	HM876672	BOLD:AAC3884	Finland	University of Oulu

Agrotis segetum	MM15904	LEFIH040-10	HM876671	BOLD:AAC3884	Finland	University of Oulu
Alcis repandata	MM01516	LEFIA452-10	HM386793	BOLD:AAA8484	Finland	University of Oulu
Alcis repandata	TLMF Lep 08149	PHLAV330-12	KM572267	BOLD:AAA8484	Austria	inatura, Dornbirn
Alcis repandata	TLMF Lep 07564	PHLAG885-12	KM573414	BOLD:AAA8484	Austria	inatura, Dornbirn
Alcis repandata	MM01517	LEFIA453-10	HM386794	BOLD:AAA8484	Finland	University of Oulu
Alcis repandata	MM02817	LEFIB843-10	HM871720	BOLD:AAA8484	Finland	University of Oulu
Aleimma loeflingiana	MM05204	LEFIC896-10	HM872713	BOLD:AAC3136	Finland	University of Oulu
Aleimma loeflingiana	TLMF Lep 08028	PHLAV209-12	KM572651	BOLD:AAC3136	Austria	inatura, Dornbirn
Aleimma loeflingiana	MM13268	LEFIF876-10	HM875558	BOLD:AAC3136	Finland	University of Oulu
Aleimma loeflingiana	MM05259	LEFIC918-10	HM872735	BOLD:AAC3136	Finland	University of Oulu
Allophyes oxyacanthae	MM04213	LEFIC505-10	HM872330	BOLD:AAC3170	Finland	University of Oulu
Allophyes oxyacanthae	TLMF Lep 06141	PHLSA686-11	KM572257	BOLD:AAC3170	Austria	Tiroler Landesmuseum Ferdinandeum
Allophyes oxyacanthae	MM17378	LEFIJ753-10	KM573703	BOLD:AAC3170	Finland	University of Oulu
Allophyes oxyacanthae	MM01744	LEFIA629-10	HM870878	BOLD:AAC3170	Finland	University of Oulu
Amblyptilia punctidactyla	MM09765	LEFIA888-10	HM387025	BOLD:AAD1903	Finland	University of Oulu
Amblyptilia punctidactyla	MM00489	LEFIB125-10	HM871031	BOLD:AAD1903	Finland	University of Oulu
Amblyptilia punctidactyla	MM02248	LEFIB576-10	HM871457	BOLD:AAD1903	Finland	University of Oulu
Amblyptilia punctidactyla	MM14088	LEFIG204-10	HM875884	BOLD:AAD1904	Finland	University of Oulu
Amblyptilia punctidactyla	TLMF Lep 09890	PHLAW093-13	KM572314	BOLD:ACE9111	Austria	Tiroler Landesmuseum Ferdinandeum
Amblyptilia punctidactyla	TLMF Lep 09907	PHLAW110-13	KM572461	BOLD:ACE9111	Austria	Tiroler Landesmuseum Ferdinandeum
Ammoconia caecimacula	TLMF Lep 08760	PHLAI265-13	KM573433	BOLD:AAE6007	Austria	Tiroler Landesmuseum Ferdinandeum
Ammoconia caecimacula	MM18036	LEFIK461-10	JF854077	BOLD:AAE6007	Finland	University of Oulu
Ammoconia caecimacula	MM15871	LEFIH007-10	HM876643	BOLD:AAE6007	Finland	University of Oulu
Ammoconia caecimacula	MM15872	LEFIH008-10	HM876644	BOLD:AAE6007	Finland	University of Oulu
Amphipoea fucosa	TLMF Lep 08180	PHLAV361-12	KM572789	BOLD:AAB5368	Austria	inatura, Dornbirn
Amphipoea fucosa	TLMF Lep 08181	PHLAV362-12	KM572227	BOLD:AAB5368	Austria	inatura, Dornbirn
Amphipoea fucosa	MM06948	LEFID831-10	HM873588	BOLD:AAB5368	Finland	University of Oulu
Amphipoea fucosa	MM14736	LEFIG577-10	HM876248	BOLD:AAB5368	Finland	University of Oulu
Amphipoea fucosa	MM04878	LEFIC759-10	HM872578	BOLD:AAB5368	Finland	University of Oulu
Amphipoea fucosa	MM01675	LEFIB403-10	HM871302	BOLD:AAB5368	Finland	University of Oulu
Amphipoea fucosa	TLMF Lep 08535	PHLAH716-12	KM573449	BOLD:AAB5368	Austria	inatura, Dornbirn

Amphipoea fucosa	MM04879	LEFIC760-10	HM872579	BOLD:AAB5368	Finland	University of Oulu
Amphipoea fucosa	MM01674	LEFIB402-10	HM871301	BOLD:AAB5368	Finland	University of Oulu
Amphipoea fucosa	MM13878	LEFIA950-10	HM387082	BOLD:AAB5368	Finland	University of Oulu
Amphipoea fucosa	MM12701	LEFIF686-10	HM875370	BOLD:AAB5368	Finland	University of Oulu
Amphipoea fucosa	MM12702	LEFIF687-10	HM875371	BOLD:AAB5368	Finland	University of Oulu
Amphipoea fucosa	MM12703	LEFIF688-10	HM875372	BOLD:AAB5368	Finland	University of Oulu
Amphipoea fucosa	MM08708	LEFIE328-10	HM874054	BOLD:AAB5368	Finland	University of Oulu
Amphipoea fucosa	MM01673	LEFIA579-10	HM870828	BOLD:AAB5368	Finland	University of Oulu
Amphipoea fucosa	MM07394	LEFID928-10	HM873678	BOLD:AAB5368	Finland	University of Oulu
Amphipoea fucosa	MM01672	LEFIA578-10	HM870827	BOLD:AAB5368	Finland	University of Oulu
Amphipoea fucosa	MM04880	LEFIC761-10	HM872580	BOLD:AAB5368	Finland	University of Oulu
Amphipoea fucosa	MM11508	LEFIF360-10	HM875045	BOLD:AAB5368	Finland	University of Oulu
Amphipoea fucosa	MM01676	LEFIB404-10	HM871303	BOLD:AAB5368	Finland	University of Oulu
Amphipoea fucosa	MM08706	LEFIE326-10	HM874052	BOLD:AAB5368	Finland	University of Oulu
Amphipoea fucosa	MM06980	LEFID854-10	HM873611	BOLD:AAB5368	Finland	University of Oulu
Amphipoea fucosa	MM11509	LEFIF361-10	HM875046	BOLD:AAB5368	Finland	University of Oulu
Amphipoea fucosa	MM08707	LEFIE327-10	HM874053	BOLD:AAB5368	Finland	University of Oulu
Amphipoea fucosa	MM07396	LEFID930-10	HM873680	BOLD:AAB5368	Finland	University of Oulu
Amphipoea fucosa	MM06993	LEFID861-10	HM873618	BOLD:AAB5368	Finland	University of Oulu
Amphipoea oculea	MM01670	LEFIA576-10	HM870825	BOLD:AAC7752	Finland	University of Oulu
Amphipoea oculea	MM00819	LEFIB301-10	HM871202	BOLD:AAC7752	Finland	University of Oulu
Amphipoea oculea	MM08324	LEFIE119-10	HM873867	BOLD:AAC7752	Finland	University of Oulu
Amphipoea oculea	MM01671	LEFIA577-10	HM870826	BOLD:AAC7752	Finland	University of Oulu
Amphipoea oculea	TLMF Lep 04673	PHLAE358-11	JN262024	BOLD:AAC7752	Austria	Tiroler Landesmuseum Ferdinandeum
Amphipoea oculea	MM04804	LEFIC728-10	HM872549	BOLD:AAC7752	Finland	University of Oulu
Amphipyra berbera	MM04850	LEFIC746-10	HM872567	BOLD:AAB4108	Finland	University of Oulu
Amphipyra berbera	MM14418	LEFIG396-10	HM876073	BOLD:AAB4108	Finland	University of Oulu
Amphipyra berbera	MM04849	LEFIC745-10	HM872566	BOLD:AAB4108	Finland	University of Oulu
Amphipyra berbera	TLMF Lep 07580	PHLAG901-12	KM572580	BOLD:AAB4108	Austria	inatura, Dornbirn
Amphipyra perflua	MM01162	LEFIA981-10	GU828660	BOLD:AAD2299	Finland	University of Oulu
Amphipyra perflua	MM01163	LEFIA178-10	HM396523	BOLD:AAD2299	Finland	University of Oulu

Amphipyra perflua	TLMF Lep 08504	PHLAH685-12	KM572902	BOLD:AAD2299	Austria	inatura, Dornbirn
Amphipyra perflua	MM04353	LEFIC574-10	HM872395	BOLD:AAD2299	Finland	University of Oulu
Amphipyra perflua	MM01548	LEFIA475-10	HM386815	BOLD:AAD2299	Finland	University of Oulu
Amphipyra perflua	MM12544	LEFIF640-10	HM875324	BOLD:AAD2299	Finland	University of Oulu
Amphipyra pyramidea	MM14419	LEFIG397-10	HM876074	BOLD:AAB4107	Finland	University of Oulu
Amphipyra pyramidea	MM04847	LEFIC744-10	HM872565	BOLD:AAB4107	Finland	University of Oulu
Amphipyra pyramidea	MM18532	LEFII105-10	KM572494	BOLD:AAB4107	Finland	University of Oulu
Amphipyra pyramidea	TLMF Lep 00293	PHLAA253-09	HM381354	BOLD:AAB4107	Austria	Tiroler Landesmuseum Ferdinandeum
Amphipyra tragopoginis	MM01730	LEFIA616-10	HM870865	BOLD:AAB3277	Finland	University of Oulu
Amphipyra tragopoginis	TLMF Lep 08550	PHLAH731-12	KM572804	BOLD:AAB3277	Austria	inatura, Dornbirn
Amphipyra tragopoginis	MM01731	LEFIA617-10	HM870866	BOLD:AAB3277	Finland	University of Oulu
Amphipyra tragopoginis	MM04766	LEFIC716-10	HM872537	BOLD:AAB3277	Finland	University of Oulu
Anacampsis blattariella	MM08998	LEFIE413-10	HM874137	BOLD:AAC9810	Finland	University of Oulu
Anacampsis blattariella	BIOUG04490-F06	GMFIG627-12	KM573325	BOLD:AAC9810	Finland	Biodiversity Institute of Ontario
Anacampsis blattariella	MM03074	LEFIB932-10	HM871809	BOLD:AAC9810	Finland	University of Oulu
Anacampsis blattariella	MM02324	LEFIB607-10	HM871486	BOLD:AAC9810	Finland	University of Oulu
Anacampsis blattariella	BIOUG04490-G01	GMFIS190-13	KM573500	BOLD:AAC9810	Finland	Biodiversity Institute of Ontario
Anacampsis blattariella	MM05036	LEFIC836-10	HM872654	BOLD:AAC9810	Finland	University of Oulu
Anacampsis blattariella	MM08997	LEFIE412-10	HM874136	BOLD:AAC9810	Finland	University of Oulu
Anacampsis blattariella	TLMF Lep 08438	PHLAH619-12	KM573387	BOLD:AAD3256	Austria	inatura, Dornbirn
Anacampsis populella	MM03073	LEFIA983-10	GU828688	BOLD:AAD3256	Finland	University of Oulu
Anacampsis populella	TLMF Lep 08201	PHLAH382-12	KM572116	BOLD:AAD3256	Austria	inatura, Dornbirn
Anacampsis populella	MM03644	LEFIC239-10	HM872083	BOLD:AAD3256	Finland	University of Oulu
Anacampsis populella	MM13400	LEFIF944-10	HM875625	BOLD:AAD3256	Finland	University of Oulu
Anania coronata	MM09813	LEFIE721-10	HM874441	BOLD:ACF0483	Finland	University of Oulu
Anania coronata	MM01868	LEFIA713-10	HM386857	BOLD:ACF0483	Finland	University of Oulu
Anania coronata	MM01869	LEFIA714-10	HM386858	BOLD:ACF0483	Finland	University of Oulu
Anania coronata	TLMF Lep 07980	PHLAV161-12	KM573160	BOLD:ACF0483	Austria	inatura, Dornbirn
Anania crocealis	MM09586	LEFIE638-10	HM874361	BOLD:AAD7537	Finland	University of Oulu
Anania crocealis	MM10392	LEFIE970-10	HM874687	BOLD:AAD7537	Finland	University of Oulu
Anania crocealis	TLMF Lep 07961	PHLAV142-12	KM573712	BOLD:AAD7537	Austria	inatura, Dornbirn

Anania crocealis	MM09585	LEFIE637-10	HM874360	BOLD:AAD7537	Finland	University of Oulu
Anania funebris	MM03248	LEFIC027-10	HM871897	BOLD:AAB4181	Finland	University of Oulu
Anania funebris	MM18400	LEFIK825-10	JN277455	BOLD:ACN5052	Finland	University of Oulu
Anania funebris	MM06366	LEFID435-10	HQ570331	BOLD:ACN5052	Finland	University of Oulu
Anania funebris	TLMF Lep 08433	PHLAH614-12	KM572373	BOLD:ACN5052	Austria	inatura, Dornbirn
Anania fuscalis	MM14652	LEFIG529-10	HM876202	BOLD:AAB9417	Finland	University of Oulu
Anania fuscalis	MM01866	LEFIA711-10	HM386856	BOLD:AAB9417	Finland	University of Oulu
Anania fuscalis	MM18399	LEFIK824-10	JN277454	BOLD:AAB9417	Finland	University of Oulu
Anania fuscalis	MM01867	LEFIA712-10	HQ963157	BOLD:AAB9417	Finland	University of Oulu
Anania fuscalis	TLMF Lep 08011	PHLAV192-12	KM572402	BOLD:AAB9417	Austria	inatura, Dornbirn
Anania hortulata	MM01155	LEFIA174-10	HM396519	BOLD:AAB0989	Finland	University of Oulu
Anania hortulata	MM01154	LEFIA173-10	HM396518	BOLD:AAB0989	Finland	University of Oulu
Anania hortulata	MM15991	LEFIJ386-10	JF853573	BOLD:AAB0989	Finland	University of Oulu
Anania hortulata	MM02937	LEFIB872-10	HM871749	BOLD:AAB0989	Finland	University of Oulu
Anania hortulata	TLMF Lep 08121	PHLAV302-12	KM572071	BOLD:AAB0989	Austria	inatura, Dornbirn
Anania hortulata	MM01851	LEFIA1137-10	GU828675	BOLD:AAB0989	Finland	University of Oulu
Anania lancealis	TLMF Lep 07981	PHLAV162-12	KM572493	BOLD:AAB9418	Austria	inatura, Dornbirn
Anania lancealis	MM17843	LEFIK268-10	KM572899	BOLD:AAB9418	Finland	University of Oulu
Anania lancealis	TLMF Lep 08157	PHLAV338-12	KM572255	BOLD:AAB9418	Austria	inatura, Dornbirn
Anania lancealis	MM17844	LEFIK269-10	KM572783	BOLD:AAB9418	Finland	University of Oulu
Anania lancealis	MM17887	LEFIK312-10	KM572481	BOLD:AAB9418	Finland	University of Oulu
Anania stachydalis	MM06695	LEFID662-10	HM873424	BOLD:AAF3932	Finland	University of Oulu
Anania stachydalis	MM05244	LEFIC910-10	HM872727	BOLD:AAF3932	Finland	University of Oulu
Anania stachydalis	MM14831	LEFIG617-10	HM876283	BOLD:AAF3932	Finland	University of Oulu
Anania stachydalis	TLMF Lep 07517	PHLAG838-12	KM572540	BOLD:AAF3932	Austria	Tiroler Landesmuseum Ferdinandeum
Anania terrealis	MM12978	LEFIF766-10	HM875450	BOLD:AAC9116	Finland	University of Oulu
Anania terrealis	TLMF Lep 08738	PHLAH934-12	KM572790	BOLD:AAC9116	Austria	Tiroler Landesmuseum Ferdinandeum
Anania terrealis	MM02447	LEFIB666-10	HM871544	BOLD:AAC9116	Finland	University of Oulu
Anania terrealis	MM08366	LEFIE137-10	HM873885	BOLD:AAC9116	Finland	University of Oulu
Anania verbascalis	MM21144	LEFIJ1284-11	KM572489	BOLD:AAF3935	Finland	University of Oulu
Anania verbascalis	TLMF Lep 07916	PHLAV097-12	KM572546	BOLD:AAF3935	Austria	inatura, Dornbirn

Anania verbascalis	MM17846	LEFIK271-10	KM573682	BOLD:AAF3935	Finland	University of Oulu
Anania verbascalis	MM17362	LEFIJ737-10	KM573089	BOLD:AAF3935	Finland	University of Oulu
Anania verbascalis	MM17890	LEFIK315-10	KM572425	BOLD:AAF3935	Finland	University of Oulu
Anania verbascalis	MM17841	LEFIK266-10	KM572592	BOLD:AAF3935	Finland	University of Oulu
Anaplectoides prasina	TLMF Lep 08136	PHLAV317-12	KM573497	BOLD:AAA2948	Austria	inatura, Dornbirn
Anaplectoides prasina	MM01639	LEFIA550-10	KM571996	BOLD:AAA2948	Finland	University of Oulu
Anaplectoides prasina	MM01638	LEFIA549-10	KM572060	BOLD:AAA2948	Finland	University of Oulu
Anaplectoides prasina	MM12547	LEFIF641-10	HM875325	BOLD:AAA2948	Finland	University of Oulu
Anaplectoides prasina	MM05089	LEFIC855-10	HM872673	BOLD:AAA2948	Finland	University of Oulu
Anarsia lineatella	TLMF Lep 08204	PHLAH385-12	KM573689	BOLD:ABZ2446	Austria	inatura, Dornbirn
Anarsia lineatella	MM17355	LEFIJ730-10	KM572249	BOLD:ABZ2446	Finland	University of Oulu
Anarsia lineatella	MM05685	LEFID076-10	HM872887	BOLD:ABZ2446	Finland	University of Oulu
Anarsia lineatella	MM21099	LEFIJ1239-11	KM572596	BOLD:ABZ2446	Finland	University of Oulu
Anarsia lineatella	MM06832	LEFID766-10	HM873523	BOLD:ABZ2446	Finland	University of Oulu
Anarsia lineatella	MM05037	LEFIC837-10	HM872655	BOLD:ABZ2446	Finland	University of Oulu
Anarta myrtilli	MM18022	LEFIK447-10	JF854069	BOLD:AAE1452	Finland	University of Oulu
Anarta myrtilli	MM15888	LEFIH024-10	HM876660	BOLD:AAE1452	Finland	University of Oulu
Anarta myrtilli	MM15887	LEFIH023-10	HM876659	BOLD:AAE1452	Finland	University of Oulu
Anarta myrtilli	TLMF Lep 09859	PHLAW062-13	KM572439	BOLD:AAE1452	Austria	Tiroler Landesmuseum Ferdinandeum
Anchinia daphnella	MM01206	LEFIA985-10	JF818720	BOLD:AAE5098	Finland	University of Oulu
Anchinia daphnella	MM01207	LEFIA986-10	JF818721	BOLD:AAE5098	Finland	University of Oulu
Anchinia daphnella	MM08880	LEFIE372-10	HM874096	BOLD:AAE5098	Finland	University of Oulu
Anchinia daphnella	MM09784	LEFIA906-10	HM387042	BOLD:ACE5878	Finland	University of Oulu
Anchinia daphnella	TLMF Lep 08392	PHLAH573-12	KM573176	BOLD:ACE5878	Austria	inatura, Dornbirn
Anchinia daphnella	MM08881	LEFIE373-10	HM874097	BOLD:ACE5878	Finland	University of Oulu
Ancylis apicella	CNCLEP00020414	LNEL218-06	KM572155	BOLD:AAD2001	Finland	Canadian National Collection of Insects, Arachnids and Nematodes
Ancylis apicella	MM03392	LEFIC091-10	HM871959	BOLD:AAD2001	Finland	University of Oulu
Ancylis apicella	MM08975	LEFIE406-10	HM874130	BOLD:AAD2001	Finland	University of Oulu
Ancylis apicella	MM02103	LEFIB510-10	HM871404	BOLD:AAD2001	Finland	University of Oulu
Ancylis apicella	TLMF Lep 09930	PHLAW133-13	KM572951	BOLD:ACE8189	Austria	inatura, Dornbirn

Ancylis badiana	CNCLEP00020415	LNEL219-06	KM573396	BOLD:AAC1552	Finland	Canadian National Collection of Insects, Arachnids and Nematodes
Ancylis badiana	MM22973	LEFIJ1686-13	KM573113	BOLD:ABY4377	Finland	University of Oulu
Ancylis badiana	MM22971	LEFIJ1684-13	KM573379	BOLD:ABY4377	Finland	University of Oulu
Ancylis badiana	MM22976	LEFIJ1689-13	KM573406	BOLD:ABY4377	Finland	University of Oulu
Ancylis badiana	MM00600	LEFIB181-10	HM871085	BOLD:ABY4377	Finland	University of Oulu
Ancylis badiana	MM22975	LEFIJ1688-13	KM572027	BOLD:ABY4377	Finland	University of Oulu
Ancylis badiana	MM22980	LEFIJ1693-13	KM571995	BOLD:ABY4377	Finland	University of Oulu
Ancylis badiana	MM04334	LEFIC557-10	HM872378	BOLD:ABY4377	Finland	University of Oulu
Ancylis badiana	MM22977	LEFIJ1690-13	KM573486	BOLD:ABY4377	Finland	University of Oulu
Ancylis badiana	MM22978	LEFIJ1691-13	KM573183	BOLD:ABY4377	Finland	University of Oulu
Ancylis badiana	MM13285	LEFIF882-10	HM875564	BOLD:ABY4377	Finland	University of Oulu
Ancylis badiana	MM18319	LEFIK744-10	JF854304	BOLD:ABY4377	Finland	University of Oulu
Ancylis badiana	TLMF Lep 08082	PHLAV263-12	KM572219	BOLD:ABY4377	Austria	inatura, Dornbirn
Ancylis badiana	MM11871	LEFIF433-10	HM875118	BOLD:ABY4377	Finland	University of Oulu
Ancylis badiana	MM22972	LEFIJ1685-13	KM572281	BOLD:ABY4377	Finland	University of Oulu
Ancylis badiana	MM22979	LEFIJ1692-13	KM572153	BOLD:ABY4377	Finland	University of Oulu
Ancylis badiana	MM02089	LEFIB503-10	HQ570264	BOLD:ACJ4507	Finland	University of Oulu
Ancylis badiana	MM02087	LEFIB501-10	HM871396	BOLD:ACJ4507	Finland	University of Oulu
Ancylis badiana	MM02144	LEFIB533-10	HM871419	BOLD:ACJ4507	Finland	University of Oulu
Ancylis badiana	TLMF Lep 07470	PHLAG791-12	KM572985	BOLD:ACJ4507	Austria	inatura, Dornbirn
Ancylis badiana	MM02088	LEFIB502-10	HM871397	BOLD:ACJ4507	Finland	University of Oulu
Ancylis badiana	MM18320	LEFIK745-10	JF854305	BOLD:ACJ4507	Finland	University of Oulu
Ancylis badiana	MM08954	LEFIE395-10	HM874119	BOLD:ACJ4507	Finland	University of Oulu
Ancylis badiana	MM08955	LEFIE396-10	HM874120	BOLD:ACJ4507	Finland	University of Oulu
Ancylis diminutana	MM02105	LEFIB511-10	HQ570265	BOLD:AAB6876	Finland	University of Oulu
Ancylis diminutana	MM06536	LEFID548-10	HM873313	BOLD:AAB6876	Finland	University of Oulu
Ancylis diminutana	TLMF Lep 08457	PHLAH638-12	KM572876	BOLD:AAB6876	Austria	inatura, Dornbirn
Ancylis diminutana	MM14123	LEFIG225-10	HM875905	BOLD:AAB6876	Finland	University of Oulu
Ancylis diminutana	MM10031	LEFIE823-10	HM874542	BOLD:AAB6876	Finland	University of Oulu
Ancylis diminutana	TLMF Lep 09950	PHLAW153-13	KM571993	BOLD:AAB6876	Austria	inatura, Dornbirn

Ancylis diminutana	MM02106	LEFIB512-10	HQ570266	BOLD:AAB6876	Finland	University of Oulu
Ancylis laetana	TLMF Lep 09939	PHLAW142-13	KM572168	BOLD:AAE1220	Austria	inatura, Dornbirn
Ancylis laetana	MM13337	LEFIF912-10	HM875594	BOLD:AAE1220	Finland	University of Oulu
Ancylis laetana	MM05480	LEFID033-10	HM872847	BOLD:AAE1220	Finland	University of Oulu
Ancylis laetana	MM20721	LEEUA662-11	KM573213	BOLD:AAE1220	Finland	University of Oulu
Ancylis laetana	MM00532	LEFIB147-10	HM871052	BOLD:AAE1220	Finland	University of Oulu
Ancylis mitterbacheriana	MM14293	LEFIG323-10	HM876002	BOLD:AAB7371	Finland	University of Oulu
Ancylis mitterbacheriana	TLMF Lep 09943	PHLAW146-13	KM572055	BOLD:AAB7371	Austria	inatura, Dornbirn
Ancylis mitterbacheriana	TLMF Lep 07934	PHLAV115-12	KM572299	BOLD:AAB7371	Austria	inatura, Dornbirn
Ancylis mitterbacheriana	MM11193	LEFIF358-10	HM875043	BOLD:AAB7371	Finland	University of Oulu
Ancylis mitterbacheriana	TLMF Lep 07469	PHLAG790-12	KM572555	BOLD:AAB7371	Austria	inatura, Dornbirn
Ancylis mitterbacheriana	MM03844	LEFIC337-10	HM872181	BOLD:AAB7371	Finland	University of Oulu
Ancylis myrtillana	MM00638	LEFIB198-10	HM871102	BOLD:ABZ7857	Finland	University of Oulu
Ancylis myrtillana	TLMF Lep 09959	PHLAW162-13	KM572090	BOLD:ABZ7857	Austria	inatura, Dornbirn
Ancylis myrtillana	MM04335	LEFIC558-10	HM872379	BOLD:ABZ7857	Finland	University of Oulu
Ancylis myrtillana	MM02085	LEFIB500-10	HM871395	BOLD:ABZ7857	Finland	University of Oulu
Ancylis unculana	TLMF Lep 08456	PHLAH637-12	KM573593	BOLD:AAD3929	Austria	inatura, Dornbirn
Ancylis unculana	MM02610	LEFIB756-10	HM871633	BOLD:AAD3930	Finland	University of Oulu
Ancylis unculana	MM02143	LEFIB532-10	HM871418	BOLD:AAD3930	Finland	University of Oulu
Ancylis unculana	MM06460	LEFID501-10	HM873266	BOLD:AAD3930	Finland	University of Oulu
Angerona prunaria	MM06691	LEFID659-10	HM873421	BOLD:AAB7137	Finland	University of Oulu
Angerona prunaria	MM01270	LEFIA237-10	HM386581	BOLD:AAB7137	Finland	University of Oulu
Angerona prunaria	MM01271	LEFIA238-10	HM386582	BOLD:AAB7137	Finland	University of Oulu
Angerona prunaria	TLMF Lep 08153	PHLAV334-12	KM572284	BOLD:AAB7137	Austria	inatura, Dornbirn
Angerona prunaria	MM01477	LEFIA415-10	HM386757	BOLD:AAB7137	Finland	University of Oulu
Anorthoa munda	MM04339	LEFIC562-10	HM872383	BOLD:AAD6193	Finland	University of Oulu
Anorthoa munda	MM04340	LEFIC563-10	HM872384	BOLD:AAD6193	Finland	University of Oulu
Anorthoa munda	TLMF Lep 08854	PHLAI359-13	KM572305	BOLD:AAD6193	Austria	Tiroler Landesmuseum Ferdinandeum
Anorthoa munda	MM04341	LEFIC564-10	HM872385	BOLD:AAD6193	Finland	University of Oulu
Anthocharis cardamines	MM00495	LEFIB129-10	HM871035	BOLD:AAB1133	Finland	University of Oulu
Anthocharis cardamines	MM00500	LEFIB131-10	HM871037	BOLD:AAB1133	Finland	University of Oulu

Anthocharis cardamines	MM17116	LEFIJ491-10	JF853616	BOLD:AAB1133	Finland	University of Oulu
Anthocharis cardamines	TLMF Lep 09806	PHLAW009-13	KM572519	BOLD:AAB1133	Austria	Tiroler Landesmuseum Ferdinandeum
Anthophila fabriciana	MM02342	LEFIB612-10	HM871491	BOLD:AAC8582	Finland	University of Oulu
Anthophila fabriciana	TLMF Lep 03622	PHLAD447-11	JN265169	BOLD:AAC8583	Austria	Tiroler Landesmuseum Ferdinandeum
Anthophila fabriciana	MM03284	LEFIC044-10	HM871914	BOLD:AAC8583	Finland	University of Oulu
Anthophila fabriciana	MM05279	LEFIC928-10	HM872744	BOLD:AAC8583	Finland	University of Oulu
Anticlea derivata	TLMF Lep 07863	PHLAV044-12	KM573467	BOLD:AAD2706	Austria	inatura, Dornbirn
Anticlea derivata	MM13886	LEFIG102-10	HM875782	BOLD:AAD2706	Finland	University of Oulu
Anticlea derivata	MM17435	LEFIJ810-10	JF853835	BOLD:AAD2706	Finland	University of Oulu
Antitype chi	MM04796	LEFIC724-10	HM872545	BOLD:AAE7040	Finland	University of Oulu
Antitype chi	MM02699	LEFIB791-10	HM871668	BOLD:AAE7040	Finland	University of Oulu
Antitype chi	MM12719	LEFIF692-10	HM875376	BOLD:AAE7040	Finland	University of Oulu
Antitype chi	TLMF Lep 06192	PHLSA737-11	KM572005	BOLD:AAE7040	Austria	Tiroler Landesmuseum Ferdinandeum
Apamea crenata	MM01173	LEFIA184-10	HM396529	BOLD:AAC0154	Finland	University of Oulu
Apamea crenata	MM01170	LEFIA991-10	GU828661	BOLD:AAC0154	Finland	University of Oulu
Apamea crenata	MM01172	LEFIA183-10	HM396528	BOLD:AAC0154	Finland	University of Oulu
Apamea crenata	TLMF Lep 07857	PHLAV038-12	KM572259	BOLD:AAC0154	Austria	inatura, Dornbirn
Apamea crenata	MM04727	LEFIC702-10	HM872523	BOLD:AAC0154	Finland	University of Oulu
Apamea furva	TLMF Lep 08770	PHLAI275-13	KM572209	BOLD:AAC7157	Austria	Tiroler Landesmuseum Ferdinandeum
Apamea furva	MM18033	LEFIK458-10	JF854075	BOLD:AAC7157	Finland	University of Oulu
Apamea furva	MM10783	LEFIF191-10	HM874885	BOLD:AAC7157	Finland	University of Oulu
Apamea furva	MM04908	LEFIC777-10	HM872596	BOLD:AAC7157	Finland	University of Oulu
Apamea illyria	MM01594	LEFIA512-10	KM572167	BOLD:AAE6985	Finland	University of Oulu
Apamea illyria	TLMF Lep 06187	PHLSA732-11	KM572125	BOLD:AAE6985	Austria	Tiroler Landesmuseum Ferdinandeum
Apamea illyria	MM12610	LEFIF663-10	HM875347	BOLD:AAE6985	Finland	University of Oulu
Apamea illyria	MM01595	LEFIA513-10	KM573620	BOLD:AAE6985	Finland	University of Oulu
Apamea lateritia	MM01734	LEFIA620-10	HM870869	BOLD:ACF5481	Finland	University of Oulu
Apamea lateritia	MM09935	LEFIE775-10	HM874494	BOLD:ACF5481	Finland	University of Oulu
Apamea lateritia	TLMF Lep 08489	PHLAH670-12	KM572198	BOLD:ACF5481	Austria	inatura, Dornbirn
Apamea lateritia	MM01735	LEFIA621-10	HM870870	BOLD:ACF5481	Finland	University of Oulu
Apamea monoglypha	TLMF Lep 08119	PHLAV300-12	KM572470	BOLD:AAB1551	Austria	inatura, Dornbirn

Apamea monoglypha	MM04354	LEFIC575-10	HM872396	BOLD:AAB1551	Finland	University of Oulu
Apamea monoglypha	MM12653	LEFIF673-10	HM875357	BOLD:ACF3527	Finland	University of Oulu
Apamea monoglypha	MM18034	LEFIK459-10	KM571984	BOLD:ACF3527	Finland	University of Oulu
Apamea rubrirena	MM06042	LEFID212-10	HM873014	BOLD:ACE5999	Finland	University of Oulu
Apamea rubrirena	MM18032	LEFIK457-10	KM573685	BOLD:ACE5999	Finland	University of Oulu
Apamea rubrirena	TLMF Lep 00311	PHLAA271-09	HM425808	BOLD:ACE5999	Austria	Tiroler Landesmuseum Ferdinandeum
Apamea rubrirena	MM12235	LEFIF547-10	HM875232	BOLD:ACE5999	Finland	University of Oulu
Apamea scolopacina	MM01669	LEFIA575-10	HM870824	BOLD:AAC0150	Finland	University of Oulu
Apamea scolopacina	MM04905	LEFIC775-10	HM872594	BOLD:AAC0150	Finland	University of Oulu
Apamea scolopacina	TLMF Lep 08188	PHLAV369-12	KM573126	BOLD:AAC0150	Austria	inatura, Dornbirn
Apamea scolopacina	MM01668	LEFIA574-10	HM870823	BOLD:AAC0150	Finland	University of Oulu
Apamea sublustris	MM17471	LEFIJ846-10	KM572608	BOLD:AAD3064	Finland	University of Oulu
Apamea sublustris	MM14369	LEFIG366-10	HM876043	BOLD:AAD3064	Finland	University of Oulu
Apamea sublustris	MM15877	LEFIH013-10	HM876649	BOLD:AAD3064	Finland	University of Oulu
Apamea sublustris	TLMF Lep 08143	PHLAV324-12	KM571946	BOLD:AAD3064	Austria	inatura, Dornbirn
Apamea unanimis	MM02396	LEFIB638-10	HM871517	BOLD:AAA8789	Finland	University of Oulu
Apamea unanimis	MM01648	LEFIA555-10	KM572496	BOLD:AAA8789	Finland	University of Oulu
Apamea unanimis	TLMF Lep 05617	PHLAF447-11	KM573642	BOLD:AAA8789	Austria	Tiroler Landesmuseum Ferdinandeum
Apamea unanimis	MM18031	LEFIK456-10	KM572893	BOLD:AAA8789	Finland	University of Oulu
Apatura ilia	MM17385	LEFIJ760-10	JF853804	BOLD:AAB7458	Finland	University of Oulu
Apatura ilia	MM17160	LEFIJ535-10	JF853652	BOLD:AAB7458	Finland	University of Oulu
Apatura ilia	MM05916	LEFID153-10	HM872960	BOLD:AAB7458	Finland	University of Oulu
Apatura ilia	TLMF Lep 09853	PHLAW056-13	KM573234	BOLD:AAB7458	Austria	Tiroler Landesmuseum Ferdinandeum
Apatura iris	MM17384	LEFIJ759-10	JF853803	BOLD:AAB7905	Finland	University of Oulu
Apatura iris	MM05914	LEFID151-10	HM872958	BOLD:AAB7905	Finland	University of Oulu
Apatura iris	TLMF Lep 10021	LEATA414-13	KM572766	BOLD:AAB7905	Austria	inatura, Dornbirn
Apatura iris	MM00353	LEFIB065-10	HM870974	BOLD:AAB7905	Finland	University of Oulu
Apeira syringaria	TLMF Lep 10020	LEATA413-13	KM572881	BOLD:AAD4663	Austria	inatura, Dornbirn
Apeira syringaria	MM01503	LEFIA439-10	HM386780	BOLD:AAD4663	Finland	University of Oulu
Apeira syringaria	MM02397	LEFIB639-10	HM871518	BOLD:AAD4663	Finland	University of Oulu
Aphantopus hyperantus	TLMF Lep 05821	PHLAF651-11	KM572807	BOLD:AAB1940	Austria	Tiroler Landesmuseum Ferdinandeum

Aphantopus hyperantus	MM03238	LEFIC025-10	HM871895	BOLD:AAB1940	Finland	University of Oulu
Aphantopus hyperantus	MM00045	LEFIA993-10	KM573204	BOLD:AAB1940	Finland	University of Oulu
Aphantopus hyperantus	MM17184	LEFIJ559-10	JF853674	BOLD:ACF2774	Finland	University of Oulu
Aphelia paleana	MM03601	LEFIC213-10	HM872057	BOLD:AAB6818	Finland	University of Oulu
Aphelia paleana	MM03496	LEFIC151-10	HM871997	BOLD:AAB6818	Finland	University of Oulu
Aphelia paleana	MM18261	LEFIK686-10	JF854252	BOLD:AAB6818	Finland	University of Oulu
Aphelia paleana	MM04997	LEFIC816-10	HM872635	BOLD:AAB6818	Finland	University of Oulu
Aphelia paleana	MM11775	LEFIF406-10	HM875091	BOLD:AAB6818	Finland	University of Oulu
Aphelia paleana	MM13167	LEFIF828-10	HM875511	BOLD:ACE9674	Finland	University of Oulu
Aphelia paleana	MM13168	LEFIF829-10	HM875512	BOLD:ACE9674	Finland	University of Oulu
Aphelia paleana	MM00345	LEFIB060-10	HM870969	BOLD:ACE9674	Finland	University of Oulu
Aphelia paleana	MM00118	LEFIA994-10	GU828605	BOLD:ACE9674	Finland	University of Oulu
Aphelia paleana	MM01902	LEFIB422-10	HM871321	BOLD:ACE9675	Finland	University of Oulu
Aphelia paleana	TLMF Lep 08430	PHLAH611-12	KM573518	BOLD:ACE9675	Austria	inatura, Dornbirn
Aphelia paleana	MM01901	LEFIB421-10	HM871320	BOLD:ACE9675	Finland	University of Oulu
Aphelia paleana	MM18260	LEFIK685-10	KM573402	BOLD:ACE9675	Finland	University of Oulu
Aphelia paleana	MM18262	LEFIK687-10	JF854253	BOLD:ACE9675	Finland	University of Oulu
Aphelia paleana	TLMF Lep 07524	PHLAG845-12	KM572695	BOLD:ACE9675	Austria	Tiroler Landesmuseum Ferdinandeum
Aphelia paleana	MM11776	LEFIF407-10	HM875092	BOLD:ACE9675	Finland	University of Oulu
Aphelia paleana	MM08875	LEFIE371-10	HM874095	BOLD:ACE9675	Finland	University of Oulu
Aphelia unitana	TLMF Lep 04681	PHLAE366-11	JN286482	BOLD:AAB6819	Austria	Tiroler Landesmuseum Ferdinandeum
Aphelia unitana	TLMF Lep 00915	PHLAB115-10	HM381487	BOLD:AAB6819	Austria	Tiroler Landesmuseum Ferdinandeum
Aphelia unitana	MM18258	LEFIK683-10	JF854250	BOLD:AAD2152	Finland	University of Oulu
Aphelia unitana	MM18259	LEFIK684-10	JF854251	BOLD:AAD2152	Finland	University of Oulu
Aphelia unitana	MM15657	LEFIG793-10	HM876445	BOLD:AAD2152	Finland	University of Oulu
Aphelia unitana	MM15656	LEFIG792-10	HM876444	BOLD:AAD2152	Finland	University of Oulu
Aphelia unitana	MM00038	LEFIB016-10	HM870929	BOLD:AAD2152	Finland	University of Oulu
Aphelia unitana	MM06129	LEFID264-10	HM873062	BOLD:AAD2152	Finland	University of Oulu
Aphelia unitana	MM15655	LEFIG791-10	HM876443	BOLD:AAD2152	Finland	University of Oulu
Aphelia viburniana	MM06779	LEFID723-10	HM873480	BOLD:ACE6890	Finland	University of Oulu
Aphelia viburniana	MM14332	LEFIG344-10	HM876021	BOLD:ACE6890	Finland	University of Oulu

Aphelia viburniana	MM06502	LEFID523-10	HM873288	BOLD:ACE6890	Finland	University of Oulu
Aphelia viburniana	MM03785	LEFIC301-10	HM872145	BOLD:ACE6890	Finland	University of Oulu
Aphelia viburniana	MM17348	LEFIJ723-10	KM573367	BOLD:ACE6891	Finland	University of Oulu
Aphelia viburniana	MM15990	LEFIJ385-10	JF853572	BOLD:ACE6891	Finland	University of Oulu
Aphelia viburniana	TLMF Lep 08023	PHLAV204-12	KM572760	BOLD:ACE6891	Austria	inatura, Dornbirn
Aphomia sociella	MM01875	LEFIA995-10	GU828676	BOLD:AAA5606	Finland	University of Oulu
Aphomia sociella	TLMF Lep 09884	PHLAW087-13	KM573659	BOLD:AAA5606	Austria	Tiroler Landesmuseum Ferdinandeum
Aphomia sociella	CNCLEP00020421	LNEL225-06	KM573245	BOLD:AAA5606	Finland	Canadian National Collection of Insects, Arachnids and Nematodes
Aphomia sociella	MM09681	LEFIE699-10	HM874420	BOLD:AAA5606	Finland	University of Oulu
Aphomia sociella	MM04040	LEFIC457-10	HM872291	BOLD:AAA5606	Finland	University of Oulu
Aplocera praeformata	MM01468	LEFIA407-10	HM386749	BOLD:AAC3875	Finland	University of Oulu
Aplocera praeformata	MM04928	LEFIC790-10	HM872609	BOLD:AAC3875	Finland	University of Oulu
Aplocera praeformata	MM12783	LEFIF720-10	HM875404	BOLD:AAC3875	Finland	University of Oulu
Aplocera praeformata	TLMF Lep 08495	PHLAH676-12	KM572424	BOLD:AAC3875	Austria	inatura, Dornbirn
Aporia crataegi	MM17131	LEFIJ506-10	JF853629	BOLD:AAA8773	Finland	University of Oulu
Aporia crataegi	MM17130	LEFIJ505-10	JF853628	BOLD:AAA8773	Finland	University of Oulu
Aporia crataegi	MM00095	LEFIA024-10	HM396374	BOLD:AAA8773	Finland	University of Oulu
Aporia crataegi	TLMF Lep 09098	PHLAI536-13	KM573218	BOLD:AAA8773	Austria	Tiroler Landesmuseum Ferdinandeum
Apotomis capreana	MM06708	LEFID670-10	HM873431	BOLD:ABZ6958	Finland	University of Oulu
Apotomis capreana	MM08882	LEFIE374-10	HM874098	BOLD:ABZ6958	Finland	University of Oulu
Apotomis capreana	MM01991	LEFIB457-10	HM871356	BOLD:ABZ6958	Finland	University of Oulu
Apotomis capreana	TLMF Lep 10030	LEATA423-13	KM572412	BOLD:ABZ6958	Austria	inatura, Dornbirn
Apotomis capreana	MM03495	LEFIC150-10	HM871996	BOLD:ABZ6958	Finland	University of Oulu
Apotomis capreana	MM04999	LEFIC818-10	HM872637	BOLD:ABZ6958	Finland	University of Oulu
Apotomis capreana	TLMF Lep 08079	PHLAV260-12	KM572369	BOLD:ABZ6958	Austria	inatura, Dornbirn
Apotomis capreana	MM06593	LEFID585-10	HM873350	BOLD:ABZ6958	Finland	University of Oulu
Apotomis infida	TLMF Lep 09393	PHLAI831-13	KM572159	BOLD:ACF3687	Austria	Tiroler Landesmuseum Ferdinandeum
Apotomis infida	MM18303	LEFIK728-10	JF854290	BOLD:ACF3687	Finland	University of Oulu
Apotomis infida	MM02968	LEFIB883-10	HM871760	BOLD:ACF3687	Finland	University of Oulu
Apotomis infida	MM01981	LEFIB453-10	HM871352	BOLD:ACF3687	Finland	University of Oulu

Apotomis infida	MM01982	LEFIB454-10	HM871353	BOLD:ACF3687	Finland	University of Oulu
Apotomis infida	MM01983	LEFIB455-10	HM871354	BOLD:ACF3687	Finland	University of Oulu
Apotomis sauciana	MM15698	LEFIG834-10	HM876483	BOLD:ABZ6958	Finland	University of Oulu
Apotomis sauciana	MM04183	LEFIA829-10	HM386969	BOLD:ABZ6958	Finland	University of Oulu
Apotomis sauciana	MM04184	LEFIA830-10	HM386970	BOLD:ABZ6958	Finland	University of Oulu
Apotomis sauciana	MM04187	LEFIJ064-10	KM573027	BOLD:ABZ6958	Finland	University of Oulu
Apotomis sauciana	MM18306	LEFIK731-10	JF854292	BOLD:ABZ6958	Finland	University of Oulu
Apotomis sauciana	MM18308	LEFIK733-10	JF854294	BOLD:ABZ6958	Finland	University of Oulu
Apotomis sauciana	MM18640	LEFIL330-10	JN274980	BOLD:ABZ6958	Finland	University of Oulu
Apotomis sauciana	MM04182	LEFIA828-10	HM386968	BOLD:ABZ6958	Finland	University of Oulu
Apotomis sauciana	TLMF Lep 03623	PHLAD448-11	JN275014	BOLD:ABZ6958	Austria	Tiroler Landesmuseum Ferdinandeum
Apotomis sauciana	MM06283	LEFID373-10	HM873170	BOLD:ABZ6958	Finland	University of Oulu
Apotomis sauciana	MM06282	LEFID372-10	HM873169	BOLD:ABZ6958	Finland	University of Oulu
Apotomis sauciana	MM15697	LEFIG833-10	HM876482	BOLD:ABZ6958	Finland	University of Oulu
Apotomis sauciana	MM15696	LEFIG832-10	HM876481	BOLD:ABZ6958	Finland	University of Oulu
Apotomis sauciana	MM21065	LEFIJ1205-11	KM573092	BOLD:ABZ6958	Finland	University of Oulu
Apotomis sauciana	MM21063	LEFIJ1203-11	KM573568	BOLD:ABZ6958	Finland	University of Oulu
Apotomis sauciana	MM08347	LEFIE131-10	HM873879	BOLD:ABZ6958	Finland	University of Oulu
Apotomis sauciana	MM21064	LEFIJ1204-11	KM572574	BOLD:ABZ6958	Finland	University of Oulu
Apotomis sauciana	MM04185	LEFIJ062-10	KM572270	BOLD:ABZ6958	Finland	University of Oulu
Apotomis sauciana	MM06823	LEFID759-10	HM873516	BOLD:ABZ6958	Finland	University of Oulu
Apotomis sauciana	MM04186	LEFIJ063-10	KM572126	BOLD:ABZ6958	Finland	University of Oulu
Aproaerema anthyllidella	MM06379	LEFID445-10	HQ570341	BOLD:AAD2266	Finland	University of Oulu
Aproaerema anthyllidella	MM06380	LEFID446-10	HQ570342	BOLD:AAD2266	Finland	University of Oulu
Aproaerema anthyllidella	MM04196	LEFIC492-10	HQ570315	BOLD:AAD2266	Finland	University of Oulu
Aproaerema anthyllidella	MM17255	LEFIJ630-10	JF853728	BOLD:AAD2266	Finland	University of Oulu
Aproaerema anthyllidella	TLMF Lep 08055	PHLAV236-12	KM572839	BOLD:AAD2266	Austria	inatura, Dornbirn
Aproaerema anthyllidella	TLMF Lep 10051	LEATA444-13	KM572663	BOLD:AAD2266	Austria	inatura, Dornbirn
Aproaerema anthyllidella	MM09497	LEFIE589-10	HM874312	BOLD:AAD2267	Finland	University of Oulu
Aproaerema anthyllidella	MM13629	LEFIG011-10	HM875691	BOLD:AAD2267	Finland	University of Oulu
Aproaerema anthyllidella	MM13630	LEFIG012-10	HM875692	BOLD:AAD2267	Finland	University of Oulu

Aproaerema anthyllidella	MM12471	LEFIF600-10	HM875284	BOLD:AAD2267	Finland	University of Oulu
Aproaerema anthyllidella	MM17205	LEFIJ580-10	KM572895	BOLD:AAD2267	Finland	University of Oulu
Aproaerema anthyllidella	MM09496	LEFIE588-10	HM874311	BOLD:AAD2267	Finland	University of Oulu
Aproaerema anthyllidella	MM21140	LEFIJ1280-11	KM572221	BOLD:AAD2267	Finland	University of Oulu
Apterogenum ypsillon	TLMF Lep 10022	LEATA415-13	KM572322	BOLD:AAD1478	Austria	inatura, Dornbirn
Apterogenum ypsillon	MM03517	LEFIC166-10	HM872012	BOLD:AAD1478	Finland	University of Oulu
Apterogenum ypsillon	MM12658	LEFIF676-10	HM875360	BOLD:AAD1478	Finland	University of Oulu
Apterogenum ypsillon	MM03656	LEFIC246-10	HM872090	BOLD:AAD1478	Finland	University of Oulu
Araschnia levana	MM06026	LEFID200-10	HM873003	BOLD:AAB1698	Finland	University of Oulu
Araschnia levana	MM06027	LEFID201-10	HM873004	BOLD:AAB1698	Finland	University of Oulu
Araschnia levana	MM14152	LEFIG238-10	HM875917	BOLD:AAB1698	Finland	University of Oulu
Araschnia levana	TLMF Lep 01127	PHLAB327-10	HQ968489	BOLD:AAB1698	Austria	Tiroler Landesmuseum Ferdinandeum
Archinemapogon yildizae	MM09920	LEFIE769-10	HM874488	BOLD:AAF5766	Finland	University of Oulu
Archinemapogon yildizae	MM02301	LEFIB593-10	HM871472	BOLD:AAF5766	Finland	University of Oulu
Archinemapogon yildizae	MM05212	LEFIC899-10	HM872716	BOLD:AAF5766	Finland	University of Oulu
Archinemapogon yildizae	TLMF Lep 09933	PHLAW136-13	KM572403	BOLD:AAF5766	Austria	inatura, Dornbirn
Archips oporana	MM08821	LEFIE366-10	HM874090	BOLD:AAD6710	Finland	University of Oulu
Archips oporana	MM03449	LEFIC115-10	HM871962	BOLD:AAD6710	Finland	University of Oulu
Archips oporana	TLMF Lep 08015	PHLAV196-12	KM572459	BOLD:AAD6710	Austria	inatura, Dornbirn
Archips oporana	MM02019	LEFIB469-10	HM871368	BOLD:AAD6710	Finland	University of Oulu
Archips podana	MM06722	LEFID679-10	HM873440	BOLD:AAB5839	Finland	University of Oulu
Archips podana	MM15190	LEFIJ303-10	JN286435	BOLD:AAB5839	Finland	University of Oulu
Archips podana	TLMF Lep 08569	PHLAH750-12	KM573356	BOLD:AAB5839	Austria	inatura, Dornbirn
Archips podana	MM05228	LEFIC906-10	HM872723	BOLD:AAB5839	Finland	University of Oulu
Archips podana	MM15653	LEFIG789-10	HM876441	BOLD:AAB5839	Finland	University of Oulu
Archips podana	MM15652	LEFIG788-10	HM876440	BOLD:AAB5839	Finland	University of Oulu
Archips rosana	TLMF Lep 08262	PHLAH443-12	KM573536	BOLD:AAB9404	Austria	inatura, Dornbirn
Archips rosana	TLMF Lep 08029	PHLAV210-12	KM572636	BOLD:AAB9404	Austria	inatura, Dornbirn
Archips rosana	MM18256	LEFIK681-10	JF854249	BOLD:ACE6948	Finland	University of Oulu
Archips rosana	MM10167	LEFIE889-10	HM874607	BOLD:ACE6948	Finland	University of Oulu
Archips rosana	MM02016	LEFIB468-10	HM871367	BOLD:ACE6948	Finland	University of Oulu

Archips xylosteana	MM09490	LEFIE585-10	HM874308	BOLD:AAC0366	Finland	University of Oulu
Archips xylosteana	MM09757	LEFIA880-10	HM387018	BOLD:AAC0366	Finland	University of Oulu
Archips xylosteana	MM04987	LEFIC811-10	HM872630	BOLD:AAC0366	Finland	University of Oulu
Archips xylosteana	TLMF Lep 08013	PHLAV194-12	KM572080	BOLD:AAC0366	Austria	inatura, Dornbirn
Arctia caja	TLMF Lep 06116	PHLSA661-11	KM572296	BOLD:AAA8530	Austria	Tiroler Landesmuseum Ferdinandeum
Arctia caja	MM07925	LEFID959-10	HM873709	BOLD:AAA8530	Finland	University of Oulu
Arctia caja	MM04921	LEFIC785-10	HM872604	BOLD:AAA8530	Finland	University of Oulu
Arctia caja	MM09741	LEFIA864-10	HM387002	BOLD:AAA8530	Finland	University of Oulu
Arctia caja	MM20723	LEEUA664-11	KM573193	BOLD:AAA8530	Finland	University of Oulu
Arctornis l-nigrum	MM15923	LEFIJ323-10	KM572905	BOLD:AAD9740	Finland	University of Oulu
Arctornis l-nigrum	MM15922	LEFIJ322-10	KM573670	BOLD:AAD9740	Finland	University of Oulu
Arctornis l-nigrum	TLMF Lep 06115	PHLSA660-11	KM572716	BOLD:AAD9740	Austria	Tiroler Landesmuseum Ferdinandeum
Argolamprotes micella	MM03089	LEFIB939-10	HM871816	BOLD:AAD2506	Finland	University of Oulu
Argolamprotes micella	MM09793	LEFIA915-10	HM387051	BOLD:AAD2506	Finland	University of Oulu
Argolamprotes micella	MM02315	LEFIB601-10	HM871480	BOLD:AAD2506	Finland	University of Oulu
Argolamprotes micella	TLMF Lep 08246	PHLAH427-12	KM573648	BOLD:AAD2506	Austria	inatura, Dornbirn
Argynnis adippe	TLMF Lep 12604	LEATC622-13	KM573714	BOLD:AAB2344	Austria	Tiroler Landesmuseum Ferdinandeum
Argynnis adippe	MM17164	LEFIJ539-10	JF853656	BOLD:AAB2344	Finland	University of Oulu
Argynnis adippe	MM17163	LEFIJ538-10	JF853655	BOLD:AAB2344	Finland	University of Oulu
Argynnis adippe	MM14397	LEFIG384-10	HM876061	BOLD:AAB2344	Finland	University of Oulu
Argynnis aglaja	MM06526	LEFID541-10	HM873306	BOLD:AAB2322	Finland	University of Oulu
Argynnis aglaja	TLMF Lep 08561	PHLAH742-12	KM572903	BOLD:AAB2322	Austria	inatura, Dornbirn
Argynnis aglaja	MM03355	LEFIC070-10	HM871939	BOLD:AAB2322	Finland	University of Oulu
Argynnis aglaja	MM17162	LEFIJ537-10	JF853654	BOLD:AAB2322	Finland	University of Oulu
Argynnis niobe	MM14895	LEFIJ279-10	JF853516	BOLD:ABY9182	Finland	University of Oulu
Argynnis niobe	MM14896	LEFIJ280-10	JF853517	BOLD:ABY9182	Finland	University of Oulu
Argynnis niobe	TLMF Lep 08562	PHLAH743-12	KM572347	BOLD:ABY9182	Austria	inatura, Dornbirn
Argynnis niobe	MM17165	LEFIJ540-10	JF853657	BOLD:ABY9182	Finland	University of Oulu
Argynnis paphia	MM06997	LEFID864-10	HM873621	BOLD:AAA8958	Finland	University of Oulu
Argynnis paphia	TLMF Lep 12602	LEATC620-13	KM573667	BOLD:AAA8958	Austria	Tiroler Landesmuseum Ferdinandeum
Argynnis paphia	MM17158	LEFIJ533-10	JF853650	BOLD:AAA8958	Finland	University of Oulu

Argynnis paphia	MM03384	LEFIC085-10	HM871953	BOLD:AAA8958	Finland	University of Oulu
Argyresthia albistria	MM13589	LEFIF988-10	HM875669	BOLD:AAD9653	Finland	University of Oulu
Argyresthia albistria	MM06773	LEFID719-10	HM873476	BOLD:AAD9653	Finland	University of Oulu
Argyresthia albistria	MM17949	LEFIK374-10	JF854007	BOLD:AAD9653	Finland	University of Oulu
Argyresthia albistria	MM09511	LEFIE599-10	HM874322	BOLD:AAD9653	Finland	University of Oulu
Argyresthia albistria	TLMF Lep 08464	PHLAH645-12	KM573132	BOLD:AAD9653	Austria	inatura, Dornbirn
Argyresthia aurulentella	TLMF Lep 09189	PHLAI627-13	KM573265	BOLD:AAF5138	Austria	Tiroler Landesmuseum Ferdinandeum
Argyresthia aurulentella	MM02627	LEFIB764-10	HM871641	BOLD:AAF5138	Finland	University of Oulu
Argyresthia aurulentella	MM14643	LEFIG525-10	HM876198	BOLD:AAF5138	Finland	University of Oulu
Argyresthia brockeella	MM02309	LEFIB596-10	HM871475	BOLD:AAD2486	Finland	University of Oulu
Argyresthia brockeella	MM03133	LEFIB959-10	HM871835	BOLD:AAD2486	Finland	University of Oulu
Argyresthia brockeella	MM08132	LEFIE043-10	HM873792	BOLD:AAD2486	Finland	University of Oulu
Argyresthia brockeella	TLMF Lep 08752	PHLAH948-12	KM573606	BOLD:AAD2486	Austria	Tiroler Landesmuseum Ferdinandeum
Argyresthia conjugella	MM06838	LEFID772-10	HM873529	BOLD:ACE7677	Finland	University of Oulu
Argyresthia conjugella	MM08380	LEFIE144-10	HM873892	BOLD:ACE7677	Finland	University of Oulu
Argyresthia conjugella	TLMF Lep 08070	PHLAV251-12	KM572913	BOLD:ACE7677	Austria	inatura, Dornbirn
Argyresthia conjugella	TLMF Lep 03626	PHLAD451-11	JN263030	BOLD:ACE7677	Austria	Tiroler Landesmuseum Ferdinandeum
Argyresthia conjugella	MM02292	LEFIB590-10	HM871469	BOLD:ACE7677	Finland	University of Oulu
Argyresthia conjugella	TLMF Lep 08746	PHLAH942-12	KM572334	BOLD:ACE7678	Austria	Tiroler Landesmuseum Ferdinandeum
Argyresthia conjugella	TLMF Lep 08269	PHLAH450-12	KM573343	BOLD:ACE7678	Austria	inatura, Dornbirn
Argyresthia conjugella	MM03137	LEFIB962-10	HM871838	BOLD:ACE7678	Finland	University of Oulu
Argyresthia glabratella	MM18111	LEFIK536-10	JF854133	BOLD:AAD4102	Finland	University of Oulu
Argyresthia glabratella	TLMF Lep 07494	PHLAG815-12	KM572545	BOLD:AAD4102	Austria	inatura, Dornbirn
Argyresthia glabratella	MM18109	LEFIK534-10	JF854131	BOLD:AAD4102	Finland	University of Oulu
Argyresthia glabratella	MM03262	LEFIC035-10	HM871905	BOLD:AAD4102	Finland	University of Oulu
Argyresthia glabratella	TLMF Lep 09212	PHLAI650-13	KM573196	BOLD:AAD4102	Austria	Tiroler Landesmuseum Ferdinandeum
Argyresthia glabratella	MM14184	LEFIG258-10	HM875937	BOLD:AAD4102	Finland	University of Oulu
Argyresthia goedartella	MM02340	LEFIB611-10	HM871490	BOLD:AAA8888	Finland	University of Oulu
Argyresthia goedartella	MM12019	LEFIF474-10	HM875159	BOLD:AAA8888	Finland	University of Oulu
Argyresthia goedartella	TLMF Lep 08224	PHLAH405-12	KM573259	BOLD:AAA8888	Austria	inatura, Dornbirn
Argyresthia goedartella	MM03136	LEFIB961-10	HM871837	BOLD:AAA8888	Finland	University of Oulu

Argyresthia pruniella	MM18105	LEFIK530-10	JF854127	BOLD:AAC2750	Finland	University of Oulu
Argyresthia pruniella	TLMF Lep 08052	PHLAV233-12	KM573647	BOLD:AAC2750	Austria	inatura, Dornbirn
Argyresthia pruniella	MM21022	LEFIJ1162-11	KM572054	BOLD:AAC2750	Finland	University of Oulu
Argyresthia pruniella	TLMF Lep 08259	PHLAH440-12	KM572365	BOLD:AAC2750	Austria	inatura, Dornbirn
Argyresthia pruniella	MM17950	LEFIK375-10	JF854008	BOLD:AAC2750	Finland	University of Oulu
Argyresthia pruniella	MM09531	LEFIE615-10	HM874338	BOLD:AAC2750	Finland	University of Oulu
Argyresthia retinella	MM03138	LEFIB963-10	HQ570278	BOLD:AAF5148	Finland	University of Oulu
Argyresthia retinella	BIOUG04118-A09	GMFIE834-12	KM573295	BOLD:AAF5148	Finland	Biodiversity Institute of Ontario
Argyresthia retinella	BIOUG04118-C12	GMFIO701-13	KM572788	BOLD:AAF5148	Finland	Biodiversity Institute of Ontario
Argyresthia retinella	BIOUG04118-D11	GMFIO712-13	KM572527	BOLD:AAF5148	Finland	Biodiversity Institute of Ontario
Argyresthia retinella	BIOUG04118-D07	GMFIO708-13	KM573479	BOLD:AAF5148	Finland	Biodiversity Institute of Ontario
Argyresthia retinella	BIOUG04118-D06	GMFIO707-13	KM572598	BOLD:AAF5148	Finland	Biodiversity Institute of Ontario
Argyresthia retinella	BIOUG04118-D05	GMFIO706-13	KM573542	BOLD:AAF5148	Finland	Biodiversity Institute of Ontario
Argyresthia retinella	BIOUG04118-D04	GMFIO705-13	KM571994	BOLD:AAF5148	Finland	Biodiversity Institute of Ontario
Argyresthia retinella	TLMF Lep 08073	PHLAV254-12	KM573483	BOLD:AAF5148	Austria	inatura, Dornbirn
Argyresthia retinella	MM02583	LEFIB742-10	HM871620	BOLD:AAF5148	Finland	University of Oulu
Argyresthia retinella	MM13594	LEFIF990-10	HM875671	BOLD:AAF5148	Finland	University of Oulu
Argyresthia retinella	BIOUG04118-A10	GMFIE835-12	KM573298	BOLD:AAF5148	Finland	Biodiversity Institute of Ontario
Argyresthia retinella	BIOUG04118-A08	GMFIE833-12	KM573417	BOLD:AAF5148	Finland	Biodiversity Institute of Ontario
Argyresthia sorbiella	MM06781	LEFID725-10	HM873482	BOLD:AAD9497	Finland	University of Oulu
Argyresthia sorbiella	MM13593	LEFIF989-10	HM875670	BOLD:AAD9497	Finland	University of Oulu
Argyresthia sorbiella	MM06767	LEFID713-10	HM873474	BOLD:AAD9497	Finland	University of Oulu
Argyresthia sorbiella	TLMF Lep 08413	PHLAH594-12	KM572740	BOLD:AAD9497	Austria	inatura, Dornbirn
Argyresthia sorbiella	MM14256	LEFIG298-10	HM875977	BOLD:AAD9497	Finland	University of Oulu
Argyresthia sorbiella	MM02629	LEFIB766-10	HM871643	BOLD:AAD9497	Finland	University of Oulu
Argyresthia svenssoni	MM18108	LEFIK533-10	JF854130	BOLD:AAD4103	Finland	University of Oulu
Argyresthia svenssoni	MM18107	LEFIK532-10	JF854129	BOLD:AAD4103	Finland	University of Oulu
Argyresthia svenssoni	MM06447	LEFIJ092-10	JF853439	BOLD:AAD4103	Finland	University of Oulu
Argyresthia svenssoni	MM06408	LEFID467-10	HM873235	BOLD:AAD4103	Finland	University of Oulu
Argyresthia svenssoni	MM06446	LEFIJ091-10	JF853438	BOLD:AAD4103	Finland	University of Oulu
Argyresthia svenssoni	MM18115	LEFIK540-10	KM572856	BOLD:AAD4103	Finland	University of Oulu

Argyresthia svenssoni	MM06445	LEFIJ090-10	JF853437	BOLD:AAD4103	Finland	University of Oulu
Argyresthia svenssoni	MM18110	LEFIK535-10	JF854132	BOLD:AAD4103	Finland	University of Oulu
Argyresthia svenssoni	TLMF Lep 09985	PHLAW188-13	KM573306	BOLD:ACI7598	Austria	inatura, Dornbirn
Argyroploce noricana	TLMF Lep 00756	PHLAA716-09	HM426110	BOLD:AAD8744	Austria	Tiroler Landesmuseum Ferdinandeum
Argyroploce noricana	MM18285	LEFIK710-10	KM573474	BOLD:AAD8744	Finland	University of Oulu
Argyroploce noricana	MM07040	LEFID885-10	JX034609	BOLD:AAD8744	Finland	University of Oulu
Argyroploce noricana	MM00083	LEFIA012-10	HM396362	BOLD:AAD8744	Finland	University of Oulu
Argyrotaenia ljungiana	TLMF Lep 04304	PHLAE084-11	JN286471	BOLD:AAA2955	Austria	Tiroler Landesmuseum Ferdinandeum
Argyrotaenia ljungiana	MM05462	LEFID027-10	HM872841	BOLD:AAA2955	Finland	University of Oulu
Argyrotaenia ljungiana	MM06287	LEFID376-10	HM873173	BOLD:AAA2955	Finland	University of Oulu
Argyrotaenia ljungiana	MM06423	LEFID481-10	HM873247	BOLD:AAA2955	Finland	University of Oulu
Argyrotaenia ljungiana	MM21107	LEFIJ1247-11	KM573457	BOLD:AAA2955	Finland	University of Oulu
Arichanna melanaria	MM01351	LEFIA305-10	HM386648	BOLD:AAC8717	Finland	University of Oulu
Arichanna melanaria	MM08115	LEFIE035-10	HM873784	BOLD:AAC8717	Finland	University of Oulu
Arichanna melanaria	TLMF Lep 02877	PHLAC842-10	JF860339	BOLD:AAC8717	Austria	Tiroler Landesmuseum Ferdinandeum
Arichanna melanaria	MM01352	LEFIA306-10	HM386649	BOLD:AAC8717	Finland	University of Oulu
Aricia artaxerxes	MM09926	LEFIE772-10	HM874491	BOLD:ACE7893	Finland	University of Oulu
Aricia artaxerxes	TLMF Lep 08522	PHLAH703-12	KM573033	BOLD:ACE7893	Austria	inatura, Dornbirn
Aricia artaxerxes	BC ZSM Lep 50441	FBLMX230-11	KM573513	BOLD:ACE7893	Austria	Research Collection of Ralf Bolz
Aricia artaxerxes	MM17139	LEFIJ514-10	JF853636	BOLD:ACE7893	Finland	University of Oulu
Aricia artaxerxes	BC ZSM Lep 50439	FBLMX228-11	KM573699	BOLD:ACE7893	Austria	Research Collection of Ralf Bolz
Aricia artaxerxes	MM03347	LEFIC063-10	HM871932	BOLD:ACE7893	Finland	University of Oulu
Aricia artaxerxes	BC ZSM Lep 50436	FBLMX225-11	KM572513	BOLD:ACE7893	Austria	Research Collection of Ralf Bolz
Aricia artaxerxes	TLMF Lep 09841	PHLAW044-13	KM572272	BOLD:ACE7893	Austria	Tiroler Landesmuseum Ferdinandeum
Aricia artaxerxes	BC ZSM Lep 50432	FBLMX221-11	KM572320	BOLD:ACE7893	Austria	Research Collection of Ralf Bolz
Aricia artaxerxes	BC ZSM Lep 50443	FBLMX232-11	KM571976	BOLD:ACE7893	Austria	Research Collection of Ralf Bolz
Aricia artaxerxes	BC ZSM Lep 50434	FBLMX223-11	KM573324	BOLD:ACE7893	Austria	Research Collection of Ralf Bolz
Aspilapteryx tringipennella	MM13728	LEFIG078-10	HM875757	BOLD:AAD1923	Finland	University of Oulu
Aspilapteryx tringipennella	MM09218	LEFIE454-10	HM874178	BOLD:AAD1923	Finland	University of Oulu

Aspilapteryx tringipennella	MM14366	LEFIG365-10	HM876042	BOLD:AAD1923	Finland	University of Oulu
Aspilapteryx tringipennella	TLMF Lep 07467	PHLAG788-12	KM572416	BOLD:AAD1923	Austria	inatura, Dornbirn
Aspilapteryx tringipennella	TLMF Lep 04421	PHLAE201-11	JN272062	BOLD:AAD1923	Austria	Tiroler Landesmuseum Ferdinandeum
Assara terebrella	CNCLEP00020420	LNEL224-06	KM572671	BOLD:AAC4140	Finland	Canadian National Collection of Insects, Arachnids and Nematodes
Assara terebrella	MM02432	LEFIB659-10	HM871537	BOLD:AAC4140	Finland	University of Oulu
Assara terebrella	MM03553	LEFIC192-10	HM872036	BOLD:AAC4140	Finland	University of Oulu
Assara terebrella	MM09811	LEFIE720-10	HM874440	BOLD:AAC4140	Finland	University of Oulu
Assara terebrella	TLMF Lep 08025	PHLAV206-12	KM573666	BOLD:ACA2238	Austria	inatura, Dornbirn
Asthena albulata	TLMF Lep 08534	PHLAH715-12	KM572582	BOLD:AAC7503	Austria	inatura, Dornbirn
Asthena albulata	MM06107	LEFID243-10	HM873042	BOLD:AAC7503	Finland	University of Oulu
Asthena albulata	MM11625	LEFIF379-10	HM875064	BOLD:AAC7503	Finland	University of Oulu
Asthena albulata	MM11624	LEFIF378-10	HM875063	BOLD:AAC7503	Finland	University of Oulu
Athetis pallustris	MM01521	LEFIA456-10	HM386796	BOLD:AAF5119	Finland	University of Oulu
Athetis pallustris	TLMF Lep 04676	PHLAE361-11	JN262025	BOLD:AAF5119	Austria	Tiroler Landesmuseum Ferdinandeum
Athetis pallustris	MM01522	LEFIA457-10	HM386797	BOLD:AAF5119	Finland	University of Oulu
Athetis pallustris	MM02778	LEFIB829-10	HM871706	BOLD:AAF5119	Finland	University of Oulu
Athrips mouffetella	MM09836	LEFIE736-10	HM874456	BOLD:AAC9993	Finland	University of Oulu
Athrips mouffetella	TLMF Lep 10045	LEATA438-13	KM573443	BOLD:AAC9993	Austria	inatura, Dornbirn
Athrips mouffetella	MM05061	LEFIC849-10	HM872667	BOLD:AAC9993	Finland	University of Oulu
Athrips mouffetella	MM09777	LEFIA899-10	HM387035	BOLD:AAC9993	Finland	University of Oulu
Atolmis rubricollis	TLMF Lep 07851	PHLAV032-12	KM572174	BOLD:AAD2377	Austria	inatura, Dornbirn
Atolmis rubricollis	MM01018	LEFIA096-10	HM396443	BOLD:AAD2377	Finland	University of Oulu
Atolmis rubricollis	MM01017	LEFIA095-10	HM396442	BOLD:AAD2377	Finland	University of Oulu
Atolmis rubricollis	MM04554	LEFIC620-10	HM872441	BOLD:AAD2377	Finland	University of Oulu
Autographa bractea	MM01233	LEFIA210-10	HM396554	BOLD:AAD2827	Finland	University of Oulu
Autographa bractea	MM01234	LEFIA211-10	HM396555	BOLD:AAD2827	Finland	University of Oulu
Autographa bractea	TLMF Lep 06206	PHLSA751-11	KM572204	BOLD:AAD2827	Austria	Tiroler Landesmuseum Ferdinandeum

Autographa bractea	MM09746	LEFIA869-10	HM387007	BOLD:AAD2827	Finland	University of Oulu
Autographa bractea	MM01232	LEFIA209-10	HM396553	BOLD:AAD2827	Finland	University of Oulu
Autographa gamma	TLMF Lep 08167	PHLAV348-12	KM573418	BOLD:AAB4345	Austria	inatura, Dornbirn
Autographa gamma	MM00328	LEFIA1013-10	GU828636	BOLD:AAB4345	Finland	University of Oulu
Autographa gamma	MM04361	LEFIC580-10	HM872401	BOLD:AAB4345	Finland	University of Oulu
Autographa gamma	MM10744	LEFIF177-10	HM874871	BOLD:AAB4345	Finland	University of Oulu
Autographa gamma	MM04637	LEFIC671-10	HM872492	BOLD:AAB4345	Finland	University of Oulu
Autographa gamma	MM12593	LEFIF656-10	HM875340	BOLD:AAB4345	Finland	University of Oulu
Autographa jota	MM09788	LEFIA910-10	HM387046	BOLD:AAA3991	Finland	University of Oulu
Autographa jota	TLMF Lep 07565	PHLAG886-12	KM573333	BOLD:AAA3991	Austria	inatura, Dornbirn
Autographa jota	MM12511	LEFIF622-10	HM875306	BOLD:AAA3991	Finland	University of Oulu
Autographa jota	MM09789	LEFIA911-10	HM387047	BOLD:AAA3991	Finland	University of Oulu
Autographa jota	MM04527	LEFIC607-10	HM872428	BOLD:AAA3991	Finland	University of Oulu
Autographa jota	MM04528	LEFIC608-10	HM872429	BOLD:AAA3991	Finland	University of Oulu
Autographa jota	MM10734	LEFIF176-10	HM874870	BOLD:AAA3991	Finland	University of Oulu
Autographa jota	MM04529	LEFIC609-10	HM872430	BOLD:AAA3991	Finland	University of Oulu
Autographa jota	MM04526	LEFIC606-10	HM872427	BOLD:AAA3991	Finland	University of Oulu
Autographa jota	MM01183	LEFIA190-10	HM396535	BOLD:AAA3991	Finland	University of Oulu
Autographa pulchrina	MM04516	LEFIC596-10	HM872417	BOLD:AAA3990	Finland	University of Oulu
Autographa pulchrina	MM02750	LEFIB814-10	HM871691	BOLD:AAA3990	Finland	University of Oulu
Autographa pulchrina	MM14481	LEFIG443-10	HM876119	BOLD:AAA3990	Finland	University of Oulu
Autographa pulchrina	MM14492	LEFIG453-10	HM876129	BOLD:AAA3990	Finland	University of Oulu
Autographa pulchrina	MM08326	LEFIE121-10	HM873869	BOLD:AAA3990	Finland	University of Oulu
Autographa pulchrina	MM08447	LEFIE174-10	HM873920	BOLD:AAA3990	Finland	University of Oulu
Autographa pulchrina	MM08448	LEFIE175-10	HM873921	BOLD:AAA3990	Finland	University of Oulu
Autographa pulchrina	MM10127	LEFIE868-10	HM874586	BOLD:AAA3990	Finland	University of Oulu
Autographa pulchrina	MM10133	LEFIE869-10	HM874587	BOLD:AAA3990	Finland	University of Oulu
Autographa pulchrina	MM01195	LEFIB391-10	HM871290	BOLD:AAA3990	Finland	University of Oulu
Autographa pulchrina	MM01196	LEFIB392-10	HM871291	BOLD:AAA3990	Finland	University of Oulu
Autographa pulchrina	MM01186	LEFIA193-10	HM396538	BOLD:AAA3990	Finland	University of Oulu
Autographa pulchrina	MM04522	LEFIC602-10	HM872423	BOLD:AAA3990	Finland	University of Oulu

Autographa pulchrina	MM01197	LEFIB393-10	HM871292	BOLD:AAA3990	Finland	University of Oulu
Autographa pulchrina	MM01201	LEFIB397-10	HM871296	BOLD:AAA3990	Finland	University of Oulu
Autographa pulchrina	MM04523	LEFIC603-10	HM872424	BOLD:AAA3990	Finland	University of Oulu
Autographa pulchrina	MM04524	LEFIC604-10	HM872425	BOLD:AAA3990	Finland	University of Oulu
Autographa pulchrina	MM01188	LEFIA195-10	HM396540	BOLD:AAA3990	Finland	University of Oulu
Autographa pulchrina	MM04525	LEFIC605-10	HM872426	BOLD:AAA3990	Finland	University of Oulu
Autographa pulchrina	MM01202	LEFIB398-10	HM871297	BOLD:AAA3990	Finland	University of Oulu
Autographa pulchrina	MM01189	LEFIA196-10	HM396541	BOLD:AAA3990	Finland	University of Oulu
Autographa pulchrina	MM14084	LEFIG201-10	HM875881	BOLD:AAA3990	Finland	University of Oulu
Autographa pulchrina	MM05503	LEFID041-10	HM872855	BOLD:AAA3990	Finland	University of Oulu
Autographa pulchrina	MM01191	LEFIA198-10	HM396543	BOLD:AAA3990	Finland	University of Oulu
Autographa pulchrina	MM05524	LEFID050-10	HM872864	BOLD:AAA3990	Finland	University of Oulu
Autographa pulchrina	MM09738	LEFIA861-10	HM386999	BOLD:AAA3990	Finland	University of Oulu
Autographa pulchrina	MM09739	LEFIA862-10	HM387000	BOLD:AAA3990	Finland	University of Oulu
Autographa pulchrina	MM07381	LEFID919-10	HM873669	BOLD:AAA3990	Finland	University of Oulu
Autographa pulchrina	MM01199	LEFIB395-10	HM871294	BOLD:AAA3990	Finland	University of Oulu
Autographa pulchrina	MM10720	LEFIF171-10	HM874865	BOLD:AAA3990	Finland	University of Oulu
Autographa pulchrina	MM01200	LEFIB396-10	HM871295	BOLD:AAA3990	Finland	University of Oulu
Autographa pulchrina	MM04519	LEFIC599-10	HM872420	BOLD:AAA3990	Finland	University of Oulu
Autographa pulchrina	MM06684	LEFID657-10	HM873419	BOLD:AAA3990	Finland	University of Oulu
Autographa pulchrina	MM08325	LEFIE120-10	HM873868	BOLD:AAA3990	Finland	University of Oulu
Autographa pulchrina	MM10724	LEFIF175-10	HM874869	BOLD:AAA3990	Finland	University of Oulu
Autographa pulchrina	MM10717	LEFIF168-10	HM874862	BOLD:AAA3990	Finland	University of Oulu
Autographa pulchrina	MM09786	LEFIA908-10	HM387044	BOLD:AAA3990	Finland	University of Oulu
Autographa pulchrina	MM09787	LEFIA909-10	HM387045	BOLD:AAA3990	Finland	University of Oulu
Autographa pulchrina	MM07759	LEFID946-10	HM873696	BOLD:AAA3990	Finland	University of Oulu
Autographa pulchrina	MM01198	LEFIB394-10	HM871293	BOLD:AAA3990	Finland	University of Oulu
Autographa pulchrina	MM06978	LEFID852-10	HM873609	BOLD:AAA3990	Finland	University of Oulu
Autographa pulchrina	MM01194	LEFIB390-10	HM871289	BOLD:AAA3990	Finland	University of Oulu
Autographa pulchrina	MM01193	LEFIB389-10	HM871288	BOLD:AAA3990	Finland	University of Oulu
Autographa pulchrina	MM04521	LEFIC601-10	HM872422	BOLD:AAA3990	Finland	University of Oulu

Autographa pulchrina	MM07382	LEFID920-10	HM873670	BOLD:AAA3990	Finland	University of Oulu
Autographa pulchrina	MM01192	LEFIA199-10	HM396544	BOLD:AAA3990	Finland	University of Oulu
Autographa pulchrina	MM07947	LEFID962-10	HM873712	BOLD:AAA3990	Finland	University of Oulu
Autographa pulchrina	MM08303	LEFIE114-10	HM873862	BOLD:AAA3990	Finland	University of Oulu
Autographa pulchrina	MM13862	LEFIA934-10	HM387068	BOLD:AAA3990	Finland	University of Oulu
Autographa pulchrina	MM10719	LEFIF170-10	HM874864	BOLD:AAA3990	Finland	University of Oulu
Autographa pulchrina	MM02751	LEFIB815-10	HM871692	BOLD:AAA3990	Finland	University of Oulu
Autographa pulchrina	MM04518	LEFIC598-10	HM872419	BOLD:AAA3990	Finland	University of Oulu
Autographa pulchrina	MM13876	LEFIA948-10	HM387080	BOLD:AAA3990	Finland	University of Oulu
Autographa pulchrina	MM13877	LEFIA949-10	HM387081	BOLD:AAA3990	Finland	University of Oulu
Autographa pulchrina	MM10721	LEFIF172-10	HM874866	BOLD:AAA3990	Finland	University of Oulu
Autographa pulchrina	TLMF Lep 04433	PHLAE213-11	JN276673	BOLD:AAA3990	Austria	Tiroler Landesmuseum Ferdinandeum
Autographa pulchrina	MM12614	LEFIF664-10	HM875348	BOLD:AAA3990	Finland	University of Oulu
Autographa pulchrina	MM04561	LEFIC625-10	HM872446	BOLD:AAA3990	Finland	University of Oulu
Autographa pulchrina	MM12595	LEFIF658-10	HM875342	BOLD:AAA3990	Finland	University of Oulu
Autographa pulchrina	MM12594	LEFIF657-10	HM875341	BOLD:AAA3990	Finland	University of Oulu
Autographa pulchrina	MM04517	LEFIC597-10	HM872418	BOLD:AAA3990	Finland	University of Oulu
Autographa pulchrina	MM04515	LEFIC595-10	HM872416	BOLD:AAA3990	Finland	University of Oulu
Autographa pulchrina	TLMF Lep 00295	PHLAA255-09	HM425792	BOLD:AAA3990	Austria	Tiroler Landesmuseum Ferdinandeum
Autographa pulchrina	MM06685	LEFID658-10	HM873420	BOLD:AAA3990	Finland	University of Oulu
Autographa pulchrina	MM09480	LEFIE581-10	HM874304	BOLD:AAA3990	Finland	University of Oulu
Autographa pulchrina	TLMF Lep 00744	PHLAA704-09	HM426101	BOLD:AAA3990	Austria	Tiroler Landesmuseum Ferdinandeum
Axylia putris	MM05142	LEFIC869-10	HM872687	BOLD:AAB6626	Finland	University of Oulu
Axylia putris	TLMF Lep 07882	PHLAV063-12	KM572713	BOLD:AAB6626	Austria	inatura, Dornbirn
Axylia putris	MM01629	LEFIA542-10	KM572031	BOLD:AAB6626	Finland	University of Oulu
Axylia putris	MM01628	LEFIA541-10	KM572025	BOLD:AAB6626	Finland	University of Oulu
Bactra lacteana	MM14258	LEFIG300-10	HM875979	BOLD:AAD3405	Finland	University of Oulu
Bactra lacteana	MM13762	LEFIG092-10	HM875771	BOLD:AAD3405	Finland	University of Oulu
Bactra lacteana	MM11013	LEFIF233-10	HM874926	BOLD:AAD3405	Finland	University of Oulu
Bactra lacteana	MM06836	LEFID770-10	HM873527	BOLD:AAD3405	Finland	University of Oulu
Bactra lacteana	TLMF Lep 09958	PHLAW161-13	KM572569	BOLD:AAD3405	Austria	inatura, Dornbirn

Bactra lacteana	MM06835	LEFID769-10	HM873526	BOLD:AAD3405	Finland	University of Oulu
Bactra lancealana	MM21123	LEFIJ1263-11	KM573258	BOLD:AAB8686	Finland	University of Oulu
Bactra lancealana	MM12364	LEFIF576-10	HM875260	BOLD:AAB8686	Finland	University of Oulu
Bactra lancealana	MM12363	LEFIF575-10	HM875259	BOLD:AAB8686	Finland	University of Oulu
Bactra lancealana	MM03907	LEFIC377-10	HM872220	BOLD:AAB8686	Finland	University of Oulu
Bactra lancealana	MM17778	LEFIK203-10	KM573430	BOLD:AAB8686	Finland	University of Oulu
Bactra lancealana	MM12145	LEFIF517-10	HM875202	BOLD:AAB8686	Finland	University of Oulu
Bactra lancealana	MM13763	LEFIG093-10	HM875772	BOLD:AAB8686	Finland	University of Oulu
Bactra lancealana	MM06531	LEFIA1018-10	GU828745	BOLD:AAB8686	Finland	University of Oulu
Bactra lancealana	MM06532	LEFID545-10	HM873310	BOLD:AAB8686	Finland	University of Oulu
Bactra lancealana	MM14172	LEFIG249-10	HM875928	BOLD:AAB8686	Finland	University of Oulu
Bactra lancealana	MM02453	LEFIB670-10	HM871548	BOLD:AAB8686	Finland	University of Oulu
Bactra lancealana	MM06530	LEFID544-10	HM873309	BOLD:AAB8686	Finland	University of Oulu
Bactra lancealana	MM02454	LEFIB671-10	HM871549	BOLD:AAB8686	Finland	University of Oulu
Bactra lancealana	MM03087	LEFIB937-10	HM871814	BOLD:AAB8686	Finland	University of Oulu
Bactra lancealana	MM03043	LEFIB917-10	HM871794	BOLD:AAB8686	Finland	University of Oulu
Bactra lancealana	TLMF Lep 08031	PHLAV212-12	KM573049	BOLD:AAB8686	Austria	inatura, Dornbirn
Bactra lancealana	MM09817	LEFIE724-10	HM874444	BOLD:AAB8686	Finland	University of Oulu
Baptria tibiale	MM17437	LEFIJ812-10	JF853837	BOLD:AAI7598	Finland	University of Oulu
Baptria tibiale	TLMF Lep 09417	PHLAI855-13	KM572243	BOLD:AAI7598	Austria	Tiroler Landesmuseum Ferdinandeum
Baptria tibiale	MM17438	LEFIJ813-10	JF853838	BOLD:AAI7598	Finland	University of Oulu
Baptria tibiale	MM17436	LEFIJ811-10	JF853836	BOLD:AAI7598	Finland	University of Oulu
Baptria tibiale	MM10471	LEFIF031-10	HM874742	BOLD:AAI7598	Finland	University of Oulu
Batrachedra pinicolella	MM22067	LEFIJ1396-12	KM573494	BOLD:AAF0077	Finland	University of Oulu
Batrachedra pinicolella	MM09830	LEFIE731-10	HM874451	BOLD:AAF0077	Finland	University of Oulu
Batrachedra pinicolella	MM16054	LEFIJ1029-11	KM573704	BOLD:AAF0077	Finland	University of Oulu
Batrachedra pinicolella	MM16053	LEFIJ1028-11	KM572193	BOLD:AAF0077	Finland	University of Oulu
Batrachedra pinicolella	MM21053	LEFIJ1193-11	KM572028	BOLD:AAF0077	Finland	University of Oulu
Batrachedra pinicolella	MM16052	LEFIJ1027-11	KM572251	BOLD:AAF0077	Finland	University of Oulu
Batrachedra pinicolella	MM22068	LEFIJ1397-12	KM573329	BOLD:AAF0077	Finland	University of Oulu
Batrachedra pinicolella	MM21051	LEFIJ1191-11	KM572185	BOLD:AAF0077	Finland	University of Oulu

Batrachedra pinicolella	MM22065	LEFIJ1394-12	KM572463	BOLD:AAF0077	Finland	University of Oulu
Batrachedra pinicolella	MM06674	LEFID647-10	HM873409	BOLD:AAF0077	Finland	University of Oulu
Batrachedra pinicolella	MM17247	LEFIJ622-10	JF853721	BOLD:AAF0077	Finland	University of Oulu
Batrachedra pinicolella	TLMF Lep 08072	PHLAV253-12	KM572754	BOLD:AAF0077	Austria	inatura, Dornbirn
Batrachedra pinicolella	MM22066	LEFIJ1395-12	KM572762	BOLD:AAF0077	Finland	University of Oulu
Batrachedra pinicolella	MM21056	LEFIJ1196-11	KM572122	BOLD:AAF0077	Finland	University of Oulu
Batrachedra pinicolella	MM21055	LEFIJ1195-11	KM572001	BOLD:AAF0077	Finland	University of Oulu
Batrachedra pinicolella	MM21054	LEFIJ1194-11	KM572297	BOLD:AAF0078	Finland	University of Oulu
Batrachedra pinicolella	MM21052	LEFIJ1192-11	KM573630	BOLD:AAF0078	Finland	University of Oulu
Batrachedra pinicolella	MM06705	LEFID667-10	HM873429	BOLD:AAF0078	Finland	University of Oulu
Batrachedra praeangusta	MM10275	LEFIE911-10	HM874628	BOLD:ABZ6897	Finland	University of Oulu
Batrachedra praeangusta	MM02328	LEFIA1020-10	GU828680	BOLD:ABZ6897	Finland	University of Oulu
Batrachedra praeangusta	TLMF Lep 12479	LEATC497-13	KM573241	BOLD:ABZ6897	Austria	Tiroler Landesmuseum Ferdinandeum
Batrachedra praeangusta	MM05053	LEFIC842-10	HM872660	BOLD:ABZ6897	Finland	University of Oulu
Bembecia ichneumoniformis	TLMF Lep 09865	PHLAW068-13	KM573015	BOLD:AAB7131	Austria	Tiroler Landesmuseum Ferdinandeum
Bembecia ichneumoniformis	MM21191	LEFIJ951-11	KM573639	BOLD:AAB7131	Finland	University of Oulu
Bembecia ichneumoniformis	MM21231	LEFIJ991-11	KM572422	BOLD:AAB7131	Finland	University of Oulu
Bembecia ichneumoniformis	MM21192	LEFIJ952-11	KM572756	BOLD:AAB7131	Finland	University of Oulu
Bena bicolorana	MM00728	LEFIB256-10	HM871159	BOLD:AAB8808	Finland	University of Oulu
Bena bicolorana	MM18519	LEFIK944-10	KM572169	BOLD:AAB8808	Finland	University of Oulu
Bena bicolorana	TLMF Lep 08105	PHLAV286-12	KM573368	BOLD:AAB8808	Austria	inatura, Dornbirn
Bena bicolorana	MM09767	LEFIA890-10	HM387026	BOLD:AAB8808	Finland	University of Oulu
Bisigna procerella	MM09254	LEFIE479-10	HM874203	BOLD:AAF0146	Finland	University of Oulu
Bisigna procerella	MM05280	LEFIA1023-10	JF818725	BOLD:AAF0146	Finland	University of Oulu
Bisigna procerella	TLMF Lep 08261	PHLAH442-12	KM572435	BOLD:AAF0146	Austria	inatura, Dornbirn
Bisigna procerella	MM11982	LEFIF464-10	HM875149	BOLD:AAF0146	Finland	University of Oulu
Bisigna procerella	MM13578	LEFIF984-10	HM875665	BOLD:AAF0146	Finland	University of Oulu

Biston betularia	MM01227	LEFIA204-10	HM396548	BOLD:ABY9367	Finland	University of Oulu
Biston betularia	MM02799	LEFIB836-10	HM871713	BOLD:ABY9367	Finland	University of Oulu
Biston betularia	MM01127	LEFIA157-10	HM396503	BOLD:ABY9367	Finland	University of Oulu
Biston betularia	MM01128	LEFIA158-10	HM396504	BOLD:ABY9367	Finland	University of Oulu
Biston betularia	TLMF Lep 07841	PHLAV022-12	KM572423	BOLD:ABY9367	Austria	inatura, Dornbirn
Biston betularia	MM05118	LEFIA1024-10	GU828713	BOLD:ABY9367	Finland	University of Oulu
Biston strataria	TLMF Lep 08814	PHLAI319-13	KM572954	BOLD:AAB4693	Austria	Tiroler Landesmuseum Ferdinandeum
Biston strataria	MM02367	LEFIB622-10	HM871501	BOLD:AAB4693	Finland	University of Oulu
Biston strataria	MM04608	LEFIC655-10	HM872476	BOLD:AAB4693	Finland	University of Oulu
Biston strataria	MM12173	LEFIF529-10	HM875214	BOLD:AAB4693	Finland	University of Oulu
Biston strataria	MM04607	LEFIC654-10	HM872475	BOLD:AAB4693	Finland	University of Oulu
Boloria aquilonaris	MM14566	LEFIG493-10	HQ570403	BOLD:AAA9406	Finland	University of Oulu
Boloria aquilonaris	TLMF Lep 00746	PHLAA706-09	HM426103	BOLD:AAA9406	Austria	Tiroler Landesmuseum Ferdinandeum
Boloria aquilonaris	MM03352	LEFIC067-10	HM871936	BOLD:AAA9406	Finland	University of Oulu
Boloria aquilonaris	MM03351	LEFIC066-10	HM871935	BOLD:AAA9406	Finland	University of Oulu
Boloria eunomia	MM17169	LEFIJ544-10	JF853660	BOLD:AAA3397	Finland	University of Oulu
Boloria eunomia	TLMF Lep 09801	PHLAW004-13	KM572223	BOLD:AAA3397	Austria	Tiroler Landesmuseum Ferdinandeum
Boloria eunomia	MM13983	LEFIG144-10	HM875824	BOLD:AAA3397	Finland	University of Oulu
Boloria eunomia	MM13982	LEFIG143-10	HM875823	BOLD:AAA3397	Finland	University of Oulu
Boloria euphrosyne	MM06452	LEFID495-10	HM873260	BOLD:AAB1747	Finland	University of Oulu
Boloria euphrosyne	TLMF Lep 08565	PHLAH746-12	KM572441	BOLD:AAB1747	Austria	inatura, Dornbirn
Boloria euphrosyne	MM17173	LEFIJ548-10	JF853663	BOLD:ACF1010	Finland	University of Oulu
Boloria euphrosyne	MM18688	LEFIL378-10	JN272539	BOLD:ACF1010	Finland	University of Oulu
Boloria napaea	MM03407	LEFIC094-10	HQ570285	BOLD:AAA9406	Finland	University of Oulu
Boloria napaea	TLMF Lep 12552	LEATC570-13	KM572854	BOLD:AAA9406	Austria	Tiroler Landesmuseum Ferdinandeum
Boloria napaea	MM04100	LEFIA746-10	HM386890	BOLD:AAA9406	Finland	University of Oulu
Boloria napaea	MM17168	LEFIJ543-10	JN272537	BOLD:AAA9406	Finland	University of Oulu
Boloria selene	TLMF Lep 06155	PHLSA700-11	KM572813	BOLD:AAA5115	Austria	Tiroler Landesmuseum Ferdinandeum
Boloria selene	MM17170	LEFIJ545-10	JF853661	BOLD:AAA5115	Finland	University of Oulu
Boloria selene	MM03274	LEFIC043-10	HM871913	BOLD:AAA5115	Finland	University of Oulu
Boloria selene	MM03195	LEFIB997-10	HM871868	BOLD:AAA5115	Finland	University of Oulu

Boloria selene	MM17172	LEFIJ547-10	KM573567	BOLD:AAA5115	Finland	University of Oulu
Boloria thore	MM04093	LEFIA739-10	HM386883	BOLD:ABZ5868	Finland	University of Oulu
Boloria thore	MM04094	LEFIA740-10	HM386884	BOLD:ABZ5868	Finland	University of Oulu
Boloria thore	MM03408	LEFIC095-10	HQ570286	BOLD:ABZ5868	Finland	University of Oulu
Boloria thore	TLMF Lep 06154	PHLSA699-11	KM572048	BOLD:ABZ5868	Austria	Tiroler Landesmuseum Ferdinandeum
Borkhausenia fuscescens	MM10400	LEFIE974-10	HM874691	BOLD:AAD4407	Finland	University of Oulu
Borkhausenia fuscescens	TLMF Lep 08299	PHLAH480-12	KM572627	BOLD:AAD4407	Austria	inatura, Dornbirn
Borkhausenia fuscescens	TLMF Lep 09893	PHLAW096-13	KM573154	BOLD:AAD4407	Austria	Tiroler Landesmuseum Ferdinandeum
Borkhausenia fuscescens	MM00132	LEFIA1028-10	JF818728	BOLD:AAD4407	Finland	University of Oulu
Borkhausenia fuscescens	MM22786	LEFIJ1518-12	KM572092	BOLD:AAD4407	Finland	University of Oulu
Borkhausenia fuscescens	MM02424	LEFIB655-10	HM871534	BOLD:AAD4407	Finland	University of Oulu
Borkhausenia fuscescens	MM09329	LEFIE507-10	HM874231	BOLD:AAD4407	Finland	University of Oulu
Brachionycha nubeculosa	MM01543	LEFIA471-10	HM386811	BOLD:AAE0860	Finland	University of Oulu
Brachionycha nubeculosa	MM01614	LEFIA530-10	KM572649	BOLD:AAE0860	Finland	University of Oulu
Brachionycha nubeculosa	MM02767	LEFIB825-10	HM871702	BOLD:AAE0860	Finland	University of Oulu
Brachionycha nubeculosa	MM01542	LEFIA1029-10	GU828667	BOLD:AAE0860	Finland	University of Oulu
Brachionycha nubeculosa	TLMF Lep 09769	LEATA352-13	KM572532	BOLD:AAE0860	Austria	Tiroler Landesmuseum Ferdinandeum
Brachmia blandella	TLMF Lep 08466	PHLAH647-12	KM573274	BOLD:AAD2457	Austria	inatura, Dornbirn
Brachmia blandella	MM18253	LEFIK678-10	JF854246	BOLD:AAD2457	Finland	University of Oulu
Brachmia blandella	MM17582	LEFIK007-10	KM573094	BOLD:AAD2457	Finland	University of Oulu
Brachmia blandella	MM08656	LEFIE302-10	HM874028	BOLD:AAD2457	Finland	University of Oulu
Brachmia blandella	TLMF Lep 08203	PHLAH384-12	KM572536	BOLD:AAD2457	Austria	inatura, Dornbirn
Brachmia inornatella	MM17279	LEFIJ654-10	JF853749	BOLD:AAI8301	Finland	University of Oulu
Brachmia inornatella	TLMF Lep 12493	LEATC511-13	KM573175	BOLD:AAI8301	Austria	Tiroler Landesmuseum Ferdinandeum
Brachmia inornatella	MM15649	LEFIG785-10	HM876437	BOLD:AAI8301	Finland	University of Oulu
Brachmia inornatella	TLMF Lep 12492	LEATC510-13	KM572615	BOLD:AAI8301	Austria	Tiroler Landesmuseum Ferdinandeum
Brachmia inornatella	MM17581	LEFIK006-10	KM573173	BOLD:AAI8301	Finland	University of Oulu
Brachmia inornatella	MM17580	LEFIK005-10	JF853913	BOLD:AAI8301	Finland	University of Oulu
Brachylomia viminalis	MM00835	LEFIB311-10	HM871211	BOLD:AAC7236	Finland	University of Oulu
Brachylomia viminalis	MM01742	LEFIA627-10	HM870876	BOLD:AAC7236	Finland	University of Oulu
Brachylomia viminalis	TLMF Lep 06124	PHLSA669-11	KM573228	BOLD:AAC7236	Austria	Tiroler Landesmuseum Ferdinandeum

Brachylomia viminalis	MM04876	LEFIC758-10	HM872577	BOLD:AAC7236	Finland	University of Oulu
Brenthis ino	MM03353	LEFIC068-10	HM871937	BOLD:AAA9312	Finland	University of Oulu
Brenthis ino	MM03354	LEFIC069-10	HM871938	BOLD:AAA9312	Finland	University of Oulu
Brenthis ino	MM17167	LEFIJ542-10	JF853659	BOLD:AAA9312	Finland	University of Oulu
Brenthis ino	TLMF Lep 09804	PHLAW007-13	KM572384	BOLD:AAA9312	Austria	Tiroler Landesmuseum Ferdinandeum
Bryotropha senectella	MM06672	LEFID645-10	HM873407	BOLD:AAD3661	Finland	University of Oulu
Bryotropha senectella	MM10345	LEFIE948-10	HM874665	BOLD:AAD3661	Finland	University of Oulu
Bryotropha senectella	TLMF Lep 10040	LEATA433-13	KM572566	BOLD:AAD3661	Austria	inatura, Dornbirn
Bryotropha senectella	MM12069	LEFIF500-10	HM875185	BOLD:AAD3661	Finland	University of Oulu
Bryotropha senectella	MM09691	LEFIE704-10	HM874425	BOLD:AAD3661	Finland	University of Oulu
Bryotropha senectella	MM09528	LEFIE613-10	HM874336	BOLD:AAD3661	Finland	University of Oulu
Bryotropha senectella	MM12070	LEFIF501-10	HM875186	BOLD:AAD3661	Finland	University of Oulu
Bryotropha senectella	MM13620	LEFIG007-10	HM875687	BOLD:AAD3661	Finland	University of Oulu
Bryotropha terrella	MM12146	LEFIF518-10	HM875203	BOLD:AAB9591	Finland	University of Oulu
Bryotropha terrella	MM21185	LEFIJ1325-11	KM572170	BOLD:AAB9591	Finland	University of Oulu
Bryotropha terrella	TLMF Lep 08043	PHLAV224-12	KM572567	BOLD:AAB9591	Austria	inatura, Dornbirn
Bryotropha terrella	MM02595	LEFIB749-10	HM871627	BOLD:AAB9591	Finland	University of Oulu
Bryotropha terrella	MM09720	LEFIA844-10	HM386984	BOLD:AAB9591	Finland	University of Oulu
Bryotropha terrella	MM19878	LEEUA470-11	JN270944	BOLD:AAB9591	Finland	University of Oulu
Bucculatrix bechsteinella	MM02642	LEFIB771-10	HM871648	BOLD:AAE8504	Finland	University of Oulu
Bucculatrix bechsteinella	MM14829	LEFIG615-10	HM876281	BOLD:AAE8504	Finland	University of Oulu
Bucculatrix bechsteinella	MM06181	LEFID303-10	HM873100	BOLD:AAE8504	Finland	University of Oulu
Bucculatrix bechsteinella	TLMF Lep 08482	PHLAH663-12	KM572037	BOLD:AAE8504	Austria	inatura, Dornbirn
Bucculatrix bechsteinella	MM13757	LEFIG089-10	HM875768	BOLD:AAE8504	Finland	University of Oulu
Bucculatrix cidarella	MM05732	LEFID109-10	HM872920	BOLD:AAI3853	Finland	University of Oulu
Bucculatrix cidarella	MM08606	LEFIE268-10	HM874001	BOLD:AAI3853	Finland	University of Oulu
Bucculatrix cidarella	MM16607	LEFIJ1031-11	KM573380	BOLD:AAI3853	Finland	University of Oulu
Bucculatrix cidarella	MM02655	LEFIB780-10	HM871657	BOLD:AAI3853	Finland	University of Oulu
Bucculatrix cidarella	MM06180	LEFID302-10	HM873099	BOLD:AAI3853	Finland	University of Oulu
Bucculatrix cidarella	TLMF Lep 08233	PHLAH414-12	KM573529	BOLD:AAU3108	Austria	inatura, Dornbirn
Bucculatrix demaryella	MM23175	LEFIJ2090-13	KM572358	BOLD:AAI8481	Finland	University of Oulu

Bucculatrix demaryella	MM06182	LEFID304-10	HM873101	BOLD:AAI8481	Finland	University of Oulu
Bucculatrix demaryella	TLMF Lep 07476	PHLAG797-12	KM573514	BOLD:AAI8481	Austria	inatura, Dornbirn
Bucculatrix demaryella	MM05482	LEFIA1030-10	GU828720	BOLD:AAI8481	Finland	University of Oulu
Bucculatrix demaryella	TLMF Lep 08074	PHLAV255-12	KM573152	BOLD:AAI8481	Austria	inatura, Dornbirn
Bucculatrix demaryella	BIOUG04118-A06	GMFID726-12	KM572236	BOLD:AAI8481	Finland	Biodiversity Institute of Ontario
Bucculatrix demaryella	BIOUG04118-A01	GMFID721-12	KM573596	BOLD:AAI8481	Finland	Biodiversity Institute of Ontario
Bucculatrix demaryella	MM15169	LEFIJ287-10	JF853524	BOLD:AAI8481	Finland	University of Oulu
Bucculatrix frangutella	TLMF Lep 08063	PHLAV244-12	KM573090	BOLD:AAI8491	Austria	inatura, Dornbirn
Bucculatrix frangutella	MM18082	LEFIK507-10	KM573140	BOLD:AAI8491	Finland	University of Oulu
Bucculatrix frangutella	MM18081	LEFIK506-10	KM572943	BOLD:AAI8491	Finland	University of Oulu
Bucculatrix frangutella	MM06179	LEFID301-10	HM873098	BOLD:AAI8491	Finland	University of Oulu
Bucculatrix nigricomella	MM06615	LEFID602-10	HM873367	BOLD:AAE8470	Finland	University of Oulu
Bucculatrix nigricomella	MM09948	LEFIE777-10	HM874496	BOLD:AAE8470	Finland	University of Oulu
Bucculatrix nigricomella	TLMF Lep 08408	PHLAH589-12	KM573294	BOLD:AAE8470	Austria	inatura, Dornbirn
Bucculatrix nigricomella	MM02626	LEFIB763-10	HM871640	BOLD:AAE8470	Finland	University of Oulu
Bucculatrix thoracella	MM18084	LEFIK509-10	JF854111	BOLD:AAE8468	Finland	University of Oulu
Bucculatrix thoracella	MM15537	LEFIG673-10	HQ963163	BOLD:AAE8468	Finland	University of Oulu
Bucculatrix thoracella	TLMF Lep 07955	PHLAV136-12	KM572743	BOLD:AAE8468	Austria	inatura, Dornbirn
Bucculatrix thoracella	MM15538	LEFIG674-10	HM876334	BOLD:AAE8468	Finland	University of Oulu
Buckleria paludum	MM17614	LEFIK039-10	KM572572	BOLD:AAI8483	Finland	University of Oulu
Buckleria paludum	MM17615	LEFIK040-10	KM572321	BOLD:AAI8483	Finland	University of Oulu
Buckleria paludum	TLMF Lep 09944	PHLAW147-13	KM572710	BOLD:AAI8483	Austria	inatura, Dornbirn
Buckleria paludum	MM09290	LEFIJ1015-11	KM572086	BOLD:AAI8483	Finland	University of Oulu
Buckleria paludum	MM21110	LEFIJ1250-11	KM572117	BOLD:AAI8483	Finland	University of Oulu
Bupalus piniaria	MM05427	LEFID006-10	HM872820	BOLD:AAC7293	Finland	University of Oulu
Bupalus piniaria	TLMF Lep 07839	PHLAV020-12	KM572091	BOLD:AAC7293	Austria	inatura, Dornbirn
Bupalus piniaria	MM12778	LEFIF718-10	HM875402	BOLD:AAC7293	Finland	University of Oulu
Bupalus piniaria	MM09470	LEFIE575-10	HM874298	BOLD:AAC7293	Finland	University of Oulu
Cabera exanthemata	MM05166	LEFIC883-10	HM872701	BOLD:AAA6653	Finland	University of Oulu
Cabera exanthemata	TLMF Lep 07909	PHLAV090-12	KM572917	BOLD:AAA6653	Austria	inatura, Dornbirn
Cabera exanthemata	MM00640	LEFIB199-10	HM871103	BOLD:AAA6653	Finland	University of Oulu

Cabera exanthemata	MM01306	LEFIA267-10	HM386611	BOLD:AAA6653	Finland	University of Oulu
Cabera pusaria	BIOUG04116-C06	GMFIH296-12	KM573611	BOLD:AAA9589	Finland	Biodiversity Institute of Ontario
Cabera pusaria	BIOUG04118-B03	GMFIF764-12	KM573338	BOLD:AAA9589	Finland	Biodiversity Institute of Ontario
Cabera pusaria	MM01304	LEFIA265-10	HM386609	BOLD:AAA9589	Finland	University of Oulu
Cabera pusaria	TLMF Lep 04658	PHLAE343-11	JN269340	BOLD:AAA9589	Austria	Tiroler Landesmuseum Ferdinandeum
Cabera pusaria	BIOUG04118-E03	GMFIO716-13	KM572659	BOLD:AAA9589	Finland	Biodiversity Institute of Ontario
Cabera pusaria	BIOUG04118-E04	GMFIP249-13	KM573361	BOLD:AAA9589	Finland	Biodiversity Institute of Ontario
Cabera pusaria	BIOUG04116-F02	GMFIQ246-13	KM572926	BOLD:AAA9589	Finland	Biodiversity Institute of Ontario
Cabera pusaria	TLMF Lep 08127	PHLAV308-12	KM572447	BOLD:AAA9589	Austria	inatura, Dornbirn
Cabera pusaria	MM01305	LEFIA266-10	HM386610	BOLD:AAA9589	Finland	University of Oulu
Cabera pusaria	BIOUG04116-C07	GMFII237-12	KM572279	BOLD:AAA9589	Finland	Biodiversity Institute of Ontario
Cabera pusaria	MM00589	LEFIB176-10	HM871080	BOLD:AAA9589	Finland	University of Oulu
Cabera pusaria	BIOUG04116-C08	GMFII238-12	KM572033	BOLD:AAA9589	Finland	Biodiversity Institute of Ontario
Calamotropha paludella	MM01876	LEFIB409-10	HM871308	BOLD:AAC7277	Finland	University of Oulu
Calamotropha paludella	MM03502	LEFIC156-10	HM872002	BOLD:AAC7277	Finland	University of Oulu
Calamotropha paludella	TLMF Lep 07532	PHLAG853-12	KM573320	BOLD:AAC7277	Austria	Tiroler Landesmuseum Ferdinandeum
Calamotropha paludella	MM13014	LEFIF780-10	HM875464	BOLD:AAC7277	Finland	University of Oulu
Callimorpha dominula	MM17454	LEFIJ829-10	JF853849	BOLD:AAD5343	Finland	University of Oulu
Callimorpha dominula	TLMF Lep 00722	PHLAA682-09	HM381463	BOLD:AAD5343	Austria	Tiroler Landesmuseum Ferdinandeum
Callimorpha dominula	MM17453	LEFIJ828-10	JF853848	BOLD:AAD5343	Finland	University of Oulu
Callisto coffeella	MM08573	LEFIE246-10	HM873980	BOLD:AAE0738	Finland	University of Oulu
Callisto coffeella	MM18091	LEFIK516-10	JN271968	BOLD:AAE0738	Finland	University of Oulu
Callisto coffeella	TLMF Lep 00939	PHLAB139-10	HQ968444	BOLD:AAE0738	Austria	Tiroler Landesmuseum Ferdinandeum
Callisto coffeella	MM08523	LEFIE208-10	HQ570366	BOLD:AAE0738	Finland	University of Oulu
Callisto coffeella	MM08522	LEFIE207-10	HQ570365	BOLD:AAE0738	Finland	University of Oulu
Calliteara pudibunda	MM00859	LEFIB323-10	HM871223	BOLD:AAB5204	Finland	University of Oulu
Calliteara pudibunda	MM01046	LEFIA121-10	HM396468	BOLD:AAB5204	Finland	University of Oulu
Calliteara pudibunda	MM01045	LEFIA120-10	HM396467	BOLD:AAB5204	Finland	University of Oulu
Calliteara pudibunda	TLMF Lep 07827	PHLAV008-12	KM573544	BOLD:AAB5204	Austria	inatura, Dornbirn
Callophrys rubi	MM05430	LEFID008-10	HM872822	BOLD:AAB0049	Finland	University of Oulu
Callophrys rubi	MM00386	LEFIB084-10	HM870993	BOLD:AAB0049	Finland	University of Oulu

Callophrys rubi	MM17118	LEFIJ493-10	JF853618	BOLD:AAB0049	Finland	University of Oulu
Callophrys rubi	TLMF Lep 10007	LEATA400-13	KM572775	BOLD:AAB0049	Austria	inatura, Dornbirn
Caloptilia alchimiella	MM18086	LEFIK511-10	JF854113	BOLD:AAD2588	Finland	University of Oulu
Caloptilia alchimiella	MM03829	LEFIC325-10	HM872169	BOLD:AAD2588	Finland	University of Oulu
Caloptilia alchimiella	MM11151	LEFIF337-10	HM875022	BOLD:AAD2588	Finland	University of Oulu
Caloptilia alchimiella	TLMF Lep 07940	PHLAV121-12	KM573491	BOLD:AAD2588	Austria	inatura, Dornbirn
Caloptilia cuculipennella	TLMF Lep 07943	PHLAV124-12	KM572476	BOLD:AAF8193	Austria	inatura, Dornbirn
Caloptilia cuculipennella	MM08518	LEFIE203-10	HQ570363	BOLD:AAF8193	Finland	University of Oulu
Caloptilia cuculipennella	MM08517	LEFIE202-10	HQ570362	BOLD:AAF8193	Finland	University of Oulu
Caloptilia cuculipennella	MM10698	LEFIF164-10	HM874858	BOLD:AAF8193	Finland	University of Oulu
Caloptilia elongella	MM05329	LEFIC958-10	HM872773	BOLD:AAE3456	Finland	University of Oulu
Caloptilia elongella	MM00447	LEFIB109-10	HM871016	BOLD:AAE3456	Finland	University of Oulu
Caloptilia elongella	MM02459	LEFIB673-10	HM871551	BOLD:AAE3456	Finland	University of Oulu
Caloptilia elongella	TLMF Lep 09960	PHLAW163-13	KM572630	BOLD:AAE3456	Austria	inatura, Dornbirn
Caloptilia elongella	MM09017	LEFIE420-10	HM874144	BOLD:AAE3456	Finland	University of Oulu
Caloptilia elongella	TLMF Lep 07958	PHLAV139-12	KM572411	BOLD:AAE3456	Austria	inatura, Dornbirn
Caloptilia elongella	MM06153	LEFID279-10	HM873077	BOLD:AAE3456	Finland	University of Oulu
Caloptilia robustella	TLMF Lep 07941	PHLAV122-12	KM572827	BOLD:AAD2587	Austria	inatura, Dornbirn
Caloptilia robustella	MM05652	LEFID053-10	HM872867	BOLD:AAD2587	Finland	University of Oulu
Caloptilia robustella	MM08519	LEFIE204-10	HM873946	BOLD:AAD2587	Finland	University of Oulu
Caloptilia robustella	MM17310	LEFIJ685-10	KM573711	BOLD:AAD2587	Finland	University of Oulu
Caloptilia robustella	MM03461	LEFIC124-10	HM871971	BOLD:AAD2587	Finland	University of Oulu
Caloptilia stigmatella	MM05355	LEFIC964-10	HM872779	BOLD:AAA9984	Finland	University of Oulu
Caloptilia stigmatella	MM00619	LEFIB190-10	HM871094	BOLD:AAA9984	Finland	University of Oulu
Caloptilia stigmatella	MM00462	LEFIB114-10	HM871021	BOLD:AAA9984	Finland	University of Oulu
Caloptilia stigmatella	TLMF Lep 08437	PHLAH618-12	KM572520	BOLD:AAA9984	Austria	inatura, Dornbirn
Caloptilia stigmatella	MM05426	LEFIA1034-10	GU828718	BOLD:AAA9984	Finland	University of Oulu
Calybites phasianipennella	TLMF Lep 08452	PHLAH633-12	KM572140	BOLD:AAD6298	Austria	inatura, Dornbirn
Calybites phasianipennella	MM00437	LEFIA038-10	HM396387	BOLD:AAD6298	Finland	University of Oulu
Calybites phasianipennella	MM10405	LEFIE976-10	HM874693	BOLD:AAD6298	Finland	University of Oulu
Campaea margaritaria	MM06747	LEFID696-10	HM873457	BOLD:AAB5336	Finland	University of Oulu

Campaea margaritaria	MM09755	LEFIA878-10	HM387016	BOLD:AAB5336	Finland	University of Oulu
Campaea margaritaria	TLMF Lep 07907	PHLAV088-12	KM573589	BOLD:AAB5336	Austria	inatura, Dornbirn
Campaea margaritaria	MM09754	LEFIA877-10	HM387015	BOLD:AAB5336	Finland	University of Oulu
Camptogramma bilineata	MM03540	LEFIC184-10	HM872028	BOLD:AAA8017	Finland	University of Oulu
Camptogramma bilineata	MM12329	LEFIF571-10	HM875256	BOLD:AAA8017	Finland	University of Oulu
Camptogramma bilineata	TLMF Lep 08131	PHLAV312-12	KM572306	BOLD:AAA8017	Austria	inatura, Dornbirn
Camptogramma bilineata	MM03702	LEFIC270-10	HM872114	BOLD:AAA8017	Finland	University of Oulu
Capua vulgana	MM03806	LEFIC309-10	HM872153	BOLD:AAD5387	Finland	University of Oulu
Capua vulgana	MM14182	LEFIG257-10	HM875936	BOLD:AAD5387	Finland	University of Oulu
Capua vulgana	BIOUG04116-A01	GMFID718-12	KM572271	BOLD:AAD5387	Finland	Biodiversity Institute of Ontario
Capua vulgana	MM03925	LEFIC388-10	HM872231	BOLD:AAD5387	Finland	University of Oulu
Capua vulgana	TLMF Lep 07933	PHLAV114-12	KM572226	BOLD:AAD5387	Austria	inatura, Dornbirn
Carpatolechia alburnella	MM12437	LEFIF591-10	HM875275	BOLD:AAF1850	Finland	University of Oulu
Carpatolechia alburnella	MM03556	LEFIC195-10	HM872039	BOLD:AAF1850	Finland	University of Oulu
Carpatolechia alburnella	TLMF Lep 12468	LEATC486-13	KM573202	BOLD:AAF1850	Austria	Tiroler Landesmuseum Ferdinandeum
Carpatolechia alburnella	MM09805	LEFIE716-10	HM874436	BOLD:AAF1850	Finland	University of Oulu
Carpatolechia fugitivella	TLMF Lep 08076	PHLAV257-12	KM573249	BOLD:AAA7652	Austria	inatura, Dornbirn
Carpatolechia fugitivella	MM15625	LEFIG761-10	HM876414	BOLD:AAA7652	Finland	University of Oulu
Carpatolechia fugitivella	TLMF Lep 08298	PHLAH479-12	KM572147	BOLD:AAA7652	Austria	inatura, Dornbirn
Carpatolechia fugitivella	MM15624	LEFIG760-10	HM876413	BOLD:AAA7652	Finland	University of Oulu
Carpatolechia fugitivella	MM12021	LEFIF475-10	HM875160	BOLD:AAA7652	Finland	University of Oulu
Carsia sororiata	MM02919	LEFIB866-10	HM871743	BOLD:AAC1640	Finland	University of Oulu
Carsia sororiata	MM08117	LEFIE036-10	HM873785	BOLD:AAC1640	Finland	University of Oulu
Carsia sororiata	TLMF Lep 12550	LEATC568-13	KM573561	BOLD:AAC1640	Austria	Tiroler Landesmuseum Ferdinandeum
Carsia sororiata	MM01438	LEFIA381-10	HM386723	BOLD:AAC1640	Finland	University of Oulu
Carterocephalus palaemon	MM10323	LEFIE936-10	HM874653	BOLD:AAA6267	Finland	University of Oulu
Carterocephalus palaemon	MM00511	LEFIB139-10	HM871044	BOLD:AAA6267	Finland	University of Oulu
Carterocephalus palaemon	MM17103	LEFIJ478-10	JF853606	BOLD:AAA6267	Finland	University of Oulu

Carterocephalus palaemon	MM17104	LEFIJ479-10	JF853607	BOLD:AAA6267	Finland	University of Oulu
Carterocephalus palaemon	TLMF Lep 09816	PHLAW019-13	KM573246	BOLD:AAA6267	Austria	Tiroler Landesmuseum Ferdinandeum
Carterocephalus palaemon	MM10322	LEFIE935-10	HM874652	BOLD:AAA6267	Finland	University of Oulu
Caryocolum cassella	MM09699	LEFIE707-10	HM874428	BOLD:AAE9472	Finland	University of Oulu
Caryocolum cassella	MM12412	LEFIF586-10	HM875270	BOLD:AAE9472	Finland	University of Oulu
Caryocolum cassella	TLMF Lep 04530	PHLAE405-11	KJ427045	BOLD:AAE9472	Austria	Tiroler Landesmuseum Ferdinandeum
Caryocolum cassella	MM09575	LEFIE633-10	HM874356	BOLD:AAE9472	Finland	University of Oulu
Caryocolum cassella	MM08149	LEFIE046-10	HM873794	BOLD:AAE9472	Finland	University of Oulu
Cataclysta lemnata	MM10011	LEFIE812-10	HM874531	BOLD:AAC2080	Finland	University of Oulu
Cataclysta lemnata	TLMF Lep 08208	PHLAH389-12	KM572687	BOLD:AAC2080	Austria	inatura, Dornbirn
Cataclysta lemnata	MM08808	LEFIE362-10	HM874086	BOLD:AAC2080	Finland	University of Oulu
Cataclysta lemnata	MM06141	LEFID270-10	HM873068	BOLD:AAC2080	Finland	University of Oulu
Catarhoe cuculata	TLMF Lep 04641	PHLAE326-11	JN279571	BOLD:AAC2047	Austria	Tiroler Landesmuseum Ferdinandeum
Catarhoe cuculata	TLMF Lep 08114	PHLAV295-12	KM573517	BOLD:AAC2047	Austria	inatura, Dornbirn
Catarhoe cuculata	MM01359	LEFIA313-10	HM386656	BOLD:AAC2047	Finland	University of Oulu
Catarhoe cuculata	MM01358	LEFIA312-10	HM386655	BOLD:AAC2047	Finland	University of Oulu
Catarhoe cuculata	MM06718	LEFID677-10	HM873438	BOLD:AAC2047	Finland	University of Oulu
Catastia marginea	MM10534	LEFIF085-10	HQ570381	BOLD:AAE9528	Finland	University of Oulu
Catastia marginea	MM10535	LEFIF086-10	HQ570382	BOLD:AAE9528	Finland	University of Oulu
Catastia marginea	TLMF Lep 08432	PHLAH613-12	KM572379	BOLD:AAE9528	Austria	inatura, Dornbirn
Catastia marginea	MM10536	LEFIF087-10	HQ570383	BOLD:AAE9528	Finland	University of Oulu
Catocala fraxini	MM02788	LEFIB832-10	HM871709	BOLD:AAC6489	Finland	University of Oulu
Catocala fraxini	TLMF Lep 12607	LEATC625-13	KM572176	BOLD:AAC6489	Austria	Tiroler Landesmuseum Ferdinandeum
Catocala fraxini	MM01839	LEFIA694-10	HM386839	BOLD:AAC6489	Finland	University of Oulu
Catocala fraxini	MM01840	LEFIA695-10	HM386840	BOLD:AAC6489	Finland	University of Oulu
Catocala nupta	MM15851	LEFIG987-10	HM876626	BOLD:ACE8723	Finland	University of Oulu
Catocala nupta	TLMF Lep 09855	PHLAW058-13	KM572102	BOLD:ACE8723	Austria	Tiroler Landesmuseum Ferdinandeum
Catocala nupta	MM04216	LEFIC508-10	HM872333	BOLD:ACE8723	Finland	University of Oulu

Catocala nupta	MM04750	LEFIC710-10	HM872531	BOLD:ACE8723	Finland	University of Oulu
Catoptria falsella	MM04958	LEFIC800-10	HM872619	BOLD:AAC2294	Finland	University of Oulu
Catoptria falsella	MM13050	LEFIF796-10	HM875480	BOLD:AAC2294	Finland	University of Oulu
Catoptria falsella	MM01920	LEFIB432-10	HM871331	BOLD:AAC2294	Finland	University of Oulu
Catoptria falsella	TLMF Lep 08742	PHLAH938-12	KM573127	BOLD:AAC2294	Austria	Tiroler Landesmuseum Ferdinandeum
Catoptria permutatellus	MM13028	LEFIF788-10	HM875472	BOLD:AAC2957	Finland	University of Oulu
Catoptria permutatellus	MM02950	LEFIB877-10	HM871754	BOLD:AAC2957	Finland	University of Oulu
Catoptria permutatellus	TLMF Lep 00911	PHLAB111-10	HM381483	BOLD:AAC2957	Austria	Tiroler Landesmuseum Ferdinandeum
Catoptria permutatellus	MM01899	LEFIB420-10	HM871319	BOLD:AAC2957	Finland	University of Oulu
Catoptria permutatellus	MM09246	LEFIE473-10	HM874197	BOLD:AAC2957	Finland	University of Oulu
Catoptria verellus	MM03554	LEFIC193-10	HM872037	BOLD:AAD5840	Finland	University of Oulu
Catoptria verellus	MM06763	LEFID709-10	HM873470	BOLD:AAD5840	Finland	University of Oulu
Catoptria verellus	MM01922	LEFIB433-10	HM871332	BOLD:AAD5840	Finland	University of Oulu
Catoptria verellus	TLMF Lep 08223	PHLAH404-12	KM573515	BOLD:AAD5840	Austria	inatura, Dornbirn
Cedestis gysseleniella	MM00675	LEFIB221-10	HM871125	BOLD:AAE4109	Finland	University of Oulu
Cedestis gysseleniella	MM13735	LEFIG082-10	HM875761	BOLD:AAE4109	Finland	University of Oulu
Cedestis gysseleniella	TLMF Lep 08071	PHLAV252-12	KM573264	BOLD:AAE4109	Austria	inatura, Dornbirn
Cedestis gysseleniella	MM02540	LEFIB721-10	HM871599	BOLD:AAE4109	Finland	University of Oulu
Celastrina argiolus	MM00488	LEFIB124-10	HM871030	BOLD:AAA7663	Finland	University of Oulu
Celastrina argiolus	TLMF Lep 09824	PHLAW027-13	KM573716	BOLD:AAA7663	Austria	Tiroler Landesmuseum Ferdinandeum
Celastrina argiolus	MM17136	LEFIJ511-10	JF853633	BOLD:AAA7663	Finland	University of Oulu
Celastrina argiolus	MM17137	LEFIJ512-10	JF853634	BOLD:AAA7663	Finland	University of Oulu
Celypha cespitana	MM06764	LEFID710-10	HM873471	BOLD:AAA9471	Finland	University of Oulu
Celypha cespitana	MM03205	LEFIC005-10	HM871875	BOLD:AAA9471	Finland	University of Oulu
Celypha cespitana	MM06545	LEFID556-10	HM873321	BOLD:AAA9471	Finland	University of Oulu
Celypha cespitana	MM05284	LEFIC929-10	HM872745	BOLD:AAA9471	Finland	University of Oulu
Celypha cespitana	MM11829	LEFIF420-10	HM875105	BOLD:AAA9471	Finland	University of Oulu
Celypha cespitana	MM23196	COLFF463-13	KM573059	BOLD:AAA9471	Finland	University of Oulu, Zoological Museum
Celypha cespitana	MM18830	LEFIL532-10	JF854644	BOLD:AAA9471	Finland	University of Oulu
Celypha cespitana	MM13295	LEFIF889-10	HM875571	BOLD:AAA9471	Finland	University of Oulu

Celypha cespitana	TLMF Lep 10039	LEATA432-13	KM572228	BOLD:AAA9471	Austria	inatura, Dornbirn
Celypha lacunana	TLMF Lep 07988	PHLAV169-12	KM572254	BOLD:AAC3531	Austria	inatura, Dornbirn
Celypha lacunana	MM23076	LEFIJ1789-13	KM572861	BOLD:AAC3531	Finland	University of Oulu
Celypha lacunana	MM23083	LEFIJ1796-13	KM573426	BOLD:AAC3531	Finland	University of Oulu
Celypha lacunana	MM23081	LEFIJ1794-13	KM572722	BOLD:AAC3531	Finland	University of Oulu
Celypha lacunana	MM23079	LEFIJ1792-13	KM573044	BOLD:AAC3531	Finland	University of Oulu
Celypha lacunana	MM23075	LEFIJ1788-13	KM572681	BOLD:AAC3531	Finland	University of Oulu
Celypha lacunana	MM03003	LEFIB901-10	HM871778	BOLD:AAC3531	Finland	University of Oulu
Celypha lacunana	MM23077	LEFIJ1790-13	KM573102	BOLD:AAC3531	Finland	University of Oulu
Celypha lacunana	MM02070	LEFIB493-10	HM871388	BOLD:AAC3531	Finland	University of Oulu
Celypha lacunana	TLMF Lep 07930	PHLAV111-12	KM573671	BOLD:AAC3531	Austria	inatura, Dornbirn
Celypha lacunana	MM23074	LEFIJ1787-13	KM573125	BOLD:ACE8186	Finland	University of Oulu
Celypha lacunana	MM13309	LEFIF897-10	HM875579	BOLD:ACE8186	Finland	University of Oulu
Celypha lacunana	MM23082	LEFIJ1795-13	KM573114	BOLD:ACE8186	Finland	University of Oulu
Celypha rivulana	MM03001	LEFIB900-10	HM871777	BOLD:AAF2534	Finland	University of Oulu
Celypha rivulana	TLMF Lep 08020	PHLAV201-12	KM573271	BOLD:AAF2534	Austria	inatura, Dornbirn
Celypha rivulana	MM13292	LEFIF886-10	HM875568	BOLD:AAF2534	Finland	University of Oulu
Celypha rivulana	MM02072	LEFIB494-10	HM871389	BOLD:AAF2534	Finland	University of Oulu
Celypha rurestrana	MM05267	LEFIC923-10	HM872740	BOLD:ABX5517	Finland	University of Oulu
Celypha rurestrana	MM09992	LEFIE803-10	HM874522	BOLD:ABX5517	Finland	University of Oulu
Celypha rurestrana	TLMF Lep 09172	PHLAI610-13	KM572300	BOLD:ABX5517	Austria	Tiroler Landesmuseum Ferdinandeum
Celypha rurestrana	MM03011	LEFIB907-10	HM871784	BOLD:ABX5517	Finland	University of Oulu
Cepphis advenaria	MM01323	LEFIA283-10	HM386627	BOLD:AAC7380	Finland	University of Oulu
Cepphis advenaria	MM11594	LEFIF372-10	HM875057	BOLD:AAC7380	Finland	University of Oulu
Cepphis advenaria	MM01335	LEFIA291-10	HM386634	BOLD:AAC7380	Finland	University of Oulu
Cepphis advenaria	MM01324	LEFIA284-10	HM386628	BOLD:AAC7380	Finland	University of Oulu
Cepphis advenaria	TLMF Lep 08152	PHLAV333-12	KM572800	BOLD:AAC7380	Austria	inatura, Dornbirn
Ceramica pisi	MM10885	LEFIF210-10	HM874904	BOLD:AAB0758	Finland	University of Oulu
Ceramica pisi	TLMF Lep 07835	PHLAV016-12	KM572900	BOLD:AAB0758	Austria	inatura, Dornbirn
Ceramica pisi	MM04552	LEFIC618-10	HM872439	BOLD:AAB0758	Finland	University of Oulu
Ceramica pisi	MM00826	LEFIB306-10	HM871207	BOLD:AAB0758	Finland	University of Oulu

Cerapteryx graminis	MM00812	LEFIB299-10	HM871200	BOLD:AAB4284	Finland	University of Oulu
Cerapteryx graminis	MM01612	LEFIA528-10	KM572110	BOLD:AAB4284	Finland	University of Oulu
Cerapteryx graminis	MM08079	LEFIE021-10	HM873770	BOLD:AAB4284	Finland	University of Oulu
Cerapteryx graminis	MM01613	LEFIA529-10	KM573093	BOLD:AAB4284	Finland	University of Oulu
Cerapteryx graminis	TLMF Lep 04617	PHLAE302-11	KM573410	BOLD:AAB4284	Austria	Tiroler Landesmuseum Ferdinandeum
Cerastis leucographa	MM04038	LEFIC455-10	HM872289	BOLD:AAD9079	Finland	University of Oulu
Cerastis leucographa	MM01579	LEFIA497-10	KM572983	BOLD:AAD9079	Finland	University of Oulu
Cerastis leucographa	TLMF Lep 04649	PHLAE334-11	JN284170	BOLD:AAD9079	Austria	Tiroler Landesmuseum Ferdinandeum
Cerastis leucographa	MM01578	LEFIA496-10	KM572865	BOLD:AAD9079	Finland	University of Oulu
Cerastis rubricosa	MM01575	LEFIA494-10	KM572473	BOLD:AAD2637	Finland	University of Oulu
Cerastis rubricosa	MM01576	LEFIA495-10	KM572380	BOLD:AAD2637	Finland	University of Oulu
Cerastis rubricosa	MM00410	LEFIB099-10	HM871006	BOLD:AAD2637	Finland	University of Oulu
Cerastis rubricosa	TLMF Lep 04622	PHLAE307-11	JN284169	BOLD:AAD2637	Austria	Tiroler Landesmuseum Ferdinandeum
Cerura vinula	MM18517	LEFIK942-10	JF854382	BOLD:AAB7277	Finland	University of Oulu
Cerura vinula	MM10749	LEFIF178-10	HM874872	BOLD:AAB7277	Finland	University of Oulu
Cerura vinula	MM00980	LEFIA067-10	HM396414	BOLD:AAB7277	Finland	University of Oulu
Cerura vinula	TLMF Lep 06180	PHLSA725-11	KM573250	BOLD:AAB7277	Austria	Tiroler Landesmuseum Ferdinandeum
Cerura vinula	TLMF Lep 07549	PHLAG870-12	KM573549	BOLD:AAB7277	Austria	inatura, Dornbirn
Cerura vinula	MM11053	LEFIF271-10	HM874964	BOLD:AAB7277	Finland	University of Oulu
Cerura vinula	MM05422	LEFID002-10	HM872816	BOLD:AAB7277	Finland	University of Oulu
Cerura vinula	MM18516	LEFIK941-10	JF854381	BOLD:AAB7277	Finland	University of Oulu
Charanyca ferruginea	MM01538	LEFIA467-10	HM386807	BOLD:AAB9497	Finland	University of Oulu
Charanyca ferruginea	MM01539	LEFIA468-10	HM386808	BOLD:AAB9497	Finland	University of Oulu
Charanyca ferruginea	MM05144	LEFIC871-10	HM872689	BOLD:AAB9497	Finland	University of Oulu
Charanyca ferruginea	TLMF Lep 06160	PHLSA705-11	KM572328	BOLD:AAB9497	Austria	Tiroler Landesmuseum Ferdinandeum
Charanyca ferruginea	TLMF Lep 08138	PHLAV319-12	KM572016	BOLD:AAB9497	Austria	inatura, Dornbirn
Charanyca trigrammica	MM14288	LEFIG320-10	HM875999	BOLD:AAB4764	Finland	University of Oulu
Charanyca trigrammica	MM12562	LEFIF649-10	HM875333	BOLD:AAB4764	Finland	University of Oulu
Charanyca trigrammica	TLMF Lep 07913	PHLAV094-12	KM572446	BOLD:AAB4764	Austria	inatura, Dornbirn
Charanyca trigrammica	MM14315	LEFIG335-10	HM876014	BOLD:AAB4764	Finland	University of Oulu
Chersotis cuprea	TLMF Lep 04616	PHLAE301-11	JN284167	BOLD:AAD0415	Austria	Tiroler Landesmuseum Ferdinandeum

Chersotis cuprea	MM01721	LEFIA609-10	HM870858	BOLD:AAD0415	Finland	University of Oulu
Chersotis cuprea	MM01720	LEFIA608-10	HM870857	BOLD:AAD0415	Finland	University of Oulu
Chersotis cuprea	MM02759	LEFIB821-10	HM871698	BOLD:AAD0415	Finland	University of Oulu
Chiasmia clathrata	MM01278	LEFIA245-10	HM386589	BOLD:AAB0547	Finland	University of Oulu
Chiasmia clathrata	MM01279	LEFIA246-10	HM386590	BOLD:AAB0547	Finland	University of Oulu
Chiasmia clathrata	BIOUG04490-F09	GMFIM275-13	KM572480	BOLD:AAB0547	Finland	Biodiversity Institute of Ontario
Chiasmia clathrata	TLMF Lep 07842	PHLAV023-12	KM573570	BOLD:AAB0547	Austria	inatura, Dornbirn
Chiasmia clathrata	MM13945	LEFIG130-10	HM875810	BOLD:AAB0547	Finland	University of Oulu
Chilo phragmitella	MM09750	LEFIA873-10	HM387011	BOLD:AAC4987	Finland	University of Oulu
Chilo phragmitella	TLMF Lep 08417	PHLAH598-12	KM572944	BOLD:AAC4987	Austria	inatura, Dornbirn
Chilo phragmitella	MM12991	LEFIF770-10	HM875454	BOLD:AAC4988	Finland	University of Oulu
Chilo phragmitella	MM14190	LEFIG259-10	HM875938	BOLD:AAC4988	Finland	University of Oulu
Chilo phragmitella	MM02448	LEFIB667-10	HM871545	BOLD:AAC4988	Finland	University of Oulu
Chionodes electella	MM09791	LEFIA913-10	HM387049	BOLD:AAD4848	Finland	University of Oulu
Chionodes electella	MM00691	LEFIB231-10	HM871134	BOLD:AAD4848	Finland	University of Oulu
Chionodes electella	TLMF Lep 08297	PHLAH478-12	KM572866	BOLD:AAD4848	Austria	inatura, Dornbirn
Chionodes electella	MM10148	LEFIE876-10	HM874594	BOLD:AAD4848	Finland	University of Oulu
Chionodes electella	TLMF Lep 08244	PHLAH425-12	KM572845	BOLD:AAD4848	Austria	inatura, Dornbirn
Chionodes fumatella	MM21189	LEFIJ1329-11	KM572021	BOLD:AAC7811	Finland	University of Oulu
Chionodes fumatella	MM03501	LEFIC155-10	HM872001	BOLD:AAC7811	Finland	University of Oulu
Chionodes fumatella	MM18653	LEFIL343-10	JN270957	BOLD:AAC7811	Finland	University of Oulu
Chionodes fumatella	MM17252	LEFIJ627-10	JF853725	BOLD:AAC7811	Finland	University of Oulu
Chionodes fumatella	MM13546	LEFIJ233-10	KM373605	BOLD:AAC7811	Finland	University of Oulu
Chionodes fumatella	MM13633	LEFIJ235-10	KM373599	BOLD:AAC7811	Finland	University of Oulu
Chionodes fumatella	MM03589	LEFIJ057-10	JF853419	BOLD:AAC7811	Finland	University of Oulu
Chionodes fumatella	MM17578	LEFIK003-10	JF853912	BOLD:AAC7811	Finland	University of Oulu
Chionodes fumatella	MM13545	LEFIF965-10	HM875646	BOLD:AAC7811	Finland	University of Oulu
Chionodes fumatella	MM10326	LEFIE938-10	HM874655	BOLD:AAC7811	Finland	University of Oulu
Chionodes fumatella	MM18600	LEFII173-10	JF853382	BOLD:AAC7811	Finland	University of Oulu
Chionodes fumatella	MM18601	LEFII174-10	JF853383	BOLD:AAC7811	Finland	University of Oulu
Chionodes fumatella	MM18605	LEFII178-10	JF853387	BOLD:AAC7811	Finland	University of Oulu

Chionodes fumatella	MM18606	LEFII179-10	JF853388	BOLD:AAC7811	Finland	University of Oulu
Chionodes fumatella	MM18607	LEFII180-10	JF853389	BOLD:AAC7811	Finland	University of Oulu
Chionodes fumatella	MM21190	LEFIJ1330-11	KM572149	BOLD:AAC7811	Finland	University of Oulu
Chionodes fumatella	MM18652	LEFIL342-10	JN270956	BOLD:AAI4278	Finland	University of Oulu
Chionodes fumatella	MM10282	LEFIJ155-10	JF853468	BOLD:AAI4278	Finland	University of Oulu
Chionodes fumatella	MM14516	LEFIG472-10	HM876148	BOLD:AAI4278	Finland	University of Oulu
Chionodes fumatella	MM18602	LEFII175-10	JF853384	BOLD:AAI4278	Finland	University of Oulu
Chionodes fumatella	MM18603	LEFII176-10	JF853385	BOLD:AAI4278	Finland	University of Oulu
Chionodes fumatella	MM18604	LEFII177-10	JF853386	BOLD:AAI4278	Finland	University of Oulu
Chionodes fumatella	TLMF Lep 09149	PHLAI587-13	KM572577	BOLD:ABY4715	Austria	Tiroler Landesmuseum Ferdinandeum
Chionodes holosericella	MM02573	LEFIB739-10	HM871617	BOLD:ABY6213	Finland	University of Oulu
Chionodes holosericella	MM08090	LEFIE025-10	HM873774	BOLD:ABY6213	Finland	University of Oulu
Chionodes holosericella	MM10386	LEFIE969-10	HM874686	BOLD:ABY6213	Finland	University of Oulu
Chionodes holosericella	TLMF Lep 03669	PHLAD494-11	JN271001	BOLD:ABY6213	Austria	Tiroler Landesmuseum Ferdinandeum
Chionodes luctuella	MM09778	LEFIA900-10	HM387036	BOLD:AAD2579	Finland	University of Oulu
Chionodes luctuella	MM10155	LEFIE880-10	HM874598	BOLD:AAD2579	Finland	University of Oulu
Chionodes luctuella	MM00693	LEFIB232-10	HM871135	BOLD:AAD2579	Finland	University of Oulu
Chionodes luctuella	TLMF Lep 01908	PHLAB1108-10	HQ968324	BOLD:ABY8068	Austria	Tiroler Landesmuseum Ferdinandeum
Chionodes luctuella	TLMF Lep 01907	PHLAB1107-10	HQ968323	BOLD:ABY8068	Austria	Tiroler Landesmuseum Ferdinandeum
Chloroclysta miata	TLMF Lep 06146	PHLSA691-11	KM572452	BOLD:AAA9515	Austria	Tiroler Landesmuseum Ferdinandeum
Chloroclysta miata	MM04634	LEFIC669-10	HM872490	BOLD:AAA9515	Finland	University of Oulu
Chloroclysta miata	MM00406	LEFIB097-10	HM871004	BOLD:AAA9515	Finland	University of Oulu
Chloroclysta miata	MM01295	LEFIA258-10	HM386602	BOLD:AAA9515	Finland	University of Oulu
Chloroclysta siterata	MM00874	LEFIB333-10	HM871233	BOLD:AAA9515	Finland	University of Oulu
Chloroclysta siterata	TLMF Lep 06147	PHLSA692-11	KM573060	BOLD:AAA9515	Austria	Tiroler Landesmuseum Ferdinandeum
Chloroclysta siterata	MM12822	LEFIF728-10	HM875412	BOLD:AAA9515	Finland	University of Oulu
Chloroclysta siterata	MM04635	LEFIC670-10	HM872491	BOLD:AAA9515	Finland	University of Oulu
Chloroclystis v-ata	MM17291	LEFIJ666-10	JF853757	BOLD:AAB1832	Finland	University of Oulu
Chloroclystis v-ata	MM10478	LEFIF038-10	HM874749	BOLD:AAB1832	Finland	University of Oulu
Chloroclystis v-ata	TLMF Lep 07877	PHLAV058-12	KM573171	BOLD:AAB1832	Austria	inatura, Dornbirn
Chloroclystis v-ata	MM17292	LEFIJ667-10	JF853758	BOLD:AAB1832	Finland	University of Oulu

Chloroclystis v-ata	MM10481	LEFIF041-10	HM874752	BOLD:AAB1832	Finland	University of Oulu
Chloroclystis v-ata	MM10482	LEFIF042-10	HM874753	BOLD:AAB1832	Finland	University of Oulu
Chloroclystis v-ata	MM10480	LEFIF040-10	HM874751	BOLD:AAB1832	Finland	University of Oulu
Chloroclystis v-ata	MM17241	LEFIJ616-10	JF853716	BOLD:AAB1832	Finland	University of Oulu
Chloroclystis v-ata	MM10479	LEFIF039-10	HM874750	BOLD:AAB1832	Finland	University of Oulu
Chrysoteuchia culmella	MM13049	LEFIF795-10	HM875479	BOLD:AAC0540	Finland	University of Oulu
Chrysoteuchia culmella	TLMF Lep 08214	PHLAH395-12	KM573658	BOLD:AAC0540	Austria	inatura, Dornbirn
Chrysoteuchia culmella	MM00626	LEFIB194-10	HM871098	BOLD:AAC0540	Finland	University of Oulu
Chrysoteuchia culmella	MM01926	LEFIB434-10	HM871333	BOLD:AAC0540	Finland	University of Oulu
Cleora cinctaria	MM04632	LEFIC668-10	HM872489	BOLD:AAC4786	Finland	University of Oulu
Cleora cinctaria	MM01299	LEFIA261-10	HM386605	BOLD:AAC4786	Finland	University of Oulu
Cleora cinctaria	MM04057	LEFIC469-10	HM872302	BOLD:AAC4786	Finland	University of Oulu
Cleora cinctaria	MM00450	LEFIA047-10	HM396396	BOLD:AAC4786	Finland	University of Oulu
Cleora cinctaria	TLMF Lep 04630	PHLAE315-11	JN269336	BOLD:AAC4786	Austria	Tiroler Landesmuseum Ferdinandeum
Clepsis rurinana	TLMF Lep 08086	PHLAV267-12	KM572111	BOLD:AAF4503	Austria	inatura, Dornbirn
Clepsis rurinana	MM06610	LEFID599-10	HM873364	BOLD:AAF4503	Finland	University of Oulu
Clepsis rurinana	MM13272	LEFIF878-10	HM875560	BOLD:AAF4503	Finland	University of Oulu
Clepsis rurinana	MM02009	LEFIB465-10	HM871364	BOLD:AAF4503	Finland	University of Oulu
Clostera anachoreta	TLMF Lep 09506	LEATA089-13	KM572178	BOLD:AAE2573	Austria	Tiroler Landesmuseum Ferdinandeum
Clostera anachoreta	MM14099	LEFIG211-10	HM875891	BOLD:AAE2573	Finland	University of Oulu
Clostera anachoreta	MM07684	LEFID936-10	HM873686	BOLD:AAE2573	Finland	University of Oulu
Clostera anachoreta	MM07683	LEFID935-10	HM873685	BOLD:AAE2573	Finland	University of Oulu
Clostera curtula	MM01003	LEFIA084-10	HM396431	BOLD:AAC5006	Finland	University of Oulu
Clostera curtula	TLMF Lep 07831	PHLAV012-12	KM573569	BOLD:AAC5006	Austria	inatura, Dornbirn
Clostera curtula	MM01004	LEFIA085-10	HM396432	BOLD:AAC5006	Finland	University of Oulu
Clostera curtula	MM04573	LEFIC631-10	HM872452	BOLD:AAC5006	Finland	University of Oulu
Clostera pigra	MM00502	LEFIB133-10	HM871039	BOLD:AAD5412	Finland	University of Oulu
Clostera pigra	TLMF Lep 06126	PHLSA671-11	KM573217	BOLD:AAD5412	Austria	Tiroler Landesmuseum Ferdinandeum
Clostera pigra	MM01008	LEFIA086-10	HM396433	BOLD:AAD5412	Finland	University of Oulu
Clostera pigra	MM12680	LEFIF680-10	HM875364	BOLD:AAD5412	Finland	University of Oulu
Clostera pigra	MM01005	LEFIA1052-10	GU828654	BOLD:AAD5412	Finland	University of Oulu

Cnephasia alticolana	MM08618	LEFIE277-10	HM874009	BOLD:ACF2531	Finland	University of Oulu
Cnephasia alticolana	MM18636	LEFIL326-10	JN286443	BOLD:ACF2531	Finland	University of Oulu
Cnephasia alticolana	MM08619	LEFIE278-10	HM874010	BOLD:ACF2531	Finland	University of Oulu
Cnephasia alticolana	TLMF Lep 08397	PHLAH578-12	KM573081	BOLD:ACF2531	Austria	inatura, Dornbirn
Cnephasia alticolana	MM15662	LEFIG798-10	HM876450	BOLD:ACF2531	Finland	University of Oulu
Cnephasia alticolana	MM15663	LEFIG799-10	HM876451	BOLD:ACF2531	Finland	University of Oulu
Cnephasia asseclana	MM03504	LEFIC158-10	HM872004	BOLD:AAA6293	Finland	University of Oulu
Cnephasia asseclana	MM06548	LEFID557-10	HM873322	BOLD:AAA6293	Finland	University of Oulu
Cnephasia asseclana	MM06712	LEFID672-10	HM873433	BOLD:AAA6293	Finland	University of Oulu
Cnephasia asseclana	MM06678	LEFID651-10	HM873413	BOLD:AAA6293	Finland	University of Oulu
Cnephasia asseclana	MM06679	LEFID652-10	HM873414	BOLD:AAA6293	Finland	University of Oulu
Cnephasia asseclana	MM06680	LEFID653-10	HM873415	BOLD:AAA6293	Finland	University of Oulu
Cnephasia asseclana	MM06681	LEFID654-10	HM873416	BOLD:AAA6293	Finland	University of Oulu
Cnephasia asseclana	MM02140	LEFIB529-10	HM871417	BOLD:AAA6293	Finland	University of Oulu
Cnephasia asseclana	MM11849	LEFIF427-10	HM875112	BOLD:AAA6293	Finland	University of Oulu
Cnephasia asseclana	TLMF Lep 09980	PHLAW183-13	KM572969	BOLD:AAA6293	Austria	inatura, Dornbirn
Cnephasia asseclana	MM06677	LEFID650-10	HM873412	BOLD:AAA6293	Finland	University of Oulu
Cnephasia asseclana	TLMF Lep 08252	PHLAH433-12	KM572456	BOLD:AAA6293	Austria	inatura, Dornbirn
Cnephasia asseclana	MM12315	LEFIF565-10	HM875250	BOLD:AAA6293	Finland	University of Oulu
Cnephasia asseclana	TLMF Lep 07985	PHLAV166-12	KM572632	BOLD:AAA6293	Austria	inatura, Dornbirn
Cnephasia asseclana	MM09559	LEFIE627-10	HM874350	BOLD:AAA6293	Finland	University of Oulu
Cnephasia asseclana	MM09743	LEFIA866-10	HM387004	BOLD:AAA6293	Finland	University of Oulu
Cnephasia asseclana	MM13863	LEFIA935-10	HM387069	BOLD:AAA6293	Finland	University of Oulu
Cnephasia asseclana	MM05257	LEFIC916-10	HM872733	BOLD:AAA6293	Finland	University of Oulu
Cnephasia asseclana	MM05256	LEFIC915-10	HM872732	BOLD:AAA6293	Finland	University of Oulu
Cnephasia asseclana	MM14449	LEFIG421-10	HM876097	BOLD:AAA6293	Finland	University of Oulu
Cnephasia asseclana	MM21120	LEFIJ1260-11	KM572928	BOLD:AAA6293	Finland	University of Oulu
Cnephasia asseclana	MM03463	LEFIC126-10	HM871973	BOLD:AAA6293	Finland	University of Oulu
Cnephasia stephensiana	MM17357	LEFIJ732-10	KM572937	BOLD:AAA6292	Finland	University of Oulu
Cnephasia stephensiana	MM13183	LEFIF838-10	HM875521	BOLD:AAA6292	Finland	University of Oulu
Cnephasia stephensiana	MM13182	LEFIF837-10	HM875520	BOLD:AAA6292	Finland	University of Oulu

Cnephasia stephensiana	MM02129	LEFIB525-10	HM871413	BOLD:AAA6292	Finland	University of Oulu
Cnephasia stephensiana	MM17356	LEFIJ731-10	KM573543	BOLD:AAA6292	Finland	University of Oulu
Cnephasia stephensiana	MM05255	LEFIC914-10	HM872731	BOLD:AAA6292	Finland	University of Oulu
Cnephasia stephensiana	MM11848	LEFIF426-10	HM875111	BOLD:AAA6292	Finland	University of Oulu
Cnephasia stephensiana	MM21108	LEFIJ1248-11	KM573292	BOLD:AAA6292	Finland	University of Oulu
Cnephasia stephensiana	MM05680	LEFID071-10	HM872883	BOLD:AAA6292	Finland	University of Oulu
Cnephasia stephensiana	MM02128	LEFIB524-10	HM871412	BOLD:AAA6292	Finland	University of Oulu
Cnephasia stephensiana	TLMF Lep 08016	PHLAV197-12	KM572068	BOLD:AAA6292	Austria	inatura, Dornbirn
Cnephasia stephensiana	MM04998	LEFIC817-10	HM872636	BOLD:AAA6292	Finland	University of Oulu
Cnephasia stephensiana	MM03468	LEFIC129-10	HM871976	BOLD:AAA6292	Finland	University of Oulu
Cnephasia stephensiana	MM05203	LEFIC895-10	HM872712	BOLD:AAA6292	Finland	University of Oulu
Cnephasia stephensiana	MM08981	LEFIE408-10	HM874132	BOLD:AAA6292	Finland	University of Oulu
Cnephasia stephensiana	TLMF Lep 08741	PHLAH937-12	KM572647	BOLD:AAA6292	Austria	Tiroler Landesmuseum Ferdinandeum
Cnephasia stephensiana	MM03467	LEFIC128-10	HM871975	BOLD:AAA6292	Finland	University of Oulu
Cochylidia subroseana	MM02558	LEFIB731-10	HM871609	BOLD:AAD2823	Finland	University of Oulu
Cochylidia subroseana	MM14118	LEFIG223-10	HM875903	BOLD:AAD2823	Finland	University of Oulu
Cochylidia subroseana	MM02991	LEFIB896-10	HM871773	BOLD:AAD2823	Finland	University of Oulu
Cochylidia subroseana	TLMF Lep 09187	PHLAI625-13	KM573285	BOLD:AAD2823	Austria	Tiroler Landesmuseum Ferdinandeum
Cochylidia subroseana	TLMF Lep 00869	PHLAB069-10	HQ968243	BOLD:AAD2823	Austria	Tiroler Landesmuseum Ferdinandeum
Cochylis dubitana	MM03037	LEFIB915-10	HM871792	BOLD:AAC3780	Finland	University of Oulu
Cochylis dubitana	TLMF Lep 09169	PHLAI607-13	KM573614	BOLD:AAC3780	Austria	Tiroler Landesmuseum Ferdinandeum
Cochylis dubitana	MM04172	LEFIA818-10	HM386959	BOLD:AAC3780	Finland	University of Oulu
Cochylis dubitana	MM13286	LEFIF883-10	HM875565	BOLD:AAC3780	Finland	University of Oulu
Cochylis dubitana	MM10346	LEFIE949-10	HM874666	BOLD:AAC3780	Finland	University of Oulu
Cochylis dubitana	MM11845	LEFIF424-10	HM875109	BOLD:AAC3780	Finland	University of Oulu
Cochylis dubitana	MM06126	LEFID261-10	HM873059	BOLD:AAC3780	Finland	University of Oulu
Cochylis dubitana	MM04171	LEFIA817-10	HM386958	BOLD:AAC3780	Finland	University of Oulu
Cochylis dubitana	MM02551	LEFIB727-10	HM871605	BOLD:AAC3780	Finland	University of Oulu
Cochylis flaviciliana	MM06608	LEFID597-10	HM873362	BOLD:AAE8888	Finland	University of Oulu
Cochylis flaviciliana	MM06758	LEFID704-10	HM873465	BOLD:AAE8888	Finland	University of Oulu
Cochylis flaviciliana	MM06891	LEFID805-10	HM873562	BOLD:AAE8888	Finland	University of Oulu

Cochylis flaviciliana	TLMF Lep 07701	PHLAH262-12	KM572729	BOLD:ABA2672	Austria	Tiroler Landesmuseum Ferdinandeum
Cochylis nana	TLMF Lep 09954	PHLAW157-13	KM572211	BOLD:ABY5998	Austria	inatura, Dornbirn
Cochylis nana	MM03201	LEFIC001-10	HM871871	BOLD:ABY5998	Finland	University of Oulu
Cochylis nana	MM14110	LEFIG218-10	HM875898	BOLD:ABY5998	Finland	University of Oulu
Cochylis nana	MM05291	LEFIC934-10	HM872749	BOLD:ABY5998	Finland	University of Oulu
Cochylis pallidana	MM17588	LEFIK013-10	KM573429	BOLD:AAE1297	Finland	University of Oulu
Cochylis pallidana	TLMF Lep 07482	PHLAG803-12	KM572814	BOLD:AAE1297	Austria	inatura, Dornbirn
Cochylis pallidana	MM06762	LEFID708-10	HM873469	BOLD:AAE1297	Finland	University of Oulu
Cochylis pallidana	MM13289	LEFIF884-10	HM875566	BOLD:AAE1297	Finland	University of Oulu
Cochylis pallidana	MM02553	LEFIB728-10	HM871606	BOLD:AAE1297	Finland	University of Oulu
Cochylis pallidana	TLMF Lep 09155	PHLAI593-13	KM572462	BOLD:AAE1297	Austria	Tiroler Landesmuseum Ferdinandeum
Cochylis pallidana	MM06248	LEFID350-10	HM873147	BOLD:AAE1297	Finland	University of Oulu
Cochylis pallidana	TLMF Lep 08428	PHLAH609-12	KM573664	BOLD:AAE1297	Austria	inatura, Dornbirn
Cochylis pallidana	MM06249	LEFID351-10	HM873148	BOLD:AAL6146	Finland	University of Oulu
Coenonympha glycerion	TLMF Lep 08289	PHLAH470-12	KM573397	BOLD:AAA9402	Austria	inatura, Dornbirn
Coenonympha glycerion	MM06198	LEFID313-10	HM873110	BOLD:AAA9402	Finland	University of Oulu
Coenonympha glycerion	MM10081	LEFIE850-10	HM874569	BOLD:AAA9402	Finland	University of Oulu
Coenonympha glycerion	MM14398	LEFIG385-10	HM876062	BOLD:AAA9402	Finland	University of Oulu
Coenonympha pamphilus	TLMF Lep 09815	PHLAW018-13	KM572834	BOLD:AAA7351	Austria	Tiroler Landesmuseum Ferdinandeum
Coenonympha pamphilus	MM14354	LEFIG356-10	HM876033	BOLD:AAA7351	Finland	University of Oulu
Coenonympha pamphilus	MM17183	LEFIJ558-10	JF853673	BOLD:AAA7351	Finland	University of Oulu
Coenonympha pamphilus	MM10171	LEFIE892-10	HM874610	BOLD:AAA7351	Finland	University of Oulu
Coenonympha tullia	MM14772	LEFIG583-10	HM876254	BOLD:AAA3563	Finland	University of Oulu
Coenonympha tullia	MM06510	LEFID529-10	HM873294	BOLD:AAA3563	Finland	University of Oulu
Coenonympha tullia	MM03232	LEFIC021-10	HM871891	BOLD:AAA3563	Finland	University of Oulu
Coenonympha tullia	TLMF Lep 10011	LEATA404-13	KM573064	BOLD:AAA3563	Austria	inatura, Dornbirn
Coleophora ahenella	MM14850	LEFIG629-10	HM876295	BOLD:AAD0941	Finland	University of Oulu
Coleophora ahenella	MM02506	LEFIB703-10	HM871581	BOLD:AAD0941	Finland	University of Oulu
Coleophora ahenella	TLMF Lep 09969	PHLAW172-13	KM573436	BOLD:AAD0941	Austria	inatura, Dornbirn
Coleophora ahenella	MM14849	LEFIG628-10	HM876294	BOLD:AAD0941	Finland	University of Oulu
Coleophora albidella	MM06529	LEFID543-10	HM873308	BOLD:AAB8489	Finland	University of Oulu

Coleophora albidella	MM06528	LEFID542-10	HM873307	BOLD:AAB8489	Finland	University of Oulu
Coleophora albidella	MM02496	LEFIB696-10	HM871574	BOLD:AAB8489	Finland	University of Oulu
Coleophora albidella	MM06702	LEFID665-10	HM873427	BOLD:AAB8489	Finland	University of Oulu
Coleophora albidella	MM06765	LEFID711-10	HM873472	BOLD:AAB8489	Finland	University of Oulu
Coleophora albidella	TLMF Lep 10038	LEATA431-13	KM572744	BOLD:AAB8489	Austria	inatura, Dornbirn
Coleophora albidella	MM12409	LEFIF585-10	HM875269	BOLD:AAB8489	Finland	University of Oulu
Coleophora albidella	MM17555	LEFIJ930-10	KM572151	BOLD:AAB8489	Finland	University of Oulu
Coleophora albidella	MM12436	LEFIF590-10	HM875274	BOLD:AAB8489	Finland	University of Oulu
Coleophora alticolella	MM06483	LEFID516-10	HM873281	BOLD:AAC6419	Finland	University of Oulu
Coleophora alticolella	TLMF Lep 09209	PHLAI647-13	KM572457	BOLD:AAC6419	Austria	Tiroler Landesmuseum Ferdinandeum
Coleophora alticolella	MM14166	LEFIG246-10	HM875925	BOLD:AAC6419	Finland	University of Oulu
Coleophora alticolella	TLMF Lep 04305	PHLAE085-11	JN265776	BOLD:AAC6419	Austria	Tiroler Landesmuseum Ferdinandeum
Coleophora alticolella	MM09908	LEFIE764-10	HM874483	BOLD:AAC6419	Finland	University of Oulu
Coleophora alticolella	MM09910	LEFIE766-10	HM874485	BOLD:AAC6419	Finland	University of Oulu
Coleophora alticolella	TLMF Lep 02845	PHLAC810-10	JF860327	BOLD:AAC6419	Austria	Tiroler Landesmuseum Ferdinandeum
Coleophora alticolella	MM03818	LEFIC316-10	HM872160	BOLD:AAC6419	Finland	University of Oulu
Coleophora alticolella	MM06554	LEFID561-10	HM873326	BOLD:AAC6419	Finland	University of Oulu
Coleophora alticolella	MM02463	LEFIB674-10	HM871552	BOLD:AAC6419	Finland	University of Oulu
Coleophora alticolella	TLMF Lep 09213	PHLAI651-13	KM572233	BOLD:AAC6419	Austria	Tiroler Landesmuseum Ferdinandeum
Coleophora alticolella	MM06383	LEFID448-10	HQ570344	BOLD:AAC6419	Finland	University of Oulu
Coleophora alticolella	MM06242	LEFID346-10	HM873143	BOLD:AAC6419	Finland	University of Oulu
Coleophora alticolella	TLMF Lep 09976	PHLAW179-13	KM573236	BOLD:AAC6419	Austria	inatura, Dornbirn
Coleophora betulella	TLMF Lep 07529	PHLAG850-12	KM573107	BOLD:AAE8825	Austria	Tiroler Landesmuseum Ferdinandeum
Coleophora betulella	MM06617	LEFID604-10	HM873369	BOLD:AAE8825	Finland	University of Oulu
Coleophora betulella	MM09797	LEFIA919-10	HM387055	BOLD:AAE8825	Finland	University of Oulu
Coleophora betulella	MM10145	LEFIE874-10	HM874592	BOLD:AAE8825	Finland	University of Oulu
Coleophora betulella	MM18171	LEFIK596-10	JF854178	BOLD:AAE8825	Finland	University of Oulu
Coleophora caespititiella	MM06232	LEFID338-10	HM873135	BOLD:AAE8821	Finland	University of Oulu
Coleophora caespititiella	MM06220	LEFID327-10	HM873124	BOLD:AAE8821	Finland	University of Oulu
Coleophora caespititiella	TLMF Lep 09965	PHLAW168-13	KM572276	BOLD:AAE8821	Austria	inatura, Dornbirn
Coleophora caespititiella	MM02472	LEFIB677-10	HM871555	BOLD:AAE8821	Finland	University of Oulu

Coleophora caespititiella	MM19895	LEFII245-11	KM572034	BOLD:AAE8821	Finland	Research Collection of E. Laasonen
Coleophora caespititiella	MM02473	LEFIB678-10	HM871556	BOLD:AAE8821	Finland	University of Oulu
Coleophora caespititiella	MM02471	LEFIB676-10	HM871554	BOLD:AAE8821	Finland	University of Oulu
Coleophora caespititiella	MM06481	LEFID514-10	HM873279	BOLD:AAE8821	Finland	University of Oulu
Coleophora caespititiella	MM06477	LEFID510-10	HM873275	BOLD:AAE8821	Finland	University of Oulu
Coleophora flavipennella	MM09703	LEFIE708-10	HM874429	BOLD:AAC3993	Finland	University of Oulu
Coleophora flavipennella	MM06704	LEFID666-10	HM873428	BOLD:AAC3993	Finland	University of Oulu
Coleophora flavipennella	MM06759	LEFID705-10	HM873466	BOLD:AAC3993	Finland	University of Oulu
Coleophora flavipennella	MM09776	LEFIE711-10	HM874431	BOLD:AAC3993	Finland	University of Oulu
Coleophora flavipennella	TLMF Lep 08238	PHLAH419-12	KM571947	BOLD:AAC3993	Austria	inatura, Dornbirn
Coleophora flavipennella	TLMF Lep 08416	PHLAH597-12	KM573548	BOLD:AAC3993	Austria	inatura, Dornbirn
Coleophora flavipennella	MM09808	LEFIE718-10	HM874438	BOLD:AAC3993	Finland	University of Oulu
Coleophora flavipennella	TLMF Lep 08257	PHLAH438-12	KM573590	BOLD:AAC3993	Austria	inatura, Dornbirn
Coleophora flavipennella	TLMF Lep 08256	PHLAH437-12	KM572956	BOLD:AAC3993	Austria	inatura, Dornbirn
Coleophora fuscocuprella	MM15575	LEFIG711-10	HM876367	BOLD:AAF3835	Finland	University of Oulu
Coleophora fuscocuprella	MM18995	LEFIL697-10	KM572715	BOLD:AAF3835	Finland	University of Oulu
Coleophora fuscocuprella	TLMF Lep 09975	PHLAW178-13	KM573444	BOLD:AAF3835	Austria	inatura, Dornbirn
Coleophora gryphipennella	MM09601	LEFIE645-10	HM874368	BOLD:AAE8826	Finland	University of Oulu
Coleophora gryphipennella	MM03932	LEFIC392-10	HM872235	BOLD:AAE8826	Finland	University of Oulu
Coleophora gryphipennella	MM09636	LEFIE671-10	HM874393	BOLD:AAE8826	Finland	University of Oulu
Coleophora gryphipennella	TLMF Lep 09203	PHLAI641-13	KM573253	BOLD:AAE8826	Austria	Tiroler Landesmuseum Ferdinandeum
Coleophora gryphipennella	MM13678	LEFIG057-10	HM875736	BOLD:AAE8826	Finland	University of Oulu
Coleophora kuehnella	MM17557	LEFIJ932-10	JF853903	BOLD:AAC6417	Finland	University of Oulu
Coleophora kuehnella	TLMF Lep 08049	PHLAV230-12	KM572139	BOLD:AAC6417	Austria	inatura, Dornbirn
Coleophora kuehnella	MM18967	LEFIL669-10	KM573538	BOLD:AAC6417	Finland	University of Oulu
Coleophora kuehnella	MM18968	LEFIL670-10	KM573270	BOLD:AAC6417	Finland	University of Oulu

Coleophora lusciniaepennella	MM23101	LEFIJ1909-13	KM572326	BOLD:AAY9622	Finland	University of Oulu
Coleophora lusciniaepennella	MM23104	LEFIJ1912-13	KM573251	BOLD:AAY9622	Finland	University of Oulu
Coleophora lusciniaepennella	MM08687	LEFIE315-10	HM874041	BOLD:AAY9622	Finland	University of Oulu
Coleophora lusciniaepennella	MM13701	LEFIH364-10	HM876980	BOLD:AAY9622	Finland	University of Oulu
Coleophora lusciniaepennella	MM23096	LEFIJ1904-13	KM572036	BOLD:AAY9622	Finland	University of Oulu
Coleophora lusciniaepennella	MM09305	LEFIE497-10	HM874221	BOLD:AAY9622	Finland	University of Oulu
Coleophora lusciniaepennella	MM23105	LEFIJ1913-13	KM573539	BOLD:AAY9622	Finland	University of Oulu
Coleophora lusciniaepennella	MM23097	LEFIJ1905-13	KM573254	BOLD:AAY9622	Finland	University of Oulu
Coleophora lusciniaepennella	MM00961	LEFIA055-10	HM396403	BOLD:AAY9622	Finland	University of Oulu
Coleophora lusciniaepennella	MM02494	LEFIB694-10	HM871572	BOLD:AAY9622	Finland	University of Oulu
Coleophora lusciniaepennella	MM23103	LEFIJ1911-13	KM571952	BOLD:AAY9622	Finland	University of Oulu
Coleophora lusciniaepennella	MM23099	LEFIJ1907-13	KM572220	BOLD:AAY9622	Finland	University of Oulu
Coleophora lusciniaepennella	MM23100	LEFIJ1908-13	KM573697	BOLD:AAY9622	Finland	University of Oulu
Coleophora lusciniaepennella	MM15982	LEFIJ378-10	JF853565	BOLD:AAY9622	Finland	University of Oulu
Coleophora lusciniaepennella	TLMF Lep 10044	LEATA437-13	KM572734	BOLD:AAY9622	Austria	inatura, Dornbirn

Coleophora lusciniaepennella	MM23098	LEFIJ1906-13	KM572654	BOLD:AAY9622	Finland	University of Oulu
Coleophora lusciniaepennella	MM15983	LEFIJ379-10	JF853566	BOLD:AAY9622	Finland	University of Oulu
Coleophora mayrella	MM14468	LEFIG432-10	HM876108	BOLD:AAB3182	Finland	University of Oulu
Coleophora mayrella	MM02532	LEFIB717-10	HM871595	BOLD:AAB3182	Finland	University of Oulu
Coleophora mayrella	TLMF Lep 08059	PHLAV240-12	KM572914	BOLD:AAB3182	Austria	inatura, Dornbirn
Coleophora mayrella	MM03169	LEFIB976-10	HM871849	BOLD:AAB3182	Finland	University of Oulu
Coleophora mayrella	TLMF Lep 08057	PHLAV238-12	KM573209	BOLD:AAB3182	Austria	inatura, Dornbirn
Coleophora milvipennis	MM08586	LEFIE253-10	HM873987	BOLD:AAB7467	Finland	University of Oulu
Coleophora milvipennis	MM06475	LEFID508-10	HM873273	BOLD:AAB7467	Finland	University of Oulu
Coleophora milvipennis	MM14058	LEFIG189-10	HM875869	BOLD:AAB7467	Finland	University of Oulu
Coleophora milvipennis	MM17883	LEFIK308-10	JF853961	BOLD:AAB7467	Finland	University of Oulu
Coleophora milvipennis	TLMF Lep 09963	PHLAW166-13	KM573520	BOLD:AAB7467	Austria	inatura, Dornbirn
Coleophora milvipennis	MM02475	LEFIB679-10	HM871557	BOLD:AAB7467	Finland	University of Oulu
Coleophora milvipennis	MM06624	LEFID610-10	HM873375	BOLD:AAB7467	Finland	University of Oulu
Coleophora orbitella	MM21136	LEFIJ1276-11	KM573267	BOLD:AAF4235	Finland	University of Oulu
Coleophora orbitella	MM09433	LEFIE548-10	HM874271	BOLD:AAF4235	Finland	University of Oulu
Coleophora orbitella	MM09350	LEFIE518-10	HM874241	BOLD:AAF4235	Finland	University of Oulu
Coleophora orbitella	MM09315	LEFIE500-10	HM874224	BOLD:AAF4235	Finland	University of Oulu
Coleophora orbitella	MM09641	LEFIE674-10	HM874396	BOLD:AAF4235	Finland	University of Oulu
Coleophora orbitella	TLMF Lep 09953	PHLAW156-13	KM572246	BOLD:AAF4235	Austria	inatura, Dornbirn
Coleophora serratella	MM06761	LEFID707-10	HM873468	BOLD:AAB5543	Finland	University of Oulu
Coleophora serratella	MM00068	LEFIA1055-10	GU828595	BOLD:AAB5543	Finland	University of Oulu
Coleophora serratella	MM09810	LEFIE719-10	HM874439	BOLD:AAB5543	Finland	University of Oulu
Coleophora serratella	MM09707	LEFIA832-10	HM386972	BOLD:AAB5543	Finland	University of Oulu
Coleophora serratella	MM00067	LEFIA1054-10	GU828594	BOLD:AAB5543	Finland	University of Oulu
Coleophora serratella	MM03484	LEFIC140-10	HM871986	BOLD:AAB5543	Finland	University of Oulu
Coleophora serratella	MM02502	LEFIB699-10	HM871577	BOLD:AAB5543	Finland	University of Oulu
Coleophora serratella	MM09708	LEFIA833-10	HM386973	BOLD:AAB5543	Finland	University of Oulu
Coleophora serratella	MM06827	LEFID762-10	HM873519	BOLD:AAB5543	Finland	University of Oulu

Coleophora serratella	MM09574	LEFIE632-10	HM874355	BOLD:AAB5543	Finland	University of Oulu
Coleophora serratella	TLMF Lep 09968	PHLAW171-13	KM572805	BOLD:AAB5543	Austria	inatura, Dornbirn
Coleophora serratella	MM00700	LEFIB238-10	HM871141	BOLD:AAB5543	Finland	University of Oulu
Coleophora serratella	MM09799	LEFIE713-10	HM874433	BOLD:AAB5543	Finland	University of Oulu
Coleophora serratella	MM06726	LEFID682-10	HM873443	BOLD:AAB5543	Finland	University of Oulu
Coleophora sternipennella	TLMF Lep 08467	PHLAH648-12	KM572466	BOLD:AAD3945	Austria	inatura, Dornbirn
Coleophora sternipennella	MM13680	LEFIG059-10	HM875738	BOLD:AAE9649	Finland	University of Oulu
Coleophora sternipennella	MM03176	LEFIB983-10	HM871855	BOLD:AAE9649	Finland	University of Oulu
Coleophora sternipennella	MM02509	LEFIB706-10	HM871584	BOLD:AAE9649	Finland	University of Oulu
Coleophora sternipennella	MM02504	LEFIB701-10	HM871579	BOLD:AAE9649	Finland	University of Oulu
Coleophora sternipennella	MM16717	ELACA491-10	KF808900	BOLD:AAE9649	Finland	University of Oulu
Coleophora sternipennella	MM03235	LEFIC023-10	HM871893	BOLD:AAE9649	Finland	University of Oulu
Coleophora sternipennella	MM10059	LEFIE840-10	HM874559	BOLD:AAE9649	Finland	University of Oulu
Coleophora sternipennella	TLMF Lep 08462	PHLAH643-12	KM572022	BOLD:AAE9649	Austria	inatura, Dornbirn
Coleophora sternipennella	MM06829	LEFID764-10	HM873521	BOLD:AAE9649	Finland	University of Oulu
Coleophora sternipennella	MM09519	LEFIE605-10	HM874328	BOLD:AAE9649	Finland	University of Oulu
Coleophora sternipennella	MM09520	LEFIE606-10	HM874329	BOLD:AAE9649	Finland	University of Oulu
Coleophora sternipennella	MM16715	ELACA489-10	KF808622	BOLD:AAE9649	Finland	University of Oulu
Coleophora taeniipennella	MM14244	LEFIG291-10	HM875970	BOLD:AAC8749	Finland	University of Oulu
Coleophora taeniipennella	MM02476	LEFIB680-10	HM871558	BOLD:AAC8749	Finland	University of Oulu
Coleophora taeniipennella	MM02500	LEFIB698-10	HM871576	BOLD:AAC8749	Finland	University of Oulu
Coleophora taeniipennella	MM14245	LEFIG292-10	HM875971	BOLD:AAC8749	Finland	University of Oulu
Coleophora taeniipennella	MM09831	LEFIE732-10	HM874452	BOLD:AAC8749	Finland	University of Oulu
Coleophora taeniipennella	TLMF Lep 07528	PHLAG849-12	KM573278	BOLD:AAC8749	Austria	Tiroler Landesmuseum Ferdinandeum
Coleophora taeniipennella	MM09909	LEFIE765-10	HM874484	BOLD:AAC8749	Finland	University of Oulu
Coleophora taeniipennella	MM12040	LEFIF488-10	HM875173	BOLD:AAC8749	Finland	University of Oulu
Coleophora trigeminella	TLMF Lep 08065	PHLAV246-12	KM573153	BOLD:AAD9304	Austria	inatura, Dornbirn
Coleophora trigeminella	MM17682	LEFIK107-10	JN265335	BOLD:AAD9304	Finland	University of Oulu
Coleophora trigeminella	MM10436	LEFIE998-10	HM874713	BOLD:AAD9304	Finland	University of Oulu
Coleophora uliginosella	MM21105	LEFIJ1245-11	KM573128	BOLD:AAE9656	Finland	University of Oulu
Coleophora uliginosella	MM20540	LEEUA481-11	KM572046	BOLD:AAE9656	Finland	University of Oulu

Coleophora uliginosella	MM15981	LEFIJ377-10	JF853564	BOLD:AAE9656	Finland	University of Oulu
Coleophora uliginosella	TLMF Lep 02846	PHLAC811-10	JF860328	BOLD:AAE9656	Austria	Tiroler Landesmuseum Ferdinandeum
Coleophora virgaureae	MM04169	LEFIA815-10	HM386956	BOLD:AAB3183	Finland	University of Oulu
Coleophora virgaureae	MM03172	LEFIB979-10	HM871852	BOLD:AAB3183	Finland	University of Oulu
Coleophora virgaureae	MM03173	LEFIB980-10	HM871853	BOLD:AAB3183	Finland	University of Oulu
Coleophora virgaureae	MM03174	LEFIB981-10	HQ570281	BOLD:AAB3183	Finland	University of Oulu
Coleophora virgaureae	MM10164	LEFIE886-10	HM874604	BOLD:AAB3183	Finland	University of Oulu
Coleophora virgaureae	TLMF Lep 09188	PHLAI626-13	KM573273	BOLD:AAB3183	Austria	Tiroler Landesmuseum Ferdinandeum
Coleophora virgaureae	MM04170	LEFIA816-10	HM386957	BOLD:AAB3183	Finland	University of Oulu
Coleophora virgaureae	MM03175	LEFIB982-10	HM871854	BOLD:AAB3183	Finland	University of Oulu
Coleophora virgaureae	MM18194	LEFIK619-10	JF854194	BOLD:AAB3183	Finland	University of Oulu
Coleophora virgaureae	MM10165	LEFIE887-10	HM874605	BOLD:AAB3183	Finland	University of Oulu
Coleophora virgaureae	MM02512	LEFIB708-10	HM871586	BOLD:AAB3183	Finland	University of Oulu
Coleophora virgaureae	MM17961	LEFIK386-10	JF854018	BOLD:AAB3183	Finland	University of Oulu
Coleophora virgaureae	MM09827	LEFIE730-10	HM874450	BOLD:AAB3183	Finland	University of Oulu
Coleophora virgaureae	MM02493	LEFIB693-10	HM871571	BOLD:AAB3183	Finland	University of Oulu
Coleophora virgaureae	MM02492	LEFIB692-10	HM871570	BOLD:AAB3183	Finland	University of Oulu
Colias hyale	TLMF Lep 09811	PHLAW014-13	KM572430	BOLD:AAA5531	Austria	Tiroler Landesmuseum Ferdinandeum
Colias hyale	MM17133	LEFIJ508-10	JF853631	BOLD:AAA5531	Finland	University of Oulu
Colias hyale	MM06097	LEFID236-10	HM873036	BOLD:AAA5531	Finland	University of Oulu
Colias palaeno	TLMF Lep 06181	PHLSA726-11	KM572538	BOLD:AAA3447	Austria	Tiroler Landesmuseum Ferdinandeum
Colias palaeno	MM17134	LEFIJ509-10	JF853632	BOLD:AAA3447	Finland	University of Oulu
Colias palaeno	MM03233	LEFIA1057-10	GU828690	BOLD:AAA3447	Finland	University of Oulu
Colias palaeno	MM03411	LEFIC097-10	HQ570288	BOLD:AAA3447	Finland	University of Oulu
Colias palaeno	MM17135	LEFIJ510-10	KM572382	BOLD:AAA3447	Finland	University of Oulu
Colocasia coryli	MM08417	LEFIE163-10	HM873909	BOLD:AAC2611	Finland	University of Oulu
Colocasia coryli	MM01524	LEFIA459-10	HM386799	BOLD:AAC2611	Finland	University of Oulu
Colocasia coryli	MM01523	LEFIA458-10	HM386798	BOLD:AAC2611	Finland	University of Oulu
Colocasia coryli	TLMF Lep 04638	PHLAE323-11	JN275669	BOLD:AAC2611	Austria	Tiroler Landesmuseum Ferdinandeum
Colostygia aptata	MM01371	LEFIA324-10	HM386667	BOLD:AAB9512	Finland	University of Oulu
Colostygia aptata	MM04931	LEFIC792-10	HM872611	BOLD:AAB9512	Finland	University of Oulu

Colostygia aptata	MM04930	LEFIC791-10	HM872610	BOLD:AAB9512	Finland	University of Oulu
Colostygia aptata	MM01400	LEFIA353-10	HM386696	BOLD:AAB9512	Finland	University of Oulu
Colostygia aptata	TLMF Lep 06201	PHLSA746-11	KM572561	BOLD:AAB9512	Austria	Tiroler Landesmuseum Ferdinandeum
Colostygia aptata	TLMF Lep 08497	PHLAH678-12	KM573669	BOLD:AAB9512	Austria	inatura, Dornbirn
Colostygia aptata	MM01370	LEFIA323-10	HM386666	BOLD:AAB9512	Finland	University of Oulu
Colostygia olivata	TLMF Lep 08797	PHLAI302-13	KM571965	BOLD:AAB9509	Austria	Tiroler Landesmuseum Ferdinandeum
Colostygia olivata	MM09833	LEFIE734-10	HM874454	BOLD:AAB9511	Finland	University of Oulu
Colostygia olivata	MM09552	LEFIE623-10	HM874346	BOLD:AAB9511	Finland	University of Oulu
Colostygia olivata	MM18441	LEFIK866-10	JF854326	BOLD:AAB9511	Finland	University of Oulu
Colostygia pectinataria	MM02922	LEFIB867-10	HM871744	BOLD:AAB4885	Finland	University of Oulu
Colostygia pectinataria	MM01388	LEFIA341-10	HM386684	BOLD:AAB4885	Finland	University of Oulu
Colostygia pectinataria	MM01387	LEFIA340-10	HM386683	BOLD:AAB4885	Finland	University of Oulu
Colostygia pectinataria	TLMF Lep 07977	PHLAV158-12	KM572932	BOLD:AAB4885	Austria	inatura, Dornbirn
Colostygia turbata	MM15814	LEFIG950-10	HM876590	BOLD:AAC0784	Finland	University of Oulu
Colostygia turbata	MM18442	LEFIK867-10	JF854327	BOLD:AAC0784	Finland	University of Oulu
Colostygia turbata	MM10469	LEFIF030-10	HM874741	BOLD:AAC0784	Finland	University of Oulu
Colostygia turbata	MM18443	LEFIK868-10	JF854328	BOLD:AAC0784	Finland	University of Oulu
Colostygia turbata	TLMF Lep 07562	PHLAG883-12	KM573487	BOLD:AAC0785	Austria	inatura, Dornbirn
Colotois pennaria	TLMF Lep 06122	PHLSA667-11	KM573504	BOLD:AAB0886	Austria	Tiroler Landesmuseum Ferdinandeum
Colotois pennaria	MM02693	LEFIB787-10	HM871664	BOLD:AAB0887	Finland	University of Oulu
Colotois pennaria	MM12218	LEFIF539-10	HM875224	BOLD:AAB0887	Finland	University of Oulu
Colotois pennaria	MM18507	LEFIK932-10	JF854374	BOLD:AAB0887	Finland	University of Oulu
Conistra rubiginea	TLMF Lep 08810	PHLAI315-13	KM573574	BOLD:AAD3682	Austria	Tiroler Landesmuseum Ferdinandeum
Conistra rubiginea	MM12191	LEFIF534-10	HM875219	BOLD:AAD3682	Finland	University of Oulu
Conistra rubiginea	MM04625	LEFIC665-10	HM872486	BOLD:ACE5168	Finland	University of Oulu
Conistra rubiginea	MM10790	LEFIF193-10	HM874887	BOLD:ACE5168	Finland	University of Oulu
Conistra rubiginosa	MM14439	LEFIG413-10	HM876089	BOLD:AAF0863	Finland	University of Oulu
Conistra rubiginosa	MM18039	LEFIK464-10	JF854078	BOLD:AAF0863	Finland	University of Oulu
Conistra rubiginosa	MM14440	LEFIG414-10	HM876090	BOLD:AAF0863	Finland	University of Oulu
Conistra rubiginosa	TLMF Lep 08785	PHLAI290-13	KM572376	BOLD:AAF0863	Austria	Tiroler Landesmuseum Ferdinandeum
Conistra vaccinii	MM01580	LEFIA498-10	KM573231	BOLD:AAB7880	Finland	University of Oulu

Conistra vaccinii	TLMF Lep 06194	PHLSA739-11	KM573336	BOLD:AAB7880	Austria	Tiroler Landesmuseum Ferdinandeum
Conistra vaccinii	MM01581	LEFIA499-10	KM573677	BOLD:AAB7880	Finland	University of Oulu
Conistra vaccinii	MM04615	LEFIC658-10	HM872479	BOLD:AAB7880	Finland	University of Oulu
Conistra vaccinii	MM00416	LEFIB104-10	HM871011	BOLD:AAB7880	Finland	University of Oulu
Coptotriche angusticollella	MM00889	LEFIB342-10	HM871242	BOLD:AAF8244	Finland	University of Oulu
Coptotriche angusticollella	TLMF Lep 08483	PHLAH664-12	KM573290	BOLD:AAF8244	Austria	inatura, Dornbirn
Coptotriche angusticollella	MM00891	LEFIB344-10	HM871244	BOLD:AAF8244	Finland	University of Oulu
Coptotriche angusticollella	MM00890	LEFIB343-10	HM871243	BOLD:AAF8244	Finland	University of Oulu
Coptotriche marginea	MM09666	LEFIE690-10	HM874411	BOLD:AAF5585	Finland	University of Oulu
Coptotriche marginea	MM21091	LEFIJ1231-11	KM572658	BOLD:AAF5585	Finland	University of Oulu
Coptotriche marginea	MM08720	LEFIE331-10	HM874057	BOLD:AAF5585	Finland	University of Oulu
Coptotriche marginea	MM09181	LEFIE434-10	HM874158	BOLD:AAF5585	Finland	University of Oulu
Coptotriche marginea	TLMF Lep 08424	PHLAH605-12	KM573169	BOLD:AAF5585	Austria	inatura, Dornbirn
Cosmia affinis	TLMF Lep 08555	PHLAH736-12	KM572576	BOLD:AAE9788	Austria	inatura, Dornbirn
Cosmia affinis	MM09284	LEFIJ1013-11	KM572331	BOLD:AAE9788	Finland	University of Oulu
Cosmia affinis	MM15945	LEFIJ345-10	KM572070	BOLD:AAE9788	Finland	University of Oulu
Cosmia pyralina	MM18727	LEFIL429-10	KM572941	BOLD:AAE9789	Finland	University of Oulu
Cosmia pyralina	MM15946	LEFIJ346-10	KM573099	BOLD:AAE9789	Finland	University of Oulu
Cosmia pyralina	TLMF Lep 08179	PHLAV360-12	KM572949	BOLD:AAE9789	Austria	inatura, Dornbirn
Cosmia pyralina	MM21084	LEFIJ1224-11	KM572720	BOLD:AAE9789	Finland	University of Oulu
Cosmia pyralina	MM21165	LEFIJ1305-11	KM572535	BOLD:AAE9789	Finland	University of Oulu
Cosmia trapezina	MM01696	LEFIA586-10	HM870835	BOLD:AAB9038	Finland	University of Oulu
Cosmia trapezina	TLMF Lep 08162	PHLAV343-12	KM573676	BOLD:AAB9038	Austria	inatura, Dornbirn
Cosmia trapezina	MM01697	LEFIA587-10	HM870836	BOLD:AAB9038	Finland	University of Oulu
Cosmia trapezina	MM04883	LEFIC762-10	HM872581	BOLD:AAB9038	Finland	University of Oulu
Cosmopterix lienigiella	TLMF Lep 08439	PHLAH620-12	KM573551	BOLD:AAJ5475	Austria	inatura, Dornbirn
Cosmopterix lienigiella	MM15605	LEFIG741-10	HM876397	BOLD:AAJ5475	Finland	University of Oulu
Cosmopterix lienigiella	MM18628	LEFIL318-10	KM572367	BOLD:AAJ5475	Finland	University of Oulu
Cosmopterix orichalcea	MM06176	LEFID299-10	HM873096	BOLD:AAJ5454	Finland	University of Oulu
Cosmopterix orichalcea	MM15603	LEFIG739-10	HM876395	BOLD:AAJ5454	Finland	University of Oulu
Cosmopterix orichalcea	MM21066	LEFIJ1206-11	KM573572	BOLD:AAJ5454	Finland	University of Oulu

Cosmopterix orichalcea	TLMF Lep 08440	PHLAH621-12	KM573545	BOLD:AAJ5454	Austria	inatura, Dornbirn
Cosmopterix orichalcea	MM18207	LEFIK632-10	KM572113	BOLD:AAJ5454	Finland	University of Oulu
Cosmorhoe ocellata	MM11590	LEFIF370-10	HM875055	BOLD:AAB4250	Finland	University of Oulu
Cosmorhoe ocellata	TLMF Lep 07975	PHLAV156-12	KM573172	BOLD:AAB4250	Austria	inatura, Dornbirn
Cosmorhoe ocellata	MM01250	LEFIA219-10	HM396562	BOLD:AAB4250	Finland	University of Oulu
Cosmorhoe ocellata	MM01251	LEFIA220-10	HM396563	BOLD:AAB4250	Finland	University of Oulu
Cosmotriche lobulina	EA1.094	LOBU006-12	KM573564	BOLD:AAE4008	Finland	Research Collection of Hannu Saarenmaa
Cosmotriche lobulina	EA1.08P	LOBU041-12	KM572431	BOLD:AAE4008	Finland	Research Collection of Hannu Saarenmaa
Cosmotriche lobulina	EA1.08O	LOBU042-12	KM573036	BOLD:AAE4008	Finland	Research Collection of Hannu Saarenmaa
Cosmotriche lobulina	EA1.08N	LOBU043-12	KM572396	BOLD:AAE4008	Finland	Research Collection of Hannu Saarenmaa
Cosmotriche lobulina	EA1.08M	LOBU044-12	KM573403	BOLD:AAE4008	Finland	University of Eastern Finland
Cosmotriche lobulina	EA1.08K	LOBU046-12	KM573499	BOLD:AAE4008	Finland	University of Eastern Finland
Cosmotriche lobulina	EA1.08H	LOBU049-12	KM572531	BOLD:AAE4008	Finland	University of Eastern Finland
Cosmotriche lobulina	EA1.08G	LOBU050-12	KM572495	BOLD:AAE4008	Finland	University of Eastern Finland
Cosmotriche lobulina	EA1.08F	LOBU051-12	KM572721	BOLD:AAE4008	Finland	University of Eastern Finland
Cosmotriche lobulina	EA1.08D	LOBU053-12	KM572801	BOLD:AAE4008	Finland	University of Eastern Finland
Cosmotriche lobulina	EA1.08C	LOBU054-12	KM572136	BOLD:AAE4008	Finland	University of Eastern Finland
Cosmotriche lobulina	EA1.08B	LOBU055-12	KM572631	BOLD:AAE4008	Finland	University of Eastern Finland
Cosmotriche lobulina	EA1.08A	LOBU056-12	KM572549	BOLD:AAE4008	Finland	University of Eastern Finland
Cosmotriche lobulina	EA1.0ED	LOBU058-12	KM573473	BOLD:AAE4008	Finland	University of Helsinki
Cosmotriche lobulina	EA1.0EC	LOBU059-12	KM572910	BOLD:AAE4008	Finland	University of Helsinki
Cosmotriche lobulina	EA1.0E9	LOBU062-12	KM572392	BOLD:AAE4008	Finland	University of Helsinki
Cosmotriche lobulina	EA1.099	LOBU001-12	KM572623	BOLD:AAE4008	Finland	Research Collection of Hannu Saarenmaa
Cosmotriche lobulina	EA1.098	LOBU002-12	KM571985	BOLD:AAE4008	Finland	Research Collection of Hannu Saarenmaa

Cosmotriche lobulina	EA1.096	LOBU004-12	KM573072	BOLD:AAE4008	Finland	Research Collection of Hannu Saarenmaa
Cosmotriche lobulina	EA1.095	LOBU005-12	KM572685	BOLD:AAE4008	Finland	Research Collection of Hannu Saarenmaa
Cosmotriche lobulina	EA1.084	LOBU016-12	KM573201	BOLD:AAE4008	Finland	University of Eastern Finland
Cosmotriche lobulina	EA1.093	LOBU007-12	KM572346	BOLD:AAE4008	Finland	Research Collection of Hannu Saarenmaa
Cosmotriche lobulina	TLMF Lep 06110	PHLSA655-11	KM572607	BOLD:AAE4008	Austria	Tiroler Landesmuseum Ferdinandeum
Cosmotriche lobulina	EA1.092	LOBU008-12	KM572698	BOLD:AAE4008	Finland	Research Collection of Hannu Saarenmaa
Cosmotriche lobulina	EA1.091	LOBU009-12	KM573475	BOLD:AAE4008	Finland	Research Collection of Hannu Saarenmaa
Cosmotriche lobulina	EA1.090	LOBU010-12	KM573011	BOLD:AAE4008	Finland	Research Collection of Hannu Saarenmaa
Cosmotriche lobulina	EA1.09I	LOBU022-12	KM572931	BOLD:AAE4008	Finland	Research Collection of Hannu Saarenmaa
Cosmotriche lobulina	EA1.09H	LOBU023-12	KM571961	BOLD:AAE4008	Finland	Research Collection of Hannu Saarenmaa
Cosmotriche lobulina	TLMF Lep 03077	PHLAD092-11	JN307297	BOLD:AAE4008	Austria	Tiroler Landesmuseum Ferdinandeum
Cosmotriche lobulina	TLMF Lep 03076	PHLAD091-11	JN307296	BOLD:AAE4008	Austria	Tiroler Landesmuseum Ferdinandeum
Cosmotriche lobulina	EA1.09G	LOBU024-12	KM572499	BOLD:AAE4008	Finland	Research Collection of Hannu Saarenmaa
Cosmotriche lobulina	EA1.09F	LOBU025-12	KM572871	BOLD:AAE4008	Finland	Research Collection of Hannu Saarenmaa
Cosmotriche lobulina	EA1.09E	LOBU026-12	KM572724	BOLD:AAE4008	Finland	Research Collection of Hannu Saarenmaa
Cosmotriche lobulina	EA1.09D	LOBU027-12	KM573014	BOLD:AAE4008	Finland	Research Collection of Hannu Saarenmaa
Cosmotriche lobulina	EA1.09A	LOBU030-12	KM573208	BOLD:AAE4008	Finland	Research Collection of Hannu Saarenmaa

Cosmotriche lobulina	EA1.08Z	LOBU031-12	KM573456	BOLD:AAE4008	Finland	Research Collection of Hannu Saarenmaa
Cosmotriche lobulina	EA1.08Y	LOBU032-12	KM573098	BOLD:AAE4008	Finland	Research Collection of Hannu Saarenmaa
Cosmotriche lobulina	EA1.08X	LOBU033-12	KM572478	BOLD:AAE4008	Finland	Research Collection of Hannu Saarenmaa
Cosmotriche lobulina	EA1.08W	LOBU034-12	KM573696	BOLD:AAE4008	Finland	Research Collection of Hannu Saarenmaa
Cosmotriche lobulina	EA1.08V	LOBU035-12	KM572692	BOLD:AAE4008	Finland	Research Collection of Hannu Saarenmaa
Cosmotriche lobulina	EA1.08T	LOBU037-12	KM573119	BOLD:AAE4008	Finland	Research Collection of Hannu Saarenmaa
Cosmotriche lobulina	EA1.08R	LOBU039-12	KM573656	BOLD:AAE4008	Finland	Research Collection of Hannu Saarenmaa
Cosmotriche lobulina	EA1.08Q	LOBU040-12	KM573037	BOLD:AAE4008	Finland	Research Collection of Hannu Saarenmaa
Cosmotriche lobulina	EA1.0E8	LOBU063-12	KM573541	BOLD:AAE4008	Finland	Research Collection of Hannu Saarenmaa
Cosmotriche lobulina	EA1.0E7	LOBU064-12	KM573516	BOLD:AAE4008	Finland	Research Collection of Hannu Saarenmaa
Cosmotriche lobulina	EA1.0E6	LOBU065-12	KM573641	BOLD:AAE4008	Finland	Research Collection of Hannu Saarenmaa
Cosmotriche lobulina	EA1.0E5	LOBU066-12	KM572907	BOLD:AAE4008	Finland	Research Collection of Hannu Saarenmaa
Cosmotriche lobulina	EA1.0E4	LOBU067-12	KM572118	BOLD:AAE4008	Finland	Research Collection of Hannu Saarenmaa
Cosmotriche lobulina	MM18678	LEFIL368-10	JN303440	BOLD:AAE4008	Finland	University of Oulu
Cosmotriche lobulina	MM07392	LEFID927-10	HM873677	BOLD:AAE4008	Finland	University of Oulu
Cosmotriche lobulina	EA1.0E3	LOBU068-12	KM572285	BOLD:AAE4008	Finland	Research Collection of Hannu Saarenmaa
Cosmotriche lobulina	EA1.089	LOBU011-12	KM572316	BOLD:AAE4008	Finland	University of Eastern Finland

Cosmotriche lobulina	EA1.086	LOBU014-12	KM572767	BOLD:AAE4008	Finland	University of Eastern Finland
Cosmotriche lobulina	EA1.085	LOBU015-12	KM572653	BOLD:AAE4008	Finland	University of Eastern Finland
Cossus cossus	TLMF Lep 07551	PHLAG872-12	KM572562	BOLD:AAE4069	Austria	inatura, Dornbirn
Cossus cossus	MM06801	LEFID742-10	HM873499	BOLD:ACE8991	Finland	University of Oulu
Cossus cossus	MM00116	LEFIA1067-10	GU828604	BOLD:ACE8991	Finland	University of Oulu
Cossus cossus	MM00354	LEFIB066-10	HM870975	BOLD:ACE8991	Finland	University of Oulu
Crambus ericella	TLMF Lep 09955	PHLAW158-13	KM572010	BOLD:AAD6953	Austria	inatura, Dornbirn
Crambus ericella	MM03457	LEFIC121-10	HM871968	BOLD:AAD6953	Finland	University of Oulu
Crambus ericella	MM06368	LEFID436-10	HQ570332	BOLD:AAD6953	Finland	University of Oulu
Crambus ericella	MM06369	LEFID437-10	HQ570333	BOLD:AAD6953	Finland	University of Oulu
Crambus lathoniellus	MM01918	LEFIB431-10	HM871330	BOLD:AAC1691	Finland	University of Oulu
Crambus lathoniellus	MM11810	LEFIF415-10	HM875100	BOLD:AAC1691	Finland	University of Oulu
Crambus lathoniellus	MM00614	LEFIB187-10	HM871091	BOLD:AAC1691	Finland	University of Oulu
Crambus lathoniellus	TLMF Lep 08010	PHLAV191-12	KM572173	BOLD:AAC1691	Austria	inatura, Dornbirn
Crambus pascuella	MM10229	LEFIE903-10	HM874620	BOLD:ACF1131	Finland	University of Oulu
Crambus pascuella	TLMF Lep 08007	PHLAV188-12	KM572935	BOLD:ACF1131	Austria	inatura, Dornbirn
Crambus pascuella	MM01917	LEFIB430-10	HM871329	BOLD:ACF1131	Finland	University of Oulu
Crambus pascuella	MM04975	LEFIC805-10	HM872624	BOLD:ACF1131	Finland	University of Oulu
Crambus perlella	MM01915	LEFIB429-10	HM871328	BOLD:AAA6137	Finland	University of Oulu
Crambus perlella	MM08284	LEFIE100-10	HM873848	BOLD:AAA6137	Finland	University of Oulu
Crambus perlella	MM04976	LEFIC806-10	HM872625	BOLD:AAA6137	Finland	University of Oulu
Crambus perlella	MM06551	LEFID559-10	HM873324	BOLD:AAA6137	Finland	University of Oulu
Crambus perlella	MM02946	LEFIB875-10	HM871752	BOLD:AAA6137	Finland	University of Oulu
Crambus perlella	TLMF Lep 08006	PHLAV187-12	KM573411	BOLD:AAA6137	Austria	inatura, Dornbirn
Crambus pratella	TLMF Lep 09922	PHLAW125-13	KM572832	BOLD:AAA6138	Austria	inatura, Dornbirn
Crambus pratella	MM03258	LEFIC033-10	HM871903	BOLD:AAA6138	Finland	University of Oulu
Crambus pratella	MM13038	LEFIF791-10	HM875475	BOLD:AAA6138	Finland	University of Oulu
Crambus pratella	MM08095	LEFIE028-10	HM873777	BOLD:AAA6138	Finland	University of Oulu
Crambus silvella	TLMF Lep 12499	LEATC517-13	KM573078	BOLD:AAJ6529	Austria	Tiroler Landesmuseum Ferdinandeum
Crambus silvella	MM13048	LEFIF794-10	HM875478	BOLD:AAJ6529	Finland	University of Oulu
Crambus silvella	MM21088	LEFIJ1228-11	KM572796	BOLD:AAJ6529	Finland	University of Oulu

Crambus silvella	MM21087	LEFIJ1227-11	KM572611	BOLD:AAJ6529	Finland	University of Oulu
Crambus uliginosellus	TLMF Lep 08215	PHLAH396-12	KM572184	BOLD:AAD6941	Austria	inatura, Dornbirn
Crambus uliginosellus	MM15787	LEFIG923-10	HM876567	BOLD:AAD6941	Finland	University of Oulu
Crambus uliginosellus	MM03363	LEFIC074-10	HM871943	BOLD:AAD6941	Finland	University of Oulu
Crambus uliginosellus	MM03362	LEFIA1068-10	GU828691	BOLD:AAD6941	Finland	University of Oulu
Craniophora ligustri	MM06745	LEFIA1069-10	KM572583	BOLD:AAB6108	Finland	University of Oulu
Craniophora ligustri	MM06802	LEFID743-10	HM873500	BOLD:AAB6108	Finland	University of Oulu
Craniophora ligustri	MM09713	LEFIA838-10	HM386978	BOLD:AAB6108	Finland	University of Oulu
Craniophora ligustri	MM00137	LEFIB034-10	HM870943	BOLD:AAB6108	Finland	University of Oulu
Craniophora ligustri	TLMF Lep 07838	PHLAV019-12	KM573423	BOLD:AAB6108	Austria	inatura, Dornbirn
Crassa tinctella	MM03838	LEFIC332-10	HM872176	BOLD:AAE3946	Finland	University of Oulu
Crassa tinctella	MM13534	LEFIF960-10	HM875641	BOLD:AAE3946	Finland	University of Oulu
Crassa tinctella	MM04077	LEFIC481-10	HM872313	BOLD:AAE3946	Finland	University of Oulu
Crassa tinctella	TLMF Lep 08101	PHLAV282-12	KM572891	BOLD:AAE3946	Austria	inatura, Dornbirn
Crocallis elinguaria	MM02805	LEFIB839-10	HM871716	BOLD:AAB0677	Finland	University of Oulu
Crocallis elinguaria	MM01367	LEFIA320-10	HM386663	BOLD:AAB0677	Finland	University of Oulu
Crocallis elinguaria	MM01368	LEFIA321-10	HM386664	BOLD:AAB0677	Finland	University of Oulu
Crocallis elinguaria	TLMF Lep 06123	PHLSA668-11	KM572315	BOLD:AAB0677	Austria	Tiroler Landesmuseum Ferdinandeum
Crypsedra gemmea	MM04845	LEFIC742-10	HM872563	BOLD:AAF1443	Finland	University of Oulu
Crypsedra gemmea	MM01740	LEFIA625-10	HM870874	BOLD:AAF1443	Finland	University of Oulu
Crypsedra gemmea	MM00832	LEFIB309-10	HM871210	BOLD:AAF1443	Finland	University of Oulu
Crypsedra gemmea	TLMF Lep 08766	PHLAI271-13	KM572000	BOLD:AAF1443	Austria	Tiroler Landesmuseum Ferdinandeum
Cryptoblabes bistriga	MM10529	LEFIF080-10	HM874787	BOLD:AAD6231	Finland	University of Oulu
Cryptoblabes bistriga	MM15769	LEFIG905-10	HM876549	BOLD:AAD6231	Finland	University of Oulu
Cryptoblabes bistriga	TLMF Lep 08085	PHLAV266-12	KM572892	BOLD:AAD6231	Austria	inatura, Dornbirn
Cryptoblabes bistriga	TLMF Lep 09924	PHLAW127-13	KM572732	BOLD:AAD6231	Austria	inatura, Dornbirn
Cryptoblabes bistriga	MM15770	LEFIG906-10	HM876550	BOLD:AAD6231	Finland	University of Oulu
Cucullia asteris	MM09220	LEFIE456-10	HM874180	BOLD:ACF3254	Finland	University of Oulu
Cucullia asteris	MM18536	LEFII109-10	JF853346	BOLD:ACF3254	Finland	University of Oulu
Cucullia asteris	TLMF Lep 06109	PHLSA654-11	KM573054	BOLD:ACF3254	Austria	Tiroler Landesmuseum Ferdinandeum
Cucullia asteris	MM18537	LEFII110-10	JF853347	BOLD:ACF3254	Finland	University of Oulu

Cucullia lucifuga	MM17487	LEFIJ862-10	JF853865	BOLD:ABY6309	Finland	University of Oulu
Cucullia lucifuga	MM18723	LEFIL425-10	JF854594	BOLD:ABY6309	Finland	University of Oulu
Cucullia lucifuga	MM17486	LEFIJ861-10	JF853864	BOLD:ABY6309	Finland	University of Oulu
Cucullia lucifuga	MM19915	LEFII265-11	KM573675	BOLD:ABY6309	Finland	Research Collection of E. Laasonen
Cucullia lucifuga	TLMF Lep 04626	PHLAE311-11	JN266572	BOLD:ABY6309	Austria	Tiroler Landesmuseum Ferdinandeum
Cucullia umbratica	MM04914	LEFIC782-10	HM872601	BOLD:AAC0849	Finland	University of Oulu
Cucullia umbratica	MM04543	LEFIA1077-10	GU828701	BOLD:AAC0849	Finland	University of Oulu
Cucullia umbratica	MM14097	LEFIG210-10	HM875890	BOLD:AAC0849	Finland	University of Oulu
Cucullia umbratica	MM06742	LEFID693-10	HM873454	BOLD:AAC0849	Finland	University of Oulu
Cucullia umbratica	TLMF Lep 08120	PHLAV301-12	KM573523	BOLD:AAC0849	Austria	inatura, Dornbirn
Cupido minimus	TLMF Lep 08272	PHLAH453-12	KM573651	BOLD:AAA9082	Austria	inatura, Dornbirn
Cupido minimus	MM10580	LEFIF124-10	HM874825	BOLD:AAA9082	Finland	University of Oulu
Cupido minimus	MM10579	LEFIF123-10	HM874824	BOLD:AAA9082	Finland	University of Oulu
Cupido minimus	MM18666	LEFIL356-10	JN276922	BOLD:AAA9082	Finland	University of Oulu
Cyaniris semiargus	TLMF Lep 10016	LEATA409-13	KM573008	BOLD:AAB3027	Austria	inatura, Dornbirn
Cyaniris semiargus	MM17145	LEFIJ520-10	KM573316	BOLD:AAB3027	Finland	University of Oulu
Cyaniris semiargus	MM17143	LEFIJ518-10	JF853640	BOLD:AAB3027	Finland	University of Oulu
Cybosia mesomella	MM01030	LEFIA107-10	HM396454	BOLD:AAD1825	Finland	University of Oulu
Cybosia mesomella	MM01029	LEFIA106-10	HM396453	BOLD:AAD1825	Finland	University of Oulu
Cybosia mesomella	MM02909	LEFIB864-10	HM871741	BOLD:AAD1825	Finland	University of Oulu
Cybosia mesomella	TLMF Lep 06114	PHLSA659-11	KM573170	BOLD:AAD1825	Austria	Tiroler Landesmuseum Ferdinandeum
Cyclophora annularia	TLMF Lep 07867	PHLAV048-12	KF807390	BOLD:AAD6103	Austria	inatura, Dornbirn
Cyclophora annularia	MM05654	LEFID055-10	HM872869	BOLD:AAD6103	Finland	University of Oulu
Cyclophora linearia	TLMF Lep 04631	PHLAE316-11	JN285757	BOLD:ABX5086	Austria	Tiroler Landesmuseum Ferdinandeum
Cyclophora linearia	MM15918	LEFIJ318-10	KF807317	BOLD:ABX5086	Finland	University of Oulu
Cydia duplicana	MM08407	LEFIE157-10	HQ570359	BOLD:AAF7700	Finland	University of Oulu
Cydia duplicana	MM03040	LEFIB916-10	HM871793	BOLD:AAF7700	Finland	University of Oulu
Cydia duplicana	MM12137	LEFIF514-10	HM875199	BOLD:AAF7700	Finland	University of Oulu
Cydia duplicana	TLMF Lep 08295	PHLAH476-12	KM572874	BOLD:ABZ7551	Austria	inatura, Dornbirn
Cydia illutana	MM00966	LEFIB382-10	HM871281	BOLD:AAD5427	Finland	University of Oulu
Cydia illutana	MM00965	LEFIB381-10	HM871280	BOLD:AAD5427	Finland	University of Oulu

Cydia illutana	TLMF Lep 09947	PHLAW150-13	KM573340	BOLD:AAD5427	Austria	inatura, Dornbirn
Cydia illutana	TLMF Lep 08248	PHLAH429-12	KM572483	BOLD:AAD5427	Austria	inatura, Dornbirn
Cydia illutana	TLMF Lep 09389	PHLAI827-13	KM572976	BOLD:AAD5427	Austria	Tiroler Landesmuseum Ferdinandeum
Cydia illutana	MM15737	LEFIG873-10	HM876518	BOLD:AAD5427	Finland	University of Oulu
Cydia illutana	MM00964	LEFIB380-10	HM871279	BOLD:AAD5427	Finland	University of Oulu
Cydia illutana	MM18808	LEFIL510-10	KM572691	BOLD:AAD5427	Finland	University of Oulu
Cydia inquinatana	TLMF Lep 08094	PHLAV275-12	KM572501	BOLD:AAE3029	Austria	inatura, Dornbirn
Cydia inquinatana	MM03875	LEFIC355-10	HM872198	BOLD:AAE3029	Finland	University of Oulu
Cydia inquinatana	MM05013	LEFIC826-10	HM872645	BOLD:AAE3029	Finland	University of Oulu
Cydia inquinatana	MM03843	LEFIC336-10	HM872180	BOLD:AAE3029	Finland	University of Oulu
Cydia nigricana	MM03888	LEFIA1082-10	GU828696	BOLD:AAA7614	Finland	University of Oulu
Cydia nigricana	MM00968	LEFIB384-10	HM871283	BOLD:AAA7614	Finland	University of Oulu
Cydia nigricana	MM05216	LEFIC902-10	HM872719	BOLD:AAA7614	Finland	University of Oulu
Cydia nigricana	MM17902	LEFIK327-10	JF853973	BOLD:AAA7614	Finland	University of Oulu
Cydia nigricana	MM21167	LEFIJ1307-11	KM572938	BOLD:AAA7614	Finland	University of Oulu
Cydia nigricana	MM00967	LEFIB383-10	HM871282	BOLD:AAA7614	Finland	University of Oulu
Cydia nigricana	MM17903	LEFIK328-10	JF853974	BOLD:AAA7614	Finland	University of Oulu
Cydia nigricana	MM09437	LEFIE552-10	HM874275	BOLD:AAA7614	Finland	University of Oulu
Cydia nigricana	TLMF Lep 04289	PHLAE069-11	JN275060	BOLD:AAA7614	Austria	Tiroler Landesmuseum Ferdinandeum
Cydia nigricana	MM17895	LEFIK320-10	JF853967	BOLD:AAA7614	Finland	University of Oulu
Cydia pomonella	TLMF Lep 07926	PHLAV107-12	KM572180	BOLD:AAA3532	Austria	inatura, Dornbirn
Cydia pomonella	MM03480	LEFIC136-10	HM871982	BOLD:AAA3532	Finland	University of Oulu
Cydia pomonella	MM01761	LEFIA645-10	HM870894	BOLD:AAA3532	Finland	University of Oulu
Cydia pomonella	MM09453	LEFIE564-10	HM874287	BOLD:AAA3532	Finland	University of Oulu
Cydia splendana	TLMF Lep 08032	PHLAV213-12	KM572672	BOLD:AAC0640	Austria	inatura, Dornbirn
Cydia splendana	MM03458	LEFIC122-10	HM871969	BOLD:AAC0640	Finland	University of Oulu
Cydia splendana	MM08633	LEFIE286-10	HM874018	BOLD:ACJ4322	Finland	University of Oulu
Cydia splendana	MM03565	LEFIC203-10	HM872047	BOLD:ACJ4322	Finland	University of Oulu
Cydia splendana	MM13175	LEFIF833-10	HM875516	BOLD:ACJ4322	Finland	University of Oulu
Cydia strobilella	MM06291	LEFID380-10	HM873177	BOLD:AAD5907	Finland	University of Oulu
Cydia strobilella	TLMF Lep 09194	PHLAI632-13	KM572559	BOLD:AAD5907	Austria	Tiroler Landesmuseum Ferdinandeum

Cydia strobilella	MM06411	LEFID469-10	HM873237	BOLD:AAD5907	Finland	University of Oulu
Cydia strobilella	MM06412	LEFID470-10	HM873238	BOLD:AAD5907	Finland	University of Oulu
Cydia succedana	TLMF Lep 07699	PHLAH260-12	KM573579	BOLD:AAB7159	Austria	Tiroler Landesmuseum Ferdinandeum
Cydia succedana	MM18349	LEFIK774-10	JN274960	BOLD:AAB7159	Finland	University of Oulu
Cydia succedana	MM11038	LEFIF256-10	HM874949	BOLD:AAB7159	Finland	University of Oulu
Cydia succedana	MM18351	LEFIK776-10	JN274962	BOLD:AAB7159	Finland	University of Oulu
Cydia succedana	MM18350	LEFIK775-10	JN274961	BOLD:ACF3585	Finland	University of Oulu
Cydia succedana	MM11037	LEFIF255-10	HM874948	BOLD:ACF3585	Finland	University of Oulu
Cymolomia hartigiana	TLMF Lep 08213	PHLAH394-12	KM572711	BOLD:AAE3063	Austria	inatura, Dornbirn
Cymolomia hartigiana	MM13316	LEFIF902-10	HM875584	BOLD:ACF2297	Finland	University of Oulu
Cymolomia hartigiana	MM10356	LEFIE956-10	HM874673	BOLD:ACF2297	Finland	University of Oulu
Cymolomia hartigiana	MM03506	LEFIC160-10	HM872006	BOLD:ACF2297	Finland	University of Oulu
Deilephila elpenor	MM00971	LEFIA059-10	HM396406	BOLD:AAB2861	Finland	University of Oulu
Deilephila elpenor	MM05160	LEFIC881-10	HM872699	BOLD:AAB2861	Finland	University of Oulu
Deilephila elpenor	MM07300	LEFID909-10	HM873659	BOLD:AAB2861	Finland	University of Oulu
Deilephila elpenor	TLMF Lep 08527	PHLAH708-12	KM572616	BOLD:AAB2861	Austria	inatura, Dornbirn
Deileptenia ribeata	TLMF Lep 08187	PHLAV368-12	KM572215	BOLD:AAC3800	Austria	inatura, Dornbirn
Deileptenia ribeata	MM06638	LEFID621-10	HM873386	BOLD:AAC3800	Finland	University of Oulu
Deileptenia ribeata	MM06692	LEFID660-10	HM873422	BOLD:AAC3800	Finland	University of Oulu
Deileptenia ribeata	MM04588	LEFIC642-10	HM872463	BOLD:AAC3800	Finland	University of Oulu
Deltote bankiana	TLMF Lep 05625	PHLAF455-11	KM573186	BOLD:AAD6191	Austria	Tiroler Landesmuseum Ferdinandeum
Deltote bankiana	MM18721	LEFIL423-10	JF854593	BOLD:AAD6191	Finland	University of Oulu
Deltote bankiana	MM18720	LEFIL422-10	JF854592	BOLD:AAD6191	Finland	University of Oulu
Deltote bankiana	MM01751	LEFIA636-10	HM870885	BOLD:AAD6191	Finland	University of Oulu
Deltote pygarga	MM04676	LEFIC693-10	HM872514	BOLD:AAB9793	Finland	University of Oulu
Deltote pygarga	MM01427	LEFIA370-10	HM386712	BOLD:AAB9793	Finland	University of Oulu
Deltote pygarga	MM01426	LEFIA369-10	HM386711	BOLD:AAB9793	Finland	University of Oulu
Deltote pygarga	TLMF Lep 07861	PHLAV042-12	KM571979	BOLD:AAB9793	Austria	inatura, Dornbirn
Deltote pygarga	MM01512	LEFIA448-10	HM386789	BOLD:AAB9793	Finland	University of Oulu
Deltote uncula	MM04601	LEFIA1088-10	GU828703	BOLD:AAF4745	Finland	University of Oulu
Deltote uncula	TLMF Lep 10003	LEATA396-13	KM573452	BOLD:AAF4745	Austria	inatura, Dornbirn

Deltote uncula	MM02925	LEFIB868-10	HM871745	BOLD:AAF4745	Finland	University of Oulu
Deltote uncula	MM18523	LEFIK948-10	JF854386	BOLD:AAF4745	Finland	University of Oulu
Dendrolimus pini	MM08302	LEFIE113-10	HM873861	BOLD:AAB6845	Finland	University of Oulu
Dendrolimus pini	MM12533	LEFIF634-10	HM875318	BOLD:AAB6845	Finland	University of Oulu
Dendrolimus pini	TLMF Lep 06156	PHLSA701-11	KM572747	BOLD:AAB6845	Austria	Tiroler Landesmuseum Ferdinandeum
Dendrolimus pini	MM01054	LEFIA125-10	HM396472	BOLD:AAB6845	Finland	University of Oulu
Denisia similella	MM03830	LEFIC326-10	HM872170	BOLD:AAE3459	Finland	University of Oulu
Denisia similella	MM02559	LEFIB732-10	HM871610	BOLD:AAE3459	Finland	University of Oulu
Denisia similella	MM03061	LEFIB923-10	HM871800	BOLD:AAE3459	Finland	University of Oulu
Denisia similella	TLMF Lep 10042	LEATA435-13	KM572962	BOLD:AAE3459	Austria	inatura, Dornbirn
Denisia similella	MM13600	LEFIF993-10	HM875674	BOLD:AAE3459	Finland	University of Oulu
Denisia stipella	MM03060	LEFIB922-10	HM871799	BOLD:AAD6108	Finland	University of Oulu
Denisia stipella	MM14279	LEFIG313-10	HM875992	BOLD:AAD6108	Finland	University of Oulu
Denisia stipella	MM08273	LEFIE094-10	HM873841	BOLD:AAD6108	Finland	University of Oulu
Denisia stipella	TLMF Lep 09945	PHLAW148-13	KM573167	BOLD:AAD6108	Austria	inatura, Dornbirn
Denisia stipella	MM08046	LEFID997-10	HM873747	BOLD:AAD6108	Finland	University of Oulu
Denisia stipella	TLMF Lep 09390	PHLAI828-13	KM573692	BOLD:AAD6108	Austria	Tiroler Landesmuseum Ferdinandeum
Denisia stipella	TLMF Lep 09979	PHLAW182-13	KM572394	BOLD:AAD6108	Austria	inatura, Dornbirn
Denticucullus pygmina	MM04644	LEFIC676-10	HM872497	BOLD:AAE9000	Finland	University of Oulu
Denticucullus pygmina	MM02703	LEFIB794-10	HM871671	BOLD:AAE9000	Finland	University of Oulu
Denticucullus pygmina	TLMF Lep 08763	PHLAI268-13	KM572811	BOLD:AAE9000	Austria	Tiroler Landesmuseum Ferdinandeum
Denticucullus pygmina	MM00830	LEFIB308-10	HM871209	BOLD:AAE9000	Finland	University of Oulu
Depressaria chaerophylli	MM08546	LEFIE229-10	HM873963	BOLD:AAF8167	Finland	University of Oulu
Depressaria chaerophylli	TLMF Lep 07471	PHLAG792-12	KM572986	BOLD:AAF8167	Austria	inatura, Dornbirn
Depressaria chaerophylli	MM18137	LEFIK562-10	JF854154	BOLD:AAF8167	Finland	University of Oulu
Depressaria chaerophylli	MM08545	LEFIE228-10	HM873962	BOLD:AAF8167	Finland	University of Oulu
Depressaria olerella	MM05300	LEFIC940-10	HM872755	BOLD:AAF8185	Finland	University of Oulu
Depressaria olerella	TLMF Lep 12538	LEATC556-13	KM572552	BOLD:AAF8185	Austria	Tiroler Landesmuseum Ferdinandeum
Depressaria olerella	MM18139	LEFIK564-10	JF854155	BOLD:AAF8185	Finland	University of Oulu
Depressaria olerella	MM02216	LEFIB566-10	HM871448	BOLD:AAF8185	Finland	University of Oulu
Depressaria olerella	MM13352	LEFIF920-10	HM875602	BOLD:AAF8185	Finland	University of Oulu

Depressaria pimpinellae	MM03217	LEFIC012-10	HM871882	BOLD:AAD6055	Finland	University of Oulu
Depressaria pimpinellae	MM00438	LEFIA039-10	HM396388	BOLD:AAD6055	Finland	University of Oulu
Depressaria pimpinellae	TLMF Lep 12534	LEATC552-13	KM573269	BOLD:AAD6055	Austria	Tiroler Landesmuseum Ferdinandeum
Depressaria pimpinellae	MM02218	LEFIB567-10	HM871449	BOLD:AAD6055	Finland	University of Oulu
Depressaria pulcherrimella	TLMF Lep 07994	PHLAV175-12	KM572289	BOLD:AAF8186	Austria	inatura, Dornbirn
Depressaria pulcherrimella	MM08544	LEFIE227-10	HM873961	BOLD:AAF8186	Finland	University of Oulu
Depressaria pulcherrimella	MM08543	LEFIE226-10	HM873960	BOLD:AAF8186	Finland	University of Oulu
Depressaria pulcherrimella	MM14699	LEFIG554-10	HM876227	BOLD:AAF8186	Finland	University of Oulu
Depressaria radiella	MM05956	LEFID175-10	HM872982	BOLD:AAB6253	Finland	University of Oulu
Depressaria radiella	TLMF Lep 12510	LEATC528-13	KM573322	BOLD:AAB6253	Austria	Tiroler Landesmuseum Ferdinandeum
Depressaria radiella	MM13202	LEFIF848-10	HM875531	BOLD:AAB6253	Finland	University of Oulu
Depressaria radiella	MM13203	LEFIF849-10	HM875532	BOLD:AAB6253	Finland	University of Oulu
Diachrysia chrysitis	MM12629	LEFIF669-10	HM875353	BOLD:AAA6511	Finland	University of Oulu
Diachrysia chrysitis	MM12628	LEFIF668-10	HM875352	BOLD:AAA6511	Finland	University of Oulu
Diachrysia chrysitis	MM01113	LEFIA147-10	HM396493	BOLD:AAA6511	Finland	University of Oulu
Diachrysia chrysitis	MM01088	LEFIA140-10	HM396486	BOLD:AAA6511	Finland	University of Oulu
Diachrysia chrysitis	MM06639	LEFID622-10	HM873387	BOLD:AAA6511	Finland	University of Oulu
Diachrysia chrysitis	MM01089	LEFIA141-10	HM396487	BOLD:AAA6511	Finland	University of Oulu
Diachrysia chrysitis	MM01091	LEFIA143-10	HM396489	BOLD:AAA6511	Finland	University of Oulu
Diachrysia chrysitis	MM01092	LEFIA144-10	HM396490	BOLD:AAA6511	Finland	University of Oulu
Diachrysia chrysitis	MM04545	LEFIC614-10	HM872435	BOLD:AAA6511	Finland	University of Oulu
Diachrysia chrysitis	MM04546	LEFIC615-10	HM872436	BOLD:AAA6511	Finland	University of Oulu
Diachrysia chrysitis	MM04547	LEFIC616-10	HM872437	BOLD:AAA6511	Finland	University of Oulu
Diachrysia chrysitis	MM01111	LEFIA145-10	HM396491	BOLD:AAA6511	Finland	University of Oulu
Diachrysia chrysitis	MM01112	LEFIA146-10	HM396492	BOLD:AAA6511	Finland	University of Oulu
Diachrysia chrysitis	MM04571	LEFIC629-10	HM872450	BOLD:AAA6511	Finland	University of Oulu
Diachrysia chrysitis	MM04590	LEFIC643-10	HM872464	BOLD:AAA6511	Finland	University of Oulu
Diachrysia chrysitis	MM04591	LEFIC644-10	HM872465	BOLD:AAA6511	Finland	University of Oulu

Diachrysia chrysitis	MM04592	LEFIC645-10	HM872466	BOLD:AAA6511	Finland	University of Oulu
Diachrysia chrysitis	MM01114	LEFIA148-10	HM396494	BOLD:AAA6511	Finland	University of Oulu
Diachrysia chrysitis	MM01115	LEFIA149-10	HM396495	BOLD:AAA6511	Finland	University of Oulu
Diachrysia chrysitis	MM04593	LEFIC646-10	HM872467	BOLD:AAA6511	Finland	University of Oulu
Diachrysia chrysitis	MM04595	LEFIC648-10	HM872469	BOLD:AAA6511	Finland	University of Oulu
Diachrysia chrysitis	MM07316	LEFID915-10	HM873665	BOLD:AAA6511	Finland	University of Oulu
Diachrysia chrysitis	MM07314	LEFID913-10	HM873663	BOLD:AAA6511	Finland	University of Oulu
Diachrysia chrysitis	MM07315	LEFID914-10	HM873664	BOLD:AAA6511	Finland	University of Oulu
Diachrysia chrysitis	MM01090	LEFIA142-10	HM396488	BOLD:AAA6511	Finland	University of Oulu
Diachrysia chrysitis	MM07313	LEFID912-10	HM873662	BOLD:AAA6511	Finland	University of Oulu
Diachrysia chrysitis	MM07312	LEFID911-10	HM873661	BOLD:AAA6511	Finland	University of Oulu
Diachrysia chrysitis	MM07383	LEFID921-10	HM873671	BOLD:AAA6511	Finland	University of Oulu
Diachrysia chrysitis	MM04736	LEFIC707-10	HM872528	BOLD:AAA6511	Finland	University of Oulu
Diachrysia chrysitis	MM01103	LEFIB385-10	HM871284	BOLD:AAA6511	Finland	University of Oulu
Diachrysia chrysitis	MM07384	LEFID922-10	HM873672	BOLD:AAA6511	Finland	University of Oulu
Diachrysia chrysitis	MM07385	LEFID923-10	HM873673	BOLD:AAA6511	Finland	University of Oulu
Diachrysia chrysitis	MM04597	LEFIC650-10	HM872471	BOLD:AAA6511	Finland	University of Oulu
Diachrysia chrysitis	MM02748	LEFIB812-10	HM871689	BOLD:AAA6511	Finland	University of Oulu
Diachrysia chrysitis	MM10774	LEFIF187-10	HM874881	BOLD:AAA6511	Finland	University of Oulu
Diachrysia chrysitis	MM04594	LEFIC647-10	HM872468	BOLD:AAA6511	Finland	University of Oulu
Diachrysia chrysitis	MM01165	LEFIB388-10	HM871287	BOLD:AAA6511	Finland	University of Oulu
Diachrysia chrysitis	MM02749	LEFIB813-10	HM871690	BOLD:AAA6511	Finland	University of Oulu
Diachrysia chrysitis	TLMF Lep 08151	PHLAV332-12	KM573613	BOLD:AAA6511	Austria	inatura, Dornbirn
Diachrysia chrysitis	MM10773	LEFIF186-10	HM874880	BOLD:AAA6511	Finland	University of Oulu
Diachrysia chrysitis	MM10772	LEFIF185-10	HM874879	BOLD:AAA6511	Finland	University of Oulu
Diachrysia chrysitis	MM01104	LEFIB386-10	HM871285	BOLD:AAA6511	Finland	University of Oulu
Diachrysia chrysitis	MM01105	LEFIB387-10	HM871286	BOLD:AAA6511	Finland	University of Oulu
Diachrysia chrysitis	MM06977	LEFID851-10	HM873608	BOLD:AAA6511	Finland	University of Oulu
Diachrysia chrysitis	MM04596	LEFIC649-10	HM872470	BOLD:AAA6511	Finland	University of Oulu
Diachrysia chrysitis	MM10771	LEFIF184-10	HM874878	BOLD:AAA6511	Finland	University of Oulu
Diachrysia chrysitis	MM10770	LEFIF183-10	HM874877	BOLD:AAA6511	Finland	University of Oulu

Diachrysia chrysitis	MM01641	LEFIB399-10	HM871298	BOLD:AAA6511	Finland	University of Oulu
Diacrisia sannio	TLMF Lep 07550	PHLAG871-12	KM573005	BOLD:AAB8660	Austria	inatura, Dornbirn
Diacrisia sannio	MM01044	LEFIA119-10	HM396466	BOLD:AAB8660	Finland	University of Oulu
Diacrisia sannio	MM01043	LEFIA118-10	HM396465	BOLD:AAB8660	Finland	University of Oulu
Diacrisia sannio	MM05163	LEFIC882-10	HM872700	BOLD:AAB8660	Finland	University of Oulu
Diarsia brunnea	MM02773	LEFIB827-10	HM871704	BOLD:AAD6686	Finland	University of Oulu
Diarsia brunnea	TLMF Lep 08142	PHLAV323-12	KM572746	BOLD:AAD6686	Austria	inatura, Dornbirn
Diarsia brunnea	MM01653	LEFIA559-10	HQ963151	BOLD:AAD6686	Finland	University of Oulu
Diarsia brunnea	MM01654	LEFIA560-10	KM573198	BOLD:AAD6686	Finland	University of Oulu
Diarsia brunnea	MM18010	LEFIK435-10	KM573123	BOLD:AAD6686	Finland	University of Oulu
Diarsia mendica	MM08075	LEFIJ112-10	KM573041	BOLD:AAB0038	Finland	University of Oulu
Diarsia mendica	MM22780	LEFIJ1512-12	KM572819	BOLD:AAB0038	Finland	University of Oulu
Diarsia mendica	MM17915	LEFIK340-10	KM573297	BOLD:AAB0038	Finland	University of Oulu
Diarsia mendica	MM18008	LEFIK433-10	JF854058	BOLD:AAB0038	Finland	University of Oulu
Diarsia mendica	MM02776	LEFIJ046-10	KM572703	BOLD:AAB0038	Finland	University of Oulu
Diarsia mendica	MM01646	LEFIA553-10	KM573557	BOLD:AAB0038	Finland	University of Oulu
Diarsia mendica	MM01647	LEFIA554-10	KM572930	BOLD:AAB0038	Finland	University of Oulu
Diarsia mendica	MM09918	LEFIJ139-10	JF853453	BOLD:AAB0038	Finland	University of Oulu
Diarsia mendica	MM08462	LEFIJ120-10	KM573068	BOLD:AAB0038	Finland	University of Oulu
Diarsia mendica	MM08461	LEFIJ119-10	KM572453	BOLD:AAB0038	Finland	University of Oulu
Diarsia mendica	MM05112	LEFIJ068-10	KM573116	BOLD:ABZ6600	Finland	University of Oulu
Diarsia mendica	TLMF Lep 06107	PHLSA652-11	KM573299	BOLD:ABZ6600	Austria	Tiroler Landesmuseum Ferdinandeum
Diarsia mendica	MM02777	LEFIJ047-10	KM572872	BOLD:ABZ6600	Finland	University of Oulu
Diarsia mendica	MM10960	LEFIJ167-10	KM573112	BOLD:ABZ6600	Finland	University of Oulu
Diarsia mendica	MM18009	LEFIK434-10	KM572634	BOLD:ABZ6600	Finland	University of Oulu
Diarsia mendica	MM04091	LEFIA737-10	HM386881	BOLD:ABZ6600	Finland	University of Oulu
Diarsia rubi	MM01643	LEFIB401-10	HM871300	BOLD:ACE8687	Finland	University of Oulu
Diarsia rubi	MM10959	LEFIF218-10	HM874911	BOLD:ACE8687	Finland	University of Oulu
Diarsia rubi	MM15958	LEFIJ358-10	KM573582	BOLD:ACE8687	Finland	University of Oulu
Diarsia rubi	MM01644	LEFIA551-10	KM571953	BOLD:ACE8687	Finland	University of Oulu
Diarsia rubi	MM01642	LEFIB400-10	HM871299	BOLD:ACE8687	Finland	University of Oulu

Diarsia rubi	MM01645	LEFIA552-10	KM573133	BOLD:ACE8687	Finland	University of Oulu
Diarsia rubi	TLMF Lep 05621	PHLAF451-11	KM572934	BOLD:ACE8687	Austria	Tiroler Landesmuseum Ferdinandeum
Diarsia rubi	MM05102	LEFIC858-10	HM872676	BOLD:ACE8687	Finland	University of Oulu
Diarsia rubi	MM05103	LEFIC859-10	HM872677	BOLD:ACE8687	Finland	University of Oulu
Diarsia rubi	MM12689	LEFIF683-10	HM875367	BOLD:ACE8687	Finland	University of Oulu
Diasemia reticularis	MM10568	LEFIF113-10	HM874816	BOLD:AAC3558	Finland	University of Oulu
Diasemia reticularis	MM10569	LEFIF114-10	HM874817	BOLD:AAC3558	Finland	University of Oulu
Diasemia reticularis	TLMF Lep 09938	PHLAW141-13	KM573700	BOLD:AAC3558	Austria	inatura, Dornbirn
Diasemia reticularis	MM10570	LEFIF115-10	HM874818	BOLD:AAC3558	Finland	University of Oulu
Dicallomera fascelina	MM15848	LEFIG984-10	HM876623	BOLD:AAE7512	Finland	University of Oulu
Dicallomera fascelina	MM06746	LEFID695-10	HM873456	BOLD:AAE7512	Finland	University of Oulu
Dicallomera fascelina	TLMF Lep 06103	PHLSA648-11	KM573275	BOLD:AAE7512	Austria	Tiroler Landesmuseum Ferdinandeum
Dicallomera fascelina	MM08578	LEFIE250-10	HM873984	BOLD:AAE7512	Finland	University of Oulu
Dichelia histrionana	MM06817	LEFID755-10	HM873512	BOLD:ACF5563	Finland	University of Oulu
Dichelia histrionana	MM06755	LEFID702-10	HM873463	BOLD:ACF5563	Finland	University of Oulu
Dichelia histrionana	TLMF Lep 08083	PHLAV264-12	KM571974	BOLD:ACF5563	Austria	inatura, Dornbirn
Dichelia histrionana	MM10415	LEFIE981-10	HM874698	BOLD:ACF5563	Finland	University of Oulu
Dichomeris alacella	MM06878	LEFID801-10	HM873558	BOLD:AAF1347	Finland	University of Oulu
Dichomeris alacella	TLMF Lep 08444	PHLAH625-12	KM572197	BOLD:AAF1347	Austria	inatura, Dornbirn
Dichomeris alacella	MM04336	LEFIC559-10	HM872380	BOLD:AAF1347	Finland	University of Oulu
Dichomeris alacella	MM02572	LEFIB738-10	HM871616	BOLD:AAF1347	Finland	University of Oulu
Dichomeris latipennella	MM09839	LEFIE738-10	HM874458	BOLD:AAF1370	Finland	University of Oulu
Dichomeris latipennella	MM18252	LEFIK677-10	JF854245	BOLD:AAF1370	Finland	University of Oulu
Dichomeris latipennella	MM09840	LEFIE739-10	HM874459	BOLD:AAF1370	Finland	University of Oulu
Dichomeris latipennella	TLMF Lep 09961	PHLAW164-13	KM572570	BOLD:AAF1370	Austria	inatura, Dornbirn
Dichomeris limosellus	TLMF Lep 07999	PHLAV180-12	KM572668	BOLD:AAV6621	Austria	inatura, Dornbirn
Dichomeris limosellus	TLMF Lep 12496	LEATC514-13	KM572058	BOLD:AAV6621	Austria	Tiroler Landesmuseum Ferdinandeum
Dichomeris limosellus	TLMF Lep 09157	PHLAI595-13	KM572245	BOLD:AAV6621	Austria	Tiroler Landesmuseum Ferdinandeum
Dichomeris limosellus	MM17729	LEFIK154-10	KM573163	BOLD:AAV6621	Finland	University of Oulu
Dichrorampha alpinana	TLMF Lep 08427	PHLAH608-12	KM573341	BOLD:AAE7519	Austria	inatura, Dornbirn
Dichrorampha alpinana	MM14448	LEFIG420-10	HM876096	BOLD:AAE7519	Finland	University of Oulu

Dichrorampha alpinana	MM11046	LEFIF264-10	HM874957	BOLD:AAE7519	Finland	University of Oulu
Dichrorampha alpinana	MM11045	LEFIF263-10	HM874956	BOLD:ACF3984	Finland	University of Oulu
Dichrorampha simpliciana	MM03494	LEFIC149-10	HM871995	BOLD:AAD3189	Finland	University of Oulu
Dichrorampha simpliciana	TLMF Lep 07523	PHLAG844-12	KM573395	BOLD:AAD3189	Austria	Tiroler Landesmuseum Ferdinandeum
Dichrorampha simpliciana	MM02165	LEFIB545-10	HM871430	BOLD:AAD3189	Finland	University of Oulu
Dichrorampha simpliciana	MM13320	LEFIF905-10	HM875587	BOLD:AAD3189	Finland	University of Oulu
Dichrorampha simpliciana	TLMF Lep 08733	PHLAH929-12	KM573149	BOLD:AAD3189	Austria	Tiroler Landesmuseum Ferdinandeum
Digitivalva reticulella	MM18120	LEFIK545-10	JF854140	BOLD:AAE8385	Finland	University of Oulu
Digitivalva reticulella	MM06606	LEFID596-10	HM873361	BOLD:AAE8385	Finland	University of Oulu
Digitivalva reticulella	TLMF Lep 04506	PHLAE381-11	KM572764	BOLD:AAE8385	Austria	Tiroler Landesmuseum Ferdinandeum
Digitivalva reticulella	MM06036	LEFID207-10	HM873009	BOLD:AAE8385	Finland	University of Oulu
Diloba caeruleocephala	MM21049	LEFIJ1189-11	KM573425	BOLD:AAE8447	Finland	University of Oulu
Diloba caeruleocephala	MM15926	LEFIJ326-10	KM571949	BOLD:AAE8447	Finland	University of Oulu
Diloba caeruleocephala	TLMF Lep 08789	PHLAI294-13	KM571945	BOLD:AAE8447	Austria	Tiroler Landesmuseum Ferdinandeum
Diloba caeruleocephala	MM21050	LEFIJ1190-11	KM572557	BOLD:AAE8447	Finland	University of Oulu
Dioryctria abietella	MM17210	LEFIJ585-10	KM572860	BOLD:AAB6957	Finland	University of Oulu
Dioryctria abietella	MM02690	LEFIB785-10	HM871662	BOLD:AAB6957	Finland	University of Oulu
Dioryctria abietella	TLMF Lep 08004	PHLAV185-12	KM572006	BOLD:AAB6957	Austria	inatura, Dornbirn
Dioryctria abietella	MM17209	LEFIJ584-10	KM573496	BOLD:AAB6957	Finland	University of Oulu
Dioryctria simplicella	MM11900	LEFIF438-10	HM875123	BOLD:AAB6958	Finland	University of Oulu
Dioryctria simplicella	MM03545	LEFIC187-10	HM872031	BOLD:AAB6958	Finland	University of Oulu
Dioryctria simplicella	MM13008	LEFIF777-10	HM875461	BOLD:AAB6958	Finland	University of Oulu
Dioryctria simplicella	MM03792	LEFIC304-10	HM872148	BOLD:AAB6958	Finland	University of Oulu
Dioryctria simplicella	TLMF Lep 07878	PHLAV059-12	KM572541	BOLD:AAB6958	Austria	inatura, Dornbirn
Dioryctria simplicella	TLMF Lep 08087	PHLAV268-12	KM572205	BOLD:AAB6958	Austria	inatura, Dornbirn
Dioryctria sylvestrella	MM13006	LEFIF776-10	HM875460	BOLD:AAD0277	Finland	University of Oulu
Dioryctria sylvestrella	MM12120	LEFIF509-10	HM875194	BOLD:AAD0277	Finland	University of Oulu
Dioryctria sylvestrella	TLMF Lep 09990	LEATA383-13	KM573137	BOLD:AAD0277	Austria	inatura, Dornbirn
Dioryctria sylvestrella	MM03538	LEFIC183-10	HM872027	BOLD:AAD0277	Finland	University of Oulu
Dioryctria sylvestrella	TLMF Lep 12487	LEATC505-13	KM573674	BOLD:AAD0277	Austria	Tiroler Landesmuseum Ferdinandeum
Diurnea fagella	TLMF Lep 09776	LEATA359-13	KM572785	BOLD:AAF5356	Austria	Tiroler Landesmuseum Ferdinandeum

Diurnea fagella	MM05960	LEFIA1098-10	GU828735	BOLD:AAF5356	Finland	University of Oulu
Diurnea lipsiella	MM03178	LEFIB984-10	HM871856	BOLD:AAD9970	Finland	University of Oulu
Diurnea lipsiella	MM04207	LEFIC500-10	HM872325	BOLD:AAD9970	Finland	University of Oulu
Diurnea lipsiella	TLMF Lep 08804	PHLAI309-13	KM573063	BOLD:AAD9970	Austria	Tiroler Landesmuseum Ferdinandeum
Diurnea lipsiella	MM04511	LEFIC592-10	HM872413	BOLD:AAD9970	Finland	University of Oulu
Doloploca punctulana	TLMF Lep 07917	PHLAV098-12	KM572772	BOLD:AAF5268	Austria	inatura, Dornbirn
Doloploca punctulana	MM19945	LEFII295-11	KM573229	BOLD:AAF5268	Finland	Research Collection of J. Itaemies
Donacaula mucronella	TLMF Lep 09771	LEATA354-13	KM572961	BOLD:AAE8467	Austria	Tiroler Landesmuseum Ferdinandeum
Donacaula mucronella	MM10413	LEFIE979-10	HM874696	BOLD:AAE8467	Finland	University of Oulu
Donacaula mucronella	MM01892	LEFIB417-10	HM871316	BOLD:AAE8467	Finland	University of Oulu
Donacaula mucronella	MM13018	LEFIF782-10	HM875466	BOLD:AAE8467	Finland	University of Oulu
Dysstroma citrata	MM00799	LEFIB295-10	HM871196	BOLD:AAA2089	Finland	University of Oulu
Dysstroma citrata	MM01432	LEFIA375-10	HM386717	BOLD:AAA2089	Finland	University of Oulu
Dysstroma citrata	TLMF Lep 06168	PHLSA713-11	KM572076	BOLD:AAA2089	Austria	Tiroler Landesmuseum Ferdinandeum
Dysstroma citrata	MM01433	LEFIA376-10	HM386718	BOLD:AAA2089	Finland	University of Oulu
Dysstroma citrata	MM08113	LEFIE034-10	HM873783	BOLD:AAA2089	Finland	University of Oulu
Dysstroma truncata	MM01479	LEFIA417-10	HM386759	BOLD:AAA2864	Finland	University of Oulu
Dysstroma truncata	MM01478	LEFIA416-10	HM386758	BOLD:AAA2864	Finland	University of Oulu
Dysstroma truncata	MM08451	LEFIE176-10	HM873922	BOLD:AAA3843	Finland	University of Oulu
Dysstroma truncata	MM14622	LEFIG516-10	HM876189	BOLD:AAA3843	Finland	University of Oulu
Dysstroma truncata	MM06645	LEFIJ096-10	JF853443	BOLD:AAA3843	Finland	University of Oulu
Dysstroma truncata	MM10124	LEFIJ154-10	JF853467	BOLD:AAA3843	Finland	University of Oulu
Dysstroma truncata	MM18434	LEFIK859-10	KM572287	BOLD:AAA3843	Finland	University of Oulu
Dysstroma truncata	MM18433	LEFIK858-10	JF854320	BOLD:AAA3843	Finland	University of Oulu
Dysstroma truncata	MM05175	LEFIJ069-10	KM572719	BOLD:AAA3843	Finland	University of Oulu
Dysstroma truncata	MM11589	LEFIJ174-10	KM572667	BOLD:AAA3843	Finland	University of Oulu
Dysstroma truncata	MM18431	LEFIK856-10	JF854318	BOLD:AAA3843	Finland	University of Oulu
Dysstroma truncata	MM18614	LEFII187-10	JF853396	BOLD:AAA3843	Finland	University of Oulu
Dysstroma truncata	MM18432	LEFIK857-10	JF854319	BOLD:AAA3843	Finland	University of Oulu
Dysstroma truncata	MM08452	LEFIJ118-10	KM572381	BOLD:AAA3843	Finland	University of Oulu
Dysstroma truncata	TLMF Lep 04613	PHLAE298-11	JN279564	BOLD:AAA3843	Austria	Tiroler Landesmuseum Ferdinandeum

Eana argentana	MM06246	LEFID349-10	HM873146	BOLD:AAA8425	Finland	University of Oulu
Eana argentana	MM10012	LEFIE813-10	HM874532	BOLD:AAA8425	Finland	University of Oulu
Eana argentana	TLMF Lep 08431	PHLAH612-12	KM573672	BOLD:AAA8425	Austria	inatura, Dornbirn
Eana argentana	MM05253	LEFIC913-10	HM872730	BOLD:AAA8425	Finland	University of Oulu
Eana incanana	MM05265	LEFIC921-10	HM872738	BOLD:AAD7476	Finland	University of Oulu
Eana incanana	TLMF Lep 07534	PHLAG855-12	KM573597	BOLD:AAD7476	Austria	Tiroler Landesmuseum Ferdinandeum
Eana incanana	MM03044	LEFIB918-10	HM871795	BOLD:AAD7476	Finland	University of Oulu
Eana incanana	MM13331	LEFIF909-10	HM875591	BOLD:AAD7476	Finland	University of Oulu
Eana osseana	TLMF Lep 09152	PHLAI590-13	KM571988	BOLD:AAA6265	Austria	Tiroler Landesmuseum Ferdinandeum
Eana osseana	MM10348	LEFIE951-10	HM874668	BOLD:AAA6265	Finland	University of Oulu
Eana osseana	MM02993	LEFIB897-10	HM871774	BOLD:AAA6265	Finland	University of Oulu
Eana osseana	MM04140	LEFIA786-10	HM386927	BOLD:AAA6265	Finland	University of Oulu
Eana penziana	TLMF Lep 00926	PHLAB126-10	HM381496	BOLD:AAB8228	Austria	Tiroler Landesmuseum Ferdinandeum
Eana penziana	MM03500	LEFIC154-10	HM872000	BOLD:AAB8228	Finland	University of Oulu
Eana penziana	TLMF Lep 00925	PHLAB125-10	HM381495	BOLD:AAB8228	Austria	Tiroler Landesmuseum Ferdinandeum
Eana penziana	MM08293	LEFIE108-10	HM873856	BOLD:AAB8228	Finland	University of Oulu
Eana penziana	MM08314	LEFIE117-10	HM873865	BOLD:AAB8228	Finland	University of Oulu
Eana penziana	MM03455	LEFIC119-10	HM871966	BOLD:AAB8228	Finland	University of Oulu
Earias clorana	MM06650	LEFIA1107-10	GU828747	BOLD:AAC7224	Finland	University of Oulu
Earias clorana	MM14434	LEFIG408-10	HM876084	BOLD:AAC7224	Finland	University of Oulu
Earias clorana	MM00114	LEFIB028-10	HM870939	BOLD:AAC7224	Finland	University of Oulu
Earias clorana	TLMF Lep 08542	PHLAH723-12	KM572774	BOLD:AAC7224	Austria	inatura, Dornbirn
Earophila badiata	MM15803	LEFIG939-10	HM876581	BOLD:AAC3576	Finland	University of Oulu
Earophila badiata	MM15804	LEFIG940-10	HM876582	BOLD:AAC3576	Finland	University of Oulu
Earophila badiata	MM06000	LEFID199-10	HM873002	BOLD:AAC3576	Finland	University of Oulu
Earophila badiata	MM05959	LEFID176-10	HM872983	BOLD:AAC3576	Finland	University of Oulu
Earophila badiata	TLMF Lep 08815	PHLAI320-13	KM573287	BOLD:AAC3576	Austria	Tiroler Landesmuseum Ferdinandeum
Ecliptopera capitata	MM18426	LEFIK851-10	JN279411	BOLD:AAC9011	Finland	University of Oulu
Ecliptopera capitata	MM10468	LEFIF029-10	HM874740	BOLD:AAC9011	Finland	University of Oulu
Ecliptopera capitata	TLMF Lep 07889	PHLAV070-12	KM572160	BOLD:AAC9011	Austria	inatura, Dornbirn
Ecliptopera capitata	MM01374	LEFIA327-10	HM386670	BOLD:AAC9011	Finland	University of Oulu

Ecliptopera silaceata	MM01321	LEFIA281-10	HM386625	BOLD:AAA2586	Finland	University of Oulu
Ecliptopera silaceata	MM08169	LEFIE053-10	HM873801	BOLD:AAA2586	Finland	University of Oulu
Ecliptopera silaceata	TLMF Lep 07849	PHLAV030-12	KM572878	BOLD:AAA2586	Austria	inatura, Dornbirn
Ecliptopera silaceata	MM01322	LEFIA282-10	HM386626	BOLD:AAA2586	Finland	University of Oulu
Ectoedemia sericopeza	TLMF Lep 07484	PHLAG805-12	KM572400	BOLD:AAC4337	Austria	inatura, Dornbirn
Ectoedemia sericopeza	MM17329	LEFIJ704-10	KM573558	BOLD:AAC4337	Finland	University of Oulu
Ectoedemia sericopeza	MM14880	LEFIJ264-10	JF853504	BOLD:AAC4337	Finland	University of Oulu
Ectoedemia sericopeza	MM21131	LEFIJ1271-11	KM572525	BOLD:AAC4337	Finland	University of Oulu
Ectoedemia sericopeza	MM21037	LEFIJ1177-11	KM572908	BOLD:AAC4337	Finland	University of Oulu
Ectropis crepuscularia	MM17219	LEFIJ594-10	JF853694	BOLD:AAA2076	Finland	University of Oulu
Ectropis crepuscularia	MM06210	LEFID322-10	HM873119	BOLD:AAA2076	Finland	University of Oulu
Ectropis crepuscularia	MM11593	LEFIJ175-10	KM573644	BOLD:AAA2076	Finland	University of Oulu
Ectropis crepuscularia	MM11592	LEFIF371-10	HM875056	BOLD:AAA2076	Finland	University of Oulu
Ectropis crepuscularia	MM12856	LEFIF734-10	HM875418	BOLD:AAA2076	Finland	University of Oulu
Ectropis crepuscularia	MM22781	LEFIJ1513-12	KM572098	BOLD:AAA2076	Finland	University of Oulu
Ectropis crepuscularia	MM14383	LEFIG377-10	HM876054	BOLD:AAA2076	Finland	University of Oulu
Ectropis crepuscularia	MM18512	LEFIK937-10	JF854378	BOLD:AAA2076	Finland	University of Oulu
Ectropis crepuscularia	MM17370	LEFIJ745-10	JF853800	BOLD:AAA2076	Finland	University of Oulu
Ectropis crepuscularia	MM00472	LEFIB118-10	HM871024	BOLD:AAA2076	Finland	University of Oulu
Ectropis crepuscularia	MM00643	LEFIB200-10	HM871104	BOLD:AAA2076	Finland	University of Oulu
Ectropis crepuscularia	MM14382	LEFIG376-10	HM876053	BOLD:AAA2076	Finland	University of Oulu
Ectropis crepuscularia	MM17992	LEFIK417-10	JF854046	BOLD:AAA2076	Finland	University of Oulu
Ectropis crepuscularia	MM07792	LEFIJ104-10	KM573109	BOLD:ACE6053	Finland	University of Oulu
Ectropis crepuscularia	TLMF Lep 08176	PHLAV357-12	KM572883	BOLD:ACE6053	Austria	inatura, Dornbirn
Ectropis crepuscularia	MM04502	LEFIC585-10	HM872406	BOLD:ACE6053	Finland	University of Oulu
Ectropis crepuscularia	MM01293	LEFIA256-10	HM386600	BOLD:ACE6053	Finland	University of Oulu
Ectropis crepuscularia	MM07791	LEFID949-10	HM873699	BOLD:ACE6053	Finland	University of Oulu
Ectropis crepuscularia	MM01428	LEFIA371-10	HM386713	BOLD:ACE6053	Finland	University of Oulu
Ectropis crepuscularia	MM18511	LEFIK936-10	JN268644	BOLD:ACE6053	Finland	University of Oulu
Eilema complana	MM01025	LEFIA102-10	HM396449	BOLD:AAB6846	Finland	University of Oulu
Eilema complana	MM01024	LEFIA101-10	HM396448	BOLD:AAB6846	Finland	University of Oulu

Eilema complana	MM04867	LEFIC752-10	HM872571	BOLD:AAB6846	Finland	University of Oulu
Eilema complana	TLMF Lep 06113	PHLSA658-11	KM572919	BOLD:AAB6846	Austria	Tiroler Landesmuseum Ferdinandeum
Eilema depressa	TLMF Lep 08554	PHLAH735-12	KM573598	BOLD:AAB6834	Austria	inatura, Dornbirn
Eilema depressa	MM01032	LEFIA109-10	HM396456	BOLD:AAB6834	Finland	University of Oulu
Eilema depressa	MM04890	LEFIC766-10	HM872585	BOLD:AAB6834	Finland	University of Oulu
Eilema depressa	MM01031	LEFIA108-10	HM396455	BOLD:AAB6834	Finland	University of Oulu
Eilema depressa	MM04891	LEFIC767-10	HM872586	BOLD:AAB6834	Finland	University of Oulu
Eilema griseola	TLMF Lep 08539	PHLAH720-12	KM572212	BOLD:AAC1074	Austria	inatura, Dornbirn
Eilema griseola	TLMF Lep 08794	PHLAI299-13	KM573252	BOLD:AAC1074	Austria	Tiroler Landesmuseum Ferdinandeum
Eilema griseola	MM11628	LEFIF381-10	HM875066	BOLD:AAC1074	Finland	University of Oulu
Eilema griseola	MM01164	LEFIA179-10	HM396524	BOLD:AAC1074	Finland	University of Oulu
Eilema griseola	MM01026	LEFIA103-10	HM396450	BOLD:AAC1074	Finland	University of Oulu
Eilema lurideola	MM01022	LEFIA099-10	HM396446	BOLD:AAB6833	Finland	University of Oulu
Eilema lurideola	MM01023	LEFIA100-10	HM396447	BOLD:AAB6833	Finland	University of Oulu
Eilema lurideola	MM17199	LEFIJ574-10	JF853687	BOLD:AAB6833	Finland	University of Oulu
Eilema lurideola	TLMF Lep 08198	PHLAV379-12	KM573461	BOLD:AAB6833	Austria	inatura, Dornbirn
Eilema lurideola	MM20534	LEEUA665-11	KM572164	BOLD:AAB6833	Finland	Research Collection of Timo Leponiemi
Eilema sororcula	MM07930	LEFID960-10	HM873710	BOLD:AAC1077	Finland	University of Oulu
Eilema sororcula	MM01831	LEFIA690-10	HM386835	BOLD:AAC1077	Finland	University of Oulu
Eilema sororcula	MM03862	LEFIC346-10	HM872189	BOLD:AAC1077	Finland	University of Oulu
Eilema sororcula	TLMF Lep 07862	PHLAV043-12	KM573277	BOLD:AAL5773	Austria	inatura, Dornbirn
Elachista adscitella	MM09261	LEFIE484-10	HM874208	BOLD:AAC3846	Finland	University of Oulu
Elachista adscitella	TLMF Lep 08412	PHLAH593-12	KM572870	BOLD:AAC3846	Austria	inatura, Dornbirn
Elachista adscitella	MM06727	LEFID683-10	HM873444	BOLD:AAC3846	Finland	University of Oulu
Elachista adscitella	MM09260	LEFIE483-10	HM874207	BOLD:AAC3846	Finland	University of Oulu
Elachista adscitella	MM12026	LEFIF477-10	HM875162	BOLD:AAC3846	Finland	University of Oulu
Elachista adscitella	MM09737	LEFIA860-10	HM386998	BOLD:AAC3846	Finland	University of Oulu
Elachista adscitella	MM13721	LEFIG071-10	HM875750	BOLD:AAC3846	Finland	University of Oulu
Elachista adscitella	MM10399	LEFIE973-10	HM874690	BOLD:AAC3846	Finland	University of Oulu
Elachista adscitella	MM20018	ELACA780-11	JN267107	BOLD:AAC3846	Finland	Research Collection of Jukka Tabell

Elachista adscitella	MM02657	LEFIA721-10	HM386865	BOLD:AAC3846	Finland	University of Oulu
Elachista adscitella	MM12027	LEFIF478-10	HM875163	BOLD:AAC3846	Finland	University of Oulu
Elachista adscitella	MM02658	LEFIA722-10	HM386866	BOLD:AAC3846	Finland	University of Oulu
Elachista adscitella	MM20017	ELACA779-11	JN267106	BOLD:AAC3846	Finland	Research Collection of Jukka Tabell
Elachista albidella	MM16211	ELACA296-10	KM573626	BOLD:AAE9972	Finland	University of Oulu
Elachista albidella	TLMF Lep 09966	PHLAW169-13	KM573108	BOLD:AAE9972	Austria	inatura, Dornbirn
Elachista albidella	MM10432	LEFIE994-10	HQ570377	BOLD:AAE9972	Finland	University of Oulu
Elachista albidella	MM03911	LEFIC381-10	HM872224	BOLD:AAE9972	Finland	University of Oulu
Elachista albidella	MM17964	LEFIK389-10	JF854021	BOLD:AAE9972	Finland	University of Oulu
Elachista albidella	MM15572	LEFIG708-10	HM876364	BOLD:AAE9972	Finland	University of Oulu
Elachista albidella	MM15571	LEFIG707-10	HM876363	BOLD:AAE9972	Finland	University of Oulu
Elachista albidella	MM17554	LEFIJ929-10	JF853902	BOLD:AAE9972	Finland	University of Oulu
Elachista albidella	MM03237	LEFIC024-10	HM871894	BOLD:AAE9972	Finland	University of Oulu
Elachista albidella	MM03912	LEFIC382-10	HM872225	BOLD:ABX6815	Finland	University of Oulu
Elachista albidella	MM06596	LEFID588-10	HM873353	BOLD:ABX6815	Finland	University of Oulu
Elachista albidella	MM06597	LEFID589-10	HM873354	BOLD:ABX6815	Finland	University of Oulu
Elachista albidella	MM16213	ELACA298-10	JF847438	BOLD:ACF5411	Finland	University of Oulu
Elachista albidella	MM16209	ELACA294-10	KM572877	BOLD:ACF5411	Finland	University of Oulu
Elachista albifrontella	MM14235	LEFIG287-10	HM875966	BOLD:AAE0022	Finland	University of Oulu
Elachista albifrontella	MM22908	LEFIJ1621-13	KM573627	BOLD:AAE0022	Finland	University of Oulu
Elachista albifrontella	MM06598	LEFID590-10	HM873355	BOLD:AAE0022	Finland	University of Oulu
Elachista albifrontella	MM06514	LEFID531-10	HM873296	BOLD:AAE0022	Finland	University of Oulu
Elachista albifrontella	MM06161	LEFID286-10	HM873084	BOLD:AAE0022	Finland	University of Oulu
Elachista albifrontella	MM12029	LEFIF479-10	HM875164	BOLD:AAE0022	Finland	University of Oulu
Elachista albifrontella	MM02662	LEFIA725-10	HM386869	BOLD:AAE0022	Finland	University of Oulu
Elachista albifrontella	TLMF Lep 09951	PHLAW154-13	KM573139	BOLD:AAE0022	Austria	inatura, Dornbirn
Elachista albifrontella	MM14309	LEFIG333-10	HM876012	BOLD:AAE0022	Finland	University of Oulu
Elachista canapennella	MM06565	LEFID571-10	HM873336	BOLD:AAB7810	Finland	University of Oulu
Elachista canapennella	TLMF Lep 07991	PHLAV172-12	KM572024	BOLD:AAB7810	Austria	inatura, Dornbirn
Elachista canapennella	MM14269	LEFIG306-10	HM875985	BOLD:AAB7810	Finland	University of Oulu
Elachista canapennella	MM05360	LEFIC968-10	HM872783	BOLD:AAB7810	Finland	University of Oulu

Elachista canapennella	MM02670	LEFIA730-10	HM386874	BOLD:AAB7810	Finland	University of Oulu
Elachista canapennella	MM02669	LEFIA729-10	HM386873	BOLD:AAB7810	Finland	University of Oulu
Elachista canapennella	MM18826	LEFIL528-10	KF809284	BOLD:AAB7810	Finland	University of Oulu
Elachista canapennella	MM13967	LEFIG137-10	HM875817	BOLD:AAB7810	Finland	University of Oulu
Elachista canapennella	MM13896	LEFIG108-10	HM875788	BOLD:AAB7810	Finland	University of Oulu
Elachista canapennella	TLMF Lep 09952	PHLAW155-13	KM573602	BOLD:AAB7810	Austria	inatura, Dornbirn
Elachista canapennella	MM14445	LEFIG418-10	HM876094	BOLD:AAB7810	Finland	University of Oulu
Elachista canapennella	MM14542	LEFIG483-10	HM876159	BOLD:AAB7810	Finland	University of Oulu
Elachista canapennella	MM05361	LEFIC969-10	HM872784	BOLD:AAB7810	Finland	University of Oulu
Elachista canapennella	MM09868	LEFIE751-10	HM874471	BOLD:AAB7810	Finland	University of Oulu
Elachista canapennella	MM06187	LEFID307-10	HM873104	BOLD:AAB7810	Finland	University of Oulu
Elachista canapennella	MM21092	LEFIJ1232-11	KF809343	BOLD:AAB7810	Finland	University of Oulu
Elachista canapennella	MM06302	LEFID390-10	HM873187	BOLD:AAB7810	Finland	University of Oulu
Elachista canapennella	MM06523	LEFID539-10	HM873304	BOLD:AAB7810	Finland	University of Oulu
Elachista compsa	MM10317	LEFIE933-10	HM874650	BOLD:AAC5666	Finland	University of Oulu
Elachista compsa	MM12148	LEFIF519-10	HM875204	BOLD:AAC5666	Finland	University of Oulu
Elachista compsa	MM10423	LEFIE985-10	HM874701	BOLD:AAC5666	Finland	University of Oulu
Elachista compsa	MM12149	LEFIF520-10	HM875205	BOLD:AAC5666	Finland	University of Oulu
Elachista compsa	MM10424	LEFIE986-10	HM874702	BOLD:AAC5666	Finland	University of Oulu
Elachista compsa	MM22906	LEFIJ1619-13	KM572103	BOLD:AAC5666	Finland	University of Oulu
Elachista compsa	TLMF Lep 09211	PHLAI649-13	KM573309	BOLD:AAC5666	Austria	Tiroler Landesmuseum Ferdinandeum
Elachista compsa	MM20043	ELACA805-11	JN267127	BOLD:AAC5666	Finland	Research Collection of Jukka Tabell
Elachista compsa	MM18827	LEFIL529-10	JF854641	BOLD:AAC5666	Finland	University of Oulu
Elachista freyerella	MM13726	LEFIG076-10	HM875755	BOLD:AAD9985	Finland	University of Oulu
Elachista freyerella	MM18158	LEFIK583-10	JF854171	BOLD:AAD9985	Finland	University of Oulu
Elachista freyerella	MM13725	LEFIG075-10	HM875754	BOLD:AAD9985	Finland	University of Oulu
Elachista freyerella	MM18156	LEFIK581-10	JF854169	BOLD:AAD9985	Finland	University of Oulu
Elachista freyerella	MM18157	LEFIK582-10	JF854170	BOLD:AAD9985	Finland	University of Oulu
Elachista freyerella	MM13724	LEFIG074-10	HM875753	BOLD:AAD9985	Finland	University of Oulu
Elachista freyerella	MM13722	LEFIG072-10	HM875751	BOLD:AAD9985	Finland	University of Oulu
Elachista freyerella	TLMF Lep 07990	PHLAV171-12	KM572971	BOLD:AAD9985	Austria	inatura, Dornbirn

Elachista freyerella	TLMF Lep 07956	PHLAV137-12	KM572114	BOLD:AAD9985	Austria	inatura, Dornbirn
Elachista freyerella	MM13723	LEFIG073-10	HM875752	BOLD:AAD9985	Finland	University of Oulu
Elachista gleichenella	MM09727	LEFIA850-10	HM386990	BOLD:AAD8163	Finland	University of Oulu
Elachista gleichenella	MM06595	LEFID587-10	HM873352	BOLD:AAD8163	Finland	University of Oulu
Elachista gleichenella	MM03998	LEFIC440-10	HM872274	BOLD:AAD8163	Finland	University of Oulu
Elachista gleichenella	MM06164	LEFID289-10	HM873087	BOLD:AAD8163	Finland	University of Oulu
Elachista gleichenella	TLMF Lep 09181	PHLAI619-13	KM573521	BOLD:AAD8163	Austria	Tiroler Landesmuseum Ferdinandeum
Elachista gleichenella	TLMF Lep 10047	LEATA440-13	KM573233	BOLD:AAD8163	Austria	inatura, Dornbirn
Elachista gleichenella	MM09265	LEFIE486-10	HM874210	BOLD:AAD8163	Finland	University of Oulu
Elachista gleichenella	MM03999	LEFIC441-10	HM872275	BOLD:AAD8163	Finland	University of Oulu
Elachista maculicerusella	MM16230	ELACA315-10	KF809365	BOLD:AAC7924	Finland	University of Oulu
Elachista maculicerusella	MM02647	LEFIB773-10	HM871650	BOLD:AAC7924	Finland	University of Oulu
Elachista maculicerusella	TLMF Lep 08422	PHLAH603-12	KM572429	BOLD:AAC7924	Austria	inatura, Dornbirn
Elachista maculicerusella	MM09733	LEFIA856-10	HQ570259	BOLD:AAC7924	Finland	University of Oulu
Elachista maculicerusella	MM17940	LEFIK365-10	JF853999	BOLD:AAC7924	Finland	University of Oulu
Elachista maculicerusella	MM03985	LEFIC433-10	HM872268	BOLD:AAC7924	Finland	University of Oulu
Elachista maculicerusella	MM02648	LEFIB774-10	HM871651	BOLD:AAC7924	Finland	University of Oulu
Elachista maculicerusella	MM14357	LEFIG359-10	HM876036	BOLD:AAC7924	Finland	University of Oulu
Elachista maculicerusella	MM16232	ELACA317-10	JF847443	BOLD:AAC7924	Finland	University of Oulu
Elachista maculicerusella	MM03986	LEFIC434-10	HM872269	BOLD:AAC7924	Finland	University of Oulu
Elachista maculicerusella	MM12025	LEFIF476-10	HM875161	BOLD:AAC7924	Finland	University of Oulu
Elachista maculicerusella	MM00861	LEFIB324-10	HM871224	BOLD:AAC7925	Finland	University of Oulu
Elachista occidentalis	MM03961	LEFIC415-10	HM872258	BOLD:AAD9988	Finland	University of Oulu
Elachista occidentalis	TLMF Lep 08401	PHLAH582-12	KM572510	BOLD:AAD9988	Austria	inatura, Dornbirn
Elachista occidentalis	MM15567	LEFIG703-10	HM876359	BOLD:AAD9988	Finland	University of Oulu
Elachista occidentalis	MM03962	LEFIC416-10	HM872259	BOLD:AAD9988	Finland	University of Oulu
Elachista occidentalis	MM15568	LEFIG704-10	HM876360	BOLD:AAD9988	Finland	University of Oulu
Elachista occidentalis	MM16217	ELACA302-10	KM572487	BOLD:AAD9988	Finland	University of Oulu
Elachista occidentalis	MM03979	LEFIC430-10	HM872266	BOLD:AAD9988	Finland	University of Oulu
Elachista occidentalis	MM16216	ELACA301-10	KM573029	BOLD:AAD9988	Finland	University of Oulu

Elachista occidentalis	MM21339	ELACA1276-12	KM572884	BOLD:AAD9988	Finland	Research Collection of Jari Junnilainen
Elachista subalbidella	MM03154	LEFIB968-10	HM871842	BOLD:AAC7905	Finland	University of Oulu
Elachista subalbidella	MM08055	LEFIE004-10	HM873753	BOLD:AAC7905	Finland	University of Oulu
Elachista subalbidella	MM05332	LEFIC960-10	HM872775	BOLD:AAC7905	Finland	University of Oulu
Elachista subalbidella	MM16735	ELACA509-10	JF847563	BOLD:AAC7905	Finland	University of Oulu
Elachista subalbidella	MM05490	LEFID036-10	HM872850	BOLD:AAC7905	Finland	University of Oulu
Elachista subalbidella	MM09037	LEFIE426-10	HM874150	BOLD:AAC7905	Finland	University of Oulu
Elachista subalbidella	TLMF Lep 02843	PHLAC808-10	JF860325	BOLD:AAC7905	Austria	Tiroler Landesmuseum Ferdinandeum
Elachista subalbidella	MM20040	ELACA802-11	JN267125	BOLD:AAC7905	Finland	Research Collection of Jukka Tabell
Elachista subalbidella	MM18154	LEFIK579-10	JF854167	BOLD:AAC7905	Finland	University of Oulu
Elachista subalbidella	TLMF Lep 02844	PHLAC809-10	JF860326	BOLD:AAC7905	Austria	Tiroler Landesmuseum Ferdinandeum
Elachista subalbidella	BIOUG04116-A02	GMFID719-12	KM572609	BOLD:AAC7905	Finland	Biodiversity Institute of Ontario
Elachista subalbidella	MM02650	LEFIB775-10	HM871652	BOLD:AAC7905	Finland	University of Oulu
Elachista subalbidella	MM13727	LEFIG077-10	HM875756	BOLD:AAC7905	Finland	University of Oulu
Elachista subalbidella	MM02651	LEFIB776-10	HM871653	BOLD:AAC7905	Finland	University of Oulu
Elachista subalbidella	MM06221	LEFID328-10	HM873125	BOLD:AAC7905	Finland	University of Oulu
Elachista subalbidella	MM14064	LEFIG191-10	HM875871	BOLD:AAC7905	Finland	University of Oulu
Elachista zernyi	MM17654	LEFIK079-10	KF809257	BOLD:AAP5631	Finland	University of Oulu
Elachista zernyi	TLMF Lep 10052	LEATA445-13	KM572702	BOLD:AAP5631	Austria	inatura, Dornbirn
Elachista zernyi	MM22606	LEFIJ1590-12	KF809274	BOLD:AAP5631	Finland	Research Collection of Jari-Pekka Kaitila
Elaphria venustula	MM18915	LEFIL617-10	KM573178	BOLD:AAE0188	Finland	University of Oulu
Elaphria venustula	MM11069	LEFIF287-10	HM874977	BOLD:AAE0188	Finland	University of Oulu
Elaphria venustula	MM06618	LEFID605-10	HM873370	BOLD:AAE0188	Finland	University of Oulu
Elaphria venustula	TLMF Lep 08158	PHLAV339-12	KM572617	BOLD:AAE0188	Austria	inatura, Dornbirn
Electrophaes corylata	TLMF Lep 04633	PHLAE318-11	JN279566	BOLD:AAC3785	Austria	Tiroler Landesmuseum Ferdinandeum
Electrophaes corylata	MM18702	LEFIL404-10	KM572534	BOLD:AAC3785	Finland	University of Oulu
Electrophaes corylata	MM01277	LEFIA244-10	HM386588	BOLD:AAC3785	Finland	University of Oulu
Electrophaes corylata	MM11619	LEFIF376-10	HM875061	BOLD:AAC3785	Finland	University of Oulu
Electrophaes corylata	MM01276	LEFIA243-10	HM386587	BOLD:AAC3785	Finland	University of Oulu

Elophila nymphaeata	MM12379	LEFIF579-10	HM875263	BOLD:AAC4080	Finland	University of Oulu
Elophila nymphaeata	MM01074	LEFIA1114-10	GU828657	BOLD:AAC4080	Finland	University of Oulu
Elophila nymphaeata	TLMF Lep 08207	PHLAH388-12	KM572590	BOLD:AAC4080	Austria	inatura, Dornbirn
Elophila nymphaeata	MM04808	LEFIC732-10	HM872553	BOLD:AAC4080	Finland	University of Oulu
Elophila nymphaeata	MM08811	LEFIE363-10	HM874087	BOLD:AAC4080	Finland	University of Oulu
Elophos vittaria	MM04136	LEFIA782-10	HM386923	BOLD:AAD3009	Finland	University of Oulu
Elophos vittaria	MM14085	LEFIG202-10	HM875882	BOLD:AAD3009	Finland	University of Oulu
Elophos vittaria	MM02824	LEFIB844-10	HM871721	BOLD:AAD3009	Finland	University of Oulu
Elophos vittaria	TLMF Lep 09996	LEATA389-13	KM573533	BOLD:AAD3010	Austria	inatura, Dornbirn
Ematurga atomaria	MM00501	LEFIB132-10	HM871038	BOLD:AAC1877	Finland	University of Oulu
Ematurga atomaria	TLMF Lep 10012	LEATA405-13	KM572072	BOLD:AAC1877	Austria	inatura, Dornbirn
Ematurga atomaria	MM18510	LEFIK935-10	JF854377	BOLD:AAC1877	Finland	University of Oulu
Ematurga atomaria	MM00562	LEFIB165-10	HM871069	BOLD:AAC1877	Finland	University of Oulu
Emmelina monodactyla	TLMF Lep 08421	PHLAH602-12	KM572213	BOLD:ACE4862	Austria	inatura, Dornbirn
Emmelina monodactyla	TLMF Lep 08009	PHLAV190-12	KM572509	BOLD:ACE4862	Austria	inatura, Dornbirn
Emmelina monodactyla	MM09764	LEFIA887-10	HM387024	BOLD:ACE4862	Finland	University of Oulu
Emmelina monodactyla	MM04982	LEFIC808-10	HM872627	BOLD:ACE4862	Finland	University of Oulu
Emmelina monodactyla	MM13389	LEFIF939-10	HM875621	BOLD:ACE4862	Finland	University of Oulu
Enargia paleacea	MM12553	LEFIF645-10	HM875329	BOLD:AAA7455	Finland	University of Oulu
Enargia paleacea	MM02745	LEFIB811-10	HM871688	BOLD:AAA7455	Finland	University of Oulu
Enargia paleacea	MM01714	LEFIA602-10	HM870851	BOLD:AAA7455	Finland	University of Oulu
Enargia paleacea	TLMF Lep 12462	LEATC480-13	KM572897	BOLD:AAA7455	Austria	Tiroler Landesmuseum Ferdinandeum
Enargia paleacea	MM01715	LEFIA603-10	HM870852	BOLD:AAA7455	Finland	University of Oulu
Endothenia ericetana	MM17232	LEFIJ607-10	JF853707	BOLD:AAD7173	Finland	University of Oulu
Endothenia ericetana	MM15709	LEFIG845-10	HM876493	BOLD:AAD7173	Finland	University of Oulu
Endothenia ericetana	TLMF Lep 07521	PHLAG842-12	KM572039	BOLD:AAD7173	Austria	Tiroler Landesmuseum Ferdinandeum
Endothenia ericetana	MM13238	LEFIF865-10	HM875547	BOLD:AAD7173	Finland	University of Oulu
Endothenia ericetana	MM23221	COLFF947-13	KM572391	BOLD:AAD7173	Finland	University of Oulu
Endothenia marginana	MM15705	LEFIG841-10	HM876489	BOLD:AAC9535	Finland	University of Oulu
Endothenia marginana	MM17227	LEFIJ602-10	JF853702	BOLD:AAC9535	Finland	University of Oulu
Endothenia marginana	MM02152	LEFIB539-10	HM871425	BOLD:AAC9535	Finland	University of Oulu

Endothenia marginana	MM02151	LEFIB538-10	HM871424	BOLD:AAC9535	Finland	University of Oulu
Endothenia marginana	MM15702	LEFIG838-10	HM876486	BOLD:AAC9535	Finland	University of Oulu
Endothenia marginana	MM15703	LEFIG839-10	HM876487	BOLD:AAC9535	Finland	University of Oulu
Endothenia marginana	MM15704	LEFIG840-10	HM876488	BOLD:AAC9535	Finland	University of Oulu
Endothenia marginana	MM15706	LEFIG842-10	HM876490	BOLD:AAC9535	Finland	University of Oulu
Endothenia marginana	MM15707	LEFIG843-10	HM876491	BOLD:AAC9535	Finland	University of Oulu
Endothenia marginana	MM15708	LEFIG844-10	HM876492	BOLD:AAC9535	Finland	University of Oulu
Endothenia marginana	MM11827	LEFIF419-10	HM875104	BOLD:AAC9535	Finland	University of Oulu
Endothenia marginana	TLMF Lep 08048	PHLAV229-12	KM573398	BOLD:AAC9535	Austria	inatura, Dornbirn
Endothenia marginana	TLMF Lep 07516	PHLAG837-12	KM572475	BOLD:AAC9535	Austria	Tiroler Landesmuseum Ferdinandeum
Endothenia nigricostana	MM08698	LEFIE321-10	HM874047	BOLD:AAF0269	Finland	University of Oulu
Endothenia nigricostana	TLMF Lep 09779	LEATA362-13	KM573203	BOLD:AAF0269	Austria	Tiroler Landesmuseum Ferdinandeum
Endothenia nigricostana	MM05924	LEFID160-10	HM872967	BOLD:AAF0269	Finland	University of Oulu
Endothenia nigricostana	MM05923	LEFID159-10	HM872966	BOLD:AAF0269	Finland	University of Oulu
Endothenia quadrimaculana	MM02110	LEFIB514-10	HM871406	BOLD:AAD4973	Finland	University of Oulu
Endothenia quadrimaculana	MM13184	LEFIF839-10	HM875522	BOLD:AAD4973	Finland	University of Oulu
Endothenia quadrimaculana	TLMF Lep 08447	PHLAH628-12	KM573608	BOLD:AAD4973	Austria	inatura, Dornbirn
Endothenia quadrimaculana	MM02113	LEFIB515-10	HM871407	BOLD:AAD4973	Finland	University of Oulu
Endothenia quadrimaculana	MM04986	LEFIC810-10	HM872629	BOLD:AAD4973	Finland	University of Oulu
Endromis versicolora	MM12175	LEFIF530-10	HM875215	BOLD:AAD4912	Finland	University of Oulu
Endromis versicolora	MM02802	LEFIB837-10	HM871714	BOLD:AAD4912	Finland	University of Oulu
Endromis versicolora	MM00394	LEFIA1118-10	GU828640	BOLD:AAD4912	Finland	University of Oulu
Endromis versicolora	MM07742	LEFID944-10	HM873694	BOLD:AAD4912	Finland	University of Oulu
Endromis versicolora	TLMF Lep 09774	LEATA357-13	KM573595	BOLD:AAD4912	Austria	Tiroler Landesmuseum Ferdinandeum
Ennomos alniaria	TLMF Lep 08531	PHLAH712-12	KM572433	BOLD:AAB2314	Austria	inatura, Dornbirn
Ennomos alniaria	MM04510	LEFIC591-10	HM872412	BOLD:AAB2314	Finland	University of Oulu

Ennomos alniaria	MM12770	LEFIF717-10	HM875401	BOLD:AAB2314	Finland	University of Oulu
Ennomos alniaria	MM07824	LEFID952-10	HM873702	BOLD:AAB2314	Finland	University of Oulu
Entephria caesiata	TLMF Lep 07561	PHLAG882-12	KM572419	BOLD:AAB9028	Austria	inatura, Dornbirn
Entephria caesiata	MM02878	LEFIB857-10	HM871734	BOLD:AAB9028	Finland	University of Oulu
Entephria caesiata	MM00796	LEFIB292-10	HM871193	BOLD:AAB9028	Finland	University of Oulu
Entephria caesiata	MM08109	LEFIE033-10	HM873782	BOLD:AAB9028	Finland	University of Oulu
Entephria flavicinctata	MM10467	LEFIF028-10	HQ570380	BOLD:AAC5436	Finland	University of Oulu
Entephria flavicinctata	TLMF Lep 09502	LEATA085-13	KM572292	BOLD:AAC5436	Austria	Tiroler Landesmuseum Ferdinandeum
Entephria flavicinctata	MM10465	LEFIF026-10	HQ570378	BOLD:AAC5436	Finland	University of Oulu
Entephria flavicinctata	MM10466	LEFIF027-10	HQ570379	BOLD:AAC5436	Finland	University of Oulu
Entephria flavicinctata	TLMF Lep 12608	LEATC626-13	KM573046	BOLD:AAC5436	Austria	Tiroler Landesmuseum Ferdinandeum
Epermenia chaerophyllella	MM14007	LEFIG153-10	HM875833	BOLD:AAC9729	Finland	University of Oulu
Epermenia chaerophyllella	MM09752	LEFIA875-10	HM387013	BOLD:AAC9729	Finland	University of Oulu
Epermenia chaerophyllella	MM09795	LEFIA917-10	HM387053	BOLD:AAC9729	Finland	University of Oulu
Epermenia chaerophyllella	TLMF Lep 08211	PHLAH392-12	KM572603	BOLD:AAC9729	Austria	inatura, Dornbirn
Epermenia chaerophyllella	MM00443	LEFIA041-10	HM396390	BOLD:AAC9729	Finland	University of Oulu
Ephestia elutella	TLMF Lep 09910	PHLAW113-13	KM573100	BOLD:AAC6157	Austria	Tiroler Landesmuseum Ferdinandeum
Ephestia elutella	MM06861	LEFID790-10	HM873547	BOLD:AAC6157	Finland	University of Oulu
Ephestia elutella	MM10369	LEFIE963-10	HM874680	BOLD:AAC6157	Finland	University of Oulu
Ephestia elutella	MM10368	LEFIE962-10	HM874679	BOLD:AAC6157	Finland	University of Oulu
Epiblema grandaevana	TLMF Lep 08740	PHLAH936-12	KM572104	BOLD:AAF2217	Austria	Tiroler Landesmuseum Ferdinandeum
Epiblema grandaevana	MM08704	LEFIE325-10	HM874051	BOLD:AAF2218	Finland	University of Oulu
Epiblema grandaevana	MM12415	LEFIF587-10	HM875271	BOLD:AAF2218	Finland	University of Oulu
Epiblema grandaevana	MM14216	LEFIG275-10	HM875954	BOLD:AAF2218	Finland	University of Oulu
Epiblema grandaevana	MM22090	LEFIJ1398-12	KM573221	BOLD:AAF2218	Finland	University of Oulu
Epiblema grandaevana	MM22091	LEFIJ1399-12	KM572485	BOLD:AAF2218	Finland	University of Oulu
Epiblema grandaevana	MM22092	LEFIJ1400-12	KM572817	BOLD:AAF2218	Finland	University of Oulu
Epiblema scutulana	TLMF Lep 09192	PHLAI630-13	KM572646	BOLD:AAC0715	Austria	Tiroler Landesmuseum Ferdinandeum
Epiblema scutulana	MM09212	LEFIE448-10	HM874172	BOLD:AAC0715	Finland	University of Oulu
Epiblema scutulana	MM14105	LEFIG215-10	HM875895	BOLD:AAC0715	Finland	University of Oulu
Epiblema scutulana	MM14107	LEFIG217-10	HM875897	BOLD:AAC0715	Finland	University of Oulu

Epiblema scutulana	MM14106	LEFIG216-10	HM875896	BOLD:AAC0715	Finland	University of Oulu
Epiblema scutulana	MM14104	LEFIG214-10	HM875894	BOLD:AAC0715	Finland	University of Oulu
Epiblema scutulana	MM17266	LEFIJ641-10	JF853738	BOLD:AAP7460	Finland	University of Oulu
Epiblema scutulana	MM17267	LEFIJ642-10	JF853739	BOLD:AAP7460	Finland	University of Oulu
Epiblema sticticana	TLMF Lep 10054	LEATA447-13	KM573040	BOLD:AAC0719	Austria	inatura, Dornbirn
Epiblema sticticana	MM09989	LEFIE800-10	HM874519	BOLD:AAC0719	Finland	University of Oulu
Epiblema sticticana	MM15727	LEFIG863-10	HM876511	BOLD:AAC0719	Finland	University of Oulu
Epiblema sticticana	MM15726	LEFIG862-10	HM876510	BOLD:AAC0719	Finland	University of Oulu
Epiblema sticticana	MM10061	LEFIE842-10	HM874561	BOLD:AAC0719	Finland	University of Oulu
Epinotia abbreviana	MM05009	LEFIC824-10	HM872643	BOLD:AAE1784	Finland	University of Oulu
Epinotia abbreviana	TLMF Lep 08093	PHLAV274-12	KM572175	BOLD:AAE1784	Austria	inatura, Dornbirn
Epinotia abbreviana	MM09749	LEFIA872-10	HM387010	BOLD:AAE1784	Finland	University of Oulu
Epinotia abbreviana	MM09747	LEFIA870-10	HM387008	BOLD:AAE1784	Finland	University of Oulu
Epinotia abbreviana	MM09748	LEFIA871-10	HM387009	BOLD:AAE1784	Finland	University of Oulu
Epinotia bilunana	TLMF Lep 09931	PHLAW134-13	KM572890	BOLD:AAD1759	Austria	inatura, Dornbirn
Epinotia bilunana	MM15718	LEFIG854-10	HM876502	BOLD:AAD1759	Finland	University of Oulu
Epinotia bilunana	MM15717	LEFIG853-10	HM876501	BOLD:AAD1759	Finland	University of Oulu
Epinotia bilunana	MM15716	LEFIG852-10	HM876500	BOLD:AAD1759	Finland	University of Oulu
Epinotia cruciana	MM03323	LEFIC053-10	HM871922	BOLD:AAC2644	Finland	University of Oulu
Epinotia cruciana	MM08349	LEFIE133-10	HM873881	BOLD:AAC2644	Finland	University of Oulu
Epinotia cruciana	MM06544	LEFID555-10	HM873320	BOLD:AAC2644	Finland	University of Oulu
Epinotia cruciana	TLMF Lep 00798	PHLAA758-09	HM426142	BOLD:AAC2644	Austria	Tiroler Landesmuseum Ferdinandeum
Epinotia cruciana	MM06921	LEFID821-10	HM873578	BOLD:AAC2644	Finland	University of Oulu
Epinotia demarniana	TLMF Lep 09925	PHLAW128-13	KM573710	BOLD:ACJ0697	Austria	inatura, Dornbirn
Epinotia demarniana	MM00759	LEFIB275-10	HM871177	BOLD:ACJ0697	Finland	University of Oulu
Epinotia demarniana	MM02125	LEFIB522-10	HQ570270	BOLD:ACJ0697	Finland	University of Oulu
Epinotia demarniana	MM03202	LEFIC002-10	HM871872	BOLD:ACJ0697	Finland	University of Oulu
Epinotia demarniana	MM14227	LEFIG281-10	HM875960	BOLD:ACJ0697	Finland	University of Oulu
Epinotia granitana	MM15719	LEFIG855-10	HM876503	BOLD:AAJ0675	Finland	University of Oulu
Epinotia granitana	MM18325	LEFIK750-10	KM572612	BOLD:AAJ0675	Finland	University of Oulu
Epinotia granitana	MM18326	LEFIK751-10	JF854310	BOLD:AAJ0675	Finland	University of Oulu

Epinotia granitana	TLMF Lep 07490	PHLAG811-12	KM573480	BOLD:AAJ0675	Austria	inatura, Dornbirn
Epinotia granitana	MM21060	LEFIJ1200-11	KM572680	BOLD:AAJ0675	Finland	University of Oulu
Epinotia immundana	MM05463	LEFID028-10	HM872842	BOLD:AAC9104	Finland	University of Oulu
Epinotia immundana	MM10063	LEFIJ150-10	JF853463	BOLD:AAC9104	Finland	University of Oulu
Epinotia immundana	MM18330	LEFIK755-10	JF854313	BOLD:AAC9104	Finland	University of Oulu
Epinotia immundana	MM18329	LEFIK754-10	JF854312	BOLD:AAC9104	Finland	University of Oulu
Epinotia immundana	MM21198	LEFIJ958-11	KM573272	BOLD:AAC9104	Finland	University of Oulu
Epinotia immundana	MM11881	LEFIF436-10	HM875121	BOLD:AAC9106	Finland	University of Oulu
Epinotia immundana	TLMF Lep 07481	PHLAG802-12	KM573364	BOLD:AAC9106	Austria	inatura, Dornbirn
Epinotia immundana	MM02153	LEFIB540-10	HQ570273	BOLD:AAC9106	Finland	University of Oulu
Epinotia immundana	MM11865	LEFIF432-10	HM875117	BOLD:AAC9106	Finland	University of Oulu
Epinotia mercuriana	TLMF Lep 03671	PHLAD496-11	JN275016	BOLD:AAE1186	Austria	Tiroler Landesmuseum Ferdinandeum
Epinotia mercuriana	MM06048	LEFID217-10	HM873018	BOLD:AAE1187	Finland	University of Oulu
Epinotia mercuriana	MM18327	LEFIK752-10	JX034694	BOLD:AAE1187	Finland	University of Oulu
Epinotia mercuriana	MM14635	LEFIG522-10	HM876195	BOLD:AAE1187	Finland	University of Oulu
Epinotia nanana	MM10398	LEFIE972-10	HM874689	BOLD:AAA8628	Finland	University of Oulu
Epinotia nanana	BIOUG04118-D10	GMFIO711-13	KM573715	BOLD:AAA8628	Finland	Biodiversity Institute of Ontario
Epinotia nanana	TLMF Lep 08245	PHLAH426-12	KM573706	BOLD:AAA8628	Austria	inatura, Dornbirn
Epinotia nanana	BIOUG04116-A10	GMFIE830-12	KM572061	BOLD:AAA8628	Finland	Biodiversity Institute of Ontario
Epinotia nanana	MM00697	LEFIB235-10	HM871138	BOLD:AAA8628	Finland	University of Oulu
Epinotia nanana	MM10163	LEFIE885-10	HM874603	BOLD:AAA8628	Finland	University of Oulu
Epinotia nisella	MM12340	LEFIJ191-10	JQ775247	BOLD:AAA7530	Finland	University of Oulu
Epinotia nisella	MM12338	LEFIJ189-10	JQ775243	BOLD:AAA7530	Finland	University of Oulu
Epinotia nisella	MM12339	LEFIJ190-10	JQ775244	BOLD:AAA7530	Finland	University of Oulu
Epinotia nisella	MM13308	LEFIF896-10	HM875578	BOLD:AAA7530	Finland	University of Oulu
Epinotia nisella	MM08949	LEFIJ127-10	JQ775242	BOLD:AAA7530	Finland	University of Oulu
Epinotia nisella	MM11880	LEFIF435-10	HM875120	BOLD:AAA7530	Finland	University of Oulu
Epinotia nisella	MM00758	LEFIB274-10	HM871176	BOLD:AAA7530	Finland	University of Oulu
Epinotia nisella	MM18331	LEFIK756-10	JQ775240	BOLD:AAA7530	Finland	University of Oulu
Epinotia nisella	MM08948	LEFIJ126-10	JQ775241	BOLD:AAA7530	Finland	University of Oulu
Epinotia nisella	MM12341	LEFIJ192-10	JQ775248	BOLD:AAA7530	Finland	University of Oulu

Epinotia nisella	TLMF Lep 08463	PHLAH644-12	KM572327	BOLD:AAA7530	Austria	inatura, Dornbirn
Epinotia ramella	MM03027	LEFIJ052-10	KM572191	BOLD:AAC6083	Finland	University of Oulu
Epinotia ramella	MM00681	LEFIB226-10	HM871130	BOLD:AAC6083	Finland	University of Oulu
Epinotia ramella	MM08215	LEFIJ113-10	KM572372	BOLD:AAC6083	Finland	University of Oulu
Epinotia ramella	MM05020	LEFIC829-10	HM872648	BOLD:AAC6083	Finland	University of Oulu
Epinotia ramella	MM08933	LEFIE391-10	HM874115	BOLD:ACE7003	Finland	University of Oulu
Epinotia ramella	TLMF Lep 08748	PHLAH944-12	KM573023	BOLD:ACE9771	Austria	Tiroler Landesmuseum Ferdinandeum
Epinotia ramella	MM01966	LEFIJ037-10	KM572107	BOLD:ACE9771	Finland	University of Oulu
Epinotia subocellana	MM03832	LEFIC327-10	HM872171	BOLD:AAB8745	Finland	University of Oulu
Epinotia subocellana	MM14025	LEFIG165-10	HM875845	BOLD:AAB8745	Finland	University of Oulu
Epinotia subocellana	MM08585	LEFIE252-10	HM873986	BOLD:AAB8745	Finland	University of Oulu
Epinotia subocellana	TLMF Lep 09983	PHLAW186-13	KM572940	BOLD:AAB8745	Austria	inatura, Dornbirn
Epinotia tedella	MM02139	LEFIB528-10	HM871416	BOLD:AAD1782	Finland	University of Oulu
Epinotia tedella	TLMF Lep 07938	PHLAV119-12	KM572418	BOLD:AAD1782	Austria	inatura, Dornbirn
Epinotia tedella	MM13338	LEFIF913-10	HM875595	BOLD:AAD1782	Finland	University of Oulu
Epinotia tedella	MM05262	LEFIC919-10	HM872736	BOLD:AAD1782	Finland	University of Oulu
Epinotia tetraquetrana	MM00544	LEFIB154-10	HM871059	BOLD:AAD1775	Finland	University of Oulu
Epinotia tetraquetrana	MM15993	LEFIJ388-10	JF853575	BOLD:AAD1775	Finland	University of Oulu
Epinotia tetraquetrana	TLMF Lep 09201	PHLAI639-13	KM572675	BOLD:AAD1775	Austria	Tiroler Landesmuseum Ferdinandeum
Epinotia tetraquetrana	TLMF Lep 09167	PHLAI605-13	KM572472	BOLD:AAD1775	Austria	Tiroler Landesmuseum Ferdinandeum
Epinotia tetraquetrana	MM00543	LEFIB153-10	HM871058	BOLD:AAD1775	Finland	University of Oulu
Epinotia tetraquetrana	MM02102	LEFIB509-10	HM871403	BOLD:AAD1775	Finland	University of Oulu
Epinotia trigonella	MM02047	LEFIB481-10	HQ570263	BOLD:AAB2504	Finland	University of Oulu
Epinotia trigonella	MM04647	LEFIC679-10	HM872500	BOLD:AAB2504	Finland	University of Oulu
Epinotia trigonella	MM18321	LEFIK746-10	JF854306	BOLD:AAB2504	Finland	University of Oulu
Epinotia trigonella	MM13231	LEFIF861-10	HM875543	BOLD:AAB2504	Finland	University of Oulu
Epinotia trigonella	TLMF Lep 08744	PHLAH940-12	KM572657	BOLD:AAB2504	Austria	Tiroler Landesmuseum Ferdinandeum
Epinotia trigonella	MM12351	LEFIF573-10	HM875258	BOLD:AAB2504	Finland	University of Oulu
Epipsilia grisescens	MM15893	LEFIH029-10	HM876665	BOLD:AAF4729	Finland	University of Oulu
Epipsilia grisescens	MM15892	LEFIH028-10	HM876664	BOLD:AAF4729	Finland	University of Oulu
Epipsilia grisescens	MM03657	LEFIC247-10	HM872091	BOLD:AAF4729	Finland	University of Oulu

Epipsilia grisescens	TLMF Lep 06099	PHLSA644-11	KM573021	BOLD:AAF4729	Austria	Tiroler Landesmuseum Ferdinandeum
Epirrhoe alternata	MM11620	LEFIF377-10	HM875062	BOLD:ACF4363	Finland	University of Oulu
Epirrhoe alternata	MM04581	LEFIC638-10	HM872459	BOLD:ACF4363	Finland	University of Oulu
Epirrhoe alternata	MM23192	COLFF459-13	KM573339	BOLD:ACF4363	Finland	University of Oulu, Zoological Museum
Epirrhoe alternata	TLMF Lep 07852	PHLAV033-12	KM572809	BOLD:ACF4363	Austria	inatura, Dornbirn
Epirrhoe alternata	MM01342	LEFIA297-10	HM386640	BOLD:ACF4363	Finland	University of Oulu
Epirrhoe alternata	MM12834	LEFIF730-10	HM875414	BOLD:ACF4363	Finland	University of Oulu
Epirrhoe alternata	MM12244	LEFIF550-10	HM875235	BOLD:ACF4363	Finland	University of Oulu
Epirrhoe alternata	MM07844	LEFID953-10	HM873703	BOLD:ACF4363	Finland	University of Oulu
Epirrhoe alternata	MM00615	LEFIB188-10	HM871092	BOLD:ACF4363	Finland	University of Oulu
Epirrhoe alternata	MM01341	LEFIA296-10	HM386639	BOLD:ACF4363	Finland	University of Oulu
Epirrhoe galiata	TLMF Lep 07866	PHLAV047-12	KM573537	BOLD:AAB2097	Austria	inatura, Dornbirn
Epirrhoe galiata	MM06895	LEFID809-10	HM873566	BOLD:AAB2097	Finland	University of Oulu
Epirrhoe galiata	MM00339	LEFIB056-10	HM870965	BOLD:AAB2097	Finland	University of Oulu
Epirrhoe galiata	MM18424	LEFIK849-10	JN279409	BOLD:AAB2097	Finland	University of Oulu
Epirrhoe rivata	MM05919	LEFID156-10	HM872963	BOLD:AAD7207	Finland	University of Oulu
Epirrhoe rivata	TLMF Lep 09880	PHLAW083-13	KM573612	BOLD:AAD7207	Austria	Tiroler Landesmuseum Ferdinandeum
Epirrhoe rivata	MM18699	LEFIL401-10	KM572293	BOLD:AAD7207	Finland	University of Oulu
Epirrhoe rivata	MM06105	LEFID242-10	HM873041	BOLD:AAD7207	Finland	University of Oulu
Epirrhoe rivata	MM15909	LEFIH045-10	HM876676	BOLD:AAD7207	Finland	University of Oulu
Epirrhoe tristata	MM06810	LEFID749-10	HM873506	BOLD:AAD7202	Finland	University of Oulu
Epirrhoe tristata	MM09475	LEFIE578-10	HM874301	BOLD:AAD7202	Finland	University of Oulu
Epirrhoe tristata	MM01376	LEFIA329-10	HM386672	BOLD:AAD7202	Finland	University of Oulu
Epirrhoe tristata	MM06201	LEFID316-10	HM873113	BOLD:AAD7202	Finland	University of Oulu
Epirrhoe tristata	TLMF Lep 08548	PHLAH729-12	KM573158	BOLD:AAD7202	Austria	inatura, Dornbirn
Epirrita autumnata	TLMF Lep 09875	PHLAW078-13	KM573584	BOLD:AAA5906	Austria	Tiroler Landesmuseum Ferdinandeum
Epirrita autumnata	MM02687	LEFIJ045-10	KM573511	BOLD:AAA5906	Finland	University of Oulu
Epirrita autumnata	MM07901	LEFIJ108-10	KM572524	BOLD:AAA5906	Finland	University of Oulu
Epirrita autumnata	MM04509	LEFIC590-10	HM872411	BOLD:AAA5906	Finland	University of Oulu
Epirrita autumnata	MM02686	LEFIB784-10	HM871661	BOLD:AAA5906	Finland	University of Oulu

Epirrita autumnata	MM18447	LEFIK872-10	JF854330	BOLD:AAA5906	Finland	University of Oulu
Epirrita autumnata	MM12813	LEFIJ198-10	KM572806	BOLD:AAA5906	Finland	University of Oulu
Epirrita autumnata	TLMF Lep 06129	PHLSA674-11	KM572134	BOLD:AAA5907	Austria	Tiroler Landesmuseum Ferdinandeum
Epirrita autumnata	MM00808	LEFIJ031-10	KM573210	BOLD:ACE7803	Finland	University of Oulu
Epirrita autumnata	MM00807	LEFIB297-10	HM871198	BOLD:ACE7803	Finland	University of Oulu
Epirrita christyi	MM14724	LEFIG570-10	HM876241	BOLD:AAB0936	Finland	University of Oulu
Epirrita christyi	MM14725	LEFIG571-10	HM876242	BOLD:AAB0936	Finland	University of Oulu
Epirrita christyi	MM14730	LEFIG574-10	HM876245	BOLD:AAB0936	Finland	University of Oulu
Epirrita christyi	MM03190	LEFIB993-10	HM871865	BOLD:AAB0936	Finland	University of Oulu
Epirrita christyi	TLMF Lep 06128	PHLSA673-11	KM572911	BOLD:AAB0936	Austria	Tiroler Landesmuseum Ferdinandeum
Erannis defoliaria	MM18515	LEFIK940-10	JF854380	BOLD:AAB4418	Finland	University of Oulu
Erannis defoliaria	MM14438	LEFIG412-10	HM876088	BOLD:AAB4418	Finland	University of Oulu
Erannis defoliaria	MM12237	LEFIF548-10	HM875233	BOLD:AAB4418	Finland	University of Oulu
Erannis defoliaria	TLMF Lep 06800	PHLAG406-12	KM572172	BOLD:AAB4418	Austria	Tiroler Landesmuseum Ferdinandeum
Erebia ligea	MM03273	LEFIC042-10	HM871912	BOLD:AAA8264	Finland	University of Oulu
Erebia ligea	MM17874	LEFIK299-10	JF853953	BOLD:AAA8264	Finland	University of Oulu
Erebia ligea	MM05734	LEFID111-10	HM872922	BOLD:AAA8264	Finland	University of Oulu
Erebia ligea	MM03272	LEFIC041-10	HM871911	BOLD:AAA8264	Finland	University of Oulu
Erebia ligea	MM14658	LEFIG532-10	HM876205	BOLD:AAA8264	Finland	University of Oulu
Erebia ligea	MM14657	LEFIG531-10	HM876204	BOLD:AAA8264	Finland	University of Oulu
Erebia ligea	MM05735	LEFID112-10	HM872923	BOLD:AAA8264	Finland	University of Oulu
Erebia ligea	TLMF Lep 08564	PHLAH745-12	KM572388	BOLD:AAA8264	Austria	inatura, Dornbirn
Erebia pandrose	MM14572	LEFIG496-10	HM876171	BOLD:AAB3454	Finland	University of Oulu
Erebia pandrose	MM00091	LEFIA020-10	HM396370	BOLD:AAB3454	Finland	University of Oulu
Erebia pandrose	TLMF Lep 09813	PHLAW016-13	KM573540	BOLD:AAB3454	Austria	Tiroler Landesmuseum Ferdinandeum
Erebia pandrose	MM06305	LEFID392-10	HM873189	BOLD:AAB3454	Finland	University of Oulu
Eriocrania sangii	MM05315	LEFIC947-10	HM872762	BOLD:AAB3766	Finland	University of Oulu
Eriocrania sangii	MM08793	LEFIE354-10	HM874078	BOLD:AAB3766	Finland	University of Oulu
Eriocrania sangii	MM14777	LEFIG588-10	HM876259	BOLD:AAB3766	Finland	University of Oulu
Eriocrania sangii	MM19758	LEEUA350-11	KM573684	BOLD:AAB3766	Finland	University of Oulu
Eriocrania sangii	MM21223	LEFIJ983-11	KM573159	BOLD:AAB3766	Finland	University of Oulu

Eriocrania sangii	MM19767	LEEUA359-11	JN303420	BOLD:AAB3766	Finland	University of Oulu
Eriocrania sangii	MM00391	LEFIB087-10	HM870995	BOLD:AAB3766	Finland	University of Oulu
Eriocrania sangii	MM19759	LEEUA351-11	JN303414	BOLD:AAB3766	Finland	University of Oulu
Eriocrania sangii	TLMF Lep 02973	PHLAC938-10	JF860406	BOLD:AAB3766	Austria	Tiroler Landesmuseum Ferdinandeum
Eriocrania sangii	MM17912	LEFIK337-10	JF853978	BOLD:AAB3766	Finland	University of Oulu
Eriocrania sangii	MM19760	LEEUA352-11	JN303415	BOLD:AAB3766	Finland	University of Oulu
Eriocrania sangii	MM08795	LEFIE356-10	HM874080	BOLD:AAB3766	Finland	University of Oulu
Eriocrania sangii	MM08799	LEFIE360-10	HM874084	BOLD:AAB3766	Finland	University of Oulu
Eriocrania sangii	MM12061	LEFIF495-10	HM875180	BOLD:AAB3766	Finland	University of Oulu
Eriocrania sangii	MM05750	LEFIJ087-10	KM572683	BOLD:AAB3766	Finland	University of Oulu
Eriocrania sangii	MM19761	LEEUA353-11	JN303416	BOLD:AAB3766	Finland	University of Oulu
Eriocrania sangii	MM00431	LEFIA032-10	HM396382	BOLD:AAB3766	Finland	University of Oulu
Eriocrania sangii	MM05747	LEFIJ084-10	JF853435	BOLD:AAB3766	Finland	University of Oulu
Eriocrania sangii	MM17954	LEFIK379-10	JF854012	BOLD:AAB3766	Finland	University of Oulu
Eriocrania sangii	MM17929	LEFIK354-10	JF853990	BOLD:AAB3766	Finland	University of Oulu
Eriocrania sangii	MM17927	LEFIK352-10	JF853988	BOLD:AAB3766	Finland	University of Oulu
Eriocrania sangii	MM17926	LEFIK351-10	JF853987	BOLD:AAB3766	Finland	University of Oulu
Eriocrania sangii	MM19766	LEEUA358-11	JN303419	BOLD:AAB3766	Finland	University of Oulu
Eriocrania sangii	MM17924	LEFIK349-10	JF853985	BOLD:AAB3766	Finland	University of Oulu
Eriocrania sangii	MM05316	LEFIC948-10	HM872763	BOLD:AAB3766	Finland	University of Oulu
Eriocrania semipurpurella	MM21225	LEFIJ985-11	KM573632	BOLD:AAB3764	Finland	University of Oulu
Eriocrania semipurpurella	MM21224	LEFIJ984-11	KM572682	BOLD:AAB3764	Finland	University of Oulu
Eriocrania semipurpurella	MM21221	LEFIJ981-11	KM572112	BOLD:AAB3764	Finland	University of Oulu
Eriocrania semipurpurella	MM21220	LEFIJ980-11	KM573262	BOLD:AAB3764	Finland	University of Oulu
Eriocrania semipurpurella	MM21219	LEFIJ979-11	KM572143	BOLD:AAB3764	Finland	University of Oulu
Eriocrania semipurpurella	MM21218	LEFIJ978-11	KM573552	BOLD:AAB3764	Finland	University of Oulu
Eriocrania semipurpurella	MM00405	LEFIB096-10	HM871003	BOLD:AAB3764	Finland	University of Oulu
Eriocrania semipurpurella	MM00404	LEFIB095-10	HM871002	BOLD:AAB3764	Finland	University of Oulu
Eriocrania semipurpurella	MM14779	LEFIG590-10	HM876261	BOLD:AAB3764	Finland	University of Oulu
Eriocrania semipurpurella	MM14780	LEFIG591-10	HM876262	BOLD:AAB3764	Finland	University of Oulu
Eriocrania semipurpurella	MM00432	LEFIA033-10	HM396383	BOLD:AAB3764	Finland	University of Oulu

Eriocrania semipurpurella	MM00430	LEFIA031-10	HM396381	BOLD:AAB3764	Finland	University of Oulu
Eriocrania semipurpurella	MM00429	LEFIA030-10	HM396380	BOLD:AAB3764	Finland	University of Oulu
Eriocrania semipurpurella	MM17955	LEFIK380-10	JF854013	BOLD:AAB3764	Finland	University of Oulu
Eriocrania semipurpurella	MM17928	LEFIK353-10	JF853989	BOLD:AAB3764	Finland	University of Oulu
Eriocrania semipurpurella	MM17925	LEFIK350-10	JF853986	BOLD:AAB3764	Finland	University of Oulu
Eriocrania semipurpurella	MM17922	LEFIK347-10	JF853984	BOLD:AAB3764	Finland	University of Oulu
Eriocrania semipurpurella	MM17910	LEFIK335-10	KM573312	BOLD:AAB3764	Finland	University of Oulu
Eriocrania semipurpurella	MM17909	LEFIK334-10	JF853976	BOLD:AAB3764	Finland	University of Oulu
Eriocrania semipurpurella	MM05748	LEFIJ085-10	KM573522	BOLD:AAB3764	Finland	University of Oulu
Eriocrania semipurpurella	MM13127	LEFIJ225-10	JF853481	BOLD:AAB3764	Finland	University of Oulu
Eriocrania semipurpurella	MM13119	LEFIJ218-10	KM573315	BOLD:AAB3764	Finland	University of Oulu
Eriocrania semipurpurella	MM13117	LEFIJ216-10	KM572084	BOLD:AAB3764	Finland	University of Oulu
Eriocrania semipurpurella	MM13111	LEFIJ210-10	KM572778	BOLD:AAB3764	Finland	University of Oulu
Eriocrania semipurpurella	MM13108	LEFIJ207-10	KM572857	BOLD:AAB3764	Finland	University of Oulu
Eriocrania semipurpurella	MM16677	LEFIJ1091-11	KM571955	BOLD:AAB3764	Finland	University of Oulu
Eriocrania semipurpurella	MM16676	LEFIJ1090-11	KM572152	BOLD:AAB3764	Finland	University of Oulu
Eriocrania semipurpurella	MM00403	LEFIB094-10	HM871001	BOLD:AAB3764	Finland	University of Oulu
Eriocrania semipurpurella	MM00414	LEFIB102-10	HM871009	BOLD:AAB3764	Finland	University of Oulu
Eriocrania semipurpurella	MM08794	LEFIE355-10	HM874079	BOLD:AAB3764	Finland	University of Oulu
Eriocrania semipurpurella	MM17923	LEFIK348-10	KM572350	BOLD:AAB3764	Finland	University of Oulu
Eriocrania semipurpurella	MM13116	LEFIJ215-10	JF853480	BOLD:AAB3764	Finland	University of Oulu
Eriocrania semipurpurella	MM19825	LEEUA417-11	KM572156	BOLD:AAB3764	Finland	University of Oulu
Eriocrania semipurpurella	MM13106	LEFIJ205-10	KM572066	BOLD:AAB3764	Finland	University of Oulu
Eriocrania semipurpurella	MM00392	LEFIB088-10	HM870996	BOLD:AAB3764	Finland	University of Oulu
Eriocrania semipurpurella	MM13112	LEFIJ211-10	KM572757	BOLD:AAB3764	Finland	University of Oulu
Eriocrania semipurpurella	MM08797	LEFIE358-10	HM874082	BOLD:AAB3764	Finland	University of Oulu
Eriocrania semipurpurella	MM19764	LEEUA356-11	JN303417	BOLD:AAB3767	Finland	University of Oulu
Eriocrania semipurpurella	TLMF Lep 02954	PHLAC919-10	JF860390	BOLD:AAB3767	Austria	Tiroler Landesmuseum Ferdinandeum
Eriocrania semipurpurella	MM21222	LEFIJ982-11	KM573079	BOLD:AAB3767	Finland	University of Oulu
Eriocrania semipurpurella	MM14778	LEFIG589-10	HM876260	BOLD:AAB3767	Finland	University of Oulu
Eriocrania semipurpurella	MM14781	LEFIG592-10	HM876263	BOLD:AAB3767	Finland	University of Oulu

Eriocrania semipurpurella	MM08796	LEFIE357-10	HM874081	BOLD:AAB3767	Finland	University of Oulu
Eriocrania semipurpurella	MM12060	LEFIF494-10	HM875179	BOLD:AAB3767	Finland	University of Oulu
Eriocrania semipurpurella	MM05319	LEFIC949-10	HM872764	BOLD:AAB3767	Finland	University of Oulu
Eriocrania semipurpurella	MM05320	LEFIC950-10	HM872765	BOLD:AAB3767	Finland	University of Oulu
Eriocrania semipurpurella	MM05321	LEFIC951-10	HM872766	BOLD:AAB3767	Finland	University of Oulu
Eriocrania semipurpurella	MM00390	LEFIA1128-10	GU828639	BOLD:AAB3767	Finland	University of Oulu
Eriocrania semipurpurella	MM00434	LEFIA035-10	HM396385	BOLD:AAB3767	Finland	University of Oulu
Eriocrania semipurpurella	MM05746	LEFIJ083-10	JF853434	BOLD:AAB3767	Finland	University of Oulu
Eriocrania semipurpurella	MM05745	LEFIJ082-10	JF853433	BOLD:AAB3767	Finland	University of Oulu
Eriocrania semipurpurella	MM05742	LEFIJ079-10	JF853430	BOLD:AAB3767	Finland	University of Oulu
Eriocrania semipurpurella	MM05749	LEFIJ086-10	KM572138	BOLD:AAB3767	Finland	University of Oulu
Eriocrania semipurpurella	MM13130	LEFIJ228-10	KM573481	BOLD:AAB3767	Finland	University of Oulu
Eriocrania semipurpurella	MM13129	LEFIJ227-10	KM571987	BOLD:AAB3767	Finland	University of Oulu
Eriocrania semipurpurella	MM13128	LEFIJ226-10	KM572741	BOLD:AAB3767	Finland	University of Oulu
Eriocrania semipurpurella	MM13126	LEFIJ224-10	KM572366	BOLD:AAB3767	Finland	University of Oulu
Eriocrania semipurpurella	MM13125	LEFIJ223-10	KM572864	BOLD:AAB3767	Finland	University of Oulu
Eriocrania semipurpurella	MM13124	LEFIJ222-10	KM572436	BOLD:AAB3767	Finland	University of Oulu
Eriocrania semipurpurella	MM13123	LEFIJ221-10	KM571980	BOLD:AAB3767	Finland	University of Oulu
Eriocrania semipurpurella	MM13122	LEFIJ220-10	KM573318	BOLD:AAB3767	Finland	University of Oulu
Eriocrania semipurpurella	MM13121	LEFIJ219-10	KM572726	BOLD:AAB3767	Finland	University of Oulu
Eriocrania semipurpurella	MM13118	LEFIJ217-10	KM573353	BOLD:AAB3767	Finland	University of Oulu
Eriocrania semipurpurella	MM13113	LEFIJ212-10	KM573192	BOLD:AAB3767	Finland	University of Oulu
Eriocrania semipurpurella	MM13109	LEFIJ208-10	JF853476	BOLD:AAB3767	Finland	University of Oulu
Eriocrania semipurpurella	MM13107	LEFIJ206-10	KM573239	BOLD:AAB3767	Finland	University of Oulu
Eriocrania semipurpurella	MM13105	LEFIJ204-10	KM573305	BOLD:AAB3767	Finland	University of Oulu
Eriocrania semipurpurella	MM19838	LEEUA430-11	KM573441	BOLD:AAB3767	Finland	University of Oulu
Eriocrania semipurpurella	MM19837	LEEUA429-11	JN303433	BOLD:AAB3767	Finland	University of Oulu
Eriocrania semipurpurella	MM19831	LEEUA423-11	KM572662	BOLD:AAB3767	Finland	University of Oulu
Eriocrania semipurpurella	MM19827	LEEUA419-11	JN303429	BOLD:AAB3767	Finland	University of Oulu
Eriocrania semipurpurella	MM19826	LEEUA418-11	KM573284	BOLD:AAB3767	Finland	University of Oulu
Eriocrania semipurpurella	MM19765	LEEUA357-11	JN303418	BOLD:AAB3767	Finland	University of Oulu

Eriocrania semipurpurella	MM19757	LEEUA349-11	JN303413	BOLD:AAB3767	Finland	University of Oulu
Eriocrania semipurpurella	MM19756	LEEUA348-11	JN303412	BOLD:AAB3767	Finland	University of Oulu
Eriocrania semipurpurella	MM19754	LEEUA346-11	JN303410	BOLD:AAB3767	Finland	University of Oulu
Eriocrania semipurpurella	MM19752	LEEUA344-11	JN303408	BOLD:AAB3767	Finland	University of Oulu
Eriocrania semipurpurella	MM19750	LEEUA342-11	JN303406	BOLD:AAB3767	Finland	University of Oulu
Eriocrania semipurpurella	MM05323	LEFIC952-10	HM872767	BOLD:AAB3767	Finland	University of Oulu
Eriocrania semipurpurella	MM19751	LEEUA343-11	JN303407	BOLD:AAB3767	Finland	University of Oulu
Eriocrania semipurpurella	MM19773	LEEUA365-11	JN303425	BOLD:AAB3768	Finland	University of Oulu
Eriocrania semipurpurella	MM19755	LEEUA347-11	JN303411	BOLD:AAB3768	Finland	University of Oulu
Eriocrania semipurpurella	MM13110	LEFIJ209-10	JF853477	BOLD:AAB3768	Finland	University of Oulu
Eriocrania semipurpurella	MM19835	LEEUA427-11	KM572490	BOLD:AAB3768	Finland	University of Oulu
Eriocrania semipurpurella	MM19833	LEEUA425-11	JN303432	BOLD:AAB3768	Finland	University of Oulu
Eriocrania semipurpurella	MM19830	LEEUA422-11	JN303431	BOLD:AAB3768	Finland	University of Oulu
Eriocrania semipurpurella	MM19828	LEEUA420-11	JN303430	BOLD:AAB3768	Finland	University of Oulu
Eriocrania semipurpurella	MM19776	LEEUA368-11	JN303427	BOLD:AAB3768	Finland	University of Oulu
Eriocrania semipurpurella	MM19771	LEEUA363-11	JN303424	BOLD:AAB3768	Finland	University of Oulu
Eriocrania semipurpurella	MM19770	LEEUA362-11	JN303423	BOLD:AAB3768	Finland	University of Oulu
Eriocrania semipurpurella	MM19769	LEEUA361-11	JN303422	BOLD:AAB3768	Finland	University of Oulu
Eriocrania semipurpurella	MM19768	LEEUA360-11	JN303421	BOLD:AAB3768	Finland	University of Oulu
Eriocrania semipurpurella	MM19753	LEEUA345-11	JN303409	BOLD:AAB3768	Finland	University of Oulu
Eriocrania semipurpurella	MM16679	LEFIJ1093-11	KM573200	BOLD:AAB3768	Finland	University of Oulu
Eriocrania semipurpurella	MM16675	LEFIJ1089-11	KM572323	BOLD:AAB3768	Finland	University of Oulu
Eriocrania semipurpurella	MM09028	LEFIE421-10	HM874145	BOLD:AAB3768	Finland	University of Oulu
Eriocrania semipurpurella	MM05324	LEFIC953-10	HM872768	BOLD:AAB3768	Finland	University of Oulu
Eriocrania semipurpurella	MM19775	LEEUA367-11	JN303426	BOLD:AAB3768	Finland	University of Oulu
Eriopsela quadrana	MM04122	LEFIA768-10	HM386911	BOLD:AAD4787	Finland	University of Oulu
Eriopsela quadrana	MM00507	LEFIB136-10	HM871042	BOLD:AAD4787	Finland	University of Oulu
Eriopsela quadrana	TLMF Lep 00868	PHLAB068-10	HQ968242	BOLD:AAD4787	Austria	Tiroler Landesmuseum Ferdinandeum
Eriopsela quadrana	MM04123	LEFIA769-10	HM386912	BOLD:AAD4787	Finland	University of Oulu
Ethmia bipunctella	MM12518	LEFIF628-10	HM875312	BOLD:AAB2343	Finland	University of Oulu
Ethmia bipunctella	MM13188	LEFIF843-10	HM875526	BOLD:AAB2343	Finland	University of Oulu

Ethmia bipunctella	TLMF Lep 09932	PHLAW135-13	KM573188	BOLD:AAB2343	Austria	inatura, Dornbirn
Ethmia bipunctella	MM05918	LEFID155-10	HM872962	BOLD:AAB2343	Finland	University of Oulu
Ethmia pusiella	MM05432	LEFID010-10	HM872824	BOLD:AAE4537	Finland	University of Oulu
Ethmia pusiella	MM01224	LEFIA201-10	HM396546	BOLD:AAE4537	Finland	University of Oulu
Ethmia pusiella	TLMF Lep 08402	PHLAH583-12	KM572295	BOLD:AAE4537	Austria	inatura, Dornbirn
Ethmia pusiella	MM00042	LEFIA1129-10	GU828588	BOLD:AAE4537	Finland	University of Oulu
Ethmia pusiella	MM01223	LEFIA200-10	HM396545	BOLD:AAE4537	Finland	University of Oulu
Euchalcia variabilis	TLMF Lep 00307	PHLAA267-09	HM425804	BOLD:AAE1616	Austria	Tiroler Landesmuseum Ferdinandeum
Euchalcia variabilis	MM15933	LEFIJ333-10	KM573352	BOLD:AAP7449	Finland	University of Oulu
Euchalcia variabilis	MM15934	LEFIJ334-10	JF853551	BOLD:AAP7449	Finland	University of Oulu
Euchoeca nebulata	TLMF Lep 07895	PHLAV076-12	KM572981	BOLD:AAD0146	Austria	inatura, Dornbirn
Euchoeca nebulata	MM01443	LEFIA386-10	HM386728	BOLD:AAD0146	Finland	University of Oulu
Euchoeca nebulata	MM01316	LEFIA276-10	HM386620	BOLD:AAD0146	Finland	University of Oulu
Euchoeca nebulata	MM01317	LEFIA277-10	HM386621	BOLD:AAD0146	Finland	University of Oulu
Euclidia glyphica	MM03193	LEFIB995-10	HM871866	BOLD:AAD0810	Finland	University of Oulu
Euclidia glyphica	MM14858	LEFIG635-10	HM876301	BOLD:AAD0810	Finland	University of Oulu
Euclidia glyphica	TLMF Lep 08115	PHLAV296-12	KM572216	BOLD:AAD0810	Austria	inatura, Dornbirn
Euclidia glyphica	MM02785	LEFIB830-10	HM871707	BOLD:AAD0810	Finland	University of Oulu
Euclidia mi	MM00361	LEFIB071-10	HM870980	BOLD:AAD6424	Finland	University of Oulu
Euclidia mi	TLMF Lep 09846	PHLAW049-13	KM572992	BOLD:AAD6424	Austria	Tiroler Landesmuseum Ferdinandeum
Euclidia mi	MM00025	LEFIB011-10	HM870924	BOLD:AAD6424	Finland	University of Oulu
Euclidia mi	MM05469	LEFIA1033-10	KM572802	BOLD:AAD6424	Finland	University of Oulu
Euclidia mi	MM00026	LEFIB012-10	HM870925	BOLD:AAD6424	Finland	University of Oulu
Eucosma cana	MM08961	LEFIE400-10	HM874124	BOLD:AAB4296	Finland	University of Oulu
Eucosma cana	MM03064	LEFIB924-10	HM871801	BOLD:AAB4296	Finland	University of Oulu
Eucosma cana	MM05258	LEFIC917-10	HM872734	BOLD:AAB4296	Finland	University of Oulu
Eucosma cana	MM13186	LEFIF841-10	HM875524	BOLD:AAB4296	Finland	University of Oulu
Eucosma cana	MM11025	LEFIF244-10	HM874937	BOLD:AAB4296	Finland	University of Oulu
Eucosma cana	MM11834	LEFIF421-10	HM875106	BOLD:AAB4296	Finland	University of Oulu
Eucosma cana	TLMF Lep 08460	PHLAH641-12	KM573404	BOLD:AAB4296	Austria	inatura, Dornbirn
Eucosma cana	MM02107	LEFIB513-10	HM871405	BOLD:AAB4296	Finland	University of Oulu

Eucosma cana	MM06661	LEFID637-10	HM873400	BOLD:AAB4296	Finland	University of Oulu
Eucosma cana	MM08963	LEFIE401-10	HM874125	BOLD:AAB4296	Finland	University of Oulu
Eucosma conterminana	TLMF Lep 08443	PHLAH624-12	KM571959	BOLD:AAC3321	Austria	inatura, Dornbirn
Eucosma conterminana	MM04346	LEFIC568-10	HM872389	BOLD:AAC3321	Finland	University of Oulu
Eucosma conterminana	MM09536	LEFIE617-10	HM874340	BOLD:AAC3321	Finland	University of Oulu
Eucosma conterminana	MM22624	LEFIJ1608-12	KM572413	BOLD:AAC3321	Finland	University of Helsinki
Eucosma conterminana	MM06887	LEFID803-10	HM873560	BOLD:AAC3321	Finland	University of Oulu
Eucosma hohenwartiana	MM09283	LEFIJ1012-11	KM572508	BOLD:AAB4295	Finland	University of Oulu
Eucosma hohenwartiana	MM09282	LEFIJ1011-11	KM572050	BOLD:AAB4295	Finland	University of Oulu
Eucosma hohenwartiana	MM21209	LEFIJ969-11	KM573028	BOLD:AAB4295	Finland	University of Oulu
Eucosma hohenwartiana	TLMF Lep 08036	PHLAV217-12	KM572265	BOLD:AAB4295	Austria	inatura, Dornbirn
Eucosma hohenwartiana	MM09439	LEFIE554-10	HM874277	BOLD:AAB4295	Finland	University of Oulu
Eucosma hohenwartiana	MM12143	LEFIF516-10	HM875201	BOLD:AAB4295	Finland	University of Oulu
Eucosma hohenwartiana	MM11020	LEFIF239-10	HM874932	BOLD:AAB4295	Finland	University of Oulu
Eucosma hohenwartiana	MM11022	LEFIF241-10	HM874934	BOLD:AAB4295	Finland	University of Oulu
Eucosma hohenwartiana	MM13764	LEFIG094-10	HM875773	BOLD:AAB4295	Finland	University of Oulu
Eucosma hohenwartiana	MM21163	LEFIJ1303-11	KM572677	BOLD:AAB4295	Finland	University of Oulu
Eucosma hohenwartiana	MM14344	LEFIG349-10	HM876026	BOLD:AAB4295	Finland	University of Oulu
Eucosma hohenwartiana	MM06899	LEFID812-10	HM873569	BOLD:AAB4295	Finland	University of Oulu
Eucosma hohenwartiana	MM14501	LEFIG460-10	HM876136	BOLD:AAB4295	Finland	University of Oulu
Eucosma hohenwartiana	MM12320	LEFIF566-10	HM875251	BOLD:AAB4295	Finland	University of Oulu
Eucosma hohenwartiana	MM09451	LEFIE563-10	HM874286	BOLD:AAB4295	Finland	University of Oulu
Eucosma hohenwartiana	MM17239	LEFIJ614-10	JF853714	BOLD:AAB4295	Finland	University of Oulu
Eucosma hohenwartiana	MM22622	LEFIJ1606-12	KM572351	BOLD:AAB4295	Finland	University of Helsinki
Eucosma hohenwartiana	MM14502	LEFIG461-10	HM876137	BOLD:AAB4295	Finland	University of Oulu
Eucosma hohenwartiana	TLMF Lep 08461	PHLAH642-12	KM573354	BOLD:AAB4295	Austria	inatura, Dornbirn
Eucosma hohenwartiana	MM17240	LEFIJ615-10	JF853715	BOLD:AAB4295	Finland	University of Oulu
Eucosma hohenwartiana	MM06631	LEFID616-10	HM873381	BOLD:AAB4295	Finland	University of Oulu
Eucosma hohenwartiana	MM06630	LEFID615-10	HM873380	BOLD:AAB4295	Finland	University of Oulu
Eucosma hohenwartiana	MM21162	LEFIJ1302-11	KM572863	BOLD:AAB4295	Finland	University of Oulu
Eucosma hohenwartiana	MM09216	LEFIE452-10	HM874176	BOLD:AAB4295	Finland	University of Oulu

Eucosma hohenwartiana	MM09215	LEFIE451-10	HM874175	BOLD:AAB4295	Finland	University of Oulu
Eucosma hohenwartiana	MM22621	LEFIJ1605-12	KM572998	BOLD:AAB4295	Finland	University of Helsinki
Eucosma hohenwartiana	MM08964	LEFIE402-10	HM874126	BOLD:AAB4295	Finland	University of Oulu
Eucosma hohenwartiana	MM14505	LEFIG464-10	HM876140	BOLD:AAB4295	Finland	University of Oulu
Eucosma hohenwartiana	MM14504	LEFIG463-10	HM876139	BOLD:AAB4295	Finland	University of Oulu
Eucosma hohenwartiana	MM22623	LEFIJ1607-12	KM573547	BOLD:AAB4295	Finland	University of Helsinki
Eucosma hohenwartiana	MM14503	LEFIG462-10	HM876138	BOLD:AAB4295	Finland	University of Oulu
Eucosma hohenwartiana	MM21161	LEFIJ1301-11	KM572885	BOLD:AAB4295	Finland	University of Oulu
Eucosmomorpha albersana	TLMF Lep 07936	PHLAV117-12	KM572023	BOLD:AAF2360	Austria	inatura, Dornbirn
Eucosmomorpha albersana	MM17939	LEFIK364-10	JF853998	BOLD:AAF2360	Finland	University of Oulu
Eucosmomorpha albersana	MM09210	LEFIE446-10	HM874170	BOLD:AAF2360	Finland	University of Oulu
Eucosmomorpha albersana	MM14257	LEFIG299-10	HM875978	BOLD:AAF2360	Finland	University of Oulu
Eudemis porphyrana	MM05198	LEFIC893-10	HM872710	BOLD:AAC6854	Finland	University of Oulu
Eudemis porphyrana	MM02056	LEFIB486-10	HM871381	BOLD:AAC6854	Finland	University of Oulu
Eudemis porphyrana	MM08923	LEFIE385-10	HM874109	BOLD:AAC6854	Finland	University of Oulu
Eudemis porphyrana	TLMF Lep 08250	PHLAH431-12	KM573565	BOLD:AAC6854	Austria	inatura, Dornbirn
Eudonia lacustrata	MM02406	LEFIB643-10	HM871522	BOLD:AAB3829	Finland	University of Oulu
Eudonia lacustrata	TLMF Lep 10037	LEATA430-13	KM572324	BOLD:AAB3829	Austria	inatura, Dornbirn
Eudonia lacustrata	MM04969	LEFIC804-10	HM872623	BOLD:AAB3829	Finland	University of Oulu
Eudonia lacustrata	CNCLEP00020419	LNEL223-06	KM573199	BOLD:AAB3829	Finland	Canadian National Collection of Insects, Arachnids and Nematodes
Eudonia laetella	TLMF Lep 00758	PHLAA718-09	HM426112	BOLD:AAE4544	Austria	Tiroler Landesmuseum Ferdinandeum
Eudonia laetella	MM21109	LEFIJ1249-11	KM573604	BOLD:AAE4544	Finland	University of Oulu
Eudonia laetella	MM13363	LEFIF924-10	HM875606	BOLD:AAE4544	Finland	University of Oulu
Eudonia laetella	MM17223	LEFIJ598-10	JF853698	BOLD:AAE4544	Finland	University of Oulu
Eudonia laetella	MM17224	LEFIJ599-10	JF853699	BOLD:AAE4544	Finland	University of Oulu
Eudonia mercurella	MM11774	LEFIF405-10	HM875090	BOLD:AAB3830	Finland	University of Oulu
Eudonia mercurella	MM03833	LEFIC328-10	HM872172	BOLD:AAB3830	Finland	University of Oulu

Eudonia mercurella	MM11768	LEFIF403-10	HM875088	BOLD:AAB3830	Finland	University of Oulu
Eudonia mercurella	TLMF Lep 08477	PHLAH658-12	KM573012	BOLD:AAB3830	Austria	inatura, Dornbirn
Eudonia mercurella	MM13360	LEFIF922-10	HM875604	BOLD:AAB3830	Finland	University of Oulu
Eudonia mercurella	MM06660	LEFID636-10	HM873399	BOLD:AAB3830	Finland	University of Oulu
Eudonia mercurella	MM18389	LEFIK814-10	JN279061	BOLD:ABZ6210	Finland	University of Oulu
Eudonia murana	MM21211	LEFIJ971-11	KM572591	BOLD:AAB9086	Finland	University of Oulu
Eudonia murana	MM04134	LEFIA780-10	HM386921	BOLD:AAB9086	Finland	University of Oulu
Eudonia murana	MM21210	LEFIJ970-11	KM573394	BOLD:AAB9086	Finland	University of Oulu
Eudonia murana	MM02954	LEFIB881-10	HM871758	BOLD:AAB9086	Finland	University of Oulu
Eudonia murana	MM18390	LEFIK815-10	JN279062	BOLD:AAB9086	Finland	University of Oulu
Eudonia murana	TLMF Lep 08406	PHLAH587-12	KM573289	BOLD:AAB9087	Austria	inatura, Dornbirn
Eudonia murana	TLMF Lep 00795	PHLAA755-09	HM426140	BOLD:AAB9087	Austria	Tiroler Landesmuseum Ferdinandeum
Eudonia murana	TLMF Lep 00784	PHLAA744-09	HM426132	BOLD:AAB9087	Austria	Tiroler Landesmuseum Ferdinandeum
Eudonia sudetica	MM00717	LEFIB248-10	HM871151	BOLD:AAB9089	Finland	University of Oulu
Eudonia sudetica	MM04135	LEFIA781-10	HM386922	BOLD:AAB9089	Finland	University of Oulu
Eudonia sudetica	MM08041	LEFID995-10	HM873745	BOLD:AAB9089	Finland	University of Oulu
Eudonia sudetica	MM14647	LEFIG528-10	HM876201	BOLD:AAB9089	Finland	University of Oulu
Eudonia sudetica	TLMF Lep 08407	PHLAH588-12	KM573030	BOLD:AAB9089	Austria	inatura, Dornbirn
Eudonia truncicolella	MM13362	LEFIF923-10	HM875605	BOLD:AAB1558	Finland	University of Oulu
Eudonia truncicolella	TLMF Lep 08737	PHLAH933-12	KM572506	BOLD:AAB1558	Austria	Tiroler Landesmuseum Ferdinandeum
Eudonia truncicolella	MM02404	LEFIB642-10	HM871521	BOLD:AAB1558	Finland	University of Oulu
Eudonia truncicolella	MM04967	LEFIA1131-10	GU828709	BOLD:AAB1558	Finland	University of Oulu
Eudonia truncicolella	MM00771	LEFIB282-10	HM871184	BOLD:AAB1558	Finland	University of Oulu
Eudonia truncicolella	MM15777	LEFIG913-10	HM876557	BOLD:AAB1558	Finland	University of Oulu
Eugnorisma depuncta	MM12668	LEFIF678-10	HM875362	BOLD:AAE4805	Finland	University of Oulu
Eugnorisma depuncta	TLMF Lep 08767	PHLAI272-13	KM573219	BOLD:AAE4806	Austria	Tiroler Landesmuseum Ferdinandeum
Eugnorisma depuncta	MM04364	LEFIC582-10	HM872403	BOLD:AAE4806	Finland	University of Oulu
Eugnorisma depuncta	MM04892	LEFIC768-10	HM872587	BOLD:AAE4806	Finland	University of Oulu
Eugraphe sigma	TLMF Lep 10024	LEATA417-13	KM572678	BOLD:AAF8517	Austria	inatura, Dornbirn
Eugraphe sigma	MM19921	LEFII271-11	KM572714	BOLD:AAF8517	Finland	Research Collection of E. Laasonen
Eugraphe sigma	MM15963	LEFIJ363-10	KM572336	BOLD:AAF8517	Finland	University of Oulu

Eulamprotes atrella	MM02544	LEFIB724-10	HM871602	BOLD:AAD5702	Finland	University of Oulu
Eulamprotes atrella	MM02543	LEFIB723-10	HM871601	BOLD:AAD5702	Finland	University of Oulu
Eulamprotes atrella	MM09041	LEFIE427-10	HM874151	BOLD:AAD5702	Finland	University of Oulu
Eulamprotes atrella	TLMF Lep 09197	PHLAI635-13	KM572618	BOLD:AAD5702	Austria	Tiroler Landesmuseum Ferdinandeum
Eulamprotes unicolorella	TLMF Lep 08058	PHLAV239-12	KM572543	BOLD:AAD1198	Austria	inatura, Dornbirn
Eulamprotes unicolorella	MM06155	LEFID281-10	HM873079	BOLD:AAD1198	Finland	University of Oulu
Eulamprotes unicolorella	MM06156	LEFID282-10	HM873080	BOLD:AAD1198	Finland	University of Oulu
Eulamprotes unicolorella	MM03845	LEFIC338-10	HM872182	BOLD:AAD1198	Finland	University of Oulu
Eulamprotes unicolorella	MM13621	LEFIG008-10	HM875688	BOLD:AAD1198	Finland	University of Oulu
Eulamprotes unicolorella	MM02563	LEFIB734-10	HM871612	BOLD:AAD1198	Finland	University of Oulu
Eulamprotes unicolorella	MM03824	LEFIC320-10	HM872164	BOLD:AAD1198	Finland	University of Oulu
Eulia ministrana	MM00064	LEFIB024-10	HM870935	BOLD:AAA7315	Finland	University of Oulu
Eulia ministrana	MM01066	LEFIA132-10	HM396479	BOLD:AAA7315	Finland	University of Oulu
Eulia ministrana	MM00078	LEFIA007-10	HM396357	BOLD:AAA7315	Finland	University of Oulu
Eulia ministrana	MM01067	LEFIA133-10	HM396480	BOLD:AAA7315	Finland	University of Oulu
Eulia ministrana	MM00109	LEFIB026-10	HM870937	BOLD:AAA7315	Finland	University of Oulu
Eulia ministrana	TLMF Lep 09144	PHLAI582-13	KM573628	BOLD:AAA7315	Austria	Tiroler Landesmuseum Ferdinandeum
Eulia ministrana	MM13206	LEFIF851-10	HM875534	BOLD:AAA7315	Finland	University of Oulu
Eulia ministrana	MM06310	LEFIA1133-10	GU828742	BOLD:AAA7315	Finland	University of Oulu
Eulia ministrana	MM03425	LEFIC105-10	HQ570296	BOLD:AAA7315	Finland	University of Oulu
Eulithis populata	TLMF Lep 06166	PHLSA711-11	KM572127	BOLD:ABZ1837	Austria	Tiroler Landesmuseum Ferdinandeum
Eulithis populata	MM05173	LEFIC885-10	HM872703	BOLD:ABZ1837	Finland	University of Oulu
Eulithis populata	MM08084	LEFIE024-10	HM873773	BOLD:ABZ1837	Finland	University of Oulu
Eulithis populata	TLMF Lep 06165	PHLSA710-11	KM573042	BOLD:ABZ1837	Austria	Tiroler Landesmuseum Ferdinandeum
Eulithis populata	MM00797	LEFIB293-10	HM871194	BOLD:ABZ1837	Finland	University of Oulu
Eumedonia eumedon	TLMF Lep 06152	PHLSA697-11	KM573528	BOLD:AAC3364	Austria	Tiroler Landesmuseum Ferdinandeum
Eumedonia eumedon	MM17140	LEFIJ515-10	JF853637	BOLD:AAC3364	Finland	University of Oulu
Eumedonia eumedon	TLMF Lep 09843	PHLAW046-13	KM572165	BOLD:AAC3364	Austria	Tiroler Landesmuseum Ferdinandeum
Eumedonia eumedon	MM03212	LEFIC009-10	HM871879	BOLD:AAC3364	Finland	University of Oulu
Eumedonia eumedon	MM14128	LEFIG228-10	HM875908	BOLD:AAC3364	Finland	University of Oulu
Euphydryas aurinia	TLMF Lep 08276	PHLAH457-12	KM572826	BOLD:ACF4353	Austria	inatura, Dornbirn

Euphydryas aurinia	MM18691	LEFIL393-10	KM572689	BOLD:ACF4353	Finland	University of Oulu
Eupithecia	BIOUG04490-E09	GMFIC395-13	KM573455	BOLD:AAB3785	Finland	Biodiversity Institute of Ontario
Eupithecia	BIOUG04116-B06	GMFIF757-12	KM572773	BOLD:AAB3785	Finland	Biodiversity Institute of Ontario
Eupithecia abietaria	TLMF Lep 07558	PHLAG879-12	KM573476	BOLD:AAD1084	Austria	inatura, Dornbirn
Eupithecia abietaria	MM14283	LEFIG316-10	HM875995	BOLD:AAD1084	Finland	University of Oulu
Eupithecia abietaria	TLMF Lep 07979	PHLAV160-12	KM572002	BOLD:AAD1084	Austria	inatura, Dornbirn
Eupithecia abietaria	MM09780	LEFIA902-10	HM387038	BOLD:AAD1084	Finland	University of Oulu
Eupithecia abietaria	MM06844	LEFID777-10	HM873534	BOLD:AAD1084	Finland	University of Oulu
Eupithecia absinthiata	MM15997	LEFIJ392-10	JF853578	BOLD:ACE4737	Finland	University of Oulu
Eupithecia absinthiata	TLMF Lep 10002	LEATA395-13	KM572341	BOLD:ACE4737	Austria	inatura, Dornbirn
Eupithecia absinthiata	MM01820	LEFIA681-10	HM386827	BOLD:ACE4737	Finland	University of Oulu
Eupithecia absinthiata	MM17244	LEFIJ619-10	JF853719	BOLD:ACE4737	Finland	University of Oulu
Eupithecia absinthiata	MM15999	LEFIJ394-10	JF853580	BOLD:ACE4737	Finland	University of Oulu
Eupithecia absinthiata	MM15994	LEFIJ389-10	JF853576	BOLD:ACE4737	Finland	University of Oulu
Eupithecia absinthiata	MM15995	LEFIJ390-10	JF853577	BOLD:ACE4737	Finland	University of Oulu
Eupithecia absinthiata	MM13864	LEFIA936-10	HM387070	BOLD:ACE4737	Finland	University of Oulu
Eupithecia absinthiata	MM01821	LEFIA682-10	HM386828	BOLD:ACE4737	Finland	University of Oulu
Eupithecia absinthiata	MM04944	LEFIC795-10	HM872614	BOLD:ACE4737	Finland	University of Oulu
Eupithecia absinthiata	TLMF Lep 08500	PHLAH681-12	KM573242	BOLD:ACE4737	Austria	inatura, Dornbirn
Eupithecia absinthiata	TLMF Lep 08541	PHLAH722-12	KM572963	BOLD:ACE4737	Austria	inatura, Dornbirn
Eupithecia absinthiata	MM15998	LEFIJ393-10	JF853579	BOLD:ACE4737	Finland	University of Oulu
Eupithecia absinthiata	MM12972	LEFIF762-10	HM875446	BOLD:ACE4737	Finland	University of Oulu
Eupithecia egenaria	MM05925	LEFID161-10	HM872968	BOLD:AAD9932	Finland	University of Oulu
Eupithecia egenaria	TLMF Lep 07982	PHLAV163-12	KM572163	BOLD:AAD9932	Austria	inatura, Dornbirn
Eupithecia egenaria	MM05657	LEFID057-10	HM872871	BOLD:AAD9932	Finland	University of Oulu
Eupithecia egenaria	MM18704	LEFIL406-10	JF854585	BOLD:AAD9932	Finland	University of Oulu
Eupithecia exiguata	MM01769	LEFIA652-10	HM870901	BOLD:AAB5464	Finland	University of Oulu
Eupithecia exiguata	MM12910	LEFIF746-10	HM875430	BOLD:AAB5464	Finland	University of Oulu
Eupithecia exiguata	MM03846	LEFIC339-10	HM872183	BOLD:AAB5464	Finland	University of Oulu
Eupithecia exiguata	TLMF Lep 09881	PHLAW084-13	KM572009	BOLD:AAB5464	Austria	Tiroler Landesmuseum Ferdinandeum
Eupithecia exiguata	TLMF Lep 07875	PHLAV056-12	KM572730	BOLD:AAB5464	Austria	inatura, Dornbirn

Eupithecia icterata	MM01798	LEFIA666-10	HQ963153	BOLD:AAB6528	Finland	University of Oulu
Eupithecia icterata	MM04941	LEFIC793-10	HM872612	BOLD:AAB6528	Finland	University of Oulu
Eupithecia icterata	MM12963	LEFIF754-10	HM875438	BOLD:AAB6528	Finland	University of Oulu
Eupithecia icterata	MM07966	LEFID968-10	HM873718	BOLD:AAB6528	Finland	University of Oulu
Eupithecia icterata	TLMF Lep 09997	LEATA390-13	KM573431	BOLD:ACE7878	Austria	inatura, Dornbirn
Eupithecia indigata	MM04079	LEFIC483-10	HM872315	BOLD:AAD4847	Finland	University of Oulu
Eupithecia indigata	MM12909	LEFIF745-10	HM875429	BOLD:AAD4847	Finland	University of Oulu
Eupithecia indigata	TLMF Lep 07873	PHLAV054-12	KM573374	BOLD:AAD4847	Austria	inatura, Dornbirn
Eupithecia indigata	MM00568	LEFIB167-10	HM871071	BOLD:AAD4847	Finland	University of Oulu
Eupithecia intricata	TLMF Lep 09799	PHLAW002-13	KM572145	BOLD:ACJ7134	Austria	Tiroler Landesmuseum Ferdinandeum
Eupithecia intricata	MM01806	LEFIA672-10	HM386820	BOLD:ACJ7134	Finland	University of Oulu
Eupithecia intricata	MM07963	LEFID967-10	HM873717	BOLD:ACJ7134	Finland	University of Oulu
Eupithecia intricata	MM18480	LEFIK905-10	JN279415	BOLD:ACJ7134	Finland	University of Oulu
Eupithecia lanceata	TLMF Lep 08811	PHLAI316-13	KM572709	BOLD:AAC5856	Austria	Tiroler Landesmuseum Ferdinandeum
Eupithecia lanceata	MM05428	LEFID007-10	HM872821	BOLD:AAC5856	Finland	University of Oulu
Eupithecia lanceata	MM11647	LEFIF383-10	HM875068	BOLD:AAC5856	Finland	University of Oulu
Eupithecia lanceata	MM04624	LEFIC664-10	HM872485	BOLD:AAC5856	Finland	University of Oulu
Eupithecia lanceata	MM05445	LEFID018-10	HM872832	BOLD:AAC5856	Finland	University of Oulu
Eupithecia lariciata	TLMF Lep 07874	PHLAV055-12	KM572109	BOLD:AAA4421	Austria	inatura, Dornbirn
Eupithecia lariciata	MM07976	LEFID970-10	HM873720	BOLD:AAA4421	Finland	University of Oulu
Eupithecia lariciata	MM18504	LEFIK929-10	JF854372	BOLD:AAA4421	Finland	University of Oulu
Eupithecia lariciata	MM18505	LEFIK930-10	JF854373	BOLD:AAA4421	Finland	University of Oulu
Eupithecia plumbeolata	MM01813	LEFIB407-10	HM871306	BOLD:AAB8936	Finland	University of Oulu
Eupithecia plumbeolata	TLMF Lep 07575	PHLAG896-12	KM572799	BOLD:AAB8936	Austria	inatura, Dornbirn
Eupithecia plumbeolata	MM01814	LEFIJ036-10	KM573024	BOLD:AAB8936	Finland	University of Oulu
Eupithecia plumbeolata	MM06512	LEFID530-10	HM873295	BOLD:AAB8936	Finland	University of Oulu
Eupithecia plumbeolata	TLMF Lep 09917	PHLAW120-13	KM572621	BOLD:AAB8936	Austria	Tiroler Landesmuseum Ferdinandeum
Eupithecia plumbeolata	MM14229	LEFIG283-10	HM875962	BOLD:AAB8936	Finland	University of Oulu
Eupithecia plumbeolata	MM01811	LEFIA675-10	HM386823	BOLD:AAB8936	Finland	University of Oulu
Eupithecia plumbeolata	MM11711	LEFIJ178-10	KM572362	BOLD:AAB8937	Finland	University of Oulu
Eupithecia plumbeolata	MM18473	LEFIK898-10	JF854348	BOLD:AAB8937	Finland	University of Oulu

Eupithecia plumbeolata	MM12492	LEFIJ195-10	KM572360	BOLD:AAB8937	Finland	University of Oulu
Eupithecia plumbeolata	MM06648	LEFID629-10	HM873392	BOLD:AAB8937	Finland	University of Oulu
Eupithecia plumbeolata	MM07959	LEFID965-10	HM873715	BOLD:AAB8937	Finland	University of Oulu
Eupithecia plumbeolata	MM18472	LEFIK897-10	JF854347	BOLD:AAB8937	Finland	University of Oulu
Eupithecia plumbeolata	MM11710	LEFIF392-10	HM875077	BOLD:AAB8937	Finland	University of Oulu
Eupithecia plumbeolata	MM12970	LEFIF760-10	HM875444	BOLD:AAB8937	Finland	University of Oulu
Eupithecia plumbeolata	MM14180	LEFIG256-10	HM875935	BOLD:AAB8937	Finland	University of Oulu
Eupithecia plumbeolata	MM18475	LEFIK900-10	JF854349	BOLD:AAB8937	Finland	University of Oulu
Eupithecia plumbeolata	MM01812	LEFIA676-10	HM386824	BOLD:AAB8937	Finland	University of Oulu
Eupithecia plumbeolata	MM18474	LEFIK899-10	KM573070	BOLD:ACF3745	Finland	University of Oulu
Eupithecia pusillata	TLMF Lep 08798	PHLAI303-13	KM573465	BOLD:ABZ6329	Austria	Tiroler Landesmuseum Ferdinandeum
Eupithecia pusillata	MM00781	LEFIB288-10	HM871189	BOLD:ABZ6329	Finland	University of Oulu
Eupithecia pusillata	MM01786	LEFIA659-10	HM870907	BOLD:ABZ6329	Finland	University of Oulu
Eupithecia pusillata	TLMF Lep 08773	PHLAI278-13	KM572224	BOLD:ABZ6329	Austria	Tiroler Landesmuseum Ferdinandeum
Eupithecia pusillata	MM17213	LEFIJ588-10	KM573453	BOLD:ABZ6329	Finland	University of Oulu
Eupithecia pusillata	MM23017	LEFIJ1730-13	KM572758	BOLD:ABZ6329	Finland	University of Oulu
Eupithecia pusillata	MM23016	LEFIJ1729-13	KM572837	BOLD:ABZ6329	Finland	University of Oulu
Eupithecia pusillata	MM23013	LEFIJ1726-13	KM573291	BOLD:ABZ6329	Finland	University of Oulu
Eupithecia pusillata	MM23012	LEFIJ1725-13	KM572349	BOLD:ABZ6329	Finland	University of Oulu
Eupithecia pusillata	MM23011	LEFIJ1724-13	KM572206	BOLD:ABZ6329	Finland	University of Oulu
Eupithecia pusillata	TLMF Lep 08772	PHLAI277-13	KM572696	BOLD:ABZ6329	Austria	Tiroler Landesmuseum Ferdinandeum
Eupithecia pusillata	MM23010	LEFIJ1723-13	KM572214	BOLD:ABZ6329	Finland	University of Oulu
Eupithecia pusillata	MM23018	LEFIJ1731-13	KM573261	BOLD:ABZ6329	Finland	University of Oulu
Eupithecia pusillata	MM23020	LEFIJ1733-13	KM573185	BOLD:ABZ6329	Finland	University of Oulu
Eupithecia pusillata	MM08143	LEFIE045-10	HM873793	BOLD:ABZ6329	Finland	University of Oulu
Eupithecia pusillata	MM08340	LEFIE127-10	HM873875	BOLD:ABZ6329	Finland	University of Oulu
Eupithecia pusillata	MM23019	LEFIJ1732-13	KM572378	BOLD:ABZ6329	Finland	University of Oulu
Eupithecia pusillata	MM23021	LEFIJ1734-13	KM572594	BOLD:ABZ6329	Finland	University of Oulu
Eupithecia pusillata	MM01785	LEFIA658-10	HM870906	BOLD:ABZ6329	Finland	University of Oulu
Eupithecia satyrata	MM01789	LEFIJ034-10	KM572182	BOLD:AAA4219	Finland	University of Oulu
Eupithecia satyrata	MM02923	LEFIJ049-10	KM572241	BOLD:AAA4219	Finland	University of Oulu

Eupithecia satyrata	MM21047	LEFIJ1187-11	KM573446	BOLD:AAA4219	Finland	University of Oulu
Eupithecia satyrata	MM18488	LEFIK913-10	KM573122	BOLD:AAA4219	Finland	University of Oulu
Eupithecia satyrata	MM18490	LEFIK915-10	JF854361	BOLD:AAA4219	Finland	University of Oulu
Eupithecia satyrata	MM18491	LEFIK916-10	JF854362	BOLD:AAA4219	Finland	University of Oulu
Eupithecia satyrata	MM18482	LEFIK907-10	JF854354	BOLD:AAA4219	Finland	University of Oulu
Eupithecia satyrata	MM18484	LEFIK909-10	JF854356	BOLD:AAA4219	Finland	University of Oulu
Eupithecia satyrata	MM18485	LEFIK910-10	JF854357	BOLD:AAA4219	Finland	University of Oulu
Eupithecia satyrata	MM18486	LEFIK911-10	JF854358	BOLD:AAA4219	Finland	University of Oulu
Eupithecia satyrata	MM18487	LEFIK912-10	JF854359	BOLD:AAA4219	Finland	University of Oulu
Eupithecia satyrata	MM18489	LEFIK914-10	JF854360	BOLD:AAA4219	Finland	University of Oulu
Eupithecia satyrata	TLMF Lep 09882	PHLAW085-13	KM572717	BOLD:AAA4219	Austria	Tiroler Landesmuseum Ferdinandeum
Eupithecia satyrata	MM06202	LEFID317-10	HM873114	BOLD:AAA4219	Finland	University of Oulu
Eupithecia satyrata	MM01787	LEFIA660-10	HM870908	BOLD:AAA4219	Finland	University of Oulu
Eupithecia satyrata	MM01788	LEFIA661-10	HM870909	BOLD:AAA4219	Finland	University of Oulu
Eupithecia satyrata	MM00566	LEFIB166-10	HM871070	BOLD:AAA4219	Finland	University of Oulu
Eupithecia satyrata	MM12962	LEFIF753-10	HM875437	BOLD:AAA4219	Finland	University of Oulu
Eupithecia satyrata	MM18797	LEFIL499-10	JF854626	BOLD:AAA4219	Finland	University of Oulu
Eupithecia satyrata	MM04689	LEFIC699-10	HM872520	BOLD:AAA4219	Finland	University of Oulu
Eupithecia satyrata	MM17985	LEFIK410-10	JF854039	BOLD:AAA4219	Finland	University of Oulu
Eupithecia satyrata	MM07962	LEFIJ111-10	KM572141	BOLD:AAA4219	Finland	University of Oulu
Eupithecia satyrata	MM12197	LEFIJ182-10	KM572079	BOLD:AAA4219	Finland	University of Oulu
Eupithecia satyrata	MM00567	LEFIJ026-10	JF853414	BOLD:AAA4219	Finland	University of Oulu
Eupithecia satyrata	MM00606	LEFIJ027-10	JF853415	BOLD:AAA4219	Finland	University of Oulu
Eupithecia satyrata	MM00633	LEFIJ028-10	JF853416	BOLD:AAA4219	Finland	University of Oulu
Eupithecia satyrata	MM23033	LEFIJ1746-13	KM573335	BOLD:AAA4219	Finland	University of Oulu
Eupithecia satyrata	MM23027	LEFIJ1740-13	KM571978	BOLD:AAA4219	Finland	University of Oulu
Eupithecia satyrata	MM23023	LEFIJ1736-13	KM572511	BOLD:AAA4219	Finland	University of Oulu
Eupithecia satyrata	MM22896	LEFIJ2044-13	KM572189	BOLD:AAA4219	Finland	University of Oulu
Eupithecia satyrata	MM23028	LEFIJ1741-13	KM573378	BOLD:AAA4219	Finland	University of Oulu
Eupithecia satyrata	MM23024	LEFIJ1737-13	KM572670	BOLD:AAA4219	Finland	University of Oulu
Eupithecia satyrata	MM23032	LEFIJ1745-13	KM573344	BOLD:AAA4219	Finland	University of Oulu

Eupithecia satyrata	MM05487	LEFIJ071-10	JF853422	BOLD:AAA4219	Finland	University of Oulu
Eupithecia satyrata	MM01790	LEFIJ035-10	KM572620	BOLD:AAA4219	Finland	University of Oulu
Eupithecia satyrata	MM01805	LEFIA671-10	HM386819	BOLD:AAA5442	Finland	University of Oulu
Eupithecia satyrata	MM18483	LEFIK908-10	JF854355	BOLD:AAA5442	Finland	University of Oulu
Eupithecia selinata	TLMF Lep 09995	LEATA388-13	KM572017	BOLD:AAD5171	Austria	inatura, Dornbirn
Eupithecia selinata	MM01808	LEFIA673-10	HM386821	BOLD:AAD5171	Finland	University of Oulu
Eupithecia selinata	MM01809	LEFIA674-10	HM386822	BOLD:AAD5171	Finland	University of Oulu
Eupithecia selinata	MM18479	LEFIK904-10	JF854352	BOLD:AAD5171	Finland	University of Oulu
Eupithecia selinata	MM05655	LEFID056-10	HM872870	BOLD:AAD5171	Finland	University of Oulu
Eupithecia subfuscata	TLMF Lep 07899	PHLAV080-12	KM573045	BOLD:ABY4251	Austria	inatura, Dornbirn
Eupithecia subfuscata	MM04228	LEFIC517-10	HM872341	BOLD:ACE8007	Finland	University of Oulu
Eupithecia subfuscata	MM06647	LEFID628-10	HM873391	BOLD:ACE8007	Finland	University of Oulu
Eupithecia subfuscata	TLMF Lep 06134	PHLSA679-11	KM572449	BOLD:ACE8007	Austria	Tiroler Landesmuseum Ferdinandeum
Eupithecia subfuscata	MM00613	LEFIB186-10	HM871090	BOLD:ACE8007	Finland	University of Oulu
Eupithecia subfuscata	BIOUG04490-E12	GMFID736-12	KM572008	BOLD:ACE8007	Finland	Biodiversity Institute of Ontario
Eupithecia subfuscata	MM07970	LEFID969-10	HM873719	BOLD:ACE8007	Finland	University of Oulu
Eupithecia subfuscata	TLMF Lep 07900	PHLAV081-12	KM573301	BOLD:ACE8007	Austria	inatura, Dornbirn
Eupithecia subfuscata	TLMF Lep 07901	PHLAV082-12	KM573400	BOLD:ACE8007	Austria	inatura, Dornbirn
Eupithecia subfuscata	TLMF Lep 07984	PHLAV165-12	KM573408	BOLD:ACE8007	Austria	inatura, Dornbirn
Eupithecia subfuscata	MM01816	LEFIA677-10	HQ963154	BOLD:ACE8007	Finland	University of Oulu
Eupithecia subfuscata	MM01817	LEFIA678-10	HQ963155	BOLD:ACE8007	Finland	University of Oulu
Eupithecia subumbrata	TLMF Lep 07570	PHLAG891-12	KM572625	BOLD:AAC9951	Austria	inatura, Dornbirn
Eupithecia subumbrata	MM10475	LEFIF035-10	HM874746	BOLD:AAC9952	Finland	University of Oulu
Eupithecia subumbrata	MM17891	LEFIK316-10	JF853964	BOLD:AAC9952	Finland	University of Oulu
Eupithecia subumbrata	MM03880	LEFIC359-10	HM872202	BOLD:AAC9952	Finland	University of Oulu
Eupithecia subumbrata	MM17334	LEFIJ709-10	JF853785	BOLD:AAC9952	Finland	University of Oulu
Eupithecia subumbrata	MM03879	LEFIC358-10	HM872201	BOLD:AAC9952	Finland	University of Oulu
Eupithecia subumbrata	MM03805	LEFIC308-10	HM872152	BOLD:AAC9952	Finland	University of Oulu
Eupithecia subumbrata	MM12965	LEFIF756-10	HM875440	BOLD:AAC9952	Finland	University of Oulu
Eupithecia subumbrata	MM10474	LEFIF034-10	HM874745	BOLD:AAC9952	Finland	University of Oulu
Eupithecia tantillaria	TLMF Lep 04606	PHLAE291-11	JN279561	BOLD:AAB3785	Austria	Tiroler Landesmuseum Ferdinandeum

Eupithecia tantillaria	MM03848	LEFIC341-10	HM872185	BOLD:AAB3785	Finland	University of Oulu
Eupithecia tantillaria	MM07961	LEFID966-10	HM873716	BOLD:AAB3785	Finland	University of Oulu
Eupithecia tantillaria	MM12961	LEFIF752-10	HM875436	BOLD:AAB3785	Finland	University of Oulu
Eupithecia tantillaria	MM09961	LEFIE783-10	HM874502	BOLD:AAB3785	Finland	University of Oulu
Eupithecia tenuiata	MM01818	LEFIA679-10	HM386825	BOLD:AAC7394	Finland	University of Oulu
Eupithecia tenuiata	TLMF Lep 08774	PHLAI279-13	KM572181	BOLD:AAC7394	Austria	Tiroler Landesmuseum Ferdinandeum
Eupithecia tenuiata	MM12968	LEFIF759-10	HM875443	BOLD:AAC7394	Finland	University of Oulu
Eupithecia tenuiata	MM03509	LEFIC162-10	HM872008	BOLD:AAC7394	Finland	University of Oulu
Eupithecia tenuiata	TLMF Lep 08546	PHLAH727-12	KM572537	BOLD:AAC7394	Austria	inatura, Dornbirn
Eupithecia tenuiata	MM01819	LEFIA680-10	HM386826	BOLD:AAC7394	Finland	University of Oulu
Eupithecia trisignaria	TLMF Lep 06140	PHLSA685-11	KM573605	BOLD:AAD5170	Austria	Tiroler Landesmuseum Ferdinandeum
Eupithecia trisignaria	MM06716	LEFID675-10	HM873436	BOLD:AAD5170	Finland	University of Oulu
Eupithecia trisignaria	MM09680	LEFIE698-10	HM874419	BOLD:AAD5170	Finland	University of Oulu
Eupithecia trisignaria	MM18492	LEFIK917-10	JF854363	BOLD:AAD5170	Finland	University of Oulu
Eupithecia venosata	MM15821	LEFIG957-10	HM876597	BOLD:AAC2038	Finland	University of Oulu
Eupithecia venosata	MM18478	LEFIK903-10	KM572014	BOLD:AAC2038	Finland	University of Oulu
Eupithecia venosata	MM15822	LEFIG958-10	HM876598	BOLD:AAC2038	Finland	University of Oulu
Eupithecia venosata	TLMF Lep 06135	PHLSA680-11	KM573134	BOLD:AAC2038	Austria	Tiroler Landesmuseum Ferdinandeum
Eupithecia virgaureata	MM10109	LEFIE862-10	HM874580	BOLD:ACE4093	Finland	University of Oulu
Eupithecia virgaureata	MM23002	LEFIJ1715-13	KM572958	BOLD:ACE4093	Finland	University of Oulu
Eupithecia virgaureata	MM23003	LEFIJ1716-13	KM572676	BOLD:ACE4093	Finland	University of Oulu
Eupithecia virgaureata	MM23004	LEFIJ1717-13	KM572547	BOLD:ACE4093	Finland	University of Oulu
Eupithecia virgaureata	MM23000	LEFIJ1713-13	KM573085	BOLD:ACE4093	Finland	University of Oulu
Eupithecia virgaureata	MM22998	LEFIJ1711-13	KM573506	BOLD:ACE4093	Finland	University of Oulu
Eupithecia virgaureata	MM23005	LEFIJ1718-13	KM573603	BOLD:ACE4093	Finland	University of Oulu
Eupithecia virgaureata	MM23006	LEFIJ1719-13	KM573279	BOLD:ACE4093	Finland	University of Oulu
Eupithecia virgaureata	MM23007	LEFIJ1720-13	KM572421	BOLD:ACE4093	Finland	University of Oulu
Eupithecia virgaureata	MM23008	LEFIJ1721-13	KM572841	BOLD:ACE4093	Finland	University of Oulu
Eupithecia virgaureata	MM23009	LEFIJ1722-13	KM572157	BOLD:ACE4093	Finland	University of Oulu
Eupithecia virgaureata	MM22999	LEFIJ1712-13	KM572465	BOLD:ACE4093	Finland	University of Oulu
Eupithecia virgaureata	TLMF Lep 08802	PHLAI307-13	KM573051	BOLD:ACE4093	Austria	Tiroler Landesmuseum Ferdinandeum

Eupithecia virgaureata	TLMF Lep 08801	PHLAI306-13	KM572222	BOLD:ACE4093	Austria	Tiroler Landesmuseum Ferdinandeum
Eupithecia virgaureata	MM01797	LEFIA665-10	HM870913	BOLD:ACE4093	Finland	University of Oulu
Eupithecia virgaureata	MM23001	LEFIJ1714-13	KM572628	BOLD:ACE4093	Finland	University of Oulu
Eupithecia virgaureata	MM12957	LEFIF750-10	HM875434	BOLD:ACE4093	Finland	University of Oulu
Eupithecia virgaureata	MM06414	LEFID472-10	HM873240	BOLD:ACE4093	Finland	University of Oulu
Eupithecia virgaureata	MM06293	LEFID382-10	HM873179	BOLD:ACE4093	Finland	University of Oulu
Eupithecia vulgata	MM08172	LEFIE056-10	HM873803	BOLD:AAA8708	Finland	University of Oulu
Eupithecia vulgata	MM08171	LEFIE055-10	HM873802	BOLD:AAA8708	Finland	University of Oulu
Eupithecia vulgata	MM00632	LEFIB196-10	HM871100	BOLD:AAA8708	Finland	University of Oulu
Eupithecia vulgata	MM00631	LEFIB195-10	HM871099	BOLD:AAA8708	Finland	University of Oulu
Eupithecia vulgata	MM01800	LEFIA668-10	HM386817	BOLD:AAA8708	Finland	University of Oulu
Eupithecia vulgata	MM01799	LEFIA667-10	HM386816	BOLD:AAA8708	Finland	University of Oulu
Eupithecia vulgata	TLMF Lep 09916	PHLAW119-13	KM573442	BOLD:AAA8708	Austria	Tiroler Landesmuseum Ferdinandeum
Eupithecia vulgata	MM12971	LEFIF761-10	HM875445	BOLD:AAA8708	Finland	University of Oulu
Euplexia lucipara	MM01540	LEFIA469-10	HM386809	BOLD:AAC2054	Finland	University of Oulu
Euplexia lucipara	MM04733	LEFIC706-10	HM872527	BOLD:AAC2054	Finland	University of Oulu
Euplexia lucipara	TLMF Lep 08135	PHLAV316-12	KM573363	BOLD:AAC2054	Austria	inatura, Dornbirn
Euplexia lucipara	MM01541	LEFIA470-10	HM386810	BOLD:AAC2054	Finland	University of Oulu
Eupoecilia ambiguella	TLMF Lep 07989	PHLAV170-12	KM573550	BOLD:AAD8039	Austria	inatura, Dornbirn
Eupoecilia ambiguella	MM15673	LEFIG809-10	HM876461	BOLD:AAD8039	Finland	University of Oulu
Eupoecilia ambiguella	MM06468	LEFID505-10	HM873270	BOLD:AAD8039	Finland	University of Oulu
Eupoecilia ambiguella	MM15672	LEFIG808-10	HM876460	BOLD:AAD8039	Finland	University of Oulu
Eupoecilia angustana	TLMF Lep 09174	PHLAI612-13	KM573576	BOLD:AAC8058	Austria	Tiroler Landesmuseum Ferdinandeum
Eupoecilia angustana	MM09005	LEFIE418-10	HM874142	BOLD:AAC8058	Finland	University of Oulu
Eupoecilia angustana	MM02556	LEFIB730-10	HM871608	BOLD:AAC8058	Finland	University of Oulu
Eupoecilia angustana	MM11846	LEFIF425-10	HM875110	BOLD:AAC8058	Finland	University of Oulu
Eupsilia transversa	TLMF Lep 06142	PHLSA687-11	KM571951	BOLD:AAC7414	Austria	Tiroler Landesmuseum Ferdinandeum
Eupsilia transversa	MM04621	LEFIC662-10	HM872483	BOLD:AAC7414	Finland	University of Oulu
Eupsilia transversa	MM15866	LEFIH002-10	HM876638	BOLD:AAC7414	Finland	University of Oulu
Eupsilia transversa	TLMF Lep 04653	PHLAE338-11	JN266573	BOLD:AAC7414	Austria	Tiroler Landesmuseum Ferdinandeum
Eupsilia transversa	MM15867	LEFIH003-10	HM876639	BOLD:AAC7414	Finland	University of Oulu

Eupsilia transversa	TLMF Lep 06195	PHLSA740-11	KM572873	BOLD:AAC7414	Austria	Tiroler Landesmuseum Ferdinandeum
Eurois	BIOUG04118-F08	GMFIT048-13	KM572179	BOLD:AAA3312	Finland	Biodiversity Institute of Ontario
Eurois occulta	TLMF Lep 00291	PHLAA251-09	HM425790	BOLD:AAA3312	Austria	Tiroler Landesmuseum Ferdinandeum
Eurois occulta	MM02786	LEFIB831-10	HM871708	BOLD:AAA3312	Finland	University of Oulu
Eurois occulta	MM01687	LEFIA582-10	HM870831	BOLD:AAA3312	Finland	University of Oulu
Eurois occulta	MM01688	LEFIA583-10	HM870832	BOLD:AAA3312	Finland	University of Oulu
Euspilapteryx auroguttella	MM14063	LEFIG190-10	HM875870	BOLD:AAD7434	Finland	University of Oulu
Euspilapteryx auroguttella	TLMF Lep 08453	PHLAH634-12	KM573327	BOLD:AAD7434	Austria	inatura, Dornbirn
Euspilapteryx auroguttella	MM02624	LEFIB762-10	HM871639	BOLD:AAD7434	Finland	University of Oulu
Euspilapteryx auroguttella	MM13894	LEFIG107-10	HM875787	BOLD:AAD7434	Finland	University of Oulu
Eustroma reticulata	TLMF Lep 04614	PHLAE299-11	JN279565	BOLD:AAC8101	Austria	Tiroler Landesmuseum Ferdinandeum
Eustroma reticulata	MM03537	LEFIC182-10	HM872026	BOLD:AAC8101	Finland	University of Oulu
Eustroma reticulata	MM03516	LEFIC165-10	HM872011	BOLD:AAC8101	Finland	University of Oulu
Eustroma reticulata	MM02373	LEFIB627-10	HM871506	BOLD:AAC8101	Finland	University of Oulu
Euthrix potatoria	MM07310	LEFID910-10	HM873660	BOLD:AAC1584	Finland	University of Oulu
Euthrix potatoria	MM01056	LEFIA126-10	HM396473	BOLD:AAC1584	Finland	University of Oulu
Euthrix potatoria	MM01057	LEFIA127-10	HM396474	BOLD:AAC1584	Finland	University of Oulu
Euthrix potatoria	TLMF Lep 08528	PHLAH709-12	KM572240	BOLD:AAC1584	Austria	inatura, Dornbirn
Euxoa nigricans	MM06955	LEFID838-10	HM873595	BOLD:ABZ9438	Finland	University of Oulu
Euxoa nigricans	MM18674	LEFIL364-10	KM573348	BOLD:ABZ9438	Finland	University of Oulu
Euxoa nigricans	MM14694	LEFIG551-10	HM876224	BOLD:ABZ9438	Finland	University of Oulu
Euxoa nigricans	MM04904	LEFIC774-10	HM872593	BOLD:ABZ9438	Finland	University of Oulu
Euxoa nigricans	MM18655	LEFIL345-10	KM573372	BOLD:ABZ9438	Finland	University of Oulu
Euxoa nigricans	MM04375	LEFIC583-10	HM872404	BOLD:ABZ9438	Finland	University of Oulu
Euxoa nigricans	TLMF Lep 06188	PHLSA733-11	KM573304	BOLD:ABZ9438	Austria	Tiroler Landesmuseum Ferdinandeum
Euxoa nigricans	MM14690	LEFIG547-10	HM876220	BOLD:ABZ9438	Finland	University of Oulu
Euxoa nigricans	MM14691	LEFIG548-10	HM876221	BOLD:ABZ9438	Finland	University of Oulu
Euxoa nigricans	MM03617	LEFIC221-10	HM872065	BOLD:ABZ9438	Finland	University of Oulu
Euxoa nigricans	MM06979	LEFID853-10	HM873610	BOLD:ABZ9438	Finland	University of Oulu
Euxoa recussa	MM00731	LEFIB259-10	HM871162	BOLD:ACE9579	Finland	University of Oulu
Euxoa recussa	MM00730	LEFIB258-10	HM871161	BOLD:ACE9579	Finland	University of Oulu

Euxoa recussa	MM18001	LEFIK426-10	JF854055	BOLD:ACE9579	Finland	University of Oulu
Euxoa recussa	TLMF Lep 06097	PHLSA642-11	KM572912	BOLD:ACE9579	Austria	Tiroler Landesmuseum Ferdinandeum
Evergestis forficalis	MM01218	LEFIA1141-10	GU828662	BOLD:AAB7956	Finland	University of Oulu
Evergestis forficalis	MM12982	LEFIF767-10	HM875451	BOLD:AAB7956	Finland	University of Oulu
Evergestis forficalis	TLMF Lep 12525	LEATC543-13	KM572793	BOLD:AAB7956	Austria	Tiroler Landesmuseum Ferdinandeum
Evergestis forficalis	MM04956	LEFIC798-10	HM872617	BOLD:AAB7956	Finland	University of Oulu
Evergestis forficalis	MM01874	LEFIA719-10	HM386863	BOLD:AAB7956	Finland	University of Oulu
Evergestis pallidata	MM01856	LEFIA701-10	HM386846	BOLD:AAB4832	Finland	University of Oulu
Evergestis pallidata	MM02957	LEFIB882-10	HM871759	BOLD:AAB4832	Finland	University of Oulu
Evergestis pallidata	TLMF Lep 08731	PHLAH927-12	KM573323	BOLD:AAB4832	Austria	Tiroler Landesmuseum Ferdinandeum
Evergestis pallidata	MM01857	LEFIA702-10	HM386847	BOLD:AAB4832	Finland	University of Oulu
Exoteleia dodecella	MM03918	LEFIC386-10	HM872229	BOLD:AAA8390	Finland	University of Oulu
Exoteleia dodecella	MM09030	LEFIE422-10	HM874146	BOLD:AAA8390	Finland	University of Oulu
Exoteleia dodecella	MM05214	LEFIC901-10	HM872718	BOLD:AAA8390	Finland	University of Oulu
Exoteleia dodecella	TLMF Lep 00871	PHLAB071-10	HQ968245	BOLD:AAA8390	Austria	Tiroler Landesmuseum Ferdinandeum
Falcaria lacertinaria	TLMF Lep 06178	PHLSA723-11	KM573165	BOLD:AAA6343	Austria	Tiroler Landesmuseum Ferdinandeum
Falcaria lacertinaria	MM01257	LEFIA225-10	HM396568	BOLD:AAA6343	Finland	University of Oulu
Falcaria lacertinaria	MM01258	LEFIA226-10	HM396569	BOLD:AAA6343	Finland	University of Oulu
Falcaria lacertinaria	TLMF Lep 09224	PHLAI661-13	KM572593	BOLD:AAA6343	Austria	Tiroler Landesmuseum Ferdinandeum
Falcaria lacertinaria	MM08167	LEFIE052-10	HM873800	BOLD:AAA6343	Finland	University of Oulu
Falcaria lacertinaria	MM01366	LEFIA319-10	HM386662	BOLD:AAA6343	Finland	University of Oulu
Falseuncaria ruficiliana	TLMF Lep 07514	PHLAG835-12	KM572684	BOLD:AAD2822	Austria	Tiroler Landesmuseum Ferdinandeum
Falseuncaria ruficiliana	MM13300	LEFIF893-10	HM875575	BOLD:AAD2822	Finland	University of Oulu
Falseuncaria ruficiliana	TLMF Lep 08046	PHLAV227-12	KM572584	BOLD:AAD2822	Austria	inatura, Dornbirn
Falseuncaria ruficiliana	MM09521	LEFIE607-10	HM874330	BOLD:AAD2822	Finland	University of Oulu
Falseuncaria ruficiliana	MM18278	LEFIK703-10	JF854268	BOLD:AAD2822	Finland	University of Oulu
Furcula furcula	MM15844	LEFIG980-10	HM876619	BOLD:ACF2827	Finland	University of Oulu
Furcula furcula	MM07680	LEFID933-10	HM873683	BOLD:ACF2827	Finland	University of Oulu
Furcula furcula	TLMF Lep 04655	PHLAE340-11	JN274481	BOLD:ACF2827	Austria	Tiroler Landesmuseum Ferdinandeum
Furcula furcula	MM15845	LEFIG981-10	HM876620	BOLD:ACF2827	Finland	University of Oulu
Gagitodes sagittata	MM11603	LEFIF373-10	HM875058	BOLD:AAD8985	Finland	University of Oulu

Gagitodes sagittata	TLMF Lep 09415	PHLAI853-13	KM572442	BOLD:AAD8985	Austria	Tiroler Landesmuseum Ferdinandeum
Gagitodes sagittata	MM06808	LEFID747-10	HM873504	BOLD:AAD8985	Finland	University of Oulu
Gagitodes sagittata	MM06750	LEFID697-10	HM873458	BOLD:AAD8985	Finland	University of Oulu
Gandaritis pyraliata	TLMF Lep 08163	PHLAV344-12	KM572390	BOLD:AAB5983	Austria	inatura, Dornbirn
Gandaritis pyraliata	MM03583	LEFIC209-10	HM872053	BOLD:AAB5983	Finland	University of Oulu
Gandaritis pyraliata	MM18427	LEFIK852-10	KM572781	BOLD:AAB5983	Finland	University of Oulu
Gandaritis pyraliata	MM12812	LEFIF726-10	HM875410	BOLD:AAB5983	Finland	University of Oulu
Gazoryctra ganna	TLMF Lep 03061	PHLAD076-11	JN307292	BOLD:AAI8414	Austria	Tiroler Landesmuseum Ferdinandeum
Gazoryctra ganna	MM12506	LEFIF618-10	HM875302	BOLD:AAI8414	Finland	University of Oulu
Gazoryctra ganna	MM12505	LEFIF617-10	HM875301	BOLD:AAI8414	Finland	University of Oulu
Gazoryctra ganna	MM17517	LEFIJ892-10	JX034573	BOLD:AAI8414	Finland	University of Oulu
Gelechia muscosella	MM06662	LEFID638-10	HM873401	BOLD:AAC8632	Finland	University of Oulu
Gelechia muscosella	MM02346	LEFIB615-10	HM871494	BOLD:AAC8632	Finland	University of Oulu
Gelechia muscosella	MM06701	LEFID664-10	HM873426	BOLD:AAC8632	Finland	University of Oulu
Gelechia muscosella	MM06698	LEFID663-10	HM873425	BOLD:AAC8632	Finland	University of Oulu
Gelechia muscosella	MM06540	LEFID551-10	HM873316	BOLD:AAC8632	Finland	University of Oulu
Gelechia muscosella	TLMF Lep 08263	PHLAH444-12	KM572128	BOLD:AAC8632	Austria	inatura, Dornbirn
Gelechia muscosella	TLMF Lep 07530	PHLAG851-12	KM572074	BOLD:AAC8632	Austria	Tiroler Landesmuseum Ferdinandeum
Gelechia muscosella	MM13875	LEFIA947-10	HM387079	BOLD:AAC8632	Finland	University of Oulu
Gelechia muscosella	MM09807	LEFIE717-10	HM874437	BOLD:AAC8632	Finland	University of Oulu
Gelechia muscosella	MM03478	LEFIC135-10	HM871981	BOLD:AAC8632	Finland	University of Oulu
Gelechia sabinellus	MM13511	LEFIF949-10	HM875630	BOLD:AAD7093	Finland	University of Oulu
Gelechia sabinellus	MM06122	LEFID257-10	HM873055	BOLD:AAD7093	Finland	University of Oulu
Gelechia sabinellus	TLMF Lep 08469	PHLAH650-12	KM573428	BOLD:AAD7093	Austria	inatura, Dornbirn
Gelechia sabinellus	MM17576	LEFIK001-10	KM373633	BOLD:AAD7093	Finland	University of Oulu
Gelechia sabinellus	MM17575	LEFIJ950-10	KM373604	BOLD:AAD7093	Finland	University of Oulu
Gelechia sabinellus	MM21089	LEFIJ1229-11	KM373626	BOLD:AAD7093	Finland	University of Oulu
Gelechia sestertiella	TLMF Lep 07708	PHLAH269-12	KM572266	BOLD:AAI7031	Austria	Tiroler Landesmuseum Ferdinandeum
Gelechia sestertiella	MM18629	LEFIL319-10	JN270955	BOLD:AAI7031	Finland	University of Oulu
Gelechia sestertiella	MM21059	LEFIJ1199-11	KM373613	BOLD:AAI7031	Finland	University of Oulu
Gelechia sestertiella	MM14421	LEFIG398-10	HM876075	BOLD:AAI7031	Finland	University of Oulu

Gelechia sororculella	MM09008	LEFIE419-10	HM874143	BOLD:AAC8633	Finland	University of Oulu
Gelechia sororculella	MM00668	LEFIB214-10	HM871118	BOLD:AAC8633	Finland	University of Oulu
Gelechia sororculella	MM00669	LEFIB215-10	HM871119	BOLD:AAC8633	Finland	University of Oulu
Gelechia sororculella	TLMF Lep 08465	PHLAH646-12	KM573454	BOLD:AAC8633	Austria	inatura, Dornbirn
Gelechia sororculella	MM13873	LEFIA945-10	HM387078	BOLD:AAC8633	Finland	University of Oulu
Geometra papilionaria	MM01146	LEFIA1147-10	KF807366	BOLD:AAB2012	Finland	University of Oulu
Geometra papilionaria	MM01147	LEFIA170-10	HM396515	BOLD:AAB2012	Finland	University of Oulu
Geometra papilionaria	MM09837	LEFIE737-10	HM874457	BOLD:AAB2012	Finland	University of Oulu
Geometra papilionaria	MM02809	LEFIB841-10	HM871718	BOLD:AAB2012	Finland	University of Oulu
Geometra papilionaria	TLMF Lep 08775	PHLAI280-13	KM572850	BOLD:AAB2012	Austria	Tiroler Landesmuseum Ferdinandeum
Glaucopsyche alexis	MM17423	LEFIJ798-10	JF853831	BOLD:AAA5424	Finland	University of Oulu
Glaucopsyche alexis	MM03860	LEFIC345-10	HM872188	BOLD:AAA5424	Finland	University of Oulu
Glaucopsyche alexis	MM17138	LEFIJ513-10	JF853635	BOLD:AAA5424	Finland	University of Oulu
Glaucopsyche alexis	MM04237	LEFIC520-10	HM872343	BOLD:AAA5424	Finland	University of Oulu
Glaucopsyche alexis	TLMF Lep 09818	PHLAW021-13	KM573281	BOLD:AAA5424	Austria	Tiroler Landesmuseum Ferdinandeum
Gluphisia crenata	MM01014	LEFIA092-10	HM396439	BOLD:AAE0994	Finland	University of Oulu
Gluphisia crenata	TLMF Lep 05618	PHLAF448-11	KM572824	BOLD:AAE0994	Austria	Tiroler Landesmuseum Ferdinandeum
Gluphisia crenata	MM01013	LEFIA091-10	HM396438	BOLD:AAE0994	Finland	University of Oulu
Gluphisia crenata	MM09769	LEFIA892-10	HM387028	BOLD:AAE0994	Finland	University of Oulu
Glyphipterix forsterella	MM21172	LEFIJ1312-11	KM573631	BOLD:AAD7071	Finland	University of Oulu
Glyphipterix forsterella	MM21186	LEFIJ1326-11	KM573360	BOLD:AAD7071	Finland	University of Oulu
Glyphipterix forsterella	MM21166	LEFIJ1306-11	KM572035	BOLD:AAD7071	Finland	University of Oulu
Glyphipterix forsterella	MM23200	COLFF467-13	KM572886	BOLD:AAD7071	Finland	University of Oulu, Zoological Museum
Glyphipterix forsterella	TLMF Lep 12491	LEATC509-13	KM572642	BOLD:AAD7071	Austria	Tiroler Landesmuseum Ferdinandeum
Glyphipterix forsterella	MM04000	LEFIC442-10	HM872276	BOLD:AAD7072	Finland	University of Oulu
Glyphipterix forsterella	MM15546	LEFIG682-10	HM876342	BOLD:AAD7072	Finland	University of Oulu
Glyphipterix forsterella	MM15545	LEFIG681-10	HM876341	BOLD:AAD7072	Finland	University of Oulu
Glyphipterix thrasonella	MM09594	LEFIE643-10	HM874366	BOLD:AAD6735	Finland	University of Oulu
Glyphipterix thrasonella	MM14262	LEFIG303-10	HM875982	BOLD:AAD6735	Finland	University of Oulu
Glyphipterix thrasonella	MM09794	LEFIA916-10	HM387052	BOLD:AAD6735	Finland	University of Oulu

Glyphipterix thrasonella	TLMF Lep 09204	PHLAI642-13	KM573048	BOLD:AAD6735	Austria	Tiroler Landesmuseum Ferdinandeum
Gnophos obfuscata	MM12883	LEFIF740-10	HM875424	BOLD:ABZ5475	Finland	University of Oulu
Gnophos obfuscata	MM15843	LEFIG979-10	HM876618	BOLD:ABZ5475	Finland	University of Oulu
Gnophos obfuscata	MM15842	LEFIG978-10	HM876617	BOLD:ABZ5475	Finland	University of Oulu
Gnophos obfuscata	TLMF Lep 00310	PHLAA270-09	HM425807	BOLD:ABZ5475	Austria	Tiroler Landesmuseum Ferdinandeum
Gnorimoschema epithymella	MM10325	LEFIE937-10	HM874654	BOLD:AAE7091	Finland	University of Oulu
Gnorimoschema epithymella	MM03091	LEFIB940-10	HM871817	BOLD:AAE7091	Finland	University of Oulu
Gnorimoschema epithymella	TLMF Lep 02965	PHLAC930-10	JF860399	BOLD:AAE7091	Austria	Tiroler Landesmuseum Ferdinandeum
Gnorimoschema epithymella	TLMF Lep 02964	PHLAC929-10	JF860398	BOLD:AAE7091	Austria	Tiroler Landesmuseum Ferdinandeum
Gnorimoschema epithymella	TLMF Lep 02963	PHLAC928-10	JF860397	BOLD:AAE7091	Austria	Tiroler Landesmuseum Ferdinandeum
Gnorimoschema epithymella	MM06043	LEFID213-10	HM873015	BOLD:AAE7091	Finland	University of Oulu
Gonepteryx rhamni	TLMF Lep 09810	PHLAW013-13	KM572370	BOLD:AAA9222	Austria	Tiroler Landesmuseum Ferdinandeum
Gonepteryx rhamni	MM17124	LEFIJ499-10	JF853623	BOLD:AAA9222	Finland	University of Oulu
Gonepteryx rhamni	MM00941	LEFIB372-10	HM871271	BOLD:AAA9222	Finland	University of Oulu
Gonepteryx rhamni	MM06996	LEFID863-10	HM873620	BOLD:AAA9222	Finland	University of Oulu
Gracillaria syringella	MM00030	LEFIA1151-10	GU828581	BOLD:AAC0054	Finland	University of Oulu
Gracillaria syringella	CNCLEP00020443	LNEL247-06	KM573334	BOLD:AAC0054	Finland	Canadian National Collection of Insects, Arachnids and Nematodes
Gracillaria syringella	TLMF Lep 07944	PHLAV125-12	KM572965	BOLD:AAC0054	Austria	inatura, Dornbirn
Gracillaria syringella	MM12451	LEFIF593-10	HM875277	BOLD:AAC0054	Finland	University of Oulu
Gracillaria syringella	TLMF Lep 07945	PHLAV126-12	KM571971	BOLD:AAC0054	Austria	inatura, Dornbirn
Gracillaria syringella	TLMF Lep 09205	PHLAI643-13	KM572587	BOLD:AAC0054	Austria	Tiroler Landesmuseum Ferdinandeum
Gracillaria syringella	MM12012	LEFIF473-10	HM875158	BOLD:AAC0054	Finland	University of Oulu
Gracillaria syringella	MM00872	LEFIB331-10	HM871231	BOLD:AAC0054	Finland	University of Oulu
Graphiphora augur	MM04870	LEFIC755-10	HM872574	BOLD:ACF0935	Finland	University of Oulu

Graphiphora augur	MM08074	LEFIE019-10	HM873768	BOLD:ACF0935	Finland	University of Oulu
Graphiphora augur	MM02757	LEFIB820-10	HM871697	BOLD:ACF0935	Finland	University of Oulu
Graphiphora augur	TLMF Lep 06100	PHLSA645-11	KM573629	BOLD:ACF0935	Austria	Tiroler Landesmuseum Ferdinandeum
Grapholita lobarzewskii	TLMF Lep 08042	PHLAV223-12	KM572239	BOLD:AAP8428	Austria	inatura, Dornbirn
Grapholita lobarzewskii	MM18893	LEFIL595-10	KM572115	BOLD:AAP8428	Finland	University of Oulu
Grapholita tenebrosana	MM17897	LEFIK322-10	KM572199	BOLD:AAD6968	Finland	University of Oulu
Grapholita tenebrosana	MM17938	LEFIK363-10	KM572353	BOLD:AAD6968	Finland	University of Oulu
Grapholita tenebrosana	MM06873	LEFID797-10	HM873554	BOLD:AAD6968	Finland	University of Oulu
Grapholita tenebrosana	MM17898	LEFIK323-10	JF853969	BOLD:AAD6968	Finland	University of Oulu
Grapholita tenebrosana	MM17900	LEFIK325-10	JF853971	BOLD:AAD6968	Finland	University of Oulu
Grapholita tenebrosana	TLMF Lep 08097	PHLAV278-12	KM572610	BOLD:AAD6968	Austria	inatura, Dornbirn
Grapholita tenebrosana	MM06874	LEFID798-10	HM873555	BOLD:AAD6968	Finland	University of Oulu
Grapholita tenebrosana	MM06472	LEFID506-10	HM873271	BOLD:AAD6968	Finland	University of Oulu
Grapholita tenebrosana	MM18612	LEFII185-10	JF853394	BOLD:AAD6968	Finland	University of Oulu
Grapholita tenebrosana	MM10360	LEFIE957-10	HM874674	BOLD:AAD6968	Finland	University of Oulu
Grapholita tenebrosana	MM21170	LEFIJ1310-11	KM572690	BOLD:AAD6968	Finland	University of Oulu
Grapholita tenebrosana	MM17323	LEFIJ698-10	KM572840	BOLD:AAD6968	Finland	University of Oulu
Grapholita tenebrosana	MM17268	LEFIJ643-10	JF853740	BOLD:AAD6968	Finland	University of Oulu
Grapholita tenebrosana	MM11042	LEFIF260-10	HM874953	BOLD:AAD6968	Finland	University of Oulu
Grapholita tenebrosana	MM16014	LEFIL725-10	KM572988	BOLD:AAD6968	Finland	University of Oulu
Grapholita tenebrosana	MM11041	LEFIF259-10	HM874952	BOLD:AAD6968	Finland	University of Oulu
Grapholita tenebrosana	MM22900	LEFIJ2048-13	KM572232	BOLD:AAD6968	Finland	University of Oulu
Grapholita tenebrosana	MM17896	LEFIK321-10	JF853968	BOLD:AAD6968	Finland	University of Oulu
Grapholita tenebrosana	MM17899	LEFIK324-10	JF853970	BOLD:AAL3224	Finland	University of Oulu
Grapholita tenebrosana	MM05511	LEFID046-10	HM872860	BOLD:AAL3224	Finland	University of Oulu
Grapholita tenebrosana	MM17901	LEFIK326-10	JF853972	BOLD:AAL3224	Finland	University of Oulu
Grapholita tenebrosana	MM17269	LEFIJ644-10	JF853741	BOLD:AAL3224	Finland	University of Oulu
Grapholita tenebrosana	MM11043	LEFIF261-10	HM874954	BOLD:AAL3224	Finland	University of Oulu
Grapholita tenebrosana	MM02430	LEFIB658-10	HM871536	BOLD:AAL3224	Finland	University of Oulu
Grapholita tenebrosana	MM16013	LEFIL724-10	KM572601	BOLD:AAL3224	Finland	University of Oulu
Grapholita tenebrosana	MM16012	LEFIL723-10	JF854701	BOLD:AAL3224	Finland	University of Oulu

Grapholita tenebrosana	MM11040	LEFIF258-10	HM874951	BOLD:AAL3224	Finland	University of Oulu
Griposia aprilina	MM18037	LEFIK462-10	KM573025	BOLD:AAC3647	Finland	University of Oulu
Griposia aprilina	MM04757	LEFIC713-10	HM872534	BOLD:AAC3647	Finland	University of Oulu
Griposia aprilina	TLMF Lep 08784	PHLAI289-13	KM572526	BOLD:AAC3647	Austria	Tiroler Landesmuseum Ferdinandeum
Griposia aprilina	MM04803	LEFIC727-10	HM872548	BOLD:AAC3647	Finland	University of Oulu
Gymnoscelis rufifasciata	TLMF Lep 08164	PHLAV345-12	KM573498	BOLD:AAA7404	Austria	inatura, Dornbirn
Gymnoscelis rufifasciata	MM01823	LEFIA683-10	HM386829	BOLD:AAA7404	Finland	University of Oulu
Gymnoscelis rufifasciata	MM01824	LEFIA684-10	HM386830	BOLD:AAA7404	Finland	University of Oulu
Gymnoscelis rufifasciata	MM12960	LEFIF751-10	HM875435	BOLD:AAA7404	Finland	University of Oulu
Gynnidomorpha alismana	MM17336	LEFIJ711-10	JF853786	BOLD:AAF7913	Finland	University of Oulu
Gynnidomorpha alismana	MM18632	LEFIL322-10	JN286439	BOLD:AAF7913	Finland	University of Oulu
Gynnidomorpha alismana	MM19266	LEFIL266-10	JQ775256	BOLD:AAF7913	Finland	University of Oulu
Gynnidomorpha alismana	MM19265	LEFIL265-10	JQ775255	BOLD:AAF7913	Finland	University of Oulu
Gynnidomorpha alismana	TLMF Lep 08448	PHLAH629-12	KM572967	BOLD:ABY4645	Austria	inatura, Dornbirn
Gypsonoma dealbana	MM02149	LEFIB537-10	HM871423	BOLD:AAB0380	Finland	University of Oulu
Gypsonoma dealbana	MM09819	LEFIE725-10	HM874445	BOLD:AAB0380	Finland	University of Oulu
Gypsonoma dealbana	MM03482	LEFIC138-10	HM871984	BOLD:AAB0380	Finland	University of Oulu
Gypsonoma dealbana	TLMF Lep 08044	PHLAV225-12	KM573211	BOLD:AAC8347	Austria	inatura, Dornbirn
Gypsonoma dealbana	TLMF Lep 08251	PHLAH432-12	KM572595	BOLD:AAC8347	Austria	inatura, Dornbirn
Gypsonoma sociana	MM02154	LEFIB541-10	HM871426	BOLD:AAA6642	Finland	University of Oulu
Gypsonoma sociana	MM06139	LEFID268-10	HM873066	BOLD:AAA6642	Finland	University of Oulu
Gypsonoma sociana	MM09722	LEFIA846-10	HM386986	BOLD:AAA6642	Finland	University of Oulu
Gypsonoma sociana	MM09717	LEFIA842-10	HM386982	BOLD:AAA6642	Finland	University of Oulu
Gypsonoma sociana	TLMF Lep 09394	PHLAI832-13	KM572003	BOLD:AAA6642	Austria	Tiroler Landesmuseum Ferdinandeum
Gypsonoma sociana	MM03199	LEFIB999-10	HM871870	BOLD:AAA6642	Finland	University of Oulu
Habrosyne pyritoides	MM17427	LEFIJ802-10	KM571958	BOLD:AAC5831	Finland	University of Oulu
Habrosyne pyritoides	TLMF Lep 07902	PHLAV083-12	KM573362	BOLD:AAC5831	Austria	inatura, Dornbirn
Habrosyne pyritoides	MM17426	LEFIJ801-10	KM572311	BOLD:AAC5831	Finland	University of Oulu
Habrosyne pyritoides	MM18671	LEFIL361-10	KM572498	BOLD:AAC5831	Finland	University of Oulu
Hada plebeja	MM04732	LEFIC705-10	HM872526	BOLD:AAC2435	Finland	University of Oulu
Hada plebeja	MM01586	LEFIA504-10	KM572551	BOLD:AAC2435	Finland	University of Oulu

Hada plebeja	MM04098	LEFIA744-10	HM386888	BOLD:AAC2435	Finland	University of Oulu
Hada plebeja	MM01587	LEFIA505-10	KM572121	BOLD:AAC2435	Finland	University of Oulu
Hada plebeja	TLMF Lep 04663	PHLAE348-11	JN272313	BOLD:AAC2435	Austria	Tiroler Landesmuseum Ferdinandeum
Hadena albimacula	MM18021	LEFIK446-10	KM573365	BOLD:AAF8086	Finland	University of Oulu
Hadena albimacula	MM00656	LEFIB206-10	HM871110	BOLD:AAF8086	Finland	University of Oulu
Hadena albimacula	TLMF Lep 04674	PHLAE359-11	JN284172	BOLD:AAF8086	Austria	Tiroler Landesmuseum Ferdinandeum
Hadena albimacula	MM15889	LEFIH025-10	HM876661	BOLD:AAF8086	Finland	University of Oulu
Hadena perplexa	MM04737	LEFIC708-10	HM872529	BOLD:AAC7968	Finland	University of Oulu
Hadena perplexa	MM12635	LEFIF670-10	HM875354	BOLD:AAC7968	Finland	University of Oulu
Hadena perplexa	TLMF Lep 04672	PHLAE357-11	JN284171	BOLD:AAC7968	Austria	Tiroler Landesmuseum Ferdinandeum
Hadena perplexa	MM00658	LEFIB208-10	HM871112	BOLD:AAC7968	Finland	University of Oulu
Hadena perplexa	TLMF Lep 01139	PHLAB339-10	HQ968501	BOLD:AAC7968	Austria	Tiroler Landesmuseum Ferdinandeum
Harpella forficella	MM10381	LEFIE967-10	HM874684	BOLD:AAE6991	Finland	University of Oulu
Harpella forficella	TLMF Lep 08216	PHLAH397-12	KM572605	BOLD:AAE6991	Austria	inatura, Dornbirn
Harpella forficella	MM09485	LEFIE583-10	HM874306	BOLD:AAE6991	Finland	University of Oulu
Harpella forficella	MM14390	LEFIG381-10	HM876058	BOLD:AAE6991	Finland	University of Oulu
Harpella forficella	MM06866	LEFIA1157-10	JF818753	BOLD:AAE6991	Finland	University of Oulu
Hecatera bicolorata	MM10058	LEFIE839-10	HM874558	BOLD:AAD3287	Finland	University of Oulu
Hecatera bicolorata	MM02382	LEFIB631-10	HM871510	BOLD:AAD3287	Finland	University of Oulu
Hecatera bicolorata	TLMF Lep 04607	PHLAE292-11	JN272308	BOLD:AAD3287	Austria	Tiroler Landesmuseum Ferdinandeum
Hecatera bicolorata	MM14290	LEFIG321-10	HM876000	BOLD:AAD3287	Finland	University of Oulu
Hedya nubiferana	MM13244	LEFIF867-10	HM875549	BOLD:AAA4552	Finland	University of Oulu
Hedya nubiferana	TLMF Lep 09926	PHLAW129-13	KM572600	BOLD:AAA4552	Austria	inatura, Dornbirn
Hedya nubiferana	MM05336	LEFIC961-10	HM872776	BOLD:AAA4552	Finland	University of Oulu
Hedya nubiferana	MM01964	LEFIB446-10	HM871345	BOLD:AAA4552	Finland	University of Oulu
Hedya ochroleucana	MM05238	LEFIC909-10	HM872726	BOLD:AAC0586	Finland	University of Oulu
Hedya ochroleucana	MM05237	LEFIC908-10	HM872725	BOLD:AAC0586	Finland	University of Oulu
Hedya ochroleucana	TLMF Lep 08022	PHLAV203-12	KM572397	BOLD:AAC0586	Austria	inatura, Dornbirn
Hedya ochroleucana	MM05003	LEFIC820-10	HM872639	BOLD:AAC0586	Finland	University of Oulu
Hedya pruniana	MM09753	LEFIA876-10	HM387014	BOLD:AAD0577	Finland	University of Oulu
Hedya pruniana	TLMF Lep 09934	PHLAW137-13	KM573180	BOLD:AAD0577	Austria	inatura, Dornbirn

Hedya pruniana	MM18302	LEFIK727-10	JF854289	BOLD:AAD0577	Finland	University of Oulu
Hedya pruniana	MM18301	LEFIK726-10	JF854288	BOLD:AAD0577	Finland	University of Oulu
Helcystogramma rufescens	MM03080	LEFIB934-10	HM871811	BOLD:AAC1177	Finland	University of Oulu
Helcystogramma rufescens	TLMF Lep 07527	PHLAG848-12	KM572389	BOLD:AAC1177	Austria	Tiroler Landesmuseum Ferdinandeum
Helcystogramma rufescens	TLMF Lep 08041	PHLAV222-12	KM573111	BOLD:AAC1177	Austria	inatura, Dornbirn
Helcystogramma rufescens	TLMF Lep 04540	PHLAE415-11	KM572195	BOLD:AAC1177	Austria	Tiroler Landesmuseum Ferdinandeum
Helcystogramma rufescens	MM08862	LEFIE370-10	HM874094	BOLD:AAC1177	Finland	University of Oulu
Helcystogramma rufescens	MM11981	LEFIF463-10	HM875148	BOLD:AAC1177	Finland	University of Oulu
Helcystogramma rufescens	MM03841	LEFIC334-10	HM872178	BOLD:AAC1177	Finland	University of Oulu
Helcystogramma rufescens	MM05274	LEFIC925-10	HM872742	BOLD:AAC1177	Finland	University of Oulu
Helcystogramma rufescens	MM02273	LEFIB584-10	HM871464	BOLD:AAC1177	Finland	University of Oulu
Helcystogramma rufescens	MM02274	LEFIB585-10	HM871465	BOLD:AAC1177	Finland	University of Oulu
Heliothis nubigera	TLMF Lep 04620	PHLAE305-11	JN272575	BOLD:AAI6741	Austria	Tiroler Landesmuseum Ferdinandeum
Heliothis nubigera	MM11113	LEFIF323-10	HM875008	BOLD:AAI6741	Finland	University of Oulu
Heliothis nubigera	TLMF Lep 06174	PHLSA719-11	KM573143	BOLD:AAI6741	Austria	Tiroler Landesmuseum Ferdinandeum
Heliothis peltigera	TLMF Lep 04621	PHLAE306-11	JN272576	BOLD:AAC6990	Austria	Tiroler Landesmuseum Ferdinandeum
Heliothis peltigera	MM15938	LEFIJ338-10	KM572408	BOLD:AAC6990	Finland	University of Oulu
Hellinsia osteodactylus	MM08472	LEFIE181-10	HQ570360	BOLD:AAD2930	Finland	University of Oulu
Hellinsia osteodactylus	TLMF Lep 04287	PHLAE067-11	JN277241	BOLD:AAD2930	Austria	Tiroler Landesmuseum Ferdinandeum
Hellinsia osteodactylus	MM13383	LEFIF937-10	HM875619	BOLD:AAD2930	Finland	University of Oulu
Hellinsia osteodactylus	MM06357	LEFID427-10	HQ570323	BOLD:AAD2930	Finland	University of Oulu
Hellinsia osteodactylus	MM03105	LEFIB948-10	HM871825	BOLD:AAD2930	Finland	University of Oulu

Hellinsia tephradactyla	MM03104	LEFIB947-10	HM871824	BOLD:AAE6943	Finland	University of Oulu
Hellinsia tephradactyla	TLMF Lep 08396	PHLAH577-12	KM572996	BOLD:AAE6943	Austria	inatura, Dornbirn
Hellinsia tephradactyla	MM18377	LEFIK802-10	JN277225	BOLD:AAE6943	Finland	University of Oulu
Hellinsia tephradactyla	BIOUG04116-B08	GMFIF759-12	KM572880	BOLD:AAE6943	Finland	Biodiversity Institute of Ontario
Hellinsia tephradactyla	MM03103	LEFIB946-10	HM871823	BOLD:AAE6943	Finland	University of Oulu
Helotropha leucostigma	TLMF Lep 08536	PHLAH717-12	KM572467	BOLD:ACE3288	Austria	inatura, Dornbirn
Helotropha leucostigma	MM01743	LEFIA628-10	HM870877	BOLD:ACE3288	Finland	University of Oulu
Helotropha leucostigma	MM07767	LEFID947-10	HM873697	BOLD:ACE3288	Finland	University of Oulu
Helotropha leucostigma	MM04651	LEFIC681-10	HM872502	BOLD:ACE3288	Finland	University of Oulu
Hemaris fuciformis	MM05477	LEFID032-10	HM872846	BOLD:AAB5591	Finland	University of Oulu
Hemaris fuciformis	MM17191	LEFIJ566-10	JF853681	BOLD:AAB5591	Finland	University of Oulu
Hemaris fuciformis	MM17192	LEFIJ567-10	KM572614	BOLD:AAB5591	Finland	University of Oulu
Hemaris fuciformis	TLMF Lep 09848	PHLAW051-13	KM573525	BOLD:AAB5591	Austria	Tiroler Landesmuseum Ferdinandeum
Hemaris tityus	TLMF Lep 09857	PHLAW060-13	KM573075	BOLD:ACE7540	Austria	Tiroler Landesmuseum Ferdinandeum
Hemaris tityus	MM08732	LEFIE338-10	HM874063	BOLD:ACE7540	Finland	University of Oulu
Hemaris tityus	MM17190	LEFIJ565-10	JF853680	BOLD:ACE7540	Finland	University of Oulu
Hemaris tityus	MM08731	LEFIE337-10	HM874062	BOLD:ACE7540	Finland	University of Oulu
Hemithea aestivaria	MM06098	LEFID237-10	HM873037	BOLD:AAA4522	Finland	University of Oulu
Hemithea aestivaria	MM10875	LEFIF209-10	HM874903	BOLD:AAA4522	Finland	University of Oulu
Hemithea aestivaria	TLMF Lep 08192	PHLAV373-12	KF807214	BOLD:AAA4522	Austria	inatura, Dornbirn
Hemithea aestivaria	MM10874	LEFIF208-10	HM874902	BOLD:AAA4522	Finland	University of Oulu
Hepialus humuli	TLMF Lep 05134	PHLAE629-11	KC970359	BOLD:AAC3448	Austria	Tiroler Landesmuseum Ferdinandeum
Hepialus humuli	TLMF Lep 05133	PHLAE628-11	KC970367	BOLD:AAC3448	Austria	Tiroler Landesmuseum Ferdinandeum
Hepialus humuli	MM01062	LEFIA131-10	HM396478	BOLD:AAC3448	Finland	University of Oulu
Hepialus humuli	MM01061	LEFIA130-10	HM396477	BOLD:AAC3448	Finland	University of Oulu
Hepialus humuli	TLMF Lep 05143	PHLAE638-11	KC970358	BOLD:AAC3448	Austria	Tiroler Landesmuseum Ferdinandeum
Hepialus humuli	TLMF Lep 05131	PHLAE626-11	KC970357	BOLD:AAC3448	Austria	Tiroler Landesmuseum Ferdinandeum
Hepialus humuli	TLMF Lep 05132	PHLAE627-11	KC970351	BOLD:AAC3448	Austria	Tiroler Landesmuseum Ferdinandeum
Hepialus humuli	TLMF Lep 05154	PHLAE649-11	KC970363	BOLD:AAC3448	Austria	Tiroler Landesmuseum Ferdinandeum
Hepialus humuli	TLMF Lep 05141	PHLAE636-11	KC970343	BOLD:AAC3448	Austria	Tiroler Landesmuseum Ferdinandeum
Hepialus humuli	MM14287	LEFIG319-10	HM875998	BOLD:AAC3448	Finland	University of Oulu

Hepialus humuli	TLMF Lep 05142	PHLAE637-11	KC970341	BOLD:AAC3448	Austria	Tiroler Landesmuseum Ferdinandeum
Hepialus humuli	TLMF Lep 05144	PHLAE639-11	KC970342	BOLD:AAT9578	Austria	Tiroler Landesmuseum Ferdinandeum
Hepialus humuli	TLMF Lep 05145	PHLAE640-11	KC970366	BOLD:AAT9578	Austria	Tiroler Landesmuseum Ferdinandeum
Hepialus humuli	TLMF Lep 04039	PHLAD674-11	JN307357	BOLD:AAT9578	Austria	Tiroler Landesmuseum Ferdinandeum
Hepialus humuli	TLMF Lep 05136	PHLAE631-11	KC970346	BOLD:ACE4469	Austria	Tiroler Landesmuseum Ferdinandeum
Hepialus humuli	TLMF Lep 05135	PHLAE630-11	KC970347	BOLD:ACE4469	Austria	Tiroler Landesmuseum Ferdinandeum
Heringocrania unimaculella	TLMF Lep 09897	PHLAW100-13	KM572851	BOLD:AAF3179	Austria	Tiroler Landesmuseum Ferdinandeum
Heringocrania unimaculella	MM08800	LEFIE361-10	HM874085	BOLD:AAF3179	Finland	University of Oulu
Heringocrania unimaculella	MM00433	LEFIA034-10	HM396384	BOLD:AAF3179	Finland	University of Oulu
Heringocrania unimaculella	MM17911	LEFIK336-10	JF853977	BOLD:AAF3179	Finland	University of Oulu
Herminia grisealis	MM04679	LEFIC695-10	HM872516	BOLD:AAC3337	Finland	University of Oulu
Herminia grisealis	TLMF Lep 08166	PHLAV347-12	KM573464	BOLD:AAC3337	Austria	inatura, Dornbirn
Herminia grisealis	MM01421	LEFIA365-10	HM386707	BOLD:AAC3337	Finland	University of Oulu
Herminia grisealis	MM01422	LEFIA366-10	HM386708	BOLD:AAC3337	Finland	University of Oulu
Herminia grisealis	TLMF Lep 04646	PHLAE331-11	JN273333	BOLD:AAC3337	Austria	Tiroler Landesmuseum Ferdinandeum
Herminia tarsicrinalis	MM11056	LEFIF274-10	HM874967	BOLD:AAC1537	Finland	University of Oulu
Herminia tarsicrinalis	TLMF Lep 08125	PHLAV306-12	KM573619	BOLD:AAC1537	Austria	inatura, Dornbirn
Herminia tarsicrinalis	MM17220	LEFIJ595-10	JF853695	BOLD:AAC1537	Finland	University of Oulu
Herminia tarsipennalis	MM01418	LEFIA362-10	HM386704	BOLD:AAC1538	Finland	University of Oulu
Herminia tarsipennalis	MM18520	LEFIK945-10	JF854384	BOLD:AAC1538	Finland	University of Oulu
Herminia tarsipennalis	MM04587	LEFIC641-10	HM872462	BOLD:AAC1538	Finland	University of Oulu
Herminia tarsipennalis	TLMF Lep 08124	PHLAV305-12	KM572529	BOLD:AAC1538	Austria	inatura, Dornbirn
Hesperia comma	MM17115	LEFIJ490-10	JF853615	BOLD:ABX6141	Finland	University of Oulu
Hesperia comma	TLMF Lep 09102	PHLAI540-13	KM573144	BOLD:ABX6141	Austria	Tiroler Landesmuseum Ferdinandeum
Hesperia comma	MM06896	LEFID810-10	HM873567	BOLD:ABX6141	Finland	University of Oulu
Hesperia comma	MM17114	LEFIJ489-10	JF853614	BOLD:ABX6141	Finland	University of Oulu
Hofmannophila pseudospretella	TLMF Lep 07525	PHLAG846-12	KM572377	BOLD:AAB0831	Austria	Tiroler Landesmuseum Ferdinandeum

Hofmannophila pseudospretella	MM04345	LEFIC567-10	HM872388	BOLD:AAB0831	Finland	University of Oulu
Hofmannophila pseudospretella	TLMF Lep 08231	PHLAH412-12	KM573661	BOLD:AAB0831	Austria	inatura, Dornbirn
Hofmannophila pseudospretella	MM10373	LEFIE965-10	HM874682	BOLD:AAB0831	Finland	University of Oulu
Hofmannophila pseudospretella	MM10372	LEFIE964-10	HM874681	BOLD:AAB0831	Finland	University of Oulu
Hoplodrina blanda	MM17365	LEFIJ740-10	KM572665	BOLD:AAC0362	Finland	University of Oulu
Hoplodrina blanda	TLMF Lep 08510	PHLAH691-12	KM572739	BOLD:AAC0362	Austria	inatura, Dornbirn
Hoplodrina blanda	MM05130	LEFIC866-10	HM872684	BOLD:AAC0362	Finland	University of Oulu
Hoplodrina blanda	MM18046	LEFIK471-10	JF854082	BOLD:AAC0362	Finland	University of Oulu
Hoplodrina blanda	MM18045	LEFIK470-10	KM573470	BOLD:AAC0362	Finland	University of Oulu
Hoplodrina octogenaria	MM12720	LEFIF693-10	HM875377	BOLD:AAB4763	Finland	University of Oulu
Hoplodrina octogenaria	TLMF Lep 08140	PHLAV321-12	KM573255	BOLD:AAB4763	Austria	inatura, Dornbirn
Hoplodrina octogenaria	MM18047	LEFIK472-10	JF854083	BOLD:AAB4763	Finland	University of Oulu
Hoplodrina octogenaria	MM17367	LEFIJ742-10	KM573391	BOLD:AAB4763	Finland	University of Oulu
Hoplodrina octogenaria	MM17366	LEFIJ741-10	KM573307	BOLD:AAB4763	Finland	University of Oulu
Hoplodrina octogenaria	MM04900	LEFIC772-10	HM872591	BOLD:AAB4763	Finland	University of Oulu
Hoplodrina octogenaria	MM01651	LEFIA1175-10	KM572364	BOLD:AAB4763	Finland	University of Oulu
Hoplodrina octogenaria	MM07463	LEFID932-10	HM873682	BOLD:AAB4763	Finland	University of Oulu
Hoplodrina octogenaria	TLMF Lep 05615	PHLAF445-11	KM572162	BOLD:AAB4763	Austria	Tiroler Landesmuseum Ferdinandeum
Hoplodrina octogenaria	MM01746	LEFIA631-10	HM870880	BOLD:AAB4763	Finland	University of Oulu
Hoplodrina octogenaria	MM01652	LEFIA558-10	KM572633	BOLD:AAB4763	Finland	University of Oulu
Horisme tersata	TLMF Lep 08170	PHLAV351-12	KM572844	BOLD:AAC5135	Austria	inatura, Dornbirn
Horisme tersata	MM12781	LEFIF719-10	HM875403	BOLD:AAC5135	Finland	University of Oulu
Horisme tersata	MM03863	LEFIC347-10	HM872190	BOLD:AAC5135	Finland	University of Oulu
Horisme tersata	MM14251	LEFIG295-10	HM875974	BOLD:AAC5135	Finland	University of Oulu
Hydraecia micacea	MM07283	LEFID908-10	HM873658	BOLD:AAB1631	Finland	University of Oulu
Hydraecia micacea	MM01723	LEFIA611-10	HM870860	BOLD:AAB1631	Finland	University of Oulu
Hydraecia micacea	MM01722	LEFIA610-10	HM870859	BOLD:AAB1631	Finland	University of Oulu

Hydraecia micacea	MM04352	LEFIC573-10	HM872394	BOLD:AAB1631	Finland	University of Oulu
Hydraecia micacea	TLMF Lep 08533	PHLAH714-12	KM573177	BOLD:AAB1631	Austria	inatura, Dornbirn
Hydraecia micacea	MM12715	LEFIF691-10	HM875375	BOLD:AAB1631	Finland	University of Oulu
Hydraecia micacea	MM08012	LEFID980-10	HM873730	BOLD:AAB1631	Finland	University of Oulu
Hydraecia micacea	MM00824	LEFIB305-10	HM871206	BOLD:AAB1631	Finland	University of Oulu
Hydraecia micacea	MM22089	LEFIJ1421-12	KM572977	BOLD:AAB1631	Finland	Research Collection of Kimmo Oesterman
Hydrelia flammeolaria	MM04553	LEFIC619-10	HM872440	BOLD:AAC5746	Finland	University of Oulu
Hydrelia flammeolaria	MM01431	LEFIA374-10	HM386716	BOLD:AAC5746	Finland	University of Oulu
Hydrelia flammeolaria	MM01430	LEFIA373-10	HM386715	BOLD:AAC5746	Finland	University of Oulu
Hydrelia flammeolaria	TLMF Lep 07868	PHLAV049-12	KM572505	BOLD:ACE6107	Austria	inatura, Dornbirn
Hydrelia sylvata	TLMF Lep 07894	PHLAV075-12	KM572325	BOLD:AAD3792	Austria	inatura, Dornbirn
Hydrelia sylvata	MM01441	LEFIA384-10	HM386726	BOLD:AAD3792	Finland	University of Oulu
Hydrelia sylvata	MM01442	LEFIA385-10	HM386727	BOLD:AAD3792	Finland	University of Oulu
Hydrelia sylvata	MM08365	LEFIE136-10	HM873884	BOLD:AAD3792	Finland	University of Oulu
Hydria cervinalis	MM04035	LEFIC453-10	HM872287	BOLD:AAC1414	Finland	University of Oulu
Hydria cervinalis	TLMF Lep 07884	PHLAV065-12	KM573286	BOLD:AAC1414	Austria	inatura, Dornbirn
Hydria cervinalis	MM18465	LEFIK890-10	JF854341	BOLD:AAC1414	Finland	University of Oulu
Hydria cervinalis	MM04034	LEFIC452-10	HM872286	BOLD:AAC1414	Finland	University of Oulu
Hydria undulata	MM01384	LEFIA337-10	HM386680	BOLD:AAA9056	Finland	University of Oulu
Hydria undulata	MM01462	LEFIA403-10	HM386745	BOLD:AAA9056	Finland	University of Oulu
Hydria undulata	MM02856	LEFIB852-10	HM871729	BOLD:AAA9056	Finland	University of Oulu
Hydria undulata	TLMF Lep 07563	PHLAG884-12	KM573482	BOLD:AAA9056	Austria	inatura, Dornbirn
Hydria undulata	MM01461	LEFIA402-10	HM386744	BOLD:AAA9056	Finland	University of Oulu
Hydriomena furcata	MM01446	LEFIA389-10	HM386731	BOLD:ACE8706	Finland	University of Oulu
Hydriomena furcata	MM01447	LEFIA390-10	HM386732	BOLD:ACE8706	Finland	University of Oulu
Hydriomena furcata	MM08123	LEFIE039-10	HM873788	BOLD:ACE8706	Finland	University of Oulu
Hydriomena furcata	TLMF Lep 06203	PHLSA748-11	KM573375	BOLD:ACE8706	Austria	Tiroler Landesmuseum Ferdinandeum
Hydriomena impluviata	TLMF Lep 04670	PHLAE355-11	JN279574	BOLD:AAB8305	Austria	Tiroler Landesmuseum Ferdinandeum
Hydriomena impluviata	MM02844	LEFIB849-10	HM871726	BOLD:ACE7346	Finland	University of Oulu
Hydriomena impluviata	MM04574	LEFIC632-10	HM872453	BOLD:ACE7346	Finland	University of Oulu

Hydriomena impluviata	MM01297	LEFIA260-10	HM386604	BOLD:ACE7346	Finland	University of Oulu
Hydriomena impluviata	MM01296	LEFIA259-10	HM386603	BOLD:ACE7346	Finland	University of Oulu
Hydriomena ruberata	MM00485	LEFIB123-10	HM871029	BOLD:AAB6494	Finland	University of Oulu
Hydriomena ruberata	MM12869	LEFIF737-10	HM875421	BOLD:AAB6494	Finland	University of Oulu
Hydriomena ruberata	MM08431	LEFIE169-10	HM873915	BOLD:AAB6494	Finland	University of Oulu
Hydriomena ruberata	TLMF Lep 04637	PHLAE322-11	JN279569	BOLD:AAB6494	Austria	Tiroler Landesmuseum Ferdinandeum
Hydriomena ruberata	MM01289	LEFIA252-10	HM386596	BOLD:AAB6494	Finland	University of Oulu
Hylaea fasciaria	MM06506	LEFID526-10	HM873291	BOLD:AAB6675	Finland	University of Oulu
Hylaea fasciaria	MM01385	LEFIA338-10	HM386681	BOLD:AAB6675	Finland	University of Oulu
Hylaea fasciaria	MM01386	LEFIA339-10	HM386682	BOLD:AAB6675	Finland	University of Oulu
Hylaea fasciaria	TLMF Lep 08132	PHLAV313-12	KM572454	BOLD:AAB6675	Austria	inatura, Dornbirn
Hyles gallii	TLMF Lep 09504	LEATA087-13	KM572858	BOLD:ACE4003	Austria	Tiroler Landesmuseum Ferdinandeum
Hyles gallii	MM12530	LEFIF633-10	HM875317	BOLD:ACE4003	Finland	University of Oulu
Hyles gallii	MM05084	LEFIA1177-10	GU828710	BOLD:ACE4003	Finland	University of Oulu
Hyles gallii	TLMF Lep 12458	LEATC476-13	KM573006	BOLD:ACE4003	Austria	Tiroler Landesmuseum Ferdinandeum
Hyles gallii	MM10703	LEFIF165-10	HM874859	BOLD:ACE4003	Finland	University of Oulu
Hypatopa binotella	MM03505	LEFIC159-10	HM872005	BOLD:AAB7100	Finland	University of Oulu
Hypatopa binotella	MM15611	LEFIG747-10	HM876403	BOLD:AAB7100	Finland	University of Oulu
Hypatopa binotella	MM00133	LEFIA1180-10	GU828611	BOLD:AAB7100	Finland	University of Oulu
Hypatopa binotella	MM08251	LEFIE084-10	HM873831	BOLD:AAB7100	Finland	University of Oulu
Hypatopa binotella	MM13538	LEFIF961-10	HM875642	BOLD:AAB7100	Finland	University of Oulu
Hypatopa binotella	TLMF Lep 08736	PHLAH932-12	KM572933	BOLD:AAB7100	Austria	Tiroler Landesmuseum Ferdinandeum
Hypatopa inunctella	TLMF Lep 08450	PHLAH631-12	KM572081	BOLD:AAE5929	Austria	inatura, Dornbirn
Hypatopa inunctella	MM06537	LEFID549-10	HM873314	BOLD:AAE5929	Finland	University of Oulu
Hypatopa inunctella	MM06538	LEFID550-10	HM873315	BOLD:AAE5929	Finland	University of Oulu
Hypena crassalis	MM04576	LEFIC634-10	HM872455	BOLD:AAD1366	Finland	University of Oulu
Hypena crassalis	MM01708	LEFIA596-10	HM870845	BOLD:AAD1366	Finland	University of Oulu
Hypena crassalis	MM01709	LEFIA597-10	HM870846	BOLD:AAD1366	Finland	University of Oulu
Hypena crassalis	MM17458	LEFIJ833-10	KM572972	BOLD:AAD1366	Finland	University of Oulu
Hypena crassalis	TLMF Lep 07578	PHLAG899-12	KM573197	BOLD:AAD1366	Austria	inatura, Dornbirn
Hypena proboscidalis	MM01545	LEFIA1181-10	GU828668	BOLD:AAB6485	Finland	University of Oulu

Hypena proboscidalis	MM02769	LEFIB826-10	HM871703	BOLD:AAB6485	Finland	University of Oulu
Hypena proboscidalis	MM01830	LEFIA689-10	HM386834	BOLD:AAB6485	Finland	University of Oulu
Hypena proboscidalis	TLMF Lep 08116	PHLAV297-12	KM572308	BOLD:AAB6485	Austria	inatura, Dornbirn
Hypena proboscidalis	MM01546	LEFIA473-10	HM386813	BOLD:AAB6485	Finland	University of Oulu
Hypercallia citrinalis	MM09742	LEFIA865-10	HM387003	BOLD:AAE1403	Finland	University of Oulu
Hypercallia citrinalis	MM00071	LEFIA1184-10	GU828596	BOLD:AAE1403	Finland	University of Oulu
Hypercallia citrinalis	MM06787	LEFID730-10	HM873487	BOLD:AAE1403	Finland	University of Oulu
Hypercallia citrinalis	MM08641	LEFIE293-10	HM874025	BOLD:AAE1403	Finland	University of Oulu
Hypercallia citrinalis	TLMF Lep 00917	PHLAB117-10	HM381489	BOLD:AAE1403	Austria	Tiroler Landesmuseum Ferdinandeum
Hypochalcia ahenella	MM16011	LEFIL722-10	KM572464	BOLD:AAD0238	Finland	University of Oulu
Hypochalcia ahenella	TLMF Lep 08001	PHLAV182-12	KM572842	BOLD:AAD0238	Austria	inatura, Dornbirn
Hypochalcia ahenella	MM14474	LEFIG438-10	HM876114	BOLD:AAD0238	Finland	University of Oulu
Hypochalcia ahenella	MM01880	LEFIB411-10	HM871310	BOLD:AAD0238	Finland	University of Oulu
Hypochalcia ahenella	MM14355	LEFIG357-10	HM876034	BOLD:AAD0238	Finland	University of Oulu
Hypomecis punctinalis	MM01281	LEFIA248-10	HM386592	BOLD:AAB1058	Finland	University of Oulu
Hypomecis punctinalis	MM01280	LEFIA247-10	HM386591	BOLD:AAB1058	Finland	University of Oulu
Hypomecis punctinalis	MM08369	LEFIE138-10	HM873886	BOLD:AAB1058	Finland	University of Oulu
Hypomecis punctinalis	TLMF Lep 07886	PHLAV067-12	KM572563	BOLD:ACA2461	Austria	inatura, Dornbirn
Hypomecis roboraria	MM01470	LEFIA408-10	HM386750	BOLD:AAC9905	Finland	University of Oulu
Hypomecis roboraria	TLMF Lep 08165	PHLAV346-12	KM572939	BOLD:AAC9905	Austria	inatura, Dornbirn
Hypomecis roboraria	MM09834	LEFIE735-10	HM874455	BOLD:AAC9905	Finland	University of Oulu
Hypomecis roboraria	MM05135	LEFIC867-10	HM872685	BOLD:AAC9905	Finland	University of Oulu
Hypoxystis pluviaria	MM17991	LEFIK416-10	JF854045	BOLD:AAD1512	Finland	University of Oulu
Hypoxystis pluviaria	MM15837	LEFIG973-10	HM876613	BOLD:AAD1512	Finland	University of Oulu
Hypoxystis pluviaria	MM01752	LEFIA637-10	HM870886	BOLD:AAD1512	Finland	University of Oulu
Hypoxystis pluviaria	MM12189	LEFIF533-10	HM875218	BOLD:AAD1512	Finland	University of Oulu
Hypoxystis pluviaria	TLMF Lep 10010	LEATA403-13	KM573707	BOLD:AAD1512	Austria	inatura, Dornbirn
Hyppa rectilinea	MM00646	LEFIB201-10	HM871105	BOLD:ABZ5210	Finland	University of Oulu
Hyppa rectilinea	TLMF Lep 00298	PHLAA258-09	HM425795	BOLD:ABZ5210	Austria	Tiroler Landesmuseum Ferdinandeum
Hyppa rectilinea	TLMF Lep 08117	PHLAV298-12	KM572013	BOLD:ABZ5210	Austria	inatura, Dornbirn
Hyppa rectilinea	MM14384	LEFIG378-10	HM876055	BOLD:ABZ5210	Finland	University of Oulu

Hyppa rectilinea	MM01655	LEFIA561-10	KM572650	BOLD:ABZ5210	Finland	University of Oulu
Hypsopygia costalis	MM11778	LEFIF408-10	HM875093	BOLD:AAA9616	Finland	University of Oulu
Hypsopygia costalis	MM01895	LEFIB418-10	HM871317	BOLD:AAA9616	Finland	University of Oulu
Hypsopygia costalis	TLMF Lep 08221	PHLAH402-12	KM572745	BOLD:AAA9616	Austria	inatura, Dornbirn
Hypsopygia costalis	MM06753	LEFID700-10	HM873461	BOLD:AAA9616	Finland	University of Oulu
Hypsopygia glaucinalis	MM01872	LEFIA717-10	HM386861	BOLD:AAC5962	Finland	University of Oulu
Hypsopygia glaucinalis	MM12984	LEFIF768-10	HM875452	BOLD:AAC5962	Finland	University of Oulu
Hypsopygia glaucinalis	MM01873	LEFIA718-10	HM386862	BOLD:AAC5962	Finland	University of Oulu
Hypsopygia glaucinalis	TLMF Lep 08219	PHLAH400-12	KM572194	BOLD:AAC5962	Austria	inatura, Dornbirn
Idaea biselata	MM02918	LEFIB865-10	HM871742	BOLD:AAB2239	Finland	University of Oulu
Idaea biselata	MM12904	LEFIF743-10	HM875427	BOLD:AAB2239	Finland	University of Oulu
Idaea biselata	TLMF Lep 08189	PHLAV370-12	KF808013	BOLD:AAB2239	Austria	inatura, Dornbirn
Idaea biselata	MM01508	LEFIA444-10	HM386785	BOLD:AAB2239	Finland	University of Oulu
Idaea muricata	MM09756	LEFIA879-10	HM387017	BOLD:AAD4604	Finland	University of Oulu
Idaea muricata	MM06826	LEFID761-10	HM873518	BOLD:AAD4604	Finland	University of Oulu
Idaea muricata	TLMF Lep 08538	PHLAH719-12	KF807271	BOLD:AAD4604	Austria	inatura, Dornbirn
Idaea muricata	MM09467	LEFIE573-10	HM874296	BOLD:AAD4604	Finland	University of Oulu
Idaea seriata	TLMF Lep 09896	PHLAW099-13	KM572401	BOLD:AAA9645	Austria	Tiroler Landesmuseum Ferdinandeum
Idaea seriata	MM06892	LEFID806-10	HM873563	BOLD:ACF4900	Finland	University of Oulu
Idaea seriata	MM18414	LEFIK839-10	JN285637	BOLD:ACF4900	Finland	University of Oulu
Idaea seriata	TLMF Lep 08191	PHLAV372-12	KF807175	BOLD:ACF4900	Austria	inatura, Dornbirn
Idaea seriata	MM10367	LEFIE961-10	HM874678	BOLD:ACF4900	Finland	University of Oulu
Idaea serpentata	TLMF Lep 08282	PHLAH463-12	KF807267	BOLD:AAA9647	Austria	inatura, Dornbirn
Idaea serpentata	MM03203	LEFIC003-10	HM871873	BOLD:AAA9647	Finland	University of Oulu
Idaea serpentata	MM06505	LEFID525-10	HM873290	BOLD:AAA9647	Finland	University of Oulu
Idaea serpentata	MM10355	LEFIE955-10	HM874672	BOLD:AAA9647	Finland	University of Oulu
Incurvaria masculella	TLMF Lep 12535	LEATC553-13	KM572100	BOLD:AAD1335	Austria	Research Collection of A. Mayr
Incurvaria masculella	MM18916	LEFIL618-10	KM572751	BOLD:AAD1335	Finland	University of Oulu
Incurvaria masculella	MM18624	LEFIL314-10	KM573248	BOLD:AAD1335	Finland	University of Oulu
Incurvaria masculella	MM05951	LEFIA1188-10	GU828733	BOLD:AAD1335	Finland	University of Oulu
Ipimorpha retusa	MM01702	LEFIA591-10	HM870840	BOLD:AAD8507	Finland	University of Oulu

Ipimorpha retusa	MM04846	LEFIC743-10	HM872564	BOLD:AAD8507	Finland	University of Oulu
Ipimorpha retusa	TLMF Lep 08544	PHLAH725-12	KM572203	BOLD:AAD8507	Austria	inatura, Dornbirn
Ipimorpha retusa	MM01701	LEFIA590-10	HM870839	BOLD:AAD8507	Finland	University of Oulu
Ipimorpha subtusa	TLMF Lep 08543	PHLAH724-12	KM572492	BOLD:AAC2458	Austria	inatura, Dornbirn
Ipimorpha subtusa	MM01698	LEFIA588-10	HM870837	BOLD:AAC2458	Finland	University of Oulu
Ipimorpha subtusa	MM18044	LEFIK469-10	JF854081	BOLD:AAC2458	Finland	University of Oulu
Ipimorpha subtusa	TLMF Lep 00305	PHLAA265-09	HM425802	BOLD:AAC2458	Austria	Tiroler Landesmuseum Ferdinandeum
Ipimorpha subtusa	MM01699	LEFIA589-10	HM870838	BOLD:AAC2458	Finland	University of Oulu
Jodis lactearia	MM14292	LEFIG322-10	HM876001	BOLD:AAD4811	Finland	University of Oulu
Jodis lactearia	MM01504	LEFIA440-10	HM386781	BOLD:AAD4811	Finland	University of Oulu
Jodis lactearia	TLMF Lep 07976	PHLAV157-12	KF807766	BOLD:AAD4811	Austria	inatura, Dornbirn
Jodis lactearia	MM01505	LEFIA441-10	HM386782	BOLD:AAD4811	Finland	University of Oulu
Jodis putata	MM01506	LEFIA442-10	HM386783	BOLD:ABZ4040	Finland	University of Oulu
Jodis putata	MM08170	LEFIE054-10	HQ570357	BOLD:ABZ4040	Finland	University of Oulu
Jodis putata	TLMF Lep 10004	LEATA397-13	KM572040	BOLD:ABZ4040	Austria	inatura, Dornbirn
Jodis putata	MM01507	LEFIA443-10	HM386784	BOLD:ABZ4040	Finland	University of Oulu
Lacanobia contigua	TLMF Lep 08147	PHLAV328-12	KM572589	BOLD:AAD8512	Austria	inatura, Dornbirn
Lacanobia contigua	MM04584	LEFIC639-10	HM872460	BOLD:AAD8512	Finland	University of Oulu
Lacanobia contigua	MM01633	LEFIA546-10	KM572488	BOLD:AAD8512	Finland	University of Oulu
Lacanobia contigua	TLMF Lep 04627	PHLAE312-11	JN272310	BOLD:AAD8512	Austria	Tiroler Landesmuseum Ferdinandeum
Lacanobia contigua	MM01632	LEFIA545-10	KM573637	BOLD:AAD8512	Finland	University of Oulu
Lacanobia contigua	MM18736	LEFIL438-10	KM572763	BOLD:AAD8512	Finland	University of Oulu
Lacanobia oleracea	TLMF Lep 08182	PHLAV363-12	KM572862	BOLD:ABY4614	Austria	inatura, Dornbirn
Lacanobia oleracea	MM01617	LEFIA533-10	KM572889	BOLD:ABY4614	Finland	University of Oulu
Lacanobia oleracea	MM21086	LEFIJ1226-11	KM572069	BOLD:ABY4614	Finland	University of Oulu
Lacanobia oleracea	MM01618	LEFIA534-10	KM573599	BOLD:ABY4614	Finland	University of Oulu
Lacanobia oleracea	MM05111	LEFIC862-10	HM872680	BOLD:ABY4614	Finland	University of Oulu
Lacanobia suasa	TLMF Lep 07880	PHLAV061-12	KM572847	BOLD:ACF5197	Austria	inatura, Dornbirn
Lacanobia suasa	MM17285	LEFIJ660-10	JF853754	BOLD:ACF5197	Finland	University of Oulu
Lacanobia suasa	MM12654	LEFIF674-10	HM875358	BOLD:ACF5197	Finland	University of Oulu
Lacanobia suasa	MM05146	LEFIC873-10	HM872691	BOLD:ACF5197	Finland	University of Oulu

Lacanobia suasa	MM05145	LEFIC872-10	HM872690	BOLD:ACF5197	Finland	University of Oulu
Lacanobia thalassina	MM01589	LEFIA507-10	KM573256	BOLD:ABY4615	Finland	University of Oulu
Lacanobia thalassina	MM01588	LEFIA506-10	KM573683	BOLD:ABY4615	Finland	University of Oulu
Lacanobia thalassina	MM00608	LEFIB183-10	HM871087	BOLD:ABY4615	Finland	University of Oulu
Lacanobia thalassina	TLMF Lep 08144	PHLAV325-12	KM573071	BOLD:ABY4615	Austria	inatura, Dornbirn
Lacanobia w-latinum	MM15953	LEFIJ353-10	KM573034	BOLD:AAC6463	Finland	University of Oulu
Lacanobia w-latinum	TLMF Lep 08107	PHLAV288-12	KM572018	BOLD:AAC6463	Austria	inatura, Dornbirn
Lacanobia w-latinum	TLMF Lep 04609	PHLAE294-11	JN272309	BOLD:AAC6463	Austria	Tiroler Landesmuseum Ferdinandeum
Lampronia corticella	MM15519	LEFIG655-10	HM876316	BOLD:AAD7517	Finland	University of Oulu
Lampronia corticella	MM15520	LEFIG656-10	HM876317	BOLD:AAD7517	Finland	University of Oulu
Lampronia corticella	MM05349	LEFIC963-10	HM872778	BOLD:AAD7517	Finland	University of Oulu
Lampronia corticella	TLMF Lep 07690	PHLAH251-12	KM572130	BOLD:AAD7517	Austria	Tiroler Landesmuseum Ferdinandeum
Lampronia rupella	MM14304	LEFIG330-10	HM876009	BOLD:AAD5208	Finland	University of Oulu
Lampronia rupella	TLMF Lep 04291	PHLAE071-11	JN273729	BOLD:AAD5208	Austria	Tiroler Landesmuseum Ferdinandeum
Lampronia rupella	TLMF Lep 04292	PHLAE072-11	JN273730	BOLD:AAD5208	Austria	Tiroler Landesmuseum Ferdinandeum
Lampronia rupella	MM02270	LEFIB582-10	HM871462	BOLD:AAD5208	Finland	University of Oulu
Lampronia rupella	MM06494	LEFID520-10	HM873285	BOLD:AAD5208	Finland	University of Oulu
Lampropteryx suffumata	TLMF Lep 07891	PHLAV072-12	KM572414	BOLD:AAB2818	Austria	inatura, Dornbirn
Lampropteryx suffumata	MM07800	LEFID950-10	HM873700	BOLD:AAB2818	Finland	University of Oulu
Lampropteryx suffumata	MM13857	LEFIA929-10	HM387063	BOLD:AAB2818	Finland	University of Oulu
Lampropteryx suffumata	MM05419	LEFIC998-10	HM872813	BOLD:AAB2818	Finland	University of Oulu
Lampropteryx suffumata	MM11570	LEFIF366-10	HM875051	BOLD:AAB2818	Finland	University of Oulu
Lampropteryx suffumata	MM01302	LEFIA264-10	HM386608	BOLD:AAB2818	Finland	University of Oulu
Lampropteryx suffumata	MM06420	LEFID478-10	HM873244	BOLD:AAB2818	Finland	University of Oulu
Lampropteryx suffumata	MM01301	LEFIA263-10	HM386607	BOLD:AAB2818	Finland	University of Oulu
Lampropteryx suffumata	MM06419	LEFID477-10	HM873243	BOLD:AAB2818	Finland	University of Oulu
Lamprotes c-aureum	MM00301	LEFIB036-10	HM870945	BOLD:AAE6100	Finland	University of Oulu
Lamprotes c-aureum	MM00019	LEFIB008-10	HM870921	BOLD:AAE6100	Finland	University of Oulu
Lamprotes c-aureum	TLMF Lep 10025	LEATA418-13	KM573701	BOLD:AAE6100	Austria	inatura, Dornbirn
Lamprotes c-aureum	MM11063	LEFIF281-10	HM874974	BOLD:AAE6100	Finland	University of Oulu
Lamprotes c-aureum	MM00018	LEFIB007-10	HM870920	BOLD:AAE6100	Finland	University of Oulu

Lamprotes c-aureum	MM03795	LEFIC305-10	HM872149	BOLD:AAE6100	Finland	University of Oulu
Laothoe populi	MM12527	LEFIF632-10	HM875316	BOLD:AAB3599	Finland	University of Oulu
Laothoe populi	MM02803	LEFIB838-10	HM871715	BOLD:AAB3599	Finland	University of Oulu
Laothoe populi	MM00976	LEFIA1198-10	GU828650	BOLD:AAB3599	Finland	University of Oulu
Laothoe populi	MM00977	LEFIA064-10	HM396411	BOLD:AAB3599	Finland	University of Oulu
Laothoe populi	TLMF Lep 08118	PHLAV299-12	KM573654	BOLD:AAB3599	Austria	inatura, Dornbirn
Lasiocampa quercus	MM11191	LEFIF356-10	HM875041	BOLD:AAC5660	Finland	University of Oulu
Lasiocampa quercus	MM15183	LEFIJ297-10	JF853534	BOLD:AAC5660	Finland	University of Oulu
Lasiocampa quercus	MM12528	LEFIJ196-10	KM573509	BOLD:AAC5660	Finland	University of Oulu
Lasiocampa quercus	MM10716	LEFIJ162-10	KM573652	BOLD:AAC5660	Finland	University of Oulu
Lasiocampa quercus	MM17998	LEFIK423-10	JF854052	BOLD:AAC5660	Finland	University of Oulu
Lasiocampa quercus	MM03714	LEFIA1200-10	GU828694	BOLD:AAC5660	Finland	University of Oulu
Lasiocampa quercus	MM00380	LEFIB078-10	HM870987	BOLD:AAC5660	Finland	University of Oulu
Lasiocampa quercus	MM06739	LEFID691-10	HM873452	BOLD:AAC5660	Finland	University of Oulu
Lasiocampa quercus	MM14213	LEFIG273-10	HM875952	BOLD:AAC5660	Finland	University of Oulu
Lasiocampa quercus	MM06634	LEFID618-10	HM873383	BOLD:AAC5660	Finland	University of Oulu
Lasiocampa quercus	MM10251	LEFIE907-10	HM874624	BOLD:AAC5660	Finland	University of Oulu
Lasiocampa quercus	TLMF Lep 08503	PHLAH684-12	KM573393	BOLD:AAC5660	Austria	inatura, Dornbirn
Lasiommata maera	MM06603	LEFID593-10	HM873358	BOLD:AAA8853	Finland	University of Oulu
Lasiommata maera	MM17188	LEFIJ563-10	JF853678	BOLD:AAA8853	Finland	University of Oulu
Lasiommata maera	MM17187	LEFIJ562-10	JF853677	BOLD:AAA8853	Finland	University of Oulu
Lasiommata maera	TLMF Lep 10017	LEATA410-13	KM572133	BOLD:ACN7901	Austria	inatura, Dornbirn
Lasiommata petropolitana	MM17189	LEFIJ564-10	JF853679	BOLD:AAC3112	Finland	University of Oulu
Lasiommata petropolitana	MM00591	LEFIB177-10	HM871081	BOLD:AAC3112	Finland	University of Oulu
Lasiommata petropolitana	TLMF Lep 08277	PHLAH458-12	KM572504	BOLD:AAC3112	Austria	inatura, Dornbirn
Lasiommata petropolitana	MM06429	LEFID485-10	HM873251	BOLD:AAC3112	Finland	University of Oulu
Lasionycta imbecilla	TLMF Lep 06108	PHLSA653-11	KM572959	BOLD:AAE1902	Austria	Tiroler Landesmuseum Ferdinandeum
Lasionycta imbecilla	TLMF Lep 08547	PHLAH728-12	KM572748	BOLD:AAE1902	Austria	inatura, Dornbirn
Lasionycta imbecilla	MM01610	LEFIA527-10	KM571992	BOLD:AAE1902	Finland	University of Oulu
Lasionycta imbecilla	MM06604	LEFID594-10	HM873359	BOLD:AAE1902	Finland	University of Oulu
Lasionycta imbecilla	MM01609	LEFIA526-10	KM573003	BOLD:AAE1902	Finland	University of Oulu

Lasionycta imbecilla	TLMF Lep 04675	PHLAE360-11	JN272314	BOLD:AAE1902	Austria	Tiroler Landesmuseum Ferdinandeum
Lasionycta proxima	TLMF Lep 08777	PHLAI282-13	KM573385	BOLD:AAD3486	Austria	Tiroler Landesmuseum Ferdinandeum
Lasionycta proxima	MM15891	LEFIH027-10	HM876663	BOLD:AAD3486	Finland	University of Oulu
Lasionycta proxima	MM09917	LEFIE768-10	HM874487	BOLD:AAD3486	Finland	University of Oulu
Lasionycta proxima	MM15890	LEFIH026-10	HM876662	BOLD:AAD3486	Finland	University of Oulu
Laspeyria flexula	TLMF Lep 07888	PHLAV069-12	KM572119	BOLD:AAC1014	Austria	inatura, Dornbirn
Laspeyria flexula	MM05088	LEFIC854-10	HM872672	BOLD:AAC1014	Finland	University of Oulu
Laspeyria flexula	MM01415	LEFIA359-10	HM386702	BOLD:AAC1014	Finland	University of Oulu
Laspeyria flexula	MM01416	LEFIA360-10	HM386703	BOLD:AAC1014	Finland	University of Oulu
Lateroligia ophiogramma	TLMF Lep 08194	PHLAV375-12	KM573657	BOLD:AAB0872	Austria	inatura, Dornbirn
Lateroligia ophiogramma	MM12687	LEFIF682-10	HM875366	BOLD:AAB0872	Finland	University of Oulu
Lateroligia ophiogramma	MM10788	LEFIF192-10	HM874886	BOLD:AAB0872	Finland	University of Oulu
Lateroligia ophiogramma	MM04871	LEFIC756-10	HM872575	BOLD:AAB0872	Finland	University of Oulu
Lathronympha strigana	MM14471	LEFIG435-10	HM876111	BOLD:AAC1866	Finland	University of Oulu
Lathronympha strigana	MM02083	LEFIB499-10	HM871394	BOLD:AAC1866	Finland	University of Oulu
Lathronympha strigana	TLMF Lep 12537	LEATC555-13	KM572836	BOLD:AAC1866	Austria	Tiroler Landesmuseum Ferdinandeum
Lathronympha strigana	MM08966	LEFIE403-10	HM874127	BOLD:AAC1866	Finland	University of Oulu
Lathronympha strigana	MM05266	LEFIC922-10	HM872739	BOLD:AAC1866	Finland	University of Oulu
Lathronympha strigana	MM09440	LEFIE555-10	HM874278	BOLD:AAC1866	Finland	University of Oulu
Leptidea juvernica	TLMF Lep 09838	PHLAW041-13	KM572629	BOLD:ABY8343	Austria	Tiroler Landesmuseum Ferdinandeum
Leptidea juvernica	TLMF Lep 09122	PHLAI560-13	KM573150	BOLD:ABY8343	Austria	Tiroler Landesmuseum Ferdinandeum
Leptidea juvernica	MM22892	LEFIJ2040-13	KM572124	BOLD:ABY8343	Finland	University of Oulu
Leptidea juvernica	TLMF Lep 08294	PHLAH475-12	KM572994	BOLD:ABY8343	Austria	inatura, Dornbirn
Leptidea sinapis	MM18773	LEFIL475-10	KM572515	BOLD:AAA6298	Finland	University of Oulu
Leptidea sinapis	MM05733	LEFID110-10	HM872921	BOLD:AAA6298	Finland	University of Oulu
Leptidea sinapis	MM04238	LEFIC521-10	HM872344	BOLD:AAA6298	Finland	University of Oulu
Leptidea sinapis	MM00498	LEFIB130-10	HM871036	BOLD:AAA6298	Finland	University of Oulu
Leptidea sinapis	TLMF Lep 06138	PHLSA683-11	KM573679	BOLD:AAA6298	Austria	Tiroler Landesmuseum Ferdinandeum
Leptidea sinapis	MM13893	LEFIG106-10	HM875786	BOLD:AAA6298	Finland	University of Oulu
Leptidea sinapis	MM06100	LEFID238-10	HM873038	BOLD:AAA6298	Finland	University of Oulu
Leucania comma	MM09493	LEFIE586-10	HM874309	BOLD:AAA3150	Finland	University of Oulu

Leucania comma	TLMF Lep 09226	PHLAI663-13	KM573031	BOLD:AAA3150	Austria	Tiroler Landesmuseum Ferdinandeum
Leucania comma	MM01602	LEFIA519-10	KM572973	BOLD:AAA3150	Finland	University of Oulu
Leucania comma	MM01601	LEFIA518-10	KM572004	BOLD:AAA3150	Finland	University of Oulu
Leucania obsoleta	MM00662	LEFIB210-10	HM871114	BOLD:AAD5689	Finland	University of Oulu
Leucania obsoleta	TLMF Lep 04452	PHLAE232-11	JN272306	BOLD:AAD5689	Austria	Tiroler Landesmuseum Ferdinandeum
Leucania obsoleta	MM04572	LEFIC630-10	HM872451	BOLD:AAD5689	Finland	University of Oulu
Leucania obsoleta	MM11514	LEFIF362-10	HM875047	BOLD:AAD5689	Finland	University of Oulu
Limnaecia phragmitella	MM00333	LEFIA1206-10	JF818765	BOLD:AAA7368	Finland	University of Oulu
Limnaecia phragmitella	TLMF Lep 12467	LEATC485-13	KM573409	BOLD:AAA7368	Austria	Tiroler Landesmuseum Ferdinandeum
Limnaecia phragmitella	MM08567	LEFIE244-10	HM873978	BOLD:AAA7368	Finland	University of Oulu
Limnaecia phragmitella	MM13549	LEFIF967-10	HM875648	BOLD:AAA7368	Finland	University of Oulu
Limnaecia phragmitella	MM06831	LEFID765-10	HM873522	BOLD:AAA7368	Finland	University of Oulu
Lithophane consocia	MM02763	LEFIB823-10	HM871700	BOLD:AAD9908	Finland	University of Oulu
Lithophane consocia	MM01556	LEFIA479-10	KM573693	BOLD:AAD9908	Finland	University of Oulu
Lithophane consocia	MM01555	LEFIA478-10	KM573328	BOLD:AAD9908	Finland	University of Oulu
Lithophane consocia	TLMF Lep 08757	PHLAI262-13	KM573083	BOLD:AAD9908	Austria	Tiroler Landesmuseum Ferdinandeum
Lithophane furcifera	MM18038	LEFIK463-10	KM572564	BOLD:AAJ2397	Finland	University of Oulu
Lithophane furcifera	MM10812	LEFIF198-10	HM874892	BOLD:AAJ2397	Finland	University of Oulu
Lithophane furcifera	TLMF Lep 09515	LEATA098-13	KM573357	BOLD:AAJ2397	Austria	Tiroler Landesmuseum Ferdinandeum
Lithophane furcifera	MM12670	LEFIF679-10	HM875363	BOLD:AAJ2397	Finland	University of Oulu
Lithosia quadra	MM14423	LEFIG400-10	HM876077	BOLD:AAB8723	Finland	University of Oulu
Lithosia quadra	MM17198	LEFIJ573-10	JF853686	BOLD:AAB8723	Finland	University of Oulu
Lithosia quadra	MM14422	LEFIG399-10	HM876076	BOLD:AAB8723	Finland	University of Oulu
Lithosia quadra	TLMF Lep 08513	PHLAH694-12	KM572652	BOLD:AAB8723	Austria	inatura, Dornbirn
Lobesia reliquana	TLMF Lep 12503	LEATC521-13	KM573424	BOLD:AAC9385	Austria	Tiroler Landesmuseum Ferdinandeum
Lobesia reliquana	MM11878	LEFIF434-10	HM875119	BOLD:AAC9385	Finland	University of Oulu
Lobesia reliquana	MM10029	LEFIE822-10	HM874541	BOLD:AAC9385	Finland	University of Oulu
Lobesia reliquana	MM15710	LEFIG846-10	HM876494	BOLD:AAC9385	Finland	University of Oulu
Lobesia reliquana	MM03817	LEFIC315-10	HM872159	BOLD:AAC9385	Finland	University of Oulu
Lobesia reliquana	MM14040	LEFIG174-10	HM875854	BOLD:AAC9385	Finland	University of Oulu
Lobesia reliquana	MM15712	LEFIG848-10	HM876496	BOLD:AAC9385	Finland	University of Oulu

Lobesia reliquana	MM06146	LEFID273-10	HM873071	BOLD:AAC9385	Finland	University of Oulu
Lobesia reliquana	MM15711	LEFIG847-10	HM876495	BOLD:AAC9385	Finland	University of Oulu
Lobophora halterata	TLMF Lep 08812	PHLAI317-13	KM573237	BOLD:AAB7562	Austria	Tiroler Landesmuseum Ferdinandeum
Lobophora halterata	MM10101	LEFIE859-10	HM874577	BOLD:AAB7562	Finland	University of Oulu
Lobophora halterata	MM01274	LEFIA241-10	HM386585	BOLD:AAB7562	Finland	University of Oulu
Lobophora halterata	TLMF Lep 04669	PHLAE354-11	JN279573	BOLD:AAB7562	Austria	Tiroler Landesmuseum Ferdinandeum
Lobophora halterata	MM01275	LEFIA242-10	HM386586	BOLD:AAB7562	Finland	University of Oulu
Lomaspilis marginata	MM01248	LEFIA217-10	HM396560	BOLD:AAB5300	Finland	University of Oulu
Lomaspilis marginata	MM01249	LEFIA218-10	HM396561	BOLD:AAB5300	Finland	University of Oulu
Lomaspilis marginata	MM01247	LEFIA1209-10	KM573010	BOLD:AAB5300	Finland	University of Oulu
Lomaspilis marginata	TLMF Lep 04618	PHLAE303-11	JN269335	BOLD:AAB5300	Austria	Tiroler Landesmuseum Ferdinandeum
Lomaspilis marginata	MM00572	LEFIB169-10	HM871073	BOLD:AAB5300	Finland	University of Oulu
Lomographa bimaculata	TLMF Lep 07864	PHLAV045-12	KM572093	BOLD:AAB9407	Austria	inatura, Dornbirn
Lomographa bimaculata	MM01311	LEFIA271-10	HM386615	BOLD:AAB9407	Finland	University of Oulu
Lomographa bimaculata	MM01310	LEFIA270-10	HM386614	BOLD:AAB9407	Finland	University of Oulu
Lomographa bimaculata	MM03704	LEFIC272-10	HM872116	BOLD:AAB9407	Finland	University of Oulu
Lomographa temerata	MM01309	LEFIA269-10	HM386613	BOLD:AAB5203	Finland	University of Oulu
Lomographa temerata	MM03703	LEFIC271-10	HM872115	BOLD:AAB5203	Finland	University of Oulu
Lomographa temerata	TLMF Lep 07865	PHLAV046-12	KM572019	BOLD:AAB5203	Austria	inatura, Dornbirn
Lomographa temerata	MM01308	LEFIA268-10	HM386612	BOLD:AAB5203	Finland	University of Oulu
Loxostege sticticalis	MM10564	LEFIF111-10	HM874815	BOLD:AAB4167	Finland	University of Oulu
Loxostege sticticalis	TLMF Lep 07998	PHLAV179-12	KM572887	BOLD:AAB4167	Austria	inatura, Dornbirn
Loxostege sticticalis	MM10563	LEFIF110-10	HM874814	BOLD:AAB4167	Finland	University of Oulu
Loxostege sticticalis	MM10562	LEFIF109-10	HM874813	BOLD:AAB4167	Finland	University of Oulu
Lycaena hippothoe	MM17123	LEFIJ498-10	JF853622	BOLD:AAB8947	Finland	University of Oulu
Lycaena hippothoe	TLMF Lep 09829	PHLAW032-13	KM573077	BOLD:AAB8947	Austria	Tiroler Landesmuseum Ferdinandeum
Lycaena hippothoe	TLMF Lep 09828	PHLAW031-13	KM572581	BOLD:AAB8947	Austria	Tiroler Landesmuseum Ferdinandeum
Lycaena hippothoe	MM17122	LEFIJ497-10	JF853621	BOLD:AAB8947	Finland	University of Oulu
Lycaena hippothoe	MM06257	LEFID354-10	HM873151	BOLD:AAB8947	Finland	University of Oulu
Lycaena phlaeas	TLMF Lep 09836	PHLAW039-13	KM572099	BOLD:AAA5867	Austria	Tiroler Landesmuseum Ferdinandeum
Lycaena phlaeas	MM17119	LEFIJ494-10	JF853619	BOLD:AAA5867	Finland	University of Oulu

Lycaena phlaeas	MM03698	LEFIC267-10	HM872111	BOLD:AAA5867	Finland	University of Oulu
Lycaena phlaeas	MM10279	LEFIE915-10	HM874632	BOLD:AAA5867	Finland	University of Oulu
Lycaena virgaureae	MM14659	LEFIG533-10	HM876206	BOLD:AAB4984	Finland	University of Oulu
Lycaena virgaureae	TLMF Lep 12603	LEATC621-13	KM573555	BOLD:AAB4984	Austria	Tiroler Landesmuseum Ferdinandeum
Lycaena virgaureae	MM00305	LEFIB038-10	HM870947	BOLD:AAB4984	Finland	University of Oulu
Lycaena virgaureae	MM17121	LEFIJ496-10	JF853620	BOLD:AAB4984	Finland	University of Oulu
Lycia hirtaria	MM02807	LEFIB840-10	HM871717	BOLD:ACF3346	Finland	University of Oulu
Lycia hirtaria	MM01484	LEFIA422-10	HM386764	BOLD:ACF3346	Finland	University of Oulu
Lycia hirtaria	MM01483	LEFIA421-10	HM386763	BOLD:ACF3346	Finland	University of Oulu
Lycia hirtaria	TLMF Lep 09777	LEATA360-13	KM573412	BOLD:ACF3346	Austria	Tiroler Landesmuseum Ferdinandeum
Lycophotia porphyrea	MM10908	LEFIF213-10	HM874907	BOLD:AAC8982	Finland	University of Oulu
Lycophotia porphyrea	MM05151	LEFIC877-10	HM872695	BOLD:AAC8982	Finland	University of Oulu
Lycophotia porphyrea	TLMF Lep 10023	LEATA416-13	KM573088	BOLD:AAC8982	Austria	inatura, Dornbirn
Lycophotia porphyrea	MM02760	LEFIB822-10	HM871699	BOLD:AAC8982	Finland	University of Oulu
Lygephila craccae	MM18522	LEFIK947-10	JF854385	BOLD:AAD9537	Finland	University of Oulu
Lygephila craccae	MM12512	LEFIF623-10	HM875307	BOLD:AAD9537	Finland	University of Oulu
Lygephila craccae	MM12561	LEFIF648-10	HM875332	BOLD:AAD9537	Finland	University of Oulu
Lygephila craccae	TLMF Lep 08512	PHLAH693-12	KM573145	BOLD:AAD9538	Austria	inatura, Dornbirn
Lygephila viciae	MM17460	LEFIJ835-10	JF853853	BOLD:AAK6149	Finland	University of Oulu
Lygephila viciae	MM18665	LEFIL355-10	JN263678	BOLD:AAK6149	Finland	University of Oulu
Lygephila viciae	TLMF Lep 08113	PHLAV294-12	KM572094	BOLD:AAK6149	Austria	inatura, Dornbirn
Lygephila viciae	MM15931	LEFIJ331-10	KM573214	BOLD:AAK6149	Finland	University of Oulu
Lygephila viciae	MM17459	LEFIJ834-10	JF853852	BOLD:AAK6149	Finland	University of Oulu
Lygephila viciae	TLMF Lep 04650	PHLAE335-11	JN264260	BOLD:AAK6149	Austria	Tiroler Landesmuseum Ferdinandeum
Lygephila viciae	TLMF Lep 06189	PHLSA734-11	KM573268	BOLD:AAK6149	Austria	Tiroler Landesmuseum Ferdinandeum
Lymantria monacha	MM00138	LEFIA1214-10	KM572571	BOLD:AAA5537	Finland	University of Oulu
Lymantria monacha	MM01050	LEFIA122-10	HM396469	BOLD:AAA5537	Finland	University of Oulu
Lymantria monacha	MM01048	LEFIA1213-10	GU828655	BOLD:AAA5537	Finland	University of Oulu
Lymantria monacha	MM12596	LEFIF659-10	HM875343	BOLD:AAA5537	Finland	University of Oulu
Lymantria monacha	TLMF Lep 08507	PHLAH688-12	KM572468	BOLD:AAA5537	Austria	inatura, Dornbirn
Lymantria monacha	MM03651	LEFIC242-10	HM872086	BOLD:AAA5537	Finland	University of Oulu

Lyonetia clerkella	MM13739	LEFIG084-10	HM875763	BOLD:AAD5255	Finland	University of Oulu
Lyonetia clerkella	TLMF Lep 12612	LEATC630-13	KM572779	BOLD:AAD5255	Austria	Tiroler Landesmuseum Ferdinandeum
Lyonetia clerkella	MM00946	LEFIB374-10	HM871273	BOLD:AAD5255	Finland	University of Oulu
Lyonetia clerkella	MM02621	LEFIB761-10	HM871638	BOLD:AAD5256	Finland	University of Oulu
Lyonetia clerkella	MM22063	LEFIJ1392-12	KM573505	BOLD:AAD5256	Finland	University of Oulu
Macaria alternata	TLMF Lep 08540	PHLAH721-12	KM573460	BOLD:AAA1496	Austria	inatura, Dornbirn
Macaria alternata	MM11578	LEFIF369-10	HM875054	BOLD:AAA1496	Finland	University of Oulu
Macaria alternata	MM01334	LEFIA290-10	HM386633	BOLD:AAA1496	Finland	University of Oulu
Macaria alternata	MM01333	LEFIA289-10	HM386632	BOLD:AAA1496	Finland	University of Oulu
Macaria brunneata	MM08082	LEFIE023-10	HM873772	BOLD:ABY9522	Finland	University of Oulu
Macaria brunneata	TLMF Lep 06170	PHLSA715-11	KM572273	BOLD:ABY9522	Austria	Tiroler Landesmuseum Ferdinandeum
Macaria brunneata	MM01455	LEFIA396-10	HM386738	BOLD:ABY9522	Finland	University of Oulu
Macaria brunneata	MM01456	LEFIA397-10	HM386739	BOLD:ABY9522	Finland	University of Oulu
Macaria fusca	TLMF Lep 12551	LEATC569-13	KM573532	BOLD:AAC7749	Austria	Tiroler Landesmuseum Ferdinandeum
Macaria fusca	TLMF Lep 09503	LEATA086-13	KM572622	BOLD:AAC7749	Austria	Tiroler Landesmuseum Ferdinandeum
Macaria fusca	MM03420	LEFIC104-10	HQ570295	BOLD:AAC7749	Finland	University of Oulu
Macaria fusca	MM14587	LEFIG503-10	HM876177	BOLD:AAC7749	Finland	University of Oulu
Macaria fusca	MM18508	LEFIK933-10	JF854375	BOLD:AAC7749	Finland	University of Oulu
Macaria liturata	MM04865	LEFIC751-10	HM872570	BOLD:ABX6221	Finland	University of Oulu
Macaria liturata	TLMF Lep 07869	PHLAV050-12	KM572417	BOLD:ABX6221	Austria	inatura, Dornbirn
Macaria liturata	MM01481	LEFIA419-10	HM386761	BOLD:ACF0383	Finland	University of Oulu
Macaria liturata	MM01480	LEFIA418-10	HM386760	BOLD:ACF4721	Finland	University of Oulu
Macaria signaria	TLMF Lep 04657	PHLAE342-11	JN269339	BOLD:AAA1255	Austria	Tiroler Landesmuseum Ferdinandeum
Macaria signaria	MM06029	LEFID202-10	HM873005	BOLD:AAA1255	Finland	University of Oulu
Macaria signaria	MM14253	LEFIG296-10	HM875975	BOLD:AAA1255	Finland	University of Oulu
Macaria signaria	MM14051	LEFIG183-10	HM875863	BOLD:AAA1255	Finland	University of Oulu
Macdunnoughia confusa	MM12215	LEFIF537-10	HM875222	BOLD:AAD5865	Finland	University of Oulu
Macdunnoughia confusa	TLMF Lep 08195	PHLAV376-12	KM572869	BOLD:AAD5865	Austria	inatura, Dornbirn
Macdunnoughia confusa	MM18525	LEFIK950-10	JF854388	BOLD:AAD5865	Finland	University of Oulu
Macdunnoughia confusa	MM12657	LEFIF675-10	HM875359	BOLD:AAD5865	Finland	University of Oulu

Macrothylacia rubi	MM01059	LEFIA128-10	HM396475	BOLD:AAD0839	Finland	University of Oulu
Macrothylacia rubi	MM01060	LEFIA129-10	HM396476	BOLD:AAD0839	Finland	University of Oulu
Macrothylacia rubi	TLMF Lep 07824	PHLAV005-12	KM572479	BOLD:AAD0839	Austria	inatura, Dornbirn
Macrothylacia rubi	MM10712	LEFIF166-10	HM874860	BOLD:AAD0839	Finland	University of Oulu
Malacosoma neustria	MM17218	LEFIJ593-10	JF853693	BOLD:AAD1119	Finland	University of Oulu
Malacosoma neustria	TLMF Lep 09500	LEATA083-13	KM572291	BOLD:AAD1119	Austria	Tiroler Landesmuseum Ferdinandeum
Malacosoma neustria	MM17217	LEFIJ592-10	JF853692	BOLD:AAD1119	Finland	University of Oulu
Mamestra brassicae	MM04910	LEFIC778-10	HM872597	BOLD:AAB7858	Finland	University of Oulu
Mamestra brassicae	TLMF Lep 07904	PHLAV085-12	KM571997	BOLD:AAB7858	Austria	inatura, Dornbirn
Mamestra brassicae	TLMF Lep 08778	PHLAI283-13	KM573288	BOLD:AAB7858	Austria	Tiroler Landesmuseum Ferdinandeum
Mamestra brassicae	MM04911	LEFIC779-10	HM872598	BOLD:AAB7858	Finland	University of Oulu
Mamestra brassicae	MM12599	LEFIF660-10	HM875344	BOLD:AAB7858	Finland	University of Oulu
Maniola jurtina	MM06902	LEFID815-10	HM873572	BOLD:AAA7785	Finland	University of Oulu
Maniola jurtina	MM06901	LEFID814-10	HM873571	BOLD:AAA7785	Finland	University of Oulu
Maniola jurtina	TLMF Lep 08291	PHLAH472-12	KM573026	BOLD:AAA7785	Austria	inatura, Dornbirn
Maniola jurtina	MM06856	LEFID787-10	HM873544	BOLD:AAA7786	Finland	University of Oulu
Marasmarcha lunaedactyla	MM11051	LEFIF269-10	HM874962	BOLD:AAL6978	Finland	University of Oulu
Marasmarcha lunaedactyla	MM11052	LEFIF270-10	HM874963	BOLD:AAL6978	Finland	University of Oulu
Marasmarcha lunaedactyla	MM18376	LEFIK801-10	KM572405	BOLD:AAL6978	Finland	University of Oulu
Marasmarcha lunaedactyla	TLMF Lep 08008	PHLAV189-12	KM573050	BOLD:ABX6234	Austria	inatura, Dornbirn
Martania taeniata	TLMF Lep 09416	PHLAI854-13	KM572619	BOLD:AAE9770	Austria	Tiroler Landesmuseum Ferdinandeum
Martania taeniata	TLMF Lep 09993	LEATA386-13	KM572829	BOLD:AAE9770	Austria	inatura, Dornbirn
Martania taeniata	MM01764	LEFIA647-10	HM870896	BOLD:AAE9770	Finland	University of Oulu
Martania taeniata	MM01448	LEFIA391-10	HM386733	BOLD:AAE9770	Finland	University of Oulu
Martania taeniata	MM08468	LEFIE180-10	HM873926	BOLD:AAE9770	Finland	University of Oulu
Matilella fusca	MM09468	LEFIE574-10	HM874297	BOLD:AAA4759	Finland	University of Oulu
Matilella fusca	MM09959	LEFIE782-10	HM874501	BOLD:AAA4759	Finland	University of Oulu
Matilella fusca	TLMF Lep 09923	PHLAW126-13	KM573585	BOLD:AAA4759	Austria	inatura, Dornbirn

Matilella fusca	MM06652	LEFID631-10	HM873394	BOLD:AAA4759	Finland	University of Oulu
Meganola strigula	MM04673	LEFIC692-10	HM872513	BOLD:AAD8314	Finland	University of Oulu
Meganola strigula	MM06723	LEFID680-10	HM873441	BOLD:AAD8314	Finland	University of Oulu
Meganola strigula	TLMF Lep 08558	PHLAH739-12	KM572007	BOLD:AAD8314	Austria	inatura, Dornbirn
Meganola strigula	MM09551	LEFIE622-10	HM874345	BOLD:AAD8314	Finland	University of Oulu
Melanchra persicariae	MM01597	LEFIA515-10	KM573690	BOLD:AAC7429	Finland	University of Oulu
Melanchra persicariae	TLMF Lep 08129	PHLAV310-12	KM572238	BOLD:AAC7429	Austria	inatura, Dornbirn
Melanchra persicariae	MM04556	LEFIC622-10	HM872443	BOLD:AAC7429	Finland	University of Oulu
Melanchra persicariae	MM01596	LEFIA514-10	KM572312	BOLD:AAC7429	Finland	University of Oulu
Melitaea athalia	MM17174	LEFIJ549-10	JF853664	BOLD:AAA3081	Finland	University of Oulu
Melitaea athalia	MM10073	LEFIE848-10	HM874567	BOLD:AAA3081	Finland	University of Oulu
Melitaea athalia	MM06109	LEFID245-10	HM873044	BOLD:AAA3081	Finland	University of Oulu
Melitaea athalia	TLMF Lep 06112	PHLSA657-11	KM572443	BOLD:AAA7584	Austria	Tiroler Landesmuseum Ferdinandeum
Melitaea athalia	TLMF Lep 01126	PHLAB326-10	HQ968488	BOLD:AAA7584	Austria	Tiroler Landesmuseum Ferdinandeum
Melitaea diamina	MM18689	LEFIL379-10	JN274637	BOLD:AAA3082	Finland	University of Oulu
Melitaea diamina	TLMF Lep 06111	PHLSA656-11	KM572712	BOLD:AAA3082	Austria	Tiroler Landesmuseum Ferdinandeum
Melitaea diamina	TLMF Lep 00723	PHLAA683-09	HM426083	BOLD:AAA3082	Austria	Tiroler Landesmuseum Ferdinandeum
Melitaea diamina	MM18690	LEFIL380-10	JN274638	BOLD:AAA3082	Finland	University of Oulu
Merrifieldia leucodactyla	MM10051	LEFIE835-10	HM874554	BOLD:AAE1533	Finland	University of Oulu
Merrifieldia leucodactyla	MM06254	LEFID353-10	HM873150	BOLD:AAE1533	Finland	University of Oulu
Merrifieldia leucodactyla	TLMF Lep 00910	PHLAB110-10	HM381482	BOLD:AAE1533	Austria	Tiroler Landesmuseum Ferdinandeum
Merrifieldia leucodactyla	MM17337	LEFIJ712-10	JF853787	BOLD:AAE1533	Finland	University of Oulu
Mesapamea secalella	MM07077	LEFII069-10	KM572280	BOLD:AAB2749	Finland	University of Oulu
Mesapamea secalella	MM07075	LEFII067-10	KM573061	BOLD:AAB2749	Finland	University of Oulu
Mesapamea secalella	MM07074	LEFII066-10	KM572486	BOLD:AAB2749	Finland	University of Oulu
Mesapamea secalella	MM07073	LEFII065-10	KM573142	BOLD:AAB2749	Finland	University of Oulu
Mesapamea secalella	MM07085	LEFII071-10	KM572966	BOLD:AAB2749	Finland	University of Oulu
Mesapamea secalella	TLMF Lep 09225	PHLAI662-13	KM573535	BOLD:AAB2749	Austria	Tiroler Landesmuseum Ferdinandeum
Mesapamea secalella	MM07076	LEFII068-10	KM572517	BOLD:AAB2749	Finland	University of Oulu
Mesapamea secalella	MM07078	LEFII070-10	KM573091	BOLD:AAB2749	Finland	University of Oulu
Mesogona oxalina	MM18726	LEFIL428-10	KM572154	BOLD:AAF2131	Finland	University of Oulu

Mesogona oxalina	TLMF Lep 12466	LEATC484-13	KM572150	BOLD:AAF2131	Austria	Tiroler Landesmuseum Ferdinandeum
Mesogona oxalina	MM14776	LEFIG587-10	HM876258	BOLD:AAF2131	Finland	University of Oulu
Mesogona oxalina	MM18913	LEFIL615-10	KM572484	BOLD:AAF2131	Finland	University of Oulu
Mesoleuca albicillata	MM08673	LEFIJ125-10	JF853448	BOLD:AAC8744	Finland	University of Oulu
Mesoleuca albicillata	MM06322	LEFID403-10	HM873200	BOLD:AAC8744	Finland	University of Oulu
Mesoleuca albicillata	TLMF Lep 04636	PHLAE321-11	JN279568	BOLD:AAC8744	Austria	Tiroler Landesmuseum Ferdinandeum
Mesoleuca albicillata	MM14254	LEFIG297-10	HM875976	BOLD:AAC8744	Finland	University of Oulu
Mesoleuca albicillata	MM01377	LEFIA330-10	HM386673	BOLD:AAC8745	Finland	University of Oulu
Mesoligia furuncula	MM01726	LEFIA612-10	HM870861	BOLD:AAC2820	Finland	University of Oulu
Mesoligia furuncula	MM04869	LEFIC754-10	HM872573	BOLD:AAC2820	Finland	University of Oulu
Mesoligia furuncula	TLMF Lep 08559	PHLAH740-12	KM572448	BOLD:AAC2820	Austria	inatura, Dornbirn
Mesoligia furuncula	MM01727	LEFIA613-10	HM870862	BOLD:AAC2820	Finland	University of Oulu
Mesotype didymata	TLMF Lep 08861	PHLAI366-13	KM572062	BOLD:AAE3950	Austria	Tiroler Landesmuseum Ferdinandeum
Mesotype didymata	MM01355	LEFIA309-10	HM386652	BOLD:AAE3950	Finland	University of Oulu
Mesotype didymata	MM00783	LEFIB289-10	HM871190	BOLD:AAE3950	Finland	University of Oulu
Mesotype didymata	MM01354	LEFIA308-10	HM386651	BOLD:AAE3950	Finland	University of Oulu
Metaxmeste schrankiana	MM13925	LEFIG119-10	HM875799	BOLD:AAE7792	Finland	University of Oulu
Metaxmeste schrankiana	MM18800	LEFIL502-10	JF854629	BOLD:AAE7792	Finland	University of Oulu
Metaxmeste schrankiana	MM10553	LEFIF101-10	HM874805	BOLD:AAE7792	Finland	University of Oulu
Metaxmeste schrankiana	MM10552	LEFIF100-10	HM874804	BOLD:AAE7792	Finland	University of Oulu
Metaxmeste schrankiana	TLMF Lep 09977	PHLAW180-13	KM573530	BOLD:AAE7792	Austria	inatura, Dornbirn
Metendothenia atropunctana	MM06353	LEFID424-10	HM873220	BOLD:ABZ7645	Finland	University of Oulu
Metendothenia atropunctana	MM01960	LEFIB444-10	HM871343	BOLD:ABZ7645	Finland	University of Oulu
Metendothenia atropunctana	MM13213	LEFIF854-10	HM875537	BOLD:ABZ7645	Finland	University of Oulu
Metendothenia atropunctana	TLMF Lep 09202	PHLAI640-13	KM573705	BOLD:ABZ7645	Austria	Tiroler Landesmuseum Ferdinandeum
Micropterix	BIOUG04118-C09	GMFIM247-13	KM572573	BOLD:AAE7973	Finland	Biodiversity Institute of Ontario
Micropterix	BIOUG04118-A02	GMFID722-12	KM573413	BOLD:AAE7973	Finland	Biodiversity Institute of Ontario

Micropterix aruncella	MM14328	LEFIJ243-10	JF853486	BOLD:AAD9347	Finland	University of Oulu
Micropterix aruncella	MM14326	LEFIG341-10	HM876018	BOLD:AAD9347	Finland	University of Oulu
Micropterix aruncella	MM14327	LEFIJ242-10	JF853485	BOLD:AAD9347	Finland	University of Oulu
Micropterix aruncella	TLMF Lep 10050	LEATA443-13	KM572792	BOLD:AAD9347	Austria	inatura, Dornbirn
Micropterix aureatella	MM18052	LEFIK477-10	JF854087	BOLD:AAE7973	Finland	University of Oulu
Micropterix aureatella	TLMF Lep 04308	PHLAE088-11	JN307393	BOLD:AAE7973	Austria	Tiroler Landesmuseum Ferdinandeum
Micropterix aureatella	MM18051	LEFIK476-10	JF854086	BOLD:AAE7973	Finland	University of Oulu
Micropterix aureatella	TLMF Lep 07485	PHLAG806-12	KM572051	BOLD:AAE7973	Austria	inatura, Dornbirn
Micropterix aureatella	MM14889	LEFIJ273-10	JF853512	BOLD:AAE7973	Finland	University of Oulu
Micropterix aureatella	MM14876	LEFIJ262-10	JF853503	BOLD:AAE7973	Finland	University of Oulu
Miltochrista miniata	MM11636	LEFIF382-10	HM875067	BOLD:AAC4781	Finland	University of Oulu
Miltochrista miniata	MM01027	LEFIA104-10	HM396451	BOLD:AAC4781	Finland	University of Oulu
Miltochrista miniata	TLMF Lep 08112	PHLAV293-12	KM572432	BOLD:AAC4781	Austria	inatura, Dornbirn
Miltochrista miniata	MM01028	LEFIA105-10	HM396452	BOLD:AAC4781	Finland	University of Oulu
Mimas tiliae	TLMF Lep 07822	PHLAV003-12	KM573168	BOLD:AAB6049	Austria	inatura, Dornbirn
Mimas tiliae	MM00969	LEFIA057-10	HM396405	BOLD:AAB6050	Finland	University of Oulu
Mimas tiliae	MM04540	LEFIC611-10	HM872432	BOLD:AAB6050	Finland	University of Oulu
Mimas tiliae	MM12516	LEFIF626-10	HM875310	BOLD:AAB6050	Finland	University of Oulu
Mniotype adusta	TLMF Lep 08776	PHLAI281-13	KM572648	BOLD:AAD5982	Austria	Tiroler Landesmuseum Ferdinandeum
Mniotype adusta	MM15875	LEFIH011-10	HM876647	BOLD:AAD5982	Finland	University of Oulu
Mniotype adusta	MM00575	LEFIB170-10	HM871074	BOLD:AAD5982	Finland	University of Oulu
Mniotype adusta	MM15876	LEFIH012-10	HM876648	BOLD:AAD5982	Finland	University of Oulu
Mniotype adusta	MM17469	LEFIJ844-10	JF853858	BOLD:AAD5982	Finland	University of Oulu
Mniotype satura	MM01736	LEFIA622-10	HM870871	BOLD:AAD0227	Finland	University of Oulu
Mniotype satura	MM07372	LEFID918-10	HM873668	BOLD:AAD0227	Finland	University of Oulu
Mniotype satura	TLMF Lep 08792	PHLAI297-13	KM573019	BOLD:AAD0227	Austria	Tiroler Landesmuseum Ferdinandeum
Mniotype satura	MM01737	LEFIA623-10	HM870872	BOLD:AAD0227	Finland	University of Oulu
Moma alpium	MM03763	LEFIC291-10	HM872135	BOLD:AAD7323	Finland	University of Oulu
Moma alpium	TLMF Lep 07834	PHLAV015-12	KM572661	BOLD:AAD7323	Austria	inatura, Dornbirn
Moma alpium	MM01536	LEFIA465-10	HM386805	BOLD:AAD7323	Finland	University of Oulu
Moma alpium	MM01537	LEFIA466-10	HM386806	BOLD:AAD7323	Finland	University of Oulu

Mompha conturbatella	MM08028	LEFID991-10	HM873741	BOLD:AAB7570	Finland	University of Oulu
Mompha conturbatella	MM00062	LEFIA1233-10	GU828591	BOLD:AAB7570	Finland	University of Oulu
Mompha conturbatella	MM02306	LEFIA1234-10	GU828679	BOLD:AAB7570	Finland	University of Oulu
Mompha conturbatella	MM09001	LEFIE415-10	HM874139	BOLD:AAB7570	Finland	University of Oulu
Mompha conturbatella	TLMF Lep 09392	PHLAI830-13	KM572875	BOLD:AAB7570	Austria	Tiroler Landesmuseum Ferdinandeum
Mompha lacteella	MM06972	LEFID848-10	HM873605	BOLD:AAB9184	Finland	University of Oulu
Mompha lacteella	MM17941	LEFIK366-10	JF854000	BOLD:AAB9184	Finland	University of Oulu
Mompha lacteella	MM06971	LEFID847-10	HM873604	BOLD:AAB9184	Finland	University of Oulu
Mompha lacteella	TLMF Lep 09170	PHLAI608-13	KM573448	BOLD:AAB9184	Austria	Tiroler Landesmuseum Ferdinandeum
Mompha langiella	MM04343	LEFIC566-10	HM872387	BOLD:AAF0121	Finland	University of Oulu
Mompha langiella	MM05970	LEFID182-10	HM872988	BOLD:AAF0121	Finland	University of Oulu
Mompha langiella	TLMF Lep 12494	LEATC512-13	KM573660	BOLD:AAF0121	Austria	Tiroler Landesmuseum Ferdinandeum
Mompha langiella	MM17395	LEFIJ770-10	JF853811	BOLD:AAF0121	Finland	University of Oulu
Mompha langiella	MM04342	LEFIC565-10	HM872386	BOLD:AAF0121	Finland	University of Oulu
Mompha locupletella	MM14441	LEFIG415-10	HM876091	BOLD:AAF2616	Finland	University of Oulu
Mompha locupletella	MM17956	LEFIK381-10	KM573421	BOLD:AAF2616	Finland	University of Oulu
Mompha locupletella	TLMF Lep 12519	LEATC537-13	KM573346	BOLD:AAF2616	Austria	Tiroler Landesmuseum Ferdinandeum
Mompha locupletella	MM14442	LEFIG416-10	HM876092	BOLD:AAF2616	Finland	University of Oulu
Mompha raschkiella	TLMF Lep 10053	LEATA446-13	KM572319	BOLD:AAE2679	Austria	inatura, Dornbirn
Mompha raschkiella	MM13617	LEFIG005-10	HM875685	BOLD:AAE2679	Finland	University of Oulu
Mompha raschkiella	MM00537	LEFIB149-10	HM871054	BOLD:AAE2679	Finland	University of Oulu
Mompha raschkiella	MM02579	LEFIB741-10	HM871619	BOLD:AAE2679	Finland	University of Oulu
Mompha subbistrigella	MM15601	LEFIG737-10	HM876393	BOLD:AAD0702	Finland	University of Oulu
Mompha subbistrigella	MM15600	LEFIG736-10	HM876392	BOLD:AAD0702	Finland	University of Oulu
Mompha subbistrigella	TLMF Lep 08051	PHLAV232-12	KM573643	BOLD:AAD0702	Austria	inatura, Dornbirn
Mompha subbistrigella	MM23198	COLFF465-13	KM572225	BOLD:AAD0702	Finland	University of Oulu, Zoological Museum
Mompha subbistrigella	TLMF Lep 09182	PHLAI620-13	KM572161	BOLD:AAD0702	Austria	Tiroler Landesmuseum Ferdinandeum
Mompha subbistrigella	MM17563	LEFIJ938-10	JF853905	BOLD:AAD0702	Finland	University of Oulu
Mompha subbistrigella	MM21101	LEFIJ1241-11	KM573553	BOLD:AAD0702	Finland	University of Oulu
Mompha terminella	TLMF Lep 09161	PHLAI599-13	KM573106	BOLD:AAZ8091	Austria	Tiroler Landesmuseum Ferdinandeum

Mompha terminella	MM05725	LEFIL706-10	KM573663	BOLD:AAZ8091	Finland	University of Oulu
Monochroa hornigi	MM18223	LEFIK648-10	JF854218	BOLD:AAK6885	Finland	University of Oulu
Monochroa hornigi	TLMF Lep 07510	PHLAG831-12	KM573013	BOLD:AAK6885	Austria	Tiroler Landesmuseum Ferdinandeum
Monochroa hornigi	MM02599	LEFIB752-10	HM871630	BOLD:AAK6885	Finland	University of Oulu
Monochroa hornigi	MM21112	LEFIJ1252-11	KM573708	BOLD:AAK6885	Finland	University of Oulu
Monochroa hornigi	MM21146	LEFIJ1286-11	KM572363	BOLD:AAK6885	Finland	University of Oulu
Monochroa hornigi	MM21147	LEFIJ1287-11	KM573384	BOLD:AAK6885	Finland	University of Oulu
Monochroa hornigi	MM18222	LEFIK647-10	JF854217	BOLD:AAK6885	Finland	University of Oulu
Monochroa hornigi	MM21094	LEFIJ1234-11	KM572514	BOLD:AAK6885	Finland	University of Oulu
Monochroa hornigi	MM17328	LEFIJ703-10	JF853780	BOLD:AAK6885	Finland	University of Oulu
Monochroa lutulentella	MM13609	LEFIF1000-10	HM874803	BOLD:AAE4780	Finland	University of Oulu
Monochroa lutulentella	MM12077	LEFIF502-10	HM875187	BOLD:AAE4780	Finland	University of Oulu
Monochroa lutulentella	MM02570	LEFIB737-10	HM871615	BOLD:AAE4780	Finland	University of Oulu
Monochroa lutulentella	MM03466	LEFIC127-10	HM871974	BOLD:AAE4780	Finland	University of Oulu
Monochroa lutulentella	TLMF Lep 07526	PHLAG847-12	KM573151	BOLD:AAE4780	Austria	Tiroler Landesmuseum Ferdinandeum
Monochroa servella	TLMF Lep 08060	PHLAV241-12	KM572700	BOLD:AAF9070	Austria	inatura, Dornbirn
Monochroa servella	TLMF Lep 07489	PHLAG810-12	KM572374	BOLD:AAF9070	Austria	inatura, Dornbirn
Monochroa servella	MM18219	LEFIK644-10	KM572794	BOLD:AAF9070	Finland	University of Oulu
Monochroa servella	MM15618	LEFIG754-10	HM876409	BOLD:AAF9070	Finland	University of Oulu
Monochroa servella	MM10452	LEFIF014-10	HM874728	BOLD:AAF9070	Finland	University of Oulu
Monopis laevigella	MM17303	LEFIJ678-10	JF853766	BOLD:AAB6631	Finland	University of Oulu
Monopis laevigella	MM21029	LEFIJ1169-11	KM572332	BOLD:AAB6631	Finland	University of Oulu
Monopis laevigella	MM21028	LEFIJ1168-11	KM572286	BOLD:AAB6631	Finland	University of Oulu
Monopis laevigella	MM21026	LEFIJ1166-11	KM573519	BOLD:AAB6631	Finland	University of Oulu
Monopis laevigella	MM21025	LEFIJ1165-11	KM571969	BOLD:AAB6631	Finland	University of Oulu
Monopis laevigella	MM17524	LEFIJ899-10	JF853887	BOLD:AAB6631	Finland	University of Oulu
Monopis laevigella	MM15527	LEFIG663-10	HM876324	BOLD:AAB6631	Finland	University of Oulu
Monopis laevigella	MM10119	LEFIE866-10	HM874584	BOLD:AAB6631	Finland	University of Oulu
Monopis laevigella	MM18625	LEFIL315-10	JN286245	BOLD:AAB6631	Finland	University of Oulu
Monopis laevigella	MM17522	LEFIJ897-10	JF853886	BOLD:AAB6631	Finland	University of Oulu
Monopis laevigella	MM17526	LEFIJ901-10	JF853889	BOLD:AAB6631	Finland	University of Oulu

Monopis laevigella	TLMF Lep 07970	PHLAV151-12	KM572597	BOLD:AAB6631	Austria	inatura, Dornbirn
Monopis laevigella	MM15526	LEFIG662-10	HM876323	BOLD:AAB6632	Finland	University of Oulu
Monopis laevigella	MM18626	LEFIL316-10	JN286246	BOLD:AAB6632	Finland	University of Oulu
Monopis laevigella	MM17525	LEFIJ900-10	JF853888	BOLD:AAB6632	Finland	University of Oulu
Monopis monachella	MM12377	LEFIF578-10	HM875262	BOLD:AAB1740	Finland	University of Oulu
Monopis monachella	MM13366	LEFIF926-10	HM875608	BOLD:AAB1740	Finland	University of Oulu
Monopis monachella	MM17249	LEFIJ624-10	JF853723	BOLD:AAB1740	Finland	University of Oulu
Monopis monachella	TLMF Lep 08436	PHLAH617-12	KM572999	BOLD:AAB1740	Austria	inatura, Dornbirn
Monopis monachella	MM11934	LEFIF450-10	HM875135	BOLD:AAB1740	Finland	University of Oulu
Monopis obviella	TLMF Lep 08054	PHLAV235-12	KM572042	BOLD:AAE4726	Austria	inatura, Dornbirn
Monopis obviella	TLMF Lep 09962	PHLAW165-13	KM572707	BOLD:AAE4726	Austria	inatura, Dornbirn
Monopis obviella	MM18928	LEFIL630-10	KM572043	BOLD:AAE4726	Finland	University of Oulu
Monopis obviella	MM21130	LEFIJ1270-11	KM573300	BOLD:AAE4726	Finland	University of Oulu
Monopis obviella	MM06790	LEFID732-10	HM873489	BOLD:AAE4726	Finland	University of Oulu
Montescardia tessulatellus	TLMF Lep 09919	PHLAW122-13	KM573419	BOLD:AAM0695	Austria	inatura, Dornbirn
Montescardia tessulatellus	MM03162	LEFIB972-10	HQ570280	BOLD:AAM0695	Finland	University of Oulu
Montescardia tessulatellus	MM18070	LEFIK495-10	JF854102	BOLD:AAM0695	Finland	University of Oulu
Montescardia tessulatellus	TLMF Lep 07918	PHLAV099-12	KM572482	BOLD:AAM0695	Austria	inatura, Dornbirn
Montescardia tessulatellus	MM08999	LEFIE414-10	HM874138	BOLD:AAM0695	Finland	University of Oulu
Montescardia tessulatellus	TLMF Lep 09191	PHLAI629-13	KM572906	BOLD:AAM0695	Austria	Tiroler Landesmuseum Ferdinandeum
Mormo maura	MM15942	LEFIJ342-10	KM572960	BOLD:AAD8730	Finland	University of Oulu
Mormo maura	TLMF Lep 09854	PHLAW057-13	KM572200	BOLD:AAD8730	Austria	Tiroler Landesmuseum Ferdinandeum
Mythimna conigera	MM01607	LEFIA524-10	KM572250	BOLD:AAC6915	Finland	University of Oulu
Mythimna conigera	MM01608	LEFIA525-10	KM573118	BOLD:AAC6915	Finland	University of Oulu
Mythimna conigera	TLMF Lep 08108	PHLAV289-12	KM572920	BOLD:AAC6915	Austria	inatura, Dornbirn
Mythimna conigera	MM04874	LEFIC757-10	HM872576	BOLD:AAC6915	Finland	University of Oulu

Mythimna ferrago	MM07263	LEFIJ103-10	KM572656	BOLD:AAC2354	Finland	University of Oulu
Mythimna ferrago	MM21045	LEFIJ1185-11	KM573622	BOLD:AAC2354	Finland	University of Oulu
Mythimna ferrago	TLMF Lep 08160	PHLAV341-12	KM572995	BOLD:AAC2354	Austria	inatura, Dornbirn
Mythimna ferrago	TLMF Lep 06127	PHLSA672-11	KM573155	BOLD:AAC2354	Austria	Tiroler Landesmuseum Ferdinandeum
Mythimna ferrago	MM01630	LEFIA543-10	KM572356	BOLD:AAC2354	Finland	University of Oulu
Mythimna ferrago	MM12241	LEFIJ183-10	KM572458	BOLD:AAC2354	Finland	University of Oulu
Mythimna ferrago	MM01631	LEFIA544-10	KM572440	BOLD:AAC2354	Finland	University of Oulu
Mythimna ferrago	MM10961	LEFIF219-10	HM874912	BOLD:AAC2355	Finland	University of Oulu
Mythimna ferrago	MM12661	LEFIJ197-10	KM573086	BOLD:AAC2355	Finland	University of Oulu
Mythimna impura	MM01606	LEFIA523-10	HQ963150	BOLD:AAB9151	Finland	University of Oulu
Mythimna impura	MM01605	LEFIA522-10	KM572706	BOLD:AAB9151	Finland	University of Oulu
Mythimna impura	TLMF Lep 05614	PHLAF444-11	KM572923	BOLD:AAB9151	Austria	Tiroler Landesmuseum Ferdinandeum
Mythimna impura	MM05124	LEFIC865-10	HM872683	BOLD:AAB9151	Finland	University of Oulu
Mythimna l-album	MM15955	LEFIJ355-10	KM572217	BOLD:AAD5882	Finland	University of Oulu
Mythimna l-album	TLMF Lep 08111	PHLAV292-12	KM572065	BOLD:AAD5882	Austria	inatura, Dornbirn
Mythimna pudorina	TLMF Lep 05626	PHLAF456-11	KM572693	BOLD:AAD6230	Austria	Tiroler Landesmuseum Ferdinandeum
Mythimna pudorina	MM18740	LEFIL442-10	JF854597	BOLD:AAD6230	Finland	University of Oulu
Mythimna pudorina	MM10930	LEFIF217-10	HM874910	BOLD:AAD6230	Finland	University of Oulu
Mythimna pudorina	MM18016	LEFIK441-10	JF854064	BOLD:AAD6230	Finland	University of Oulu
Mythimna straminea	MM10923	LEFIF215-10	HM874908	BOLD:AAC9826	Finland	University of Oulu
Mythimna straminea	MM05155	LEFIC879-10	HM872697	BOLD:AAC9826	Finland	University of Oulu
Mythimna straminea	MM17380	LEFIJ755-10	KM573347	BOLD:AAC9826	Finland	University of Oulu
Mythimna straminea	TLMF Lep 08185	PHLAV366-12	KM573331	BOLD:AAC9826	Austria	inatura, Dornbirn
Mythimna turca	TLMF Lep 08145	PHLAV326-12	KM572444	BOLD:AAF8311	Austria	inatura, Dornbirn
Mythimna turca	MM18907	LEFIL609-10	KM573625	BOLD:AAF8311	Finland	University of Oulu
Mythimna turca	MM17514	LEFIJ889-10	JF853881	BOLD:AAF8311	Finland	University of Oulu
Mythimna turca	MM11083	LEFIF300-10	HM874987	BOLD:AAF8311	Finland	University of Oulu
Naenia typica	MM04557	LEFIC623-10	HM872444	BOLD:AAC9760	Finland	University of Oulu
Naenia typica	MM04897	LEFIC770-10	HM872589	BOLD:AAC9760	Finland	University of Oulu
Naenia typica	MM09740	LEFIA863-10	HM387001	BOLD:AAC9760	Finland	University of Oulu
Naenia typica	TLMF Lep 08184	PHLAV365-12	KM573678	BOLD:AAC9760	Austria	inatura, Dornbirn

Nemapogon cloacella	MM14228	LEFIG282-10	HM875961	BOLD:AAC5133	Finland	University of Oulu
Nemapogon cloacella	MM03836	LEFIC331-10	HM872175	BOLD:AAC5133	Finland	University of Oulu
Nemapogon cloacella	MM03160	LEFIB971-10	HM871845	BOLD:AAC5133	Finland	University of Oulu
Nemapogon cloacella	MM02294	LEFIB591-10	HM871470	BOLD:ABY6823	Finland	University of Oulu
Nemapogon cloacella	TLMF Lep 09178	PHLAI616-13	KM573280	BOLD:ABY6823	Austria	Tiroler Landesmuseum Ferdinandeum
Nemapogon wolffiella	TLMF Lep 09894	PHLAW097-13	KM572282	BOLD:AAD6594	Austria	Tiroler Landesmuseum Ferdinandeum
Nemapogon wolffiella	MM08049	LEFID999-10	HM873749	BOLD:AAD6594	Finland	University of Oulu
Nemapogon wolffiella	MM08701	LEFIE322-10	HM874048	BOLD:AAD6594	Finland	University of Oulu
Nemapogon wolffiella	MM08048	LEFID998-10	HM873748	BOLD:AAD6594	Finland	University of Oulu
Nematopogon pilella	TLMF Lep 12508	LEATC526-13	KM573440	BOLD:AAC5324	Austria	Tiroler Landesmuseum Ferdinandeum
Nematopogon pilella	MM03896	LEFIJ061-10	KM573238	BOLD:AAC5324	Finland	University of Oulu
Nematopogon pilella	MM13990	LEFIJ237-10	JF853482	BOLD:AAC5324	Finland	University of Oulu
Nematopogon pilella	MM08574	LEFIE247-10	HM873981	BOLD:AAC5324	Finland	University of Oulu
Nematopogon robertella	TLMF Lep 07922	PHLAV103-12	KM572521	BOLD:AAD1738	Austria	inatura, Dornbirn
Nematopogon robertella	BIOUG04116-D12	GMFIM244-13	KM572056	BOLD:AAF2382	Finland	Biodiversity Institute of Ontario
Nematopogon robertella	MM03375	LEFIC079-10	HM871947	BOLD:AAF2382	Finland	University of Oulu
Nematopogon robertella	MM06031	LEFID203-10	HM873006	BOLD:AAF2382	Finland	University of Oulu
Nematopogon robertella	MM14134	LEFIG230-10	HM875909	BOLD:AAF2382	Finland	University of Oulu
Nematopogon robertella	BIOUG04116-D11	GMFIM243-13	KM573039	BOLD:AAF2382	Finland	Biodiversity Institute of Ontario
Nematopogon schwarziellus	TLMF Lep 07921	PHLAV102-12	KM572011	BOLD:ACE6316	Austria	inatura, Dornbirn
Nematopogon schwarziellus	TLMF Lep 07479	PHLAG800-12	KM573016	BOLD:ACE6316	Austria	inatura, Dornbirn
Nematopogon schwarziellus	MM14030	LEFIG169-10	HM875849	BOLD:ACE6316	Finland	University of Oulu
Nematopogon schwarziellus	MM10014	LEFIJ145-10	JF853458	BOLD:ACE6316	Finland	University of Oulu
Nematopogon schwarziellus	MM10013	LEFIE814-10	HM874533	BOLD:ACE6316	Finland	University of Oulu
Nematopogon swammerdamella	MM05293	LEFIA1243-10	GU828715	BOLD:AAD1739	Finland	University of Oulu

Nematopogon swammerdamella	TLMF Lep 07920	PHLAV101-12	KM572853	BOLD:AAD1739	Austria	inatura, Dornbirn
Nematopogon swammerdamella	MM13371	LEFIF929-10	HM875611	BOLD:AAD1739	Finland	University of Oulu
Nematopogon swammerdamella	MM13975	LEFIG140-10	HM875820	BOLD:AAD1739	Finland	University of Oulu
Nematopogon swammerdamella	MM05961	LEFID177-10	HM872984	BOLD:AAD1739	Finland	University of Oulu
Nemophora degeerella	MM15509	LEFIG645-10	HM876308	BOLD:AAC1892	Finland	University of Oulu
Nemophora degeerella	MM09760	LEFIA883-10	HM387020	BOLD:AAC1892	Finland	University of Oulu
Nemophora degeerella	MM15508	LEFIG644-10	HM876307	BOLD:AAC1892	Finland	University of Oulu
Nemophora degeerella	MM09554	LEFIE624-10	HM874347	BOLD:AAC1892	Finland	University of Oulu
Nemophora degeerella	TLMF Lep 09164	PHLAI602-13	KM573610	BOLD:AAC1892	Austria	Tiroler Landesmuseum Ferdinandeum
Nemophora degeerella	MM06587	LEFID582-10	HM873347	BOLD:AAC1892	Finland	University of Oulu
Nemophora metallica	TLMF Lep 08014	PHLAV195-12	KM573376	BOLD:AAE5236	Austria	inatura, Dornbirn
Nemophora metallica	MM06621	LEFID607-10	HM873372	BOLD:ACF0411	Finland	University of Oulu
Nemophora metallica	MM06622	LEFID608-10	HM873373	BOLD:ACF0411	Finland	University of Oulu
Nemophora metallica	MM06620	LEFID606-10	HM873371	BOLD:ACF0411	Finland	University of Oulu
Neofaculta ericetella	TLMF Lep 08078	PHLAV259-12	KM573512	BOLD:AAD1501	Austria	inatura, Dornbirn
Neofaculta ericetella	MM03909	LEFIC379-10	HM872222	BOLD:AAD1501	Finland	University of Oulu
Neofaculta ericetella	MM13971	LEFIG139-10	HM875819	BOLD:AAD1501	Finland	University of Oulu
Neofaculta ericetella	MM13970	LEFIG138-10	HM875818	BOLD:AAD1501	Finland	University of Oulu
Neofaculta ericetella	TLMF Lep 09949	PHLAW152-13	KM573386	BOLD:AAD1501	Austria	inatura, Dornbirn
Neofaculta infernella	MM05472	LEFID030-10	HM872844	BOLD:AAC1362	Finland	University of Oulu
Neofaculta infernella	MM18251	LEFIK676-10	JF854244	BOLD:AAC1362	Finland	University of Oulu
Neofaculta infernella	MM03437	LEFIC109-10	HQ570300	BOLD:AAC1362	Finland	University of Oulu
Neofaculta infernella	MM17908	LEFIK333-10	JX034580	BOLD:AAC1362	Finland	University of Oulu
Neofaculta infernella	MM09029	LEFIJ130-10	JX034600	BOLD:AAC1362	Finland	University of Oulu
Neofaculta infernella	MM18250	LEFIK675-10	JF854243	BOLD:AAC1362	Finland	University of Oulu
Neofaculta infernella	MM13508	LEFIJ232-10	JX034649	BOLD:AAC1363	Finland	University of Oulu
Neofaculta infernella	MM02323	LEFIB606-10	HM871485	BOLD:AAC1363	Finland	University of Oulu

Neofaculta infernella	MM13507	LEFIF948-10	HM875629	BOLD:AAC1363	Finland	University of Oulu
Neofaculta infernella	MM21173	LEFIJ1313-11	KM573566	BOLD:AAC1363	Finland	University of Oulu
Neofaculta infernella	MM21174	LEFIJ1314-11	KM572812	BOLD:AAC1363	Finland	University of Oulu
Neofaculta infernella	MM04223	LEFIC514-10	HM872338	BOLD:AAC1363	Finland	University of Oulu
Neofaculta infernella	MM17906	LEFIK331-10	JX034666	BOLD:AAC1363	Finland	University of Oulu
Neofaculta infernella	MM17907	LEFIK332-10	JX034662	BOLD:AAC1363	Finland	University of Oulu
Neofaculta infernella	TLMF Lep 00867	PHLAB067-10	HQ968241	BOLD:AAC1363	Austria	Tiroler Landesmuseum Ferdinandeum
Neofaculta infernella	MM18249	LEFIK674-10	JF854242	BOLD:AAC1363	Finland	University of Oulu
Neofaculta infernella	TLMF Lep 00913	PHLAB113-10	HM381485	BOLD:AAC1363	Austria	Tiroler Landesmuseum Ferdinandeum
Nephopterix angustella	TLMF Lep 08217	PHLAH398-12	KM572770	BOLD:AAD0751	Austria	inatura, Dornbirn
Nephopterix angustella	MM17234	LEFIJ609-10	JF853709	BOLD:AAD0751	Finland	University of Oulu
Noctua comes	TLMF Lep 08761	PHLAI266-13	KM573645	BOLD:AAA2633	Austria	Tiroler Landesmuseum Ferdinandeum
Noctua comes	MM11092	LEFIF304-10	HM874991	BOLD:AAA2633	Finland	University of Oulu
Noctua comes	MM12550	LEFIF643-10	HM875327	BOLD:AAA2633	Finland	University of Oulu
Noctua comes	MM12551	LEFIF644-10	HM875328	BOLD:AAA2633	Finland	University of Oulu
Noctua fimbriata	MM12578	LEFIF652-10	HM875336	BOLD:AAA6454	Finland	University of Oulu
Noctua fimbriata	MM04752	LEFIA1247-10	GU828705	BOLD:AAA6454	Finland	University of Oulu
Noctua fimbriata	MM10816	LEFIF199-10	HM874893	BOLD:AAA6454	Finland	University of Oulu
Noctua fimbriata	TLMF Lep 08553	PHLAH734-12	KM573415	BOLD:AAA6454	Austria	inatura, Dornbirn
Noctua fimbriata	MM04753	LEFIC711-10	HM872532	BOLD:AAA6454	Finland	University of Oulu
Noctua janthe	TLMF Lep 07722	PHLAH283-12	KM572816	BOLD:AAA7574	Austria	Tiroler Landesmuseum Ferdinandeum
Noctua janthe	MM19920	LEFII270-11	KM573337	BOLD:AAA7574	Finland	Research Collection of E. Laasonen
Noctua janthe	TLMF Lep 07723	PHLAH284-12	KM573616	BOLD:AAA7574	Austria	Tiroler Landesmuseum Ferdinandeum
Noctua janthina	MM18742	LEFIL444-10	KM572997	BOLD:ABZ6181	Finland	University of Oulu
Noctua janthina	MM17492	LEFIJ867-10	JF853869	BOLD:ABZ6181	Finland	University of Oulu
Noctua janthina	TLMF Lep 00292	PHLAA252-09	HM425791	BOLD:ABZ6181	Austria	Tiroler Landesmuseum Ferdinandeum
Noctua janthina	MM15961	LEFIJ361-10	KM572780	BOLD:ABZ6181	Finland	University of Oulu
Noctua janthina	MM19919	LEFII269-11	KM573311	BOLD:ABZ6181	Finland	Research Collection of E. Laasonen
Noctua pronuba	MM01686	LEFIA581-10	HM870830	BOLD:AAA2632	Finland	University of Oulu
Noctua pronuba	MM04912	LEFIC780-10	HM872599	BOLD:AAA2632	Finland	University of Oulu
Noctua pronuba	MM01685	LEFIA580-10	HM870829	BOLD:AAA2632	Finland	University of Oulu

Noctua pronuba	TLMF Lep 08154	PHLAV335-12	KM573432	BOLD:AAA2632	Austria	inatura, Dornbirn
Nola confusalis	MM03773	LEFIC298-10	HM872142	BOLD:AAB5563	Finland	University of Oulu
Nola confusalis	MM13887	LEFIG103-10	HM875783	BOLD:AAB5563	Finland	University of Oulu
Nola confusalis	MM07911	LEFID957-10	HM873707	BOLD:AAB5563	Finland	University of Oulu
Nola confusalis	TLMF Lep 04644	PHLAE329-11	JN284314	BOLD:AAB5563	Austria	Tiroler Landesmuseum Ferdinandeum
Nomophila noctuella	MM17341	LEFIJ716-10	JF853789	BOLD:AAA7880	Finland	University of Oulu
Nomophila noctuella	MM04206	LEFIC499-10	HM872324	BOLD:AAA7880	Finland	University of Oulu
Nomophila noctuella	TLMF Lep 08197	PHLAV378-12	KM572434	BOLD:AAA7880	Austria	inatura, Dornbirn
Nomophila noctuella	TLMF Lep 06175	PHLSA720-11	KM572409	BOLD:AAA7880	Austria	Tiroler Landesmuseum Ferdinandeum
Nomophila noctuella	MM05508	LEFID044-10	HM872858	BOLD:AAA7880	Finland	University of Oulu
Nonagria typhae	TLMF Lep 08863	PHLAI368-13	KM572469	BOLD:AAD0414	Austria	Tiroler Landesmuseum Ferdinandeum
Nonagria typhae	MM10213	LEFIE902-10	HM874619	BOLD:AAD0414	Finland	University of Oulu
Nonagria typhae	MM18026	LEFIK451-10	JF854072	BOLD:AAD0414	Finland	University of Oulu
Nonagria typhae	MM18025	LEFIK450-10	JF854071	BOLD:AAD0414	Finland	University of Oulu
Notocelia cynosbatella	MM01972	LEFIB449-10	HM871348	BOLD:AAC3246	Finland	University of Oulu
Notocelia cynosbatella	MM22899	LEFIJ2047-13	KM572843	BOLD:AAC3246	Finland	University of Oulu
Notocelia cynosbatella	MM03018	LEFIB910-10	HM871787	BOLD:AAC3246	Finland	University of Oulu
Notocelia cynosbatella	TLMF Lep 07962	PHLAV143-12	KM573594	BOLD:AAC3246	Austria	inatura, Dornbirn
Notocelia cynosbatella	MM13185	LEFIF840-10	HM875523	BOLD:AAC3246	Finland	University of Oulu
Notocelia rosaecolana	MM18335	LEFIK760-10	JF854317	BOLD:AAC1134	Finland	University of Oulu
Notocelia rosaecolana	TLMF Lep 10028	LEATA421-13	KM572354	BOLD:AAC1134	Austria	inatura, Dornbirn
Notocelia rosaecolana	MM08020	LEFID987-10	HM873737	BOLD:AAC1134	Finland	University of Oulu
Notocelia rosaecolana	MM08021	LEFID988-10	HM873738	BOLD:AAC1134	Finland	University of Oulu
Notocelia tetragonana	MM19240	LEFIL240-10	KM573601	BOLD:AAC0717	Finland	University of Oulu
Notocelia tetragonana	TLMF Lep 08090	PHLAV271-12	KM572137	BOLD:AAC0717	Austria	inatura, Dornbirn
Notocelia tetragonana	MM19906	LEFII256-11	KM573617	BOLD:AAC0717	Finland	Research Collection of E. Laasonen
Notocelia tetragonana	MM19907	LEFII257-11	KM572131	BOLD:AAC0717	Finland	Research Collection of E. Laasonen
Notocelia uddmanniana	MM01970	LEFIB448-10	HM871347	BOLD:AAC1148	Finland	University of Oulu
Notocelia uddmanniana	MM13212	LEFIF853-10	HM875536	BOLD:AAC1148	Finland	University of Oulu
Notocelia uddmanniana	MM03016	LEFIB909-10	HM871786	BOLD:AAC1148	Finland	University of Oulu
Notocelia uddmanniana	TLMF Lep 08024	PHLAV205-12	KM572952	BOLD:AAC1148	Austria	inatura, Dornbirn

Notodonta dromedarius	TLMF Lep 08532	PHLAH713-12	KM573592	BOLD:AAC1145	Austria	inatura, Dornbirn
Notodonta dromedarius	TLMF Lep 07872	PHLAV053-12	KM573115	BOLD:AAC1145	Austria	inatura, Dornbirn
Notodonta dromedarius	TLMF Lep 08511	PHLAH692-12	KM571998	BOLD:AAC1145	Austria	inatura, Dornbirn
Notodonta dromedarius	TLMF Lep 04662	PHLAE347-11	JN274482	BOLD:AAC1145	Austria	Tiroler Landesmuseum Ferdinandeum
Notodonta dromedarius	TLMF Lep 08141	PHLAV322-12	KM572664	BOLD:AAC1145	Austria	inatura, Dornbirn
Notodonta dromedarius	MM10751	LEFIF179-10	HM874873	BOLD:AAC1146	Finland	University of Oulu
Notodonta dromedarius	MM07685	LEFID937-10	HM873687	BOLD:AAC1146	Finland	University of Oulu
Notodonta dromedarius	MM04512	LEFIC593-10	HM872414	BOLD:AAC1146	Finland	University of Oulu
Notodonta dromedarius	MM00121	LEFIB030-10	HM870941	BOLD:AAC1146	Finland	University of Oulu
Notodonta dromedarius	MM00998	LEFIA1250-10	GU828653	BOLD:AAC1146	Finland	University of Oulu
Notodonta ziczac	TLMF Lep 07828	PHLAV009-12	KM573646	BOLD:ABY9866	Austria	inatura, Dornbirn
Notodonta ziczac	MM04923	LEFIC786-10	HM872605	BOLD:ABY9866	Finland	University of Oulu
Notodonta ziczac	MM01002	LEFIA083-10	HM396430	BOLD:ABY9866	Finland	University of Oulu
Notodonta ziczac	MM01001	LEFIA082-10	HM396429	BOLD:ABY9866	Finland	University of Oulu
Nycteola degenerana	MM01834	LEFIA691-10	HM386836	BOLD:AAE2641	Finland	University of Oulu
Nycteola degenerana	TLMF Lep 04645	PHLAE330-11	JN278423	BOLD:AAE2641	Austria	Tiroler Landesmuseum Ferdinandeum
Nycteola degenerana	BIOUG04490-E10	GMFID734-12	KM572626	BOLD:AAE2641	Finland	Biodiversity Institute of Ontario
Nycteola degenerana	MM04623	LEFIC663-10	HM872484	BOLD:AAE2641	Finland	University of Oulu
Nycteola degenerana	MM00135	LEFIA1252-10	GU828612	BOLD:AAE2641	Finland	University of Oulu
Nycteola degenerana	MM01835	LEFIA692-10	HM386837	BOLD:AAE2641	Finland	University of Oulu
Nycteola degenerana	MM02930	LEFIB871-10	HM871748	BOLD:AAE2641	Finland	University of Oulu
Nycteola revayana	MM05998	LEFID198-10	HM873001	BOLD:AAB8993	Finland	University of Oulu
Nycteola revayana	MM14490	LEFIG452-10	HM876128	BOLD:AAB8993	Finland	University of Oulu
Nycteola revayana	TLMF Lep 07908	PHLAV089-12	KM573190	BOLD:AAB8993	Austria	inatura, Dornbirn
Nycteola revayana	MM18660	LEFIL350-10	JN278407	BOLD:AAB8993	Finland	University of Oulu
Nycteola revayana	MM05407	LEFIC994-10	HM872809	BOLD:AAB8993	Finland	University of Oulu
Nymphula nitidulata	MM00767	LEFIB279-10	HM871181	BOLD:AAE2813	Finland	University of Oulu
Nymphula nitidulata	MM13005	LEFIF775-10	HM875459	BOLD:AAF2874	Finland	University of Oulu
Nymphula nitidulata	TLMF Lep 08414	PHLAH595-12	KM572207	BOLD:AAF2874	Austria	inatura, Dornbirn
Nymphula nitidulata	MM01897	LEFIB419-10	HM871318	BOLD:AAF2874	Finland	University of Oulu
Ochlodes sylvanus	MM02929	LEFIB870-10	HM871747	BOLD:AAB4864	Finland	University of Oulu

Ochlodes sylvanus	MM02928	LEFIB869-10	HM871746	BOLD:AAB4864	Finland	University of Oulu
Ochlodes sylvanus	MM17112	LEFIJ487-10	JF853612	BOLD:AAB4864	Finland	University of Oulu
Ochlodes sylvanus	TLMF Lep 08286	PHLAH467-12	KM572728	BOLD:AAB4864	Austria	inatura, Dornbirn
Ochropacha duplaris	MM01230	LEFIA207-10	HM396551	BOLD:AAC6798	Finland	University of Oulu
Ochropacha duplaris	TLMF Lep 04643	PHLAE328-11	JN307421	BOLD:AAC6798	Austria	Tiroler Landesmuseum Ferdinandeum
Ochropacha duplaris	MM08165	LEFIE051-10	HM873799	BOLD:AAC6798	Finland	University of Oulu
Ochropacha duplaris	MM01231	LEFIA208-10	HM396552	BOLD:AAC6798	Finland	University of Oulu
Ochropleura plecta	MM01585	LEFIA503-10	KM573020	BOLD:AAA3074	Finland	University of Oulu
Ochropleura plecta	MM01584	LEFIA502-10	KM572786	BOLD:AAA3074	Finland	University of Oulu
Ochropleura plecta	MM22894	LEFIJ2042-13	KM572277	BOLD:ABY0189	Finland	University of Oulu
Ochropleura plecta	TLMF Lep 07859	PHLAV040-12	KM573097	BOLD:ACE7640	Austria	inatura, Dornbirn
Ocnerostoma friesei	MM16016	LEFIL727-10	JF854703	BOLD:AAF4475	Finland	University of Oulu
Ocnerostoma friesei	TLMF Lep 08484	PHLAH665-12	KM572993	BOLD:AAF4475	Austria	inatura, Dornbirn
Ocnerostoma friesei	MM15536	LEFIG672-10	HM876333	BOLD:AAF4475	Finland	University of Oulu
Ocnerostoma friesei	MM00545	LEFIB155-10	HM871060	BOLD:AAF4475	Finland	University of Oulu
Ocnerostoma friesei	MM15535	LEFIG671-10	HM876332	BOLD:AAF4475	Finland	University of Oulu
Odezia atrata	TLMF Lep 09840	PHLAW043-13	KM572298	BOLD:AAD5242	Austria	Tiroler Landesmuseum Ferdinandeum
Odezia atrata	MM03239	LEFIC026-10	HM871896	BOLD:AAD5243	Finland	University of Oulu
Odezia atrata	MM06562	LEFID568-10	HM873333	BOLD:AAD5243	Finland	University of Oulu
Odezia atrata	MM01378	LEFIA331-10	HM386674	BOLD:AAD5243	Finland	University of Oulu
Odezia atrata	TLMF Lep 09514	LEATA097-13	KM571962	BOLD:ACG1959	Austria	Tiroler Landesmuseum Ferdinandeum
Odontopera bidentata	MM00648	LEFIB203-10	HM871107	BOLD:AAB6560	Finland	University of Oulu
Odontopera bidentata	MM04666	LEFIC687-10	HM872508	BOLD:AAB6560	Finland	University of Oulu
Odontopera bidentata	TLMF Lep 04659	PHLAE344-11	JN269341	BOLD:AAB6560	Austria	Tiroler Landesmuseum Ferdinandeum
Odontopera bidentata	MM01486	LEFIA423-10	HM386765	BOLD:AAB6560	Finland	University of Oulu
Odontosia carmelita	TLMF Lep 04625	PHLAE310-11	JN274447	BOLD:AAD7238	Austria	Tiroler Landesmuseum Ferdinandeum
Odontosia carmelita	MM00987	LEFIA069-10	HM396416	BOLD:AAD7238	Finland	University of Oulu
Odontosia carmelita	MM04501	LEFIC584-10	HM872405	BOLD:AAD7238	Finland	University of Oulu
Odontosia carmelita	MM00988	LEFIA070-10	HM396417	BOLD:AAD7238	Finland	University of Oulu
Oecophora bractella	TLMF Lep 09171	PHLAI609-13	KM571970	BOLD:AAC1073	Austria	Tiroler Landesmuseum Ferdinandeum
Oecophora bractella	MM03831	LEFIA1258-10	GU828695	BOLD:AAC1073	Finland	University of Oulu

Oecophora bractella	MM03842	LEFIC335-10	HM872179	BOLD:AAC1073	Finland	University of Oulu
Oecophora bractella	MM13190	LEFIF844-10	HM875527	BOLD:AAC1073	Finland	University of Oulu
Oecophora bractella	MM14375	LEFIG370-10	HM876047	BOLD:AAC1073	Finland	University of Oulu
Oidaematophorus lithodactyla	MM09587	LEFIE639-10	HM874362	BOLD:AAD8626	Finland	University of Oulu
Oidaematophorus lithodactyla	MM09588	LEFIE640-10	HM874363	BOLD:AAD8626	Finland	University of Oulu
Oidaematophorus lithodactyla	MM10393	LEFIE971-10	HM874688	BOLD:AAD8626	Finland	University of Oulu
Oidaematophorus lithodactyla	TLMF Lep 07513	PHLAG834-12	KM572777	BOLD:AAD8626	Austria	Tiroler Landesmuseum Ferdinandeum
Oidaematophorus rogenhoferi	MM06583	LEFID580-10	HM873345	BOLD:AAE5830	Finland	University of Oulu
Oidaematophorus rogenhoferi	MM06393	LEFID454-10	HM873225	BOLD:AAE5830	Finland	University of Oulu
Oidaematophorus rogenhoferi	MM06582	LEFID579-10	HM873344	BOLD:AAE5830	Finland	University of Oulu
Oidaematophorus rogenhoferi	TLMF Lep 08747	PHLAH943-12	KM573073	BOLD:AAE5830	Austria	Tiroler Landesmuseum Ferdinandeum
Olethreutes arcuella	MM18282	LEFIK707-10	JF854271	BOLD:AAC2510	Finland	University of Oulu
Olethreutes arcuella	MM09557	LEFIE626-10	HM874349	BOLD:AAC2510	Finland	University of Oulu
Olethreutes arcuella	MM13294	LEFIF888-10	HM875570	BOLD:AAC2510	Finland	University of Oulu
Olethreutes arcuella	TLMF Lep 09158	PHLAI596-13	KM572613	BOLD:AAC2510	Austria	Tiroler Landesmuseum Ferdinandeum
Olethreutinae	BIOUG04116-E03	GMFIO697-13	KM573472	BOLD:AAC3531	Finland	Biodiversity Institute of Ontario
Oligia strigilis	MM01667	LEFIA573-10	HM870822	BOLD:AAB4833	Finland	University of Oulu
Oligia strigilis	TLMF Lep 07887	PHLAV068-12	KM572477	BOLD:AAB4833	Austria	inatura, Dornbirn
Oligia strigilis	MM01666	LEFIA572-10	HM870821	BOLD:AAB4833	Finland	University of Oulu
Oligia strigilis	TLMF Lep 07860	PHLAV041-12	KM573096	BOLD:AAB4833	Austria	inatura, Dornbirn
Oligia strigilis	MM09474	LEFIE577-10	HM874300	BOLD:AAB4833	Finland	University of Oulu
Oligia strigilis	MM03663	LEFIC251-10	HM872095	BOLD:AAB4833	Finland	University of Oulu
Oncocera semirubella	MM03576	LEFIC207-10	HM872051	BOLD:AAD7672	Finland	University of Oulu

Oncocera semirubella	MM13011	LEFIF779-10	HM875463	BOLD:AAD7672	Finland	University of Oulu
Oncocera semirubella	MM01878	LEFIB410-10	HM871309	BOLD:AAD7672	Finland	University of Oulu
Oncocera semirubella	TLMF Lep 09156	PHLAI594-13	KM573508	BOLD:AAD7672	Austria	Tiroler Landesmuseum Ferdinandeum
Operophtera brumata	MM14726	LEFIG572-10	HM876243	BOLD:AAA3963	Finland	University of Oulu
Operophtera brumata	MM08270	LEFIE091-10	HM873838	BOLD:AAA3963	Finland	University of Oulu
Operophtera brumata	MM08226	LEFIE076-10	HM873823	BOLD:AAA3963	Finland	University of Oulu
Operophtera brumata	TLMF Lep 06802	PHLAG408-12	KM572660	BOLD:AAA3963	Austria	Tiroler Landesmuseum Ferdinandeum
Operophtera fagata	MM02698	LEFIB790-10	HM871667	BOLD:AAD0141	Finland	University of Oulu
Operophtera fagata	TLMF Lep 08788	PHLAI293-13	KM573587	BOLD:AAD0141	Austria	Tiroler Landesmuseum Ferdinandeum
Operophtera fagata	MM18445	LEFIK870-10	KM573195	BOLD:AAD0141	Finland	University of Oulu
Operophtera fagata	MM08269	LEFIE090-10	HM873837	BOLD:AAD0141	Finland	University of Oulu
Opisthograptis luteolata	MM01350	LEFIA304-10	HM386647	BOLD:AAA9865	Finland	University of Oulu
Opisthograptis luteolata	MM04672	LEFIC691-10	HM872512	BOLD:AAA9865	Finland	University of Oulu
Opisthograptis luteolata	TLMF Lep 04632	PHLAE317-11	JN269337	BOLD:AAA9865	Austria	Tiroler Landesmuseum Ferdinandeum
Opisthograptis luteolata	MM12768	LEFIF716-10	HM875400	BOLD:AAA9865	Finland	University of Oulu
Orgyia antiqua	TLMF Lep 12460	LEATC478-13	KM573141	BOLD:AAA6432	Austria	Tiroler Landesmuseum Ferdinandeum
Orgyia antiqua	MM04770	LEFIC718-10	HM872539	BOLD:AAA6432	Finland	University of Oulu
Orgyia antiqua	MM09339	LEFIE512-10	HM874236	BOLD:AAA6432	Finland	University of Oulu
Orgyia antiqua	MM12217	LEFIF538-10	HM875223	BOLD:AAA6432	Finland	University of Oulu
Orthonama obstipata	MM18698	LEFIL400-10	KM573680	BOLD:AAA3431	Finland	University of Oulu
Orthonama obstipata	TLMF Lep 08787	PHLAI292-13	KM572737	BOLD:AAA3431	Austria	Tiroler Landesmuseum Ferdinandeum
Orthosia cerasi	TLMF Lep 07871	PHLAV052-12	KM572371	BOLD:AAC3426	Austria	inatura, Dornbirn
Orthosia cerasi	MM04612	LEFIC656-10	HM872477	BOLD:AAC3426	Finland	University of Oulu
Orthosia cerasi	MM12106	LEFIF508-10	HM875193	BOLD:AAC3426	Finland	University of Oulu
Orthosia cerasi	MM04613	LEFIC657-10	HM872478	BOLD:AAC3426	Finland	University of Oulu
Orthosia cruda	MM04616	LEFIC659-10	HM872480	BOLD:AAD9024	Finland	University of Oulu
Orthosia cruda	MM04617	LEFIC660-10	HM872481	BOLD:AAD9024	Finland	University of Oulu
Orthosia cruda	TLMF Lep 08808	PHLAI313-13	KM572823	BOLD:AAD9024	Austria	Tiroler Landesmuseum Ferdinandeum
Orthosia cruda	MM04618	LEFIC661-10	HM872482	BOLD:AAD9024	Finland	University of Oulu
Orthosia gothica	MM01574	LEFIA493-10	KM573681	BOLD:AAB6211	Finland	University of Oulu
Orthosia gothica	MM08419	LEFIE164-10	HM873910	BOLD:AAB6211	Finland	University of Oulu

Orthosia gothica	TLMF Lep 08852	PHLAI357-13	KM573224	BOLD:AAB6211	Austria	Tiroler Landesmuseum Ferdinandeum
Orthosia gothica	MM01573	LEFIA492-10	KM572558	BOLD:AAB6211	Finland	University of Oulu
Orthosia gothica	MM02733	LEFIB807-10	HM871684	BOLD:AAB6211	Finland	University of Oulu
Orthosia gracilis	MM01566	LEFIA488-10	KM573313	BOLD:AAC2300	Finland	University of Oulu
Orthosia gracilis	MM01567	LEFIA489-10	KM573640	BOLD:AAC2300	Finland	University of Oulu
Orthosia gracilis	MM04504	LEFIC587-10	HM872408	BOLD:AAC2300	Finland	University of Oulu
Orthosia gracilis	TLMF Lep 08809	PHLAI314-13	KM572063	BOLD:AAC2300	Austria	Tiroler Landesmuseum Ferdinandeum
Orthosia incerta	TLMF Lep 08856	PHLAI361-13	KM572955	BOLD:ABY5277	Austria	Tiroler Landesmuseum Ferdinandeum
Orthosia incerta	BIOUG04118-A04	GMFID724-12	KM572523	BOLD:ABY5277	Finland	Biodiversity Institute of Ontario
Orthosia incerta	MM02743	LEFIB810-10	HM871687	BOLD:ABY5277	Finland	University of Oulu
Orthosia incerta	MM01563	LEFIA486-10	KM571964	BOLD:ABY5277	Finland	University of Oulu
Orthosia incerta	MM01564	LEFIA487-10	KM572067	BOLD:ABY5277	Finland	University of Oulu
Orthosia opima	TLMF Lep 08853	PHLAI358-13	KM573136	BOLD:ACE8591	Austria	Tiroler Landesmuseum Ferdinandeum
Orthosia opima	MM01562	LEFIA485-10	KM572803	BOLD:ACE8591	Finland	University of Oulu
Orthosia opima	MM00415	LEFIB103-10	HM871010	BOLD:ACE8591	Finland	University of Oulu
Orthosia opima	MM01561	LEFIA484-10	KM571963	BOLD:ACE8591	Finland	University of Oulu
Orthosia populeti	TLMF Lep 08855	PHLAI360-13	KM573293	BOLD:AAD3183	Austria	Tiroler Landesmuseum Ferdinandeum
Orthosia populeti	MM00453	LEFIB111-10	HM871018	BOLD:AAD3183	Finland	University of Oulu
Orthosia populeti	MM01570	LEFIA491-10	KM572029	BOLD:AAD3183	Finland	University of Oulu
Orthosia populeti	MM01569	LEFIA490-10	KM573056	BOLD:AAD3183	Finland	University of Oulu
Orthosia populeti	MM13860	LEFIA932-10	HM387066	BOLD:AAD3183	Finland	University of Oulu
Orthotaenia undulana	BIOUG04116-A07	GMFIE827-12	KM573653	BOLD:AAB4021	Finland	Biodiversity Institute of Ontario
Orthotaenia undulana	MM03014	LEFIB908-10	HM871785	BOLD:AAB4021	Finland	University of Oulu
Orthotaenia undulana	MM11910	LEFIF443-10	HM875128	BOLD:AAB4021	Finland	University of Oulu
Orthotaenia undulana	TLMF Lep 09920	PHLAW123-13	KM572528	BOLD:AAB4021	Austria	inatura, Dornbirn
Orthotaenia undulana	MM08483	LEFIE182-10	HM873927	BOLD:AAB4021	Finland	University of Oulu
Orthotaenia undulana	MM18287	LEFIK712-10	JF854275	BOLD:AAB4021	Finland	University of Oulu
Orthotaenia undulana	MM13293	LEFIF887-10	HM875569	BOLD:AAB4021	Finland	University of Oulu
Orthotaenia undulana	MM02068	LEFIB492-10	HM871387	BOLD:AAB4021	Finland	University of Oulu
Orthotaenia undulana	BIOUG04490-F01	GMFIE841-12	KM573588	BOLD:AAB4021	Finland	Biodiversity Institute of Ontario
Ourapteryx sambucaria	MM09715	LEFIA840-10	HM386980	BOLD:AAB4472	Finland	University of Oulu

Ourapteryx sambucaria	MM01150	LEFIA172-10	HM396517	BOLD:AAB4472	Finland	University of Oulu
Ourapteryx sambucaria	MM01149	LEFIA171-10	HM396516	BOLD:AAB4472	Finland	University of Oulu
Ourapteryx sambucaria	TLMF Lep 08156	PHLAV337-12	KM572171	BOLD:AAB4472	Austria	inatura, Dornbirn
Ourapteryx sambucaria	MM09772	LEFIA895-10	HM387031	BOLD:AAB4472	Finland	University of Oulu
Pachetra sagittigera	TLMF Lep 07832	PHLAV013-12	KM572742	BOLD:AAC1169	Austria	inatura, Dornbirn
Pachetra sagittigera	MM18909	LEFIL611-10	KM573018	BOLD:ACF4123	Finland	University of Oulu
Pachetra sagittigera	MM15957	LEFIJ357-10	KM572927	BOLD:ACF4123	Finland	University of Oulu
Pammene fasciana	MM09774	LEFIA897-10	HM387033	BOLD:AAC8302	Finland	University of Oulu
Pammene fasciana	MM03827	LEFIC323-10	HM872167	BOLD:AAC8302	Finland	University of Oulu
Pammene fasciana	MM09773	LEFIA896-10	HM387032	BOLD:AAC8302	Finland	University of Oulu
Pammene fasciana	TLMF Lep 09773	LEATA356-13	KM572859	BOLD:AAC8302	Austria	Tiroler Landesmuseum Ferdinandeum
Pammene ignorata	MM18644	LEFIL334-10	JN274982	BOLD:AAF1865	Finland	University of Oulu
Pammene ignorata	MM05217	LEFIC903-10	HM872720	BOLD:AAF1865	Finland	University of Oulu
Pammene ignorata	MM18894	LEFIL596-10	KM573147	BOLD:AAF1865	Finland	University of Oulu
Pammene ignorata	MM04310	LEFIC551-10	HM872372	BOLD:AAF1865	Finland	University of Oulu
Pammene ignorata	MM09253	LEFIE478-10	HM874202	BOLD:AAF1865	Finland	University of Oulu
Pammene ignorata	MM11044	LEFIF262-10	HM874955	BOLD:AAF1865	Finland	University of Oulu
Pammene ignorata	MM17606	LEFIK031-10	KM572936	BOLD:AAF1865	Finland	University of Oulu
Pammene ignorata	TLMF Lep 09772	LEATA355-13	KM573053	BOLD:AAF1865	Austria	Tiroler Landesmuseum Ferdinandeum
Pammene ochsenheimeriana	TLMF Lep 09982	PHLAW185-13	KM573225	BOLD:AAF1961	Austria	inatura, Dornbirn
Pammene ochsenheimeriana	MM09844	LEFIE741-10	HM874461	BOLD:AAF1961	Finland	University of Oulu
Pammene ochsenheimeriana	MM18359	LEFIK784-10	JN274968	BOLD:AAF1961	Finland	University of Oulu
Pammene ochsenheimeriana	MM09843	LEFIE740-10	HM874460	BOLD:AAF1961	Finland	University of Oulu
Pandemis cerasana	MM02012	LEFIB466-10	HM871365	BOLD:AAA3660	Finland	University of Oulu
Pandemis cerasana	TLMF Lep 08018	PHLAV199-12	KM572953	BOLD:AAA3660	Austria	inatura, Dornbirn
Pandemis cerasana	MM13194	LEFIF845-10	HM875528	BOLD:AAA3660	Finland	University of Oulu
Pandemis cerasana	MM03256	LEFIC031-10	HM871901	BOLD:AAA3660	Finland	University of Oulu

Pandemis cinnamomeana	MM03365	LEFIC075-10	HM871944	BOLD:AAD0575	Finland	University of Oulu
Pandemis cinnamomeana	TLMF Lep 08017	PHLAV198-12	KM572427	BOLD:AAD0575	Austria	inatura, Dornbirn
Pandemis cinnamomeana	MM01076	LEFIA135-10	HM396482	BOLD:AAD0575	Finland	University of Oulu
Pandemis cinnamomeana	MM13198	LEFIF847-10	HM875530	BOLD:AAD0575	Finland	University of Oulu
Pandemis cinnamomeana	MM01075	LEFIA1275-10	GU828658	BOLD:AAD0575	Finland	University of Oulu
Pandemis dumetana	MM04989	LEFIC812-10	HM872631	BOLD:AAD2733	Finland	University of Oulu
Pandemis dumetana	MM03507	LEFIC161-10	HM872007	BOLD:AAD2733	Finland	University of Oulu
Pandemis dumetana	TLMF Lep 08419	PHLAH600-12	KM572398	BOLD:AAD2733	Austria	inatura, Dornbirn
Pandemis dumetana	MM03574	LEFIC206-10	HM872050	BOLD:AAD2733	Finland	University of Oulu
Panemeria tenebrata	MM00007	LEFIB004-10	HM870917	BOLD:AAD0566	Finland	University of Oulu
Panemeria tenebrata	MM09978	LEFIE794-10	HM874513	BOLD:AAD0566	Finland	University of Oulu
Panemeria tenebrata	TLMF Lep 09860	PHLAW063-13	KM573694	BOLD:AAD0566	Austria	Tiroler Landesmuseum Ferdinandeum
Panemeria tenebrata	MM00005	LEFIA1276-10	KM573638	BOLD:AAD0566	Finland	University of Oulu
Panemeria tenebrata	MM00006	LEFIB003-10	HM870916	BOLD:AAD0566	Finland	University of Oulu
Panolis flammea	MM01583	LEFIA501-10	KM572588	BOLD:AAC5406	Finland	University of Oulu
Panolis flammea	MM00397	LEFIB091-10	HM870998	BOLD:AAC5406	Finland	University of Oulu
Panolis flammea	MM01582	LEFIA500-10	KM572077	BOLD:AAC5406	Finland	University of Oulu
Panolis flammea	TLMF Lep 07881	PHLAV062-12	KM572810	BOLD:AAC5406	Austria	inatura, Dornbirn
Panthea coenobita	MM04583	LEFIA1277-10	GU828702	BOLD:AAE7030	Finland	University of Oulu
Panthea coenobita	MM18528	LEFII101-10	JF853341	BOLD:AAE7030	Finland	University of Oulu
Panthea coenobita	TLMF Lep 06190	PHLSA735-11	KM572146	BOLD:AAE7030	Austria	Tiroler Landesmuseum Ferdinandeum
Panthea coenobita	TLMF Lep 08126	PHLAV307-12	KM573468	BOLD:AAE7030	Austria	inatura, Dornbirn
Panthea coenobita	MM05913	LEFID150-10	HM872957	BOLD:AAE7030	Finland	University of Oulu
Papestra biren	MM14830	LEFIG616-10	HM876282	BOLD:AAA9849	Finland	University of Oulu
Papestra biren	MM04101	LEFIA747-10	HM386891	BOLD:AAA9849	Finland	University of Oulu
Papestra biren	MM12519	LEFIF629-10	HM875313	BOLD:AAA9849	Finland	University of Oulu
Papestra biren	TLMF Lep 04652	PHLAE337-11	JN272312	BOLD:AAA9849	Austria	Tiroler Landesmuseum Ferdinandeum
Papilio machaon	TLMF Lep 09113	PHLAI551-13	KM572218	BOLD:AAA5810	Austria	Tiroler Landesmuseum Ferdinandeum
Papilio machaon	MM05702	LEFID090-10	HM872901	BOLD:AAA5810	Finland	University of Oulu
Papilio machaon	MM00727	LEFIB255-10	HM871158	BOLD:AAA5810	Finland	University of Oulu
Papilio machaon	MM05450	LEFID021-10	HM872835	BOLD:AAA5810	Finland	University of Oulu

Parachronistis albiceps	MM09445	LEFIE559-10	HM874282	BOLD:AAD5736	Finland	University of Oulu
Parachronistis albiceps	MM06038	LEFID209-10	HM873011	BOLD:AAD5736	Finland	University of Oulu
Parachronistis albiceps	MM06876	LEFID800-10	HM873557	BOLD:AAD5736	Finland	University of Oulu
Parachronistis albiceps	MM06737	LEFID690-10	HM873451	BOLD:AAD5736	Finland	University of Oulu
Parachronistis albiceps	MM09735	LEFIA858-10	HM386996	BOLD:AAD5736	Finland	University of Oulu
Parachronistis albiceps	MM09224	LEFIE458-10	HM874182	BOLD:AAD5736	Finland	University of Oulu
Parachronistis albiceps	TLMF Lep 08258	PHLAH439-12	KM573174	BOLD:ACA9804	Austria	inatura, Dornbirn
Paradarisa consonaria	MM01294	LEFIA257-10	HM386601	BOLD:AAC3271	Finland	University of Oulu
Paradarisa consonaria	MM01515	LEFIA451-10	HM386792	BOLD:AAC3271	Finland	University of Oulu
Paradarisa consonaria	MM03807	LEFIC310-10	HM872154	BOLD:AAC3271	Finland	University of Oulu
Paradarisa consonaria	TLMF Lep 04660	PHLAE345-11	JN269342	BOLD:AAC3271	Austria	Tiroler Landesmuseum Ferdinandeum
Paradarisa consonaria	MM01292	LEFIA255-10	HM386599	BOLD:AAC3271	Finland	University of Oulu
Paranthrene tabaniformis	MM08396	LEFIE150-10	HM873898	BOLD:ACF0531	Finland	University of Oulu
Paranthrene tabaniformis	MM15756	LEFIG892-10	HM876537	BOLD:ACF0531	Finland	University of Oulu
Paranthrene tabaniformis	MM08395	LEFIA1278-10	GU828823	BOLD:ACF0531	Finland	University of Oulu
Paranthrene tabaniformis	TLMF Lep 09089	PHLAI527-13	KM573451	BOLD:ACF0532	Austria	Tiroler Landesmuseum Ferdinandeum
Parapoynx stratiotata	MM06872	LEFID796-10	HM873553	BOLD:AAC1045	Finland	University of Oulu
Parapoynx stratiotata	TLMF Lep 07533	PHLAG854-12	KM571954	BOLD:AAC1045	Austria	Tiroler Landesmuseum Ferdinandeum
Parapoynx stratiotata	MM03544	LEFIC186-10	HM872030	BOLD:AAC1045	Finland	University of Oulu
Parapoynx stratiotata	MM08813	LEFIE364-10	HM874088	BOLD:AAC1045	Finland	University of Oulu
Pararge aegeria	MM17185	LEFIJ560-10	JF853675	BOLD:AAA4515	Finland	University of Oulu
Pararge aegeria	MM18693	LEFIL395-10	KM572979	BOLD:AAA4515	Finland	University of Oulu
Pararge aegeria	MM17186	LEFIJ561-10	JF853676	BOLD:AAA4515	Finland	University of Oulu
Pararge aegeria	TLMF Lep 09822	PHLAW025-13	KM573345	BOLD:AAA4515	Austria	Tiroler Landesmuseum Ferdinandeum
Parasemia plantaginis	MM03197	LEFIB998-10	HM871869	BOLD:AAB6883	Finland	University of Oulu
Parasemia plantaginis	TLMF Lep 09845	PHLAW048-13	KM572445	BOLD:AAB6883	Austria	Tiroler Landesmuseum Ferdinandeum
Parasemia plantaginis	MM10075	LEFIE849-10	HM874568	BOLD:AAB6883	Finland	University of Oulu
Parasemia plantaginis	MM10138	LEFIE870-10	HM874588	BOLD:AAB6883	Finland	University of Oulu
Parastichtis suspecta	MM01717	LEFIA605-10	HM870854	BOLD:AAB4551	Finland	University of Oulu
Parastichtis suspecta	MM02764	LEFIB824-10	HM871701	BOLD:AAB4551	Finland	University of Oulu
Parastichtis suspecta	TLMF Lep 10026	LEATA419-13	KM572404	BOLD:AAB4551	Austria	inatura, Dornbirn

Parastichtis suspecta	MM01716	LEFIA604-10	HM870853	BOLD:AAB4551	Finland	University of Oulu
Paratalanta hyalinalis	MM02377	LEFIB629-10	HM871508	BOLD:AAF7132	Finland	University of Oulu
Paratalanta hyalinalis	MM17845	LEFIK270-10	KM572044	BOLD:AAF7132	Finland	University of Oulu
Paratalanta hyalinalis	TLMF Lep 08178	PHLAV359-12	KM572045	BOLD:AAF7132	Austria	inatura, Dornbirn
Paratalanta hyalinalis	MM11134	LEFIF329-10	HM875014	BOLD:AAF7132	Finland	University of Oulu
Paratalanta pandalis	MM01853	LEFIA699-10	HM386844	BOLD:AAE7251	Finland	University of Oulu
Paratalanta pandalis	TLMF Lep 07892	PHLAV073-12	KM572848	BOLD:AAE7251	Austria	inatura, Dornbirn
Paratalanta pandalis	MM04330	LEFIC556-10	HM872377	BOLD:AAE7251	Finland	University of Oulu
Paratalanta pandalis	MM01852	LEFIA698-10	HM386843	BOLD:AAE7251	Finland	University of Oulu
Parectopa ononidis	TLMF Lep 09176	PHLAI614-13	KM572303	BOLD:AAE3311	Austria	Tiroler Landesmuseum Ferdinandeum
Parectopa ononidis	MM14361	LEFIG362-10	HM876039	BOLD:AAE3311	Finland	University of Oulu
Parectopa ononidis	MM14470	LEFIG434-10	HM876110	BOLD:AAE3311	Finland	University of Oulu
Parectopa ononidis	MM03209	LEFIC008-10	HM871878	BOLD:AAE3311	Finland	University of Oulu
Parnassius mnemosyne	MM17420	LEFIJ795-10	JF853829	BOLD:AAA3342	Finland	University of Oulu
Parnassius mnemosyne	MM17419	LEFIJ794-10	JF853828	BOLD:AAA3342	Finland	University of Oulu
Parnassius mnemosyne	TLMF Lep 06150	PHLSA695-11	KM573649	BOLD:AAA3343	Austria	Tiroler Landesmuseum Ferdinandeum
Parornix betulae	TLMF Lep 09967	PHLAW170-13	KM572253	BOLD:AAE3418	Austria	inatura, Dornbirn
Parornix betulae	MM17974	LEFIK399-10	JF854031	BOLD:AAE3418	Finland	University of Oulu
Parornix betulae	MM06299	LEFID387-10	HM873184	BOLD:AAE3418	Finland	University of Oulu
Parornix betulae	MM06186	LEFID306-10	HM873103	BOLD:ABZ4246	Finland	University of Oulu
Parornix betulae	MM03970	LEFIC424-10	HM872264	BOLD:ABZ4246	Finland	University of Oulu
Parornix betulae	MM02603	LEFIB754-10	HQ963158	BOLD:ABZ4246	Finland	University of Oulu
Parornix devoniella	MM07010	LEFID874-10	HM873631	BOLD:AAD1352	Finland	University of Oulu
Parornix devoniella	TLMF Lep 07495	PHLAG816-12	KM572415	BOLD:AAD1352	Austria	inatura, Dornbirn
Parornix devoniella	TLMF Lep 07947	PHLAV128-12	KM573187	BOLD:AAD1352	Austria	inatura, Dornbirn
Parornix devoniella	MM09610	LEFIE650-10	HM874373	BOLD:AAD1352	Finland	University of Oulu
Pasiphila debiliata	MM01829	LEFIA688-10	HQ963156	BOLD:AAE7106	Finland	University of Oulu
Pasiphila debiliata	MM01828	LEFIA687-10	HM386833	BOLD:AAE7106	Finland	University of Oulu
Pasiphila debiliata	MM05184	LEFIC887-10	HM872705	BOLD:AAE7106	Finland	University of Oulu
Pasiphila debiliata	TLMF Lep 10005	LEATA398-13	KM572735	BOLD:AAE7106	Austria	inatura, Dornbirn
Pasiphila debiliata	MM00715	LEFIB247-10	HM871150	BOLD:AAE7106	Finland	University of Oulu

Pasiphila rectangulata	TLMF Lep 08159	PHLAV340-12	KM573624	BOLD:AAA3075	Austria	inatura, Dornbirn
Pasiphila rectangulata	MM01825	LEFIA685-10	HM386831	BOLD:AAA3075	Finland	University of Oulu
Pasiphila rectangulata	MM12977	LEFIF765-10	HM875449	BOLD:AAA3075	Finland	University of Oulu
Pasiphila rectangulata	MM07981	LEFID973-10	HM873723	BOLD:AAA3075	Finland	University of Oulu
Pasiphila rectangulata	MM01826	LEFIA686-10	HM386832	BOLD:AAA3075	Finland	University of Oulu
Pasiphila rectangulata	MM00712	LEFIB245-10	HM871148	BOLD:AAA3075	Finland	University of Oulu
Pelochrista caecimaculana	MM18642	LEFIL332-10	KM573580	BOLD:AAE7175	Finland	University of Oulu
Pelochrista caecimaculana	MM18336	LEFIK761-10	JN274951	BOLD:AAE7175	Finland	University of Oulu
Pelochrista caecimaculana	MM05917	LEFID154-10	HM872961	BOLD:AAE7175	Finland	University of Oulu
Pelochrista caecimaculana	TLMF Lep 07509	PHLAG830-12	KM572635	BOLD:AAE7175	Austria	Tiroler Landesmuseum Ferdinandeum
Pennisetia hylaeiformis	MM06964	LEFID842-10	HM873599	BOLD:AAD3203	Finland	University of Oulu
Pennisetia hylaeiformis	MM05827	LEFIA1286-10	KM573189	BOLD:AAD3203	Finland	University of Oulu
Pennisetia hylaeiformis	MM06963	LEFIA1285-10	GU828753	BOLD:AAD3203	Finland	University of Oulu
Pennisetia hylaeiformis	MM06965	LEFID843-10	HM873600	BOLD:AAD3203	Finland	University of Oulu
Pennisetia hylaeiformis	TLMF Lep 09088	PHLAI526-13	KM573459	BOLD:AAD3203	Austria	Tiroler Landesmuseum Ferdinandeum
Pennithera firmata	MM15807	LEFIG943-10	HM876583	BOLD:AAC6743	Finland	University of Oulu
Pennithera firmata	MM04805	LEFIC729-10	HM872550	BOLD:AAC6743	Finland	University of Oulu
Pennithera firmata	MM12819	LEFIJ200-10	KM572387	BOLD:AAC6743	Finland	University of Oulu
Pennithera firmata	TLMF Lep 08765	PHLAI270-13	KM572301	BOLD:AAC6743	Austria	Tiroler Landesmuseum Ferdinandeum
Pennithera firmata	MM15808	LEFIG944-10	HM876584	BOLD:AAC6743	Finland	University of Oulu
Peribatodes secundaria	MM06806	LEFID745-10	HM873502	BOLD:AAC2923	Finland	University of Oulu
Peribatodes secundaria	TLMF Lep 08551	PHLAH732-12	KM572575	BOLD:AAC2923	Austria	inatura, Dornbirn
Peribatodes secundaria	MM06807	LEFID746-10	HM873503	BOLD:AAC2923	Finland	University of Oulu
Peribatodes secundaria	MM09579	LEFIE635-10	HM874358	BOLD:AAC2923	Finland	University of Oulu
Perittia farinella	MM06229	LEFID335-10	HM873132	BOLD:AAL2425	Finland	University of Oulu
Perittia farinella	MM06230	LEFID336-10	HM873133	BOLD:AAL2425	Finland	University of Oulu
Perittia farinella	MM08383	LEFIE147-10	HM873895	BOLD:AAL2425	Finland	University of Oulu
Perittia farinella	TLMF Lep 07468	PHLAG789-12	KM573043	BOLD:AAL2425	Austria	inatura, Dornbirn
Perizoma affinitata	MM18469	LEFIK894-10	JF854345	BOLD:AAC0743	Finland	University of Oulu
Perizoma affinitata	MM18470	LEFIK895-10	KM573401	BOLD:AAC0743	Finland	University of Oulu
Perizoma affinitata	MM01343	LEFIA298-10	HM386641	BOLD:AAC0743	Finland	University of Oulu

Perizoma affinitata	MM10117	LEFIE865-10	HM874583	BOLD:AAC0743	Finland	University of Oulu
Perizoma affinitata	MM00094	LEFIA023-10	HM396373	BOLD:AAC0743	Finland	University of Oulu
Perizoma affinitata	MM01344	LEFIA299-10	HM386642	BOLD:AAC0743	Finland	University of Oulu
Perizoma affinitata	TLMF Lep 04635	PHLAE320-11	JN279567	BOLD:AAC0743	Austria	Tiroler Landesmuseum Ferdinandeum
Perizoma alchemillata	MM03610	LEFIC218-10	HM872062	BOLD:AAA9313	Finland	University of Oulu
Perizoma alchemillata	TLMF Lep 08800	PHLAI305-13	KM572750	BOLD:AAA9313	Austria	Tiroler Landesmuseum Ferdinandeum
Perizoma alchemillata	MM01454	LEFIA395-10	HM386737	BOLD:AAA9313	Finland	University of Oulu
Perizoma alchemillata	MM00719	LEFIB250-10	HM871153	BOLD:AAA9313	Finland	University of Oulu
Perizoma blandiata	MM01437	LEFIA380-10	HM386722	BOLD:AAC6889	Finland	University of Oulu
Perizoma blandiata	MM01509	LEFIA445-10	HM386786	BOLD:AAC6889	Finland	University of Oulu
Perizoma blandiata	MM07900	LEFID956-10	HM873706	BOLD:AAC6889	Finland	University of Oulu
Perizoma blandiata	MM00335	LEFIB052-10	HM870961	BOLD:AAC6889	Finland	University of Oulu
Perizoma blandiata	MM09825	LEFIE729-10	HM874449	BOLD:AAC6889	Finland	University of Oulu
Perizoma blandiata	TLMF Lep 07569	PHLAG890-12	KM573389	BOLD:AAC6889	Austria	inatura, Dornbirn
Perizoma hydrata	MM11666	LEFIF387-10	HM875072	BOLD:AAC0743	Finland	University of Oulu
Perizoma hydrata	TLMF Lep 08496	PHLAH677-12	KM572395	BOLD:AAC0743	Austria	inatura, Dornbirn
Perizoma hydrata	MM12844	LEFIF731-10	HM875415	BOLD:AAC0743	Finland	University of Oulu
Perizoma hydrata	MM04686	LEFIC698-10	HM872519	BOLD:AAC0743	Finland	University of Oulu
Perizoma hydrata	MM18468	LEFIK893-10	JF854344	BOLD:AAC0743	Finland	University of Oulu
Perizoma hydrata	MM18467	LEFIK892-10	JF854343	BOLD:AAC0743	Finland	University of Oulu
Perizoma minorata	MM04107	LEFIA753-10	HM386897	BOLD:AAD4210	Finland	University of Oulu
Perizoma minorata	MM06363	LEFID433-10	HQ570329	BOLD:AAD4210	Finland	University of Oulu
Perizoma minorata	MM00090	LEFIA019-10	HM396369	BOLD:AAD4210	Finland	University of Oulu
Perizoma minorata	TLMF Lep 00315	PHLAA275-09	HM381356	BOLD:AAD4210	Austria	Tiroler Landesmuseum Ferdinandeum
Petrophora chlorosata	MM04506	LEFIC588-10	HM872409	BOLD:AAC0420	Finland	University of Oulu
Petrophora chlorosata	TLMF Lep 07973	PHLAV154-12	KM572736	BOLD:AAC0420	Austria	inatura, Dornbirn
Petrophora chlorosata	MM17371	LEFIJ746-10	KM573227	BOLD:AAC0420	Finland	University of Oulu
Petrophora chlorosata	MM05420	LEFIC999-10	HM872814	BOLD:AAC0420	Finland	University of Oulu
Pexicopia malvella	MM03491	LEFIC146-10	HM871992	BOLD:AAD9025	Finland	University of Oulu
Pexicopia malvella	TLMF Lep 07519	PHLAG840-12	KM572818	BOLD:AAD9025	Austria	Tiroler Landesmuseum Ferdinandeum
Pexicopia malvella	MM06850	LEFID781-10	HM873538	BOLD:AAD9025	Finland	University of Oulu

Pexicopia malvella	TLMF Lep 08234	PHLAH415-12	KM572846	BOLD:AAD9025	Austria	inatura, Dornbirn
Pexicopia malvella	TLMF Lep 09914	PHLAW117-13	KM573618	BOLD:AAD9025	Austria	Tiroler Landesmuseum Ferdinandeum
Pexicopia malvella	MM03479	LEFIA1289-10	GU828692	BOLD:AAD9025	Finland	University of Oulu
Pexicopia malvella	MM12429	LEFIF589-10	HM875273	BOLD:AAD9025	Finland	University of Oulu
Phalera bucephala	TLMF Lep 07826	PHLAV007-12	KM572982	BOLD:AAC0945	Austria	inatura, Dornbirn
Phalera bucephala	MM00982	LEFIA1292-10	GU828652	BOLD:AAC0945	Finland	University of Oulu
Phalera bucephala	MM00122	LEFIA1291-10	GU828607	BOLD:AAC0945	Finland	University of Oulu
Phalera bucephala	MM09382	LEFIE529-10	HM874252	BOLD:AAC0945	Finland	University of Oulu
Phalera bucephala	MM00983	LEFIA068-10	HM396415	BOLD:AAC0945	Finland	University of Oulu
Phalonidia gilvicomana	MM19244	LEFIL244-10	JQ775265	BOLD:AAQ3489	Finland	University of Oulu
Phalonidia gilvicomana	MM19245	LEFIL245-10	JF854549	BOLD:AAQ3489	Finland	University of Oulu
Phalonidia gilvicomana	TLMF Lep 08226	PHLAH407-12	KM572451	BOLD:AAQ3489	Austria	inatura, Dornbirn
Pharmacis fusconebulosa	MM07992	LEFID975-10	HM873725	BOLD:AAF2520	Finland	University of Oulu
Pharmacis fusconebulosa	TLMF Lep 03062	PHLAD077-11	KM573405	BOLD:AAF2520	Austria	Tiroler Landesmuseum Ferdinandeum
Pharmacis fusconebulosa	MM01847	LEFIA1163-10	GU828674	BOLD:AAF2520	Finland	University of Oulu
Pharmacis fusconebulosa	MM03163	LEFIB973-10	HM871846	BOLD:AAF2520	Finland	University of Oulu
Pharmacis fusconebulosa	MM05223	LEFIC905-10	HM872722	BOLD:AAF2520	Finland	University of Oulu
Pharmacis fusconebulosa	MM01846	LEFIA1162-10	GU828673	BOLD:AAF2520	Finland	University of Oulu
Pharmacis lupulina	TLMF Lep 04412	PHLAE192-11	JN307412	BOLD:AAA8451	Austria	Tiroler Landesmuseum Ferdinandeum
Pharmacis lupulina	MM19180	LEFIL180-10	KM572970	BOLD:AAA8451	Finland	University of Oulu
Pharmacis lupulina	MM19179	LEFIL179-10	KM572697	BOLD:AAA8451	Finland	University of Oulu
Phaulernis fulviguttella	MM18904	LEFIL606-10	KM573007	BOLD:AAI4589	Finland	University of Oulu
Phaulernis fulviguttella	MM11049	LEFIF267-10	HM874960	BOLD:AAI4589	Finland	University of Oulu
Phaulernis fulviguttella	MM14645	LEFIG526-10	HM876199	BOLD:AAI4589	Finland	University of Oulu
Phaulernis fulviguttella	MM15751	LEFIG887-10	HM876532	BOLD:AAI4589	Finland	University of Oulu
Phaulernis fulviguttella	TLMF Lep 04306	PHLAE086-11	JN269622	BOLD:AAI4589	Austria	Tiroler Landesmuseum Ferdinandeum
Pheosia gnoma	MM09913	LEFIE767-10	HM874486	BOLD:AAC5101	Finland	University of Oulu
Pheosia gnoma	MM00996	LEFIA078-10	HM396425	BOLD:AAC5101	Finland	University of Oulu
Pheosia gnoma	MM00997	LEFIA079-10	HM396426	BOLD:AAC5101	Finland	University of Oulu
Pheosia gnoma	MM05106	LEFIC860-10	HM872678	BOLD:AAC5101	Finland	University of Oulu
Pheosia gnoma	TLMF Lep 08485	PHLAH666-12	KM572097	BOLD:AAC5101	Austria	inatura, Dornbirn

Pheosia tremula	MM00994	LEFIA076-10	HM396423	BOLD:AAB5913	Finland	University of Oulu
Pheosia tremula	MM08333	LEFIE125-10	HM873873	BOLD:AAB5913	Finland	University of Oulu
Pheosia tremula	MM00995	LEFIA077-10	HM396424	BOLD:AAB5913	Finland	University of Oulu
Pheosia tremula	TLMF Lep 08790	PHLAI295-13	KM572123	BOLD:AAB5913	Austria	Tiroler Landesmuseum Ferdinandeum
Phiaris bipunctana	MM18286	LEFIK711-10	JF854274	BOLD:AAA6005	Finland	University of Oulu
Phiaris bipunctana	TLMF Lep 09936	PHLAW139-13	KM573223	BOLD:AAA6005	Austria	inatura, Dornbirn
Phiaris bipunctana	MM03005	LEFIB902-10	HM871779	BOLD:AAA6005	Finland	University of Oulu
Phiaris bipunctana	MM08494	LEFIE185-10	HM873930	BOLD:AAA6005	Finland	University of Oulu
Phiaris bipunctana	MM08291	LEFIE106-10	HM873854	BOLD:AAA6005	Finland	University of Oulu
Phiaris dissolutana	TLMF Lep 10041	LEATA434-13	KM573032	BOLD:ABZ7390	Austria	inatura, Dornbirn
Phiaris dissolutana	MM03007	LEFIB903-10	HM871780	BOLD:ABZ7390	Finland	University of Oulu
Phiaris dissolutana	MM18284	LEFIK709-10	JF854273	BOLD:ABZ7390	Finland	University of Oulu
Phiaris dissolutana	MM03008	LEFIB904-10	HM871781	BOLD:ABZ7390	Finland	University of Oulu
Phiaris micana	MM03367	LEFIC076-10	HM871945	BOLD:ACE6219	Finland	University of Oulu
Phiaris micana	TLMF Lep 10029	LEATA422-13	KM572275	BOLD:ACE6219	Austria	inatura, Dornbirn
Phiaris micana	MM18288	LEFIK713-10	JF854276	BOLD:ACE6219	Finland	University of Oulu
Phiaris micana	TLMF Lep 09940	PHLAW143-13	KM573445	BOLD:ACE6219	Austria	inatura, Dornbirn
Phiaris micana	MM03330	LEFIC054-10	HM871923	BOLD:ACE6219	Finland	University of Oulu
Phiaris palustrana	MM03286	LEFIC045-10	HM871915	BOLD:AAC8201	Finland	University of Oulu
Phiaris palustrana	MM13317	LEFIF903-10	HM875585	BOLD:AAC8201	Finland	University of Oulu
Phiaris palustrana	MM02052	LEFIB485-10	HM871380	BOLD:AAC8201	Finland	University of Oulu
Phiaris palustrana	TLMF Lep 02835	PHLAC800-10	JF860317	BOLD:AAC8201	Austria	Tiroler Landesmuseum Ferdinandeum
Phiaris schulziana	TLMF Lep 09147	PHLAI585-13	KM573067	BOLD:ACF5701	Austria	Tiroler Landesmuseum Ferdinandeum
Phiaris schulziana	MM04120	LEFIA766-10	HM386909	BOLD:ACF5701	Finland	University of Oulu
Phiaris schulziana	MM04118	LEFIA764-10	HM386907	BOLD:ACF5701	Finland	University of Oulu
Phiaris schulziana	MM03787	LEFIC303-10	HM872147	BOLD:ACF5701	Finland	University of Oulu
Phiaris schulziana	MM06203	LEFID318-10	HM873115	BOLD:ACF5701	Finland	University of Oulu
Phiaris schulziana	MM06286	LEFID375-10	HM873172	BOLD:ACF5701	Finland	University of Oulu
Phiaris schulziana	MM06311	LEFID396-10	HM873193	BOLD:ACF5701	Finland	University of Oulu
Phiaris schulziana	MM06351	LEFID423-10	HM873219	BOLD:ACF5701	Finland	University of Oulu
Phiaris schulziana	MM18289	LEFIK714-10	JF854277	BOLD:ACF5701	Finland	University of Oulu

Phiaris schulziana	MM18290	LEFIK715-10	JF854278	BOLD:ACF5701	Finland	University of Oulu
Phiaris schulziana	MM18291	LEFIK716-10	JF854279	BOLD:ACF5701	Finland	University of Oulu
Phiaris schulziana	MM08474	LEFIJ121-10	JX034686	BOLD:ACF5701	Finland	University of Oulu
Phiaris schulziana	MM08475	LEFIJ122-10	JX034661	BOLD:ACF5701	Finland	University of Oulu
Phiaris schulziana	MM14581	LEFIJ250-10	JX034593	BOLD:ACF5701	Finland	University of Oulu
Phiaris schulziana	MM14582	LEFIJ251-10	JX034636	BOLD:ACF5701	Finland	University of Oulu
Phiaris schulziana	MM04119	LEFIA765-10	HM386908	BOLD:ACF5701	Finland	University of Oulu
Phiaris schulziana	MM21043	LEFIJ1183-11	KM573009	BOLD:ACF5701	Finland	University of Oulu
Phigalia pilosaria	MM07736	LEFID941-10	HM873691	BOLD:AAD0877	Finland	University of Oulu
Phigalia pilosaria	MM04605	LEFIC653-10	HM872474	BOLD:AAD0877	Finland	University of Oulu
Phigalia pilosaria	TLMF Lep 09775	LEATA358-13	KM573573	BOLD:AAD0877	Austria	Tiroler Landesmuseum Ferdinandeum
Phigalia pilosaria	MM01482	LEFIA420-10	HM386762	BOLD:AAD0877	Finland	University of Oulu
Philereme transversata	MM18449	LEFIK874-10	KM572753	BOLD:AAC5104	Finland	University of Oulu
Philereme transversata	TLMF Lep 08193	PHLAV374-12	KM572187	BOLD:AAC5104	Austria	inatura, Dornbirn
Philereme transversata	MM10411	LEFIE978-10	HM874695	BOLD:AAC5104	Finland	University of Oulu
Philereme vetulata	TLMF Lep 08860	PHLAI365-13	KM572975	BOLD:AAD2984	Austria	Tiroler Landesmuseum Ferdinandeum
Philereme vetulata	MM09781	LEFIA903-10	HM387039	BOLD:AAD2984	Finland	University of Oulu
Philereme vetulata	MM09782	LEFIA904-10	HM387040	BOLD:AAD2984	Finland	University of Oulu
Philereme vetulata	MM18448	LEFIK873-10	KM572560	BOLD:AAD2984	Finland	University of Oulu
Phlogophora meticulosa	TLMF Lep 08806	PHLAI311-13	KM571991	BOLD:AAB7358	Austria	Tiroler Landesmuseum Ferdinandeum
Phlogophora meticulosa	MM17288	LEFIJ663-10	KM572186	BOLD:AAB7358	Finland	University of Oulu
Phlogophora meticulosa	MM14436	LEFIG410-10	HM876086	BOLD:AAB7358	Finland	University of Oulu
Phlogophora meticulosa	MM14737	LEFIG578-10	HM876249	BOLD:AAB7358	Finland	University of Oulu
Photedes fluxa	MM04907	LEFIC776-10	HM872595	BOLD:AAD8063	Finland	University of Oulu
Photedes fluxa	TLMF Lep 04454	PHLAE234-11	JN287084	BOLD:AAD8063	Austria	Tiroler Landesmuseum Ferdinandeum
Photedes fluxa	MM01626	LEFIA539-10	KM572669	BOLD:AAD8063	Finland	University of Oulu
Photedes fluxa	MM01627	LEFIA540-10	KM573434	BOLD:AAD8063	Finland	University of Oulu
Phragmataecia castaneae	MM00170	LEFIA1297-10	GU828623	BOLD:AAE6494	Finland	University of Oulu
Phragmataecia castaneae	MM11006	LEFIF226-10	HM874919	BOLD:AAE6494	Finland	University of Oulu
Phragmataecia castaneae	TLMF Lep 05624	PHLAF454-11	KM573673	BOLD:AAE6494	Austria	Tiroler Landesmuseum Ferdinandeum
Phragmatobia fuliginosa	MM01042	LEFIA117-10	HM396464	BOLD:AAA6178	Finland	University of Oulu

Phragmatobia fuliginosa	MM04920	LEFIC784-10	HM872603	BOLD:AAA6178	Finland	University of Oulu
Phragmatobia fuliginosa	MM01041	LEFIA116-10	HM396463	BOLD:AAA6178	Finland	University of Oulu
Phragmatobia fuliginosa	TLMF Lep 07848	PHLAV029-12	KM573485	BOLD:AAA6178	Austria	inatura, Dornbirn
Phragmatobia fuliginosa	MM06360	LEFID430-10	HQ570326	BOLD:AAA6178	Finland	University of Oulu
Phragmatobia fuliginosa	MM00672	LEFIB218-10	HM871122	BOLD:AAA6178	Finland	University of Oulu
Phtheochroa inopiana	MM02117	LEFIB517-10	HQ570268	BOLD:ABZ3448	Finland	University of Oulu
Phtheochroa inopiana	MM02118	LEFIB518-10	HQ570269	BOLD:ABZ3448	Finland	University of Oulu
Phtheochroa inopiana	MM15670	LEFIG806-10	HM876458	BOLD:ABZ3448	Finland	University of Oulu
Phtheochroa inopiana	TLMF Lep 08084	PHLAV265-12	KM573560	BOLD:ABZ3448	Austria	inatura, Dornbirn
Phycita roborella	MM05197	LEFIC892-10	HM872709	BOLD:AAB8710	Finland	University of Oulu
Phycita roborella	TLMF Lep 08002	PHLAV183-12	KM572399	BOLD:AAB8710	Austria	inatura, Dornbirn
Phycita roborella	MM06768	LEFID714-10	HM873475	BOLD:AAB8710	Finland	University of Oulu
Phycita roborella	MM04952	LEFIC797-10	HM872616	BOLD:AAB8710	Finland	University of Oulu
Phycita roborella	MM09785	LEFIA907-10	HM387043	BOLD:AAB8710	Finland	University of Oulu
Phycitodes binaevella	TLMF Lep 09180	PHLAI618-13	KM572688	BOLD:AAD4425	Austria	Tiroler Landesmuseum Ferdinandeum
Phycitodes binaevella	MM02437	LEFIB660-10	HM871538	BOLD:AAD4425	Finland	University of Oulu
Phycitodes binaevella	MM18387	LEFIK812-10	JN275981	BOLD:AAD4425	Finland	University of Oulu
Phycitodes binaevella	MM03552	LEFIC191-10	HM872035	BOLD:AAD4425	Finland	University of Oulu
Phycitodes saxicola	MM03635	LEFIC235-10	HM872079	BOLD:AAD9531	Finland	University of Oulu
Phycitodes saxicola	MM03634	LEFIC234-10	HM872078	BOLD:AAD9531	Finland	University of Oulu
Phycitodes saxicola	MM03633	LEFIC233-10	HM872077	BOLD:AAD9531	Finland	University of Oulu
Phycitodes saxicola	TLMF Lep 09173	PHLAI611-13	KM573369	BOLD:AAD9531	Austria	Tiroler Landesmuseum Ferdinandeum
Phyllonorycter cavella	TLMF Lep 09964	PHLAW167-13	KM573212	BOLD:AAF6253	Austria	inatura, Dornbirn
Phyllonorycter cavella	MM00555	LEFIB164-10	HM871068	BOLD:AAF6253	Finland	University of Oulu
Phyllonorycter cavella	MM06191	LEFID310-10	HM873107	BOLD:AAF6253	Finland	University of Oulu
Phyllonorycter cavella	MM00554	LEFIB163-10	HM871067	BOLD:AAF6253	Finland	University of Oulu
Phyllonorycter harrisella	MM11158	LEFIF342-10	HM875027	BOLD:AAF6926	Finland	University of Oulu
Phyllonorycter harrisella	TLMF Lep 08481	PHLAH662-12	KM572386	BOLD:AAF6926	Austria	inatura, Dornbirn
Phyllonorycter harrisella	MM11157	LEFIF341-10	HM875026	BOLD:AAF6926	Finland	University of Oulu
Phyllonorycter harrisella	MM11137	LEFIF331-10	HM875016	BOLD:AAF6926	Finland	University of Oulu

Phyllonorycter maestingella	MM22762	LEFIJ1494-12	KM571973	BOLD:AAL6962	Finland	University of Oulu
Phyllonorycter maestingella	MM20748	LEFIJ1370-12	KM573266	BOLD:AAL6962	Finland	University of Oulu
Phyllonorycter maestingella	MM20747	LEFIJ1369-12	KM573359	BOLD:AAL6962	Finland	University of Oulu
Phyllonorycter maestingella	TLMF Lep 07953	PHLAV134-12	KM572263	BOLD:AAL6962	Austria	inatura, Dornbirn
Phyllonorycter strigulatella	BIOUG04118-A03	GMFID723-12	KM573427	BOLD:AAD5287	Finland	Biodiversity Institute of Ontario
Phyllonorycter strigulatella	MM00547	LEFIB157-10	HM871062	BOLD:AAD5287	Finland	University of Oulu
Phyllonorycter strigulatella	BIOUG04118-C10	GMFIM248-13	KM572679	BOLD:AAD5287	Finland	Biodiversity Institute of Ontario
Phyllonorycter strigulatella	TLMF Lep 07992	PHLAV173-12	KM572686	BOLD:AAD5287	Austria	inatura, Dornbirn
Phyllonorycter strigulatella	MM02636	LEFIB767-10	HM871644	BOLD:AAD5287	Finland	University of Oulu
Phyllonorycter strigulatella	MM09319	LEFIE501-10	HM874225	BOLD:AAD5287	Finland	University of Oulu
Phymatopus hecta	TLMF Lep 00743	PHLAA703-09	HM426100	BOLD:AAD8916	Austria	Tiroler Landesmuseum Ferdinandeum
Phymatopus hecta	MM14454	LEFIG423-10	HM876099	BOLD:AAD8916	Finland	University of Oulu
Phymatopus hecta	MM01848	LEFIA696-10	HM386841	BOLD:AAD8916	Finland	University of Oulu
Phymatopus hecta	MM03332	LEFIC055-10	HM871924	BOLD:AAD8916	Finland	University of Oulu
Phytometra viridaria	MM18719	LEFIL421-10	JF854591	BOLD:AAD4078	Finland	University of Oulu
Phytometra viridaria	TLMF Lep 00300	PHLAA260-09	HM425797	BOLD:AAD4078	Austria	Tiroler Landesmuseum Ferdinandeum
Phytometra viridaria	MM18718	LEFIL420-10	JF854590	BOLD:AAD4078	Finland	University of Oulu
Phytometra viridaria	MM21078	LEFIJ1218-11	KM571983	BOLD:AAD4078	Finland	University of Oulu
Pieris brassicae	MM03697	LEFIC266-10	HM872110	BOLD:AAB0552	Finland	University of Oulu
Pieris brassicae	TLMF Lep 09808	PHLAW011-13	KM572942	BOLD:AAB0552	Austria	Tiroler Landesmuseum Ferdinandeum
Pieris brassicae	MM03696	LEFIC265-10	HM872109	BOLD:AAB0552	Finland	University of Oulu
Pieris brassicae	MM17127	LEFIJ502-10	JF853625	BOLD:AAB0552	Finland	University of Oulu

Pieris napi	MM00509	LEFIB138-10	HM871043	BOLD:AAA2226	Finland	University of Oulu
Pieris napi	MM17125	LEFIJ500-10	JF853624	BOLD:AAA2226	Finland	University of Oulu
Pieris napi	MM00514	LEFIB141-10	HM871046	BOLD:AAA2226	Finland	University of Oulu
Pieris napi	TLMF Lep 09807	PHLAW010-13	KM573117	BOLD:AAA2226	Austria	Tiroler Landesmuseum Ferdinandeum
Pieris rapae	MM06585	LEFIA1301-10	KM573502	BOLD:AAA2224	Finland	University of Oulu
Pieris rapae	TLMF Lep 10009	LEATA402-13	KM573655	BOLD:AAA2224	Austria	inatura, Dornbirn
Pieris rapae	MM17128	LEFIJ503-10	JF853626	BOLD:AAA2224	Finland	University of Oulu
Pieris rapae	MM17129	LEFIJ504-10	JF853627	BOLD:AAA2224	Finland	University of Oulu
Piniphila bifasciana	MM14295	LEFIG324-10	HM876003	BOLD:AAC5440	Finland	University of Oulu
Piniphila bifasciana	TLMF Lep 08069	PHLAV250-12	KM572269	BOLD:AAC5440	Austria	inatura, Dornbirn
Piniphila bifasciana	MM15701	LEFIG837-10	HM876485	BOLD:AAC5440	Finland	University of Oulu
Piniphila bifasciana	MM15700	LEFIG836-10	HM876484	BOLD:AAC5440	Finland	University of Oulu
Plagodis	BIOUG04490-E08	GMFIC394-13	KM571972	BOLD:AAA6014	Finland	Biodiversity Institute of Ontario
Plagodis	BIOUG04118-A05	GMFID725-12	KM572879	BOLD:AAA6014	Finland	Biodiversity Institute of Ontario
Plagodis dolabraria	MM01253	LEFIA222-10	HM396565	BOLD:AAB8023	Finland	University of Oulu
Plagodis dolabraria	TLMF Lep 04634	PHLAE319-11	JN269338	BOLD:AAB8023	Austria	Tiroler Landesmuseum Ferdinandeum
Plagodis dolabraria	MM01252	LEFIA221-10	HM396564	BOLD:AAB8023	Finland	University of Oulu
Plagodis dolabraria	MM04669	LEFIC689-10	HM872510	BOLD:AAB8023	Finland	University of Oulu
Plagodis pulveraria	MM01261	LEFIA229-10	HM396572	BOLD:AAA6014	Finland	University of Oulu
Plagodis pulveraria	MM01262	LEFIA230-10	HM396573	BOLD:AAA6014	Finland	University of Oulu
Plagodis pulveraria	MM00503	LEFIB134-10	HM871040	BOLD:AAA6014	Finland	University of Oulu
Plagodis pulveraria	TLMF Lep 04667	PHLAE352-11	JN269343	BOLD:AAA6014	Austria	Tiroler Landesmuseum Ferdinandeum
Platyptilia calodactyla	MM09921	LEFIE770-10	HM874489	BOLD:AAF4026	Finland	University of Oulu
Platyptilia calodactyla	MM21080	LEFIJ1220-11	KM572500	BOLD:AAF4026	Finland	University of Oulu
Platyptilia calodactyla	MM18373	LEFIK798-10	KM573226	BOLD:AAF4026	Finland	University of Oulu
Platyptilia calodactyla	TLMF Lep 04288	PHLAE068-11	JN277242	BOLD:AAF4026	Austria	Tiroler Landesmuseum Ferdinandeum
Platyptilia calodactyla	MM03100	LEFIJ053-10	KM572256	BOLD:AAF4026	Finland	University of Oulu
Platyptilia calodactyla	TLMF Lep 08411	PHLAH592-12	KM572904	BOLD:AAF4026	Austria	inatura, Dornbirn
Platyptilia calodactyla	MM21079	LEFIJ1219-11	KM573471	BOLD:AAF4026	Finland	University of Oulu
Platyptilia calodactyla	MM21081	LEFIJ1221-11	KM572645	BOLD:AAF4026	Finland	University of Oulu
Platyptilia calodactyla	MM18371	LEFIK796-10	JN277222	BOLD:AAF4026	Finland	University of Oulu

Platyptilia calodactyla	MM18372	LEFIK797-10	JN277223	BOLD:AAF4026	Finland	University of Oulu
Platyptilia calodactyla	MM03099	LEFIB944-10	HM871821	BOLD:AAF4027	Finland	University of Oulu
Platyptilia calodactyla	MM06373	LEFID440-10	HQ570336	BOLD:ABX5335	Finland	University of Oulu
Platyptilia gonodactyla	TLMF Lep 08410	PHLAH591-12	KM572727	BOLD:AAD4179	Austria	inatura, Dornbirn
Platyptilia gonodactyla	TLMF Lep 08441	PHLAH622-12	KM572082	BOLD:AAD4179	Austria	inatura, Dornbirn
Platyptilia gonodactyla	MM10204	LEFIE900-10	HQ570376	BOLD:AAD4179	Finland	University of Oulu
Platyptilia gonodactyla	MM01081	LEFIA137-10	HM396483	BOLD:AAD4179	Finland	University of Oulu
Platyptilia gonodactyla	MM18370	LEFIK795-10	JN277221	BOLD:AAD4179	Finland	University of Oulu
Platyptilia nemoralis	TLMF Lep 04508	PHLAE383-11	KM573074	BOLD:AAL4692	Austria	Tiroler Landesmuseum Ferdinandeum
Platyptilia nemoralis	MM06090	LEFID231-10	HM873031	BOLD:AAL4692	Finland	University of Oulu
Plebejus argus	MM09925	LEFIE771-10	HM874490	BOLD:ABZ1727	Finland	University of Oulu
Plebejus argus	MM03336	LEFIC056-10	HM871925	BOLD:ABZ1727	Finland	University of Oulu
Plebejus argus	MM17141	LEFIJ516-10	JF853638	BOLD:ABZ1727	Finland	University of Oulu
Plebejus argus	TLMF Lep 09809	PHLAW012-13	KM573493	BOLD:ABZ1727	Austria	Tiroler Landesmuseum Ferdinandeum
Plebejus idas	MM03338	LEFIC058-10	HM871927	BOLD:AAA3628	Finland	University of Oulu
Plebejus idas	MM03337	LEFIC057-10	HM871926	BOLD:AAA3628	Finland	University of Oulu
Plebejus idas	MM03346	LEFIC062-10	HM871931	BOLD:AAA3628	Finland	University of Oulu
Plebejus idas	TLMF Lep 08515	PHLAH696-12	KM572599	BOLD:AAA3628	Austria	inatura, Dornbirn
Plemyria rubiginata	MM01382	LEFIA335-10	HM386678	BOLD:AAC1712	Finland	University of Oulu
Plemyria rubiginata	MM00801	LEFIB296-10	HM871197	BOLD:AAC1712	Finland	University of Oulu
Plemyria rubiginata	TLMF Lep 08110	PHLAV291-12	KM572318	BOLD:AAC1712	Austria	inatura, Dornbirn
Plemyria rubiginata	MM01383	LEFIA336-10	HM386679	BOLD:AAC1712	Finland	University of Oulu
Pleuroptya ruralis	MM10241	LEFIE906-10	HM874623	BOLD:AAB6255	Finland	University of Oulu
Pleuroptya ruralis	MM00325	LEFIA1304-10	GU828634	BOLD:AAB6255	Finland	University of Oulu
Pleuroptya ruralis	MM01850	LEFIA697-10	HM386842	BOLD:AAB6255	Finland	University of Oulu
Pleuroptya ruralis	CNCLEP00020424	LNEL228-06	KM573447	BOLD:AAB6255	Finland	Canadian National Collection of Insects, Arachnids and Nematodes
Pleuroptya ruralis	MM01160	LEFIA176-10	HM396521	BOLD:AAB6255	Finland	University of Oulu
Pleuroptya ruralis	MM01159	LEFIA175-10	HM396520	BOLD:AAB6255	Finland	University of Oulu
Pleuroptya ruralis	TLMF Lep 08177	PHLAV358-12	KM572078	BOLD:AAB6255	Austria	inatura, Dornbirn
Pleurota bicostella	MM00060	LEFIB022-10	HM870933	BOLD:AAD4880	Finland	University of Oulu

Pleurota bicostella	MM00059	LEFIA1305-10	JF818781	BOLD:AAD4880	Finland	University of Oulu
Pleurota bicostella	TLMF Lep 09927	PHLAW130-13	KM571948	BOLD:AAD4880	Austria	inatura, Dornbirn
Pleurota bicostella	MM00061	LEFIB023-10	HM870934	BOLD:AAD4880	Finland	University of Oulu
Plodia interpunctella	TLMF Lep 09900	PHLAW103-13	KM572075	BOLD:AAB2462	Austria	Tiroler Landesmuseum Ferdinandeum
Plodia interpunctella	MM17620	LEFIK045-10	JF853922	BOLD:AAB2462	Finland	University of Oulu
Plodia interpunctella	MM15774	LEFIG910-10	HM876554	BOLD:AAB2462	Finland	University of Oulu
Plutella xylostella	MM23149	LEFIJ1957-13	KF370883	BOLD:AAA1513	Finland	University of Oulu
Plutella xylostella	MM03119	LEFIB955-10	HQ570277	BOLD:AAA1513	Finland	University of Oulu
Plutella xylostella	TLMF Lep 08237	PHLAH418-12	KF370832	BOLD:AAA1513	Austria	inatura, Dornbirn
Plutella xylostella	MM22996	LEFIJ1709-13	KF370752	BOLD:AAA1513	Finland	University of Oulu
Plutella xylostella	MM23148	LEFIJ1956-13	KF370629	BOLD:AAA1513	Finland	University of Oulu
Plutella xylostella	MM23147	LEFIJ1955-13	KF370769	BOLD:AAA1513	Finland	University of Oulu
Plutella xylostella	MM22993	LEFIJ1706-13	KF370797	BOLD:AAA1513	Finland	University of Oulu
Plutella xylostella	MM22994	LEFIJ1707-13	KF370607	BOLD:AAA1513	Finland	University of Oulu
Plutella xylostella	MM22991	LEFIJ1704-13	KF370647	BOLD:AAA1513	Finland	University of Oulu
Plutella xylostella	MM22995	LEFIJ1708-13	KF370684	BOLD:AAA1513	Finland	University of Oulu
Plutella xylostella	MM00110	LEFIA1306-10	GU828601	BOLD:AAA1513	Finland	University of Oulu
Plutella xylostella	MM22992	LEFIJ1705-13	KF370722	BOLD:AAA1513	Finland	University of Oulu
Plutella xylostella	MM22997	LEFIJ1710-13	KF370758	BOLD:AAA1513	Finland	University of Oulu
Plutella xylostella	TLMF Lep 07967	PHLAV148-12	KF370790	BOLD:AAA1513	Austria	inatura, Dornbirn
Plutella xylostella	MM00043	LEFIA1307-10	KM573317	BOLD:AAA1513	Finland	University of Oulu
Plutella xylostella	MM08054	LEFIE003-10	HM873752	BOLD:AAA1513	Finland	University of Oulu
Plutella xylostella	MM02280	LEFIB587-10	HM871467	BOLD:AAA1513	Finland	University of Oulu
Poecilocampa populi	MM08339	LEFIE126-10	HM873874	BOLD:AAC8994	Finland	University of Oulu
Poecilocampa populi	MM02696	LEFIA1308-10	GU828686	BOLD:AAC8994	Finland	University of Oulu
Poecilocampa populi	TLMF Lep 06215	PHLSA760-11	KM572544	BOLD:AAC8994	Austria	Tiroler Landesmuseum Ferdinandeum
Poecilocampa populi	MM02715	LEFIB801-10	HM871678	BOLD:AAC8994	Finland	University of Oulu
Poecilocampa populi	MM08223	LEFIE074-10	HM873821	BOLD:AAC8994	Finland	University of Oulu
Polia bombycina	MM12540	LEFIF638-10	HM875322	BOLD:AAD1839	Finland	University of Oulu
Polia bombycina	TLMF Lep 09227	PHLAI664-13	KM573342	BOLD:AAD1839	Austria	Tiroler Landesmuseum Ferdinandeum
Polia bombycina	MM01750	LEFIA635-10	HM870884	BOLD:AAD1839	Finland	University of Oulu

Polia bombycina	MM12542	LEFIF639-10	HM875323	BOLD:AAD1839	Finland	University of Oulu
Polia bombycina	MM05120	LEFIC864-10	HM872682	BOLD:AAD1839	Finland	University of Oulu
Polia hepatica	MM17918	LEFIK343-10	KM573462	BOLD:AAE1370	Finland	University of Oulu
Polia hepatica	TLMF Lep 09999	LEATA392-13	KM572338	BOLD:AAE1370	Austria	inatura, Dornbirn
Polia hepatica	MM17917	LEFIK342-10	KM573713	BOLD:AAE1370	Finland	University of Oulu
Polia hepatica	MM17916	LEFIK341-10	KM572491	BOLD:AAE1370	Finland	University of Oulu
Polia nebulosa	MM12549	LEFIF642-10	HM875326	BOLD:AAE1369	Finland	University of Oulu
Polia nebulosa	MM10808	LEFIF197-10	HM874891	BOLD:AAE1369	Finland	University of Oulu
Polia nebulosa	MM05090	LEFIC856-10	HM872674	BOLD:AAE1369	Finland	University of Oulu
Polia nebulosa	MM01749	LEFIA634-10	HM870883	BOLD:AAE1369	Finland	University of Oulu
Polia nebulosa	TLMF Lep 08106	PHLAV287-12	KM573503	BOLD:AAE1369	Austria	inatura, Dornbirn
Polia nebulosa	TLMF Lep 08174	PHLAV355-12	KM572522	BOLD:AAE1369	Austria	inatura, Dornbirn
Polia nebulosa	TLMF Lep 08173	PHLAV354-12	KM572948	BOLD:AAE1369	Austria	inatura, Dornbirn
Polychrysia moneta	MM01123	LEFIA154-10	HM396500	BOLD:AAD3729	Finland	University of Oulu
Polychrysia moneta	MM01166	LEFIA180-10	HM396525	BOLD:AAD3729	Finland	University of Oulu
Polychrysia moneta	TLMF Lep 04615	PHLAE300-11	JN276674	BOLD:AAD3729	Austria	Tiroler Landesmuseum Ferdinandeum
Polychrysia moneta	MM01122	LEFIA153-10	HM396499	BOLD:AAD3729	Finland	University of Oulu
Polygonia c-album	MM00384	LEFIB082-10	HM870991	BOLD:ABY7043	Finland	University of Oulu
Polygonia c-album	MM00385	LEFIB083-10	HM870992	BOLD:ABY7043	Finland	University of Oulu
Polygonia c-album	TLMF Lep 09812	PHLAW015-13	KM572201	BOLD:ABY7043	Austria	Tiroler Landesmuseum Ferdinandeum
Polygonia c-album	MM17161	LEFIJ536-10	JF853653	BOLD:ABY7043	Finland	University of Oulu
Polyommatus icarus	MM10352	LEFIE954-10	HM874671	BOLD:AAA3303	Finland	University of Oulu
Polyommatus icarus	TLMF Lep 08273	PHLAH454-12	KM573399	BOLD:AAA3303	Austria	inatura, Dornbirn
Polyommatus icarus	MM17147	LEFIJ522-10	JF853641	BOLD:AAA3303	Finland	University of Oulu
Polyommatus icarus	MM17146	LEFIJ521-10	KM572032	BOLD:AAA3303	Finland	University of Oulu
Prays fraxinella	MM09803	LEFIE714-10	HM874434	BOLD:AAB3722	Finland	University of Oulu
Prays fraxinella	MM11965	LEFIF461-10	HM875146	BOLD:AAB3722	Finland	University of Oulu
Prays fraxinella	MM00130	LEFIB032-10	HM870942	BOLD:AAB3722	Finland	University of Oulu
Prays fraxinella	MM11966	LEFIF462-10	HM875147	BOLD:AAB3722	Finland	University of Oulu
Prays fraxinella	MM09804	LEFIE715-10	HM874435	BOLD:AAB3722	Finland	University of Oulu
Prays fraxinella	TLMF Lep 12486	LEATC504-13	KM572420	BOLD:AAB3722	Austria	Tiroler Landesmuseum Ferdinandeum

Prays fraxinella	MM06819	LEFID756-10	HM873513	BOLD:AAB3722	Finland	University of Oulu
Prays ruficeps	MM17338	LEFIJ713-10	JF853788	BOLD:AAB3723	Finland	University of Oulu
Prays ruficeps	TLMF Lep 07935	PHLAV116-12	KM572882	BOLD:AAB3723	Austria	inatura, Dornbirn
Prays ruficeps	MM11918	LEFIF446-10	HM875131	BOLD:AAB3723	Finland	University of Oulu
Prays ruficeps	MM00112	LEFIA1311-10	GU828602	BOLD:AAB3723	Finland	University of Oulu
Prays ruficeps	MM11921	LEFIF449-10	HM875134	BOLD:AAB3723	Finland	University of Oulu
Prays ruficeps	MM09690	LEFIE703-10	HM874424	BOLD:AAB3723	Finland	University of Oulu
Prays ruficeps	TLMF Lep 07483	PHLAG804-12	KM572929	BOLD:AAB3723	Austria	inatura, Dornbirn
Prays ruficeps	MM08536	LEFIE220-10	HM873956	BOLD:AAB3723	Finland	University of Oulu
Prays ruficeps	MM11919	LEFIF447-10	HM875132	BOLD:AAB3723	Finland	University of Oulu
Prays ruficeps	MM08535	LEFIE219-10	HM873955	BOLD:AAB3723	Finland	University of Oulu
Prays ruficeps	MM17231	LEFIJ606-10	JF853706	BOLD:AAB3723	Finland	University of Oulu
Pristerognatha penthinana	TLMF Lep 09160	PHLAI598-13	KM571975	BOLD:AAL5814	Austria	Tiroler Landesmuseum Ferdinandeum
Pristerognatha penthinana	MM06218	LEFID325-10	HM873122	BOLD:AAL5814	Finland	University of Oulu
Pristerognatha penthinana	MM18839	LEFIL541-10	JF854647	BOLD:AAL5814	Finland	University of Oulu
Prolita sexpunctella	MM03220	LEFIC014-10	HM871884	BOLD:ABZ5400	Finland	University of Oulu
Prolita sexpunctella	MM06425	LEFID482-10	HM873248	BOLD:ABZ5400	Finland	University of Oulu
Prolita sexpunctella	TLMF Lep 00757	PHLAA717-09	HM426111	BOLD:ABZ5400	Austria	Tiroler Landesmuseum Ferdinandeum
Prolita sexpunctella	MM18233	LEFIK658-10	JF854228	BOLD:ABZ5400	Finland	University of Oulu
Protolampra sobrina	MM01719	LEFIA607-10	HM870856	BOLD:AAE8181	Finland	University of Oulu
Protolampra sobrina	TLMF Lep 08769	PHLAI274-13	KM573450	BOLD:AAE8181	Austria	Tiroler Landesmuseum Ferdinandeum
Protolampra sobrina	MM01718	LEFIA606-10	HM870855	BOLD:AAE8181	Finland	University of Oulu
Protolampra sobrina	MM02737	LEFIB808-10	HM871685	BOLD:AAE8181	Finland	University of Oulu
Pseudargyrotoza conwagana	MM14310	LEFIG334-10	HM876013	BOLD:AAC8983	Finland	University of Oulu
Pseudargyrotoza conwagana	MM11917	LEFIF445-10	HM875130	BOLD:AAC8983	Finland	University of Oulu
Pseudargyrotoza conwagana	TLMF Lep 07927	PHLAV108-12	KM573148	BOLD:AAC8983	Austria	inatura, Dornbirn
Pseudargyrotoza conwagana	MM00680	LEFIB225-10	HM871129	BOLD:AAC8983	Finland	University of Oulu

Pseudatemelia josephinae	MM02513	LEFIB709-10	HM871587	BOLD:AAC3200	Finland	University of Oulu
Pseudatemelia josephinae	MM03048	LEFIB919-10	HM871796	BOLD:AAC3200	Finland	University of Oulu
Pseudatemelia josephinae	MM03049	LEFIB920-10	HM871797	BOLD:AAC3200	Finland	University of Oulu
Pseudatemelia josephinae	MM09249	LEFIE475-10	HM874199	BOLD:AAC3200	Finland	University of Oulu
Pseudatemelia josephinae	MM08032	LEFID992-10	HM873742	BOLD:AAC3200	Finland	University of Oulu
Pseudatemelia josephinae	MM05031	LEFIC834-10	HM872652	BOLD:AAC3200	Finland	University of Oulu
Pseudatemelia josephinae	MM00066	LEFIA1321-10	GU828593	BOLD:AAC3200	Finland	University of Oulu
Pseudatemelia josephinae	MM13333	LEFIF910-10	HM875592	BOLD:AAC3200	Finland	University of Oulu
Pseudatemelia josephinae	BIOUG04118-E06	GMFIQ249-13	KM573554	BOLD:AAC3200	Finland	Biodiversity Institute of Ontario
Pseudatemelia josephinae	MM05190	LEFIC889-10	HM872707	BOLD:AAC3200	Finland	University of Oulu
Pseudatemelia josephinae	MM00037	LEFIA1320-10	GU828585	BOLD:AAC3200	Finland	University of Oulu
Pseudatemelia josephinae	MM11940	LEFIF452-10	HM875137	BOLD:AAC3200	Finland	University of Oulu
Pseudatemelia josephinae	BIOUG04116-A04	GMFIE824-12	KM571950	BOLD:AAC3200	Finland	Biodiversity Institute of Ontario
Pseudatemelia josephinae	MM02514	LEFIB710-10	HM871588	BOLD:AAC3200	Finland	University of Oulu
Pseudatemelia josephinae	MM14391	LEFIG382-10	HM876059	BOLD:AAC3200	Finland	University of Oulu
Pseudatemelia josephinae	MM06757	LEFID703-10	HM873464	BOLD:AAC3200	Finland	University of Oulu
Pseudatemelia josephinae	MM09816	LEFIE723-10	HM874443	BOLD:AAC3200	Finland	University of Oulu
Pseudatemelia josephinae	BIOUG04116-A05	GMFIE825-12	KM573350	BOLD:AAC3200	Finland	Biodiversity Institute of Ontario
Pseudatemelia josephinae	MM02519	LEFIB711-10	HM871589	BOLD:AAC3200	Finland	University of Oulu
Pseudatemelia josephinae	TLMF Lep 08253	PHLAH434-12	KM572183	BOLD:AAC3200	Austria	inatura, Dornbirn
Pseudohermenias abietana	MM02076	LEFIB496-10	HM871391	BOLD:AAD7589	Finland	University of Oulu
Pseudohermenias abietana	MM14222	LEFIG278-10	HM875957	BOLD:AAD7589	Finland	University of Oulu
Pseudohermenias abietana	MM06143	LEFID272-10	HM873070	BOLD:AAD7589	Finland	University of Oulu
Pseudohermenias abietana	TLMF Lep 09929	PHLAW132-13	KM573392	BOLD:AAD7589	Austria	inatura, Dornbirn
Pseudoips prasinana	MM01591	LEFIA509-10	KM572694	BOLD:AAB8807	Finland	University of Oulu
Pseudoips prasinana	MM04729	LEFIC703-10	HM872524	BOLD:AAB8807	Finland	University of Oulu
Pseudoips prasinana	MM01590	LEFIA508-10	KM572991	BOLD:AAB8807	Finland	University of Oulu
Pseudoips prasinana	MM00107	LEFIA1322-10	GU828600	BOLD:AAB8807	Finland	University of Oulu

Pseudoips prasinana	TLMF Lep 07858	PHLAV039-12	KM573129	BOLD:AAB8807	Austria	inatura, Dornbirn
Pseudopanthera macularia	MM18708	LEFIL410-10	KM573636	BOLD:AAC7055	Finland	University of Oulu
Pseudopanthera macularia	MM15835	LEFIG971-10	HM876611	BOLD:AAC7055	Finland	University of Oulu
Pseudopanthera macularia	TLMF Lep 04678	PHLAE363-11	JN269345	BOLD:AAC7055	Austria	Tiroler Landesmuseum Ferdinandeum
Pseudopanthera macularia	MM08577	LEFIE249-10	HM873983	BOLD:AAC7055	Finland	University of Oulu
Pseudopostega crepusculella	TLMF Lep 09199	PHLAI637-13	KM572026	BOLD:AAE8087	Austria	Tiroler Landesmuseum Ferdinandeum
Pseudopostega crepusculella	MM12490	LEFIF612-10	HM875296	BOLD:AAE8088	Finland	University of Oulu
Pseudopostega crepusculella	MM12485	LEFIF608-10	HM875292	BOLD:AAE8088	Finland	University of Oulu
Pseudopostega crepusculella	MM12486	LEFIF609-10	HM875293	BOLD:AAE8088	Finland	University of Oulu
Pseudosciaphila branderiana	MM01908	LEFIB425-10	HM871324	BOLD:AAD9269	Finland	University of Oulu
Pseudosciaphila branderiana	MM13180	LEFIF836-10	HM875519	BOLD:AAD9269	Finland	University of Oulu
Pseudosciaphila branderiana	MM14285	LEFIG318-10	HM875997	BOLD:AAD9269	Finland	University of Oulu
Pseudosciaphila branderiana	TLMF Lep 08000	PHLAV181-12	KM572359	BOLD:AAD9269	Austria	inatura, Dornbirn
Psoricoptera gibbosella	MM05057	LEFIC845-10	HM872663	BOLD:AAD0608	Finland	University of Oulu
Psoricoptera gibbosella	MM05059	LEFIC847-10	HM872665	BOLD:AAD0608	Finland	University of Oulu
Psoricoptera gibbosella	MM05056	LEFIC844-10	HM872662	BOLD:AAD0608	Finland	University of Oulu
Psoricoptera gibbosella	TLMF Lep 08236	PHLAH417-12	KM572410	BOLD:AAD0608	Austria	inatura, Dornbirn
Psoricoptera gibbosella	MM05058	LEFIC846-10	HM872664	BOLD:AAD0608	Finland	University of Oulu
Psyche casta	MM06388	LEFID452-10	HM873223	BOLD:AAC5848	Finland	University of Oulu
Psyche casta	MM14342	LEFIG347-10	HM876024	BOLD:AAC5848	Finland	University of Oulu

Psyche casta	MM18619	LEFIJ417-10	JF853586	BOLD:AAC5848	Finland	University of Oulu
Psyche casta	MM14890	LEFIJ274-10	JF853513	BOLD:AAC5848	Finland	University of Oulu
Psyche casta	MM12066	LEFIF497-10	HM875182	BOLD:AAC5848	Finland	University of Oulu
Psyche casta	MM06556	LEFID563-10	HM873328	BOLD:AAC5848	Finland	University of Oulu
Psyche casta	MM08593	LEFIE258-10	HM873992	BOLD:AAC5848	Finland	University of Oulu
Psyche casta	MM08506	LEFIE191-10	HM873936	BOLD:AAC5848	Finland	University of Oulu
Psyche casta	MM08505	LEFIE190-10	HM873935	BOLD:AAC5848	Finland	University of Oulu
Psyche casta	MM00049	LEFIB020-10	HM870932	BOLD:AAC5848	Finland	University of Oulu
Psyche casta	MM21034	LEFIJ1174-11	KM573082	BOLD:AAC5848	Finland	University of Oulu
Psyche casta	MM21035	LEFIJ1175-11	KM572355	BOLD:AAC5848	Finland	University of Oulu
Psyche casta	MM14341	LEFIG346-10	HM876023	BOLD:AAC5848	Finland	University of Oulu
Psyche casta	MM21175	LEFIJ1315-11	KM573489	BOLD:AAC5848	Finland	University of Oulu
Psyche casta	MM21160	LEFIJ1300-11	KM572516	BOLD:AAC5848	Finland	University of Oulu
Psyche casta	MM06387	LEFIA1325-10	GU828743	BOLD:AAC5848	Finland	University of Oulu
Psyche casta	TLMF Lep 10014	LEATA407-13	KM572052	BOLD:AAC5848	Austria	inatura, Dornbirn
Psyche casta	TLMF Lep 09901	PHLAW104-13	KM572831	BOLD:AAC5848	Austria	Tiroler Landesmuseum Ferdinandeum
Psyche casta	MM06274	LEFID366-10	HM873163	BOLD:ACL8669	Finland	University of Oulu
Psyche casta	MM10019	LEFIE817-10	HM874536	BOLD:ACL8669	Finland	University of Oulu
Psyche casta	MM21036	LEFIJ1176-11	KM572868	BOLD:ACL8669	Finland	University of Oulu
Psyche crassiorella	MM13247	LEFIF869-10	HM875551	BOLD:AAC5849	Finland	University of Oulu
Psyche crassiorella	MM12065	LEFIF496-10	HM875181	BOLD:AAC5849	Finland	University of Oulu
Psyche crassiorella	MM13248	LEFIF870-10	HM875552	BOLD:AAC5849	Finland	University of Oulu
Psyche crassiorella	MM13249	LEFIF871-10	HM875553	BOLD:AAC5849	Finland	University of Oulu
Psyche crassiorella	MM13251	LEFIF873-10	HM875555	BOLD:AAC5849	Finland	University of Oulu
Psyche crassiorella	TLMF Lep 09892	PHLAW095-13	KM572313	BOLD:AAD3657	Austria	Tiroler Landesmuseum Ferdinandeum
Psyche crassiorella	MM13250	LEFIF872-10	HM875554	BOLD:AAD3657	Finland	University of Oulu
Pterapherapteryx sexalata	MM01766	LEFIA649-10	HM870898	BOLD:AAD0485	Finland	University of Oulu
Pterapherapteryx sexalata	MM01511	LEFIA447-10	HM386788	BOLD:AAD0485	Finland	University of Oulu
Pterapherapteryx sexalata	MM04577	LEFIC635-10	HM872456	BOLD:AAD0485	Finland	University of Oulu
Pterapherapteryx sexalata	MM01436	LEFIA379-10	HM386721	BOLD:AAD0485	Finland	University of Oulu
Pterapherapteryx sexalata	TLMF Lep 08803	PHLAI308-13	KM573121	BOLD:AAD0485	Austria	Tiroler Landesmuseum Ferdinandeum

Pterostoma palpina	MM00990	LEFIA072-10	HM396419	BOLD:AAB5417	Finland	University of Oulu
Pterostoma palpina	TLMF Lep 07830	PHLAV011-12	KM572088	BOLD:AAB5417	Austria	inatura, Dornbirn
Pterostoma palpina	MM08247	LEFIE081-10	HM873828	BOLD:AAB5417	Finland	University of Oulu
Pterostoma palpina	MM14081	LEFIG200-10	HM875880	BOLD:AAB5417	Finland	University of Oulu
Pterostoma palpina	MM04598	LEFIC651-10	HM872472	BOLD:AAB5417	Finland	University of Oulu
Pterostoma palpina	MM00663	LEFIB211-10	HM871115	BOLD:AAB5417	Finland	University of Oulu
Pterostoma palpina	MM10850	LEFIF205-10	HM874899	BOLD:AAB5417	Finland	University of Oulu
Pterostoma palpina	MM12580	LEFIF653-10	HM875337	BOLD:AAB5417	Finland	University of Oulu
Pterostoma palpina	MM02794	LEFIB834-10	HM871711	BOLD:AAB5417	Finland	University of Oulu
Pterostoma palpina	MM00991	LEFIA073-10	HM396420	BOLD:AAB5417	Finland	University of Oulu
Pterostoma palpina	MM05423	LEFID003-10	HM872817	BOLD:AAB5417	Finland	University of Oulu
Pterotopteryx dodecadactyla	MM15763	LEFIG899-10	HM876543	BOLD:AAL2276	Finland	University of Oulu
Pterotopteryx dodecadactyla	TLMF Lep 08735	PHLAH931-12	KM573355	BOLD:AAL2276	Austria	Tiroler Landesmuseum Ferdinandeum
Pterotopteryx dodecadactyla	MM18646	LEFIL336-10	KM572283	BOLD:AAL2276	Finland	University of Oulu
Ptilodon capucina	MM02792	LEFIB833-10	HM871710	BOLD:AAC0728	Finland	University of Oulu
Ptilodon capucina	TLMF Lep 07879	PHLAV060-12	KM572752	BOLD:AAC0728	Austria	inatura, Dornbirn
Ptilodon capucina	MM00992	LEFIA074-10	HM396421	BOLD:AAC0728	Finland	University of Oulu
Ptilodon capucina	MM00993	LEFIA075-10	HM396422	BOLD:AAC0728	Finland	University of Oulu
Pyralis farinalis	MM12381	LEFIF580-10	HM875264	BOLD:AAB3316	Finland	University of Oulu
Pyralis farinalis	MM00051	LEFIA1334-10	GU828590	BOLD:AAB3316	Finland	University of Oulu
Pyralis farinalis	TLMF Lep 08003	PHLAV184-12	KM573035	BOLD:AAY8728	Austria	inatura, Dornbirn
Pyrausta aerealis	TLMF Lep 00912	PHLAB112-10	HM381484	BOLD:AAC7368	Austria	Tiroler Landesmuseum Ferdinandeum
Pyrausta aerealis	MM17839	LEFIK264-10	KM572264	BOLD:AAF8527	Finland	University of Oulu
Pyrausta aurata	MM10557	LEFIF104-10	HM874808	BOLD:AAB6530	Finland	University of Oulu
Pyrausta aurata	TLMF Lep 08425	PHLAH606-12	KM572784	BOLD:AAB6530	Austria	inatura, Dornbirn
Pyrausta aurata	MM10556	LEFIF103-10	HM874807	BOLD:AAB6530	Finland	University of Oulu
Pyrausta aurata	MM10555	LEFIF102-10	HM874806	BOLD:AAB6530	Finland	University of Oulu
Pyrausta aurata	MM17277	LEFIJ652-10	JF853747	BOLD:AAB6530	Finland	University of Oulu

Pyrausta cingulata	TLMF Lep 04290	PHLAE070-11	JN277468	BOLD:ACF2687	Austria	Tiroler Landesmuseum Ferdinandeum
Pyrausta cingulata	MM03376	LEFIC080-10	HM871948	BOLD:ACF2687	Finland	University of Oulu
Pyrausta cingulata	MM04220	LEFIC512-10	HM872336	BOLD:ACF2687	Finland	University of Oulu
Pyrausta cingulata	MM04219	LEFIC511-10	HM872335	BOLD:ACF2687	Finland	University of Oulu
Pyrausta cingulata	MM18799	LEFIL501-10	JF854628	BOLD:ACF2687	Finland	University of Oulu
Pyrausta cingulata	TLMF Lep 09889	PHLAW092-13	KM572643	BOLD:ACF2687	Austria	Tiroler Landesmuseum Ferdinandeum
Pyrausta despicata	MM06837	LEFID771-10	HM873528	BOLD:AAB9618	Finland	University of Oulu
Pyrausta despicata	MM03447	LEFIC114-10	HM871961	BOLD:AAB9618	Finland	University of Oulu
Pyrausta despicata	MM13003	LEFIF774-10	HM875458	BOLD:AAB9618	Finland	University of Oulu
Pyrausta despicata	MM11762	LEFIF398-10	HM875083	BOLD:AAB9618	Finland	University of Oulu
Pyrausta despicata	TLMF Lep 07893	PHLAV074-12	KM572852	BOLD:AAB9618	Austria	inatura, Dornbirn
Pyrausta purpuralis	TLMF Lep 09915	PHLAW118-13	KM573510	BOLD:AAB6531	Austria	Tiroler Landesmuseum Ferdinandeum
Pyrausta purpuralis	MM11676	LEFIF388-10	HM875073	BOLD:AAB6531	Finland	University of Oulu
Pyrausta purpuralis	TLMF Lep 07919	PHLAV100-12	KM572309	BOLD:AAB6531	Austria	inatura, Dornbirn
Pyrausta purpuralis	MM01882	LEFIB412-10	HM871311	BOLD:AAB6531	Finland	University of Oulu
Pyrausta purpuralis	MM14677	LEFIG539-10	HM876212	BOLD:AAB6531	Finland	University of Oulu
Pyrausta purpuralis	TLMF Lep 08102	PHLAV283-12	KM573546	BOLD:AAB6531	Austria	inatura, Dornbirn
Pyrgus alveus	TLMF Lep 09842	PHLAW045-13	KM572268	BOLD:ABZ6967	Austria	Tiroler Landesmuseum Ferdinandeum
Pyrgus alveus	MM06791	LEFID733-10	HM873490	BOLD:ABZ6967	Finland	University of Oulu
Pyrgus alveus	MM18681	LEFIL371-10	JN277847	BOLD:ABZ6967	Finland	University of Oulu
Pyrgus andromedae	MM17109	LEFIJ484-10	JN277846	BOLD:ACE9283	Finland	University of Oulu
Pyrgus andromedae	MM04102	LEFIA748-10	HM386892	BOLD:ACE9283	Finland	University of Oulu
Pyrgus andromedae	TLMF Lep 09802	PHLAW005-13	KM573527	BOLD:ACE9283	Austria	Tiroler Landesmuseum Ferdinandeum
Pyrgus andromedae	MM17108	LEFIJ483-10	JN277845	BOLD:ACE9283	Finland	University of Oulu
Pyrgus andromedae	MM10572	LEFIF116-10	HQ570386	BOLD:ACE9283	Finland	University of Oulu
Pyrgus malvae	MM00029	LEFIB013-10	HM870926	BOLD:AAB6141	Finland	University of Oulu
Pyrgus malvae	TLMF Lep 08278	PHLAH459-12	KM572761	BOLD:AAB6141	Austria	inatura, Dornbirn
Pyrgus malvae	TLMF Lep 09832	PHLAW035-13	KM572512	BOLD:AAB6141	Austria	Tiroler Landesmuseum Ferdinandeum
Pyrgus malvae	MM00850	LEFIB319-10	HM871219	BOLD:AAB6141	Finland	University of Oulu
Pyrgus malvae	MM00034	LEFIA1335-10	GU828583	BOLD:AAB6141	Finland	University of Oulu
Pyrrhia umbra	MM05114	LEFIA1336-10	GU828712	BOLD:AAC7527	Finland	University of Oulu

Pyrrhia umbra	TLMF Lep 04661	PHLAE346-11	JN272577	BOLD:AAC7527	Austria	Tiroler Landesmuseum Ferdinandeum
Pyrrhia umbra	MM05115	LEFIC863-10	HM872681	BOLD:AAC7527	Finland	University of Oulu
Pyrrhia umbra	MM01125	LEFIA155-10	HM396501	BOLD:AAC7527	Finland	University of Oulu
Pyrrhia umbra	MM01126	LEFIA156-10	HM396502	BOLD:AAC7527	Finland	University of Oulu
Recurvaria leucatella	TLMF Lep 08220	PHLAH401-12	KM573358	BOLD:AAD6088	Austria	inatura, Dornbirn
Recurvaria leucatella	MM05493	LEFID039-10	HM872853	BOLD:AAD6088	Finland	University of Oulu
Recurvaria leucatella	MM05044	LEFIC840-10	HM872658	BOLD:AAD6088	Finland	University of Oulu
Recurvaria leucatella	TLMF Lep 08296	PHLAH477-12	KM572548	BOLD:AAD6088	Austria	inatura, Dornbirn
Recurvaria leucatella	MM02317	LEFIB602-10	HM871481	BOLD:AAD6088	Finland	University of Oulu
Rheumaptera	BIOUG04490-F08	GMFIM274-13	KM572699	BOLD:AAA5435	Finland	Biodiversity Institute of Ontario
Rheumaptera hastata	MM18452	LEFIK877-10	JF854333	BOLD:AAA5435	Finland	University of Oulu
Rheumaptera hastata	TLMF Lep 09874	PHLAW077-13	KM573191	BOLD:AAA5435	Austria	Tiroler Landesmuseum Ferdinandeum
Rheumaptera hastata	MM10108	LEFIE861-10	HM874579	BOLD:AAA5435	Finland	University of Oulu
Rheumaptera hastata	MM06321	LEFID402-10	HM873199	BOLD:AAA5435	Finland	University of Oulu
Rheumaptera hastata	MM18450	LEFIK875-10	JF854331	BOLD:AAA5435	Finland	University of Oulu
Rheumaptera hastata	MM00580	LEFIB174-10	HM871078	BOLD:AAA5435	Finland	University of Oulu
Rheumaptera hastata	MM18451	LEFIK876-10	JF854332	BOLD:AAA5435	Finland	University of Oulu
Rheumaptera subhastata	MM18462	LEFIK887-10	JF854338	BOLD:AAA5435	Finland	University of Oulu
Rheumaptera subhastata	MM06292	LEFID381-10	HM873178	BOLD:AAA5435	Finland	University of Oulu
Rheumaptera subhastata	MM06308	LEFID394-10	HM873191	BOLD:AAA5435	Finland	University of Oulu
Rheumaptera subhastata	MM18459	LEFIK884-10	JX034571	BOLD:AAA5435	Finland	University of Oulu
Rheumaptera subhastata	MM18457	LEFIK882-10	JF854336	BOLD:AAA5435	Finland	University of Oulu
Rheumaptera subhastata	MM17987	LEFIK412-10	JF854041	BOLD:AAA5435	Finland	University of Oulu
Rheumaptera subhastata	MM18460	LEFIK885-10	JF854337	BOLD:AAA5435	Finland	University of Oulu
Rheumaptera subhastata	TLMF Lep 09873	PHLAW076-13	KM573131	BOLD:AAA5435	Austria	Tiroler Landesmuseum Ferdinandeum
Rheumaptera subhastata	MM18456	LEFIK881-10	JN279414	BOLD:AAA5435	Finland	University of Oulu
Rheumaptera subhastata	MM18454	LEFIK879-10	JF854334	BOLD:AAA5435	Finland	University of Oulu
Rheumaptera subhastata	MM18455	LEFIK880-10	JF854335	BOLD:AAA5435	Finland	University of Oulu
Rheumaptera subhastata	MM17986	LEFIK411-10	JF854040	BOLD:AAA5435	Finland	University of Oulu
Rheumaptera subhastata	MM10312	LEFIE932-10	HM874649	BOLD:AAA5436	Finland	University of Oulu
Rheumaptera subhastata	MM18463	LEFIK888-10	JF854339	BOLD:AAA5436	Finland	University of Oulu

Rheumaptera subhastata	MM18464	LEFIK889-10	JF854340	BOLD:AAA5436	Finland	University of Oulu
Rhigognostis senilella	TLMF Lep 09168	PHLAI606-13	KM573276	BOLD:AAF7514	Austria	Tiroler Landesmuseum Ferdinandeum
Rhigognostis senilella	TLMF Lep 07399	PHLAG720-12	KM572825	BOLD:AAF7514	Austria	inatura, Dornbirn
Rhigognostis senilella	MM08542	LEFIE225-10	HQ570369	BOLD:AAF7514	Finland	University of Oulu
Rhigognostis senilella	MM08541	LEFIE224-10	HQ570368	BOLD:AAF7514	Finland	University of Oulu
Rhigognostis senilella	MM08348	LEFIE132-10	HM873880	BOLD:AAF7514	Finland	University of Oulu
Rhopobota naevana	MM22966	LEFIJ1679-13	KM572725	BOLD:AAA9812	Finland	University of Oulu
Rhopobota naevana	MM22967	LEFIJ1680-13	KM572945	BOLD:AAA9812	Finland	University of Oulu
Rhopobota naevana	TLMF Lep 08227	PHLAH408-12	KM571957	BOLD:AAA9812	Austria	inatura, Dornbirn
Rhopobota naevana	MM22968	LEFIJ1681-13	KM571977	BOLD:AAA9812	Finland	University of Oulu
Rhopobota naevana	MM22969	LEFIJ1682-13	KM572455	BOLD:AAA9812	Finland	University of Oulu
Rhopobota naevana	MM13304	LEFIF895-10	HM875577	BOLD:AAA9812	Finland	University of Oulu
Rhopobota naevana	TLMF Lep 08098	PHLAV279-12	KM572978	BOLD:AAA9812	Austria	inatura, Dornbirn
Rhopobota naevana	MM00760	LEFIB276-10	HM871178	BOLD:AAA9812	Finland	University of Oulu
Rhopobota naevana	MM02121	LEFIB520-10	HM871409	BOLD:AAA9812	Finland	University of Oulu
Rhopobota naevana	MM08217	LEFIE073-10	HM873820	BOLD:AAA9812	Finland	University of Oulu
Rhopobota naevana	MM22961	LEFIJ1674-13	KM571956	BOLD:AAA9812	Finland	University of Oulu
Rhopobota naevana	MM22970	LEFIJ1683-13	KM573591	BOLD:AAA9812	Finland	University of Oulu
Rhopobota naevana	MM22962	LEFIJ1675-13	KM572177	BOLD:AAA9812	Finland	University of Oulu
Rhopobota naevana	MM22963	LEFIJ1676-13	KM573495	BOLD:AAA9812	Finland	University of Oulu
Rhopobota naevana	MM22964	LEFIJ1677-13	KM572855	BOLD:AAA9812	Finland	University of Oulu
Rhopobota naevana	MM22965	LEFIJ1678-13	KM572673	BOLD:AAA9812	Finland	University of Oulu
Rhopobota stagnana	MM14095	LEFIG209-10	HM875889	BOLD:AAF7954	Finland	University of Oulu
Rhopobota stagnana	MM14094	LEFIG208-10	HM875888	BOLD:AAF7954	Finland	University of Oulu
Rhopobota stagnana	MM11017	LEFIF236-10	HM874929	BOLD:AAF7954	Finland	University of Oulu
Rhopobota stagnana	TLMF Lep 08479	PHLAH660-12	KM573004	BOLD:AAF7954	Austria	inatura, Dornbirn
Rhyacionia pinicolana	TLMF Lep 00923	PHLAB123-10	HM381493	BOLD:AAC4601	Austria	Tiroler Landesmuseum Ferdinandeum
Rhyacionia pinicolana	TLMF Lep 00916	PHLAB116-10	HM381488	BOLD:AAC4601	Austria	Tiroler Landesmuseum Ferdinandeum
Rhyacionia pinicolana	MM02096	LEFIB505-10	HM871399	BOLD:AAC4601	Finland	University of Oulu
Rhyacionia pinicolana	TLMF Lep 08080	PHLAV261-12	KM573435	BOLD:AAC4601	Austria	inatura, Dornbirn
Rhyacionia pinicolana	MM03110	LEFIB951-10	HM871828	BOLD:AAC4601	Finland	University of Oulu

Rhyacionia pinicolana	MM09576	LEFIE634-10	HM874357	BOLD:AAC4601	Finland	University of Oulu
Rhyacionia pinivorana	TLMF Lep 07925	PHLAV106-12	KM572640	BOLD:AAB9878	Austria	inatura, Dornbirn
Rhyacionia pinivorana	MM14337	LEFIG345-10	HM876022	BOLD:AAB9878	Finland	University of Oulu
Rhyacionia pinivorana	MM00677	LEFIB223-10	HM871127	BOLD:AAB9878	Finland	University of Oulu
Rhyacionia pinivorana	MM02147	LEFIB536-10	HM871422	BOLD:AAB9878	Finland	University of Oulu
Rhyacionia pinivorana	MM03224	LEFIC015-10	HM871885	BOLD:AAB9878	Finland	University of Oulu
Rhyparia purpurata	TLMF Lep 05623	PHLAF453-11	KM572278	BOLD:AAD5293	Austria	Tiroler Landesmuseum Ferdinandeum
Rhyparia purpurata	MM08355	LEFIE135-10	HM873883	BOLD:AAD5293	Finland	University of Oulu
Rhyparia purpurata	MM00323	LEFIB047-10	HM870956	BOLD:AAD5293	Finland	University of Oulu
Rhyparia purpurata	MM08354	LEFIE134-10	HM873882	BOLD:AAD5293	Finland	University of Oulu
Rivula sericealis	MM02905	LEFIJ048-10	KM573650	BOLD:AAB4777	Finland	University of Oulu
Rivula sericealis	MM01409	LEFIA356-10	HM386699	BOLD:AAB4777	Finland	University of Oulu
Rivula sericealis	MM07817	LEFIJ105-10	KM573668	BOLD:AAB4777	Finland	University of Oulu
Rivula sericealis	MM05176	LEFIC886-10	HM872704	BOLD:AAB4777	Finland	University of Oulu
Rivula sericealis	MM00324	LEFIA1341-10	KM572352	BOLD:AAB4777	Finland	University of Oulu
Rivula sericealis	MM02904	LEFIB862-10	HM871739	BOLD:AAB4777	Finland	University of Oulu
Rivula sericealis	TLMF Lep 07898	PHLAV079-12	KM572095	BOLD:AAB4777	Austria	inatura, Dornbirn
Rivula sericealis	MM11634	LEFIJ177-10	KM572909	BOLD:ABX5628	Finland	University of Oulu
Rivula sericealis	MM01404	LEFIA1340-10	GU828664	BOLD:ABX5628	Finland	University of Oulu
Roeslerstammia erxlebella	MM15529	LEFIG665-10	HM876326	BOLD:AAE7694	Finland	University of Oulu
Roeslerstammia erxlebella	MM15528	LEFIG664-10	HM876325	BOLD:AAE7694	Finland	University of Oulu
Roeslerstammia erxlebella	TLMF Lep 08240	PHLAH421-12	KM573156	BOLD:AAE7694	Austria	inatura, Dornbirn
Roeslerstammia erxlebella	MM00303	LEFIA1342-10	GU828627	BOLD:AAE7694	Finland	University of Oulu
Roeslerstammia erxlebella	MM10170	LEFIE891-10	HM874609	BOLD:AAE7694	Finland	University of Oulu
Roeslerstammia erxlebella	MM21113	LEFIJ1253-11	KM572990	BOLD:AAE7694	Finland	University of Oulu
Saturnia pavonia	TLMF Lep 04435	PHLAE215-11	JN278460	BOLD:AAA8149	Austria	Tiroler Landesmuseum Ferdinandeum
Saturnia pavonia	MM12537	LEFIF637-10	HM875321	BOLD:AAA8149	Finland	University of Oulu
Saturnia pavonia	MM00002	LEFIA1351-10	GU828573	BOLD:AAA8149	Finland	University of Oulu
Saturnia pavonia	TLMF Lep 04436	PHLAE216-11	JN278461	BOLD:AAA8149	Austria	Tiroler Landesmuseum Ferdinandeum
Sciota adelphella	MM17812	LEFIK237-10	JF853944	BOLD:AAE3809	Finland	University of Oulu
Sciota adelphella	MM17811	LEFIK236-10	JF853943	BOLD:AAE3809	Finland	University of Oulu

Sciota adelphella	TLMF Lep 09778	LEATA361-13	KM572755	BOLD:AAE3809	Austria	Tiroler Landesmuseum Ferdinandeum
Sciota adelphella	MM17810	LEFIK235-10	KM572798	BOLD:AAE3809	Finland	University of Oulu
Sciota hostilis	MM06867	LEFID792-10	HM873549	BOLD:AAD3590	Finland	University of Oulu
Sciota hostilis	TLMF Lep 07473	PHLAG794-12	KM573501	BOLD:AAD3590	Austria	inatura, Dornbirn
Sciota hostilis	MM02443	LEFIB663-10	HM871541	BOLD:AAD3590	Finland	University of Oulu
Sciota hostilis	TLMF Lep 09937	PHLAW140-13	KM573526	BOLD:AAD3590	Austria	inatura, Dornbirn
Sciota hostilis	MM11797	LEFIF414-10	HM875099	BOLD:AAD3590	Finland	University of Oulu
Scoliopteryx libatrix	TLMF Lep 08183	PHLAV364-12	KM572606	BOLD:ACE7197	Austria	inatura, Dornbirn
Scoliopteryx libatrix	MM00407	LEFIA1355-10	GU828641	BOLD:ACE7197	Finland	University of Oulu
Scoliopteryx libatrix	MM14733	LEFIG576-10	HM876247	BOLD:ACE7197	Finland	University of Oulu
Scoliopteryx libatrix	MM08201	LEFIE068-10	HM873815	BOLD:ACE7197	Finland	University of Oulu
Scoliopteryx libatrix	MM14732	LEFIG575-10	HM876246	BOLD:ACE7197	Finland	University of Oulu
Scoliopteryx libatrix	TLMF Lep 04639	PHLAE324-11	JN263519	BOLD:ACE7197	Austria	Tiroler Landesmuseum Ferdinandeum
Scoparia ambigualis	MM06656	LEFID633-10	HM873396	BOLD:AAB1555	Finland	University of Oulu
Scoparia ambigualis	MM06655	LEFID632-10	HM873395	BOLD:AAB1555	Finland	University of Oulu
Scoparia ambigualis	MM13358	LEFIF921-10	HM875603	BOLD:AAB1555	Finland	University of Oulu
Scoparia ambigualis	MM14230	LEFIG284-10	HM875963	BOLD:AAB1555	Finland	University of Oulu
Scoparia ambigualis	MM05338	LEFIC962-10	HM872777	BOLD:AAB1555	Finland	University of Oulu
Scoparia ambigualis	TLMF Lep 07491	PHLAG812-12	KM572507	BOLD:AAB1555	Austria	inatura, Dornbirn
Scoparia ambigualis	MM15781	LEFIG917-10	HM876561	BOLD:AAB1555	Finland	University of Oulu
Scoparia ambigualis	MM06657	LEFID634-10	HM873397	BOLD:AAB1555	Finland	University of Oulu
Scoparia ambigualis	MM15780	LEFIG916-10	HM876560	BOLD:AAB1555	Finland	University of Oulu
Scoparia ambigualis	MM15779	LEFIG915-10	HM876559	BOLD:AAB1555	Finland	University of Oulu
Scoparia ambigualis	MM03234	LEFIC022-10	HM871892	BOLD:AAB1555	Finland	University of Oulu
Scoparia ambigualis	MM02411	LEFIB646-10	HM871525	BOLD:AAB1555	Finland	University of Oulu
Scoparia ambigualis	MM14296	LEFIG325-10	HM876004	BOLD:AAB1555	Finland	University of Oulu
Scoparia ambigualis	MM15778	LEFIG914-10	HM876558	BOLD:AAB1555	Finland	University of Oulu
Scoparia ambigualis	MM15782	LEFIG918-10	HM876562	BOLD:AAB1555	Finland	University of Oulu
Scoparia ancipitella	TLMF Lep 08734	PHLAH930-12	KM572248	BOLD:AAB1556	Austria	Tiroler Landesmuseum Ferdinandeum
Scoparia ancipitella	MM06854	LEFID785-10	HM873542	BOLD:AAB1556	Finland	University of Oulu
Scoparia ancipitella	MM00695	LEFIB233-10	HM871136	BOLD:AAB1556	Finland	University of Oulu

Scoparia ancipitella	MM02412	LEFIB647-10	HM871526	BOLD:AAB1556	Finland	University of Oulu
Scoparia ancipitella	MM02953	LEFIB880-10	HM871757	BOLD:AAB1556	Finland	University of Oulu
Scoparia ancipitella	MM02952	LEFIB879-10	HM871756	BOLD:AAB1556	Finland	University of Oulu
Scoparia ancipitella	TLMF Lep 09185	PHLAI623-13	KM572946	BOLD:AAB1556	Austria	Tiroler Landesmuseum Ferdinandeum
Scoparia ancipitella	BIOUG04116-E05	GMFIO699-13	KM572957	BOLD:AAB1556	Finland	Biodiversity Institute of Ontario
Scoparia ancipitella	BIOUG04116-E06	GMFIO700-13	KM572838	BOLD:AAB1556	Finland	Biodiversity Institute of Ontario
Scoparia basistrigalis	MM17225	LEFIJ600-10	JF853700	BOLD:AAB1555	Finland	University of Oulu
Scoparia basistrigalis	MM11763	LEFIF399-10	HM875084	BOLD:AAB1555	Finland	University of Oulu
Scoparia basistrigalis	TLMF Lep 08045	PHLAV226-12	KM572828	BOLD:AAB1555	Austria	inatura, Dornbirn
Scoparia basistrigalis	TLMF Lep 08026	PHLAV207-12	KM571989	BOLD:AAB1555	Austria	inatura, Dornbirn
Scoparia basistrigalis	TLMF Lep 08480	PHLAH661-12	KM572142	BOLD:AAB1555	Austria	inatura, Dornbirn
Scoparia basistrigalis	TLMF Lep 08021	PHLAV202-12	KM573022	BOLD:AAB1555	Austria	inatura, Dornbirn
Scoparia basistrigalis	MM11773	LEFIF404-10	HM875089	BOLD:AAB1555	Finland	University of Oulu
Scoparia basistrigalis	MM17226	LEFIJ601-10	JF853701	BOLD:AAB1555	Finland	University of Oulu
Scoparia basistrigalis	MM11766	LEFIF402-10	HM875087	BOLD:AAB1555	Finland	University of Oulu
Scoparia basistrigalis	MM11765	LEFIF401-10	HM875086	BOLD:AAB1555	Finland	University of Oulu
Scoparia basistrigalis	MM11764	LEFIF400-10	HM875085	BOLD:AAB1555	Finland	University of Oulu
Scoparia basistrigalis	MM17982	LEFIK407-10	JF854036	BOLD:AAB1555	Finland	University of Oulu
Scoparia pyralella	TLMF Lep 07522	PHLAG843-12	KM572407	BOLD:AAC1440	Austria	Tiroler Landesmuseum Ferdinandeum
Scoparia pyralella	MM06636	LEFID620-10	HM873385	BOLD:ABZ0561	Finland	University of Oulu
Scoparia pyralella	MM14374	LEFIG369-10	HM876046	BOLD:ABZ0561	Finland	University of Oulu
Scoparia pyralella	MM02403	LEFIB641-10	HM871520	BOLD:ABZ0561	Finland	University of Oulu
Scoparia subfusca	TLMF Lep 00785	PHLAA745-09	HM426133	BOLD:AAB1557	Austria	Tiroler Landesmuseum Ferdinandeum
Scoparia subfusca	TLMF Lep 00783	PHLAA743-09	HM426131	BOLD:AAB1557	Austria	Tiroler Landesmuseum Ferdinandeum
Scoparia subfusca	MM15776	LEFIG912-10	HM876556	BOLD:AAB1557	Finland	University of Oulu
Scoparia subfusca	MM02409	LEFIB644-10	HM871523	BOLD:AAB1557	Finland	University of Oulu
Scoparia subfusca	MM02410	LEFIB645-10	HM871524	BOLD:AAB1557	Finland	University of Oulu
Scoparia subfusca	MM04962	LEFIC802-10	HM872621	BOLD:AAB1557	Finland	University of Oulu
Scopula caricaria	MM17860	LEFIK285-10	KF807405	BOLD:AAF0636	Finland	University of Oulu
Scopula caricaria	MM17859	LEFIK284-10	KF808136	BOLD:AAF0636	Finland	University of Oulu
Scopula caricaria	TLMF Lep 08526	PHLAH707-12	KF807801	BOLD:AAF0636	Austria	inatura, Dornbirn

Scopula caricaria	MM10462	LEFIF023-10	HM874737	BOLD:AAF0636	Finland	University of Oulu
Scopula floslactata	MM01338	LEFIA294-10	HM386637	BOLD:AAC5048	Finland	University of Oulu
Scopula floslactata	TLMF Lep 07974	PHLAV155-12	KF807782	BOLD:AAC5048	Austria	inatura, Dornbirn
Scopula floslactata	BIOUG04490-E11	GMFID735-12	KM571967	BOLD:AAC5048	Finland	Biodiversity Institute of Ontario
Scopula floslactata	MM01339	LEFIA295-10	HM386638	BOLD:AAC5048	Finland	University of Oulu
Scopula floslactata	MM02862	LEFIB855-10	HM871732	BOLD:AAC5048	Finland	University of Oulu
Scopula incanata	TLMF Lep 08498	PHLAH679-12	KF807436	BOLD:AAC9956	Austria	inatura, Dornbirn
Scopula incanata	MM18413	LEFIK838-10	JN285636	BOLD:AAC9956	Finland	University of Oulu
Scopula incanata	MM12852	LEFIF733-10	HM875417	BOLD:AAC9956	Finland	University of Oulu
Scopula incanata	MM04558	LEFIC624-10	HM872445	BOLD:AAC9956	Finland	University of Oulu
Scopula ornata	TLMF Lep 08148	PHLAV329-12	KF807279	BOLD:AAB1550	Austria	inatura, Dornbirn
Scopula ornata	MM07059	LEFID886-10	KF807391	BOLD:AAB1550	Finland	University of Oulu
Scopula ornata	MM10461	LEFIF022-10	HM874736	BOLD:AAB1550	Finland	University of Oulu
Scopula ternata	MM08463	LEFIE179-10	HM873925	BOLD:AAA5384	Finland	University of Oulu
Scopula ternata	TLMF Lep 00314	PHLAA274-09	HM425810	BOLD:AAA5384	Austria	Tiroler Landesmuseum Ferdinandeum
Scopula ternata	MM01445	LEFIA388-10	HM386730	BOLD:AAA5384	Finland	University of Oulu
Scopula ternata	MM01444	LEFIA387-10	HM386729	BOLD:AAA5384	Finland	University of Oulu
Scopula virgulata	MM22893	LEFIJ2041-13	KM572020	BOLD:AAP7443	Finland	University of Oulu
Scopula virgulata	MM18696	LEFIL398-10	JF854583	BOLD:AAP7443	Finland	University of Oulu
Scopula virgulata	MM17429	LEFIJ804-10	JF853833	BOLD:AAP7443	Finland	University of Oulu
Scopula virgulata	MM17430	LEFIJ805-10	KM573709	BOLD:AAP7443	Finland	University of Oulu
Scopula virgulata	TLMF Lep 08128	PHLAV309-12	KF807655	BOLD:AAP7443	Austria	inatura, Dornbirn
Scopula virgulata	MM18695	LEFIL397-10	JF854582	BOLD:AAP7443	Finland	University of Oulu
Scotopteryx chenopodiata	MM12825	LEFIF729-10	HM875413	BOLD:AAB5023	Finland	University of Oulu
Scotopteryx chenopodiata	MM01399	LEFIA352-10	HM386695	BOLD:AAB5023	Finland	University of Oulu
Scotopteryx chenopodiata	MM21178	LEFIJ1318-11	KM572556	BOLD:AAB5023	Finland	University of Oulu
Scotopteryx chenopodiata	TLMF Lep 08494	PHLAH675-12	KM572542	BOLD:AAB5023	Austria	inatura, Dornbirn
Scotopteryx chenopodiata	MM08120	LEFIE037-10	HM873786	BOLD:AAB5023	Finland	University of Oulu
Scrobipalpa acuminatella	MM02537	LEFIB720-10	HM871598	BOLD:AAC1644	Finland	University of Oulu
Scrobipalpa acuminatella	MM17275	LEFIJ650-10	JF853745	BOLD:AAC1644	Finland	University of Oulu
Scrobipalpa acuminatella	MM14050	LEFIG182-10	HM875862	BOLD:AAC1644	Finland	University of Oulu

Scrobipalpa acuminatella	MM21125	LEFIJ1265-11	KF808535	BOLD:AAC1644	Finland	University of Oulu
Scrobipalpa acuminatella	MM03835	LEFIC330-10	HM872174	BOLD:AAC1644	Finland	University of Oulu
Scrobipalpa acuminatella	TLMF Lep 00902	PHLAB102-10	HM381474	BOLD:AAC1644	Austria	Tiroler Landesmuseum Ferdinandeum
Scrobipalpa pauperella	TLMF Lep 12470	LEATC488-13	KM573146	BOLD:AAF1201	Austria	Tiroler Landesmuseum Ferdinandeum
Scrobipalpa pauperella	MM00637	LEFIB197-10	HM871101	BOLD:AAF1201	Finland	University of Oulu
Scrobipalpa pauperella	MM15635	LEFIG771-10	HM876423	BOLD:AAF1201	Finland	University of Oulu
Scrobipalpa pauperella	MM15636	LEFIG772-10	HM876424	BOLD:AAF1201	Finland	University of Oulu
Scrobipalpopsis petasitis	MM10495	LEFIF055-10	HM874766	BOLD:AAE1641	Finland	University of Oulu
Scrobipalpopsis petasitis	TLMF Lep 00866	PHLAB066-10	HQ968240	BOLD:AAE1641	Austria	Tiroler Landesmuseum Ferdinandeum
Scrobipalpopsis petasitis	TLMF Lep 00865	PHLAB065-10	HQ968239	BOLD:AAE1641	Austria	Tiroler Landesmuseum Ferdinandeum
Scrobipalpopsis petasitis	MM11172	LEFIF351-10	HM875036	BOLD:AAE1641	Finland	University of Oulu
Scrobipalpopsis petasitis	MM18235	LEFIK660-10	JF854230	BOLD:AAE1641	Finland	University of Oulu
Scythris laminella	TLMF Lep 12528	LEATC546-13	KM572771	BOLD:AAJ7286	Austria	Tiroler Landesmuseum Ferdinandeum
Scythris laminella	MM15608	LEFIG744-10	HM876400	BOLD:AAJ7286	Finland	University of Oulu
Scythris laminella	MM17301	LEFIJ676-10	JF853764	BOLD:AAJ7286	Finland	University of Oulu
Scythris laminella	MM17302	LEFIJ677-10	JF853765	BOLD:AAJ7286	Finland	University of Oulu
Scythris noricella	TLMF Lep 08743	PHLAH939-12	KM573438	BOLD:AAA9046	Austria	Tiroler Landesmuseum Ferdinandeum
Scythris noricella	MM06104	LEFID241-10	HM873040	BOLD:AAA9046	Finland	University of Oulu
Scythris obscurella	MM18204	LEFIK629-10	JF854203	BOLD:AAF1117	Finland	University of Oulu
Scythris obscurella	MM06455	LEFID497-10	HM873262	BOLD:AAF1117	Finland	University of Oulu
Scythris obscurella	TLMF Lep 04510	PHLAE385-11	KM573194	BOLD:AAF1117	Austria	Tiroler Landesmuseum Ferdinandeum
Scythris obscurella	MM06456	LEFID498-10	HM873263	BOLD:AAF1117	Finland	University of Oulu
Selenia dentaria	MM01267	LEFIA235-10	HM386579	BOLD:AAB7343	Finland	University of Oulu
Selenia dentaria	MM00475	LEFIB119-10	HM871025	BOLD:AAB7343	Finland	University of Oulu
Selenia dentaria	TLMF Lep 08525	PHLAH706-12	KM573104	BOLD:AAB7343	Austria	inatura, Dornbirn
Selenia dentaria	MM08423	LEFIE167-10	HM873913	BOLD:AAB7343	Finland	University of Oulu
Selenia dentaria	MM01268	LEFIA236-10	HM386580	BOLD:AAB7343	Finland	University of Oulu
Selenia lunularia	MM03711	LEFIC275-10	HM872119	BOLD:AAB1585	Finland	University of Oulu
Selenia lunularia	MM03764	LEFIC292-10	HM872136	BOLD:AAB1585	Finland	University of Oulu
Selenia lunularia	MM03387	LEFIC087-10	HM871955	BOLD:AAB1585	Finland	University of Oulu
Selenia lunularia	TLMF Lep 07840	PHLAV021-12	KM573559	BOLD:ABY7097	Austria	inatura, Dornbirn

Selenia tetralunaria	MM10853	LEFIF206-10	HM874900	BOLD:AAB7329	Finland	University of Oulu
Selenia tetralunaria	MM08421	LEFIE166-10	HM873912	BOLD:AAB7329	Finland	University of Oulu
Selenia tetralunaria	MM01360	LEFIA314-10	HM386657	BOLD:AAB7329	Finland	University of Oulu
Selenia tetralunaria	TLMF Lep 07843	PHLAV024-12	KM572655	BOLD:AAB7329	Austria	inatura, Dornbirn
Selenia tetralunaria	MM01353	LEFIA307-10	HM386650	BOLD:AAB7329	Finland	University of Oulu
Selenia tetralunaria	MM01266	LEFIA234-10	HM870819	BOLD:AAB7329	Finland	University of Oulu
Semioscopis avellanella	MM18129	LEFIK554-10	JF854146	BOLD:AAH9809	Finland	University of Oulu
Semioscopis avellanella	TLMF Lep 07959	PHLAV140-12	KM572132	BOLD:AAH9809	Austria	inatura, Dornbirn
Semioscopis avellanella	MM01135	LEFIA161-10	HM396507	BOLD:AAH9809	Finland	University of Oulu
Semioscopis avellanella	MM01136	LEFIA162-10	HM396508	BOLD:AAH9809	Finland	University of Oulu
Setema cereola	MM17452	LEFIJ827-10	JF853847	BOLD:AAJ7543	Finland	University of Oulu
Setema cereola	MM19914	LEFII264-11	KM573695	BOLD:AAJ7543	Finland	Research Collection of E. Laasonen
Setema cereola	MM21031	LEFIJ1171-11	KM573488	BOLD:AAJ7543	Finland	University of Oulu
Setema cereola	TLMF Lep 06120	PHLSA665-11	KM573222	BOLD:AAJ7543	Austria	Tiroler Landesmuseum Ferdinandeum
Setema cereola	MM15929	LEFIJ329-10	JF853548	BOLD:AAJ7543	Finland	University of Oulu
Setema cereola	TLMF Lep 00294	PHLAA254-09	HM381355	BOLD:AAJ7543	Austria	Tiroler Landesmuseum Ferdinandeum
Setina irrorella	MM17197	LEFIJ572-10	JF853685	BOLD:ABZ4612	Finland	University of Oulu
Setina irrorella	MM05904	LEFID143-10	HM872953	BOLD:ABZ4612	Finland	University of Oulu
Setina irrorella	MM10589	LEFIF133-10	HM874831	BOLD:ABZ4612	Finland	University of Oulu
Setina irrorella	TLMF Lep 00306	PHLAA266-09	HM425803	BOLD:ACF4655	Austria	Tiroler Landesmuseum Ferdinandeum
Setina irrorella	TLMF Lep 06119	PHLSA664-11	KM572083	BOLD:ACF4655	Austria	Tiroler Landesmuseum Ferdinandeum
Sideridis reticulata	MM01593	LEFIA511-10	KM573055	BOLD:AAD4204	Finland	University of Oulu
Sideridis reticulata	MM12607	LEFIF662-10	HM875346	BOLD:AAD4204	Finland	University of Oulu
Sideridis reticulata	MM01592	LEFIA510-10	KM573017	BOLD:AAD4204	Finland	University of Oulu
Sideridis reticulata	TLMF Lep 07581	PHLAG902-12	KM572345	BOLD:AAD4204	Austria	inatura, Dornbirn
Sideridis rivularis	MM01619	LEFIA535-10	KM573095	BOLD:AAC8418	Finland	University of Oulu
Sideridis rivularis	MM14101	LEFIG212-10	HM875892	BOLD:AAC8418	Finland	University of Oulu
Sideridis rivularis	MM04758	LEFIC714-10	HM872535	BOLD:AAC8418	Finland	University of Oulu
Sideridis rivularis	TLMF Lep 08186	PHLAV367-12	KM573181	BOLD:AAC8418	Austria	inatura, Dornbirn
Sideridis rivularis	TLMF Lep 08807	PHLAI312-13	KM572765	BOLD:AAC8418	Austria	Tiroler Landesmuseum Ferdinandeum
Siona lineata	MM01272	LEFIA239-10	HM386583	BOLD:AAC1903	Finland	University of Oulu

Siona lineata	MM04731	LEFIC704-10	HM872525	BOLD:AAC1903	Finland	University of Oulu
Siona lineata	TLMF Lep 09876	PHLAW079-13	KM572787	BOLD:AAC1903	Austria	Tiroler Landesmuseum Ferdinandeum
Siona lineata	MM01273	LEFIA240-10	HM386584	BOLD:AAC1903	Finland	University of Oulu
Sitochroa verticalis	TLMF Lep 08005	PHLAV186-12	KM572428	BOLD:AAB9443	Austria	inatura, Dornbirn
Sitochroa verticalis	MM01870	LEFIA715-10	HM386859	BOLD:AAB9443	Finland	University of Oulu
Sitochroa verticalis	MM01871	LEFIA716-10	HM386860	BOLD:AAB9443	Finland	University of Oulu
Sitochroa verticalis	MM12934	LEFIF747-10	HM875431	BOLD:AAB9443	Finland	University of Oulu
Sorhagenia janiszewskae	MM02331	LEFIA1366-10	GU828682	BOLD:AAF4360	Finland	University of Oulu
Sorhagenia janiszewskae	MM02333	LEFIB609-10	HM871488	BOLD:AAF4360	Finland	University of Oulu
Sorhagenia janiszewskae	MM02332	LEFIB608-10	HM871487	BOLD:AAF4360	Finland	University of Oulu
Sorhagenia janiszewskae	TLMF Lep 09190	PHLAI628-13	KM573161	BOLD:AAF4360	Austria	Tiroler Landesmuseum Ferdinandeum
Sorhagenia janiszewskae	MM02330	LEFIA1365-10	GU828681	BOLD:AAF4360	Finland	University of Oulu
Sorhagenia rhamniella	TLMF Lep 08260	PHLAH441-12	KM572701	BOLD:AAD3133	Austria	inatura, Dornbirn
Sorhagenia rhamniella	MM09736	LEFIA859-10	HM386997	BOLD:AAD3133	Finland	University of Oulu
Sorhagenia rhamniella	TLMF Lep 08064	PHLAV245-12	KM572344	BOLD:AAD3133	Austria	inatura, Dornbirn
Spargania luctuata	MM08455	LEFIE178-10	HM873924	BOLD:AAA2806	Finland	University of Oulu
Spargania luctuata	MM01391	LEFIA344-10	HM386687	BOLD:AAA2806	Finland	University of Oulu
Spargania luctuata	TLMF Lep 07567	PHLAG888-12	KM572393	BOLD:AAA2806	Austria	inatura, Dornbirn
Spargania luctuata	MM01392	LEFIA345-10	HM386688	BOLD:AAA2806	Finland	University of Oulu
Spatalistis bifasciana	MM11009	LEFIF229-10	HM874922	BOLD:AAF4512	Finland	University of Oulu
Spatalistis bifasciana	MM15664	LEFIG800-10	HM876452	BOLD:AAF4512	Finland	University of Oulu
Spatalistis bifasciana	TLMF Lep 09957	PHLAW160-13	KM572135	BOLD:AAF4512	Austria	inatura, Dornbirn
Sphinx ligustri	MM14214	LEFIG274-10	HM875953	BOLD:AAB6107	Finland	University of Oulu
Sphinx ligustri	MM06633	LEFID617-10	HM873382	BOLD:AAB6107	Finland	University of Oulu
Sphinx ligustri	TLMF Lep 07820	PHLAV001-12	KM572333	BOLD:AAB6107	Austria	inatura, Dornbirn
Sphinx pinastri	MM00979	LEFIA066-10	HM396413	BOLD:AAA8863	Finland	University of Oulu
Sphinx pinastri	TLMF Lep 07821	PHLAV002-12	KM572768	BOLD:AAA8863	Austria	inatura, Dornbirn
Sphinx pinastri	MM00978	LEFIA065-10	HM396412	BOLD:AAA8863	Finland	University of Oulu
Sphinx pinastri	MM13861	LEFIA933-10	HM387067	BOLD:AAA8863	Finland	University of Oulu
Spilonota ocellana	MM03620	LEFIC223-10	HM872067	BOLD:ABZ4399	Finland	University of Oulu
Spilonota ocellana	MM03473	LEFIC132-10	HM871978	BOLD:ABZ4399	Finland	University of Oulu

Spilonota ocellana	MM09821	LEFIE727-10	HM874447	BOLD:ABZ4399	Finland	University of Oulu
Spilonota ocellana	MM05985	LEFID190-10	HM872995	BOLD:ABZ4399	Finland	University of Oulu
Spilonota ocellana	TLMF Lep 08266	PHLAH447-12	KM572120	BOLD:ABZ4399	Austria	inatura, Dornbirn
Spilonota ocellana	MM13241	LEFIF866-10	HM875548	BOLD:ABZ4399	Finland	University of Oulu
Spilonota ocellana	MM09512	LEFIE600-10	HM874323	BOLD:ABZ4399	Finland	University of Oulu
Spilonota ocellana	MM09718	LEFIE709-10	HM874430	BOLD:ABZ4399	Finland	University of Oulu
Spilonota ocellana	MM02155	LEFIB542-10	HM871427	BOLD:ABZ4399	Finland	University of Oulu
Spilonota ocellana	MM05007	LEFIC823-10	HM872642	BOLD:ABZ4399	Finland	University of Oulu
Spilonota ocellana	MM03558	LEFIC197-10	HM872041	BOLD:ABZ4399	Finland	University of Oulu
Spilonota ocellana	TLMF Lep 10036	LEATA429-13	KM572340	BOLD:ABZ4399	Austria	inatura, Dornbirn
Spilosoma lubricipeda	MM04659	LEFIC685-10	HM872506	BOLD:AAB2233	Finland	University of Oulu
Spilosoma lubricipeda	MM01039	LEFIA114-10	HM396461	BOLD:AAB2233	Finland	University of Oulu
Spilosoma lubricipeda	MM01040	LEFIA115-10	HM396462	BOLD:AAB2233	Finland	University of Oulu
Spilosoma lubricipeda	TLMF Lep 06159	PHLSA704-11	KM573000	BOLD:AAB2233	Austria	Tiroler Landesmuseum Ferdinandeum
Spilosoma lubricipeda	MM00858	LEFIA1369-10	KM573469	BOLD:AAB2233	Finland	University of Oulu
Spilosoma lutea	MM05171	LEFIC884-10	HM872702	BOLD:AAC0134	Finland	University of Oulu
Spilosoma lutea	MM01035	LEFIA112-10	HM396459	BOLD:AAC0134	Finland	University of Oulu
Spilosoma lutea	TLMF Lep 08280	PHLAH461-12	KM573420	BOLD:AAC0134	Austria	inatura, Dornbirn
Spilosoma lutea	MM01036	LEFIA113-10	HM396460	BOLD:AAC0134	Finland	University of Oulu
Spodoptera exigua	MM21046	LEFIJ1186-11	KM573052	BOLD:AAA6644	Finland	University of Oulu
Spodoptera exigua	MM15939	LEFIJ339-10	JF853552	BOLD:AAA6644	Finland	University of Oulu
Spodoptera exigua	MM15940	LEFIJ340-10	KM572984	BOLD:AAA6644	Finland	University of Oulu
Spodoptera exigua	TLMF Lep 06197	PHLSA742-11	KM573047	BOLD:AAA6644	Austria	Tiroler Landesmuseum Ferdinandeum
Standfussiana lucernea	MM17221	LEFIJ596-10	JF853696	BOLD:AAE9254	Finland	University of Oulu
Standfussiana lucernea	MM12509	LEFIF620-10	HM875304	BOLD:AAE9254	Finland	University of Oulu
Standfussiana lucernea	TLMF Lep 06098	PHLSA643-11	KM573437	BOLD:AAE9254	Austria	Tiroler Landesmuseum Ferdinandeum
Standfussiana lucernea	MM17500	LEFIJ875-10	JX034653	BOLD:AAE9254	Finland	University of Oulu
Standfussiana lucernea	MM17499	LEFIJ874-10	JX034667	BOLD:AAE9254	Finland	University of Oulu
Stathmopoda pedella	TLMF Lep 08229	PHLAH410-12	KM573207	BOLD:AAD4282	Austria	inatura, Dornbirn
Stathmopoda pedella	MM02351	LEFIA1371-10	KM572795	BOLD:AAD4282	Finland	University of Oulu
Stathmopoda pedella	MM13872	LEFIA944-10	HM387077	BOLD:AAD4282	Finland	University of Oulu

Stathmopoda pedella	CNCLEP00020448	LNEL252-06	KM572242	BOLD:AAD4282	Finland	Canadian National Collection of Insects, Arachnids and Nematodes
Stathmopoda pedella	MM02178	LEFIA1370-10	JF818790	BOLD:AAD4282	Finland	University of Oulu
Stauropus fagi	MM04544	LEFIC613-10	HM872434	BOLD:AAD0646	Finland	University of Oulu
Stauropus fagi	MM00851	LEFIB320-10	HM871220	BOLD:AAD0646	Finland	University of Oulu
Stauropus fagi	TLMF Lep 04656	PHLAE341-11	JN307423	BOLD:AAD0646	Austria	Tiroler Landesmuseum Ferdinandeum
Stauropus fagi	MM00981	LEFIA1374-10	GU828651	BOLD:AAD0646	Finland	University of Oulu
Stauropus fagi	MM08373	LEFIE139-10	HM873887	BOLD:AAD0646	Finland	University of Oulu
Stenoptilia pterodactyla	MM03503	LEFIC157-10	HM872003	BOLD:AAC7533	Finland	University of Oulu
Stenoptilia pterodactyla	MM03098	LEFIB943-10	HM871820	BOLD:AAC7533	Finland	University of Oulu
Stenoptilia pterodactyla	TLMF Lep 08404	PHLAH585-12	KM572666	BOLD:AAC7533	Austria	inatura, Dornbirn
Stenoptilia pterodactyla	MM02253	LEFIB577-10	HM871458	BOLD:AAC7533	Finland	University of Oulu
Stenoptilia pterodactyla	MM03683	LEFIC260-10	HM872104	BOLD:AAC7533	Finland	University of Oulu
Sterrhopterix standfussi	MM14127	LEFIG227-10	HM875907	BOLD:AAI0085	Finland	University of Oulu
Sterrhopterix standfussi	TLMF Lep 09505	LEATA088-13	KM572290	BOLD:AAI0085	Austria	Tiroler Landesmuseum Ferdinandeum
Sterrhopterix standfussi	MM08501	LEFIE186-10	HM873931	BOLD:AAI0085	Finland	University of Oulu
Sterrhopterix standfussi	MM08502	LEFIE187-10	HM873932	BOLD:AAI0085	Finland	University of Oulu
Sterrhopterix standfussi	MM17518	LEFIJ893-10	JF853884	BOLD:AAI0085	Finland	University of Oulu
Stigmella dryadella	TLMF Lep 02871	PHLAC836-10	KM573110	BOLD:AAV8362	Austria	Tiroler Landesmuseum Ferdinandeum
Stigmella dryadella	MM23205	COLFF175-13	KM572791	BOLD:ACD1518	Finland	University of Oulu, Zoological Museum
Stigmella floslactella	MM09606	LEFIE648-10	HM874371	BOLD:AAF3363	Finland	University of Oulu
Stigmella floslactella	MM09605	LEFIE647-10	HM874370	BOLD:AAF3363	Finland	University of Oulu
Stigmella floslactella	TLMF Lep 08300	PHLAH481-12	KM573563	BOLD:AAF3363	Austria	inatura, Dornbirn
Stigmella floslactella	MM07007	LEFID871-10	HM873628	BOLD:AAF3363	Finland	University of Oulu
Stigmella myrtillella	MM15174	LEFIJ290-10	JF853527	BOLD:AAJ4189	Finland	University of Oulu
Stigmella myrtillella	MM06330	LEFID407-10	HM873203	BOLD:AAJ4189	Finland	University of Oulu
Stigmella myrtillella	MM00935	LEFIB369-10	HM871268	BOLD:AAJ4189	Finland	University of Oulu
Stigmella myrtillella	MM00936	LEFIB370-10	HM871269	BOLD:AAJ4189	Finland	University of Oulu
Stigmella myrtillella	TLMF Lep 09216	PHLAI654-13	KM573243	BOLD:AAJ4189	Austria	Tiroler Landesmuseum Ferdinandeum
Stigmella myrtillella	MM21040	LEFIJ1180-11	KM572041	BOLD:AAJ4189	Finland	University of Oulu

Stigmella pretiosa	TLMF Lep 02872	PHLAC837-10	JF860337	BOLD:AAI0004	Austria	Tiroler Landesmuseum Ferdinandeum
Stigmella pretiosa	MM09643	LEFIE676-10	HM874398	BOLD:AAI0004	Finland	University of Oulu
Stigmella pretiosa	MM22761	LEFIJ1493-12	KM572342	BOLD:AAI0004	Finland	University of Oulu
Stigmella pretiosa	MM09642	LEFIE675-10	HM874397	BOLD:AAI0004	Finland	University of Oulu
Swammerdamia compunctella	TLMF Lep 09143	PHLAI581-13	KM572210	BOLD:AAH9903	Austria	Tiroler Landesmuseum Ferdinandeum
Swammerdamia compunctella	MM00704	LEFIB241-10	HM871144	BOLD:AAH9903	Finland	University of Oulu
Swammerdamia compunctella	MM02616	LEFIB759-10	HM871636	BOLD:AAH9903	Finland	University of Oulu
Swammerdamia compunctella	MM03148	LEFIB966-10	HQ570279	BOLD:AAH9903	Finland	University of Oulu
Synanthedon formicaeformis	TLMF Lep 09871	PHLAW074-13	KM573130	BOLD:AAC4578	Austria	Tiroler Landesmuseum Ferdinandeum
Synanthedon formicaeformis	MM18789	LEFIL491-10	JF854619	BOLD:ABZ6492	Finland	University of Oulu
Synanthedon formicaeformis	MM00673	LEFIB219-10	HM871123	BOLD:ABZ6492	Finland	University of Oulu
Synanthedon formicaeformis	MM08402	LEFIE156-10	HM873903	BOLD:ABZ6492	Finland	University of Oulu
Synanthedon formicaeformis	MM08399	LEFIE153-10	HM873900	BOLD:ABZ6492	Finland	University of Oulu
Synanthedon formicaeformis	MM15760	LEFIG896-10	HM876541	BOLD:ABZ6492	Finland	University of Oulu
Synanthedon formicaeformis	MM18794	LEFIL496-10	JF854623	BOLD:ABZ6492	Finland	University of Oulu
Synanthedon spheciformis	MM17877	LEFIK302-10	JF853956	BOLD:AAE2758	Finland	University of Oulu
Synanthedon spheciformis	TLMF Lep 09872	PHLAW075-13	KM573390	BOLD:AAE2758	Austria	Tiroler Landesmuseum Ferdinandeum
Synanthedon spheciformis	MM15758	LEFIG894-10	HM876539	BOLD:AAE2758	Finland	University of Oulu
Synanthedon spheciformis	MM18790	LEFIL492-10	JF854620	BOLD:AAE2758	Finland	University of Oulu
Synanthedon spheciformis	MM17407	LEFIJ782-10	JF853821	BOLD:AAE2758	Finland	University of Oulu

Synanthedon spheciformis	MM17408	LEFIJ783-10	KM572833	BOLD:AAE2758	Finland	University of Oulu
Synanthedon spheciformis	MM17878	LEFIK303-10	JF853957	BOLD:AAE2758	Finland	University of Oulu
Synanthedon tipuliformis	MM00117	LEFIB029-10	HM870940	BOLD:AAC1840	Finland	University of Oulu
Synanthedon tipuliformis	MM17410	LEFIJ785-10	JF853822	BOLD:AAC1840	Finland	University of Oulu
Synanthedon tipuliformis	TLMF Lep 09870	PHLAW073-13	KM572234	BOLD:AAC1840	Austria	Tiroler Landesmuseum Ferdinandeum
Syncopacma cinctella	MM03081	LEFIB935-10	HM871812	BOLD:AAD7223	Finland	University of Oulu
Syncopacma cinctella	MM21180	LEFIJ1320-11	KM573206	BOLD:AAD7223	Finland	University of Oulu
Syncopacma cinctella	MM00750	LEFIB270-10	HM871172	BOLD:AAD7223	Finland	University of Oulu
Syncopacma cinctella	TLMF Lep 09948	PHLAW151-13	KM573303	BOLD:AAD7223	Austria	inatura, Dornbirn
Syncopacma cinctella	MM02313	LEFIB599-10	HM871478	BOLD:AAD7223	Finland	University of Oulu
Syncopacma cinctella	MM02314	LEFIB600-10	HM871479	BOLD:AAD7223	Finland	University of Oulu
Syncopacma cinctella	MM12005	LEFIF471-10	HM875156	BOLD:AAD7223	Finland	University of Oulu
Syncopacma cinctella	MM09716	LEFIA841-10	HM386981	BOLD:AAD7223	Finland	University of Oulu
Syncopacma cinctella	MM13528	LEFIF955-10	HM875636	BOLD:AAD7223	Finland	University of Oulu
Syncopacma cinctella	MM09503	LEFIE594-10	HM874317	BOLD:AAD7223	Finland	University of Oulu
Syncopacma cinctella	MM13874	LEFIA946-10	HQ570260	BOLD:AAD7223	Finland	University of Oulu
Syncopacma cinctella	TLMF Lep 08039	PHLAV220-12	KM571943	BOLD:AAD7223	Austria	inatura, Dornbirn
Syncopacma sangiella	MM21150	LEFIJ1290-11	KM572916	BOLD:AAE8758	Finland	University of Oulu
Syncopacma sangiella	MM21182	LEFIJ1322-11	KM573531	BOLD:AAE8758	Finland	University of Oulu
Syncopacma sangiella	MM04807	LEFIC731-10	HM872552	BOLD:AAE8758	Finland	University of Oulu
Syncopacma sangiella	TLMF Lep 08400	PHLAH581-12	KM572624	BOLD:AAE8758	Austria	inatura, Dornbirn
Syncopacma sangiella	MM17727	LEFIK152-10	KF809181	BOLD:AAE8758	Finland	University of Oulu
Syncopacma taeniolella	TLMF Lep 08040	PHLAV221-12	KM573534	BOLD:AAE8756	Austria	inatura, Dornbirn
Syncopacma taeniolella	MM09506	LEFIE597-10	HM874320	BOLD:AAE8756	Finland	University of Oulu
Syncopacma taeniolella	MM09505	LEFIE596-10	HM874319	BOLD:AAE8756	Finland	University of Oulu
Syncopacma taeniolella	MM09507	LEFIE598-10	HM874321	BOLD:AAE8756	Finland	University of Oulu
Syncopacma taeniolella	MM09501	LEFIE592-10	HM874315	BOLD:AAE8756	Finland	University of Oulu
Syncopacma taeniolella	MM09504	LEFIE595-10	HM874318	BOLD:AAE8756	Finland	University of Oulu
Syndemis musculana	CNCLEP00020418	LNEL222-06	KM572368	BOLD:ABY7128	Finland	Canadian National Collection of Insects, Arachnids and Nematodes
Syndemis musculana	MM00524	LEFIB144-10	HM871049	BOLD:ABY7128	Finland	University of Oulu

Syndemis musculana	TLMF Lep 07924	PHLAV105-12	KM572550	BOLD:ABY7128	Austria	inatura, Dornbirn
Syndemis musculana	MM02045	LEFIB480-10	HM871377	BOLD:ABY7128	Finland	University of Oulu
Syndemis musculana	MM05292	LEFIC935-10	HM872750	BOLD:ABY7128	Finland	University of Oulu
Syngrapha interrogationis	TLMF Lep 08499	PHLAH680-12	KM572190	BOLD:AAB3481	Austria	inatura, Dornbirn
Syngrapha interrogationis	MM02752	LEFIB816-10	HM871693	BOLD:AAB3481	Finland	University of Oulu
Syngrapha interrogationis	MM07253	LEFID903-10	HM873653	BOLD:AAB3481	Finland	University of Oulu
Syngrapha interrogationis	TLMF Lep 01138	PHLAB338-10	HQ968500	BOLD:AAB3481	Austria	Tiroler Landesmuseum Ferdinandeum
Syngrapha interrogationis	MM04885	LEFIC763-10	HM872582	BOLD:AAB3481	Finland	University of Oulu
Syngrapha interrogationis	TLMF Lep 00297	PHLAA257-09	HM425794	BOLD:AAB3481	Austria	Tiroler Landesmuseum Ferdinandeum
Taleporia tubulosa	MM13263	LEFIF874-10	HM875556	BOLD:AAC2838	Finland	University of Oulu
Taleporia tubulosa	MM09951	LEFIE779-10	HM874498	BOLD:AAC2838	Finland	University of Oulu
Taleporia tubulosa	MM02176	LEFIB549-10	HM871432	BOLD:AAC2838	Finland	University of Oulu
Taleporia tubulosa	MM10085	LEFIE853-10	HM874571	BOLD:AAC2838	Finland	University of Oulu
Taleporia tubulosa	MM02175	LEFIA1384-10	GU828677	BOLD:AAC2838	Finland	University of Oulu
Taleporia tubulosa	TLMF Lep 08242	PHLAH423-12	KM573138	BOLD:AAC2838	Austria	inatura, Dornbirn
Taleporia tubulosa	MM14274	LEFIG310-10	HM875989	BOLD:AAC2838	Finland	University of Oulu
Taleporia tubulosa	MM05290	LEFIC933-10	HM872748	BOLD:AAC2838	Finland	University of Oulu
Taleporia tubulosa	MM09952	LEFIE780-10	HM874499	BOLD:AAC2838	Finland	University of Oulu
Teleiodes flavimaculella	MM10491	LEFIF051-10	HM874762	BOLD:AAH9847	Finland	University of Oulu
Teleiodes flavimaculella	MM09223	LEFIE457-10	HM874181	BOLD:AAH9847	Finland	University of Oulu
Teleiodes flavimaculella	TLMF Lep 07946	PHLAV127-12	KM572602	BOLD:AAH9847	Austria	inatura, Dornbirn
Teleiodes flavimaculella	MM17574	LEFIJ949-10	KM373632	BOLD:AAH9847	Finland	University of Oulu
Teleiodes luculella	MM18229	LEFIK654-10	JF854224	BOLD:AAD2632	Finland	University of Oulu
Teleiodes luculella	MM09775	LEFIA898-10	HM387034	BOLD:AAD2632	Finland	University of Oulu
Teleiodes luculella	TLMF Lep 08050	PHLAV231-12	KM573103	BOLD:AAD2632	Austria	inatura, Dornbirn
Teleiodes luculella	MM09792	LEFIA914-10	HM387050	BOLD:AAD2632	Finland	University of Oulu
Teleiodes vulgella	MM10490	LEFIF050-10	HM874761	BOLD:AAE9855	Finland	University of Oulu
Teleiodes vulgella	MM13610	LEFIG001-10	HM875681	BOLD:AAE9855	Finland	University of Oulu
Teleiodes vulgella	MM12470	LEFIF599-10	HM875283	BOLD:AAE9855	Finland	University of Oulu
Teleiodes vulgella	TLMF Lep 08053	PHLAV234-12	KM572638	BOLD:AAE9855	Austria	inatura, Dornbirn
Teleiodes wagae	TLMF Lep 08061	PHLAV242-12	KM573687	BOLD:AAV7088	Austria	inatura, Dornbirn

Teleiodes wagae	MM17710	LEFIK135-10	JN270945	BOLD:AAV7088	Finland	University of Oulu
Teleiodes wagae	MM17711	LEFIK136-10	JN270946	BOLD:AAV7088	Finland	University of Oulu
Tethea or	MM01169	LEFIA182-10	HM396527	BOLD:AAC9855	Finland	University of Oulu
Tethea or	MM01168	LEFIA181-10	HM396526	BOLD:AAC9855	Finland	University of Oulu
Tethea or	MM00647	LEFIB202-10	HM871106	BOLD:AAC9855	Finland	University of Oulu
Tethea or	TLMF Lep 04654	PHLAE339-11	JN286226	BOLD:AAC9856	Austria	Tiroler Landesmuseum Ferdinandeum
Tetheella fluctuosa	TLMF Lep 12461	LEATC479-13	KM572188	BOLD:AAD3647	Austria	Tiroler Landesmuseum Ferdinandeum
Tetheella fluctuosa	MM01501	LEFIA437-10	HM386778	BOLD:AAD3647	Finland	University of Oulu
Tetheella fluctuosa	MM09790	LEFIA912-10	HM387048	BOLD:AAD3647	Finland	University of Oulu
Tetheella fluctuosa	MM01502	LEFIA438-10	HM386779	BOLD:AAD3647	Finland	University of Oulu
Thera cognata	MM11612	LEFIF374-10	HM875059	BOLD:AAB9686	Finland	University of Oulu
Thera cognata	MM11613	LEFIF375-10	HM875060	BOLD:AAB9686	Finland	University of Oulu
Thera cognata	MM06805	LEFID744-10	HM873501	BOLD:AAB9686	Finland	University of Oulu
Thera cognata	TLMF Lep 06162	PHLSA707-11	KM573586	BOLD:AAB9686	Austria	Tiroler Landesmuseum Ferdinandeum
Thera juniperata	TLMF Lep 08786	PHLAI291-13	KM573381	BOLD:AAA3914	Austria	Tiroler Landesmuseum Ferdinandeum
Thera juniperata	MM15810	LEFIG946-10	HM876586	BOLD:AAA3914	Finland	University of Oulu
Thera juniperata	MM02885	LEFIB860-10	HM871737	BOLD:AAA3914	Finland	University of Oulu
Thera juniperata	TLMF Lep 06799	PHLAG405-12	KM573478	BOLD:AAA3914	Austria	Tiroler Landesmuseum Ferdinandeum
Thera juniperata	MM15809	LEFIG945-10	HM876585	BOLD:AAA3914	Finland	University of Oulu
Thera juniperata	TLMF Lep 06130	PHLSA675-11	KM573477	BOLD:AAA3914	Austria	Tiroler Landesmuseum Ferdinandeum
Thera obeliscata	TLMF Lep 08168	PHLAV349-12	KM573296	BOLD:AAA7521	Austria	inatura, Dornbirn
Thera obeliscata	MM00787	LEFIJ029-10	KM573069	BOLD:AAA7521	Finland	University of Oulu
Thera obeliscata	MM07906	LEFIJ110-10	KM572733	BOLD:AAA7521	Finland	University of Oulu
Thera obeliscata	MM07905	LEFIJ109-10	KM572015	BOLD:AAA7521	Finland	University of Oulu
Thera obeliscata	MM00788	LEFIJ030-10	KM573314	BOLD:AAA7521	Finland	University of Oulu
Thera obeliscata	MM18439	LEFIK864-10	JF854324	BOLD:AAA7521	Finland	University of Oulu
Thera obeliscata	MM18440	LEFIK865-10	JF854325	BOLD:AAA7521	Finland	University of Oulu
Thera obeliscata	MM04559	LEFIJ065-10	KM573164	BOLD:AAA7521	Finland	University of Oulu
Thera obeliscata	MM01356	LEFIA310-10	HM386653	BOLD:AAA7521	Finland	University of Oulu
Thera obeliscata	MM14602	LEFIG508-10	HM876182	BOLD:AAA7521	Finland	University of Oulu
Thera obeliscata	MM01357	LEFIA311-10	HM386654	BOLD:AAA7521	Finland	University of Oulu

Thera obeliscata	MM12818	LEFIJ199-10	KM573058	BOLD:AAA7522	Finland	University of Oulu
Thera variata	MM17988	LEFIK413-10	JF854042	BOLD:AAA7521	Finland	University of Oulu
Thera variata	TLMF Lep 06161	PHLSA706-11	KM573087	BOLD:AAA7521	Austria	Tiroler Landesmuseum Ferdinandeum
Thera variata	MM03322	LEFIC052-10	HM871921	BOLD:AAA7521	Finland	University of Oulu
Thera variata	TLMF Lep 06185	PHLSA730-11	KM573615	BOLD:AAA7521	Austria	Tiroler Landesmuseum Ferdinandeum
Thera variata	MM17369	LEFIJ744-10	JF853799	BOLD:AAA7521	Finland	University of Oulu
Thera variata	MM18438	LEFIK863-10	JF854323	BOLD:AAA7521	Finland	University of Oulu
Thera variata	MM00718	LEFIB249-10	HM871152	BOLD:AAA7521	Finland	University of Oulu
Thera variata	MM18437	LEFIK862-10	KM572235	BOLD:AAA7521	Finland	University of Oulu
Thera variata	TLMF Lep 08161	PHLAV342-12	KM573422	BOLD:AAA7521	Austria	inatura, Dornbirn
Thera variata	MM18436	LEFIK861-10	JF854322	BOLD:AAA7521	Finland	University of Oulu
Thera variata	MM18435	LEFIK860-10	JF854321	BOLD:AAA7521	Finland	University of Oulu
Thera variata	MM17989	LEFIK414-10	JF854043	BOLD:AAA7521	Finland	University of Oulu
Thera variata	MM17990	LEFIK415-10	JF854044	BOLD:AAA7521	Finland	University of Oulu
Thiotricha subocellea	MM09334	LEFIE510-10	HM874234	BOLD:AAD7105	Finland	University of Oulu
Thiotricha subocellea	MM05683	LEFID074-10	HM872885	BOLD:AAD7105	Finland	University of Oulu
Thiotricha subocellea	MM09333	LEFIA1390-10	JF818795	BOLD:AAD7105	Finland	University of Oulu
Thiotricha subocellea	MM17233	LEFIJ608-10	JF853708	BOLD:AAD7105	Finland	University of Oulu
Thiotricha subocellea	TLMF Lep 00901	PHLAB101-10	HM381473	BOLD:AAD7105	Austria	Tiroler Landesmuseum Ferdinandeum
Tholera cespitis	TLMF Lep 12547	LEATC565-13	KM573302	BOLD:AAD2239	Austria	Tiroler Landesmuseum Ferdinandeum
Tholera cespitis	MM04754	LEFIC712-10	HM872533	BOLD:AAD2239	Finland	University of Oulu
Tholera cespitis	MM07351	LEFID917-10	HM873667	BOLD:AAD2239	Finland	University of Oulu
Tholera cespitis	MM01741	LEFIA626-10	HM870875	BOLD:ACE5542	Finland	University of Oulu
Tholera decimalis	MM04795	LEFIC723-10	HM872544	BOLD:AAC9682	Finland	University of Oulu
Tholera decimalis	MM01728	LEFIA614-10	HM870863	BOLD:AAC9682	Finland	University of Oulu
Tholera decimalis	TLMF Lep 08758	PHLAI263-13	KM571968	BOLD:AAC9682	Austria	Tiroler Landesmuseum Ferdinandeum
Tholera decimalis	MM01729	LEFIA615-10	HM870864	BOLD:AAC9682	Finland	University of Oulu
Thyatira batis	MM03707	LEFIC274-10	HM872118	BOLD:AAC4671	Finland	University of Oulu
Thyatira batis	TLMF Lep 07890	PHLAV071-12	KM572821	BOLD:AAC4671	Austria	inatura, Dornbirn
Thyatira batis	MM00027	LEFIA1393-10	GU828580	BOLD:AAC4671	Finland	University of Oulu
Thyatira batis	MM01144	LEFIA168-10	HM396514	BOLD:AAC4671	Finland	University of Oulu

Thyatira batis	MM01145	LEFIA169-10	HQ963148	BOLD:ABY7313	Finland	University of Oulu
Thymelicus lineola	TLMF Lep 09830	PHLAW033-13	KM573383	BOLD:AAA6759	Austria	Tiroler Landesmuseum Ferdinandeum
Thymelicus lineola	MM03333	LEFIA1394-10	KM573575	BOLD:AAA6760	Finland	University of Oulu
Thymelicus lineola	MM17368	LEFIJ743-10	JF853798	BOLD:AAA6760	Finland	University of Oulu
Thymelicus lineola	MM14671	LEFIG536-10	HM876209	BOLD:AAA6760	Finland	University of Oulu
Tiliacea aurago	TLMF Lep 06193	PHLSA738-11	KM572776	BOLD:AAD4895	Austria	Tiroler Landesmuseum Ferdinandeum
Tiliacea aurago	MM03179	LEFIB985-10	HM871857	BOLD:AAD4895	Finland	University of Oulu
Tiliacea aurago	MM18042	LEFIK467-10	JF854080	BOLD:AAD4895	Finland	University of Oulu
Tiliacea aurago	MM04792	LEFIC721-10	HM872542	BOLD:AAD4895	Finland	University of Oulu
Tiliacea citrago	TLMF Lep 08762	PHLAI267-13	KM572085	BOLD:AAF7502	Austria	Tiroler Landesmuseum Ferdinandeum
Tiliacea citrago	MM06120	LEFID255-10	HM873053	BOLD:AAF7502	Finland	University of Oulu
Tiliacea citrago	MM15868	LEFIH004-10	HM876640	BOLD:AAF7502	Finland	University of Oulu
Tiliacea citrago	MM18043	LEFIK468-10	KM572530	BOLD:AAF7502	Finland	University of Oulu
Timandra comae	MM04838	LEFIC737-10	HM872558	BOLD:AAB0828	Finland	University of Oulu
Timandra comae	MM04806	LEFIC730-10	HM872551	BOLD:AAB0828	Finland	University of Oulu
Timandra comae	MM04645	LEFIC677-10	HM872498	BOLD:AAB0828	Finland	University of Oulu
Timandra comae	TLMF Lep 08545	PHLAH726-12	KF807555	BOLD:AAB0828	Austria	inatura, Dornbirn
Timandra comae	MM12899	LEFIF741-10	HM875425	BOLD:AAB0828	Finland	University of Oulu
Timandra comae	MM01496	LEFIA433-10	HM386774	BOLD:AAB0828	Finland	University of Oulu
Timandra comae	MM12785	LEFIF722-10	HM875406	BOLD:AAB0828	Finland	University of Oulu
Timandra comae	MM12784	LEFIF721-10	HM875405	BOLD:AAB0828	Finland	University of Oulu
Timandra comae	MM01361	LEFIA315-10	HM386658	BOLD:AAB0828	Finland	University of Oulu
Tinea pellionella	TLMF Lep 09906	PHLAW109-13	KM572101	BOLD:AAB9203	Austria	Tiroler Landesmuseum Ferdinandeum
Tinea pellionella	TLMF Lep 08474	PHLAH655-12	KM572229	BOLD:AAB9203	Austria	inatura, Dornbirn
Tinea pellionella	TLMF Lep 09909	PHLAW112-13	KM573263	BOLD:AAB9203	Austria	Tiroler Landesmuseum Ferdinandeum
Tinea pellionella	MM06611	LEFIA1398-10	KF808797	BOLD:AAB9203	Finland	University of Oulu
Tinea pellionella	MM10377	LEFIE966-10	HM874683	BOLD:AAB9203	Finland	University of Oulu
Tinea pellionella	MM06612	LEFID600-10	HM873365	BOLD:AAB9203	Finland	University of Oulu
Tinea semifulvella	MM02338	LEFIB610-10	HM871489	BOLD:AAC3847	Finland	University of Oulu
Tinea semifulvella	MM03823	LEFIC319-10	HM872163	BOLD:AAC3847	Finland	University of Oulu
Tinea semifulvella	TLMF Lep 07968	PHLAV149-12	KM572260	BOLD:AAC3847	Austria	inatura, Dornbirn

Tinea semifulvella	MM13522	LEFIF952-10	HM875633	BOLD:AAC3847	Finland	University of Oulu
Tinea semifulvella	MM09751	LEFIA874-10	HM387012	BOLD:AAC3847	Finland	University of Oulu
Tinea trinotella	TLMF Lep 07937	PHLAV118-12	KM572921	BOLD:AAD5562	Austria	inatura, Dornbirn
Tinea trinotella	MM10309	LEFIE930-10	HM874647	BOLD:AAD5562	Finland	University of Oulu
Tinea trinotella	MM08378	LEFIE143-10	HM873891	BOLD:AAD5562	Finland	University of Oulu
Tinea trinotella	TLMF Lep 07966	PHLAV147-12	KM572337	BOLD:AAD5562	Austria	inatura, Dornbirn
Tischeria ekebladella	MM00943	LEFIA1442-10	GU828648	BOLD:AAF8247	Finland	University of Oulu
Tischeria ekebladella	TLMF Lep 08062	PHLAV243-12	KM572888	BOLD:AAF8247	Austria	inatura, Dornbirn
Tischeria ekebladella	MM19896	LEFII246-11	KM573066	BOLD:AAF8247	Finland	Research Collection of E. Laasonen
Tischeria ekebladella	MM00033	LEFIB014-10	HM870927	BOLD:AAF8247	Finland	University of Oulu
Tischeria ekebladella	MM05331	LEFIC959-10	HM872774	BOLD:AAF8247	Finland	University of Oulu
Tischeria ekebladella	MM00945	LEFIB373-10	HM871272	BOLD:AAF8247	Finland	University of Oulu
Tortrix viridana	MM04996	LEFIC815-10	HM872634	BOLD:AAC2506	Finland	University of Oulu
Tortrix viridana	TLMF Lep 08019	PHLAV200-12	KM572769	BOLD:AAC2506	Austria	inatura, Dornbirn
Tortrix viridana	MM00510	LEFIA1403-10	GU828644	BOLD:AAC2506	Finland	University of Oulu
Tortrix viridana	MM06724	LEFID681-10	HM873442	BOLD:AAC2506	Finland	University of Oulu
Trachea atriplicis	MM00344	LEFIB059-10	HM870968	BOLD:AAE0836	Finland	University of Oulu
Trachea atriplicis	TLMF Lep 08508	PHLAH689-12	KM572208	BOLD:AAE0836	Austria	inatura, Dornbirn
Trachea atriplicis	MM01161	LEFIA177-10	HM396522	BOLD:AAE0836	Finland	University of Oulu
Trachea atriplicis	MM00855	LEFIB321-10	HM871221	BOLD:AAE0836	Finland	University of Oulu
Trachea atriplicis	TLMF Lep 08281	PHLAH462-12	KM573105	BOLD:AAE0836	Austria	inatura, Dornbirn
Trichiura crataegi	MM13871	LEFIA943-10	HM387076	BOLD:AAB4489	Finland	University of Oulu
Trichiura crataegi	TLMF Lep 08491	PHLAH672-12	KM572357	BOLD:AAB4489	Austria	inatura, Dornbirn
Trichiura crataegi	MM00836	LEFIB312-10	HM871212	BOLD:AAB4489	Finland	University of Oulu
Trichiura crataegi	MM02797	LEFIB835-10	HM871712	BOLD:AAB4489	Finland	University of Oulu
Trichiura crataegi	MM08292	LEFIE107-10	HM873855	BOLD:AAB4489	Finland	University of Oulu
Trichiura crataegi	MM07389	LEFID925-10	HM873675	BOLD:AAB4489	Finland	University of Oulu
Trichiura crataegi	MM12574	LEFIF650-10	HM875334	BOLD:AAB4489	Finland	University of Oulu
Trichiura crataegi	MM13870	LEFIA942-10	HM387075	BOLD:AAB4489	Finland	University of Oulu
Trichiura crataegi	MM01052	LEFIA124-10	HM396471	BOLD:AAB4489	Finland	University of Oulu
Trichiura crataegi	MM01051	LEFIA123-10	HM396470	BOLD:AAB4489	Finland	University of Oulu

Trichiura crataegi	MM03616	LEFIC220-10	HM872064	BOLD:AAB4489	Finland	University of Oulu
Trichiura crataegi	MM08104	LEFIE032-10	HM873781	BOLD:AAB4489	Finland	University of Oulu
Trichopteryx carpinata	TLMF Lep 04668	PHLAE353-11	JN279572	BOLD:AAC7472	Austria	Tiroler Landesmuseum Ferdinandeum
Trichopteryx carpinata	MM08387	LEFIE148-10	HM873896	BOLD:AAC7472	Finland	University of Oulu
Trichopteryx carpinata	MM01142	LEFIA166-10	HM396512	BOLD:AAC7472	Finland	University of Oulu
Trichopteryx carpinata	MM04633	LEFIA1406-10	GU828704	BOLD:AAC7472	Finland	University of Oulu
Trichopteryx carpinata	MM01143	LEFIA167-10	HM396513	BOLD:AAC7472	Finland	University of Oulu
Trichopteryx polycommata	MM07856	LEFID954-10	HM873704	BOLD:AAC5988	Finland	University of Oulu
Trichopteryx polycommata	TLMF Lep 08859	PHLAI364-13	KM572815	BOLD:AAC5988	Austria	Tiroler Landesmuseum Ferdinandeum
Trichopteryx polycommata	MM04024	LEFIC451-10	HM872285	BOLD:AAC5988	Finland	University of Oulu
Trichopteryx polycommata	MM00448	LEFIA045-10	HM396394	BOLD:AAC5988	Finland	University of Oulu
Triodia sylvina	MM08007	LEFID978-10	HM873728	BOLD:AAD0500	Finland	University of Oulu
Triodia sylvina	MM02372	LEFIB626-10	HM871505	BOLD:AAD0500	Finland	University of Oulu
Triodia sylvina	TLMF Lep 12464	LEATC482-13	KM573349	BOLD:AAD0500	Austria	Tiroler Landesmuseum Ferdinandeum
Triodia sylvina	MM04769	LEFIC717-10	HM872538	BOLD:AAD0500	Finland	University of Oulu
Triodia sylvina	MM04983	LEFIC809-10	HM872628	BOLD:AAD0500	Finland	University of Oulu
Triphosa dubitata	MM15815	LEFIG951-10	HM876591	BOLD:AAD3995	Finland	University of Oulu
Triphosa dubitata	MM18466	LEFIK891-10	JF854342	BOLD:AAD3995	Finland	University of Oulu
Triphosa dubitata	MM04218	LEFIC510-10	HM872334	BOLD:AAD3995	Finland	University of Oulu
Triphosa dubitata	TLMF Lep 06121	PHLSA666-11	KM573057	BOLD:AAD3995	Austria	Tiroler Landesmuseum Ferdinandeum
Trisateles emortualis	TLMF Lep 07978	PHLAV159-12	KM572989	BOLD:AAC2587	Austria	inatura, Dornbirn
Trisateles emortualis	MM01513	LEFIA449-10	HM386790	BOLD:AAC2587	Finland	University of Oulu
Trisateles emortualis	MM01419	LEFIA363-10	HM386705	BOLD:AAC2587	Finland	University of Oulu
Trisateles emortualis	MM04877	LEFIA1408-10	GU828707	BOLD:AAC2587	Finland	University of Oulu
Trisateles emortualis	MM01514	LEFIA450-10	HM386791	BOLD:AAC2587	Finland	University of Oulu
Tyria jacobaeae	TLMF Lep 04129	PHLAD764-11	JN262974	BOLD:AAB5189	Austria	Tiroler Landesmuseum Ferdinandeum
Tyria jacobaeae	TLMF Lep 04128	PHLAD763-11	JN262973	BOLD:AAB5189	Austria	Tiroler Landesmuseum Ferdinandeum
Tyria jacobaeae	MM10597	LEFIF138-10	HM874833	BOLD:AAB5189	Finland	University of Oulu

Udea accolalis	BC MTD Lep 00765	IBLPC659-11	JF852284	BOLD:AAJ7913	Austria	Tiroler Landesmuseum Ferdinandeum
Udea accolalis	MM17851	LEFIK276-10	KM573062	BOLD:AAJ7913	Finland	University of Oulu
Udea accolalis	MM19946	LEFII296-11	KM572968	BOLD:AAJ7913	Finland	Research Collection of J. Itaemies
Udea accolalis	TLMF Lep 07396	PHLAG717-12	KM572059	BOLD:AAJ7913	Austria	inatura, Dornbirn
Udea accolalis	MM21134	LEFIJ1274-11	KM572705	BOLD:AAJ7913	Finland	University of Oulu
Udea decrepitalis	TLMF Lep 07571	PHLAG892-12	KM572637	BOLD:ABY5633	Austria	inatura, Dornbirn
Udea decrepitalis	MM01862	LEFIA707-10	HM386852	BOLD:ABY5633	Finland	University of Oulu
Udea decrepitalis	MM01863	LEFIA708-10	HM386853	BOLD:ABY5633	Finland	University of Oulu
Udea decrepitalis	MM02941	LEFIB873-10	HM871750	BOLD:ABY5633	Finland	University of Oulu
Udea inquinatalis	MM14586	LEFIG502-10	HQ570404	BOLD:AAB9747	Finland	University of Oulu
Udea inquinatalis	MM04139	LEFIA785-10	HM386926	BOLD:AAB9747	Finland	University of Oulu
Udea inquinatalis	MM04133	LEFIA779-10	HM386920	BOLD:AAB9747	Finland	University of Oulu
Udea inquinatalis	TLMF Lep 02834	PHLAC799-10	JF860316	BOLD:AAB9747	Austria	Tiroler Landesmuseum Ferdinandeum
Udea nebulalis	MM06605	LEFID595-10	HM873360	BOLD:AAD5509	Finland	University of Oulu
Udea nebulalis	MM08327	LEFIE122-10	HM873870	BOLD:AAD5509	Finland	University of Oulu
Udea nebulalis	TLMF Lep 03624	PHLAD449-11	JN284790	BOLD:AAD5509	Austria	Tiroler Landesmuseum Ferdinandeum
Udea nebulalis	MM13868	LEFIA940-10	HM387073	BOLD:AAD5509	Finland	University of Oulu
Udea nebulalis	TLMF Lep 00914	PHLAB114-10	HM381486	BOLD:AAD5509	Austria	Tiroler Landesmuseum Ferdinandeum
Udea prunalis	TLMF Lep 08568	PHLAH749-12	KM572057	BOLD:AAC2028	Austria	inatura, Dornbirn
Udea prunalis	MM01861	LEFIA706-10	HM386851	BOLD:AAC2028	Finland	University of Oulu
Udea prunalis	MM01860	LEFIA705-10	HM386850	BOLD:AAC2028	Finland	University of Oulu
Udea prunalis	MM02944	LEFIB874-10	HM871751	BOLD:AAC2028	Finland	University of Oulu
Vanessa atalanta	TLMF Lep 09851	PHLAW054-13	KM573621	BOLD:AAA8638	Austria	Tiroler Landesmuseum Ferdinandeum
Vanessa atalanta	MM00953	LEFIB379-10	HM871278	BOLD:AAA8638	Finland	University of Oulu
Vanessa atalanta	MM14193	LEFIG261-10	HM875940	BOLD:AAA8638	Finland	University of Oulu
Vanessa atalanta	MM17151	LEFIJ526-10	JF853645	BOLD:AAA8638	Finland	University of Oulu
Vanessa cardui	TLMF Lep 09852	PHLAW055-13	KM573216	BOLD:AAA5337	Austria	Tiroler Landesmuseum Ferdinandeum
Vanessa cardui	MM13949	LEFIG133-10	HM875813	BOLD:AAA5337	Finland	University of Oulu
Vanessa cardui	MM17150	LEFIJ525-10	JF853644	BOLD:AAA5337	Finland	University of Oulu
Vanessa cardui	MM00952	LEFIB378-10	HM871277	BOLD:AAA5337	Finland	University of Oulu
Venusia blomeri	MM14282	LEFIG315-10	HM875994	BOLD:AAC9722	Finland	University of Oulu

Venusia blomeri	MM01755	LEFIA640-10	HM870889	BOLD:AAC9722	Finland	University of Oulu
Venusia blomeri	MM01313	LEFIA273-10	HM386617	BOLD:AAC9722	Finland	University of Oulu
Venusia blomeri	MM01312	LEFIA272-10	HM386616	BOLD:AAC9722	Finland	University of Oulu
Venusia blomeri	TLMF Lep 08196	PHLAV377-12	KM573382	BOLD:AAC9722	Austria	inatura, Dornbirn
Venusia blomeri	MM01457	LEFIA398-10	HM386740	BOLD:AAC9722	Finland	University of Oulu
Venusia cambrica	MM07864	LEFID955-10	HM873705	BOLD:AAA1586	Finland	University of Oulu
Venusia cambrica	TLMF Lep 06179	PHLSA724-11	KM573688	BOLD:AAA1586	Austria	Tiroler Landesmuseum Ferdinandeum
Venusia cambrica	MM10483	LEFIF043-10	HM874754	BOLD:AAA1586	Finland	University of Oulu
Venusia cambrica	MM15832	LEFIG968-10	HM876608	BOLD:AAA1586	Finland	University of Oulu
Venusia cambrica	MM10484	LEFIF044-10	HM874755	BOLD:AAA1586	Finland	University of Oulu
Vitula biviella	MM17820	LEFIK245-10	KM573662	BOLD:AAH7769	Finland	University of Oulu
Vitula biviella	TLMF Lep 08478	PHLAH659-12	KM573247	BOLD:AAH7769	Austria	inatura, Dornbirn
Vitula biviella	MM21115	LEFIJ1255-11	KM572674	BOLD:AAH7769	Finland	University of Oulu
Xanthia icteritia	MM08182	LEFIE060-10	HM873807	BOLD:AAC9311	Finland	University of Oulu
Xanthia icteritia	MM23222	COLFF948-13	KM572385	BOLD:AAC9311	Finland	University of Oulu
Xanthia icteritia	MM01707	LEFIA595-10	HM870844	BOLD:AAC9311	Finland	University of Oulu
Xanthia icteritia	TLMF Lep 08795	PHLAI300-13	KM572460	BOLD:AAC9311	Austria	Tiroler Landesmuseum Ferdinandeum
Xanthia icteritia	MM01706	LEFIA594-10	HM870843	BOLD:AAC9311	Finland	University of Oulu
Xanthia togata	MM00814	LEFIB300-10	HM871201	BOLD:AAC9312	Finland	University of Oulu
Xanthia togata	TLMF Lep 08768	PHLAI273-13	KM573439	BOLD:AAC9312	Austria	Tiroler Landesmuseum Ferdinandeum
Xanthia togata	MM04794	LEFIC722-10	HM872543	BOLD:AAC9312	Finland	University of Oulu
Xanthia togata	MM08183	LEFIE061-10	HM873808	BOLD:AAC9312	Finland	University of Oulu
Xanthorhoe biriviata	MM15800	LEFIG936-10	HM876578	BOLD:AAD1977	Finland	University of Oulu
Xanthorhoe biriviata	MM10463	LEFIF024-10	HM874738	BOLD:AAD1977	Finland	University of Oulu
Xanthorhoe biriviata	MM15801	LEFIG937-10	HM876579	BOLD:AAD1977	Finland	University of Oulu
Xanthorhoe biriviata	TLMF Lep 06200	PHLSA745-11	KM572867	BOLD:AAD1977	Austria	Tiroler Landesmuseum Ferdinandeum
Xanthorhoe biriviata	TLMF Lep 08171	PHLAV352-12	KM572073	BOLD:AAD1977	Austria	inatura, Dornbirn
Xanthorhoe decoloraria	TLMF Lep 05854	PHLSA399-11	KM572578	BOLD:AAA5318	Austria	Tiroler Landesmuseum Ferdinandeum
Xanthorhoe decoloraria	MM06361	LEFID431-10	HQ570327	BOLD:AAA5318	Finland	University of Oulu
Xanthorhoe decoloraria	MM04106	LEFIA752-10	HM386896	BOLD:AAA5318	Finland	University of Oulu
Xanthorhoe decoloraria	MM02857	LEFIB853-10	HM871730	BOLD:AAA5318	Finland	University of Oulu

Xanthorhoe designata	MM02858	LEFIB854-10	HM871731	BOLD:AAA7001	Finland	University of Oulu
Xanthorhoe designata	MM10118	LEFIJ153-10	JF853466	BOLD:AAA7001	Finland	University of Oulu
Xanthorhoe designata	MM03701	LEFIC269-10	HM872113	BOLD:AAA7001	Finland	University of Oulu
Xanthorhoe designata	MM07831	LEFIJ107-10	KM573326	BOLD:ABZ0894	Finland	University of Oulu
Xanthorhoe designata	MM12336	LEFIJ187-10	KM572329	BOLD:ABZ0894	Finland	University of Oulu
Xanthorhoe designata	MM08341	LEFIJ117-10	KM572641	BOLD:ABZ0894	Finland	University of Oulu
Xanthorhoe designata	MM07830	LEFIJ106-10	KM573571	BOLD:ABZ0894	Finland	University of Oulu
Xanthorhoe designata	MM06028	LEFIJ088-10	KM572950	BOLD:ABZ0894	Finland	University of Oulu
Xanthorhoe designata	TLMF Lep 07896	PHLAV077-12	KM573257	BOLD:ABZ0894	Austria	inatura, Dornbirn
Xanthorhoe designata	MM06362	LEFID432-10	HQ570328	BOLD:ABZ0894	Finland	University of Oulu
Xanthorhoe ferrugata	MM12873	LEFIJ203-10	KM573220	BOLD:ACF4785	Finland	University of Oulu
Xanthorhoe ferrugata	MM17340	LEFIJ715-10	KM572718	BOLD:ACF4785	Finland	University of Oulu
Xanthorhoe ferrugata	MM03872	LEFIJ060-10	KM572639	BOLD:ACF4785	Finland	University of Oulu
Xanthorhoe ferrugata	TLMF Lep 07897	PHLAV078-12	KM572918	BOLD:ACF4785	Austria	inatura, Dornbirn
Xanthorhoe ferrugata	TLMF Lep 07971	PHLAV152-12	KM573702	BOLD:ACF4785	Austria	inatura, Dornbirn
Xanthorhoe ferrugata	MM03801	LEFIJ059-10	JF853421	BOLD:ACF4785	Finland	University of Oulu
Xanthorhoe ferrugata	MM04839	LEFIJ066-10	KM572503	BOLD:ACF4785	Finland	University of Oulu
Xanthorhoe ferrugata	MM18421	LEFIK846-10	JN279406	BOLD:ACF4785	Finland	University of Oulu
Xanthorhoe ferrugata	MM18422	LEFIK847-10	JN279407	BOLD:ACF4785	Finland	University of Oulu
Xanthorhoe ferrugata	MM03514	LEFIC164-10	HM872010	BOLD:ACF4785	Finland	University of Oulu
Xanthorhoe ferrugata	MM11618	LEFIJ176-10	KM573490	BOLD:ACF4785	Finland	University of Oulu
Xanthorhoe ferrugata	MM09677	LEFIJ134-10	JF853452	BOLD:ACF4785	Finland	University of Oulu
Xanthorhoe ferrugata	MM01396	LEFIA349-10	HM386692	BOLD:ACF4785	Finland	University of Oulu
Xanthorhoe ferrugata	MM12839	LEFIJ202-10	KM571966	BOLD:ACF4785	Finland	University of Oulu
Xanthorhoe ferrugata	MM12838	LEFIJ201-10	KM572915	BOLD:ACF4785	Finland	University of Oulu
Xanthorhoe ferrugata	MM01765	LEFIA648-10	HM870897	BOLD:ACF4785	Finland	University of Oulu
Xanthorhoe ferrugata	MM13943	LEFIG129-10	HM875809	BOLD:ACF5678	Finland	University of Oulu
Xanthorhoe ferrugata	MM01440	LEFIA383-10	HM386725	BOLD:ACF5678	Finland	University of Oulu
Xanthorhoe ferrugata	MM12337	LEFIJ188-10	KM573466	BOLD:ACF5678	Finland	University of Oulu
Xanthorhoe fluctuata	MM00088	LEFIA017-10	HM396367	BOLD:AAA6836	Finland	University of Oulu
Xanthorhoe fluctuata	MM01369	LEFIA322-10	HM386665	BOLD:AAA6836	Finland	University of Oulu

Xanthorhoe fluctuata	TLMF Lep 08779	PHLAI284-13	KM573578	BOLD:AAA6836	Austria	Tiroler Landesmuseum Ferdinandeum
Xanthorhoe fluctuata	MM01326	LEFIA286-10	HM386629	BOLD:AAA6836	Finland	University of Oulu
Xanthorhoe montanata	TLMF Lep 08557	PHLAH738-12	KM573407	BOLD:AAB2524	Austria	inatura, Dornbirn
Xanthorhoe montanata	MM18420	LEFIK845-10	JN279405	BOLD:AAB2524	Finland	University of Oulu
Xanthorhoe montanata	MM02846	LEFIB850-10	HM871727	BOLD:AAB2524	Finland	University of Oulu
Xanthorhoe montanata	MM08453	LEFIE177-10	HM873923	BOLD:AAB2524	Finland	University of Oulu
Xanthorhoe quadrifasiata	TLMF Lep 08552	PHLAH733-12	KM572749	BOLD:AAC9330	Austria	inatura, Dornbirn
Xanthorhoe quadrifasiata	MM23189	COLFF456-13	KM573698	BOLD:AAC9330	Finland	University of Oulu, Zoological Museum
Xanthorhoe quadrifasiata	MM01474	LEFIA412-10	HM386754	BOLD:AAC9330	Finland	University of Oulu
Xanthorhoe quadrifasiata	MM01473	LEFIA411-10	HM386753	BOLD:AAC9330	Finland	University of Oulu
Xanthorhoe quadrifasiata	MM02826	LEFIB845-10	HM871722	BOLD:AAC9330	Finland	University of Oulu
Xanthorhoe spadicearia	MM01345	LEFIA300-10	HM386643	BOLD:AAB7980	Finland	University of Oulu
Xanthorhoe spadicearia	MM04108	LEFIA754-10	HM386898	BOLD:AAB7980	Finland	University of Oulu
Xanthorhoe spadicearia	MM01346	LEFIA301-10	HM386644	BOLD:AAB7980	Finland	University of Oulu
Xanthorhoe spadicearia	TLMF Lep 10008	LEATA401-13	KM573691	BOLD:AAB7980	Austria	inatura, Dornbirn
Xestia alpicola	LN-BD0177	LENOA177-11	KM572820	BOLD:ABZ1718	Finland	Research Collection of Bernard Dardenne
Xestia alpicola	MM18749	LEFIL451-10	JF854600	BOLD:ABZ1718	Finland	University of Oulu
Xestia alpicola	MM08067	LEFIE015-10	HM873764	BOLD:ABZ1718	Finland	University of Oulu
Xestia alpicola	TLMF Lep 06101	PHLSA646-11	KM572383	BOLD:ABZ1718	Austria	Tiroler Landesmuseum Ferdinandeum
Xestia alpicola	MM08068	LEFIE016-10	HM873765	BOLD:ABZ1718	Finland	University of Oulu
Xestia alpicola	MM12102	LEFIF507-10	HM875192	BOLD:ABZ1718	Finland	University of Oulu
Xestia ashworthii	MM21061	LEFIJ1201-11	KM573332	BOLD:ABY8432	Finland	University of Oulu
Xestia ashworthii	MM17509	LEFIJ884-10	KM572980	BOLD:ABY8432	Finland	University of Oulu
Xestia ashworthii	MM21062	LEFIJ1202-11	KM573484	BOLD:ABY8432	Finland	University of Oulu
Xestia ashworthii	TLMF Lep 00313	PHLAA273-09	HM425809	BOLD:ABY8432	Austria	Tiroler Landesmuseum Ferdinandeum
Xestia ashworthii	MM17510	LEFIJ885-10	KM572731	BOLD:ABY8432	Finland	University of Oulu
Xestia baja	MM01659	LEFIA565-10	KM572038	BOLD:AAA2590	Finland	University of Oulu
Xestia baja	MM00828	LEFIB307-10	HM871208	BOLD:AAA2590	Finland	University of Oulu
Xestia baja	TLMF Lep 08549	PHLAH730-12	KM572148	BOLD:AAA2590	Austria	inatura, Dornbirn

Xestia baja	MM01658	LEFIA564-10	KM571944	BOLD:AAA2590	Finland	University of Oulu
Xestia baja	TLMF Lep 08793	PHLAI298-13	KM572604	BOLD:AAA2590	Austria	Tiroler Landesmuseum Ferdinandeum
Xestia c-nigrum	MM07709	LEFID939-10	HM873689	BOLD:AAA2144	Finland	University of Oulu
Xestia c-nigrum	MM06870	LEFID794-10	HM873551	BOLD:AAA2144	Finland	University of Oulu
Xestia c-nigrum	MM04640	LEFIC673-10	HM872494	BOLD:AAA2144	Finland	University of Oulu
Xestia c-nigrum	TLMF Lep 07836	PHLAV017-12	KM572166	BOLD:AAA2144	Austria	inatura, Dornbirn
Xestia c-nigrum	MM17284	LEFIJ659-10	KM572339	BOLD:AAA2144	Finland	University of Oulu
Xestia collina	MM01635	LEFIA548-10	KM573240	BOLD:AAE1853	Finland	University of Oulu
Xestia collina	TLMF Lep 06177	PHLSA722-11	KM573600	BOLD:AAE1853	Austria	Tiroler Landesmuseum Ferdinandeum
Xestia collina	MM01634	LEFIA547-10	KM572361	BOLD:AAE1853	Finland	University of Oulu
Xestia collina	MM07403	LEFID931-10	HM873681	BOLD:AAE1853	Finland	University of Oulu
Xestia speciosa	MM18760	LEFIL462-10	JF854608	BOLD:ACE4666	Finland	University of Oulu
Xestia speciosa	MM00840	LEFIB313-10	HM871213	BOLD:ACE4666	Finland	University of Oulu
Xestia speciosa	MM02739	LEFIB809-10	HM871686	BOLD:ACE4666	Finland	University of Oulu
Xestia speciosa	MM08072	LEFIE017-10	HM873766	BOLD:ACE4666	Finland	University of Oulu
Xestia speciosa	MM08073	LEFIE018-10	HM873767	BOLD:ACE4666	Finland	University of Oulu
Xestia speciosa	MM06113	LEFID249-10	HM873048	BOLD:ACE4666	Finland	University of Oulu
Xestia speciosa	MM18762	LEFIL464-10	JF854610	BOLD:ACE4666	Finland	University of Oulu
Xestia speciosa	TLMF Lep 04431	PHLAE211-11	JN284164	BOLD:ACF2698	Austria	Tiroler Landesmuseum Ferdinandeum
Xestia speciosa	TLMF Lep 04432	PHLAE212-11	JN284165	BOLD:ACF2698	Austria	Tiroler Landesmuseum Ferdinandeum
Xestia speciosa	TLMF Lep 00182	PHLAA142-09	GU689197	BOLD:ACF2698	Austria	Tiroler Landesmuseum Ferdinandeum
Xestia speciosa	TLMF Lep 00183	PHLAA143-09	GU689151	BOLD:ACF2698	Austria	Tiroler Landesmuseum Ferdinandeum
Xestia speciosa	TLMF Lep 08488	PHLAH669-12	KM572274	BOLD:ACF2698	Austria	inatura, Dornbirn
Xestia triangulum	MM18833	LEFIL535-10	KM573101	BOLD:AAB8843	Finland	University of Oulu
Xestia triangulum	MM01599	LEFIA516-10	KM572049	BOLD:AAB8843	Finland	University of Oulu
Xestia triangulum	TLMF Lep 08155	PHLAV336-12	KM573492	BOLD:AAB8843	Austria	inatura, Dornbirn
Xestia triangulum	MM05110	LEFIC861-10	HM872679	BOLD:AAB8843	Finland	University of Oulu
Xestia triangulum	MM01600	LEFIA517-10	KM573120	BOLD:AAB8843	Finland	University of Oulu
Xestia xanthographa	TLMF Lep 12549	LEATC567-13	KM573609	BOLD:AAA6806	Austria	Tiroler Landesmuseum Ferdinandeum
Xylena vetusta	TLMF Lep 05820	PHLAF650-11	KM573260	BOLD:AAC2612	Austria	Tiroler Landesmuseum Ferdinandeum
Xylena vetusta	MM01560	LEFIA483-10	KM571981	BOLD:AAC2612	Finland	University of Oulu

Xylena vetusta	MM04503	LEFIC586-10	HM872407	BOLD:AAC2612	Finland	University of Oulu
Xylena vetusta	MM01559	LEFIA482-10	KM573308	BOLD:AAC2612	Finland	University of Oulu
Yponomeuta evonymella	MM02179	LEFIB550-10	HM871433	BOLD:AAA7740	Finland	University of Oulu
Yponomeuta evonymella	MM00306	LEFIA1427-10	GU828628	BOLD:AAA7740	Finland	University of Oulu
Yponomeuta evonymella	TLMF Lep 08089	PHLAV270-12	KM573230	BOLD:AAA7740	Austria	inatura, Dornbirn
Yponomeuta evonymella	MM00307	LEFIB039-10	HM870948	BOLD:AAA7740	Finland	University of Oulu
Yponomeuta evonymella	MM05233	LEFIC907-10	HM872724	BOLD:AAA7740	Finland	University of Oulu
Yponomeuta evonymella	MM13368	LEFIF927-10	HM875609	BOLD:AAA7740	Finland	University of Oulu
Yponomeuta evonymella	MM08126	LEFIE040-10	HM873789	BOLD:AAA7740	Finland	University of Oulu
Yponomeuta malinellus	TLMF Lep 08473	PHLAH654-12	KM572901	BOLD:AAA7740	Austria	inatura, Dornbirn
Yponomeuta malinellus	TLMF Lep 09888	PHLAW091-13	KM572261	BOLD:AAA7740	Austria	Tiroler Landesmuseum Ferdinandeum
Yponomeuta malinellus	MM11851	LEFIF428-10	HM875113	BOLD:AAA7740	Finland	University of Oulu
Yponomeuta malinellus	MM02418	LEFIB651-10	HM871530	BOLD:AAA7740	Finland	University of Oulu
Yponomeuta malinellus	MM15532	LEFIG668-10	HM876329	BOLD:AAA7740	Finland	University of Oulu
Yponomeuta malinellus	MM02419	LEFIB652-10	HM871531	BOLD:AAA7740	Finland	University of Oulu
Yponomeuta malinellus	MM09458	LEFIE569-10	HM874292	BOLD:AAA7740	Finland	University of Oulu
Yponomeuta plumbella	TLMF Lep 08030	PHLAV211-12	KM573583	BOLD:ACL2673	Austria	inatura, Dornbirn
Yponomeuta plumbella	MM17242	LEFIJ617-10	JF853717	BOLD:ACL2673	Finland	University of Oulu
Yponomeuta sedella	TLMF Lep 12473	LEATC491-13	KM572437	BOLD:AAE4875	Austria	Tiroler Landesmuseum Ferdinandeum
Yponomeuta sedella	MM14386	LEFIG379-10	HM876056	BOLD:AAE4875	Finland	University of Oulu
Yponomeuta sedella	MM11861	LEFIF430-10	HM875115	BOLD:AAE4875	Finland	University of Oulu
Yponomeuta sedella	MM03114	LEFIB953-10	HM871830	BOLD:AAE4875	Finland	University of Oulu
Ypsolopha falcella	MM06417	LEFID475-10	HQ963161	BOLD:ACF4757	Finland	University of Oulu
Ypsolopha falcella	TLMF Lep 08241	PHLAH422-12	KM573686	BOLD:ACF4757	Austria	inatura, Dornbirn
Ypsolopha falcella	MM05027	LEFIC831-10	HM872649	BOLD:ACF4757	Finland	University of Oulu
Ypsolopha falcella	MM17333	LEFIJ708-10	JF853784	BOLD:ACF4757	Finland	University of Oulu
Ypsolopha nemorella	TLMF Lep 08405	PHLAH586-12	KM572237	BOLD:AAE4873	Austria	inatura, Dornbirn
Ypsolopha nemorella	MM02185	LEFIB553-10	HM871436	BOLD:AAE4873	Finland	University of Oulu
Ypsolopha nemorella	MM06416	LEFID474-10	HQ963160	BOLD:AAE4873	Finland	University of Oulu
Ypsolopha nemorella	MM06418	LEFID476-10	HM873242	BOLD:AAE4873	Finland	University of Oulu
Ypsolopha ustella	MM09709	LEFIA834-10	HM386974	BOLD:AAD0001	Finland	University of Oulu

Ypsolopha ustella	MM05314	LEFIC946-10	HM872761	BOLD:AAD0001	Finland	University of Oulu
Ypsolopha ustella	TLMF Lep 08034	PHLAV215-12	KM572925	BOLD:AAD0001	Austria	inatura, Dornbirn
Ypsolopha ustella	MM13377	LEFIF934-10	HM875616	BOLD:AAD0001	Finland	University of Oulu
Ypsolopha ustella	TLMF Lep 07960	PHLAV141-12	KM573556	BOLD:AAD0001	Austria	inatura, Dornbirn
Zeiraphera isertana	MM05011	LEFIC825-10	HM872644	BOLD:AAD1936	Finland	University of Oulu
Zeiraphera isertana	MM03618	LEFIC222-10	HM872066	BOLD:AAD1936	Finland	University of Oulu
Zeiraphera isertana	MM10364	LEFIE958-10	HM874675	BOLD:AAD1936	Finland	University of Oulu
Zeiraphera isertana	TLMF Lep 08047	PHLAV228-12	KM573524	BOLD:AAD1936	Austria	inatura, Dornbirn
Zeiraphera ratzeburgiana	TLMF Lep 08230	PHLAH411-12	KM572317	BOLD:AAD8502	Austria	inatura, Dornbirn
Zeiraphera ratzeburgiana	MM08972	LEFIE405-10	HM874129	BOLD:ACF5190	Finland	University of Oulu
Zeiraphera ratzeburgiana	BIOUG04116-B11	GMFIG612-12	KM572105	BOLD:ACF5190	Finland	Biodiversity Institute of Ontario
Zeiraphera ratzeburgiana	MM00699	LEFIB237-10	HM871140	BOLD:ACF5190	Finland	University of Oulu
Zeiraphera ratzeburgiana	MM13314	LEFIF900-10	HM875582	BOLD:ACF5190	Finland	University of Oulu
Zelleria hepariella	TLMF Lep 07400	PHLAG721-12	KM572196	BOLD:AAF6476	Austria	inatura, Dornbirn
Zelleria hepariella	MM08533	LEFIE218-10	HQ570367	BOLD:AAF6476	Finland	University of Oulu
Zelleria hepariella	MM08657	LEFIE303-10	HM874029	BOLD:AAF6476	Finland	University of Oulu
Zelleria hepariella	TLMF Lep 08202	PHLAH383-12	KM572808	BOLD:AAF6476	Austria	inatura, Dornbirn
Zelleria hepariella	MM14636	LEFIG523-10	HM876196	BOLD:AAF6476	Finland	University of Oulu
Zeuzera pyrina	TLMF Lep 08133	PHLAV314-12	KM572471	BOLD:AAD8444	Austria	inatura, Dornbirn
Zeuzera pyrina	MM18950	LEFIL652-10	KM572974	BOLD:AAD8444	Finland	University of Oulu
Zygaena exulans	MM18406	LEFIK831-10	JX034642	BOLD:AAD7474	Finland	University of Oulu
Zygaena exulans	TLMF Lep 12548	LEATC566-13	KM571990	BOLD:AAD7474	Austria	Tiroler Landesmuseum Ferdinandeum
Zygaena exulans	MM15793	LEFIG929-10	HQ570417	BOLD:AAD7474	Finland	University of Oulu
Zygaena exulans	MM15794	LEFIG930-10	HQ570418	BOLD:AAD7474	Finland	University of Oulu
Zygaena filipendulae	MM09460	LEFIE570-10	HM874293	BOLD:AAC8100	Finland	University of Oulu
Zygaena filipendulae	MM06904	LEFIA1430-10	GU828750	BOLD:AAC8100	Finland	University of Oulu
Zygaena filipendulae	TLMF Lep 08566	PHLAH747-12	KM572835	BOLD:AAC8100	Austria	inatura, Dornbirn
Zygaena filipendulae	MM09489	LEFIE584-10	HM874307	BOLD:AAC8100	Finland	University of Oulu
Zygaena filipendulae	MM06905	LEFID817-10	HM873574	BOLD:AAC8100	Finland	University of Oulu
Zygaena filipendulae	MM09461	LEFIE571-10	HM874294	BOLD:AAC8100	Finland	University of Oulu
Zygaena lonicerae	MM06769	LEFID715-10	HQ570352	BOLD:AAD7508	Finland	University of Oulu

Zygaena lonicerae	TLMF Lep 10018	LEATA411-13	KM573633	BOLD:AAD7508	Austria	inatura, Dornbirn
Zygaena lonicerae	MM18408	LEFIK833-10	KM572406	BOLD:AAD7508	Finland	University of Oulu
Zygaena lonicerae	MM06770	LEFID716-10	HQ570353	BOLD:AAD7508	Finland	University of Oulu
Zygaena viciae	MM06772	LEFID718-10	HQ570355	BOLD:AAC8126	Finland	University of Oulu
Zygaena viciae	TLMF Lep 09823	PHLAW026-13	KM572830	BOLD:AAC8126	Austria	Tiroler Landesmuseum Ferdinandeum
Zygaena viciae	MM22052	LEFIJ1381-12	KM572307	BOLD:AAC8126	Finland	University of Oulu
Zygaena viciae	MM22053	LEFIJ1382-12	KM573377	BOLD:AAC8126	Finland	University of Oulu
Zygaena viciae	MM22054	LEFIJ1383-12	KM572738	BOLD:AAC8126	Finland	University of Oulu
Zygaena viciae	MM06771	LEFID717-10	HQ570354	BOLD:AAC8126	Finland	University of Oulu
Zygaena viciae	MM18407	LEFIK832-10	JN303436	BOLD:AAU0288	Finland	University of Oulu
