# Supplementary material for: Testing DNA Barcode Performance in 1000 Species of European Lepidoptera: Large Geographic Distances Have Small Genetic Impacts
Source: PLoS One. 2014 Dec 26;9(12):e115774. doi: 10.1371/journal.pone.0115774 (PMC4277373; doi:10.1371/journal.pone.0115774)
Supplement: S2 Appendix — List of families and species, barcoded material per country, barcode gap analysis (intraspecific variation and distance to nearest neighbor), numbers of BINs and shared BINs, distribution pattern and dispersal capacity. (PDF) [file pone.0115774.s002.pdf]

Appendix S2: List of families and species, barcoded material per country, barcode gap analysis (intraspecific variation and distance to nearest neighbor), numbers of BINs and shared BINs, distribution pattern and dispersal capacity

Family	Species	nFI	nAT	Mean Intra	Max Intra	Nearest Neighbour NN	Distance to NN	n-BIN/ n-shared	distribution pattern	dispersal capacity
Adelidae	Nematopogon pilella	3	1	0,44	0,62	Nematopogon swammerdamella	7,76	1/1	fragmented	
Adelidae	Nematopogon robertella	5	1	0,9	2,78	Nematopogon pilella	7,93	2/0	continuous	
Adelidae	Nematopogon schwarziellus	3	2	0,06	0,15	Nematopogon pilella	8,64	1/1	fragmented	
Adelidae	Nematopogon swammerdamella	4	2	0,13	0,32	Nematopogon pilella	7,76	1/1	continuous	
Adelidae	Nemophora degeerella	5	1	0,23	0,49	Nemophora metallica	8,75	1/1	continuous	
Adelidae	Nemophora metallica	3	1	0,84	1,71	Cauchas fibulella	8,42	2/0	continuous	
Alucitidae	Pterotopteryx dodecadactyla	2	1	0,2	0,31	Elachista gleichenella	12,21	1/1	fragmented	
Argyresthiidae	Argyresthia albistria	4	1	0	0	Argyresthia conjugella	5,39	1/1	continuous	
Argyresthiidae	Argyresthia aurulentella	2	1	0,1	0,15	Argyresthia svenssoni	7,06	1/1	continuous	
Argyresthiidae	Argyresthia brockeella	3	1	0,08	0,16	Argyresthia goedartella	8,65	1/1	continuous	
Argyresthiidae	Argyresthia conjugella	4	4	0,95	1,71	Argyresthia albistria	5,39	2/2	continuous	
Argyresthiidae	Argyresthia glabratella	4	2	0,88	1,65	Argyresthia aurulentella	7,06	1/1	fragmented	
Argyresthiidae	Argyresthia goedartella	3	1	1,01	2,02	Argyresthia retinella	7,91	1/1	continuous	
Argyresthiidae	Argyresthia pruniella	4	2	0,3	0,62	Argyresthia sorbiella	3,3	1/1	continuous	
Argyresthiidae	Argyresthia retinella	12	1	0,7	1,72	Argyresthia conjugella	7,02	1/1	continuous	
Argyresthiidae	Argyresthia sorbiella	5	1	0	0	Argyresthia pruniella	3,3	1/1	continuous	
Argyresthiidae	Argyresthia svenssoni	8	1	0,34	1,24	Argyresthia aurulentella	7,06	2/0	disjunct	
Batrachedridae	Batrachedra pinicolella	17	1	2,03	6,9	Scopula incanata	8,61	2/1	continuous	
Batrachedridae	Batrachedra praeangusta	3	1	0	0	Batrachedra pinicolella	9,48	1/1	continuous	
Blastobasidae	Hypatopa binotella	5	1	0,63	1,11	Hypatopa inunctella	7,81	1/1	continuous	
Blastobasidae	Hypatopa inunctella	2	1	0,41	0,62	Hypatopa binotella	7,81	1/1	fragmented	
Bucculatricidae	Bucculatrix bechsteinella	4	1	0,52	1,24	Bucculatrix cidarella	7,52	1/1	continuous	poor
Bucculatricidae	Bucculatrix cidarella	5	1	1,24	3,3	Bucculatrix bechsteinella	7,52	2/0	continous	poor

Bucculatricidae	Bucculatrix demaryella	6	2	0,08	0,31	Bucculatrix bechsteinella	8,3	1/1	fragmented	poor
Bucculatricidae	Bucculatrix frangutella	3	1	0,69	1,39	Cabera pusaria	11,37	1/1	continuous	poor
Bucculatricidae	Bucculatrix nigricomella	3	1	0,41	0,62	Habrosyne pyritoides	12,27	1/1	continuous	poor
Bucculatricidae	Bucculatrix thoracella	3	1	0,05	0,15	Bucculatrix bechsteinella	8,24	1/1	continuous	poor
Chimabachidae	Diurnea fagella	1	1	0	0	Diurnea lipsiella	6,58	1/1	continuous	
Chimabachidae	Diurnea lipsiella	3	1	0	0	Diurnea fagella	6,58	1/1	continuous	
Choreutidae	Anthophila fabriciana	3	1	1,27	2,5	Xestia c-nigrum	12,24	2/1	continuous	
Coleophoridae	Coleophora ahenella	3	1	0,63	1,29	Coleophora fuscocuprella	5,24	1/1	continuous	poor
Coleophoridae	Coleophora albidella	8	1	0,14	0,46	Coleophora betulella	3,79	1/1	continuous	poor
Coleophoridae	Coleophora alticolella	9	5	0,35	0,93	Coleophora taeniipennella	5,35	1/1	continuous	poor
Coleophoridae	Coleophora betulella	4	1	0,18	0,31	Coleophora albidella	3,79	1/1	continuous	poor
Coleophoridae	Coleophora caespititiella	8	1	0,12	0,34	Coleophora sternipennella	5,59	1/1	continuous	poor
Coleophoridae	Coleophora flavipennella	5	4	0,47	1,71	Coleophora serratella	4,49	1/1	continuous	poor
Coleophoridae	Coleophora fuscocuprella	2	1	0	0	Coleophora ahenella	5,24	1/1	continuous	poor
Coleophoridae	Coleophora gryphipennella	4	1	0,07	0,17	Coleophora flavipennella	6,84	1/1	continuous	poor
Coleophoridae	Coleophora kuehnella	3	1	0,09	0,2	Coleophora betulella	5,52	1/1	continuous	poor
Coleophoridae	Coleophora lusciniaepennella	16	1	0,06	0,55	Coleophora ahenella	5,61	1/1	continuous	poor
Coleophoridae	Coleophora mayrella	3	2	0,28	0,46	Coleophora flavipennella	8,41	1/1	continuous	poor
Coleophoridae	Coleophora milvipennis	6	1	0,28	0,62	Coleophora gryphipennella	8,75	1/1	continuous	poor
Coleophoridae	Coleophora orbitella	5	1	0,16	0,31	Coleophora ahenella	5,28	1/1	continuous	poor
Coleophoridae	Coleophora serratella	13	1	0,33	0,77	Coleophora flavipennella	4,49	1/1	continuous	poor
Coleophoridae	Coleophora sternipennella	11	2	0,85	4,6	Coleophora caespititiella	5,59	2/1	continuous	poor
Coleophoridae	Coleophora taeniipennella	7	1	0,04	0,18	Coleophora alticolella	5,35	1/1	continuous	poor
Coleophoridae	Coleophora trigeminella	2	1	0,2	0,31	Coleophora caespititiella	9,02	1/1	fragmented	poor
Coleophoridae	Coleophora uliginosella	3	1	0,08	0,15	Coleophora flavipennella	6,89	1/1	fragmented	poor
Coleophoridae	Coleophora virgaureae	14	1	0,09	0,31	Coleophora sternipennella	6,07	1/1	continuous	poor
Cosmopterigidae	Cosmopterix lienigiella	2	1	0	0	Cosmopterix orichalcea	6,73	1/1	continuous	
Cosmopterigidae	Cosmopterix orichalcea	4	1	0,09	0,15	Cosmopterix lienigiella	6,73	1/1	continuous	
Cosmopterigidae	Limnaecia phragmitella	4	1	0	0	Achlya flavicornis	10,14	1/1	continuous	
Cosmopterigidae	Sorhagenia janiszewskae	4	1	0,41	0,83	Sorhagenia rhamniella	6,76	1/1	continuous	
Cosmopterigidae	Sorhagenia rhamniella	1	2	0	0	Sorhagenia janiszewskae	6,76	1/1	continuous	

Cossidae	Cossus cossus	3	1	1,4	2,35	Rheumaptera hastata	12,44	2/0	continuous
Cossidae	Phragmataecia castaneae	2	1	0,2	0,31	Aphelia paleana	13,02	1/1	continuous
Cossidae	Zeuzera pyrina	1	1	0	0	Limnaecia phragmitella	11,43	1/1	continuous
Crambidae	Agriphila inquinatella	4	1	0,06	0,15	Agriphila straminella	7,79	1/1	continuous
Crambidae	Agriphila straminella	5	1	1,91	3,63	Catoptria permutatellus	7,41	2/1	continuous
Crambidae	Agriphila tristella	3	1	0,08	0,15	Agriphila straminella	7,95	1/1	continuous
Crambidae	Anania coronata	3	1	0	0	Anania crocealis	8,24	1/1	continuous
Crambidae	Anania crocealis	3	1	0,08	0,18	Anania verbascalis	6,67	1/1	continuous
Crambidae	Anania funebris	3	1	0,93	1,77	Anania verbascalis	5,31	2/1	continuous
Crambidae	Anania fuscalis	4	1	0,06	0,15	Anania terrealis	6,4	1/1	continuous
Crambidae	Anania hortulata	5	1	0,18	0,47	Anania verbascalis	7,89	1/1	continuous
Crambidae	Anania lancealis	4	1	0,25	0,62	Anania crocealis	7,26	1/1	continuous
Crambidae	Anania stachydalis	3	1	0,23	0,47	Anania verbascalis	8,13	1/1	continuous
Crambidae	Anania terrealis	3	1	0,31	0,62	Anania fuscalis	6,4	1/1	continuous
Crambidae	Anania verbascalis	5	1	0,23	0,32	Anania funebris	5,31	1/1	continuous
Crambidae	Calamotropha paludella	3	1	0,7	1,01	Crambus ericella	11,2	1/1	continuous
Crambidae	Cataclysta lemnata	3	1	0,08	0,17	Conistra vaccinii	10,95	1/1	continuous
Crambidae	Catoptria falsella	3	1	0,38	0,62	Agriphila inquinatella	7,95	1/1	continuous
Crambidae	Catoptria permutatellus	4	1	0	0	Agriphila straminella	7,41	1/1	continuous
Crambidae	Catoptria verellus	3	1	0,23	0,46	Catoptria falsella	9,33	1/1	continuous
Crambidae	Chilo phragmitella	4	1	1,94	3,14	Eudonia truncicolella	9,96	2/1	continuous
Crambidae	Chrysoteuchia culmella	3	1	0,58	1,18	Crambus perlella	8,04	1/1	continuous
Crambidae	Crambus ericella	3	1	0,08	0,15	Crambus pratella	4,91	1/1	continuous
Crambidae	Crambus lathoniellus	3	1	0,16	0,32	Crambus uliginosellus	5,57	1/1	continuous
Crambidae	Crambus pascuella	3	1	0,31	0,46	Crambus pratella	5,75	1/1	continuous
Crambidae	Crambus perlella	5	1	0,24	0,62	Crambus ericella	5,8	1/1	continuous
Crambidae	Crambus pratella	3	1	0,15	0,31	Crambus ericella	4,91	1/1	continuous
Crambidae	Crambus silvella	3	1	0,15	0,31	Crambus ericella	7,98	1/1	continuous
Crambidae	Crambus uliginosellus	3	1	0,16	0,31	Crambus ericella	5,42	1/1	continuous
Crambidae	Diasemia reticularis	3	1	0,08	0,15	Parasemia plantaginis	8,75	1/1	continuous
Crambidae	Donacaula mucronella	3	1	0,47	0,79	Scoparia ancipitella	11,12	1/1	continuous

Crambidae	Elophila nymphaeata	4	1	0,25	0,62	Conistra vaccinii	11,18	1/1	continuous
Crambidae	Eudonia lacustrata	3	1	0,05	0,15	Eudonia truncicolella	4,21	1/1	continuous
Crambidae	Eudonia laetella	4	1	0,25	0,46	Eudonia lacustrata	5,09	1/1	continuous
Crambidae	Eudonia mercurella	6	1	0,77	2,85	Eudonia truncicolella	4,58	2/1	continuous
Crambidae	Eudonia murana	5	3	1,34	2,5	Eudonia mercurella	5,26	2/0	continuous
Crambidae	Eudonia sudetica	4	1	0,68	1,71	Eudonia mercurella	6,08	1/1	fragmented
Crambidae	Eudonia truncicolella	5	1	0,5	1,41	Eudonia lacustrata	4,21	1/1	continuous
Crambidae	Evergestis forficalis	4	1	0,2	0,51	Evergestis pallidata	9,14	1/1	continuous
Crambidae	Evergestis pallidata	3	1	0	0	Nomophila noctuella	8,06	1/1	continuous
Crambidae	Loxostege sticticalis	3	1	0,23	0,46	Anania crocealis	6,72	1/1	continuous
Crambidae	Metaxmeste schrankiana	4	1	0,12	0,31	Nomophila noctuella	7,89	1/1	disjunct
Crambidae	Nomophila noctuella	3	1	0	0	Metaxmeste schrankiana	7,89	1/1	migrating
Crambidae	Nymphula nitidulata	3	1	1,98	3,55	Agonopterix kaekeritziana	10,7	2/1	continuous
Crambidae	Parapoynx stratiotata	3	1	0,55	1,09	Yponomeuta malinellus	11,63	1/1	continuous
Crambidae	Paratalanta hyalinalis	3	1	0	0	Anania crocealis	7,33	1/1	continuous
Crambidae	Paratalanta pandalis	3	1	0,38	0,77	Paratalanta hyalinalis	7,34	1/1	continuous
Crambidae	Pleuroptya ruralis	6	1	0,35	0,78	Eudonia truncicolella	8,4	1/1	continuous
Crambidae	Pyrausta aerealis	1	1	3,09	3,09	Anania crocealis	8,24	2/0	continuous
Crambidae	Pyrausta aurata	4	1	0	0	Pyrausta purpuralis	9,67	1/1	continuous
Crambidae	Pyrausta cingulata	4	2	0,1	0,32	Anania verbascalis	7,05	1/1	continuous
Crambidae	Pyrausta despicata	4	1	0,06	0,15	Pyrausta cingulata	8,58	1/1	continuous
Crambidae	Pyrausta purpuralis	3	3	0,13	0,31	Pyrausta aurata	9,67	1/1	continuous
Crambidae	Scoparia ambigualis	14	2	0,3	0,62	Scoparia basistrigalis	0	1/1	continuous
Crambidae	Scoparia ancipitella	5	2	0,13	0,46	Eudonia mercurella	6,13	1/1	continuous
Crambidae	Scoparia basistrigalis	7	3	0,12	0,46	Scoparia ambigualis	0	1/1	continuous
Crambidae	Scoparia pyralella	3	1	1,27	2,5	Scoparia ambigualis	6,15	2/0	continuous
Crambidae	Scoparia subfusca	3	2	0,63	1,55	Scoparia ancipitella	11,16	2/1	continuous
Crambidae	Sitochroa verticalis	3	1	0,41	0,77	Loxostege sticticalis	7,91	1/1	continuous
Crambidae	Udea accolalis	3	2	0,09	0,16	Udea nebulalis	7,82	1/1	continuous
Crambidae	Udea decrepitalis	3	1	0,15	0,31	Udea inquinatalis	2,66	1/1	continuous
Crambidae	Udea inquinatalis	3	1	0,18	0,31	Udea decrepitalis	2,66	1/1	continuous

Crambidae	Udea nebulalis	3	2	0,25	0,46	Udea inquinatalis	5,92	1/1	fragmented	
Crambidae	Udea prunalis	3	1	0,67	0,93	Udea decrepitalis	5,44	1/1	continuous	
Depressariidae	Anchinia daphnella	5	1	0,74	1,26	Depressaria olerella	9,44	2/1	continuous	
Depressariidae	Agonopterix angelicella	14	1	0,97	4,1	Agonopterix astrantiae	3,13	3/1	continuous	
Depressariidae	Agonopterix arenella	4	1	0,06	0,16	Agonopterix kaekeritziana	4,74	1/1	continuous	
Depressariidae	Agonopterix astrantiae	3	1	0	0	Agonopterix angelicella	3,13	1/1	fragmented	
Depressariidae	Agonopterix conterminella	10	1	0,35	1,63	Agonopterix liturosa	4,9	1/1	continuous	
Depressariidae	Agonopterix heracliana	5	1	1,52	2,87	Agonopterix angelicella	4,04	2/0	continuous	
Depressariidae	Agonopterix kaekeritziana	4	1	0,31	0,64	Agonopterix angelicella	3,23	1/1	continuous	
Depressariidae	Agonopterix liturosa	3	2	0,19	0,53	Agonopterix conterminella	4,9	1/1	continuous	
Depressariidae	Agonopterix ocellana	3	1	0	0	Agonopterix angelicella	4,1	1/1	continuous	
Depressariidae	Depressaria chaerophylli	3	1	0	0	Depressaria pimpinellae	6,4	1/1	continuous	
Depressariidae	Depressaria olerella	4	1	0,5	0,77	Depressaria pulcherrimella	5,91	1/1	continuous	
Depressariidae	Depressaria pimpinellae	3	1	0,08	0,16	Depressaria radiella	3,62	1/1	continuous	
Depressariidae	Depressaria pulcherrimella	3	1	0	0	Depressaria olerella	5,91	1/1	continuous	
Depressariidae	Depressaria radiella	3	1	0	0	Depressaria pimpinellae	3,62	1/1	continuous	
Drepanidae	Achlya flavicornis	4	2	1,36	2,69	Habrosyne pyritoides	8,06	2/1	continuous	
Drepanidae	Falcaria lacertinaria	4	2	0,08	0,15	Ochropacha duplaris	9,62	1/1	continuous	
Drepanidae	Habrosyne pyritoides	3	1	0,08	0,15	Tetheella fluctuosa	6,38	1/1	continuous	
Drepanidae	Ochropacha duplaris	3	1	0,08	0,15	Tetheella fluctuosa	6,06	1/1	continuous	
Drepanidae	Tethea or	3	1	1,65	3,15	Habrosyne pyritoides	8,24	2/0	continuous	
Drepanidae	Tetheella fluctuosa	3	1	0	0	Ochropacha duplaris	6,06	1/1	continuous	
Drepanidae	Thyatira batis	4	1	0,88	2,02	Ochropacha duplaris	7,39	2/1	continuous	
Elachistidae	Elachista adscitella	12	1	0,2	0,77	Elachista subalbidella	6,74	1/1	continuous	poor
Elachistidae	Elachista albidella	13	1	1,24	2,66	Elachista occidentalis	10,67	3/1	continuous	poor
Elachistidae	Elachista albifrontella	8	1	0,17	0,31	Elachista zernyi	7,92	1/1	continuous	poor
Elachistidae	Elachista canapennella	16	2	0,02	0,16	Elachista zernyi	7,62	1/1	continuous	poor
Elachistidae	Elachista compsa	8	1	0,46	2,07	Elachista albifrontella	9	1/1	fragmented	poor
Elachistidae	Elachista freyerella	8	2	0,19	0,61	Elachista occidentalis	9,11	1/1	continuous	poor
Elachistidae	Elachista gleichenella	6	2	0,43	1,74	Elachista occidentalis	9,09	2/1	continuous	poor
Elachistidae	Elachista maculicerusella	11	1	0,7	3,79	Elachista zernyi	8,78	2/1	continuous	poor

Elachistidae	Elachista occidentalis	8	1	0,23	0,48	Spodoptera exigua	8,75	1/1	fragmented	poor
Elachistidae	Elachista subalbidella	14	2	0,17	0,47	Elachista adscitella	6,74	1/1	continuous	poor
Elachistidae	Elachista zernyi	2	1	0	0	Elachista canapennella	7,62	1/1	disjunct	poor
Elachistidae	Ethmia bipunctella	3	1	0,15	0,31	Ethmia pusiella	8,92	1/1	continuous	poor
Elachistidae	Ethmia pusiella	4	1	0	0	Ethmia bipunctella	8,92	1/1	continuous	poor
Elachistidae	Hypercallia citrinalis	4	1	0,62	1,23	Xestia triangulum	9,79	1/1	continuous	poor
Elachistidae	Perittia farinella	3	1	0,15	0,31	Elachista gleichenella	11,45	1/1	continuous	poor
Elachistidae	Semioscopis avellanella	3	1	0,08	0,15	Agonopterix angelicella	8,92	1/1	continuous	poor
Endromidae	Endromis versicolora	4	1	0,28	0,64	Parasemia plantaginis	9,44	1/1	continuous	
Epermeniidae	Epermenia chaerophyllella	4	1	0,6	0,99	Phaulernis fulviguttella	10,35	1/1	continuous	
Epermeniidae	Phaulernis fulviguttella	4	1	0,56	0,79	Epermenia chaerophyllella	10,35	1/1	continuous	
Erebidae	Arctia caja	4	1	0,52	0,93	Parasemia plantaginis	5,07	1/1	continuous	good
Erebidae	Arctornis l-nigrum	2	1	0,31	0,48	Rivula sericealis	9,64	1/1	continuous	good
Erebidae	Atolmis rubricollis	3	1	0	0	Eilema lurideola	7,59	1/1	continuous	good
Erebidae	Callimorpha dominula	2	1	0	0	Arctia caja	7,4	1/1	continuous	good
Erebidae	Calliteara pudibunda	3	1	0,16	0,32	Enargia paleacea	10,67	1/1	continuous	good
Erebidae	Catocala fraxini	3	1	0,1	0,16	Catocala nupta	4,76	1/1	continuous	good
Erebidae	Catocala nupta	3	1	0,08	0,15	Catocala fraxini	4,76	1/1	continuous	good
Erebidae	Cybosia mesomella	3	1	0,18	0,31	Setema cereola	8,43	1/1	continuous	good
Erebidae	Diacrisia sannio	3	1	0,16	0,32	Arctia caja	6,22	1/1	continuous	good
Erebidae	Dicallomera fascelina	3	1	0	0	Noctua fimbriata	9,37	1/1	continuous	good
Erebidae	Eilema complana	3	1	0,08	0,15	Setema cereola	6,26	1/1	continuous	good
Erebidae	Eilema depressa	4	1	0,12	0,31	Atolmis rubricollis	8,09	1/1	continuous	good
Erebidae	Eilema griseola	3	2	0,21	0,46	Setema cereola	7,09	1/1	continuous	good
Erebidae	Eilema lurideola	4	1	0,12	0,31	Setema cereola	5,12	1/1	continuous	good
Erebidae	Eilema sororcula	3	1	1,65	3,31	Setema cereola	6,75	2/0	continuous	good
Erebidae	Euclidia glyphica	3	1	0	0	Xestia ashworthii	8,14	1/1	continuous	good
Erebidae	Euclidia mi	4	1	0,06	0,16	Euclidia glyphica	8,9	1/1	continuous	good
Erebidae	Herminia grisealis	3	2	0,19	0,33	Herminia tarsipennalis	6,55	1/1	continuous	good
Erebidae	Herminia tarsicrinalis	2	1	0,1	0,15	Herminia grisealis	7,87	1/1	continuous	good
Erebidae	Herminia tarsipennalis	3	1	0,1	0,16	Herminia grisealis	6,55	1/1	continuous	good

Erebidae	Hypena crassalis	4	1	0,06	0,16	Hypena proboscidalis	8,28	1/1	continuous	good
Erebidae	Hypena proboscidalis	4	1	0	0	Hypena crassalis	8,28	1/1	continuous	good
Erebidae	Laspeyria flexula	3	1	0,08	0,15	Trisateles emortualis	9,29	1/1	continuous	good
Erebidae	Lithosia quadra	3	1	0	0	Brachionycha nubeculosa	8,93	1/1	continuous	good
Erebidae	Lygephila craccae	3	1	1,82	3,63	Lygephila viciae	8,13	2/0	continuous	good
Erebidae	Lygephila viciae	4	3	0	0	Lygephila craccae	8,13	1/1	continuous	good
Erebidae	Lymantria monacha	5	1	0,16	0,46	Apamea illyria	9,67	1/1	continuous	good
Erebidae	Miltochrista miniata	3	1	0	0	Elaphria venustula	9,27	1/1	continuous	good
Erebidae	Orgyia antiqua	3	1	0,41	0,77	Euclidia mi	9,63	1/1	continuous	good
Erebidae	Parasemia plantaginis	3	1	0	0	Arctia caja	5,07	1/1	continuous	good
Erebidae	Phragmatobia fuliginosa	5	1	0,13	0,31	Spilosoma lubricipeda	7,56	1/1	continuous	good
Erebidae	Phytometra viridaria	3	1	0,57	0,93	Orthosia populeti	7,89	1/1	continuous	good
Erebidae	Rhyparia purpurata	3	1	0	0	Diacrisia sannio	6,56	1/1	continuous	good
Erebidae	Rivula sericealis	8	1	0,95	2,56	Xestia ashworthii	7,51	2/1	continuous	good
Erebidae	Scoliopteryx libatrix	4	2	0,05	0,15	Xestia ashworthii	8,75	1/1	continuous	good
Erebidae	Setema cereola	4	2	0,27	0,61	Eilema lurideola	5,12	1/1	disjunct	good
Erebidae	Setina irrorella	3	2	1,76	2,85	Setema cereola	8,92	2/0	continuous	good
Erebidae	Spilosoma lubricipeda	4	1	0	0	Phragmatobia fuliginosa	7,56	1/1	continuous	good
Erebidae	Spilosoma lutea	3	1	0,08	0,15	Spilosoma lubricipeda	8,09	1/1	continuous	good
Erebidae	Trisateles emortualis	4	1	0,13	0,33	Pyrrhia umbra	8,02	1/1	continuous	good
Erebidae	Tyria jacobaeae	2	1	0,2	0,31	Callimorpha dominula	7,93	1/1	continuous	good
Eriocraniidae	Eriocrania sangii	24	1	0,37	1,08	Eriocrania semipurpurella	6,18	1/1	fragmented	
Eriocraniidae	Eriocrania semipurpurella	97	1	3,61	8,52	Eriocrania sangii	6,18	1/1	continuous	
Eriocraniidae	Heringocrania unimaculella	3	1	0,08	0,15	Eriocrania semipurpurella	10,41	1/1	continuous	
Gelechiidae	Acompsia cinerella	3	3	0,36	0,62	Bena bicolorana	9,27	1/1	continuous	
Gelechiidae	Anacampsis blattariella	7	1	0,75	2,99	Anacampsis populella	0,64	2/0	continuous	
Gelechiidae	Anacampsis populella	3	1	0,4	0,8	Anacampsis blattariella	0,64	1/1	continuous	
Gelechiidae	Anarsia lineatella	5	1	0,05	0,15	Carpatolechia alburnella	8,75	1/1	continuous	
Gelechiidae	Aproaerema anthyllidella	11	2	1,78	3,3	Syncopacma cinctella	11,05	2/1	continuous	
Gelechiidae	Argolamprotes micella	3	1	0,15	0,31	Monochroa lutulentella	10,16	1/1	continuous	
Gelechiidae	Athrips mouffetella	3	1	0,23	0,46	Prolita sexpunctella	11,55	1/1	continuous	

Gelechiidae	Brachmia blandella	3	2	0,31	0,62	Brachmia inornatella	7,73	1/1	continuous
Gelechiidae	Brachmia inornatella	4	2	0	0	Brachmia blandella	7,73	1/1	continuous
Gelechiidae	Bryotropha senectella	7	1	0,13	0,31	Bryotropha terrella	9,81	1/1	continuous
Gelechiidae	Bryotropha terrella	5	1	0,48	1,08	Bryotropha senectella	9,81	1/1	continuous
Gelechiidae	Carpatolechia alburnella	3	1	0,46	0,62	Carpatolechia fugitivella	6,07	1/1	continuous
Gelechiidae	Carpatolechia fugitivella	3	2	0,09	0,15	Carpatolechia alburnella	6,07	1/1	continuous
Gelechiidae	Caryocolum cassella	4	1	0,42	0,62	Depressaria radiella	9,71	1/1	fragmented
Gelechiidae	Chionodes electella	3	2	0,28	0,62	Chionodes fumatella	5,73	1/1	continuous
Gelechiidae	Chionodes fumatella	22	1	2,91	6,3	Chionodes electella	5,73	3/0	continuous
Gelechiidae	Chionodes holosericella	3	1	0,31	0,61	Chionodes fumatella	5,73	1/1	disjunct
Gelechiidae	Chionodes luctuella	3	2	1,4	2,44	Chionodes fumatella	6,91	2/0	fragmented
Gelechiidae	Dichomeris alacella	3	1	0,39	0,63	Dichomeris latipennella	5,42	1/1	continuous
Gelechiidae	Dichomeris latipennella	3	1	0,08	0,15	Dichomeris alacella	5,42	1/1	continuous
Gelechiidae	Dichomeris limosellus	3	1	0,54	1,08	Carpatolechia alburnella	9,97	1/1	fragmented
Gelechiidae	Eulamprotes atrella	3	1	0	0	Eulamprotes unicolorella	7,26	1/1	continuous
Gelechiidae	Eulamprotes unicolorella	6	1	0	0	Eulamprotes atrella	7,26	1/1	continuous
Gelechiidae	Exoteleia dodecella	3	1	0,05	0,15	Scoparia ancipitella	9,76	1/1	continuous
Gelechiidae	Gelechia muscosella	8	2	0,03	0,16	Gelechia sestertiella	8,94	1/1	continuous
Gelechiidae	Gelechia sabinellus	5	1	0,64	1,24	Gelechia sestertiella	8,77	1/1	continuous
Gelechiidae	Gelechia sestertiella	3	1	0,08	0,15	Gelechia sabinellus	8,77	1/1	fragmented
Gelechiidae	Gelechia sororculella	4	1	1,37	2,34	Gelechia sabinellus	9,14	2/1	continuous
Gelechiidae	Gnorimoschema epithymella	3	3	0,24	0,48	Caryocolum cassella	9,72	1/1	disjunct
Gelechiidae	Helcystogramma rufescens	3	1	0,09	0,31	Deileptenia ribeata	9,56	1/1	continuous
Gelechiidae	Monochroa hornigi	8	1	0	0	Monochroa lutulentella	7,59	1/1	continuous
Gelechiidae	Monochroa lutulentella	4	1	0,53	1,24	Monochroa hornigi	7,59	1/1	continuous
Gelechiidae	Monochroa servella	3	2	0,09	0,15	Monochroa hornigi	8,41	1/1	continuous
Gelechiidae	Neofaculta ericetella	3	2	0,28	0,46	Neofaculta infernella	5,08	1/1	continuous
Gelechiidae	Neofaculta infernella	14	2	4,38	8,88	Neofaculta ericetella	5,08	2/1	continuous
Gelechiidae	Parachronistis albiceps	6	1	1,05	3,31	Xestia ashworthii	11,39	2/0	continuous
Gelechiidae	Pexicopia malvella	4	3	0,09	0,16	Scoparia ambigualis	8,21	1/1	continuous
Gelechiidae	Prolita sexpunctella	3	1	0,38	0,77	Chionodes fumatella	8,75	1/1	fragmented

Gelechiidae	Psoricoptera gibbosella	4	1	0,39	0,99	Teleiodes vulgella	8,1	1/1	continuous
Gelechiidae	Recurvaria leucatella	3	2	0,17	0,46	Teleiodes wagae	9,19	1/1	continuous
Gelechiidae	Scrobipalpa acuminatella	5	1	0,32	0,46	Scrobipalpa pauperella	9,51	1/1	continuous
Gelechiidae	Scrobipalpa pauperella	3	1	0,05	0,15	Scrobipalpa acuminatella	9,51	1/1	continuous
Gelechiidae	Scrobipalpopsis petasitis	3	2	0,56	0,93	Gnorimoschema epithymella	11,16	1/1	disjunct
Gelechiidae	Syncopacma cinctella	10	2	0,13	0,31	Syncopacma sangiella	7,57	1/1	continuous
Gelechiidae	Syncopacma sangiella	4	1	0	0	Syncopacma cinctella	7,57	1/1	continuous
Gelechiidae	Syncopacma taeniolella	5	1	0,1	0,31	Syncopacma sangiella	7,74	1/1	continuous
Gelechiidae	Teleiodes flavimaculella	3	1	0	0	Teleiodes luculella	3,14	1/1	continuous
Gelechiidae	Teleiodes luculella	3	1	0,08	0,15	Teleiodes flavimaculella	3,14	1/1	continuous
Gelechiidae	Teleiodes vulgella	3	1	0,2	0,31	Carpatolechia alburnella	7,56	1/1	continuous
Gelechiidae	Teleiodes wagae	2	1	0,22	0,35	Carpatolechia alburnella	7,05	1/1	continuous
Gelechiidae	Thiotricha subocellea	4	1	0,71	1,24	Evergestis pallidata	9,27	1/1	continuous
Geometridae	Abraxas sylvata	5	1	0,24	0,31	Epirrhoe alternata	10,77	1/1	continuous
Geometridae	Acasis viretata	3	1	0,31	0,62	Hydria cervinalis	11,43	1/1	continuous
Geometridae	Agriopis marginaria	2	1	0,1	0,15	Peribatodes secundaria	6,89	1/1	continuous
Geometridae	Alcis repandata	3	2	0,18	0,31	Arichanna melanaria	8,58	1/1	continuous
Geometridae	Angerona prunaria	4	1	0,18	0,46	Peribatodes secundaria	8,24	1/1	continuous
Geometridae	Anticlea derivata	2	1	1,03	1,39	Xanthorhoe designata	9,28	1/1	continuous
Geometridae	Apeira syringaria	2	1	0,21	0,31	Xanthorhoe designata	10,52	1/1	continuous
Geometridae	Aplocera praeformata	3	1	0,26	0,47	Xanthorhoe designata	9,1	1/1	continuous
Geometridae	Arichanna melanaria	3	1	0,15	0,31	Bupalus piniaria	5,41	1/1	continuous
Geometridae	Asthena albulata	3	1	0,23	0,46	Camptogramma bilineata	9,1	1/1	continuous
Geometridae	Baptria tibiale	4	1	0,15	0,31	Xanthorhoe designata	9,14	1/1	fragmented
Geometridae	Biston betularia	5	1	0,19	0,48	Biston strataria	7,4	1/1	continuous
Geometridae	Biston strataria	4	1	0,25	0,46	Biston betularia	7,4	1/1	continuous
Geometridae	Bupalus piniaria	3	1	0	0	Arichanna melanaria	5,41	1/1	continuous
Geometridae	Cabera exanthemata	3	1	0,27	0,48	Cabera pusaria	5,66	1/1	continuous
Geometridae	Cabera pusaria	10	2	0,22	0,49	Cabera exanthemata	5,66	1/1	continuous
Geometridae	Campaea margaritaria	3	1	0	0	Lycia hirtaria	10,14	1/1	continuous
Geometridae	Camptogramma bilineata	3	1	0,74	1,08	Xanthorhoe quadrifasiata	6,58	1/1	continuous

Geometridae	Carsia sororiata	3	1	0,64	0,77	Eupithecia egenaria	10,32	1/1	fragmented
Geometridae	Catarhoe cuculata	3	2	0,06	0,16	Xanthorhoe designata	8,05	1/1	continuous
Geometridae	Cepphis advenaria	4	1	0,12	0,31	Peribatodes secundaria	8,42	1/1	continuous
Geometridae	Chiasmia clathrata	4	1	0,25	0,32	Pseudopanthera macularia	8,21	1/1	continuous
Geometridae	Chloroclysta miata	3	1	0,08	0,16	Chloroclysta siterata	1,87	1/1	continuous
Geometridae	Chloroclysta siterata	3	1	0,54	1,08	Chloroclysta miata	1,87	1/1	continuous
Geometridae	Chloroclystis v-ata	8	1	0,07	0,31	Gymnoscelis rufifasciata	8,24	1/1	continuous
Geometridae	Cleora cinctaria	4	1	0,22	0,32	Phigalia pilosaria	9,56	1/1	continuous
Geometridae	Colostygia aptata	5	2	0,27	0,62	Colostygia olivata	7,26	1/1	continuous
Geometridae	Colostygia olivata	3	1	1,65	3,3	Colostygia turbata	6,39	2/0	continuous
Geometridae	Colostygia pectinataria	3	1	0,96	1,61	Colostygia turbata	8,41	1/1	continuous
Geometridae	Colostygia turbata	4	1	1,2	3,03	Colostygia olivata	6,39	2/0	disjunct
Geometridae	Colotois pennaria	3	1	1,41	2,82	Mesapamea secalella	9,44	2/0	continuous
Geometridae	Cosmorhoe ocellata	3	1	0,44	1	Pennithera firmata	8,65	1/1	continuous
Geometridae	Crocallis elinguaria	3	1	0	0	Rivula sericealis	11,94	1/1	continuous
Geometridae	Cyclophora annularia	1	1	0,15	0,15	Cyclophora linearia	3,97	1/1	continuous
Geometridae	Cyclophora linearia	1	1	0	0	Cyclophora annularia	3,97	1/1	continuous
Geometridae	Deileptenia ribeata	3	1	0,18	0,31	Siona lineata	7,22	1/1	continuous
Geometridae	Dysstroma citrata	4	1	0,38	0,62	Dysstroma truncata	6,93	1/1	continuous
Geometridae	Dysstroma truncata	14	1	1,63	6,47	Dysstroma citrata	6,93	2/1	continuous
Geometridae	Earophila badiata	4	1	0,05	0,15	Plemyria rubiginata	11,41	1/1	continuous
Geometridae	Ecliptopera capitata	3	1	0,29	0,46	Ecliptopera silaceata	9,18	1/1	continuous
Geometridae	Ecliptopera silaceata	3	1	0,24	0,48	Ecliptopera capitata	9,18	1/1	continuous
Geometridae	Ectropis crepuscularia	19	1	3,23	6,97	Deileptenia ribeata	7,95	2/1	continuous
Geometridae	Electrophaes corylata	4	1	0,28	0,61	Thera obeliscata	9,11	1/1	continuous
Geometridae	Elophos vittaria	3	1	3,08	6,12	Hydria undulata	8,95	2/0	fragmented
Geometridae	Ematurga atomaria	3	1	0,69	1,24	Hypomecis punctinalis	8,88	1/1	continuous
Geometridae	Ennomos alniaria	3	1	0,08	0,16	Elophos vittaria	10,38	1/1	continuous
Geometridae	Entephria caesiata	3	1	0,39	0,77	Entephria flavicinctata	5,92	1/1	continuous
Geometridae	Entephria flavicinctata	3	2	0,46	0,77	Entephria caesiata	5,92	1/1	fragmented
Geometridae	Epirrhoe alternata	9	1	0,19	0,46	Epirrhoe rivata	3,14	1/1	continuous

Geometridae	Epirrhoe galiata	3	1	0,08	0,15	Epirrhoe tristata	6,84	1/1	continuous
Geometridae	Epirrhoe rivata	4	1	0,37	0,64	Epirrhoe alternata	3,14	1/1	continuous
Geometridae	Epirrhoe tristata	4	1	0,57	0,96	Epirrhoe rivata	6,35	1/1	continuous
Geometridae	Epirrita autumnata	8	2	1,8	3,95	Epirrita christyi	8,12	3/2	continuous
Geometridae	Epirrita christyi	4	1	0,12	0,32	Epirrita autumnata	8,12	1/1	continuous
Geometridae	Erannis defoliaria	3	1	0,26	0,46	Deileptenia ribeata	8,24	1/1	continuous
Geometridae	Euchoeca nebulata	3	1	0,08	0,15	Hydrelia flammeolaria	8,43	1/1	continuous
Geometridae	Eulithis populata	3	2	0,12	0,31	Epirrhoe alternata	9,21	1/1	continuous
Geometridae	Eupithecia abietaria	3	2	0,12	0,31	Eupithecia tenuiata	9,3	1/1	continuous
Geometridae	Eupithecia absinthiata	11	3	0,31	1,24	Eupithecia satyrata	5,24	1/1	continuous
Geometridae	Eupithecia egenaria	3	1	0,1	0,16	Eupithecia satyrata	7,67	1/1	continuous
Geometridae	Eupithecia exiguata	3	2	0,15	0,31	Eupithecia icterata	6,59	1/1	continuous
Geometridae	Eupithecia icterata	4	1	0,71	1,7	Eupithecia vulgata	6,07	2/0	continuous
Geometridae	Eupithecia indigata	3	1	0,31	0,47	Eupithecia absinthiata	7,09	1/1	continuous
Geometridae	Eupithecia intricata	3	1	0	0	Eupithecia absinthiata	8,27	1/1	continuous
Geometridae	Eupithecia lanceata	4	1	0,28	0,65	Eupithecia absinthiata	7,73	1/1	continuous
Geometridae	Eupithecia lariciata	3	1	0,1	0,15	Eupithecia absinthiata	9,49	1/1	continuous
Geometridae	Eupithecia plumbeolata	17	2	1,99	4,33	Eupithecia tenuiata	7,43	2/1	continuous
Geometridae	Eupithecia pusillata	16	3	0,21	1,47	Eupithecia tenuiata	7,25	1/1	continuous
Geometridae	Eupithecia satyrata	36	1	0,69	5,17	Eupithecia absinthiata	5,24	2/1	continuous
Geometridae	Eupithecia selinata	4	1	0	0	Eupithecia plumbeolata	8,33	1/1	continuous
Geometridae	Eupithecia subfuscata	7	5	0,77	3,14	Eupithecia absinthiata	8,1	2/1	continuous
Geometridae	Eupithecia subumbrata	8	1	0,58	2,34	Eupithecia icterata	7,3	2/0	continuous
Geometridae	Eupithecia tantillaria	6	1	0,29	0,99	Eupithecia lanceata	8,13	1/1	continuous
Geometridae	Eupithecia tenuiata	4	2	0,27	0,62	Eupithecia pusillata	7,25	1/1	continuous
Geometridae	Eupithecia trisignaria	3	1	0,23	0,46	Eupithecia tenuiata	7,6	1/1	continuous
Geometridae	Eupithecia venosata	3	1	0,41	0,62	Eupithecia tantillaria	8,8	1/1	continuous
Geometridae	Eupithecia virgaureata	17	2	0,25	1,45	Eupithecia pusillata	9,51	1/1	continuous
Geometridae	Eupithecia vulgata	7	1	1,1	2,35	Eupithecia icterata	6,07	1/1	continuous
Geometridae	Eustroma reticulata	3	1	0,15	0,31	Xanthorhoe fluctuata	8,68	1/1	continuous
Geometridae	Gagitodes sagittata	3	1	0,56	0,92	Eupithecia tenuiata	10,51	1/1	continuous

Geometridae	Gandaritis pyraliata	3	1	1,48	2,34	Xanthorhoe quadrifasiata	8,58	1/1	continuous
Geometridae	Geometra papilionaria	34	1	0,61	1,55	Depressaria radiella	10,02	1/1	continuous
Geometridae	Gnophos obfuscata	3	1	0,33	0,62	Siona lineata	7,39	1/1	continuous
Geometridae	Gymnoscelis rufifasciata	3	1	1	1,55	Chloroclystis v-ata	8,24	1/1	continuous
Geometridae	Hemithea aestivaria	3	1	0,23	0,46	Cabera pusaria	8,97	1/1	continuous
Geometridae	Horisme tersata	3	1	0,33	0,62	Hydrelia sylvata	10,74	1/1	continuous
Geometridae	Hydrelia flammeolaria	3	1	1,4	2,73	Lampropteryx suffumata	7,5	2/0	continuous
Geometridae	Hydrelia sylvata	3	1	0,08	0,17	Venusia blomeri	7,27	1/1	continuous
Geometridae	Hydria cervinalis	3	1	0	0	Hydria undulata	6,44	1/1	continuous
Geometridae	Hydria undulata	4	1	0,06	0,16	Rheumaptera subhastata	5,94	1/1	continuous
Geometridae	Hydriomena furcata	3	1	0,31	0,46	Hydriomena ruberata	7,9	1/1	continuous
Geometridae	Hydriomena impluviata	4	1	0,81	1,87	Hydriomena ruberata	5,61	2/0	continuous
Geometridae	Hydriomena ruberata	4	1	0,28	0,46	Hydriomena impluviata	5,61	1/1	continuous
Geometridae	Hylaea fasciaria	3	1	0,39	0,8	Angerona prunaria	9,46	1/1	continuous
Geometridae	Hypomecis punctinalis	3	1	3,49	7	Deileptenia ribeata	8,41	2/0	continuous
Geometridae	Hypomecis roboraria	3	1	0,15	0,31	Lycia hirtaria	8,24	1/1	continuous
Geometridae	Hypoxystis pluviaria	4	1	0,25	0,48	Jodis putata	7,73	1/1	fragmented
Geometridae	Idaea biselata	3	1	0,08	0,16	Idaea muricata	11,29	1/1	continuous
Geometridae	Idaea muricata	3	1	0	0	Idaea seriata	8,24	1/1	continuous
Geometridae	Idaea seriata	3	2	1,26	3,15	Idaea muricata	8,24	2/1	continuous
Geometridae	Idaea serpentata	3	1	0,15	0,31	Idaea muricata	9,28	1/1	continuous
Geometridae	Jodis lactearia	3	1	0,1	0,16	Jodis putata	3,37	1/1	continuous
Geometridae	Jodis putata	3	1	0	0	Jodis lactearia	3,37	1/1	continuous
Geometridae	Lampropteryx suffumata	8	1	0,1	0,46	Hydrelia flammeolaria	7,5	1/1	continuous
Geometridae	Lobophora halterata	3	2	0,15	0,31	Pterapherapteryx sexalata	6,4	1/1	continuous
Geometridae	Lomaspilis marginata	4	1	0,37	0,96	Pseudopanthera macularia	9,79	1/1	continuous
Geometridae	Lomographa bimaculata	3	1	0	0	Xanthorhoe fluctuata	9,44	1/1	continuous
Geometridae	Lomographa temerata	3	1	0,05	0,15	Jodis lactearia	9,45	1/1	continuous
Geometridae	Lycia hirtaria	3	1	0,15	0,31	Agriopis marginaria	7,75	1/1	continuous
Geometridae	Macaria alternata	3	1	0,32	0,64	Macaria liturata	6,78	1/1	continuous
Geometridae	Macaria brunneata	3	1	0,08	0,15	Macaria fusca	8,66	1/1	continuous

Geometridae	Macaria fusca	3	2	0,68	1,24	Macaria brunneata	8,66	1/1	disjunct
Geometridae	Macaria liturata	3	1	1,32	2,18	Macaria signaria	5,28	3/1	continuous
Geometridae	Macaria signaria	3	1	0	0	Macaria liturata	5,28	1/1	continuous
Geometridae	Martania taeniata	3	1	0,34	0,78	Epirrhoe tristata	7,74	1/1	continuous
Geometridae	Mesoleuca albicillata	4	1	3	6,16	Entephria flavicinctata	8,77	2/1	continuous
Geometridae	Mesotype didymata	3	1	0,21	0,33	Perizoma minorata	9,82	1/1	continuous
Geometridae	Odezia atrata	3	2	2,31	3,95	Venusia blomeri	8,93	3/1	continuous
Geometridae	Odontopera bidentata	3	1	0	0	Mesapamea secalella	9,83	1/1	continuous
Geometridae	Operophtera brumata	3	1	0,57	0,78	Operophtera fagata	6,97	1/1	continuous
Geometridae	Operophtera fagata	3	1	0,15	0,31	Operophtera brumata	6,97	1/1	continuous
Geometridae	Opisthograptis luteolata	3	1	0,08	0,16	Deileptenia ribeata	8,07	1/1	continuous
Geometridae	Orthonama obstipata	1	1	0,15	0,15	Eupithecia selinata	8,65	1/1	migrating
Geometridae	Ourapteryx sambucaria	4	1	0,4	0,62	Deileptenia ribeata	8,75	1/1	continuous
Geometridae	Paradarisa consonaria	4	1	0,06	0,16	Ectropis crepuscularia	9,44	1/1	continuous
Geometridae	Pasiphila debiliata	4	1	0,16	0,33	Pasiphila rectangulata	11,02	1/1	continuous
Geometridae	Pasiphila rectangulata	5	1	0,18	0,31	Colostygia turbata	8,76	1/1	continuous
Geometridae	Pennithera firmata	4	1	0,12	0,31	Cosmorhoe ocellata	8,65	1/1	continuous
Geometridae	Peribatodes secundaria	3	1	0,08	0,15	Agriopis marginaria	6,89	1/1	continuous
Geometridae	Perizoma affinitata	6	1	0,75	1,32	Perizoma hydrata	0,15	1/1	continuous
Geometridae	Perizoma alchemillata	3	1	0,63	1,24	Perizoma minorata	9,53	1/1	continuous
Geometridae	Perizoma blandiata	5	1	0	0	Perizoma minorata	8,14	1/1	continuous
Geometridae	Perizoma hydrata	5	1	0	0	Perizoma affinitata	0,15	1/1	continuous
Geometridae	Perizoma minorata	3	1	0	0	Perizoma blandiata	8,14	1/1	continuous
Geometridae	Petrophora chlorosata	3	1	0,15	0,31	Agriopis marginaria	8,77	1/1	continuous
Geometridae	Phigalia pilosaria	3	1	0,08	0,17	Peribatodes secundaria	8,05	1/1	continuous
Geometridae	Philereme transversata	2	1	0,51	0,62	Philereme vetulata	9,9	1/1	continuous
Geometridae	Philereme vetulata	3	1	0,23	0,31	Philereme transversata	9,9	1/1	continuous
Geometridae	Plagodis dolabraria	3	1	0,21	0,32	Plagodis pulveraria	4,91	1/1	continuous
Geometridae	Plagodis pulveraria	5	1	0,6	1,08	Plagodis dolabraria	4,91	1/1	continuous
Geometridae	Plemyria rubiginata	3	1	0,16	0,32	Chloroclysta siterata	7,52	1/1	continuous
Geometridae	Pseudopanthera macularia	3	1	0,31	0,62	Arichanna melanaria	8,07	1/1	continuous

Geometridae	Pterapherapteryx sexalata	4	1	0,48	0,77	Lobophora halterata	6,4	1/1	continuous
Geometridae	Rheumaptera hastata	7	1	0,41	1	Rheumaptera subhastata	1,28	1/1	continuous
Geometridae	Rheumaptera subhastata	14	1	1,73	5,07	Rheumaptera hastata	1,28	2/1	fragmented
Geometridae	Scopula caricaria	3	1	0,15	0,31	Scopula floslactata	8,28	1/1	continuous
Geometridae	Scopula floslactata	4	1	0,2	0,31	Scopula incanata	6,5	1/1	continuous
Geometridae	Scopula incanata	3	1	0,51	0,62	Scopula floslactata	6,5	1/1	continuous
Geometridae	Scopula ornata	2	1	0,31	0,47	Scopula incanata	8,92	1/1	continuous
Geometridae	Scopula ternata	3	1	0,87	1,39	Scopula floslactata	7,2	1/1	continuous
Geometridae	Scopula virgulata	5	1	0,18	0,31	Scopula ternata	8,58	1/1	continuous
Geometridae	Scotopteryx chenopodiata	4	1	0,06	0,16	Xanthorhoe designata	13,49	1/1	continuous
Geometridae	Selenia dentaria	4	1	0,42	0,62	Selenia tetralunaria	9,42	1/1	continuous
Geometridae	Selenia lunularia	3	1	1,33	2,67	Jodis lactearia	8,07	2/0	continuous
Geometridae	Selenia tetralunaria	5	1	0,05	0,16	Selenia lunularia	8,97	1/1	continuous
Geometridae	Siona lineata	3	1	0,18	0,31	Deileptenia ribeata	7,22	1/1	continuous
Geometridae	Spargania luctuata	3	1	0,05	0,16	Hydriomena furcata	9,62	1/1	continuous
Geometridae	Thera cognata	3	1	0	0	Thera obeliscata	7,25	1/1	continuous
Geometridae	Thera juniperata	3	3	0,55	1,19	Thera cognata	9,21	1/1	continuous
Geometridae	Thera obeliscata	11	1	1,25	7,37	Thera variata	0	2/1	continuous
Geometridae	Thera variata	10	3	0,09	0,61	Thera obeliscata	0	1/1	continuous
Geometridae	Timandra comae	8	1	0,07	0,31	Hedya pruniana	10,31	1/1	continuous
Geometridae	Trichopteryx carpinata	4	1	0,4	0,77	Trichopteryx polycommata	7,6	1/1	continuous
Geometridae	Trichopteryx polycommata	3	1	0,15	0,31	Trichopteryx carpinata	7,6	1/1	continuous
Geometridae	Triphosa dubitata	3	1	0,28	0,46	Hydria cervinalis	8,42	1/1	continuous
Geometridae	Venusia blomeri	5	1	0,1	0,31	Hydrelia sylvata	7,27	1/1	continuous
Geometridae	Venusia cambrica	4	1	0,15	0,31	Venusia blomeri	8,68	1/1	continuous
Geometridae	Xanthorhoe biriviata	3	2	0,15	0,31	Xanthorhoe decoloraria	6,89	1/1	continuous
Geometridae	Xanthorhoe decoloraria	3	1	0,69	1,08	Xanthorhoe montanata	5,4	1/1	disjunct
Geometridae	Xanthorhoe designata	9	1	1,13	2,54	Xanthorhoe fluctuata	4,6	2/1	continuous
Geometridae	Xanthorhoe ferrugata	17	2	1,22	3,63	Xanthorhoe spadicearia	5,26	2/1	continuous
Geometridae	Xanthorhoe fluctuata	3	1	0,62	0,96	Xanthorhoe designata	4,6	1/1	continuous
Geometridae	Xanthorhoe montanata	3	1	0,53	1,08	Xanthorhoe decoloraria	5,4	1/1	continuous

Geometridae	Xanthorhoe quadrifasiata	4	1	0	0	Camptogramma bilineata	6,58	1/1	continuous	
Geometridae	Xanthorhoe spadicearia	3	1	0,27	0,49	Xanthorhoe ferrugata	5,26	1/1	continuous	
Glyphipterigidae	Digitivalva reticulella	3	1	0,08	0,15	Teleiodes wagae	10,66	1/1	fragmented	
Glyphipterigidae	Glyphipterix forsterella	7	1	3,1	5,78	Mompha terminella	12,48	2/1	continuous	
Glyphipterigidae	Glyphipterix thrasonella	3	1	0	0	Elachista occidentalis	11,37	1/1	continuous	
Gracillariidae	Aspilapteryx tringipennella	3	2	0,37	0,62	Caloptilia cuculipennella	11,73	1/1	continuous	
Gracillariidae	Callisto coffeella	4	1	0,46	1,08	Parornix betulae	10,14	1/1	disjunct	poor
Gracillariidae	Caloptilia alchimiella	3	1	0	0	Caloptilia robustella	5,08	1/1	continuous	poor
Gracillariidae	Caloptilia cuculipennella	3	1	0,8	1,39	Caloptilia elongella	9,96	1/1	fragmented	poor
Gracillariidae	Caloptilia elongella	5	2	0,32	0,77	Caloptilia alchimiella	7,06	1/1	continuous	poor
Gracillariidae	Caloptilia robustella	4	1	0,09	0,15	Caloptilia alchimiella	5,08	1/1	continuous	poor
Gracillariidae	Caloptilia stigmatella	4	1	0,12	0,31	Caloptilia elongella	10,7	1/1	continuous	poor
Gracillariidae	Calybites phasianipennella	2	1	0	0	Deileptenia ribeata	11,38	1/1	continuous	poor
Gracillariidae	Euspilapteryx auroguttella	3	1	0,1	0,16	Caloptilia elongella	11,91	1/1	continuous	poor
Gracillariidae	Gracillaria syringella	5	3	0,04	0,16	Caloptilia cuculipennella	10,56	1/1	continuous	poor
Gracillariidae	Parectopa ononidis	3	1	0,58	0,92	Archips rosana	12,52	1/1	continuous	poor
Gracillariidae	Parornix betulae	5	1	1,71	3,19	Parornix devoniella	8,09	2/1	continuous	poor
Gracillariidae	Parornix devoniella	2	2	0,46	0,93	Parornix betulae	8,09	1/1	continuous	poor
Gracillariidae	Phyllonorycter cavella	3	1	0	0	Phyllonorycter maestingella	7,57	1/1	continuous	poor
Gracillariidae	Phyllonorycter harrisella	3	1	0,15	0,31	Phyllonorycter strigulatella	11,67	1/1	continuous	poor
Gracillariidae	Phyllonorycter maestingella	3	1	0,08	0,15	Phyllonorycter cavella	7,57	1/1	continuous	poor
Gracillariidae	Phyllonorycter strigulatella	5	1	0,54	1,08	Phyllonorycter maestingella	9,31	1/1	continuous	poor
Hepialidae	Gazoryctra ganna	3	1	0,96	1,71	Pharmacis fusconebulosa	11,88	1/1	disjunct	
Hepialidae	Hepialus humuli	3	13	1,46	3,31	Pharmacis lupulina	8,8	3/1	continuous	
Hepialidae	Pharmacis fusconebulosa	5	1	0,14	0,5	Pharmacis lupulina	3,04	1/1	continuous	
Hepialidae	Pharmacis lupulina	2	1	0,2	0,31	Pharmacis fusconebulosa	3,04	1/1	continuous	
Hepialidae	Phymatopus hecta	3	1	0,05	0,16	Triodia sylvina	8,97	1/1	continuous	
Hepialidae	Triodia sylvina	4	1	0,38	0,64	Pharmacis fusconebulosa	7,45	1/1	continuous	
Hesperiidae	Carterocephalus palaemon	5	1	0,25	0,46	Thyatira batis	10,14	1/1	continuous	
Hesperiidae	Hesperia comma	3	1	0,4	0,81	Ochlodes sylvanus	7,06	1/1	continuous	
Hesperiidae	Ochlodes sylvanus	3	1	0,08	0,16	Hesperia comma	7,06	1/1	continuous	

Hesperiidae	Pyrgus alveus	2	1	0,41	0,62	Pyrgus andromedae	2,81	1/1	continuous	
Hesperiidae	Pyrgus andromedae	4	1	0,38	0,96	Pyrgus alveus	2,81	1/1	disjunct	
Hesperiidae	Pyrgus malvae	3	2	0,06	0,15	Pyrgus andromedae	3,29	1/1	continuous	
Hesperiidae	Thymelicus lineola	3	1	1,35	2,65	Ochlodes sylvanus	8,88	2/0	continuous	
Lasiocampidae	Cosmotriche lobulina	54	2	0,26	0,93	Euthrix potatoria	8,61	1/1	continuous	
Lasiocampidae	Dendrolimus pini	3	1	0,26	0,46	Macrothylacia rubi	10,01	1/1	continuous	
Lasiocampidae	Euthrix potatoria	3	1	0,08	0,15	Cosmotriche lobulina	8,61	1/1	continuous	
Lasiocampidae	Lasiocampa quercus	11	1	0,63	2,34	Arichanna melanaria	10,49	1/1	continuous	
Lasiocampidae	Macrothylacia rubi	3	1	0,08	0,15	Deileptenia ribeata	9,44	1/1	continuous	
Lasiocampidae	Malacosoma neustria	2	1	0,1	0,15	Biston betularia	9,16	1/1	continuous	
Lasiocampidae	Poecilocampa populi	4	1	0,49	1,24	Deileptenia ribeata	9,96	1/1	continuous	
Lasiocampidae	Trichiura crataegi	11	1	0,28	1,55	Poecilocampa populi	10,96	1/1	continuous	
Lycaenidae	Aricia artaxerxes	3	8	0	0	Plebejus argus	5,4	1/1	continuous	
Lycaenidae	Callophrys rubi	3	1	0	0	Glaucopsyche alexis	7,22	1/1	continuous	
Lycaenidae	Celastrina argiolus	3	1	0,08	0,15	Plebejus argus	7,73	1/1	continuous	
Lycaenidae	Cupido minimus	3	1	0,18	0,32	Callophrys rubi	7,56	1/1	continuous	
Lycaenidae	Cyaniris semiargus	2	1	0,1	0,15	Eumedonia eumedon	5,57	1/1	continuous	
Lycaenidae	Eumedonia eumedon	3	2	0,55	0,92	Cyaniris semiargus	5,57	1/1	continuous	
Lycaenidae	Glaucopsyche alexis	4	1	0	0	Callophrys rubi	7,22	1/1	continuous	
Lycaenidae	Lycaena hippothoe	3	2	0,12	0,31	Lycaena virgaureae	3,46	1/1	continuous	
Lycaenidae	Lycaena phlaeas	3	1	0,23	0,46	Lycaena hippothoe	5,24	1/1	continuous	
Lycaenidae	Lycaena virgaureae	3	1	0,26	0,47	Lycaena hippothoe	3,46	1/1	continuous	
Lycaenidae	Plebejus argus	3	1	0,23	0,46	Plebejus idas	2,81	1/1	continuous	
Lycaenidae	Plebejus idas	3	1	0,08	0,15	Plebejus argus	2,81	1/1	continuous	
Lycaenidae	Polyommatus icarus	3	1	0,8	1,24	Plebejus argus	6,93	1/1	continuous	
Lyonetiidae	Lyonetia clerkella	4	1	3,08	5,11	Parasemia plantaginis	12,09	2/1	continuous	
Lypusidae	Pseudatemelia josephinae	19	1	0,2	0,77	Athetis pallustris	9,46	1/1	continuous	
Micropterigidae	Micropterix aruncella	3	1	0,23	0,46	Micropterix aureatella	4,72	1/1	continuous	poor
Micropterigidae	Micropterix aureatella	6	2	0,34	0,82	Micropterix aruncella	4,72	1/1	continuous	poor
Momphidae	Mompha conturbatella	4	1	0,3	0,78	Mompha lacteella	5,23	1/1	continuous	poor
Momphidae	Mompha lacteella	3	1	0,23	0,46	Mompha conturbatella	5,23	1/1	continuous	poor

Momphidae	Mompha langiella	4	1	0,06	0,16	Tholera cespitis	10,67	1/1	continuous	poor
Momphidae	Mompha locupletella	3	1	0,23	0,46	Tyria jacobaeae	10,7	1/1	continuous	poor
Momphidae	Mompha raschkiella	3	1	0	0	Xestia ashworthii	11,51	1/1	continuous	poor
Momphidae	Mompha subbistrigella	5	2	0,18	0,33	Mompha lacteella	6,13	1/1	continuous	poor
Momphidae	Mompha terminella	1	1	0,15	0,15	Pyrrhia umbra	9,8	1/1	continuous	poor
Nepticulidae	Ectoedemia sericopeza	4	1	0,12	0,31	Thyatira batis	11,73	1/1	continuous	poor
Nepticulidae	Stigmella dryadella	1	1	2,18	2,18	Stigmella pretiosa	8,94	2/0	disjunct	poor
Nepticulidae	Stigmella floslactella	3	1	0	0	Stigmella myrtillella	9,27	1/1	continuous	poor
Nepticulidae	Stigmella myrtillella	5	1	0,29	0,62	Stigmella floslactella	9,27	1/1	continuous	poor
Nepticulidae	Stigmella pretiosa	3	1	0,09	0,17	Stigmella dryadella	8,94	1/1	fragmented	poor
Noctuidae	Abrostola triplasia	3	1	0,54	1,08	Macdunnoughia confusa	8,24	2/1	continuous	good
Noctuidae	Acronicta alni	13	1	0,15	0,46	Acronicta auricoma	8,43	1/1	continuous	good
Noctuidae	Acronicta auricoma	3	1	0,15	0,31	Acronicta rumicis	5,72	1/1	continuous	good
Noctuidae	Acronicta cuspis	3	1	0	0	Acronicta psi	2,97	1/1	continuous	good
Noctuidae	Acronicta euphorbiae	2	1	0	0	Acronicta auricoma	6,42	1/1	continuous	good
Noctuidae	Acronicta leporina	4	1	0,21	0,31	Acronicta euphorbiae	7,41	1/1	continuous	good
Noctuidae	Acronicta psi	5	1	0,44	0,93	Acronicta cuspis	2,97	1/1	continuous	good
Noctuidae	Acronicta rumicis	4	1	0,06	0,15	Acronicta auricoma	5,72	1/1	continuous	good
Noctuidae	Agrochola circellaris	3	1	0,23	0,47	Eupsilia transversa	6,56	1/1	continuous	good
Noctuidae	Agrochola helvola	3	1	0,08	0,15	Eupsilia transversa	6,36	1/1	continuous	good
Noctuidae	Agrochola litura	3	1	0,1	0,15	Eupsilia transversa	6,77	1/1	continuous	good
Noctuidae	Agrochola lota	3	1	0,49	0,77	Ipimorpha retusa	6,57	1/1	continuous	good
Noctuidae	Agrochola macilenta	3	1	0,38	0,46	Conistra vaccinii	7,09	1/1	continuous	good
Noctuidae	Agrochola nitida	3	1	0,38	0,77	Mesapamea secalella	4,85	1/1	continuous	good
Noctuidae	Agrotis clavis	3	1	0,15	0,31	Agrotis exclamationis	3,19	1/1	continuous	good
Noctuidae	Agrotis exclamationis	4	2	0	0	Agrotis clavis	3,19	1/1	continuous	good
Noctuidae	Agrotis ipsilon	3	1	0,15	0,31	Agrotis exclamationis	3,61	1/1	continuous	good
Noctuidae	Agrotis segetum	3	1	0,2	0,31	Agrotis exclamationis	3,19	1/1	continuous	good
Noctuidae	Allophyes oxyacanthae	3	1	0,15	0,31	Acronicta rumicis	8,41	1/1	continuous	good
Noctuidae	Ammoconia caecimacula	3	1	0,33	0,62	Antitype chi	7,73	1/1	continuous	good
Noctuidae	Amphipoea fucosa	23	3	0,19	0,82	Amphipoea oculea	2,77	1/1	continuous	good

Noctuidae	Amphipoea oculea	5	1	0,04	0,15	Amphipoea fucosa	2,77	1/1	continuous	good
Noctuidae	Amphipyra berbera	3	1	0	0	Graphiphora augur	7,05	1/1	migrating	good
Noctuidae	Amphipyra perflua	5	1	0	0	Xestia c-nigrum	8,87	1/1	continuous	good
Noctuidae	Amphipyra pyramidea	3	1	0,18	0,31	Amphipyra berbera	7,28	1/1	continuous	good
Noctuidae	Amphipyra tragopoginis	3	1	0,62	1,08	Hadena albimacula	7,08	1/1	continuous	good
Noctuidae	Anaplectoides prasina	4	1	0,06	0,15	Eurois occulta	3,86	1/1	continuous	good
Noctuidae	Anarta myrtilli	3	1	0	0	Anorthoa munda	4,91	1/1	continuous	good
Noctuidae	Anorthoa munda	3	1	0	0	Orthosia opima	3,29	1/1	continuous	good
Noctuidae	Antitype chi	3	1	0,08	0,15	Xylena vetusta	6,39	1/1	continuous	good
Noctuidae	Apamea crenata	4	1	0,31	0,46	Apamea rubrirena	5,11	1/1	continuous	good
Noctuidae	Apamea furva	3	1	0,41	0,77	Apamea rubrirena	4,45	1/1	continuous	good
Noctuidae	Apamea illyria	3	1	0,08	0,16	Apamea furva	5,08	1/1	continuous	good
Noctuidae	Apamea lateritia	3	1	0,26	0,46	Apamea rubrirena	3,96	1/1	continuous	good
Noctuidae	Apamea monoglypha	3	1	1,24	1,87	Apamea sublustris	3,31	2/1	continuous	good
Noctuidae	Apamea rubrirena	3	1	0,26	0,46	Apamea lateritia	3,96	1/1	fragmented	good
Noctuidae	Apamea scolopacina	3	1	0,1	0,15	Amphipoea fucosa	5,81	1/1	continuous	good
Noctuidae	Apamea sublustris	3	1	0,38	0,77	Apamea monoglypha	3,31	1/1	continuous	good
Noctuidae	Apamea unanimis	3	1	0,15	0,31	Apamea illyria	5,26	1/1	continuous	good
Noctuidae	Apterogenum ypsillon	3	1	0,57	0,96	Enargia paleacea	4,74	1/1	continuous	good
Noctuidae	Athetis pallustris	3	1	0,23	0,46	Papestra biren	5,89	1/1	continuous	good
Noctuidae	Autographa bractea	4	1	0	0	Autographa pulchrina	5,97	1/1	continuous	good
Noctuidae	Autographa gamma	5	1	0	0	Autographa pulchrina	5,14	1/1	migrating	good
Noctuidae	Autographa jota	9	1	0	0	Autographa pulchrina	2,91	1/1	continuous	good
Noctuidae	Autographa pulchrina	63	3	0,17	0,96	Autographa jota	2,91	1/1	continuous	good
Noctuidae	Axylia putris	3	1	0,08	0,16	Xestia ashworthii	5,58	1/1	continuous	good
Noctuidae	Brachionycha nubeculosa	4	1	0,68	1,55	Parasemia plantaginis	7,05	1/1	continuous	good
Noctuidae	Brachylomia viminalis	3	1	0,6	0,8	Agrochola nitida	9,14	1/1	continuous	good
Noctuidae	Ceramica pisi	3	1	0	0	Papestra biren	4,09	1/1	continuous	good
Noctuidae	Cerapteryx graminis	4	1	0,19	0,49	Tholera decimalis	4,57	1/1	continuous	good
Noctuidae	Cerastis leucographa	3	1	0,17	0,34	Cerastis rubricosa	3,09	1/1	fragmented	good
Noctuidae	Cerastis rubricosa	3	1	0,15	0,31	Cerastis leucographa	3,09	1/1	continuous	good

Noctuidae	Charanyca ferruginea	3	2	0	0	Euxoa recussa	6,21	1/1	continuous	good
Noctuidae	Charanyca trigrammica	3	1	0,08	0,15	Hoplodrina octogenaria	6,07	1/1	continuous	good
Noctuidae	Chersotis cuprea	3	1	0,08	0,16	Epipsilia grisescens	6,36	1/1	continuous	good
Noctuidae	Colocasia coryli	3	1	0,62	1,24	Panthea coenobita	8,45	1/1	continuous	good
Noctuidae	Conistra rubiginea	3	1	1	1,55	Conistra rubiginosa	5,41	2/1	continuous	good
Noctuidae	Conistra rubiginosa	3	1	0,15	0,31	Conistra vaccinii	4,06	1/1	continuous	good
Noctuidae	Conistra vaccinii	4	1	0	0	Conistra rubiginosa	4,06	1/1	continuous	good
Noctuidae	Cosmia affinis	2	1	0	0	Eupsilia transversa	5,89	1/1	continuous	good
Noctuidae	Cosmia pyralina	4	1	0,06	0,15	Enargia paleacea	5,56	1/1	continuous	good
Noctuidae	Cosmia trapezina	3	1	0	0	Apterogenum ypsillon	4,92	1/1	continuous	good
Noctuidae	Craniophora ligustri	4	1	0,06	0,15	Griposia aprilina	7,05	1/1	continuous	good
Noctuidae	Crypsedra gemmea	3	1	0,15	0,31	Amphipoea fucosa	6,23	1/1	continuous	good
Noctuidae	Cucullia asteris	3	1	0	0	Cucullia umbratica	4,74	1/1	continuous	good
Noctuidae	Cucullia lucifuga	4	1	0,06	0,15	Cucullia umbratica	4,1	1/1	continuous	good
Noctuidae	Cucullia umbratica	4	1	0,18	0,31	Cucullia lucifuga	4,1	1/1	continuous	good
Noctuidae	Deltote bankiana	3	1	0	0	Deltote uncula	5,56	1/1	continuous	good
Noctuidae	Deltote pygarga	4	1	0	0	Deltote uncula	6,39	1/1	continuous	good
Noctuidae	Deltote uncula	3	1	0,15	0,31	Deltote bankiana	5,56	1/1	continuous	good
Noctuidae	Denticucullus pygmina	3	1	0,54	1,08	Amphipoea fucosa	5,95	1/1	continuous	good
Noctuidae	Diachrysia chrysitis	47	1	0,05	0,49	Macdunnoughia confusa	7,67	1/1	continuous	good
Noctuidae	Diarsia brunnea	4	1	0,16	0,33	Diarsia rubi	4,49	1/1	continuous	good
Noctuidae	Diarsia mendica	15	1	2,08	4,46	Diarsia rubi	4,15	2/1	continuous	good
Noctuidae	Diarsia rubi	9	1	0,17	0,46	Diarsia mendica	4,15	1/1	continuous	good
Noctuidae	Diloba caeruleocephala	3	1	0,64	1,24	Cerapteryx graminis	6,95	1/1	continuous	good
Noctuidae	Elaphria venustula	3	1	0,08	0,15	Sideridis rivularis	6,85	1/1	continuous	good
Noctuidae	Enargia paleacea	4	1	1,37	2,18	Apterogenum ypsillon	4,74	1/1	continuous	good
Noctuidae	Epipsilia grisescens	3	1	0,08	0,15	Standfussiana lucernea	3,79	1/1	continuous	good
Noctuidae	Euchalcia variabilis	2	1	2,9	4,42	Lamprotes c-aureum	7,59	2/0	fragmented	good
Noctuidae	Eugnorisma depuncta	3	1	1,87	3,64	Xestia c-nigrum	5,43	2/1	fragmented	good
Noctuidae	Eugraphe sigma	2	1	0,41	0,62	Xestia c-nigrum	5,42	1/1	continuous	good
Noctuidae	Euplexia lucipara	3	1	0,26	0,46	Orthosia opima	7,06	1/1	continuous	good

Noctuidae	Eupsilia transversa	3	3	0,15	0,46	Mesogona oxalina	3,77	1/1	continuous	good
Noctuidae	Eurois occulta	4	1	0	0	Xestia c-nigrum	3,86	1/1	continuous	good
Noctuidae	Euxoa nigricans	10	1	0,23	0,62	Euxoa recussa	2,98	1/1	continuous	good
Noctuidae	Euxoa recussa	3	1	0	0	Euxoa nigricans	2,98	1/1	continuous	good
Noctuidae	Graphiphora augur	3	1	0,15	0,31	Eurois occulta	4,03	1/1	continuous	good
Noctuidae	Griposia aprilina	3	1	0	0	Eupsilia transversa	5,72	1/1	continuous	good
Noctuidae	Hada plebeja	4	1	0,09	0,16	Hecatera bicolorata	4,36	1/1	continuous	good
Noctuidae	Hadena albimacula	3	1	0,19	0,33	Sideridis rivularis	5,34	1/1	continuous	good
Noctuidae	Hadena perplexa	3	2	0,37	0,62	Hada plebeja	5,09	1/1	continuous	good
Noctuidae	Hecatera bicolorata	3	1	0,15	0,31	Hada plebeja	4,36	1/1	continuous	good
Noctuidae	Heliothis nubigera	1	2	0,51	0,62	Heliothis peltigera	6,23	1/1	migrating	good
Noctuidae	Heliothis peltigera	1	1	0	0	Heliothis nubigera	6,23	1/1	migrating	good
Noctuidae	Helotropha leucostigma	3	1	0	0	Amphipoea fucosa	4,94	1/1	continuous	good
Noctuidae	Hoplodrina blanda	4	1	0,28	0,46	Hoplodrina octogenaria	3,65	1/1	continuous	good
Noctuidae	Hoplodrina octogenaria	9	2	0,02	0,16	Hoplodrina blanda	3,65	1/1	continuous	good
Noctuidae	Hydraecia micacea	8	1	0,13	0,31	Apamea rubrirena	5,61	1/1	continuous	good
Noctuidae	Hyppa rectilinea	3	2	0,44	0,64	Apamea rubrirena	6,93	1/1	continuous	good
Noctuidae	Ipimorpha retusa	3	1	0,08	0,15	Cosmia trapezina	5,57	1/1	continuous	good
Noctuidae	Ipimorpha subtusa	3	2	0	0	Mesapamea secalella	7,52	1/1	continuous	good
Noctuidae	Lacanobia contigua	4	2	0,14	0,32	Lacanobia suasa	3,77	1/1	continuous	good
Noctuidae	Lacanobia oleracea	4	1	0,16	0,32	Lacanobia suasa	3,26	1/1	continuous	good
Noctuidae	Lacanobia suasa	4	1	0,09	0,16	Lacanobia thalassina	2,02	1/1	continuous	good
Noctuidae	Lacanobia thalassina	3	1	0	0	Lacanobia suasa	2,02	1/1	continuous	good
Noctuidae	Lacanobia w-latinum	1	2	0,31	0,31	Lacanobia suasa	3,79	1/1	continuous	good
Noctuidae	Lamprotes c-aureum	5	1	0,1	0,31	Euchalcia variabilis	7,59	1/1	continuous	good
Noctuidae	Lasionycta imbecilla	3	3	0,42	0,77	Lacanobia oleracea	5,89	1/1	continuous	good
Noctuidae	Lasionycta proxima	3	1	0,99	1,39	Anorthoa munda	6,05	1/1	continuous	good
Noctuidae	Lateroligia ophiogramma	3	1	0,08	0,15	Amphipoea fucosa	4,6	1/1	continuous	good
Noctuidae	Leucania comma	3	1	0,24	0,48	Leucania obsoleta	3,58	1/1	continuous	good
Noctuidae	Leucania obsoleta	3	1	0,21	0,39	Leucania comma	3,58	1/1	continuous	good
Noctuidae	Lithophane consocia	3	1	0	0	Lithophane furcifera	3,04	1/1	continuous	good

Noctuidae	Lithophane furcifera	3	1	0,08	0,15	Lithophane consocia	3,04	1/1	continuous	good
Noctuidae	Lycophotia porphyrea	3	1	0,15	0,31	Euxoa nigricans	5,24	1/1	continuous	good
Noctuidae	Macdunnoughia confusa	3	1	0	0	Autographa bractea	7,4	1/1	migrating	good
Noctuidae	Mamestra brassicae	3	1	0	0	Lacanobia suasa	4,75	1/1	continuous	good
Noctuidae	Melanchra persicariae	3	1	0	0	Lacanobia suasa	5,4	1/1	continuous	good
Noctuidae	Mesapamea secalella	7	1	0,14	0,34	Amphipoea fucosa	4,08	1/1	continuous	good
Noctuidae	Mesogona oxalina	3	1	0,24	0,54	Eupsilia transversa	3,77	1/1	continuous	good
Noctuidae	Mesoligia furuncula	3	1	0,08	0,16	Mesapamea secalella	4,69	1/1	continuous	good
Noctuidae	Mniotype adusta	4	1	0,34	0,62	Griposia aprilina	6,89	1/1	continuous	good
Noctuidae	Mniotype satura	3	1	0	0	Amphipoea oculea	7,34	1/1	continuous	good
Noctuidae	Moma alpium	3	1	0,05	0,16	Deltote uncula	8,67	1/1	continuous	good
Noctuidae	Mormo maura	1	1	0,77	0,77	Enargia paleacea	7,6	1/1	continuous	good
Noctuidae	Mythimna conigera	3	1	0,18	0,31	Mythimna impura	6,62	1/1	continuous	good
Noctuidae	Mythimna ferrago	7	2	1,77	4,3	Leucania obsoleta	5,87	2/1	continuous	good
Noctuidae	Mythimna impura	3	1	0,38	0,77	Mythimna conigera	6,62	1/1	continuous	good
Noctuidae	Mythimna l-album	1	1	0	0	Leucania obsoleta	5,22	1/1	migrating	good
Noctuidae	Mythimna pudorina	3	1	0,46	0,77	Mythimna turca	5,75	1/1	continuous	good
Noctuidae	Mythimna straminea	3	1	0,49	0,92	Mythimna turca	7,1	1/1	continuous	good
Noctuidae	Mythimna turca	3	1	0,13	0,31	Leucania obsoleta	5,72	1/1	continuous	good
Noctuidae	Naenia typica	3	1	0,15	0,31	Eurois occulta	5,96	1/1	continuous	good
Noctuidae	Noctua comes	3	1	0,08	0,15	Noctua fimbriata	4,91	1/1	continuous	good
Noctuidae	Noctua fimbriata	4	1	0,12	0,31	Noctua janthe	3,45	1/1	continuous	good
Noctuidae	Noctua janthe	2	1	0	0	Noctua janthina	2,82	1/1	continuous	good
Noctuidae	Noctua janthina	4	1	0,06	0,15	Noctua janthe	2,82	1/1	continuous	good
Noctuidae	Noctua pronuba	3	1	0,38	0,61	Noctua fimbriata	4,09	1/1	continuous	good
Noctuidae	Nonagria typhae	3	1	0,39	0,77	Amphipoea fucosa	5,25	1/1	continuous	good
Noctuidae	Ochropleura plecta	3	1	2,12	3,47	Agrotis ipsilon	5,07	2/1	continuous	good
Noctuidae	Oligia strigilis	3	1	0	0	Amphipoea fucosa	5,74	1/1	continuous	good
Noctuidae	Orthosia cerasi	3	1	0	0	Orthosia gracilis	5,9	1/1	continuous	good
Noctuidae	Orthosia cruda	3	1	0,34	0,64	Anorthoa munda	4,27	1/1	continuous	good
Noctuidae	Orthosia gothica	4	1	0	0	Anorthoa munda	3,45	1/1	continuous	good

Noctuidae	Orthosia gracilis	3	1	0,08	0,16	Orthosia opima	1,86	1/1	continuous	good
Noctuidae	Orthosia incerta	4	1	0	0	Orthosia gracilis	4,94	1/1	continuous	good
Noctuidae	Orthosia opima	3	1	0	0	Orthosia gracilis	1,86	1/1	continuous	good
Noctuidae	Orthosia populeti	4	1	0	0	Anorthoa munda	3,63	1/1	continuous	good
Noctuidae	Pachetra sagittigera	2	1	0,83	1,24	Orthosia opima	5,52	2/0	continuous	good
Noctuidae	Panemeria tenebrata	4	1	0,15	0,31	Orthosia populeti	7,55	1/1	continuous	good
Noctuidae	Panolis flammea	3	1	0,39	0,79	Orthosia populeti	6,03	1/1	continuous	good
Noctuidae	Panthea coenobita	3	2	0,07	0,17	Papestra biren	7,72	1/1	continuous	good
Noctuidae	Papestra biren	3	1	0,08	0,16	Lacanobia oleracea	3,61	1/1	continuous	good
Noctuidae	Parastichtis suspecta	3	1	0,08	0,16	Griposia aprilina	5,81	1/1	continuous	good
Noctuidae	Phlogophora meticulosa	3	1	0	0	Agrotis clavis	6,89	1/1	migrating	good
Noctuidae	Photedes fluxa	3	1	0,52	0,78	Amphipoea fucosa	5,25	1/1	continuous	good
Noctuidae	Polia bombycina	4	1	0,13	0,32	Polia hepatica	4,26	1/1	continuous	good
Noctuidae	Polia hepatica	3	1	0,08	0,16	Polia bombycina	4,26	1/1	continuous	good
Noctuidae	Polia nebulosa	4	3	0,16	0,46	Polia bombycina	5,62	1/1	continuous	good
Noctuidae	Polychrysia moneta	3	1	0	0	Lamprotes c-aureum	8,91	1/1	continuous	good
Noctuidae	Protolampra sobrina	3	1	0	0	Xestia ashworthii	5,59	1/1	continuous	good
Noctuidae	Pyrrhia umbra	4	1	0,49	1,08	Cosmia affinis	6,72	1/1	continuous	good
Noctuidae	Sideridis reticulata	3	1	0	0	Lacanobia oleracea	4,42	1/1	continuous	good
Noctuidae	Sideridis rivularis	3	2	0,17	0,31	Anarta myrtilli	5,33	1/1	continuous	good
Noctuidae	Spodoptera exigua	3	1	0,26	0,46	Leucania obsoleta	7,33	1/1	migrating	good
Noctuidae	Standfussiana lucernea	4	1	0,12	0,31	Epipsilia grisescens	3,79	1/1	disjunct	good
Noctuidae	Syngrapha interrogationis	3	3	0,1	0,32	Autographa gamma	7,23	1/1	continuous	good
Noctuidae	Tholera cespitis	3	1	4,22	9,63	Eurois occulta	4,4	1/1	continuous	good
Noctuidae	Tholera decimalis	3	1	0,15	0,31	Cerapteryx graminis	4,57	1/1	continuous	good
Noctuidae	Tiliacea aurago	3	1	0,18	0,31	Tiliacea citrago	5,57	1/1	continuous	good
Noctuidae	Tiliacea citrago	3	1	0,08	0,15	Eupsilia transversa	5,22	1/1	continuous	good
Noctuidae	Trachea atriplicis	3	2	0,06	0,16	Noctua fimbriata	7,19	1/1	continuous	good
Noctuidae	Xanthia icteritia	4	1	0,44	0,77	Ipimorpha retusa	5,73	1/1	continuous	good
Noctuidae	Xanthia togata	3	1	0,15	0,31	Tiliacea citrago	6,73	1/1	continuous	good
Noctuidae	Xestia alpicola	5	1	0,05	0,15	Xestia baja	4,91	1/1	disjunct	good

Noctuidae	Xestia ashworthii	4	1	0,29	0,62	Xestia c-nigrum	2	1/1	continuous	good
Noctuidae	Xestia baja	3	2	0,09	0,16	Xestia alpicola	4,91	1/1	continuous	good
Noctuidae	Xestia c-nigrum	4	1	0	0	Xestia ashworthii	2	1/1	continuous	good
Noctuidae	Xestia collina	4	1	0,23	0,46	Tholera cespitis	4,75	1/1	continuous	good
Noctuidae	Xestia speciosa	7	5	1,39	2,78	Eurois occulta	4,55	2/0	fragmented	good
Noctuidae	Xestia triangulum	4	1	0,06	0,16	Xestia c-nigrum	2,92	1/1	continuous	good
Noctuidae	Xylena vetusta	3	1	0,47	0,95	Eupsilia transversa	4,9	1/1	continuous	good
Nolidae	Bena bicolorana	3	1	0,15	0,31	Agrotis clavis	7,72	1/1	continuous	
Nolidae	Earias clorana	3	1	0,08	0,15	Amphipoea fucosa	8,41	1/1	continuous	
Nolidae	Meganola strigula	3	1	0,15	0,31	Ochropacha duplaris	8,75	1/1	continuous	
Nolidae	Nola confusalis	3	1	0,23	0,46	Apamea rubrirena	8,58	1/1	continuous	
Nolidae	Nycteola degenerana	6	1	0,45	1,27	Nycteola revayana	3,91	1/1	continuous	
Nolidae	Nycteola revayana	5	1	0,37	0,8	Nycteola degenerana	3,91	1/1	continuous	
Nolidae	Pseudoips prasinana	4	1	0,06	0,15	Bena bicolorana	7,9	1/1	continuous	
Notodontidae	Cerura vinula	7	1	0,35	0,78	Furcula furcula	8,94	1/1	continuous	good
Notodontidae	Clostera anachoreta	3	1	0,08	0,15	Clostera pigra	6,22	1/1	continuous	good
Notodontidae	Clostera curtula	3	1	0,1	0,15	Clostera pigra	5,57	1/1	continuous	good
Notodontidae	Clostera pigra	4	1	0,06	0,15	Clostera curtula	5,57	1/1	continuous	good
Notodontidae	Furcula furcula	3	1	0,67	0,92	Cerura vinula	8,94	1/1	continuous	good
Notodontidae	Gluphisia crenata	3	1	0	0	Ethmia pusiella	10,84	1/1	continuous	good
Notodontidae	Notodonta dromedarius	5	5	3,64	6,59	Notodonta ziczac	5,77	2/0	continuous	good
Notodontidae	Notodonta ziczac	3	1	0,08	0,15	Notodonta dromedarius	5,77	1/1	continuous	good
Notodontidae	Odontosia carmelita	3	1	0,23	0,46	Orthosia opima	8,24	1/1	continuous	good
Notodontidae	Phalera bucephala	4	1	0,09	0,17	Xestia ashworthii	8,51	1/1	continuous	good
Notodontidae	Pheosia gnoma	4	1	0	0	Pheosia tremula	7,8	1/1	continuous	good
Notodontidae	Pheosia tremula	3	1	0,23	0,31	Pheosia gnoma	7,8	1/1	continuous	good
Notodontidae	Pterostoma palpina	10	1	0,29	0,92	Ochropacha duplaris	9,62	1/1	continuous	good
Notodontidae	Ptilodon capucina	3	1	0,56	1,08	Odontosia carmelita	11,8	1/1	continuous	good
Notodontidae	Stauropus fagi	4	1	0,09	0,15	Rivula sericealis	9,97	1/1	continuous	good
Nymphalidae	Aglais io	3	1	0	0	Polygonia c-album	5,63	1/1	continuous	good
Nymphalidae	Aglais urticae	3	1	0,21	0,32	Aglais io	6,07	1/1	continuous	good

Nymphalidae	Apatura ilia	3	1	0,39	0,77	Apatura iris	7,47	1/1	continuous	good
Nymphalidae	Apatura iris	3	1	0	0	Apatura ilia	7,47	1/1	continuous	good
Nymphalidae	Aphantopus hyperantus	3	1	1,49	2,86	Maniola jurtina	8,45	2/1	continuous	good
Nymphalidae	Araschnia levana	3	1	0	0	Polygonia c-album	9,11	1/1	continuous	good
Nymphalidae	Argynnis adippe	3	1	0,33	0,46	Argynnis niobe	2,82	1/1	continuous	good
Nymphalidae	Argynnis aglaja	3	1	0,23	0,46	Brenthis ino	5,23	1/1	continuous	good
Nymphalidae	Argynnis niobe	3	1	0,31	0,62	Argynnis adippe	2,82	1/1	continuous	good
Nymphalidae	Argynnis paphia	3	1	0	0	Argynnis niobe	5,77	1/1	continuous	good
Nymphalidae	Boloria aquilonaris	3	1	0,15	0,31	Boloria napaea	1,08	1/1	continuous	good
Nymphalidae	Boloria eunomia	3	1	0,54	1,08	Brenthis ino	7,57	1/1	continuous	good
Nymphalidae	Boloria euphrosyne	3	1	1,77	2,66	Boloria thore	7,94	2/1	continuous	good
Nymphalidae	Boloria napaea	3	1	0,15	0,31	Boloria aquilonaris	1,08	1/1	disjunct	good
Nymphalidae	Boloria selene	4	1	0,31	0,46	Boloria thore	4,11	1/1	continuous	good
Nymphalidae	Boloria thore	3	1	0,18	0,32	Boloria selene	4,11	1/1	disjunct	good
Nymphalidae	Brenthis ino	3	1	0,47	0,96	Argynnis aglaja	5,23	1/1	continuous	good
Nymphalidae	Coenonympha glycerion	3	1	0,48	0,82	Coenonympha tullia	8,12	1/1	continuous	good
Nymphalidae	Coenonympha pamphilus	3	1	0,28	0,46	Coenonympha tullia	8,14	1/1	continuous	good
Nymphalidae	Coenonympha tullia	3	1	0,51	0,77	Coenonympha glycerion	8,12	1/1	continuous	good
Nymphalidae	Erebia ligea	7	1	0,08	0,31	Erebia pandrose	8,48	1/1	continuous	good
Nymphalidae	Erebia pandrose	3	1	0,08	0,15	Erebia ligea	8,48	1/1	disjunct	good
Nymphalidae	Euphydryas aurinia	1	1	0,31	0,31	Polygonia c-album	10,33	1/1	continuous	good
Nymphalidae	Lasiommata maera	3	1	0,69	1,4	Lasiommata petropolitana	5,72	1/1	continuous	good
Nymphalidae	Lasiommata petropolitana	3	1	0,08	0,15	Lasiommata maera	5,72	1/1	continuous	good
Nymphalidae	Maniola jurtina	3	1	1,98	3,53	Aphantopus hyperantus	8,45	2/1	continuous	good
Nymphalidae	Melitaea athalia	3	2	3,15	5,31	Melitaea diamina	6,1	2/0	continuous	good
Nymphalidae	Melitaea diamina	2	2	0	0	Melitaea athalia	6,1	1/1	continuous	good
Nymphalidae	Pararge aegeria	3	1	0	0	Lasiommata maera	9,96	1/1	continuous	good
Nymphalidae	Polygonia c-album	3	1	0,18	0,32	Aglais io	5,63	1/1	continuous	good
Nymphalidae	Vanessa atalanta	3	1	0,08	0,16	Vanessa cardui	5,76	1/1	migrating	good
Nymphalidae	Vanessa cardui	3	1	0,11	0,16	Vanessa atalanta	5,76	1/1	migrating	good
Oecophoridae	Bisigna procerella	4	1	0,2	0,55	Papilio machaon	10,76	1/1	continuous	

Oecophoridae	Borkhausenia fuscescens	5	2	0,6	1,39	Xanthorhoe designata	11,55	1/1	continuous	
Oecophoridae	Crassa tinctella	3	1	0,16	0,31	Scopula floslactata	9,39	1/1	continuous	
Oecophoridae	Denisia similella	4	1	0,17	0,48	Denisia stipella	7,53	1/1	continuous	
Oecophoridae	Denisia stipella	4	3	1,04	3	Denisia similella	7,53	1/1	continuous	
Oecophoridae	Harpella forficella	4	1	0	0	Metaxmeste schrankiana	9,61	1/1	continuous	
Oecophoridae	Hofmannophila pseudospretella	3	2	0,06	0,16	Ethmia pusiella	9,96	1/1	continuous	
Oecophoridae	Oecophora bractella	4	1	0	0	Denisia stipella	9,64	1/1	continuous	
Oecophoridae	Pleurota bicostella	3	1	0,31	0,62	Diloba caeruleocephala	10,84	1/1	continuous	
Oecophoridae	Stathmopoda pedella	4	1	0,09	0,17	Catocala nupta	8,06	1/1	continuous	
Opostegidae	Pseudopostega crepusculella	3	1	1,58	3,16	Swammerdamia compunctella	14,64	2/0	continuous	
Papilionidae	Papilio machaon	3	1	0,69	0,92	Bisigna procerella	10,76	1/1	continuous	
Papilionidae	Parnassius mnemosyne	2	1	2,09	3,14	Zelleria hepariella	8,83	2/0	continuous	
Pieridae	Anthocharis cardamines	3	1	0,28	0,46	Gonepteryx rhamni	11,55	1/1	continuous	good
Pieridae	Aporia crataegi	3	1	0,26	0,31	Brenthis ino	11,11	1/1	continuous	good
Pieridae	Colias hyale	2	1	0,41	0,62	Colias palaeno	4,77	1/1	continuous	good
Pieridae	Colias palaeno	5	1	0	0	Colias hyale	4,77	1/1	continuous	good
Pieridae	Gonepteryx rhamni	3	1	0,08	0,15	Callophrys rubi	9,09	1/1	continuous	good
Pieridae	Leptidea juvernica	1	3	0,16	0,33	Leptidea sinapis	2,67	1/1	continuous	good
Pieridae	Leptidea sinapis	6	1	0,07	0,16	Leptidea juvernica	2,67	1/1	continuous	good
Pieridae	Pieris brassicae	3	1	0,31	0,61	Pieris napi	10,32	1/1	continuous	good
Pieridae	Pieris napi	3	1	1,82	2,66	Pieris rapae	8,06	2/1	continuous	good
Pieridae	Pieris rapae	3	1	0,81	1,28	Pieris napi	8,06	1/1	continuous	good
Plutellidae	Plutella xylostella	15	2	0,66	2,18	Yponomeuta plumbella	11,2	1/1	continuous	
Plutellidae	Rhigognostis senilella	3	2	0,52	1,08	Yponomeuta plumbella	10,32	1/1	fragmented	
Praydidae	Prays fraxinella	6	1	0,45	0,93	Prays ruficeps	2,99	1/1	continuous	
Praydidae	Prays ruficeps	9	2	0,09	0,31	Prays fraxinella	2,99	1/1	continuous	
Prodoxidae	Incurvaria masculella	3	1	0,16	0,32	Aleimma loeflingiana	15,15	1/1	continuous	
Prodoxidae	Lampronia corticella	3	1	0,32	0,71	Ethmia pusiella	14,09	1/1	continuous	
Prodoxidae	Lampronia rupella	3	2	0,9	1,41	Ethmia pusiella	15,63	1/1	continuous	
Psychidae	Psyche casta	19	2	0,72	2,27	Psyche crassiorella	7,99	1/1	continuous	poor

Psychidae	Psyche crassiorella	6	1	1,95	4,28	Psyche casta	7,99	2/1	continuous	poor
Psychidae	Sterrhopterix standfussi	4	1	0,12	0,31	Exoteleia dodecella	16,44	1/1	fragmented	poor
Psychidae	Taleporia tubulosa	8	1	0,91	2,93	Aleimma loeflingiana	14,83	1/1	continuous	poor
Pterophoridae	Adaina microdactyla	2	2	0,08	0,15	Xanthorhoe montanata	11,35	1/1	continuous	
Pterophoridae	Amblyptilia punctidactyla	4	2	1,66	3,46	Stenoptilia pterodactyla	10,93	3/0	continuous	
Pterophoridae	Buckleria paludum	4	1	0,06	0,16	Stenoptilia pterodactyla	11,04	1/1	continuous	
Pterophoridae	Emmelina monodactyla	3	2	0,06	0,15	Recurvaria leucatella	10,86	1/1	continuous	
Pterophoridae	Hellinsia osteodactylus	4	1	0,45	1,24	Xanthorhoe spadicearia	14,2	1/1	continuous	
Pterophoridae	Hellinsia tephradactyla	3	1	0,19	0,32	Merrifieldia leucodactyla	12,27	1/1	continuous	
Pterophoridae	Marasmarcha lunaedactyla	3	1	1,49	2,98	Syncopacma sangiella	12,09	2/0	fragmented	
Pterophoridae	Merrifieldia leucodactyla	3	1	0,31	0,62	Agonopterix kaekeritziana	9,44	1/1	continuous	
Pterophoridae	Oidaematophorus lithodactyla	3	1	0,15	0,31	Merrifieldia leucodactyla	11,37	1/1	continuous	
Pterophoridae	Oidaematophorus rogenhoferi	3	1	0,46	0,77	Thyatira batis	12,84	1/1	disjunct	
Pterophoridae	Platyptilia calodactyla	10	2	1,59	7,33	Platyptilia nemoralis	5,2	3/1	continuous	
Pterophoridae	Platyptilia gonodactyla	3	2	0,12	0,31	Platyptilia nemoralis	4,94	1/1	continuous	
Pterophoridae	Platyptilia nemoralis	1	1	0,15	0,15	Platyptilia gonodactyla	4,94	1/1	fragmented	
Pterophoridae	Stenoptilia pterodactyla	4	1	0,12	0,32	Amblyptilia punctidactyla	10,93	1/1	continuous	
Pyralidae	Acrobasis advenella	4	1	0,49	0,93	Evergestis pallidata	9,63	1/1	continuous	
Pyralidae	Aphomia sociella	4	1	1,24	2,04	Carpatolechia alburnella	11,37	1/1	continuous	
Pyralidae	Assara terebrella	4	1	1,85	4,29	Oncocera semirubella	12,27	2/0	continuous	
Pyralidae	Catastia marginea	3	1	0	0	Sciota adelphella	7,45	1/1	disjunct	
Pyralidae	Cryptoblabes bistriga	3	2	0,18	0,31	Chionodes fumatella	12,12	1/1	continuous	
Pyralidae	Dioryctria abietella	3	1	0	0	Dioryctria simplicella	4,84	1/1	continuous	
Pyralidae	Dioryctria simplicella	4	2	0	0	Dioryctria abietella	4,84	1/1	continuous	
Pyralidae	Dioryctria sylvestrella	3	2	0,12	0,31	Dioryctria abietella	9,29	1/1	continuous	
Pyralidae	Ephestia elutella	3	1	0,69	1,08	Vitula biviella	11,04	1/1	continuous	
Pyralidae	Hypochalcia ahenella	4	1	0,46	0,77	Phycita roborella	8,26	1/1	continuous	
Pyralidae	Hypsopygia costalis	3	1	0,35	0,58	Hypsopygia glaucinalis	10,86	1/1	continuous	
Pyralidae	Hypsopygia glaucinalis	3	1	0	0	Hypsopygia costalis	10,86	1/1	continuous	
Pyralidae	Matilella fusca	3	1	0,08	0,15	Sciota adelphella	7,11	1/1	continuous	
Pyralidae	Nephopterix angustella	1	1	0,31	0,31	Oncocera semirubella	10,49	1/1	continuous	

Pyralidae	Oncocera semirubella	3	1	0,08	0,16	Catastia marginea	8,06	1/1	continuous	
Pyralidae	Phycita roborella	4	1	0,52	1,08	Dioryctria abietella	7,9	1/1	continuous	
Pyralidae	Phycitodes binaevella	3	1	0,66	0,92	Phycitodes saxicola	10,11	1/1	continuous	
Pyralidae	Phycitodes saxicola	3	1	0,11	0,17	Phycitodes binaevella	10,11	1/1	fragmented	
Pyralidae	Plodia interpunctella	2	1	0,32	0,5	Vitula biviella	9,75	1/1	continuous	
Pyralidae	Pyralis farinalis	2	1	2,55	3,85	Hypsopygia costalis	13,35	2/0	continuous	
Pyralidae	Sciota adelphella	3	1	0	0	Sciota hostilis	5,73	1/1	continuous	
Pyralidae	Sciota hostilis	3	2	0,12	0,31	Sciota adelphella	5,73	1/1	continuous	
Pyralidae	Vitula biviella	2	1	0,11	0,16	Plodia interpunctella	9,75	1/1	continuous	
Roeslerstammiidae	Roeslerstammia erxlebella	5	1	0,05	0,17	Oecophora bractella	11,19	1/1	continuous	
Saturniidae	Aglia tau	3	1	0	0	Rivula sericealis	9,97	1/1	continuous	good
Saturniidae	Saturnia pavonia	2	2	0,28	0,46	Ethmia pusiella	10,98	1/1	continuous	good
Scythrididae	Scythris laminella	3	1	0,15	0,31	Batrachedra pinicolella	9,53	1/1	continuous	
Scythrididae	Scythris noricella	1	1	0,15	0,15	Parnassius mnemosyne	9,79	1/1	disjunct	
Scythrididae	Scythris obscurella	3	1	0,7	1,41	Tholera decimalis	10,77	1/1	disjunct	
Sesiidae	Bembecia ichneumoniformis	3	1	0,08	0,15	Synanthedon formicaeformis	12,11	1/1	continuous	
Sesiidae	Paranthrene tabaniformis	3	1	1,24	2,34	Xanthorhoe montanata	13,37	2/0	continuous	
Sesiidae	Pennisetia hylaeiformis	4	1	0	0	Teleiodes wagae	13,17	1/1	continuous	
Sesiidae	Synanthedon formicaeformis	6	1	0,85	2,6	Synanthedon spheciformis	11,2	2/0	continuous	
Sesiidae	Synanthedon spheciformis	6	1	0,31	0,79	Synanthedon tipuliformis	11,14	1/1	continuous	
Sesiidae	Synanthedon tipuliformis	2	1	0,72	0,92	Synanthedon spheciformis	11,14	1/1	continuous	
Sphingidae	Deilephila elpenor	3	1	0	0	Hyles gallii	6,74	1/1	continuous	good
Sphingidae	Hemaris fuciformis	3	1	0	0	Hemaris tityus	5,17	1/1	continuous	good
Sphingidae	Hemaris tityus	3	1	0,08	0,15	Hemaris fuciformis	5,17	1/1	continuous	good
Sphingidae	Hyles gallii	3	2	0,18	0,46	Deilephila elpenor	6,74	1/1	continuous	good
Sphingidae	Laothoe populi	4	1	0	0	Macaria fusca	10,31	1/1	continuous	good
Sphingidae	Mimas tiliae	3	1	1,25	2,5	Hemaris tityus	10,86	2/0	continuous	good
Sphingidae	Sphinx ligustri	2	1	0,2	0,31	Sphinx pinastri	4,42	1/1	continuous	good
Sphingidae	Sphinx pinastri	3	1	0,39	0,77	Sphinx ligustri	4,42	1/1	continuous	good
Tineidae	Agnathosia mendicella	4	1	0,12	0,31	Ancylis apicella	13,19	1/1	continuous	poor
Tineidae	Archinemapogon yildizae	3	1	0,08	0,15	Nemapogon cloacella	9,64	1/1	continuous	poor

Tineidae	Monopis laevigella	14	1	1,94	5,25	Monopis monachella	12,09	2/1	continuous	poor
Tineidae	Monopis monachella	4	1	0	0	Monopis laevigella	12,09	1/1	continuous	poor
Tineidae	Monopis obviella	3	2	0,09	0,15	Monopis laevigella	13,39	1/1	continuous	poor
Tineidae	Montescardia tessulatellus	3	1	0,4	0,77	Cabera pusaria	12,54	1/1	continuous	poor
Tineidae	Nemapogon cloacella	4	1	1,34	2,18	Nemapogon wolffiella	6,27	2/1	continuous	poor
Tineidae	Nemapogon wolffiella	3	1	0	0	Nemapogon cloacella	6,27	1/1	continuous	poor
Tineidae	Tinea pellionella	3	3	0,93	2,03	Cosmia affinis	12,81	1/1	continuous	poor
Tineidae	Tinea semifulvella	4	1	0,06	0,15	Neofaculta infernella	13,01	1/1	continuous	poor
Tineidae	Tinea trinotella	2	2	0,8	1,24	Diurnea lipsiella	13,91	1/1	continuous	poor
Tischeriidae	Coptotriche angusticollella	3	1	0	0	Coptotriche marginea	11,55	1/1	continuous	poor
Tischeriidae	Coptotriche marginea	4	1	0,25	0,61	Coptotriche angusticollella	11,55	1/1	continuous	poor
Tischeriidae	Tischeria ekebladella	5	1	0,06	0,17	Scoparia ambigualis	13,7	1/1	continuous	poor
Tortricidae	Acleris bergmanniana	3	1	0	0	Acleris forsskaleana	6,27	1/1	continuous	poor
Tortricidae	Acleris ferrugana	3	1	0,39	0,79	Acleris forsskaleana	5,87	1/1	continuous	poor
Tortricidae	Acleris forsskaleana	3	1	0,55	1,08	Acleris ferrugana	5,87	1/1	continuous	poor
Tortricidae	Acleris hastiana	9	1	0,5	1,1	Acleris umbrana	6,23	1/1	continuous	poor
Tortricidae	Acleris laterana	5	1	0,57	1,39	Acleris umbrana	7,82	1/1	continuous	poor
Tortricidae	Acleris maccana	5	3	0,04	0,15	Acleris ferrugana	6,39	1/1	continuous	poor
Tortricidae	Acleris schalleriana	3	2	0,09	0,15	Acleris variegana	6,9	1/1	continuous	poor
Tortricidae	Acleris shepherdana	2	1	0	0	Acleris umbrana	7,45	1/1	continuous	poor
Tortricidae	Acleris umbrana	3	1	0	0	Acleris variegana	5,69	1/1	continuous	poor
Tortricidae	Acleris variegana	2	1	1,35	2,03	Acleris umbrana	5,69	1/1	continuous	poor
Tortricidae	Adoxophyes orana	10	1	0,05	0,32	Aphelia paleana	9,93	1/1	continuous	poor
Tortricidae	Aethes cnicana	13	3	0,07	0,31	Aethes rubigana	0,77	1/1	continuous	poor
Tortricidae	Aethes hartmanniana	4	1	1,71	2,77	Aethes cnicana	8,07	1/1	continuous	poor
Tortricidae	Aethes rubigana	10	1	0,4	2,26	Aethes cnicana	0,77	1/1	continuous	poor
Tortricidae	Aethes rutilana	10	1	1,05	2,03	Gynnidomorpha alismana	8,75	2/1	continuous	poor
Tortricidae	Aethes smeathmanniana	4	1	0,11	0,31	Aethes cnicana	6,34	1/1	continuous	poor
Tortricidae	Agapeta hamana	2	1	0,1	0,15	Agapeta zoegana	6,39	1/1	continuous	poor
Tortricidae	Agapeta zoegana	5	1	1,71	5,1	Agapeta hamana	6,39	2/0	continuous	poor
Tortricidae	Aleimma loeflingiana	3	1	0,08	0,16	Tortrix viridana	5,87	1/1	continuous	poor

Tortricidae	Ancylis apicella	4	1	0,62	1,49	Ancylis badiana	4,99	2/0	continuous	poor
Tortricidae	Ancylis badiana	22	2	1,29	2,94	Ancylis apicella	4,99	3/2	continuous	poor
Tortricidae	Ancylis diminutana	5	2	0	0	Eana penziana	10,15	1/1	continuous	poor
Tortricidae	Ancylis laetana	4	1	0,12	0,19	Ancylis mitterbacheriana	8,26	1/1	continuous	poor
Tortricidae	Ancylis mitterbacheriana	3	3	0,47	0,79	Ancylis laetana	8,26	1/1	continuous	poor
Tortricidae	Ancylis myrtillana	3	1	0,24	0,48	Ancylis apicella	7,24	1/1	continuous	poor
Tortricidae	Ancylis unculana	3	1	1,44	3	Ancylis apicella	6,49	2/0	continuous	poor
Tortricidae	Aphelia paleana	15	2	1,09	2,76	Aphelia unitana	3,3	3/1	continuous	poor
Tortricidae	Aphelia unitana	7	2	1,32	3,26	Aphelia paleana	3,3	2/0	continuous	poor
Tortricidae	Aphelia viburniana	5	1	1,32	2,34	Aphelia unitana	6,56	2/1	continuous	poor
Tortricidae	Apotomis capreana	6	2	0,28	0,93	Apotomis sauciana	0,62	1/1	continuous	poor
Tortricidae	Apotomis infida	5	1	0,46	0,77	Apotomis sauciana	1,7	1/1	continuous	poor
Tortricidae	Apotomis sauciana	19	1	0,2	0,92	Apotomis capreana	0,62	1/1	continuous	poor
Tortricidae	Archips oporana	3	1	0	0	Archips podana	7,8	1/1	continuous	poor
Tortricidae	Archips podana	5	1	0,08	0,15	Aphelia unitana	7,58	1/1	continuous	poor
Tortricidae	Archips rosana	3	2	0,91	1,61	Archips xylosteana	3,79	2/0	continuous	poor
Tortricidae	Archips xylosteana	3	1	0,49	0,77	Archips rosana	3,79	1/1	continuous	poor
Tortricidae	Argyroploce noricana	3	1	0	0	Metendothenia atropunctana	5,9	1/1	disjunct	poor
Tortricidae	Argyrotaenia ljungiana	4	1	0	0	Pandemis dumetana	8,24	1/1	continuous	poor
Tortricidae	Bactra lacteana	5	1	0,14	0,31	Bactra lancealana	3,94	1/1	continuous	poor
Tortricidae	Bactra lancealana	16	1	0,55	0,94	Bactra lacteana	3,94	1/1	continuous	poor
Tortricidae	Capua vulgana	4	1	0,09	0,16	Celypha rivulana	9,45	1/1	continuous	poor
Tortricidae	Celypha cespitana	8	1	0,46	0,95	Celypha rurestrana	4,44	1/1	continuous	poor
Tortricidae	Celypha lacunana	12	2	0,72	1,8	Piniphila bifasciana	6,12	2/1	continuous	poor
Tortricidae	Celypha rivulana	3	1	0,64	1,08	Piniphila bifasciana	5,23	1/1	continuous	poor
Tortricidae	Celypha rurestrana	3	1	0,08	0,15	Celypha cespitana	4,44	1/1	continuous	poor
Tortricidae	Clepsis rurinana	3	1	0	0	Ancylis apicella	8,84	1/1	continuous	poor
Tortricidae	Cnephasia alticolana	5	1	0	0	Cnephasia asseclana	2,07	1/1	continuous	poor
Tortricidae	Cnephasia asseclana	19	3	0,09	0,33	Cnephasia alticolana	2,07	1/1	continuous	poor
Tortricidae	Cnephasia stephensiana	15	2	0,02	0,16	Cnephasia asseclana	9	1/1	continuous	poor
Tortricidae	Cochylidia subroseana	3	2	0,21	0,46	Cochylis flaviciliana	6,75	1/1	continuous	poor

Tortricidae	Cochylis dubitana	8	1	0,18	0,46	Cochylis nana	7,66	1/1	continuous	poor
Tortricidae	Cochylis flaviciliana	3	1	1,3	2,6	Cochylidia subroseana	6,75	2/0	continuous	poor
Tortricidae	Cochylis nana	3	1	0,21	0,46	Cochylis dubitana	7,66	1/1	continuous	poor
Tortricidae	Cochylis pallidana	6	3	0,83	3,3	Cochylidia subroseana	8,6	2/1	continuous	poor
Tortricidae	Cydia duplicana	3	1	0,8	1,59	Cydia illutana	6,89	1/1	continuous	poor
Tortricidae	Cydia illutana	5	3	0	0	Cydia strobilella	5,27	1/1	continuous	poor
Tortricidae	Cydia inquinatana	3	1	0	0	Cydia illutana	6,89	1/1	fragmented	poor
Tortricidae	Cydia nigricana	9	1	0,75	1,45	Cydia succedana	6,38	1/1	continuous	poor
Tortricidae	Cydia pomonella	3	1	0,88	1,57	Cydia illutana	6,68	1/1	continuous	poor
Tortricidae	Cydia splendana	4	1	1,21	1,87	Cydia illutana	5,8	2/1	continuous	poor
Tortricidae	Cydia strobilella	3	1	0,36	0,62	Cydia illutana	5,27	1/1	continuous	poor
Tortricidae	Cydia succedana	5	1	1,29	2,18	Cydia nigricana	6,38	2/1	continuous	poor
Tortricidae	Cymolomia hartigiana	3	1	0,78	1,55	Phiaris schulziana	8,63	2/0	continuous	poor
Tortricidae	Dichelia histrionana	3	1	0	0	Syndemis musculana	7,61	1/1	continuous	poor
Tortricidae	Dichrorampha alpinana	3	1	0,85	1,71	Dichrorampha simpliciana	5,74	2/1	continuous	poor
Tortricidae	Dichrorampha simpliciana	3	2	1,03	1,61	Dichrorampha alpinana	5,74	1/1	continuous	poor
Tortricidae	Doloploca punctulana	1	1	0,17	0,17	Eana penziana	5,2	1/1	continuous	poor
Tortricidae	Eana argentana	3	1	0,16	0,32	Eana osseana	3,14	1/1	continuous	poor
Tortricidae	Eana incanana	3	1	0	0	Eana penziana	3,51	1/1	continuous	poor
Tortricidae	Eana osseana	3	1	0,33	0,46	Eana argentana	3,14	1/1	continuous	poor
Tortricidae	Eana penziana	4	2	0,49	0,98	Eana incanana	3,51	1/1	continuous	poor
Tortricidae	Endothenia ericetana	4	1	0,06	0,16	Endothenia quadrimaculana	6,28	1/1	continuous	poor
Tortricidae	Endothenia marginana	11	2	0,07	0,31	Endothenia nigricostana	7,1	1/1	continuous	poor
Tortricidae	Endothenia nigricostana	3	1	0,08	0,15	Endothenia quadrimaculana	7,1	1/1	continuous	poor
Tortricidae	Endothenia quadrimaculana	4	1	0,09	0,15	Endothenia ericetana	6,28	1/1	continuous	poor
Tortricidae	Epiblema grandaevana	6	1	1,63	4,94	Epiblema scutulana	8,11	2/0	continuous	poor
Tortricidae	Epiblema scutulana	7	1	1,42	3,34	Pelochrista caecimaculana	7,4	2/1	continuous	poor
Tortricidae	Epiblema sticticana	4	1	0	0	Epiblema grandaevana	8,31	1/1	continuous	poor
Tortricidae	Epinotia abbreviana	4	1	0,31	0,65	Epinotia granitana	7,17	1/1	continuous	poor
Tortricidae	Epinotia bilunana	3	1	0	0	Epinotia tetraquetrana	6,24	1/1	continuous	poor
Tortricidae	Epinotia cruciana	4	1	0,96	1,87	Epinotia mercuriana	4,12	1/1	continuous	poor

Tortricidae	Epinotia demarniana	4	1	0	0	Epinotia tetraquetrana	6,77	1/1	continuous	poor
Tortricidae	Epinotia granitana	4	1	0	0	Epinotia tedella	5,82	1/1	continuous	poor
Tortricidae	Epinotia immundana	8	1	2,91	5,29	Epinotia bilunana	6,94	2/1	continuous	poor
Tortricidae	Epinotia mercuriana	3	1	1,36	2,66	Epinotia cruciana	4,12	2/0	disjunct	poor
Tortricidae	Epinotia nanana	5	1	0,13	0,33	Epinotia tetraquetrana	6,86	1/1	continuous	poor
Tortricidae	Epinotia nisella	9	1	0,98	2,19	Epinotia bilunana	8,1	1/1	continuous	poor
Tortricidae	Epinotia ramella	6	1	3,09	5,29	Epinotia bilunana	9,38	2/1	continuous	poor
Tortricidae	Epinotia subocellana	3	1	0,1	0,16	Epinotia abbreviana	7,66	1/1	continuous	poor
Tortricidae	Epinotia tedella	3	1	0,53	0,79	Epinotia granitana	5,82	1/1	continuous	poor
Tortricidae	Epinotia tetraquetrana	4	2	0,2	0,31	Epinotia bilunana	6,24	1/1	continuous	poor
Tortricidae	Epinotia trigonella	5	1	1,17	2,35	Epinotia abbreviana	8,74	1/1	continuous	poor
Tortricidae	Eriopsela quadrana	3	1	0,99	1,9	Epinotia tetraquetrana	10,6	1/1	continuous	poor
Tortricidae	Eucosma cana	9	1	0,25	0,96	Eucosma conterminana	6,75	1/1	continuous	poor
Tortricidae	Eucosma conterminana	4	1	0,31	0,62	Eucosma cana	6,75	1/1	continuous	poor
Tortricidae	Eucosma hohenwartiana	30	2	0,38	1,39	Eucosma cana	7,06	1/1	continuous	poor
Tortricidae	Eucosmomorpha albersana	3	1	0,08	0,16	Phiaris schulziana	9,45	1/1	continuous	poor
Tortricidae	Eudemis porphyrana	3	1	0	0	Doloploca punctulana	7,45	1/1	continuous	poor
Tortricidae	Eulia ministrana	8	1	0,44	1,48	Rhopobota naevana	8,7	1/1	continuous	poor
Tortricidae	Eupoecilia ambiguella	3	1	0,26	0,31	Eupoecilia angustana	8	1/1	continuous	poor
Tortricidae	Eupoecilia angustana	3	1	0,08	0,18	Eupoecilia ambiguella	8	1/1	continuous	poor
Tortricidae	Falseuncaria ruficiliana	3	2	0,68	1,08	Cochylidia subroseana	7,05	1/1	continuous	poor
Tortricidae	Grapholita lobarzewskii	1	1	0,15	0,15	Grapholita tenebrosana	7,58	1/1	fragmented	poor
Tortricidae	Grapholita tenebrosana	26	1	2,77	5,94	Grapholita lobarzewskii	7,58	2/1	continuous	poor
Tortricidae	Gynnidomorpha alismana	4	1	0,87	2,18	Archips rosana	7,73	2/0	continuous	poor
Tortricidae	Gypsonoma dealbana	3	2	1,52	2,35	Rhopobota naevana	7,86	2/0	continuous	poor
Tortricidae	Gypsonoma sociana	5	1	0,25	0,62	Gypsonoma dealbana	9,53	1/1	continuous	poor
Tortricidae	Hedya nubiferana	3	1	0,62	1,08	Hedya pruniana	6,22	1/1	continuous	poor
Tortricidae	Hedya ochroleucana	3	1	0,32	0,66	Celypha rivulana	10	1/1	continuous	poor
Tortricidae	Hedya pruniana	3	1	0	0	Hedya nubiferana	6,22	1/1	continuous	poor
Tortricidae	Lathronympha strigana	5	1	0,05	0,16	Ancylis myrtillana	11,2	1/1	continuous	poor
Tortricidae	Lobesia reliquana	8	1	0,12	0,32	Epinotia abbreviana	9,29	1/1	continuous	poor

Tortricidae	Metendothenia atropunctana	3	1	0,08	0,15	Argyroploce noricana	5,9	1/1	continuous	poor
Tortricidae	Notocelia cynosbatella	4	1	0,84	1,55	Notocelia uddmanniana	7,82	1/1	continuous	poor
Tortricidae	Notocelia rosaecolana	3	1	0	0	Epinotia abbreviana	10,4	1/1	continuous	poor
Tortricidae	Notocelia tetragonana	3	1	0	0	Epiblema scutulana	8,95	1/1	continuous	poor
Tortricidae	Notocelia uddmanniana	3	1	0,28	0,53	Zeiraphera isertana	7,42	1/1	continuous	poor
Tortricidae	Olethreutes arcuella	3	1	0,08	0,15	Phiaris palustrana	6,32	1/1	continuous	poor
Tortricidae	Orthotaenia undulana	8	1	0,11	0,46	Celypha rivulana	6,15	1/1	continuous	poor
Tortricidae	Pammene fasciana	3	1	0	0	Pammene ignorata	5,74	1/1	continuous	poor
Tortricidae	Pammene ignorata	7	1	0,88	2,07	Pammene fasciana	5,74	1/1	continuous	poor
Tortricidae	Pammene ochsenheimeriana	3	1	0	0	Pammene fasciana	8,79	1/1	continuous	poor
Tortricidae	Pandemis cerasana	3	1	0,38	0,77	Pandemis cinnamomeana	5,56	1/1	continuous	poor
Tortricidae	Pandemis cinnamomeana	4	1	0,21	0,46	Pandemis dumetana	5,56	1/1	continuous	poor
Tortricidae	Pandemis dumetana	3	1	0	0	Pandemis cinnamomeana	5,56	1/1	continuous	poor
Tortricidae	Pelochrista caecimaculana	3	1	0,15	0,31	Epiblema scutulana	7,4	1/1	continuous	poor
Tortricidae	Phalonidia gilvicomana	2	1	1,66	2,58	Gynnidomorpha alismana	10,64	1/1	continuous	poor
Tortricidae	Phiaris bipunctana	4	1	0,06	0,15	Celypha rivulana	6,24	1/1	continuous	poor
Tortricidae	Phiaris dissolutana	3	1	0,23	0,46	Piniphila bifasciana	6,93	1/1	continuous	poor
Tortricidae	Phiaris micana	3	2	0,49	0,93	Phiaris palustrana	1,77	1/1	continuous	poor
Tortricidae	Phiaris palustrana	3	1	0,32	0,66	Phiaris micana	1,77	1/1	continuous	poor
Tortricidae	Phiaris schulziana	16	1	0,45	1,24	Celypha rivulana	6,24	1/1	continuous	poor
Tortricidae	Phtheochroa inopiana	3	1	0	0	Gynnidomorpha alismana	8,92	1/1	continuous	poor
Tortricidae	Piniphila bifasciana	3	1	0,54	1,08	Celypha rivulana	5,23	1/1	continuous	poor
Tortricidae	Pristerognatha penthinana	2	1	0,15	0,31	Piniphila bifasciana	7,91	1/1	continuous	poor
Tortricidae	Pseudargyrotoza conwagana	3	1	0,21	0,33	Celypha rivulana	7,49	1/1	continuous	poor
Tortricidae	Pseudohermenias abietana	3	1	0,56	0,93	Celypha rivulana	7,56	1/1	continuous	poor
Tortricidae	Pseudosciaphila branderiana	3	1	0,08	0,15	Hedya pruniana	8,59	1/1	continuous	poor
Tortricidae	Rhopobota naevana	14	2	0,16	0,47	Rhopobota stagnana	5,51	1/1	continuous	poor
Tortricidae	Rhopobota stagnana	3	1	0,31	0,62	Rhopobota naevana	5,51	1/1	continuous	poor
Tortricidae	Rhyacionia pinicolana	3	3	0	0	Zeiraphera ratzeburgiana	11,33	1/1	continuous	poor
Tortricidae	Rhyacionia pinivorana	4	1	0,18	0,46	Epinotia abbreviana	10,01	1/1	continuous	poor
Tortricidae	Spatalistis bifasciana	2	1	0	0	Acleris forsskaleana	8,93	1/1	continuous	poor

Tortricidae	Spilonota ocellana	10	2	0,13	0,33	Eucosma cana	11,2	1/1	continuous	poor
Tortricidae	Syndemis musculana	4	1	0,25	0,32	Archips rosana	7,21	1/1	continuous	poor
Tortricidae	Tortrix viridana	3	1	0,21	0,33	Aleimma loeflingiana	5,87	1/1	continuous	poor
Tortricidae	Zeiraphera isertana	3	1	0,08	0,16	Zeiraphera ratzeburgiana	4,76	1/1	continuous	poor
Tortricidae	Zeiraphera ratzeburgiana	4	1	0,5	1,24	Zeiraphera isertana	4,76	2/0	continuous	poor
Yponomeutidae	Cedestis gysseleniella	3	1	0,41	0,77	Yponomeuta evonymella	9,6	1/1	continuous	
Yponomeutidae	Ocnerostoma friesei	4	1	0,25	0,48	Rheumaptera subhastata	11,77	1/1	continuous	
Yponomeutidae	Swammerdamia compunctella	3	1	0,08	0,18	Zelleria hepariella	9,21	1/1	continuous	
Yponomeutidae	Yponomeuta evonymella	6	1	0,12	0,46	Yponomeuta malinellus	1,55	1/1	continuous	
Yponomeutidae	Yponomeuta malinellus	5	2	0,31	0,62	Yponomeuta evonymella	1,55	1/1	continuous	
Yponomeutidae	Yponomeuta plumbella	3	1	1,08	1,08	Yponomeuta evonymella	8,41	1/1	continuous	
Yponomeutidae	Yponomeuta sedella	3	1	0,15	0,31	Yponomeuta evonymella	8,7	1/1	continuous	
Yponomeutidae	Zelleria hepariella	3	2	0	0	Parnassius mnemosyne	8,83	1/1	continuous	
Ypsolophidae	Ypsolopha falcella	3	1	0,15	0,31	Ypsolopha nemorella	4,75	1/1	continuous	
Ypsolophidae	Ypsolopha nemorella	3	1	0,49	0,77	Ypsolopha falcella	4,75	1/1	continuous	
Ypsolophidae	Ypsolopha ustella	3	2	0,43	0,66	Ypsolopha falcella	9,27	1/1	continuous	
Zygaenidae	Adscita statices	6	1	0,3	0,64	Anania crocealis	10,84	1/1	continuous	
Zygaenidae	Zygaena exulans	3	1	0,93	1,86	Zygaena viciae	7,07	2/0	disjunct	
Zygaenidae	Zygaena filipendulae	5	1	0,52	0,94	Zygaena lonicerae	5,98	1/1	continuous	
Zygaenidae	Zygaena lonicerae	3	1	0,94	1,87	Zygaena filipendulae	5,98	1/1	continuous	
Zygaenidae	Zygaena viciae	5	1	1,46	5,11	Zygaena exulans	7,07	2/1	continuous	
