# Supplementary material for: Testing DNA Barcode Performance in 1000 Species of European Lepidoptera: Large Geographic Distances Have Small Genetic Impacts
Source: PLoS One. 2014 Dec 26;9(12):e115774. doi: 10.1371/journal.pone.0115774 (PMC4277373; doi:10.1371/journal.pone.0115774)
Supplement: S3 Appendix — Neighbor Joining Tree. Neighbor Joining Tree (BOLD-Aligner, Kimura 2 parameter) for 5777 barcoded specimens (>500 bp) representing 1004 species and 1137 BINs. (PDF) [file pone.0115774.s003.pdf]

BOLD TaxonID Tree

Title	:	Finland and Austria: Vorarlberg Lepidoptera [DS-FVLEP]
Date	:	20-August-2014
Data Type	:	Nucleotide
Distance Model	:	Kimura 2 Parameter
Marker	:	COI-5P
Codon Positions	:	1st, 2nd, 3rd
Labels	:	Country & Province, SampleID, Sequence Length, BIN uri
Filters	:	Length > 200
Colorization	:	[blue]=Stop Codons [red]=Contamination or misidentification

Sequence Count	:	5777
Species count	:	1004
Genus count	:	543
Family count	:	58
Unidentified	:	9

BIN Count	:	1137

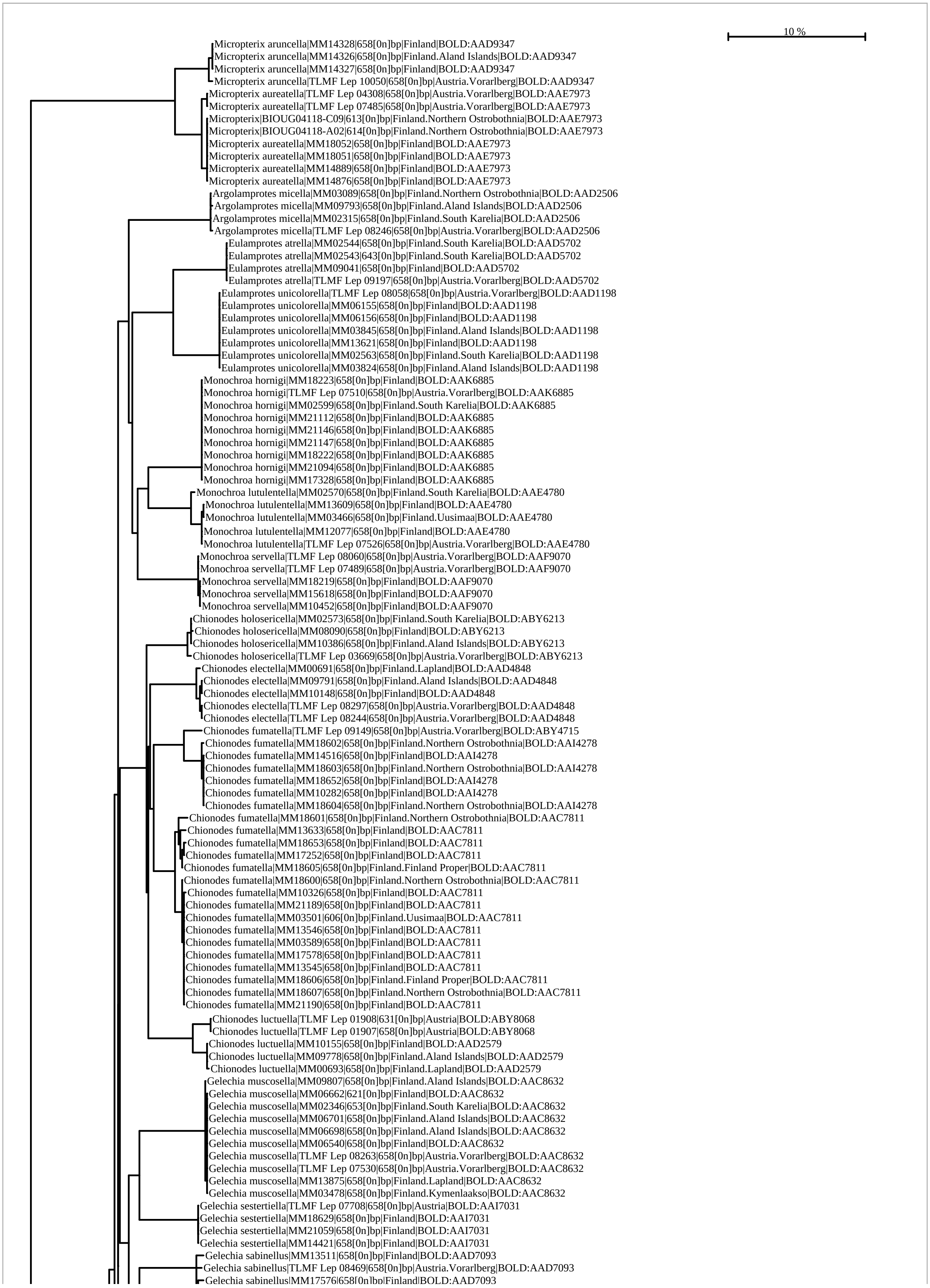
10 %
Micropterix aruncella|MM14328|658[0n]bp|Finland|BOLD:AAD9347
Micropterix aruncella|MM14326|658[0n]bp|Finland.Aland Islands|BOLD:AAD9347
Micropterix aruncella|MM14327|658[0n]bp|Finland|BOLD:AAD9347
Micropterix aruncella|TLMF Lep 10050|658[0n]bp|Austria.Vorarlberg|BOLD:AAD9347
Micropterix aureatella|TLMF Lep 04308|658[0n]bp|Austria.Vorarlberg|BOLD:AAE7973
Micropterix aureatella|TLMF Lep 07485|658[0n]bp|Austria.Vorarlberg|BOLD:AAE7973
Micropterix|BIOUG04118-C09|613[0n]bp|Finland.Northern Ostrobothnia|BOLD:AAE7973
Micropterix|BIOUG04118-A02|614[0n]bp|Finland.Northern Ostrobothnia|BOLD:AAE7973
Micropterix aureatella|MM18052|658[0n]bp|Finland|BOLD:AAE7973
Micropterix aureatella|MM18051|658[0n]bp|Finland|BOLD:AAE7973
Micropterix aureatella|MM14889|658[0n]bp|Finland|BOLD:AAE7973
Micropterix aureatella|MM14876|658[0n]bp|Finland|BOLD:AAE7973
Argolamprotes micella|MM03089|658[0n]bp|Finland.Northern Ostrobothnia|BOLD:AAD2506
Argolamprotes micella|MM09793|658[0n]bp|Finland.Aland Islands|BOLD:AAD2506
Argolamprotes micella|MM02315|658[0n]bp|Finland.South Karelia|BOLD:AAD2506
Argolamprotes micella|TLMF Lep 08246|658[0n]bp|Austria.Vorarlberg|BOLD:AAD2506
Eulamprotes atrella|MM02544|658[0n]bp|Finland.South Karelia|BOLD:AAD5702
Eulamprotes atrella|MM02543|643[0n]bp|Finland.South Karelia|BOLD:AAD5702
Eulamprotes atrella|MM09041|658[0n]bp|Finland|BOLD:AAD5702
Eulamprotes atrella|TLMF Lep 09197|658[0n]bp|Austria.Vorarlberg|BOLD:AAD5702
Eulamprotes unicolorella|TLMF Lep 08058|658[0n]bp|Austria.Vorarlberg|BOLD:AAD1198
Eulamprotes unicolorella|MM06155|658[0n]bp|Finland|BOLD:AAD1198
Eulamprotes unicolorella|MM06156|658[0n]bp|Finland|BOLD:AAD1198
Eulamprotes unicolorella|MM03845|658[0n]bp|Finland.Aland Islands|BOLD:AAD1198
Eulamprotes unicolorella|MM13621|658[0n]bp|Finland|BOLD:AAD1198
Eulamprotes unicolorella|MM02563|658[0n]bp|Finland.South Karelia|BOLD:AAD1198
Eulamprotes unicolorella|MM03824|658[0n]bp|Finland.Aland Islands|BOLD:AAD1198
Monochroa hornigi|MM18223|658[0n]bp|Finland|BOLD:AAK6885
Monochroa hornigi|TLMF Lep 07510|658[0n]bp|Austria.Vorarlberg|BOLD:AAK6885
Monochroa hornigi|MM02599|658[0n]bp|Finland.South Karelia|BOLD:AAK6885
Monochroa hornigi|MM21112|658[0n]bp|Finland|BOLD:AAK6885
Monochroa hornigi|MM21146|658[0n]bp|Finland|BOLD:AAK6885
Monochroa hornigi|MM21147|658[0n]bp|Finland|BOLD:AAK6885
Monochroa hornigi|MM18222|658[0n]bp|Finland|BOLD:AAK6885
Monochroa hornigi|MM21094|658[0n]bp|Finland|BOLD:AAK6885
Monochroa hornigi|MM17328|658[0n]bp|Finland|BOLD:AAK6885
Monochroa lutulentella|MM02570|658[0n]bp|Finland.South Karelia|BOLD:AAE4780
Monochroa lutulentella|MM13609|658[0n]bp|Finland|BOLD:AAE4780
Monochroa lutulentella|MM03466|658[0n]bp|Finland.Uusimaa|BOLD:AAE4780
Monochroa lutulentella|MM12077|658[0n]bp|Finland|BOLD:AAE4780
Monochroa lutulentella|TLMF Lep 07526|658[0n]bp|Austria.Vorarlberg|BOLD:AAE4780
Monochroa servella|TLMF Lep 08060|658[0n]bp|Austria.Vorarlberg|BOLD:AAF9070
Monochroa servella|TLMF Lep 07489|658[0n]bp|Austria.Vorarlberg|BOLD:AAF9070
Monochroa servella|MM18219|658[0n]bp|Finland|BOLD:AAF9070
Monochroa servella|MM15618|658[0n]bp|Finland|BOLD:AAF9070
Monochroa servella|MM10452|658[0n]bp|Finland|BOLD:AAF9070
Chionodes holosericella|MM02573|658[0n]bp|Finland.South Karelia|BOLD:ABY6213
Chionodes holosericella|MM08090|658[0n]bp|Finland|BOLD:ABY6213
Chionodes holosericella|MM10386|658[0n]bp|Finland.Aland Islands|BOLD:ABY6213
Chionodes holosericella|TLMF Lep 03669|658[0n]bp|Austria.Vorarlberg|BOLD:ABY6213
Chionodes electella|MM00691|658[0n]bp|Finland.Lapland|BOLD:AAD4848
Chionodes electella|MM09791|658[0n]bp|Finland.Aland Islands|BOLD:AAD4848
Chionodes electella|MM10148|658[0n]bp|Finland|BOLD:AAD4848
Chionodes electella|TLMF Lep 08297|658[0n]bp|Austria.Vorarlberg|BOLD:AAD4848
Chionodes electella|TLMF Lep 08244|658[0n]bp|Austria.Vorarlberg|BOLD:AAD4848
Chionodes fumatella|TLMF Lep 09149|658[0n]bp|Austria.Vorarlberg|BOLD:ABY4715
Chionodes fumatella|MM18602|658[0n]bp|Finland.Northern Ostrobothnia|BOLD:AAI4278
Chionodes fumatella|MM14516|658[0n]bp|Finland|BOLD:AAI4278
Chionodes fumatella|MM18603|658[0n]bp|Finland.Northern Ostrobothnia|BOLD:AAI4278
Chionodes fumatella|MM18652|658[0n]bp|Finland|BOLD:AAI4278
Chionodes fumatella|MM10282|658[0n]bp|Finland|BOLD:AAI4278
Chionodes fumatella|MM18604|658[0n]bp|Finland.Northern Ostrobothnia|BOLD:AAI4278
Chionodes fumatella|MM18601|658[0n]bp|Finland.Northern Ostrobothnia|BOLD:AAC7811
Chionodes fumatella|MM13633|658[0n]bp|Finland|BOLD:AAC7811
Chionodes fumatella|MM18653|658[0n]bp|Finland|BOLD:AAC7811
Chionodes fumatella|MM17252|658[0n]bp|Finland|BOLD:AAC7811
Chionodes fumatella|MM18605|658[0n]bp|Finland.Finland Proper|BOLD:AAC7811
Chionodes fumatella|MM18600|658[0n]bp|Finland.Northern Ostrobothnia|BOLD:AAC7811
Chionodes fumatella|MM10326|658[0n]bp|Finland|BOLD:AAC7811
Chionodes fumatella|MM21189|658[0n]bp|Finland|BOLD:AAC7811
Chionodes fumatella|MM03501|606[0n]bp|Finland.Uusimaa|BOLD:AAC7811
Chionodes fumatella|MM13546|658[0n]bp|Finland|BOLD:AAC7811
Chionodes fumatella|MM03589|658[0n]bp|Finland|BOLD:AAC7811
Chionodes fumatella|MM17578|658[0n]bp|Finland|BOLD:AAC7811
Chionodes fumatella|MM13545|658[0n]bp|Finland|BOLD:AAC7811
Chionodes fumatella|MM18606|658[0n]bp|Finland.Finland Proper|BOLD:AAC7811
Chionodes fumatella|MM18607|658[0n]bp|Finland.Northern Ostrobothnia|BOLD:AAC7811
Chionodes fumatella|MM21190|658[0n]bp|Finland|BOLD:AAC7811
Chionodes luctuella|TLMF Lep 01908|631[0n]bp|Austria|BOLD:ABY8068
Chionodes luctuella|TLMF Lep 01907|658[0n]bp|Austria|BOLD:ABY8068
Chionodes luctuella|MM10155|658[0n]bp|Finland|BOLD:AAD2579
Chionodes luctuella|MM09778|658[0n]bp|Finland.Aland Islands|BOLD:AAD2579
Chionodes luctuella|MM00693|658[0n]bp|Finland.Lapland|BOLD:AAD2579
Gelechia muscosella|MM09807|658[0n]bp|Finland.Aland Islands|BOLD:AAC8632
Gelechia muscosella|MM06662|621[0n]bp|Finland|BOLD:AAC8632
Gelechia muscosella|MM02346|653[0n]bp|Finland.South Karelia|BOLD:AAC8632
Gelechia muscosella|MM06701|658[0n]bp|Finland.Aland Islands|BOLD:AAC8632
Gelechia muscosella|MM06698|658[0n]bp|Finland.Aland Islands|BOLD:AAC8632
Gelechia muscosella|MM06540|658[0n]bp|Finland|BOLD:AAC8632
Gelechia muscosella|TLMF Lep 08263|658[0n]bp|Austria.Vorarlberg|BOLD:AAC8632
Gelechia muscosella|TLMF Lep 07530|658[0n]bp|Austria.Vorarlberg|BOLD:AAC8632
Gelechia muscosella|MM13875|658[0n]bp|Finland.Lapland|BOLD:AAC8632
Gelechia muscosella|MM03478|658[0n]bp|Finland.Kymenlaakso|BOLD:AAC8632
Gelechia sestertiella|TLMF Lep 07708|658[0n]bp|Austria|BOLD:AAI7031
Gelechia sestertiella|MM18629|658[0n]bp|Finland|BOLD:AAI7031
Gelechia sestertiella|MM21059|658[0n]bp|Finland|BOLD:AAI7031
Gelechia sestertiella|MM14421|658[0n]bp|Finland|BOLD:AAI7031
Gelechia sabinellus|MM13511|658[0n]bp|Finland|BOLD:AAD7093
Gelechia sabinellus|TLMF Lep 08469|658[0n]bp|Austria.Vorarlberg|BOLD:AAD7093
Gelechia sabinellus|MM17576|658[0n]bp|Finland|BOLD:AAD7093

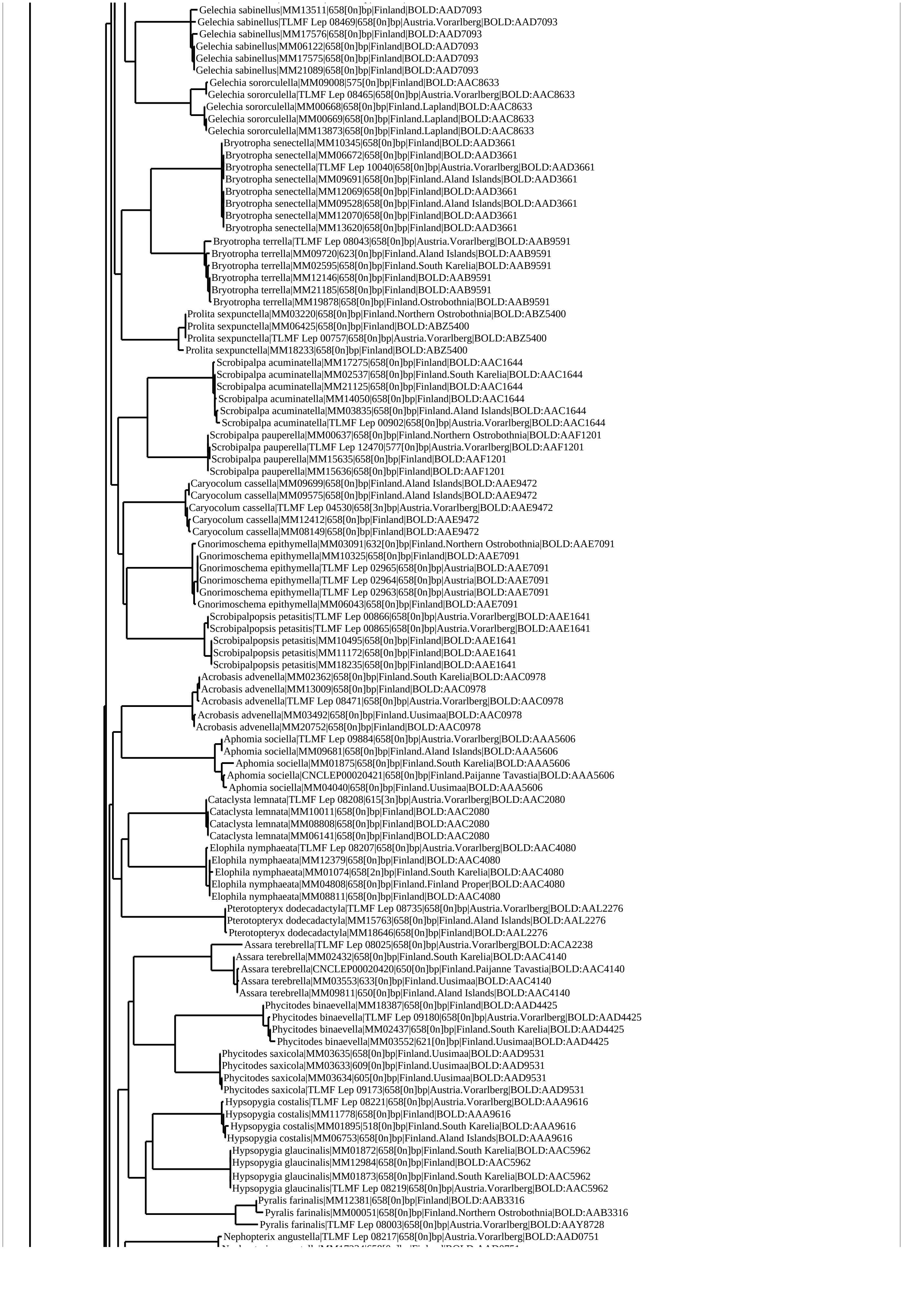
Gelechia sabinellus|MM13511|658[0n]bp|Finland|BOLD:AAD7093
Gelechia sabinellus|TLMF Lep 08469|658[0n]bp|Austria.Vorarlberg|BOLD:AAD7093
Gelechia sabinellus|MM17576|658[0n]bp|Finland|BOLD:AAD7093
Gelechia sabinellus|MM06122|658[0n]bp|Finland|BOLD:AAD7093
Gelechia sabinellus|MM17575|658[0n]bp|Finland|BOLD:AAD7093
Gelechia sabinellus|MM21089|658[0n]bp|Finland|BOLD:AAD7093
Gelechia sororculella|MM09008|575[0n]bp|Finland|BOLD:AAC8633
Gelechia sororculella|TLMF Lep 08465|658[0n]bp|Austria.Vorarlberg|BOLD:AAC8633
Gelechia sororculella|MM00668|658[0n]bp|Finland.Lapland|BOLD:AAC8633
Gelechia sororculella|MM00669|658[0n]bp|Finland.Lapland|BOLD:AAC8633
Gelechia sororculella|MM13873|658[0n]bp|Finland.Lapland|BOLD:AAC8633
Bryotropha senectella|MM10345|658[0n]bp|Finland|BOLD:AAD3661
Bryotropha senectella|MM06672|658[0n]bp|Finland|BOLD:AAD3661
Bryotropha senectella|TLMF Lep 10040|658[0n]bp|Austria.Vorarlberg|BOLD:AAD3661
Bryotropha senectella|MM09691|658[0n]bp|Finland.Aland Islands|BOLD:AAD3661
Bryotropha senectella|MM12069|658[0n]bp|Finland|BOLD:AAD3661
Bryotropha senectella|MM09528|658[0n]bp|Finland.Aland Islands|BOLD:AAD3661
Bryotropha senectella|MM12070|658[0n]bp|Finland|BOLD:AAD3661
Bryotropha senectella|MM13620|658[0n]bp|Finland|BOLD:AAD3661
Bryotropha terrella|TLMF Lep 08043|658[0n]bp|Austria.Vorarlberg|BOLD:AAB9591
Bryotropha terrella|MM09720|623[0n]bp|Finland.Aland Islands|BOLD:AAB9591
Bryotropha terrella|MM02595|658[0n]bp|Finland.South Karelia|BOLD:AAB9591
Bryotropha terrella|MM12146|658[0n]bp|Finland|BOLD:AAB9591
Bryotropha terrella|MM21185|658[0n]bp|Finland|BOLD:AAB9591
Bryotropha terrella|MM19878|658[0n]bp|Finland.Ostrobothnia|BOLD:AAB9591
Prolita sexpunctella|MM03220|658[0n]bp|Finland.Northern Ostrobothnia|BOLD:ABZ5400
Prolita sexpunctella|MM06425|658[0n]bp|Finland|BOLD:ABZ5400
Prolita sexpunctella|TLMF Lep 00757|658[0n]bp|Austria.Vorarlberg|BOLD:ABZ5400
Prolita sexpunctella|MM18233|658[0n]bp|Finland|BOLD:ABZ5400
Scrobipalpa acuminatella|MM17275|658[0n]bp|Finland|BOLD:AAC1644
Scrobipalpa acuminatella|MM02537|658[0n]bp|Finland.South Karelia|BOLD:AAC1644
Scrobipalpa acuminatella|MM21125|658[0n]bp|Finland|BOLD:AAC1644
Scrobipalpa acuminatella|MM14050|658[0n]bp|Finland|BOLD:AAC1644
Scrobipalpa acuminatella|MM03835|658[0n]bp|Finland.Aland Islands|BOLD:AAC1644
Scrobipalpa acuminatella|TLMF Lep 00902|658[0n]bp|Austria.Vorarlberg|BOLD:AAC1644
Scrobipalpa pauperella|MM00637|658[0n]bp|Finland.Northern Ostrobothnia|BOLD:AAF1201
Scrobipalpa pauperella|TLMF Lep 12470|577[0n]bp|Austria.Vorarlberg|BOLD:AAF1201
Scrobipalpa pauperella|MM15635|658[0n]bp|Finland|BOLD:AAF1201
Scrobipalpa pauperella|MM15636|658[0n]bp|Finland|BOLD:AAF1201
Caryocolum cassella|MM09699|658[0n]bp|Finland.Aland Islands|BOLD:AAE9472
Caryocolum cassella|MM09575|658[0n]bp|Finland.Aland Islands|BOLD:AAE9472
Caryocolum cassella|TLMF Lep 04530|658[3n]bp|Austria.Vorarlberg|BOLD:AAE9472
Caryocolum cassella|MM12412|658[0n]bp|Finland|BOLD:AAE9472
Caryocolum cassella|MM08149|658[0n]bp|Finland|BOLD:AAE9472
Gnorimoschema epithymella|MM03091|632[0n]bp|Finland.Northern Ostrobothnia|BOLD:AAE7091
Gnorimoschema epithymella|MM10325|658[0n]bp|Finland|BOLD:AAE7091
Gnorimoschema epithymella|TLMF Lep 02965|658[0n]bp|Austria|BOLD:AAE7091
Gnorimoschema epithymella|TLMF Lep 02964|658[0n]bp|Austria|BOLD:AAE7091
Gnorimoschema epithymella|TLMF Lep 02963|658[0n]bp|Austria|BOLD:AAE7091
Gnorimoschema epithymella|MM06043|658[0n]bp|Finland|BOLD:AAE7091
Scrobipalpopsis petasitis|TLMF Lep 00866|658[0n]bp|Austria.Vorarlberg|BOLD:AAE1641
Scrobipalpopsis petasitis|TLMF Lep 00865|658[0n]bp|Austria.Vorarlberg|BOLD:AAE1641
Scrobipalpopsis petasitis|MM10495|658[0n]bp|Finland|BOLD:AAE1641
Scrobipalpopsis petasitis|MM11172|658[0n]bp|Finland|BOLD:AAE1641
Scrobipalpopsis petasitis|MM18235|658[0n]bp|Finland|BOLD:AAE1641
Acrobasis advenella|MM02362|658[0n]bp|Finland.South Karelia|BOLD:AAC0978
Acrobasis advenella|MM13009|658[0n]bp|Finland|BOLD:AAC0978
Acrobasis advenella|TLMF Lep 08471|658[0n]bp|Austria.Vorarlberg|BOLD:AAC0978
Acrobasis advenella|MM03492|658[0n]bp|Finland.Uusimaa|BOLD:AAC0978
Acrobasis advenella|MM20752|658[0n]bp|Finland|BOLD:AAC0978
Aphomia sociella|TLMF Lep 09884|658[0n]bp|Austria.Vorarlberg|BOLD:AAA5606
Aphomia sociella|MM09681|658[0n]bp|Finland.Aland Islands|BOLD:AAA5606
Aphomia sociella|MM01875|658[0n]bp|Finland.South Karelia|BOLD:AAA5606
Aphomia sociella|CNCLEP00020421|658[0n]bp|Finland.Paijanne Tavastia|BOLD:AAA5606
Aphomia sociella|MM04040|658[0n]bp|Finland.Uusimaa|BOLD:AAA5606
Cataclysta lemnata|TLMF Lep 08208|615[3n]bp|Austria.Vorarlberg|BOLD:AAC2080
Cataclysta lemnata|MM10011|658[0n]bp|Finland|BOLD:AAC2080
Cataclysta lemnata|MM08808|658[0n]bp|Finland|BOLD:AAC2080
Cataclysta lemnata|MM06141|658[0n]bp|Finland|BOLD:AAC2080
Elophila nymphaeata|TLMF Lep 08207|658[0n]bp|Austria.Vorarlberg|BOLD:AAC4080
Elophila nymphaeata|MM12379|658[0n]bp|Finland|BOLD:AAC4080
Elophila nymphaeata|MM01074|658[2n]bp|Finland.South Karelia|BOLD:AAC4080
Elophila nymphaeata|MM04808|658[0n]bp|Finland.Finland Proper|BOLD:AAC4080
Elophila nymphaeata|MM08811|658[0n]bp|Finland|BOLD:AAC4080
Pterotopteryx dodecadactyla|TLMF Lep 08735|658[0n]bp|Austria.Vorarlberg|BOLD:AAL2276
Pterotopteryx dodecadactyla|MM15763|658[0n]bp|Finland.Aland Islands|BOLD:AAL2276
Pterotopteryx dodecadactyla|MM18646|658[0n]bp|Finland|BOLD:AAL2276
Assara terebrella|TLMF Lep 08025|658[0n]bp|Austria.Vorarlberg|BOLD:ACA2238
Assara terebrella|MM02432|658[0n]bp|Finland.South Karelia|BOLD:AAC4140
Assara terebrella|CNCLEP00020420|650[0n]bp|Finland.Paijanne Tavastia|BOLD:AAC4140
Assara terebrella|MM03553|633[0n]bp|Finland.Uusimaa|BOLD:AAC4140
Assara terebrella|MM09811|650[0n]bp|Finland.Aland Islands|BOLD:AAC4140
Phycitodes binaevella|MM18387|658[0n]bp|Finland|BOLD:AAD4425
Phycitodes binaevella|TLMF Lep 09180|658[0n]bp|Austria.Vorarlberg|BOLD:AAD4425
Phycitodes binaevella|MM02437|658[0n]bp|Finland.South Karelia|BOLD:AAD4425
Phycitodes binaevella|MM03552|621[0n]bp|Finland.Uusimaa|BOLD:AAD4425
Phycitodes saxicola|MM03635|658[0n]bp|Finland.Uusimaa|BOLD:AAD9531
Phycitodes saxicola|MM03633|609[0n]bp|Finland.Uusimaa|BOLD:AAD9531
Phycitodes saxicola|MM03634|605[0n]bp|Finland.Uusimaa|BOLD:AAD9531
Phycitodes saxicola|TLMF Lep 09173|658[0n]bp|Austria.Vorarlberg|BOLD:AAD9531
Hypsopygia costalis|TLMF Lep 08221|658[0n]bp|Austria.Vorarlberg|BOLD:AAA9616
Hypsopygia costalis|MM11778|658[0n]bp|Finland|BOLD:AAA9616
Hypsopygia costalis|MM01895|518[0n]bp|Finland.South Karelia|BOLD:AAA9616
Hypsopygia costalis|MM06753|658[0n]bp|Finland.Aland Islands|BOLD:AAA9616
Hypsopygia glaucinalis|MM01872|658[0n]bp|Finland.South Karelia|BOLD:AAC5962
Hypsopygia glaucinalis|MM12984|658[0n]bp|Finland|BOLD:AAC5962
Hypsopygia glaucinalis|MM01873|658[0n]bp|Finland.South Karelia|BOLD:AAC5962
Hypsopygia glaucinalis|TLMF Lep 08219|658[0n]bp|Austria.Vorarlberg|BOLD:AAC5962
Pyralis farinalis|MM12381|658[0n]bp|Finland|BOLD:AAB3316
Pyralis farinalis|MM00051|658[0n]bp|Finland.Northern Ostrobothnia|BOLD:AAB3316
Pyralis farinalis|TLMF Lep 08003|658[0n]bp|Austria.Vorarlberg|BOLD:AAY8728
Nephopterix angustella|TLMF Lep 08217|658[0n]bp|Austria.Vorarlberg|BOLD:AAD0751

Pyralis farinalis|MM00051|658[0n]bp|Finland.Northern Ostrobothnia|BOLD:AAB5316
Pyralis farinalis|TLMF Lep 08003|658[0n]bp|Austria.Vorarlberg|BOLD:AAY8728
Nephopterix angustella|TLMF Lep 08217|658[0n]bp|Austria.Vorarlberg|BOLD:AAD0751
Nephopterix angustella|MM17234|658[0n]bp|Finland|BOLD:AAD0751
Dioryctria abietella|MM17210|658[0n]bp|Finland|BOLD:AAB6957
Dioryctria abietella|MM02690|658[0n]bp|Finland.South Karelia|BOLD:AAB6957
Dioryctria abietella|TLMF Lep 08004|658[0n]bp|Austria.Vorarlberg|BOLD:AAB6957
Dioryctria abietella|MM17209|658[0n]bp|Finland|BOLD:AAB6957
Dioryctria simplicella|MM03545|563[0n]bp|Finland.Uusimaa|BOLD:AAB6958
Dioryctria simplicella|MM13008|658[0n]bp|Finland|BOLD:AAB6958
Dioryctria simplicella|MM11900|658[0n]bp|Finland|BOLD:AAB6958
Dioryctria simplicella|MM03792|654[0n]bp|Finland.Northern Ostrobothnia|BOLD:AAB6958
Dioryctria simplicella|TLMF Lep 07878|658[0n]bp|Austria.Vorarlberg|BOLD:AAB6958
Dioryctria simplicella|TLMF Lep 08087|658[0n]bp|Austria.Vorarlberg|BOLD:AAB6958
Dioryctria sylvestrella|MM03538|658[1n]bp|Finland.Uusimaa|BOLD:AAD0277
Dioryctria sylvestrella|MM13006|658[0n]bp|Finland|BOLD:AAD0277
Dioryctria sylvestrella|MM12120|658[0n]bp|Finland|BOLD:AAD0277
Dioryctria sylvestrella|TLMF Lep 09990|658[0n]bp|Austria.Vorarlberg|BOLD:AAD0277
Dioryctria sylvestrella|TLMF Lep 12487|658[0n]bp|Austria.Vorarlberg|BOLD:AAD0277
Hypochalcia ahenella|MM16011|658[0n]bp|Finland|BOLD:AAD0238
Hypochalcia ahenella|MM14474|658[0n]bp|Finland|BOLD:AAD0238
Hypochalcia ahenella|MM01880|658[0n]bp|Finland.South Karelia|BOLD:AAD0238
Hypochalcia ahenella|TLMF Lep 08001|658[0n]bp|Austria.Vorarlberg|BOLD:AAD0238
Hypochalcia ahenella|MM14355|658[0n]bp|Finland.Aland Islands|BOLD:AAD0238
Phycita roborella|MM04952|658[0n]bp|Finland.Finland Proper|BOLD:AAB8710
Phycita roborella|TLMF Lep 08002|658[0n]bp|Austria.Vorarlberg|BOLD:AAB8710
Phycita roborella|MM05197|658[0n]bp|Finland.Finland Proper|BOLD:AAB8710
Phycita roborella|MM06768|658[0n]bp|Finland.Aland Islands|BOLD:AAB8710
Phycita roborella|MM09785|658[0n]bp|Finland.Aland Islands|BOLD:AAB8710
Oncocera semirubella|MM13011|658[0n]bp|Finland|BOLD:AAD7672
Oncocera semirubella|MM03576|658[0n]bp|Finland.Kymenlaakso|BOLD:AAD7672
Oncocera semirubella|MM01878|622[0n]bp|Finland.South Karelia|BOLD:AAD7672
Oncocera semirubella|TLMF Lep 09156|658[0n]bp|Austria.Vorarlberg|BOLD:AAD7672
Catastia marginea|MM10534|658[0n]bp|Finland|BOLD:AAE9528
Catastia marginea|MM10535|658[0n]bp|Finland|BOLD:AAE9528
Catastia marginea|TLMF Lep 08432|658[0n]bp|Austria.Vorarlberg|BOLD:AAE9528
Catastia marginea|MM10536|658[0n]bp|Finland|BOLD:AAE9528
Matilella fusca|MM09468|658[0n]bp|Finland.Aland Islands|BOLD:AAA4759
Matilella fusca|MM09959|658[0n]bp|Finland|BOLD:AAA4759
Matilella fusca|TLMF Lep 09923|658[0n]bp|Austria.Vorarlberg|BOLD:AAA4759
Matilella fusca|MM06652|658[0n]bp|Finland|BOLD:AAA4759
Sciota adelphella|MM17812|658[0n]bp|Finland|BOLD:AAE3809
Sciota adelphella|MM17811|658[0n]bp|Finland|BOLD:AAE3809
Sciota adelphella|TLMF Lep 09778|658[0n]bp|Austria.Vorarlberg|BOLD:AAE3809
Sciota adelphella|MM17810|624[0n]bp|Finland|BOLD:AAE3809
Sciota hostilis|MM06867|658[0n]bp|Finland.Aland Islands|BOLD:AAD3590
Sciota hostilis|TLMF Lep 07473|658[0n]bp|Austria.Vorarlberg|BOLD:AAD3590
Sciota hostilis|MM02443|658[0n]bp|Finland.South Karelia|BOLD:AAD3590
Sciota hostilis|TLMF Lep 09937|658[0n]bp|Austria.Vorarlberg|BOLD:AAD3590
Sciota hostilis|MM11797|658[0n]bp|Finland|BOLD:AAD3590
Ephestia elutella|MM06861|658[0n]bp|Finland.Aland Islands|BOLD:AAC6157
Ephestia elutella|MM10369|658[0n]bp|Finland.Aland Islands|BOLD:AAC6157
Ephestia elutella|TLMF Lep 09910|658[0n]bp|Austria.Vorarlberg|BOLD:AAC6157
Ephestia elutella|MM10368|658[0n]bp|Finland.Aland Islands|BOLD:AAC6157
Plodia interpunctella|TLMF Lep 09900|658[0n]bp|Austria.Vorarlberg|BOLD:AAB2462
Plodia interpunctella|MM17620|658[0n]bp|Finland|BOLD:AAB2462
Plodia interpunctella|MM15774|610[0n]bp|Finland|BOLD:AAB2462
Vitula biviella|MM17820|625[0n]bp|Finland|BOLD:AAH7769
Vitula biviella|MM21115|658[0n]bp|Finland|BOLD:AAH7769
Vitula biviella|TLMF Lep 08478|658[0n]bp|Austria.Vorarlberg|BOLD:AAH7769
Prays fraxinella|MM11965|658[0n]bp|Finland|BOLD:AAB3722
Prays fraxinella|MM00130|658[0n]bp|Finland.Uusimaa|BOLD:AAB3722
Prays fraxinella|MM11966|658[0n]bp|Finland|BOLD:AAB3722
Prays fraxinella|MM09803|658[0n]bp|Finland.Aland Islands|BOLD:AAB3722
Prays fraxinella|MM09804|658[0n]bp|Finland.Aland Islands|BOLD:AAB3722
Prays fraxinella|TLMF Lep 12486|658[0n]bp|Austria.Vorarlberg|BOLD:AAB3722
Prays fraxinella|MM06819|658[0n]bp|Finland.Aland Islands|BOLD:AAB3722
Prays ruficeps|MM00112|670[0n]bp|Finland.Uusimaa|BOLD:AAB3723
Prays ruficeps|MM09690|658[0n]bp|Finland.Aland Islands|BOLD:AAB3723
Prays ruficeps|MM11921|658[0n]bp|Finland|BOLD:AAB3723
Prays ruficeps|TLMF Lep 07935|658[0n]bp|Austria.Vorarlberg|BOLD:AAB3723
Prays ruficeps|MM17338|658[1n]bp|Finland|BOLD:AAB3723
Prays ruficeps|MM08536|658[0n]bp|Finland|BOLD:AAB3723
Prays ruficeps|MM11919|658[0n]bp|Finland|BOLD:AAB3723
Prays ruficeps|MM08535|658[0n]bp|Finland.Aland Islands|BOLD:AAB3723
Prays ruficeps|MM11918|658[0n]bp|Finland|BOLD:AAB3723
Prays ruficeps|TLMF Lep 07483|658[0n]bp|Austria.Vorarlberg|BOLD:AAB3723
Prays ruficeps|MM17231|658[0n]bp|Finland|BOLD:AAB3723
Cedestis gysseleniella|MM00675|658[0n]bp|Finland.Lapland|BOLD:AAE4109
Cedestis gysseleniella|MM13735|658[0n]bp|Finland|BOLD:AAE4109
Cedestis gysseleniella|TLMF Lep 08071|658[0n]bp|Austria.Vorarlberg|BOLD:AAE4109
Cedestis gysseleniella|MM02540|658[0n]bp|Finland.South Karelia|BOLD:AAE4109
Swammerdamia compunctella|MM03148|658[0n]bp|Finland.Northern Ostrobothnia|BOLD:AAH9903
Swammerdamia compunctella|MM02616|658[0n]bp|Finland.South Karelia|BOLD:AAH9903
Swammerdamia compunctella|TLMF Lep 09143|658[0n]bp|Austria.Vorarlberg|BOLD:AAH9903
Swammerdamia compunctella|MM00704|567[0n]bp|Finland.Lapland|BOLD:AAH9903
Parapoynx stratiotata|TLMF Lep 07533|658[4n]bp|Austria.Vorarlberg|BOLD:AAC1045
Parapoynx stratiotata|MM06872|639[0n]bp|Finland.Aland Islands|BOLD:AAC1045
Parapoynx stratiotata|MM03544|658[0n]bp|Finland.Uusimaa|BOLD:AAC1045
Parapoynx stratiotata|MM08813|658[0n]bp|Finland|BOLD:AAC1045
Yponomeuta evonymella|MM00306|670[0n]bp|Finland.Northern Ostrobothnia|BOLD:AAA7740
Yponomeuta evonymella|MM00307|658[0n]bp|Finland.Northern Ostrobothnia|BOLD:AAA7740
Yponomeuta evonymella|TLMF Lep 08089|658[0n]bp|Austria.Vorarlberg|BOLD:AAA7740
Yponomeuta evonymella|MM13368|658[0n]bp|Finland|BOLD:AAA7740
Yponomeuta evonymella|MM02179|658[0n]bp|Finland.South Karelia|BOLD:AAA7740
Yponomeuta evonymella|MM05233|636[0n]bp|Finland.Finland Proper|BOLD:AAA7740
Yponomeuta evonymella|MM08126|658[0n]bp|Finland|BOLD:AAA7740
Yponomeuta malinellus|MM09458|658[0n]bp|Finland.Aland Islands|BOLD:AAA7740
Yponomeuta malinellus|TLMF Lep 09888|658[0n]bp|Austria.Vorarlberg|BOLD:AAA7740
Yponomeuta malinellus|MM02419|658[0n]bp|Finland.South Karelia|BOLD:AAA7740
Yponomeuta malinellus|MM11851|658[0n]bp|Finland|BOLD:AAA7740
Yponomeuta malinellus|MM02418|629[0n]bp|Finland.South Karelia|BOLD:AAA7740
Yponomeuta malinellus|MM15532|658[0n]bp|Finland|BOLD:AAA7740

Yponomeuta malinellus|MM11851|658[0n]bp|Finland|BOLD:AAA7740
Yponomeuta malinellus|MM02418|629[0n]bp|Finland.South Karelia|BOLD:AAA7740
Yponomeuta malinellus|MM15532|658[0n]bp|Finland|BOLD:AAA7740
Yponomeuta malinellus|TLMF Lep 08473|658[0n]bp|Austria.Vorarlberg|BOLD:AAA7740
Yponomeuta sedella|MM11861|658[0n]bp|Finland|BOLD:AAE4875
Yponomeuta sedella|MM03114|658[0n]bp|Finland.Northern Ostrobothnia|BOLD:AAE4875
Yponomeuta sedella|MM14386|658[0n]bp|Finland.Aland Islands|BOLD:AAE4875
Yponomeuta sedella|TLMF Lep 12473|658[0n]bp|Austria.Vorarlberg|BOLD:AAE4875
Ocnerostoma friesei|MM16016|658[0n]bp|Finland|BOLD:AAF4475
Ocnerostoma friesei|MM00545|636[0n]bp|Finland.Northern Ostrobothnia|BOLD:AAF4475
Ocnerostoma friesei|TLMF Lep 08484|658[0n]bp|Austria.Vorarlberg|BOLD:AAF4475
Ocnerostoma friesei|MM15536|658[0n]bp|Finland|BOLD:AAF4475
Ocnerostoma friesei|MM15535|658[0n]bp|Finland|BOLD:AAF4475
Plutella xylostella|MM08054|658[0n]bp|Finland|BOLD:AAA1513
Plutella xylostella|MM23149|602[0n]bp|Finland|BOLD:AAA1513
Plutella xylostella|MM00110|670[0n]bp|Finland.Uusimaa|BOLD:AAA1513
Plutella xylostella|MM22993|650[0n]bp|Finland|BOLD:AAA1513
Plutella xylostella|MM22991|632[0n]bp|Finland|BOLD:AAA1513
Plutella xylostella|MM03119|658[0n]bp|Finland.Northern Ostrobothnia|BOLD:AAA1513
Plutella xylostella|TLMF Lep 08237|658[0n]bp|Austria.Vorarlberg|BOLD:AAA1513
Plutella xylostella|MM00043|609[1n]bp|Finland.Northern Ostrobothnia|BOLD:AAA1513
Plutella xylostella|MM23148|658[0n]bp|Finland|BOLD:AAA1513
Plutella xylostella|MM23147|653[0n]bp|Finland|BOLD:AAA1513
Plutella xylostella|MM22996|658[0n]bp|Finland|BOLD:AAA1513
Plutella xylostella|MM22994|633[0n]bp|Finland|BOLD:AAA1513
Plutella xylostella|MM22995|631[0n]bp|Finland|BOLD:AAA1513
Plutella xylostella|MM22992|616[0n]bp|Finland|BOLD:AAA1513
Plutella xylostella|TLMF Lep 07967|658[0n]bp|Austria.Vorarlberg|BOLD:AAA1513
Plutella xylostella|MM22997|613[0n]bp|Finland|BOLD:AAA1513
Plutella xylostella|MM02280|658[0n]bp|Finland.South Karelia|BOLD:AAA1513
Rhigognostis senilella|TLMF Lep 09168|658[0n]bp|Austria.Vorarlberg|BOLD:AAF7514
Rhigognostis senilella|TLMF Lep 07399|658[0n]bp|Austria.Vorarlberg|BOLD:AAF7514
Rhigognostis senilella|MM08542|658[0n]bp|Finland|BOLD:AAF7514
Rhigognostis senilella|MM08541|658[0n]bp|Finland|BOLD:AAF7514
Rhigognostis senilella|MM08348|658[0n]bp|Finland|BOLD:AAF7514
Yponomeuta plumbella|TLMF Lep 08030|658[0n]bp|Austria.Vorarlberg|BOLD:ACL2673
Yponomeuta plumbella|MM17242|658[0n]bp|Finland|BOLD:ACL2673
Hypercallia citrinalis|MM06787|658[0n]bp|Finland.Aland Islands|BOLD:AAE1403
Hypercallia citrinalis|MM08641|658[0n]bp|Finland.Aland Islands|BOLD:AAE1403
Hypercallia citrinalis|MM09742|658[0n]bp|Finland.Aland Islands|BOLD:AAE1403
Hypercallia citrinalis|MM00071|670[0n]bp|Finland.Aland Islands|BOLD:AAE1403
Hypercallia citrinalis|TLMF Lep 00917|658[0n]bp|Austria.Vorarlberg|BOLD:AAE1403
Argyresthia brockeella|MM02309|636[0n]bp|Finland.South Karelia|BOLD:AAD2486
Argyresthia brockeella|MM03133|658[0n]bp|Finland.Northern Ostrobothnia|BOLD:AAD2486
Argyresthia brockeella|MM08132|658[0n]bp|Finland|BOLD:AAD2486
Argyresthia brockeella|TLMF Lep 08752|658[0n]bp|Austria.Vorarlberg|BOLD:AAD2486
Argyresthia goedartella|MM02340|658[0n]bp|Finland.South Karelia|BOLD:AAA8888
Argyresthia goedartella|MM12019|658[0n]bp|Finland|BOLD:AAA8888
Argyresthia goedartella|TLMF Lep 08224|658[0n]bp|Austria.Vorarlberg|BOLD:AAA8888
Argyresthia goedartella|MM03136|658[0n]bp|Finland.Northern Ostrobothnia|BOLD:AAA8888
Argyresthia retinella|TLMF Lep 08073|658[1n]bp|Austria.Vorarlberg|BOLD:AAF5148
Argyresthia retinella|MM03138|658[1n]bp|Finland.Northern Ostrobothnia|BOLD:AAF5148
Argyresthia retinella|MM13594|658[0n]bp|Finland|BOLD:AAF5148
Argyresthia retinella|BIOUG04118-C12|513[1n]bp|Finland.Northern Ostrobothnia|
Argyresthia retinella|BIOUG04118-A09|593[0n]bp|Finland.Northern Ostrobothnia|BOLD:AAF5148
Argyresthia retinella|MM02583|624[0n]bp|Finland.South Karelia|BOLD:AAF5148
Argyresthia retinella|BIOUG04118-D06|624[0n]bp|Finland.Northern Ostrobothnia|BOLD:AAF5148
Argyresthia retinella|BIOUG04118-D07|619[0n]bp|Finland.Northern Ostrobothnia|BOLD:AAF5148
Argyresthia retinella|BIOUG04118-A10|613[0n]bp|Finland.Northern Ostrobothnia|BOLD:AAF5148
Argyresthia retinella|BIOUG04118-D11|593[0n]bp|Finland.Northern Ostrobothnia|BOLD:AAF5148
Argyresthia retinella|BIOUG04118-D05|634[0n]bp|Finland.Northern Ostrobothnia|BOLD:AAF5148
Argyresthia retinella|BIOUG04118-D04|635[0n]bp|Finland.Northern Ostrobothnia|BOLD:AAF5148
Argyresthia retinella|BIOUG04118-A08|594[0n]bp|Finland.Northern Ostrobothnia|BOLD:AAF5148
Argyresthia albistria|MM13589|658[0n]bp|Finland|BOLD:AAD9653
Argyresthia albistria|MM06773|658[0n]bp|Finland.Aland Islands|BOLD:AAD9653
Argyresthia albistria|MM17949|658[0n]bp|Finland|BOLD:AAD9653
Argyresthia albistria|MM09511|658[0n]bp|Finland.Aland Islands|BOLD:AAD9653
Argyresthia albistria|TLMF Lep 08464|658[0n]bp|Austria.Vorarlberg|BOLD:AAD9653
Argyresthia conjugella|TLMF Lep 08746|658[0n]bp|Austria.Vorarlberg|BOLD:ACE7678
Argyresthia conjugella|TLMF Lep 08269|658[0n]bp|Austria.Vorarlberg|BOLD:ACE7678
Argyresthia conjugella|MM03137|658[0n]bp|Finland.Northern Ostrobothnia|BOLD:ACE7678
Argyresthia conjugella|MM06838|658[0n]bp|Finland.Aland Islands|BOLD:ACE7677
Argyresthia conjugella|MM08380|658[0n]bp|Finland|BOLD:ACE7677
Argyresthia conjugella|TLMF Lep 08070|658[0n]bp|Austria.Vorarlberg|BOLD:ACE7677
Argyresthia conjugella|TLMF Lep 03626|658[0n]bp|Austria.Vorarlberg|BOLD:ACE7677
Argyresthia conjugella|MM02292|658[0n]bp|Finland.South Karelia|BOLD:ACE7677
Argyresthia pruniella|MM18105|658[0n]bp|Finland|BOLD:AAC2750
Argyresthia pruniella|TLMF Lep 08052|658[0n]bp|Austria.Vorarlberg|BOLD:AAC2750
Argyresthia pruniella|MM21022|658[0n]bp|Finland|BOLD:AAC2750
Argyresthia pruniella|MM17950|658[0n]bp|Finland|BOLD:AAC2750
Argyresthia pruniella|TLMF Lep 08259|658[0n]bp|Austria.Vorarlberg|BOLD:AAC2750
Argyresthia pruniella|MM09531|658[0n]bp|Finland.Aland Islands|BOLD:AAC2750
Argyresthia sorbiella|MM14256|658[0n]bp|Finland.Aland Islands|BOLD:AAD9497
Argyresthia sorbiella|TLMF Lep 08413|658[0n]bp|Austria.Vorarlberg|BOLD:AAD9497
Argyresthia sorbiella|MM06767|658[0n]bp|Finland.Aland Islands|BOLD:AAD9497
Argyresthia sorbiella|MM13593|658[0n]bp|Finland|BOLD:AAD9497
Argyresthia sorbiella|MM06781|658[0n]bp|Finland.Aland Islands|BOLD:AAD9497
Argyresthia sorbiella|MM02629|658[1n]bp|Finland.South Karelia|BOLD:AAD9497
Argyresthia aurulentella|TLMF Lep 09189|658[0n]bp|Austria.Vorarlberg|BOLD:AAF5138
Argyresthia aurulentella|MM02627|658[0n]bp|Finland.South Karelia|BOLD:AAF5138
Argyresthia aurulentella|MM14643|658[0n]bp|Finland|BOLD:AAF5138
Argyresthia glabratella|MM18111|658[0n]bp|Finland|BOLD:AAD4102
Argyresthia glabratella|TLMF Lep 07494|658[0n]bp|Austria.Vorarlberg|BOLD:AAD4102
Argyresthia glabratella|TLMF Lep 09212|614[0n]bp|Austria.Vorarlberg|BOLD:AAD4102
Argyresthia glabratella|MM03262|658[0n]bp|Finland.Northern Ostrobothnia|BOLD:AAD4102
Argyresthia glabratella|MM18109|658[0n]bp|Finland|BOLD:AAD4102
Argyresthia glabratella|MM14184|658[0n]bp|Finland|BOLD:AAD4102
Argyresthia svenssoni|MM18108|658[0n]bp|Finland|BOLD:AAD4103
Argyresthia svenssoni|MM06446|658[0n]bp|Finland|BOLD:AAD4103
Argyresthia svenssoni|MM06408|658[0n]bp|Finland|BOLD:AAD4103
Argyresthia svenssoni|MM06447|658[0n]bp|Finland|BOLD:AAD4103
Argyresthia svenssoni|MM18107|658[0n]bp|Finland|BOLD:AAD4103
Argyresthia svenssoni|MM18115|658[0n]bp|Finland|BOLD:AAD4103

[illegible]
Argyresthia svenssoni|MM06447|658[0n]bp|Finland|BOLD:AAD4103
Argyresthia svenssoni|MM18107|658[0n]bp|Finland|BOLD:AAD4103
Argyresthia svenssoni|MM18115|658[0n]bp|Finland|BOLD:AAD4103
Argyresthia svenssoni|MM06445|658[0n]bp|Finland|BOLD:AAD4103
Argyresthia svenssoni|MM18110|658[0n]bp|Finland|BOLD:AAD4103
Argyresthia svenssoni|TLMF Lep 09985|658[0n]bp|Austria.Vorarlberg|BOLD:ACI7598
Ypsolopha falcella|TLMF Lep 08241|658[0n]bp|Austria.Vorarlberg|BOLD:ACF4757
Ypsolopha falcella|MM17333|658[0n]bp|Finland|BOLD:ACF4757
Ypsolopha falcella|MM06417|658[0n]bp|Finland|BOLD:ACF4757
Ypsolopha falcella|MM05027|658[0n]bp|Finland.Finland Proper|BOLD:ACF4757
Ypsolopha nemorella|MM06416|658[0n]bp|Finland|BOLD:AAE4873
Ypsolopha nemorella|MM02185|658[0n]bp|Finland.South Karelia|BOLD:AAE4873
Ypsolopha nemorella|MM06418|658[0n]bp|Finland|BOLD:AAE4873
Ypsolopha nemorella|TLMF Lep 08405|658[0n]bp|Austria.Vorarlberg|BOLD:AAE4873
Ypsolopha ustella|MM09709|658[0n]bp|Finland.Aland Islands|BOLD:AAD0001
Ypsolopha ustella|MM05314|611[0n]bp|Finland.Finland Proper|BOLD:AAD0001
Ypsolopha ustella|MM13377|658[0n]bp|Finland|BOLD:AAD0001
Ypsolopha ustella|TLMF Lep 08034|658[0n]bp|Austria.Vorarlberg|BOLD:AAD0001
Ypsolopha ustella|TLMF Lep 07960|658[0n]bp|Austria.Vorarlberg|BOLD:AAD0001
Clostera anachoreta|MM14099|658[0n]bp|Finland|BOLD:AAE2573
Clostera anachoreta|TLMF Lep 09506|658[0n]bp|Austria.Vorarlberg|BOLD:AAE2573
Clostera anachoreta|MM07684|658[0n]bp|Finland|BOLD:AAE2573
Clostera anachoreta|MM07683|658[0n]bp|Finland|BOLD:AAE2573
Clostera curtula|MM01003|658[0n]bp|Finland.South Karelia|BOLD:AAC5006
Clostera curtula|MM01004|658[0n]bp|Finland.South Karelia|BOLD:AAC5006
Clostera curtula|TLMF Lep 07831|658[0n]bp|Austria.Vorarlberg|BOLD:AAC5006
Clostera curtula|MM04573|658[0n]bp|Finland.Finland Proper|BOLD:AAC5006
Clostera pigra|TLMF Lep 06126|658[0n]bp|Austria.Vorarlberg|BOLD:AAD5412
Clostera pigra|MM00502|658[0n]bp|Finland.Northern Ostrobothnia|BOLD:AAD5412
Clostera pigra|MM01008|658[0n]bp|Finland.South Karelia|BOLD:AAD5412
Clostera pigra|MM12680|658[0n]bp|Finland|BOLD:AAD5412
Clostera pigra|MM01005|670[0n]bp|Finland.South Karelia|BOLD:AAD5412
Gluphisia crenata|MM01014|658[0n]bp|Finland.South Karelia|BOLD:AAE0994
Gluphisia crenata|TLMF Lep 05618|658[0n]bp|Austria.Vorarlberg|BOLD:AAE0994
Gluphisia crenata|MM01013|658[0n]bp|Finland.South Karelia|BOLD:AAE0994
Gluphisia crenata|MM09769|658[0n]bp|Finland.Aland Islands|BOLD:AAE0994
Cosmopterix lienigiella|TLMF Lep 08439|658[0n]bp|Austria.Vorarlberg|BOLD:AAJ5475
Cosmopterix lienigiella|MM15605|658[0n]bp|Finland|BOLD:AAJ5475
Cosmopterix lienigiella|MM18628|658[0n]bp|Finland|BOLD:AAJ5475
Cosmopterix orichalcea|MM06176|658[0n]bp|Finland|BOLD:AAJ5454
Cosmopterix orichalcea|MM15603|658[0n]bp|Finland|BOLD:AAJ5454
Cosmopterix orichalcea|MM21066|658[0n]bp|Finland|BOLD:AAJ5454
Cosmopterix orichalcea|TLMF Lep 08440|658[0n]bp|Austria.Vorarlberg|BOLD:AAJ5454
Cosmopterix orichalcea|MM18207|658[0n]bp|Finland|BOLD:AAJ5454
Bena bicolorana|MM18519|658[0n]bp|Finland|BOLD:AAB8808
Bena bicolorana|MM00728|658[0n]bp|Finland|BOLD:AAB8808
Bena bicolorana|TLMF Lep 08105|658[0n]bp|Austria.Vorarlberg|BOLD:AAB8808
Bena bicolorana|MM09767|658[0n]bp|Finland.Aland Islands|BOLD:AAB8808
Pseudoips prasinana|MM01591|658[0n]bp|Finland.South Karelia|BOLD:AAB8807
Pseudoips prasinana|MM00107|670[0n]bp|Finland.Uusimaa|BOLD:AAB8807
Pseudoips prasinana|MM04729|658[0n]bp|Finland.Finland Proper|BOLD:AAB8807
Pseudoips prasinana|MM01590|658[0n]bp|Finland.South Karelia|BOLD:AAB8807
Pseudoips prasinana|TLMF Lep 07858|658[0n]bp|Austria.Vorarlberg|BOLD:AAB8807
Hypatopa binotella|MM00133|670[0n]bp|Finland.Uusimaa|BOLD:AAB7100
Hypatopa binotella|MM15611|658[0n]bp|Finland.Aland Islands|BOLD:AAB7100
Hypatopa binotella|MM08251|658[0n]bp|Finland|BOLD:AAB7100
Hypatopa binotella|MM03505|598[0n]bp|Finland.Uusimaa|BOLD:AAB7100
Hypatopa binotella|MM13538|658[0n]bp|Finland|BOLD:AAB7100
Hypatopa binotella|TLMF Lep 08736|658[0n]bp|Austria.Vorarlberg|BOLD:AAB7100
Hypatopa inunctella|TLMF Lep 08450|658[0n]bp|Austria.Vorarlberg|BOLD:AAE5929
Hypatopa inunctella|MM06537|658[0n]bp|Finland|BOLD:AAE5929
Hypatopa inunctella|MM06538|658[0n]bp|Finland|BOLD:AAE5929
Lyonetia clerkella|MM13739|658[0n]bp|Finland|BOLD:AAD5255
Lyonetia clerkella|TLMF Lep 12612|658[0n]bp|Austria.Vorarlberg|BOLD:AAD5255
Lyonetia clerkella|MM00946|658[0n]bp|Finland.Aland Islands|BOLD:AAD5255
Lyonetia clerkella|MM02621|658[0n]bp|Finland.South Karelia|BOLD:AAD5256
Lyonetia clerkella|MM22063|658[0n]bp|Finland|BOLD:AAD5256
Aproaerema anthyllidella|TLMF Lep 08055|658[0n]bp|Austria.Vorarlberg|BOLD:AAD2266
Aproaerema anthyllidella|MM06379|658[0n]bp|Finland|BOLD:AAD2266
Aproaerema anthyllidella|MM06380|658[0n]bp|Finland|BOLD:AAD2266
Aproaerema anthyllidella|MM04196|658[0n]bp|Finland.Lapland|BOLD:AAD2266
Aproaerema anthyllidella|MM17255|658[0n]bp|Finland|BOLD:AAD2266
Aproaerema anthyllidella|TLMF Lep 10051|658[0n]bp|Austria.Vorarlberg|BOLD:AAD2266
Aproaerema anthyllidella|MM09497|658[0n]bp|Finland.Aland Islands|BOLD:AAD2267
Aproaerema anthyllidella|MM12471|658[0n]bp|Finland|BOLD:AAD2267
Aproaerema anthyllidella|MM13630|658[0n]bp|Finland|BOLD:AAD2267
Aproaerema anthyllidella|MM13629|658[0n]bp|Finland|BOLD:AAD2267
Aproaerema anthyllidella|MM17205|576[0n]bp|Finland|BOLD:AAD2267
Aproaerema anthyllidella|MM09496|658[0n]bp|Finland.Aland Islands|BOLD:AAD2267
Aproaerema anthyllidella|MM21140|658[0n]bp|Finland|BOLD:AAD2267
Syncopacma sangiella|MM21182|658[0n]bp|Finland|BOLD:AAE8758
Syncopacma sangiella|MM17727|658[0n]bp|Finland|BOLD:AAE8758
Syncopacma sangiella|TLMF Lep 08400|658[0n]bp|Austria.Vorarlberg|BOLD:AAE8758
Syncopacma sangiella|MM21150|658[0n]bp|Finland|BOLD:AAE8758
Syncopacma sangiella|MM04807|658[0n]bp|Finland.Finland Proper|BOLD:AAE8758
Syncopacma cinctella|MM02313|658[0n]bp|Finland.South Karelia|BOLD:AAD7223
Syncopacma cinctella|TLMF Lep 09948|658[0n]bp|Austria.Vorarlberg|BOLD:AAD7223
Syncopacma cinctella|MM13528|658[0n]bp|Finland|BOLD:AAD7223
Syncopacma cinctella|MM02314|658[0n]bp|Finland.South Karelia|BOLD:AAD7223
Syncopacma cinctella|MM12005|658[0n]bp|Finland|BOLD:AAD7223
Syncopacma cinctella|TLMF Lep 08039|658[0n]bp|Austria.Vorarlberg|BOLD:AAD7223
Syncopacma cinctella|MM00750|658[0n]bp|Finland.Northern Ostrobothnia|BOLD:AAD7223
Syncopacma cinctella|MM09503|658[0n]bp|Finland.Aland Islands|BOLD:AAD7223
Syncopacma cinctella|MM21180|658[0n]bp|Finland|BOLD:AAD7223
Syncopacma cinctella|MM13874|658[0n]bp|Finland.Lapland|BOLD:AAD7223
Syncopacma cinctella|MM09716|658[0n]bp|Finland.Aland Islands|BOLD:AAD7223
Syncopacma cinctella|MM03081|658[0n]bp|Finland.Northern Ostrobothnia|BOLD:AAD7223
Syncopacma taeniolella|MM09504|658[0n]bp|Finland.Aland Islands|BOLD:AAE8756
Syncopacma taeniolella|MM09501|658[0n]bp|Finland.Aland Islands|BOLD:AAE8756
Syncopacma taeniolella|MM09507|658[0n]bp|Finland.Aland Islands|BOLD:AAE8756
Syncopacma taeniolella|TLMF Lep 08040|658[0n]bp|Austria.Vorarlberg|BOLD:AAE8756
Syncopacma taeniolella|MM09505|658[0n]bp|Finland.Aland Islands|BOLD:AAE8756
[illegible]

Syncopacma taeniolella|MM09507|658[0n]bp|Finland.Aland Islands|BOLD:AAE8756
Syncopacma taeniolella|TLMF Lep 08040|658[0n]bp|Austria.Vorarlberg|BOLD:AAE8756
Syncopacma taeniolella|MM09505|658[0n]bp|Finland.Aland Islands|BOLD:AAE8756
Syncopacma taeniolella|MM09506|658[0n]bp|Finland.Aland Islands|BOLD:AAE8756
Scythris noricella|TLMF Lep 08743|658[0n]bp|Austria.Vorarlberg|BOLD:AAA9046
Scythris noricella|MM06104|658[0n]bp|Finland|BOLD:AAA9046
Phragmataecia castaneae|MM00170|670[0n]bp|Finland.Finland Proper|BOLD:AAE6494
Phragmataecia castaneae|MM11006|658[0n]bp|Finland|BOLD:AAE6494
Phragmataecia castaneae|TLMF Lep 05624|658[0n]bp|Austria.Vorarlberg|BOLD:AAE6494
Parnassius mnemosyne|MM17420|658[0n]bp|Finland|BOLD:AAA3342
Parnassius mnemosyne|MM17419|658[0n]bp|Finland|BOLD:AAA3342
Parnassius mnemosyne|TLMF Lep 06150|658[0n]bp|Austria.Vorarlberg|BOLD:AAA3343
Zelleria hepariella|MM08657|658[0n]bp|Finland.Aland Islands|BOLD:AAF6476
Zelleria hepariella|TLMF Lep 08202|615[0n]bp|Austria.Vorarlberg|BOLD:AAF6476
Zelleria hepariella|MM08533|658[0n]bp|Finland.Aland Islands|BOLD:AAF6476
Zelleria hepariella|MM14636|658[0n]bp|Finland.Aland Islands|BOLD:AAF6476
Zelleria hepariella|TLMF Lep 07400|658[0n]bp|Austria.Vorarlberg|BOLD:AAF6476
Dichomeris limosellus|TLMF Lep 07999|658[0n]bp|Austria.Vorarlberg|BOLD:AAV6621
Dichomeris limosellus|TLMF Lep 12496|658[0n]bp|Austria.Vorarlberg|BOLD:AAV6621
Dichomeris limosellus|TLMF Lep 09157|658[0n]bp|Austria.Vorarlberg|BOLD:AAV6621
Dichomeris limosellus|MM17729|658[0n]bp|Finland|BOLD:AAV6621
Bisigna procerella|MM09254|658[0n]bp|Finland|BOLD:AAF0146
Bisigna procerella|MM05280|565[0n]bp|Finland.Finland Proper|BOLD:AAF0146
Bisigna procerella|TLMF Lep 08261|658[0n]bp|Austria.Vorarlberg|BOLD:AAF0146
Bisigna procerella|MM11982|658[0n]bp|Finland|BOLD:AAF0146
Bisigna procerella|MM13578|658[0n]bp|Finland|BOLD:AAF0146
Crassa tinctella|MM13534|658[0n]bp|Finland|BOLD:AAE3946
Crassa tinctella|MM03838|658[0n]bp|Finland.Aland Islands|BOLD:AAE3946
Crassa tinctella|MM04077|632[0n]bp|Finland.Aland Islands|BOLD:AAE3946
Crassa tinctella|TLMF Lep 08101|658[0n]bp|Austria.Vorarlberg|BOLD:AAE3946
Denisia similella|TLMF Lep 10042|658[0n]bp|Austria.Vorarlberg|BOLD:AAE3459
Denisia similella|MM02559|635[0n]bp|Finland.South Karelia|BOLD:AAE3459
Denisia similella|MM03830|657[0n]bp|Finland.Aland Islands|BOLD:AAE3459
Denisia similella|MM03061|618[0n]bp|Finland.Northern Ostrobothnia|BOLD:AAE3459
Denisia similella|MM13600|656[0n]bp|Finland|BOLD:AAE3459
Denisia stipella|TLMF Lep 09945|658[0n]bp|Austria.Vorarlberg|BOLD:AAD6108
Denisia stipella|MM03060|633[0n]bp|Finland.Northern Ostrobothnia|BOLD:AAD6108
Denisia stipella|MM14279|658[0n]bp|Finland.Aland Islands|BOLD:AAD6108
Denisia stipella|MM08273|658[0n]bp|Finland|BOLD:AAD6108
Denisia stipella|MM08046|658[0n]bp|Finland|BOLD:AAD6108
Denisia stipella|TLMF Lep 09390|658[0n]bp|Austria.Vorarlberg|BOLD:AAD6108
Denisia stipella|TLMF Lep 09979|658[0n]bp|Austria.Vorarlberg|BOLD:AAD6108
Harpella forficella|MM10381|658[0n]bp|Finland.Aland Islands|BOLD:AAE6991
Harpella forficella|TLMF Lep 08216|658[0n]bp|Austria.Vorarlberg|BOLD:AAE6991
Harpella forficella|MM09485|658[0n]bp|Finland.Aland Islands|BOLD:AAE6991
Harpella forficella|MM14390|658[0n]bp|Finland.Aland Islands|BOLD:AAE6991
Harpella forficella|MM06866|670[0n]bp|Finland.Aland Islands|BOLD:AAE6991
Oecophora bractella|MM03831|670[0n]bp|Finland.Aland Islands|BOLD:AAC1073
Oecophora bractella|TLMF Lep 09171|658[0n]bp|Austria.Vorarlberg|BOLD:AAC1073
Oecophora bractella|MM03842|658[0n]bp|Finland.Aland Islands|BOLD:AAC1073
Oecophora bractella|MM13190|658[0n]bp|Finland|BOLD:AAC1073
Oecophora bractella|MM14375|658[0n]bp|Finland.Aland Islands|BOLD:AAC1073
Adscita statices|MM00313|635[0n]bp|Finland.Northern Ostrobothnia|BOLD:AAD5110
Adscita statices|MM06560|658[0n]bp|Finland|BOLD:AAD5110
Adscita statices|MM06561|658[0n]bp|Finland|BOLD:AAD5110
Adscita statices|MM18630|658[0n]bp|Finland|BOLD:AAD5110
Adscita statices|MM00312|670[0n]bp|Finland.Northern Ostrobothnia|BOLD:AAD5110
Adscita statices|TLMF Lep 10013|658[0n]bp|Austria.Vorarlberg|BOLD:AAD5110
Adscita statices|MM17364|658[1n]bp|Finland|BOLD:AAD5110
Neofaculta ericetella|TLMF Lep 08078|658[0n]bp|Austria.Vorarlberg|BOLD:AAD1501
Neofaculta ericetella|MM03909|658[0n]bp|Finland.Northern Ostrobothnia|BOLD:AAD1501
Neofaculta ericetella|MM13971|658[0n]bp|Finland|BOLD:AAD1501
Neofaculta ericetella|MM13970|658[0n]bp|Finland|BOLD:AAD1501
Neofaculta ericetella|TLMF Lep 09949|658[0n]bp|Austria.Vorarlberg|BOLD:AAD1501
Neofaculta infernella|MM03437|658[0n]bp|Finland.Lapland|BOLD:AAC1362
Neofaculta infernella|MM17908|637[1n]bp|Finland|BOLD:AAC1362
Neofaculta infernella|MM05472|658[0n]bp|Finland.Northern Ostrobothnia|BOLD:AAC1362
Neofaculta infernella|MM09029|658[0n]bp|Finland|BOLD:AAC1362
Neofaculta infernella|MM18251|658[0n]bp|Finland|BOLD:AAC1362
Neofaculta infernella|MM18250|658[0n]bp|Finland|BOLD:AAC1362
Neofaculta infernella|MM02323|626[0n]bp|Finland.South Karelia|BOLD:AAC1363
Neofaculta infernella|MM13507|655[0n]bp|Finland|BOLD:AAC1363
Neofaculta infernella|MM13508|658[0n]bp|Finland|BOLD:AAC1363
Neofaculta infernella|MM21173|658[0n]bp|Finland|BOLD:AAC1363
Neofaculta infernella|MM21174|658[0n]bp|Finland|BOLD:AAC1363
Neofaculta infernella|MM04223|658[0n]bp|Finland.South Karelia|BOLD:AAC1363
Neofaculta infernella|MM17906|658[0n]bp|Finland|BOLD:AAC1363
Neofaculta infernella|MM17907|658[0n]bp|Finland|BOLD:AAC1363
Neofaculta infernella|MM18249|658[0n]bp|Finland|BOLD:AAC1363
Neofaculta infernella|TLMF Lep 00867|658[0n]bp|Austria.Vorarlberg|BOLD:AAC1363
Neofaculta infernella|TLMF Lep 00913|658[0n]bp|Austria.Vorarlberg|BOLD:AAC1363
Aporia crataegi|MM00095|658[0n]bp|Finland.Northern Ostrobothnia|BOLD:AAA8773
Aporia crataegi|MM17131|658[0n]bp|Finland|BOLD:AAA8773
Aporia crataegi|MM17130|658[0n]bp|Finland|BOLD:AAA8773
Aporia crataegi|TLMF Lep 09098|658[0n]bp|Austria.Vorarlberg|BOLD:AAA8773
Nymphula nitidulata|MM00767|621[0n]bp|Finland.Northern Ostrobothnia|BOLD:AAE2813
Nymphula nitidulata|MM13005|658[0n]bp|Finland|BOLD:AAF2874
Nymphula nitidulata|TLMF Lep 08414|658[0n]bp|Austria.Vorarlberg|BOLD:AAF2874
Nymphula nitidulata|MM01897|615[0n]bp|Finland.South Karelia|BOLD:AAF2874
Pieris brassicae|TLMF Lep 09808|658[0n]bp|Austria.Vorarlberg|BOLD:AAB0552
Pieris brassicae|MM03697|658[0n]bp|Finland.Uusimaa|BOLD:AAB0552
Pieris brassicae|MM03696|658[0n]bp|Finland.Uusimaa|BOLD:AAB0552
Pieris brassicae|MM17127|658[0n]bp|Finland|BOLD:AAB0552
Pieris napi|MM00509|658[0n]bp|Finland.Northern Ostrobothnia|BOLD:AAA2226
Pieris napi|MM00514|658[0n]bp|Finland.Northern Ostrobothnia|BOLD:AAA2226
Pieris napi|MM17125|658[0n]bp|Finland|BOLD:AAA2226
Pieris napi|TLMF Lep 09807|658[0n]bp|Austria.Vorarlberg|BOLD:AAA2226
Pieris rapae|MM06585|670[0n]bp|Finland.North Karelia|BOLD:AAA2224
Pieris rapae|TLMF Lep 10009|658[0n]bp|Austria.Vorarlberg|BOLD:AAA2224
Pieris rapae|MM17128|658[0n]bp|Finland|BOLD:AAA2224
Pieris rapae|MM17129|634[0n]bp|Finland|BOLD:AAA2224
Anchinia daphnella|MM09784|658[0n]bp|Finland.Aland Islands|BOLD:ACE5878
Anchinia daphnella|TLMF Lep 08392|658[0n]bp|Austria.Vorarlberg|BOLD:ACE5878

Pieris rapae|MM17129|634[0n]bp|Finland|BOLD:AAA2224
Anchinia daphnella|MM09784|658[0n]bp|Finland.Aland Islands|BOLD:ACE5878
Anchinia daphnella|TLMF Lep 08392|658[0n]bp|Austria.Vorarlberg|BOLD:ACE5878
Anchinia daphnella|MM08881|658[0n]bp|Finland|BOLD:ACE5878
Anchinia daphnella|MM01206|609[0n]bp|Finland.South Karelia|BOLD:AAE5098
Anchinia daphnella|MM01207|670[0n]bp|Finland.South Karelia|BOLD:AAE5098
Anchinia daphnella|MM08880|658[0n]bp|Finland|BOLD:AAE5098
Diurnea fagella|TLMF Lep 09776|658[0n]bp|Austria.Vorarlberg|BOLD:AAF5356
Diurnea fagella|MM05960|670[0n]bp|Finland.Aland Islands|BOLD:AAF5356
Diurnea lipsiella|MM03178|658[0n]bp|Finland.Aland Islands|BOLD:AAD9970
Diurnea lipsiella|MM04207|658[0n]bp|Finland.Aland Islands|BOLD:AAD9970
Diurnea lipsiella|TLMF Lep 08804|658[0n]bp|Austria.Vorarlberg|BOLD:AAD9970
Diurnea lipsiella|MM04511|658[0n]bp|Finland.Finland Proper|BOLD:AAD9970
Sorhagenia janiszewskae|MM02331|670[0n]bp|Finland.South Karelia|BOLD:AAF4360
Sorhagenia janiszewskae|TLMF Lep 09190|658[0n]bp|Austria.Vorarlberg|BOLD:AAF4360
Sorhagenia janiszewskae|MM02332|658[0n]bp|Finland.South Karelia|BOLD:AAF4360
Sorhagenia janiszewskae|MM02330|609[0n]bp|Finland.South Karelia|BOLD:AAF4360
Sorhagenia janiszewskae|MM02333|658[0n]bp|Finland.South Karelia|BOLD:AAF4360
Sorhagenia rhamniella|MM09736|658[0n]bp|Finland.Aland Islands|BOLD:AAD3133
Sorhagenia rhamniella|TLMF Lep 08064|658[0n]bp|Austria.Vorarlberg|BOLD:AAD3133
Sorhagenia rhamniella|TLMF Lep 08260|658[0n]bp|Austria.Vorarlberg|BOLD:AAD3133
Depressaria olerella|TLMF Lep 12538|658[0n]bp|Austria.Vorarlberg|BOLD:AAF8185
Depressaria olerella|MM18139|658[0n]bp|Finland|BOLD:AAF8185
Depressaria olerella|MM05300|641[0n]bp|Finland.Finland Proper|BOLD:AAF8185
Depressaria olerella|MM02216|658[0n]bp|Finland.South Karelia|BOLD:AAF8185
Depressaria olerella|MM13352|658[0n]bp|Finland|BOLD:AAF8185
Depressaria pulcherrimella|TLMF Lep 07994|658[0n]bp|Austria.Vorarlberg|BOLD:AAF8186
Depressaria pulcherrimella|MM08544|658[0n]bp|Finland|BOLD:AAF8186
Depressaria pulcherrimella|MM08543|658[0n]bp|Finland|BOLD:AAF8186
Depressaria pulcherrimella|MM14699|658[0n]bp|Finland|BOLD:AAF8186
Depressaria chaerophylli|MM08546|658[0n]bp|Finland|BOLD:AAF8167
Depressaria chaerophylli|TLMF Lep 07471|658[1n]bp|Austria.Vorarlberg|BOLD:AAF8167
Depressaria chaerophylli|MM18137|658[0n]bp|Finland|BOLD:AAF8167
Depressaria chaerophylli|MM08545|658[0n]bp|Finland|BOLD:AAF8167
Depressaria pimpinellae|TLMF Lep 12534|658[0n]bp|Austria.Vorarlberg|BOLD:AAD6055
Depressaria pimpinellae|MM00438|658[0n]bp|Finland|BOLD:AAD6055
Depressaria pimpinellae|MM03217|658[0n]bp|Finland.Northern Ostrobothnia|BOLD:AAD6055
Depressaria pimpinellae|MM02218|637[0n]bp|Finland.South Karelia|BOLD:AAD6055
Depressaria radiella|TLMF Lep 12510|658[0n]bp|Austria.Vorarlberg|BOLD:AAB6253
Depressaria radiella|MM05956|658[0n]bp|Finland.Aland Islands|BOLD:AAB6253
Depressaria radiella|MM13202|656[0n]bp|Finland|BOLD:AAB6253
Depressaria radiella|MM13203|658[0n]bp|Finland|BOLD:AAB6253
Agonopterix heracliana|MM01086|658[0n]bp|Finland.South Karelia|BOLD:ACF1608
Agonopterix heracliana|MM00364|670[0n]bp|Finland|BOLD:ACF1608
Agonopterix heracliana|MM01087|658[0n]bp|Finland.South Karelia|BOLD:ACF1608
Agonopterix heracliana|TLMF Lep 07957|658[0n]bp|Austria.Vorarlberg|BOLD:AAC7415
Agonopterix heracliana|MM00418|639[0n]bp|Finland.Northern Ostrobothnia|BOLD:ACF1608
Agonopterix heracliana|MM00911|658[0n]bp|Finland.Aland Islands|BOLD:ACF1608
Agonopterix angelicella|MM23127|658[0n]bp|Finland|BOLD:ACE0304
Agonopterix angelicella|MM23125|658[0n]bp|Finland|BOLD:ABZ4758
Agonopterix angelicella|MM08211|658[0n]bp|Finland|BOLD:AAE3381
Agonopterix angelicella|MM23132|658[0n]bp|Finland|BOLD:AAE3381
Agonopterix angelicella|MM23131|658[0n]bp|Finland|BOLD:AAE3381
Agonopterix angelicella|MM22949|658[0n]bp|Finland|BOLD:AAE3381
Agonopterix angelicella|MM23129|658[0n]bp|Finland|BOLD:AAE3381
Agonopterix angelicella|MM23130|658[0n]bp|Finland|BOLD:AAE3381
Agonopterix angelicella|TLMF Lep 04507|658[0n]bp|Austria.Vorarlberg|BOLD:AAE3381
Agonopterix angelicella|MM02196|658[0n]bp|Finland.South Karelia|BOLD:AAE3381
Agonopterix angelicella|MM23128|658[0n]bp|Finland|BOLD:AAE3381
Agonopterix angelicella|MM22950|614[0n]bp|Finland|BOLD:AAE3381
Agonopterix angelicella|MM03053|581[0n]bp|Finland.Northern Ostrobothnia|BOLD:AAE3381
Agonopterix angelicella|MM23124|658[0n]bp|Finland|BOLD:AAE3381
Agonopterix angelicella|MM23133|658[0n]bp|Finland|BOLD:AAE3381
Agonopterix astrantiae|MM18146|658[0n]bp|Finland|BOLD:AAJ1481
Agonopterix astrantiae|MM03748|654[0n]bp|Finland.Aland Islands|BOLD:AAJ1481
Agonopterix astrantiae|TLMF Lep 05638|658[0n]bp|Austria.Vorarlberg|BOLD:AAJ1481
Agonopterix astrantiae|MM03749|658[0n]bp|Finland.Aland Islands|BOLD:AAJ1481
Agonopterix arenella|MM04061|638[0n]bp|Finland.Northern Ostrobothnia|BOLD:AAC6982
Agonopterix arenella|TLMF Lep 07997|658[0n]bp|Austria.Vorarlberg|BOLD:AAC6982
Agonopterix arenella|CNCLEP00020426|658[0n]bp|Finland.Paijanne Tavastia|BOLD:AAC6982
Agonopterix arenella|MM13348|658[0n]bp|Finland|BOLD:AAC6982
Agonopterix arenella|MM02191|634[0n]bp|Finland.South Karelia|BOLD:AAC6982
Agonopterix kaekeritziana|MM18997|658[0n]bp|Finland|BOLD:AAF7198
Agonopterix kaekeritziana|MM14764|658[0n]bp|Finland|BOLD:AAF7198
Agonopterix kaekeritziana|MM02681|658[0n]bp|Finland.South Karelia|BOLD:AAF7198
Agonopterix kaekeritziana|MM03648|658[0n]bp|Finland.Uusimaa|BOLD:AAF7198
Agonopterix kaekeritziana|TLMF Lep 07993|634[0n]bp|Austria.Vorarlberg|BOLD:AAF7198
Agonopterix ocellana|MM00504|658[0n]bp|Finland.Lapland|BOLD:AAF7176
Agonopterix ocellana|TLMF Lep 07987|658[0n]bp|Austria.Vorarlberg|BOLD:AAF7176
Agonopterix ocellana|MM00461|658[0n]bp|Finland.South Karelia|BOLD:AAF7176
Agonopterix ocellana|MM18145|658[0n]bp|Finland|BOLD:AAF7176
Agonopterix conterminella|MM02198|625[0n]bp|Finland.South Karelia|BOLD:AAE7213
Agonopterix conterminella|MM18889|658[0n]bp|Finland|BOLD:AAE7213
Agonopterix conterminella|MM18890|658[0n]bp|Finland|BOLD:AAE7213
Agonopterix conterminella|MM00768|658[0n]bp|Finland.Northern Ostrobothnia|BOLD:AAE7213
Agonopterix conterminella|MM18888|658[0n]bp|Finland|BOLD:AAE7213
Agonopterix conterminella|MM18891|658[0n]bp|Finland|BOLD:AAE7213
Agonopterix conterminella|MM17544|658[0n]bp|Finland|BOLD:AAE7213
Agonopterix conterminella|MM17547|658[0n]bp|Finland|BOLD:AAE7213
Agonopterix conterminella|MM10277|658[0n]bp|Finland|BOLD:AAE7213
Agonopterix conterminella|MM15555|658[0n]bp|Finland|BOLD:AAE7213
Agonopterix conterminella|TLMF Lep 08446|658[0n]bp|Austria.Vorarlberg|BOLD:AAE7213
Agonopterix liturosa|MM02201|576[0n]bp|Finland.South Karelia|BOLD:AAE7191
Agonopterix liturosa|TLMF Lep 05639|658[0n]bp|Austria.Vorarlberg|BOLD:AAE7191
Agonopterix liturosa|MM06815|658[0n]bp|Finland.Aland Islands|BOLD:AAE7191
Agonopterix liturosa|TLMF Lep 07995|658[0n]bp|Austria.Vorarlberg|BOLD:AAE7191
Agonopterix liturosa|MM13226|658[0n]bp|Finland|BOLD:AAE7191
Semioscopis avellanella|TLMF Lep 07959|658[0n]bp|Austria.Vorarlberg|BOLD:AAH9809
Semioscopis avellanella|MM01135|658[0n]bp|Finland.South Karelia|BOLD:AAH9809
Semioscopis avellanella|MM01136|658[0n]bp|Finland.South Karelia|BOLD:AAH9809
Semioscopis avellanella|MM18129|658[0n]bp|Finland|BOLD:AAH9809
Thiotricha subocellea|TLMF Lep 00901|658[0n]bp|Austria.Vorarlberg|BOLD:AAD7105
Thiotricha subocellea|MM05683|658[0n]bp|Finland.Aland Islands|[illegible]

Semioscopis avellanella|MM01130|658[0n]bp|Finland.South Karelia|BOLD:AAH9809
Semioscopis avellanella|MM18129|658[0n]bp|Finland|BOLD:AAH9809
Thiotricha subocellea|TLMF Lep 00901|658[0n]bp|Austria.Vorarlberg|BOLD:AAD7105
Thiotricha subocellea|MM05683|658[0n]bp|Finland.Aland Islands|BOLD:AAD7105
Thiotricha subocellea|MM09333|670[0n]bp|Finland.Uusimaa|BOLD:AAD7105
Thiotricha subocellea|MM09334|658[0n]bp|Finland|BOLD:AAD7105
Thiotricha subocellea|MM17233|658[0n]bp|Finland|BOLD:AAD7105
Ochropacha duplaris|MM01230|658[0n]bp|Finland.South Karelia|BOLD:AAC6798
Ochropacha duplaris|TLMF Lep 04643|658[0n]bp|Austria.Vorarlberg|BOLD:AAC6798
Ochropacha duplaris|MM08165|658[0n]bp|Finland|BOLD:AAC6798
Ochropacha duplaris|MM01231|658[0n]bp|Finland.South Karelia|BOLD:AAC6798
Achlya flavicornis|MM10869|658[0n]bp|Finland|BOLD:ABX5484
Achlya flavicornis|MM01519|670[0n]bp|Finland.South Karelia|BOLD:ABX5484
Achlya flavicornis|TLMF Lep 09499|658[0n]bp|Austria.Vorarlberg|BOLD:AAD6559
Achlya flavicornis|TLMF Lep 12459|658[0n]bp|Austria.Vorarlberg|BOLD:AAD6559
Achlya flavicornis|MM00413|648[0n]bp|Finland.Northern Ostrobothnia|BOLD:AAD6559
Achlya flavicornis|MM01520|658[0n]bp|Finland.South Karelia|BOLD:AAD6559
Tethea or|TLMF Lep 04654|658[0n]bp|Austria.Vorarlberg|BOLD:AAC9856
Tethea or|MM01169|658[0n]bp|Finland.South Karelia|BOLD:AAC9855
Tethea or|MM01168|658[0n]bp|Finland.South Karelia|BOLD:AAC9855
Tethea or|MM00647|658[0n]bp|Finland.Northern Ostrobothnia|BOLD:AAC9855
Habrosyne pyritoides|MM17427|658[0n]bp|Finland|BOLD:AAC5831
Habrosyne pyritoides|TLMF Lep 07902|658[0n]bp|Austria.Vorarlberg|BOLD:AAC5831
Habrosyne pyritoides|MM17426|658[0n]bp|Finland|BOLD:AAC5831
Habrosyne pyritoides|MM18671|658[0n]bp|Finland|BOLD:AAC5831
Tetheella fluctuosa|MM09790|658[0n]bp|Finland.Aland Islands|BOLD:AAD3647
Tetheella fluctuosa|MM01502|658[0n]bp|Finland.South Karelia|BOLD:AAD3647
Tetheella fluctuosa|MM01501|658[0n]bp|Finland.South Karelia|BOLD:AAD3647
Tetheella fluctuosa|TLMF Lep 12461|658[0n]bp|Austria.Vorarlberg|BOLD:AAD3647
Thyatira batis|MM01145|658[0n]bp|Finland.South Karelia|BOLD:ABY7313
Thyatira batis|MM00027|670[0n]bp|Finland.South Karelia|BOLD:AAC4671
Thyatira batis|MM03707|658[0n]bp|Finland.Aland Islands|BOLD:AAC4671
Thyatira batis|MM01144|658[0n]bp|Finland.South Karelia|BOLD:AAC4671
Thyatira batis|TLMF Lep 07890|658[0n]bp|Austria.Vorarlberg|BOLD:AAC4671
Pexicopia malvella|MM03491|614[0n]bp|Finland.Uusimaa|BOLD:AAD9025
Pexicopia malvella|MM06850|658[0n]bp|Finland.Aland Islands|BOLD:AAD9025
Pexicopia malvella|MM03479|670[0n]bp|Finland.Kymenlaakso|BOLD:AAD9025
Pexicopia malvella|TLMF Lep 07519|658[0n]bp|Austria.Vorarlberg|BOLD:AAD9025
Pexicopia malvella|TLMF Lep 08234|658[0n]bp|Austria.Vorarlberg|BOLD:AAD9025
Pexicopia malvella|TLMF Lep 09914|658[0n]bp|Austria.Vorarlberg|BOLD:AAD9025
Pexicopia malvella|MM12429|658[0n]bp|Finland|BOLD:AAD9025
Cyclophora annularia|TLMF Lep 07867|658[0n]bp|Austria.Vorarlberg|BOLD:AAD6103
Cyclophora annularia|MM05654|658[0n]bp|Finland.Uusimaa|BOLD:AAD6103
Cyclophora linearia|TLMF Lep 04631|658[0n]bp|Austria.Vorarlberg|BOLD:ABX5086
Cyclophora linearia|MM15918|658[0n]bp|Finland|BOLD:ABX5086
Timandra comae|MM01496|658[0n]bp|Finland.South Karelia|BOLD:AAB0828
Timandra comae|TLMF Lep 08545|658[0n]bp|Austria.Vorarlberg|BOLD:AAB0828
Timandra comae|MM01361|658[0n]bp|Finland.South Karelia|BOLD:AAB0828
Timandra comae|MM12899|658[0n]bp|Finland|BOLD:AAB0828
Timandra comae|MM04645|658[0n]bp|Finland.Finland Proper|BOLD:AAB0828
Timandra comae|MM12784|658[0n]bp|Finland|BOLD:AAB0828
Timandra comae|MM12785|658[0n]bp|Finland|BOLD:AAB0828
Timandra comae|MM04838|658[0n]bp|Finland.Finland Proper|BOLD:AAB0828
Timandra comae|MM04806|658[0n]bp|Finland.Finland Proper|BOLD:AAB0828
Pyrausta despicata|MM06837|658[0n]bp|Finland.Aland Islands|BOLD:AAB9618
Pyrausta despicata|MM03447|658[0n]bp|Finland.Uusimaa|BOLD:AAB9618
Pyrausta despicata|MM13003|658[0n]bp|Finland|BOLD:AAB9618
Pyrausta despicata|MM11762|658[0n]bp|Finland|BOLD:AAB9618
Pyrausta despicata|TLMF Lep 07893|658[0n]bp|Austria.Vorarlberg|BOLD:AAB9618
Paratalanta hyalinalis|MM17845|503[0n]bp|Finland|BOLD:AAF7132
Paratalanta hyalinalis|MM02377|658[0n]bp|Finland.South Karelia|BOLD:AAF7132
Paratalanta hyalinalis|TLMF Lep 08178|658[0n]bp|Austria.Vorarlberg|BOLD:AAF7132
Paratalanta hyalinalis|MM11134|633[0n]bp|Finland|BOLD:AAF7132
Paratalanta pandalis|TLMF Lep 07892|658[0n]bp|Austria.Vorarlberg|BOLD:AAE7251
Paratalanta pandalis|MM01853|658[0n]bp|Finland.South Karelia|BOLD:AAE7251
Paratalanta pandalis|MM04330|658[0n]bp|Finland.Finland Proper|BOLD:AAE7251
Paratalanta pandalis|MM01852|658[0n]bp|Finland.South Karelia|BOLD:AAE7251
Anania coronata|MM09813|658[0n]bp|Finland.Aland Islands|BOLD:ACF0483
Anania coronata|MM01868|658[0n]bp|Finland.South Karelia|BOLD:ACF0483
Anania coronata|MM01869|658[0n]bp|Finland.South Karelia|BOLD:ACF0483
Anania coronata|TLMF Lep 07980|658[0n]bp|Austria.Vorarlberg|BOLD:ACF0483
Anania lancealis|TLMF Lep 07981|658[0n]bp|Austria.Vorarlberg|BOLD:AAB9418
Anania lancealis|MM17843|624[1n]bp|Finland|BOLD:AAB9418
Anania lancealis|TLMF Lep 08157|658[0n]bp|Austria.Vorarlberg|BOLD:AAB9418
Anania lancealis|MM17844|658[0n]bp|Finland|BOLD:AAB9418
Anania lancealis|MM17887|658[0n]bp|Finland|BOLD:AAB9418
Anania hortulata|MM01155|658[0n]bp|Finland.South Karelia|BOLD:AAB0989
Anania hortulata|MM01154|658[0n]bp|Finland.South Karelia|BOLD:AAB0989
Anania hortulata|MM15991|658[0n]bp|Finland|BOLD:AAB0989
Anania hortulata|TLMF Lep 08121|658[0n]bp|Austria.Vorarlberg|BOLD:AAB0989
Anania hortulata|MM02937|620[0n]bp|Finland.Northern Ostrobothnia|BOLD:AAB0989
Anania hortulata|MM01851|658[0n]bp|Finland.South Karelia|BOLD:AAB0989
Anania fuscalis|MM01866|658[0n]bp|Finland.South Karelia|BOLD:AAB9417
Anania fuscalis|MM14652|658[0n]bp|Finland|BOLD:AAB9417
Anania fuscalis|MM18399|658[0n]bp|Finland|BOLD:AAB9417
Anania fuscalis|MM01867|658[0n]bp|Finland.South Karelia|BOLD:AAB9417
Anania fuscalis|TLMF Lep 08011|658[0n]bp|Austria.Vorarlberg|BOLD:AAB9417
Anania terrealis|TLMF Lep 08738|658[0n]bp|Austria.Vorarlberg|BOLD:AAC9116
Anania terrealis|MM02447|658[0n]bp|Finland.South Karelia|BOLD:AAC9116
Anania terrealis|MM12978|658[0n]bp|Finland|BOLD:AAC9116
Anania terrealis|MM08366|658[0n]bp|Finland|BOLD:AAC9116
Anania stachydalis|MM06695|658[0n]bp|Finland.Aland Islands|BOLD:AAF3932
Anania stachydalis|MM05244|637[0n]bp|Finland.Finland Proper|BOLD:AAF3932
Anania stachydalis|MM14831|658[0n]bp|Finland|BOLD:AAF3932
Anania stachydalis|TLMF Lep 07517|658[0n]bp|Austria.Vorarlberg|BOLD:AAF3932
Pyrausta aerealis|TLMF Lep 00912|658[0n]bp|Austria.Vorarlberg|BOLD:AAC7368
Pyrausta aerealis|MM17839|636[1n]bp|Finland|BOLD:AAF8527
Anania crocealis|MM09586|658[0n]bp|Finland.Aland Islands|BOLD:AAD7537
Anania crocealis|MM10392|571[0n]bp|Finland.Aland Islands|BOLD:AAD7537
Anania crocealis|TLMF Lep 07961|658[0n]bp|Austria.Vorarlberg|BOLD:AAD7537
Anania crocealis|MM09585|658[0n]bp|Finland.Aland Islands|BOLD:AAD7537
Loxostege sticticalis|MM10564|658[0n]bp|Finland|BOLD:AAB4167
Loxostege sticticalis|TLMF Lep 07998|658[0n]bp|Austria.Vorarlberg|BOLD:AAB4167
[illegible]

Anania crocealis|MM09585|658[0n]bp|Finland.Aland Islands|BOLD:AAD7537
Loxostege sticticalis|MM10564|658[0n]bp|Finland|BOLD:AAB4167
Loxostege sticticalis|TLMF Lep 07998|658[0n]bp|Austria.Vorarlberg|BOLD:AAB4167
Loxostege sticticalis|MM10563|658[0n]bp|Finland|BOLD:AAB4167
Loxostege sticticalis|MM10562|658[0n]bp|Finland|BOLD:AAB4167
Anania funebris|MM18400|658[0n]bp|Finland|BOLD:ACN5052
Anania funebris|MM06366|658[0n]bp|Finland|BOLD:ACN5052
Anania funebris|TLMF Lep 08433|658[0n]bp|Austria.Vorarlberg|BOLD:ACN5052
Anania funebris|MM03248|635[0n]bp|Finland.Northern Ostrobothnia|BOLD:AAB4181
Anania verbascalis|TLMF Lep 07916|658[0n]bp|Austria.Vorarlberg|BOLD:AAF3935
Anania verbascalis|MM17846|658[0n]bp|Finland|BOLD:AAF3935
Anania verbascalis|MM17362|658[0n]bp|Finland|BOLD:AAF3935
Anania verbascalis|MM21144|658[0n]bp|Finland|BOLD:AAF3935
Anania verbascalis|MM17890|658[0n]bp|Finland|BOLD:AAF3935
Anania verbascalis|MM17841|632[0n]bp|Finland|BOLD:AAF3935
Pyrausta cingulata|MM04220|628[0n]bp|Finland.North Karelia|BOLD:ACF2687
Pyrausta cingulata|TLMF Lep 04290|658[0n]bp|Austria.Vorarlberg|BOLD:ACF2687
Pyrausta cingulata|MM03376|658[0n]bp|Finland.Northern Ostrobothnia|BOLD:ACF2687
Pyrausta cingulata|MM04219|658[0n]bp|Finland.North Karelia|BOLD:ACF2687
Pyrausta cingulata|MM18799|658[0n]bp|Finland|BOLD:ACF2687
Pyrausta cingulata|TLMF Lep 09889|658[0n]bp|Austria.Vorarlberg|BOLD:ACF2687
Pyrausta aurata|MM10557|622[0n]bp|Finland|BOLD:AAB6530
Pyrausta aurata|TLMF Lep 08425|658[0n]bp|Austria.Vorarlberg|BOLD:AAB6530
Pyrausta aurata|MM10556|658[0n]bp|Finland|BOLD:AAB6530
Pyrausta aurata|MM10555|658[0n]bp|Finland|BOLD:AAB6530
Pyrausta aurata|MM17277|658[0n]bp|Finland|BOLD:AAB6530
Pyrausta purpuralis|TLMF Lep 09915|658[0n]bp|Austria.Vorarlberg|BOLD:AAB6531
Pyrausta purpuralis|MM01882|658[0n]bp|Finland.South Karelia|BOLD:AAB6531
Pyrausta purpuralis|TLMF Lep 07919|658[0n]bp|Austria.Vorarlberg|BOLD:AAB6531
Pyrausta purpuralis|MM11676|658[0n]bp|Finland|BOLD:AAB6531
Pyrausta purpuralis|MM14677|638[0n]bp|Finland|BOLD:AAB6531
Pyrausta purpuralis|TLMF Lep 08102|658[0n]bp|Austria.Vorarlberg|BOLD:AAB6531
Sitochroa verticalis|MM12934|658[0n]bp|Finland|BOLD:AAB9443
Sitochroa verticalis|MM01870|658[0n]bp|Finland.South Karelia|BOLD:AAB9443
Sitochroa verticalis|TLMF Lep 08005|658[0n]bp|Austria.Vorarlberg|BOLD:AAB9443
Sitochroa verticalis|MM01871|658[0n]bp|Finland.South Karelia|BOLD:AAB9443
Catoptria falsella|MM04958|658[0n]bp|Finland.Finland Proper|BOLD:AAC2294
Catoptria falsella|MM13050|658[0n]bp|Finland|BOLD:AAC2294
Catoptria falsella|MM01920|658[0n]bp|Finland.South Karelia|BOLD:AAC2294
Catoptria falsella|TLMF Lep 08742|658[0n]bp|Austria.Vorarlberg|BOLD:AAC2294
Agriphila inquinatella|TLMF Lep 08730|658[0n]bp|Austria.Vorarlberg|BOLD:ACN8815
Agriphila inquinatella|MM13032|658[0n]bp|Finland|BOLD:ACN8815
Agriphila inquinatella|MM22627|658[0n]bp|Finland|BOLD:ACN8815
Agriphila inquinatella|MM04957|658[0n]bp|Finland.Finland Proper|BOLD:ACN8815
Agriphila inquinatella|MM01886|658[0n]bp|Finland.South Karelia|BOLD:ACN8815
Agriphila straminella|TLMF Lep 08470|658[0n]bp|Austria.Vorarlberg|BOLD:AAC0267
Agriphila straminella|MM13031|658[0n]bp|Finland|BOLD:AAC0267
Agriphila straminella|MM02948|658[0n]bp|Finland.Northern Ostrobothnia|BOLD:AAC0268
Agriphila straminella|MM01913|658[0n]bp|Finland.South Karelia|BOLD:AAC0268
Agriphila straminella|MM11806|658[0n]bp|Finland|BOLD:AAC0268
Agriphila straminella|MM08487|658[0n]bp|Finland|BOLD:AAC0268
Agriphila tristella|TLMF Lep 08732|658[0n]bp|Austria.Vorarlberg|BOLD:AAB9062
Agriphila tristella|MM04963|658[0n]bp|Finland.Finland Proper|BOLD:AAB9062
Agriphila tristella|MM01912|658[0n]bp|Finland.South Karelia|BOLD:AAB9062
Agriphila tristella|MM14676|658[0n]bp|Finland|BOLD:AAB9062
Catoptria permutatellus|MM13028|658[0n]bp|Finland|BOLD:AAC2957
Catoptria permutatellus|MM02950|658[0n]bp|Finland.Northern Ostrobothnia|BOLD:AAC2957
Catoptria permutatellus|TLMF Lep 00911|658[0n]bp|Austria.Vorarlberg|BOLD:AAC2957
Catoptria permutatellus|MM01899|658[0n]bp|Finland.South Karelia|BOLD:AAC2957
Catoptria permutatellus|MM09246|658[0n]bp|Finland|BOLD:AAC2957
Catoptria verellus|MM03554|658[0n]bp|Finland.Kymenlaakso|BOLD:AAD5840
Catoptria verellus|MM06763|658[0n]bp|Finland.Aland Islands|BOLD:AAD5840
Catoptria verellus|MM01922|658[0n]bp|Finland.South Karelia|BOLD:AAD5840
Catoptria verellus|TLMF Lep 08223|658[0n]bp|Austria.Vorarlberg|BOLD:AAD5840
Chrysoteuchia culmella|TLMF Lep 08214|658[0n]bp|Austria.Vorarlberg|BOLD:AAC0540
Chrysoteuchia culmella|MM13049|658[0n]bp|Finland|BOLD:AAC0540
Chrysoteuchia culmella|MM00626|658[0n]bp|Finland.Northern Ostrobothnia|BOLD:AAC0540
Chrysoteuchia culmella|MM01926|603[0n]bp|Finland.South Karelia|BOLD:AAC0540
Crambus silvella|MM21088|658[0n]bp|Finland|BOLD:AAJ6529
Crambus silvella|TLMF Lep 12499|658[0n]bp|Austria.Vorarlberg|BOLD:AAJ6529
Crambus silvella|MM13048|658[0n]bp|Finland|BOLD:AAJ6529
Crambus silvella|MM21087|658[0n]bp|Finland|BOLD:AAJ6529
Crambus lathoniellus|MM01918|658[0n]bp|Finland.South Karelia|BOLD:AAC1691
Crambus lathoniellus|MM00614|632[0n]bp|Finland.Northern Ostrobothnia|BOLD:AAC1691
Crambus lathoniellus|MM11810|658[0n]bp|Finland|BOLD:AAC1691
Crambus lathoniellus|TLMF Lep 08010|658[0n]bp|Austria.Vorarlberg|BOLD:AAC1691
Crambus perlella|MM08284|658[0n]bp|Finland|BOLD:AAA6137
Crambus perlella|MM04976|658[0n]bp|Finland.Finland Proper|BOLD:AAA6137
Crambus perlella|MM06551|658[0n]bp|Finland|BOLD:AAA6137
Crambus perlella|MM01915|620[0n]bp|Finland.South Karelia|BOLD:AAA6137
Crambus perlella|MM02946|614[0n]bp|Finland.Northern Ostrobothnia|BOLD:AAA6137
Crambus perlella|TLMF Lep 08006|658[0n]bp|Austria.Vorarlberg|BOLD:AAA6137
Crambus pascuella|TLMF Lep 08007|658[0n]bp|Austria.Vorarlberg|BOLD:ACF1131
Crambus pascuella|MM10229|658[0n]bp|Finland|BOLD:ACF1131
Crambus pascuella|MM01917|635[0n]bp|Finland.South Karelia|BOLD:ACF1131
Crambus pascuella|MM04975|658[0n]bp|Finland.Finland Proper|BOLD:ACF1131
Crambus pratella|TLMF Lep 09922|658[0n]bp|Austria.Vorarlberg|BOLD:AAA6138
Crambus pratella|MM03258|658[0n]bp|Finland.Northern Ostrobothnia|BOLD:AAA6138
Crambus pratella|MM13038|658[0n]bp|Finland|BOLD:AAA6138
Crambus pratella|MM08095|658[0n]bp|Finland|BOLD:AAA6138
Crambus ericella|TLMF Lep 09955|658[0n]bp|Austria.Vorarlberg|BOLD:AAD6953
Crambus ericella|MM03457|658[0n]bp|Finland.Uusimaa|BOLD:AAD6953
Crambus ericella|MM06368|658[0n]bp|Finland|BOLD:AAD6953
Crambus ericella|MM06369|658[0n]bp|Finland|BOLD:AAD6953
Crambus uliginosellus|MM03363|658[0n]bp|Finland.Northern Ostrobothnia|BOLD:AAD6941
Crambus uliginosellus|MM15787|658[0n]bp|Finland|BOLD:AAD6941
Crambus uliginosellus|TLMF Lep 08215|658[0n]bp|Austria.Vorarlberg|BOLD:AAD6941
Crambus uliginosellus|MM03362|658[0n]bp|Finland.Northern Ostrobothnia|BOLD:AAD6941
Calamotropha paludella|MM01876|614[0n]bp|Finland.South Karelia|BOLD:AAC7277
Calamotropha paludella|TLMF Lep 07532|658[0n]bp|Austria.Vorarlberg|BOLD:AAC7277
Calamotropha paludella|MM03502|658[0n]bp|Finland.Uusimaa|BOLD:AAC7277
Calamotropha paludella|MM13014|658[0n]bp|Finland|BOLD:AAC7277
Chilo phragmitella|MM09750|658[0n]bp|Finland.Aland Islands|BOLD:AAC4987

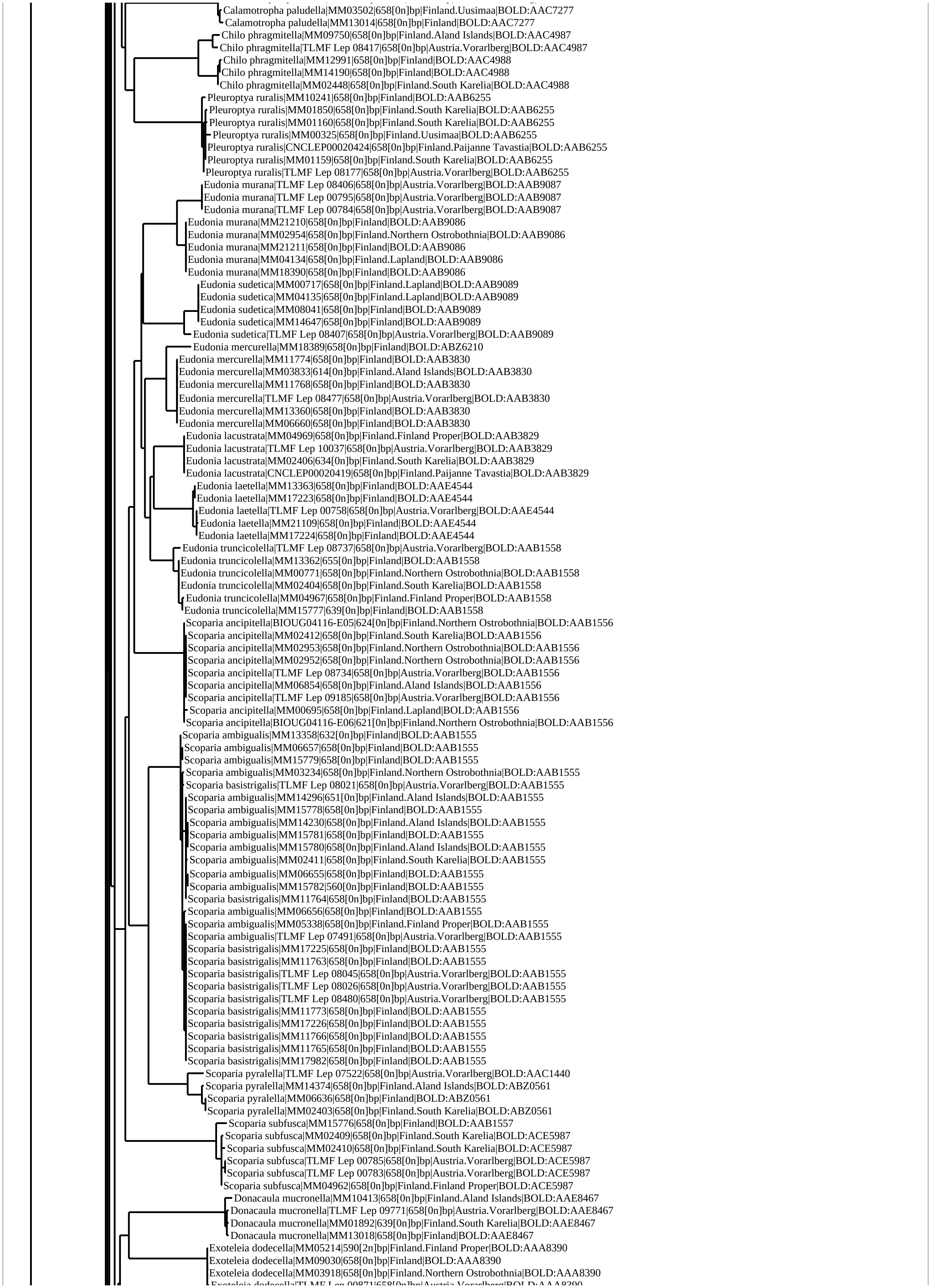
Calamotropha paludella|MM03502|658[0n]bp|Finland.Uusimaa|BOLD:AAC7277
Calamotropha paludella|MM13014|658[0n]bp|Finland|BOLD:AAC7277
Chilo phragmitella|MM09750|658[0n]bp|Finland.Aland Islands|BOLD:AAC4987
Chilo phragmitella|TLMF Lep 08417|658[0n]bp|Austria.Vorarlberg|BOLD:AAC4987
Chilo phragmitella|MM12991|658[0n]bp|Finland|BOLD:AAC4988
Chilo phragmitella|MM14190|658[0n]bp|Finland|BOLD:AAC4988
Chilo phragmitella|MM02448|658[0n]bp|Finland.South Karelia|BOLD:AAC4988
Pleuroptya ruralis|MM10241|658[0n]bp|Finland|BOLD:AAB6255
Pleuroptya ruralis|MM01850|658[0n]bp|Finland.South Karelia|BOLD:AAB6255
Pleuroptya ruralis|MM01160|658[0n]bp|Finland.South Karelia|BOLD:AAB6255
Pleuroptya ruralis|MM00325|658[0n]bp|Finland.Uusimaa|BOLD:AAB6255
Pleuroptya ruralis|CNCLEP00020424|658[0n]bp|Finland.Paijanne Tavastia|BOLD:AAB6255
Pleuroptya ruralis|MM01159|658[0n]bp|Finland.South Karelia|BOLD:AAB6255
Pleuroptya ruralis|TLMF Lep 08177|658[0n]bp|Austria.Vorarlberg|BOLD:AAB6255
Eudonia murana|TLMF Lep 08406|658[0n]bp|Austria.Vorarlberg|BOLD:AAB9087
Eudonia murana|TLMF Lep 00795|658[0n]bp|Austria.Vorarlberg|BOLD:AAB9087
Eudonia murana|TLMF Lep 00784|658[0n]bp|Austria.Vorarlberg|BOLD:AAB9087
Eudonia murana|MM21210|658[0n]bp|Finland|BOLD:AAB9086
Eudonia murana|MM02954|658[0n]bp|Finland.Northern Ostrobothnia|BOLD:AAB9086
Eudonia murana|MM21211|658[0n]bp|Finland|BOLD:AAB9086
Eudonia murana|MM04134|658[0n]bp|Finland.Lapland|BOLD:AAB9086
Eudonia murana|MM18390|658[0n]bp|Finland|BOLD:AAB9086
Eudonia sudetica|MM00717|658[0n]bp|Finland.Lapland|BOLD:AAB9089
Eudonia sudetica|MM04135|658[0n]bp|Finland.Lapland|BOLD:AAB9089
Eudonia sudetica|MM08041|658[0n]bp|Finland|BOLD:AAB9089
Eudonia sudetica|MM14647|658[0n]bp|Finland|BOLD:AAB9089
Eudonia sudetica|TLMF Lep 08407|658[0n]bp|Austria.Vorarlberg|BOLD:AAB9089
Eudonia mercurella|MM18389|658[0n]bp|Finland|BOLD:ABZ6210
Eudonia mercurella|MM11774|658[0n]bp|Finland|BOLD:AAB3830
Eudonia mercurella|MM03833|614[0n]bp|Finland.Aland Islands|BOLD:AAB3830
Eudonia mercurella|MM11768|658[0n]bp|Finland|BOLD:AAB3830
Eudonia mercurella|TLMF Lep 08477|658[0n]bp|Austria.Vorarlberg|BOLD:AAB3830
Eudonia mercurella|MM13360|658[0n]bp|Finland|BOLD:AAB3830
Eudonia mercurella|MM06660|658[0n]bp|Finland|BOLD:AAB3830
Eudonia lacustrata|MM04969|658[0n]bp|Finland.Finland Proper|BOLD:AAB3829
Eudonia lacustrata|TLMF Lep 10037|658[0n]bp|Austria.Vorarlberg|BOLD:AAB3829
Eudonia lacustrata|MM02406|634[0n]bp|Finland.South Karelia|BOLD:AAB3829
Eudonia lacustrata|CNCLEP00020419|658[0n]bp|Finland.Paijanne Tavastia|BOLD:AAB3829
Eudonia laetella|MM13363|658[0n]bp|Finland|BOLD:AAE4544
Eudonia laetella|MM17223|658[0n]bp|Finland|BOLD:AAE4544
Eudonia laetella|TLMF Lep 00758|658[0n]bp|Austria.Vorarlberg|BOLD:AAE4544
Eudonia laetella|MM21109|658[0n]bp|Finland|BOLD:AAE4544
Eudonia laetella|MM17224|658[0n]bp|Finland|BOLD:AAE4544
Eudonia truncicolella|TLMF Lep 08737|658[0n]bp|Austria.Vorarlberg|BOLD:AAB1558
Eudonia truncicolella|MM13362|655[0n]bp|Finland|BOLD:AAB1558
Eudonia truncicolella|MM00771|658[0n]bp|Finland.Northern Ostrobothnia|BOLD:AAB1558
Eudonia truncicolella|MM02404|658[0n]bp|Finland.South Karelia|BOLD:AAB1558
Eudonia truncicolella|MM04967|658[0n]bp|Finland.Finland Proper|BOLD:AAB1558
Eudonia truncicolella|MM15777|639[0n]bp|Finland|BOLD:AAB1558
Scoparia ancipitella|BIOUG04116-E05|624[0n]bp|Finland.Northern Ostrobothnia|BOLD:AAB1556
Scoparia ancipitella|MM02412|658[0n]bp|Finland.South Karelia|BOLD:AAB1556
Scoparia ancipitella|MM02953|658[0n]bp|Finland.Northern Ostrobothnia|BOLD:AAB1556
Scoparia ancipitella|MM02952|658[0n]bp|Finland.Northern Ostrobothnia|BOLD:AAB1556
Scoparia ancipitella|TLMF Lep 08734|658[0n]bp|Austria.Vorarlberg|BOLD:AAB1556
Scoparia ancipitella|MM06854|658[0n]bp|Finland.Aland Islands|BOLD:AAB1556
Scoparia ancipitella|TLMF Lep 09185|658[0n]bp|Austria.Vorarlberg|BOLD:AAB1556
Scoparia ancipitella|MM00695|658[0n]bp|Finland.Lapland|BOLD:AAB1556
Scoparia ancipitella|BIOUG04116-E06|621[0n]bp|Finland.Northern Ostrobothnia|BOLD:AAB1556
Scoparia ambigualis|MM13358|632[0n]bp|Finland|BOLD:AAB1555
Scoparia ambigualis|MM06657|658[0n]bp|Finland|BOLD:AAB1555
Scoparia ambigualis|MM15779|658[0n]bp|Finland|BOLD:AAB1555
Scoparia ambigualis|MM03234|658[0n]bp|Finland.Northern Ostrobothnia|BOLD:AAB1555
Scoparia basistrigalis|TLMF Lep 08021|658[0n]bp|Austria.Vorarlberg|BOLD:AAB1555
Scoparia ambigualis|MM14296|651[0n]bp|Finland.Aland Islands|BOLD:AAB1555
Scoparia ambigualis|MM15778|658[0n]bp|Finland|BOLD:AAB1555
Scoparia ambigualis|MM14230|658[0n]bp|Finland.Aland Islands|BOLD:AAB1555
Scoparia ambigualis|MM15781|658[0n]bp|Finland|BOLD:AAB1555
Scoparia ambigualis|MM15780|658[0n]bp|Finland.Aland Islands|BOLD:AAB1555
Scoparia ambigualis|MM02411|658[0n]bp|Finland.South Karelia|BOLD:AAB1555
Scoparia ambigualis|MM06655|658[0n]bp|Finland|BOLD:AAB1555
Scoparia ambigualis|MM15782|560[0n]bp|Finland|BOLD:AAB1555
Scoparia basistrigalis|MM11764|658[0n]bp|Finland|BOLD:AAB1555
Scoparia ambigualis|MM06656|658[0n]bp|Finland|BOLD:AAB1555
Scoparia ambigualis|MM05338|658[0n]bp|Finland.Finland Proper|BOLD:AAB1555
Scoparia ambigualis|TLMF Lep 07491|658[0n]bp|Austria.Vorarlberg|BOLD:AAB1555
Scoparia basistrigalis|MM17225|658[0n]bp|Finland|BOLD:AAB1555
Scoparia basistrigalis|MM11763|658[0n]bp|Finland|BOLD:AAB1555
Scoparia basistrigalis|TLMF Lep 08045|658[0n]bp|Austria.Vorarlberg|BOLD:AAB1555
Scoparia basistrigalis|TLMF Lep 08026|658[0n]bp|Austria.Vorarlberg|BOLD:AAB1555
Scoparia basistrigalis|TLMF Lep 08480|658[0n]bp|Austria.Vorarlberg|BOLD:AAB1555
Scoparia basistrigalis|MM11773|658[0n]bp|Finland|BOLD:AAB1555
Scoparia basistrigalis|MM17226|658[0n]bp|Finland|BOLD:AAB1555
Scoparia basistrigalis|MM11766|658[0n]bp|Finland|BOLD:AAB1555
Scoparia basistrigalis|MM11765|658[0n]bp|Finland|BOLD:AAB1555
Scoparia basistrigalis|MM17982|658[0n]bp|Finland|BOLD:AAB1555
Scoparia pyralella|TLMF Lep 07522|658[0n]bp|Austria.Vorarlberg|BOLD:AAC1440
Scoparia pyralella|MM14374|658[0n]bp|Finland.Aland Islands|BOLD:ABZ0561
Scoparia pyralella|MM06636|658[0n]bp|Finland|BOLD:ABZ0561
Scoparia pyralella|MM02403|658[0n]bp|Finland.South Karelia|BOLD:ABZ0561
Scoparia subfusca|MM15776|658[0n]bp|Finland|BOLD:AAB1557
Scoparia subfusca|MM02409|658[0n]bp|Finland.South Karelia|BOLD:ACE5987
Scoparia subfusca|MM02410|658[0n]bp|Finland.South Karelia|BOLD:ACE5987
Scoparia subfusca|TLMF Lep 00785|658[0n]bp|Austria.Vorarlberg|BOLD:ACE5987
Scoparia subfusca|TLMF Lep 00783|658[0n]bp|Austria.Vorarlberg|BOLD:ACE5987
Scoparia subfusca|MM04962|658[0n]bp|Finland.Finland Proper|BOLD:ACE5987
Donacaula mucronella|MM10413|658[0n]bp|Finland.Aland Islands|BOLD:AAE8467
Donacaula mucronella|TLMF Lep 09771|658[0n]bp|Austria.Vorarlberg|BOLD:AAE8467
Donacaula mucronella|MM01892|639[0n]bp|Finland.South Karelia|BOLD:AAE8467
Donacaula mucronella|MM13018|658[0n]bp|Finland|BOLD:AAE8467
Exoteleia dodecella|MM05214|590[2n]bp|Finland.Finland Proper|BOLD:AAA8390
Exoteleia dodecella|MM09030|658[0n]bp|Finland|BOLD:AAA8390
Exoteleia dodecella|MM03918|658[0n]bp|Finland.Northern Ostrobothnia|BOLD:AAA8390
Exoteleia dodecella|TLMF Lep 00871|658[0n]bp|Austria.Vorarlberg|BOLD:AAA8390

Exoteleia dodecella|MM0[illegible]|[illegible]bp|Finland.Finland Proper|BOLD:AAA8390
Exoteleia dodecella|MM09030|658[0n]bp|Finland|BOLD:AAA8390
Exoteleia dodecella|MM03918|658[0n]bp|Finland.Northern Ostrobothnia|BOLD:AAA8390
Exoteleia dodecella|TLMF Lep 00871|658[0n]bp|Austria.Vorarlberg|BOLD:AAA8390
Metaxmeste schrankiana|MM13925|658[1n]bp|Finland|BOLD:AAE7792
Metaxmeste schrankiana|MM18800|658[0n]bp|Finland|BOLD:AAE7792
Metaxmeste schrankiana|MM10553|658[0n]bp|Finland|BOLD:AAE7792
Metaxmeste schrankiana|MM10552|658[0n]bp|Finland|BOLD:AAE7792
Metaxmeste schrankiana|TLMF Lep 09977|658[0n]bp|Austria.Vorarlberg|BOLD:AAE7792
Evergestis forficalis|MM12982|658[0n]bp|Finland|BOLD:AAB7956
Evergestis forficalis|MM01218|658[0n]bp|Finland.South Karelia|BOLD:AAB7956
Evergestis forficalis|TLMF Lep 12525|596[0n]bp|Austria.Vorarlberg|BOLD:AAB7956
Evergestis forficalis|MM04956|602[0n]bp|Finland.Finland Proper|BOLD:AAB7956
Evergestis forficalis|MM01874|658[0n]bp|Finland.South Karelia|BOLD:AAB7956
Evergestis pallidata|MM01856|658[0n]bp|Finland.South Karelia|BOLD:AAB4832
Evergestis pallidata|MM02957|658[0n]bp|Finland.Northern Ostrobothnia|BOLD:AAB4832
Evergestis pallidata|TLMF Lep 08731|658[0n]bp|Austria.Vorarlberg|BOLD:AAB4832
Evergestis pallidata|MM01857|658[0n]bp|Finland.South Karelia|BOLD:AAB4832
Nomophila noctuella|MM17341|658[1n]bp|Finland|BOLD:AAA7880
Nomophila noctuella|MM04206|658[0n]bp|Finland.Aland Islands|BOLD:AAA7880
Nomophila noctuella|TLMF Lep 08197|658[0n]bp|Austria.Vorarlberg|BOLD:AAA7880
Nomophila noctuella|TLMF Lep 06175|658[0n]bp|Austria.Vorarlberg|BOLD:AAA7880
Nomophila noctuella|MM05508|658[0n]bp|Finland.Ostrobothnia|BOLD:AAA7880
Diasemia reticularis|TLMF Lep 09938|658[0n]bp|Austria.Vorarlberg|BOLD:AAC3558
Diasemia reticularis|MM10568|658[0n]bp|Finland|BOLD:AAC3558
Diasemia reticularis|MM10569|658[0n]bp|Finland|BOLD:AAC3558
Diasemia reticularis|MM10570|658[0n]bp|Finland|BOLD:AAC3558
Udea accolalis|TLMF Lep 07396|658[0n]bp|Austria.Vorarlberg|BOLD:AAJ7913
Udea accolalis|BC MTD Lep 00765|658[0n]bp|Austria.Vorarlberg|BOLD:AAJ7913
Udea accolalis|MM17851|634[0n]bp|Finland|BOLD:AAJ7913
Udea accolalis|MM21134|658[0n]bp|Finland|BOLD:AAJ7913
Udea accolalis|MM19946|658[0n]bp|Finland.Uusimaa|BOLD:AAJ7913
Udea nebulalis|TLMF Lep 00914|658[0n]bp|Austria.Vorarlberg|BOLD:AAD5509
Udea nebulalis|TLMF Lep 03624|658[0n]bp|Austria.Vorarlberg|BOLD:AAD5509
Udea nebulalis|MM06605|658[0n]bp|Finland|BOLD:AAD5509
Udea nebulalis|MM08327|658[0n]bp|Finland|BOLD:AAD5509
Udea nebulalis|MM13868|642[0n]bp|Finland.Lapland|BOLD:AAD5509
Udea decrepitalis|MM01863|658[0n]bp|Finland.South Karelia|BOLD:ABY5633
Udea decrepitalis|MM02941|658[0n]bp|Finland.Northern Ostrobothnia|BOLD:ABY5633
Udea decrepitalis|MM01862|658[0n]bp|Finland.South Karelia|BOLD:ABY5633
Udea decrepitalis|TLMF Lep 07571|658[0n]bp|Austria.Vorarlberg|BOLD:ABY5633
Udea inquinatalis|MM14586|658[0n]bp|Finland|BOLD:AAB9747
Udea inquinatalis|MM04139|658[0n]bp|Finland.Lapland|BOLD:AAB9747
Udea inquinatalis|MM04133|658[0n]bp|Finland.Lapland|BOLD:AAB9747
Udea inquinatalis|TLMF Lep 02834|658[0n]bp|Austria.Vorarlberg|BOLD:AAB9747
Udea prunalis|TLMF Lep 08568|658[0n]bp|Austria.Vorarlberg|BOLD:AAC2028
Udea prunalis|MM01861|658[0n]bp|Finland.South Karelia|BOLD:AAC2028
Udea prunalis|MM01860|658[0n]bp|Finland.South Karelia|BOLD:AAC2028
Udea prunalis|MM02944|658[0n]bp|Finland.Northern Ostrobothnia|BOLD:AAC2028
Anarsia lineatella|TLMF Lep 08204|658[0n]bp|Austria.Vorarlberg|BOLD:ABZ2446
Anarsia lineatella|MM21099|658[0n]bp|Finland|BOLD:ABZ2446
Anarsia lineatella|MM06832|658[0n]bp|Finland.Aland Islands|BOLD:ABZ2446
Anarsia lineatella|MM17355|658[0n]bp|Finland|BOLD:ABZ2446
Anarsia lineatella|MM05685|658[0n]bp|Finland.Uusimaa|BOLD:ABZ2446
Anarsia lineatella|MM05037|658[0n]bp|Finland.Finland Proper|BOLD:ABZ2446
Gonepteryx rhamni|MM17124|658[0n]bp|Finland|BOLD:AAA9222
Gonepteryx rhamni|TLMF Lep 09810|658[0n]bp|Austria.Vorarlberg|BOLD:AAA9222
Gonepteryx rhamni|MM00941|658[0n]bp|Finland.Northern Ostrobothnia|BOLD:AAA9222
Gonepteryx rhamni|MM06996|658[0n]bp|Finland|BOLD:AAA9222
Helcystogramma rufescens|TLMF Lep 07527|658[0n]bp|Austria.Vorarlberg|BOLD:AAC1177
Helcystogramma rufescens|TLMF Lep 08041|658[0n]bp|Austria.Vorarlberg|BOLD:AAC1177
Helcystogramma rufescens|MM05274|657[0n]bp|Finland.Finland Proper|BOLD:AAC1177
Helcystogramma rufescens|MM03841|658[0n]bp|Finland.Aland Islands|BOLD:AAC1177
Helcystogramma rufescens|MM11981|658[0n]bp|Finland|BOLD:AAC1177
Helcystogramma rufescens|TLMF Lep 04540|658[0n]bp|Austria.Vorarlberg|BOLD:AAC1177
Helcystogramma rufescens|MM03080|658[0n]bp|Finland.Northern Ostrobothnia|BOLD:AAC1177
Helcystogramma rufescens|MM08862|658[0n]bp|Finland|BOLD:AAC1177
Helcystogramma rufescens|MM02273|642[0n]bp|Finland.South Karelia|BOLD:AAC1177
Helcystogramma rufescens|MM02274|628[0n]bp|Finland.South Karelia|BOLD:AAC1177
Lycaena phlaeas|TLMF Lep 09836|658[0n]bp|Austria.Vorarlberg|BOLD:AAA5867
Lycaena phlaeas|MM17119|658[0n]bp|Finland|BOLD:AAA5867
Lycaena phlaeas|MM03698|658[0n]bp|Finland.Uusimaa|BOLD:AAA5867
Lycaena phlaeas|MM10279|658[0n]bp|Finland|BOLD:AAA5867
Lycaena hippothoe|TLMF Lep 09828|658[0n]bp|Austria.Vorarlberg|BOLD:AAB8947
Lycaena hippothoe|TLMF Lep 09829|658[0n]bp|Austria.Vorarlberg|BOLD:AAB8947
Lycaena hippothoe|MM17123|658[0n]bp|Finland|BOLD:AAB8947
Lycaena hippothoe|MM17122|658[0n]bp|Finland|BOLD:AAB8947
Lycaena hippothoe|MM06257|658[0n]bp|Finland|BOLD:AAB8947
Lycaena virgaureae|MM14659|658[0n]bp|Finland|BOLD:AAB4984
Lycaena virgaureae|TLMF Lep 12603|632[0n]bp|Austria.Vorarlberg|BOLD:AAB4984
Lycaena virgaureae|MM00305|642[0n]bp|Finland.Northern Ostrobothnia|BOLD:AAB4984
Lycaena virgaureae|MM17121|658[0n]bp|Finland|BOLD:AAB4984
Callophrys rubi|MM05430|658[0n]bp|Finland.Northern Ostrobothnia|BOLD:AAB0049
Callophrys rubi|MM00386|658[0n]bp|Finland.Northern Ostrobothnia|BOLD:AAB0049
Callophrys rubi|MM17118|658[0n]bp|Finland|BOLD:AAB0049
Callophrys rubi|TLMF Lep 10007|658[0n]bp|Austria.Vorarlberg|BOLD:AAB0049
Cupido minimus|TLMF Lep 08272|658[0n]bp|Austria.Vorarlberg|BOLD:AAA9082
Cupido minimus|MM10580|622[0n]bp|Finland|BOLD:AAA9082
Cupido minimus|MM10579|658[0n]bp|Finland|BOLD:AAA9082
Cupido minimus|MM18666|658[0n]bp|Finland|BOLD:AAA9082
Celastrina argiolus|TLMF Lep 09824|658[0n]bp|Austria.Vorarlberg|BOLD:AAA7663
Celastrina argiolus|MM00488|658[0n]bp|Finland.Northern Ostrobothnia|BOLD:AAA7663
Celastrina argiolus|MM17136|658[0n]bp|Finland|BOLD:AAA7663
Celastrina argiolus|MM17137|658[0n]bp|Finland|BOLD:AAA7663
Glaucopsyche alexis|MM17423|658[0n]bp|Finland|BOLD:AAA5424
Glaucopsyche alexis|MM03860|658[0n]bp|Finland.Finland Proper|BOLD:AAA5424
Glaucopsyche alexis|MM17138|658[0n]bp|Finland|BOLD:AAA5424
Glaucopsyche alexis|MM04237|658[0n]bp|Finland.South Karelia|BOLD:AAA5424
Glaucopsyche alexis|TLMF Lep 09818|658[0n]bp|Austria.Vorarlberg|BOLD:AAA5424
Plebejus argus|MM03336|658[0n]bp|Finland.Northern Ostrobothnia|BOLD:ABZ1727
Plebejus argus|MM09925|658[0n]bp|Finland|BOLD:ABZ1727
Plebejus argus|MM17141|658[0n]bp|Finland|BOLD:ABZ1727
Plebejus argus|TLMF Lep 09809|658[0n]bp|Austria.Vorarlberg|BOLD:ABZ1727

Plebejus argus|MM09925|658[0n]bp|Finland|BOLD:ABZ1727
Plebejus argus|MM17141|658[0n]bp|Finland|BOLD:ABZ1727
Plebejus argus|TLMF Lep 09809|658[0n]bp|Austria.Vorarlberg|BOLD:ABZ1727
Plebejus idas|MM03337|658[0n]bp|Finland.Northern Ostrobothnia|BOLD:AAA3628
Plebejus idas|MM03338|658[0n]bp|Finland.Northern Ostrobothnia|BOLD:AAA3628
Plebejus idas|MM03346|658[0n]bp|Finland.Northern Ostrobothnia|BOLD:AAA3628
Plebejus idas|TLMF Lep 08515|658[0n]bp|Austria.Vorarlberg|BOLD:AAA3628
Aricia artaxerxes|MM09926|658[0n]bp|Finland|BOLD:ACE7893
Aricia artaxerxes|TLMF Lep 08522|658[0n]bp|Austria.Vorarlberg|BOLD:ACE7893
Aricia artaxerxes|BC ZSM Lep 50441|658[0n]bp|Austria.Vorarlberg|BOLD:ACE7893
Aricia artaxerxes|MM17139|658[0n]bp|Finland|BOLD:ACE7893
Aricia artaxerxes|BC ZSM Lep 50439|658[0n]bp|Austria.Vorarlberg|BOLD:ACE7893
Aricia artaxerxes|MM03347|658[0n]bp|Finland.Northern Ostrobothnia|BOLD:ACE7893
Aricia artaxerxes|BC ZSM Lep 50436|658[0n]bp|Austria.Vorarlberg|BOLD:ACE7893
Aricia artaxerxes|TLMF Lep 09841|658[0n]bp|Austria.Vorarlberg|BOLD:ACE7893
Aricia artaxerxes|BC ZSM Lep 50432|658[0n]bp|Austria.Vorarlberg|BOLD:ACE7893
Aricia artaxerxes|BC ZSM Lep 50443|658[0n]bp|Austria.Vorarlberg|BOLD:ACE7893
Aricia artaxerxes|BC ZSM Lep 50434|658[0n]bp|Austria.Vorarlberg|BOLD:ACE7893
Cyaniris semiargus|TLMF Lep 10016|658[0n]bp|Austria.Vorarlberg|BOLD:AAB3027
Cyaniris semiargus|MM17145|658[0n]bp|Finland|BOLD:AAB3027
Cyaniris semiargus|MM17143|658[0n]bp|Finland|BOLD:AAB3027
Eumedonia eumedon|TLMF Lep 06152|658[0n]bp|Austria.Vorarlberg|BOLD:AAC3364
Eumedonia eumedon|TLMF Lep 09843|658[0n]bp|Austria.Vorarlberg|BOLD:AAC3364
Eumedonia eumedon|MM03212|658[0n]bp|Finland.Northern Ostrobothnia|BOLD:AAC3364
Eumedonia eumedon|MM17140|658[0n]bp|Finland|BOLD:AAC3364
Eumedonia eumedon|MM14128|658[0n]bp|Finland|BOLD:AAC3364
Polyommatus icarus|TLMF Lep 08273|658[0n]bp|Austria.Vorarlberg|BOLD:AAA3303
Polyommatus icarus|MM10352|658[0n]bp|Finland|BOLD:AAA3303
Polyommatus icarus|MM17147|658[0n]bp|Finland|BOLD:AAA3303
Polyommatus icarus|MM17146|658[0n]bp|Finland|BOLD:AAA3303
Colias hyale|TLMF Lep 09811|658[0n]bp|Austria.Vorarlberg|BOLD:AAA5531
Colias hyale|MM17133|658[0n]bp|Finland|BOLD:AAA5531
Colias hyale|MM06097|658[0n]bp|Finland|BOLD:AAA5531
Colias palaeno|MM03233|670[0n]bp|Finland.Northern Ostrobothnia|BOLD:AAA3447
Colias palaeno|TLMF Lep 06181|658[0n]bp|Austria.Vorarlberg|BOLD:AAA3447
Colias palaeno|MM17134|658[0n]bp|Finland|BOLD:AAA3447
Colias palaeno|MM03411|658[0n]bp|Finland.Lapland|BOLD:AAA3447
Colias palaeno|MM17135|658[0n]bp|Finland|BOLD:AAA3447
Leptidea juvernica|TLMF Lep 09122|658[0n]bp|Austria.Vorarlberg|BOLD:ABY8343
Leptidea juvernica|MM22892|658[0n]bp|Finland|BOLD:ABY8343
Leptidea juvernica|TLMF Lep 09838|658[0n]bp|Austria.Vorarlberg|BOLD:ABY8343
Leptidea juvernica|TLMF Lep 08294|615[0n]bp|Austria.Vorarlberg|BOLD:ABY8343
Leptidea sinapis|MM13893|658[0n]bp|Finland|BOLD:AAA6298
Leptidea sinapis|MM04238|658[0n]bp|Finland.South Karelia|BOLD:AAA6298
Leptidea sinapis|MM05733|658[0n]bp|Finland.North Karelia|BOLD:AAA6298
Leptidea sinapis|MM18773|658[0n]bp|Finland|BOLD:AAA6298
Leptidea sinapis|MM00498|638[0n]bp|Finland.Northern Ostrobothnia|BOLD:AAA6298
Leptidea sinapis|TLMF Lep 06138|658[0n]bp|Austria.Vorarlberg|BOLD:AAA6298
Leptidea sinapis|MM06100|658[0n]bp|Finland|BOLD:AAA6298
Pyrgus alveus|TLMF Lep 09842|658[0n]bp|Austria.Vorarlberg|BOLD:ABZ6967
Pyrgus alveus|MM06791|658[0n]bp|Finland.Aland Islands|BOLD:ABZ6967
Pyrgus alveus|MM18681|658[0n]bp|Finland|BOLD:ABZ6967
Pyrgus andromedae|TLMF Lep 09802|658[0n]bp|Austria.Vorarlberg|BOLD:ACE9283
Pyrgus andromedae|MM17108|658[1n]bp|Finland|BOLD:ACE9283
Pyrgus andromedae|MM04102|630[0n]bp|Finland.Lapland|BOLD:ACE9283
Pyrgus andromedae|MM17109|658[0n]bp|Finland|BOLD:ACE9283
Pyrgus andromedae|MM10572|631[0n]bp|Finland|BOLD:ACE9283
Pyrgus malvae|MM00029|658[0n]bp|Finland.South Karelia|BOLD:AAB6141
Pyrgus malvae|TLMF Lep 08278|658[0n]bp|Austria.Vorarlberg|BOLD:AAB6141
Pyrgus malvae|TLMF Lep 09832|658[0n]bp|Austria.Vorarlberg|BOLD:AAB6141
Pyrgus malvae|MM00850|658[0n]bp|Finland.Aland Islands|BOLD:AAB6141
Pyrgus malvae|MM00034|670[0n]bp|Finland.Aland Islands|BOLD:AAB6141
Carterocephalus palaemon|MM00511|658[0n]bp|Finland.Northern Ostrobothnia|BOLD:AAA6267
Carterocephalus palaemon|MM17104|658[0n]bp|Finland|BOLD:AAA6267
Carterocephalus palaemon|MM10323|658[0n]bp|Finland|BOLD:AAA6267
Carterocephalus palaemon|MM17103|658[0n]bp|Finland|BOLD:AAA6267
Carterocephalus palaemon|TLMF Lep 09816|658[0n]bp|Austria.Vorarlberg|BOLD:AAA6267
Carterocephalus palaemon|MM10322|658[0n]bp|Finland|BOLD:AAA6267
Hesperia comma|TLMF Lep 09102|625[0n]bp|Austria.Vorarlberg|BOLD:ABX6141
Hesperia comma|MM17115|658[0n]bp|Finland|BOLD:ABX6141
Hesperia comma|MM06896|658[0n]bp|Finland.Aland Islands|BOLD:ABX6141
Hesperia comma|MM17114|658[0n]bp|Finland|BOLD:ABX6141
Ochlodes sylvanus|MM17112|658[0n]bp|Finland|BOLD:AAB4864
Ochlodes sylvanus|MM02929|632[0n]bp|Finland.Northern Ostrobothnia|BOLD:AAB4864
Ochlodes sylvanus|MM02928|648[0n]bp|Finland.Northern Ostrobothnia|BOLD:AAB4864
Ochlodes sylvanus|TLMF Lep 08286|615[0n]bp|Austria.Vorarlberg|BOLD:AAB4864
Thymelicus lineola|TLMF Lep 09830|658[0n]bp|Austria.Vorarlberg|BOLD:AAA6759
Thymelicus lineola|MM17368|658[0n]bp|Finland|BOLD:AAA6760
Thymelicus lineola|MM03333|670[0n]bp|Finland.Northern Ostrobothnia|BOLD:AAA6760
Thymelicus lineola|MM14671|658[0n]bp|Finland|BOLD:AAA6760
Boloria euphrosyne|MM06452|658[0n]bp|Finland|BOLD:AAB1747
Boloria euphrosyne|TLMF Lep 08565|658[0n]bp|Austria.Vorarlberg|BOLD:AAB1747
Boloria euphrosyne|MM17173|658[0n]bp|Finland|BOLD:ACF1010
Boloria euphrosyne|MM18688|658[0n]bp|Finland|BOLD:ACF1010
Boloria selene|TLMF Lep 06155|658[0n]bp|Austria.Vorarlberg|BOLD:AAA5115
Boloria selene|MM17170|658[0n]bp|Finland|BOLD:AAA5115
Boloria selene|MM03274|658[0n]bp|Finland.Northern Ostrobothnia|BOLD:AAA5115
Boloria selene|MM03195|657[0n]bp|Finland.Northern Ostrobothnia|BOLD:AAA5115
Boloria selene|MM17172|658[0n]bp|Finland|BOLD:AAA5115
Boloria thore|MM04093|627[0n]bp|Finland.Lapland|BOLD:ABZ5868
Boloria thore|MM04094|658[1n]bp|Finland.Lapland|BOLD:ABZ5868
Boloria thore|MM03408|658[0n]bp|Finland.Lapland|BOLD:ABZ5868
Boloria thore|TLMF Lep 06154|658[0n]bp|Austria.Vorarlberg|BOLD:ABZ5868
Boloria eunomia|TLMF Lep 09801|658[0n]bp|Austria.Vorarlberg|BOLD:AAA3397
Boloria eunomia|MM17169|658[0n]bp|Finland|BOLD:AAA3397
Boloria eunomia|MM13983|658[0n]bp|Finland|BOLD:AAA3397
Boloria eunomia|MM13982|658[0n]bp|Finland|BOLD:AAA3397
Boloria aquilonaris|MM14566|658[0n]bp|Finland|BOLD:AAA9406
Boloria aquilonaris|TLMF Lep 00746|658[0n]bp|Austria.Vorarlberg|BOLD:AAA9406
Boloria aquilonaris|MM03352|658[0n]bp|Finland.Northern Ostrobothnia|BOLD:AAA9406
Boloria aquilonaris|MM03351|658[0n]bp|Finland.Northern Ostrobothnia|BOLD:AAA9406
Boloria napaea|TLMF Lep 12552|658[0n]bp|Austria.Vorarlberg|BOLD:AAA9406
Boloria napaea|MM03407|658[0n]bp|Finland.Lapland|BOLD:AAA9406

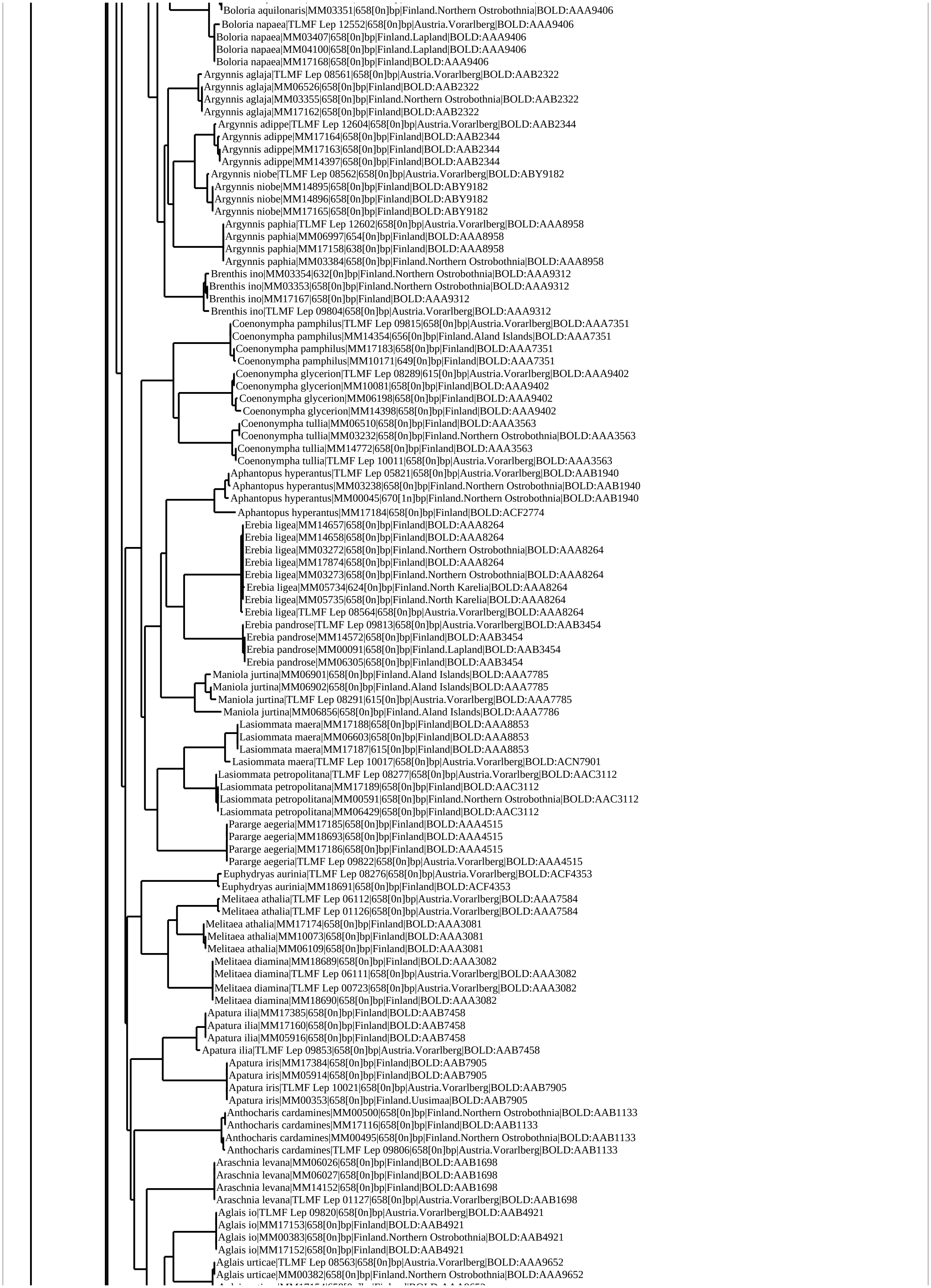

Boloria aquilonaris|MM03351|658[0n]bp|Finland.Northern Ostrobothnia|BOLD:AAA9406
Boloria napaea|TLMF Lep 12552|658[0n]bp|Austria.Vorarlberg|BOLD:AAA9406
Boloria napaea|MM03407|658[0n]bp|Finland.Lapland|BOLD:AAA9406
Boloria napaea|MM04100|658[0n]bp|Finland.Lapland|BOLD:AAA9406
Boloria napaea|MM17168|658[0n]bp|Finland|BOLD:AAA9406
Argynnis aglaja|TLMF Lep 08561|658[0n]bp|Austria.Vorarlberg|BOLD:AAB2322
Argynnis aglaja|MM06526|658[0n]bp|Finland|BOLD:AAB2322
Argynnis aglaja|MM03355|658[0n]bp|Finland.Northern Ostrobothnia|BOLD:AAB2322
Argynnis aglaja|MM17162|658[0n]bp|Finland|BOLD:AAB2322
Argynnis adippe|TLMF Lep 12604|658[0n]bp|Austria.Vorarlberg|BOLD:AAB2344
Argynnis adippe|MM17164|658[0n]bp|Finland|BOLD:AAB2344
Argynnis adippe|MM17163|658[0n]bp|Finland|BOLD:AAB2344
Argynnis adippe|MM14397|658[0n]bp|Finland|BOLD:AAB2344
Argynnis niobe|TLMF Lep 08562|658[0n]bp|Austria.Vorarlberg|BOLD:ABY9182
Argynnis niobe|MM14895|658[0n]bp|Finland|BOLD:ABY9182
Argynnis niobe|MM14896|658[0n]bp|Finland|BOLD:ABY9182
Argynnis niobe|MM17165|658[0n]bp|Finland|BOLD:ABY9182
Argynnis paphia|TLMF Lep 12602|658[0n]bp|Austria.Vorarlberg|BOLD:AAA8958
Argynnis paphia|MM06997|654[0n]bp|Finland|BOLD:AAA8958
Argynnis paphia|MM17158|638[0n]bp|Finland|BOLD:AAA8958
Argynnis paphia|MM03384|658[0n]bp|Finland.Northern Ostrobothnia|BOLD:AAA8958
Brenthis ino|MM03354|632[0n]bp|Finland.Northern Ostrobothnia|BOLD:AAA9312
Brenthis ino|MM03353|658[0n]bp|Finland.Northern Ostrobothnia|BOLD:AAA9312
Brenthis ino|MM17167|658[0n]bp|Finland|BOLD:AAA9312
Brenthis ino|TLMF Lep 09804|658[0n]bp|Austria.Vorarlberg|BOLD:AAA9312
Coenonympha pamphilus|TLMF Lep 09815|658[0n]bp|Austria.Vorarlberg|BOLD:AAA7351
Coenonympha pamphilus|MM14354|656[0n]bp|Finland.Aland Islands|BOLD:AAA7351
Coenonympha pamphilus|MM17183|658[0n]bp|Finland|BOLD:AAA7351
Coenonympha pamphilus|MM10171|649[0n]bp|Finland|BOLD:AAA7351
Coenonympha glycerion|TLMF Lep 08289|615[0n]bp|Austria.Vorarlberg|BOLD:AAA9402
Coenonympha glycerion|MM10081|658[0n]bp|Finland|BOLD:AAA9402
Coenonympha glycerion|MM06198|658[0n]bp|Finland|BOLD:AAA9402
Coenonympha glycerion|MM14398|658[0n]bp|Finland|BOLD:AAA9402
Coenonympha tullia|MM06510|658[0n]bp|Finland|BOLD:AAA3563
Coenonympha tullia|MM03232|658[0n]bp|Finland.Northern Ostrobothnia|BOLD:AAA3563
Coenonympha tullia|MM14772|658[0n]bp|Finland|BOLD:AAA3563
Coenonympha tullia|TLMF Lep 10011|658[0n]bp|Austria.Vorarlberg|BOLD:AAA3563
Aphantopus hyperantus|TLMF Lep 05821|658[0n]bp|Austria.Vorarlberg|BOLD:AAB1940
Aphantopus hyperantus|MM03238|658[0n]bp|Finland.Northern Ostrobothnia|BOLD:AAB1940
Aphantopus hyperantus|MM00045|670[1n]bp|Finland.Northern Ostrobothnia|BOLD:AAB1940
Aphantopus hyperantus|MM17184|658[0n]bp|Finland|BOLD:ACF2774
Erebia ligea|MM14657|658[0n]bp|Finland|BOLD:AAA8264
Erebia ligea|MM14658|658[0n]bp|Finland|BOLD:AAA8264
Erebia ligea|MM03272|658[0n]bp|Finland.Northern Ostrobothnia|BOLD:AAA8264
Erebia ligea|MM17874|658[0n]bp|Finland|BOLD:AAA8264
Erebia ligea|MM03273|658[0n]bp|Finland.Northern Ostrobothnia|BOLD:AAA8264
Erebia ligea|MM05734|624[0n]bp|Finland.North Karelia|BOLD:AAA8264
Erebia ligea|MM05735|658[0n]bp|Finland.North Karelia|BOLD:AAA8264
Erebia ligea|TLMF Lep 08564|658[0n]bp|Austria.Vorarlberg|BOLD:AAA8264
Erebia pandrose|TLMF Lep 09813|658[0n]bp|Austria.Vorarlberg|BOLD:AAB3454
Erebia pandrose|MM14572|658[0n]bp|Finland|BOLD:AAB3454
Erebia pandrose|MM00091|658[0n]bp|Finland.Lapland|BOLD:AAB3454
Erebia pandrose|MM06305|658[0n]bp|Finland|BOLD:AAB3454
Maniola jurtina|MM06901|658[0n]bp|Finland.Aland Islands|BOLD:AAA7785
Maniola jurtina|MM06902|658[0n]bp|Finland.Aland Islands|BOLD:AAA7785
Maniola jurtina|TLMF Lep 08291|615[0n]bp|Austria.Vorarlberg|BOLD:AAA7785
Maniola jurtina|MM06856|658[0n]bp|Finland.Aland Islands|BOLD:AAA7786
Lasiommata maera|MM17188|658[0n]bp|Finland|BOLD:AAA8853
Lasiommata maera|MM06603|658[0n]bp|Finland|BOLD:AAA8853
Lasiommata maera|MM17187|615[0n]bp|Finland|BOLD:AAA8853
Lasiommata maera|TLMF Lep 10017|658[0n]bp|Austria.Vorarlberg|BOLD:ACN7901
Lasiommata petropolitana|TLMF Lep 08277|658[0n]bp|Austria.Vorarlberg|BOLD:AAC3112
Lasiommata petropolitana|MM17189|658[0n]bp|Finland|BOLD:AAC3112
Lasiommata petropolitana|MM00591|658[0n]bp|Finland.Northern Ostrobothnia|BOLD:AAC3112
Lasiommata petropolitana|MM06429|658[0n]bp|Finland|BOLD:AAC3112
Pararge aegeria|MM17185|658[0n]bp|Finland|BOLD:AAA4515
Pararge aegeria|MM18693|658[0n]bp|Finland|BOLD:AAA4515
Pararge aegeria|MM17186|658[0n]bp|Finland|BOLD:AAA4515
Pararge aegeria|TLMF Lep 09822|658[0n]bp|Austria.Vorarlberg|BOLD:AAA4515
Euphydryas aurinia|TLMF Lep 08276|658[0n]bp|Austria.Vorarlberg|BOLD:ACF4353
Euphydryas aurinia|MM18691|658[0n]bp|Finland|BOLD:ACF4353
Melitaea athalia|TLMF Lep 06112|658[0n]bp|Austria.Vorarlberg|BOLD:AAA7584
Melitaea athalia|TLMF Lep 01126|658[0n]bp|Austria.Vorarlberg|BOLD:AAA7584
Melitaea athalia|MM17174|658[0n]bp|Finland|BOLD:AAA3081
Melitaea athalia|MM10073|658[0n]bp|Finland|BOLD:AAA3081
Melitaea athalia|MM06109|658[0n]bp|Finland|BOLD:AAA3081
Melitaea diamina|MM18689|658[0n]bp|Finland|BOLD:AAA3082
Melitaea diamina|TLMF Lep 06111|658[0n]bp|Austria.Vorarlberg|BOLD:AAA3082
Melitaea diamina|TLMF Lep 00723|658[0n]bp|Austria.Vorarlberg|BOLD:AAA3082
Melitaea diamina|MM18690|658[0n]bp|Finland|BOLD:AAA3082
Apatura ilia|MM17385|658[0n]bp|Finland|BOLD:AAB7458
Apatura ilia|MM17160|658[0n]bp|Finland|BOLD:AAB7458
Apatura ilia|MM05916|658[0n]bp|Finland|BOLD:AAB7458
Apatura ilia|TLMF Lep 09853|658[0n]bp|Austria.Vorarlberg|BOLD:AAB7458
Apatura iris|MM17384|658[0n]bp|Finland|BOLD:AAB7905
Apatura iris|MM05914|658[0n]bp|Finland|BOLD:AAB7905
Apatura iris|TLMF Lep 10021|658[0n]bp|Austria.Vorarlberg|BOLD:AAB7905
Apatura iris|MM00353|658[0n]bp|Finland.Uusimaa|BOLD:AAB7905
Anthocharis cardamines|MM00500|658[0n]bp|Finland.Northern Ostrobothnia|BOLD:AAB1133
Anthocharis cardamines|MM17116|658[0n]bp|Finland|BOLD:AAB1133
Anthocharis cardamines|MM00495|658[0n]bp|Finland.Northern Ostrobothnia|BOLD:AAB1133
Anthocharis cardamines|TLMF Lep 09806|658[0n]bp|Austria.Vorarlberg|BOLD:AAB1133
Araschnia levana|MM06026|658[0n]bp|Finland|BOLD:AAB1698
Araschnia levana|MM06027|658[0n]bp|Finland|BOLD:AAB1698
Araschnia levana|MM14152|658[0n]bp|Finland|BOLD:AAB1698
Araschnia levana|TLMF Lep 01127|658[0n]bp|Austria.Vorarlberg|BOLD:AAB1698
Aglais io|TLMF Lep 09820|658[0n]bp|Austria.Vorarlberg|BOLD:AAB4921
Aglais io|MM17153|658[0n]bp|Finland|BOLD:AAB4921
Aglais io|MM00383|658[0n]bp|Finland.Northern Ostrobothnia|BOLD:AAB4921
Aglais io|MM17152|658[0n]bp|Finland|BOLD:AAB4921
Aglais urticae|TLMF Lep 08563|658[0n]bp|Austria.Vorarlberg|BOLD:AAA9652
Aglais urticae|MM00382|658[0n]bp|Finland.Northern Ostrobothnia|BOLD:AAA9652

Aglais io|MM17152|658[0n]bp|Finland|BOLD:AAB4921
Aglais urticae|TLMF Lep 08563|658[0n]bp|Austria.Vorarlberg|BOLD:AAA9652
Aglais urticae|MM00382|658[0n]bp|Finland.Northern Ostrobothnia|BOLD:AAA9652
Aglais urticae|MM17154|658[0n]bp|Finland|BOLD:AAA9652
Aglais urticae|MM00381|634[0n]bp|Finland.Northern Ostrobothnia|BOLD:AAA9652
Polygonia c-album|MM00384|658[0n]bp|Finland.Northern Ostrobothnia|BOLD:ABY7043
Polygonia c-album|MM00385|633[0n]bp|Finland.Northern Ostrobothnia|BOLD:ABY7043
Polygonia c-album|TLMF Lep 09812|650[0n]bp|Austria.Vorarlberg|BOLD:ABY7043
Polygonia c-album|MM17161|658[0n]bp|Finland|BOLD:ABY7043
Vanessa atalanta|MM14193|658[0n]bp|Finland|BOLD:AAA8638
Vanessa atalanta|MM00953|632[0n]bp|Finland.Northern Ostrobothnia|BOLD:AAA8638
Vanessa atalanta|TLMF Lep 09851|658[0n]bp|Austria.Vorarlberg|BOLD:AAA8638
Vanessa atalanta|MM17151|658[0n]bp|Finland|BOLD:AAA8638
Vanessa cardui|MM17150|658[0n]bp|Finland|BOLD:AAA5337
Vanessa cardui|MM00952|621[0n]bp|Finland.Northern Ostrobothnia|BOLD:AAA5337
Vanessa cardui|MM13949|658[0n]bp|Finland|BOLD:AAA5337
Vanessa cardui|TLMF Lep 09852|658[0n]bp|Austria.Vorarlberg|BOLD:AAA5337
Cerura vinula|TLMF Lep 06180|658[0n]bp|Austria.Vorarlberg|BOLD:AAB7277
Cerura vinula|TLMF Lep 07549|658[0n]bp|Austria.Vorarlberg|BOLD:AAB7277
Cerura vinula|MM18517|658[0n]bp|Finland|BOLD:AAB7277
Cerura vinula|MM11053|658[0n]bp|Finland|BOLD:AAB7277
Cerura vinula|MM10749|649[0n]bp|Finland|BOLD:AAB7277
Cerura vinula|MM00980|658[0n]bp|Finland.South Karelia|BOLD:AAB7277
Cerura vinula|MM05422|658[0n]bp|Finland.Aland Islands|BOLD:AAB7277
Cerura vinula|MM18516|658[0n]bp|Finland|BOLD:AAB7277
Furcula furcula|MM15844|658[0n]bp|Finland|BOLD:ACF2827
Furcula furcula|TLMF Lep 04655|658[0n]bp|Austria.Vorarlberg|BOLD:ACF2827
Furcula furcula|MM07680|658[0n]bp|Finland|BOLD:ACF2827
Furcula furcula|MM15845|658[0n]bp|Finland|BOLD:ACF2827
Mompha locupletella|TLMF Lep 12519|658[0n]bp|Austria.Vorarlberg|BOLD:AAF2616
Mompha locupletella|MM14441|658[0n]bp|Finland|BOLD:AAF2616
Mompha locupletella|MM17956|658[0n]bp|Finland|BOLD:AAF2616
Mompha locupletella|MM14442|656[0n]bp|Finland|BOLD:AAF2616
Mompha raschkiella|TLMF Lep 10053|658[0n]bp|Austria.Vorarlberg|BOLD:AAE2679
Mompha raschkiella|MM13617|658[0n]bp|Finland|BOLD:AAE2679
Mompha raschkiella|MM00537|658[0n]bp|Finland.Northern Ostrobothnia|BOLD:AAE2679
Mompha raschkiella|MM02579|658[0n]bp|Finland.South Karelia|BOLD:AAE2679
Mompha terminella|TLMF Lep 09161|658[0n]bp|Austria.Vorarlberg|BOLD:AAZ8091
Mompha terminella|MM05725|658[0n]bp|Finland|BOLD:AAZ8091
Aglia tau|MM00321|670[0n]bp|Finland.Uusimaa|BOLD:AAB6586
Aglia tau|MM00017|658[0n]bp|Finland.North Karelia|BOLD:AAB6586
Aglia tau|TLMF Lep 07823|658[0n]bp|Austria.Vorarlberg|BOLD:AAB6586
Aglia tau|MM10715|634[0n]bp|Finland|BOLD:AAB6586
Phalera bucephala|MM09382|652[0n]bp|Finland|BOLD:AAC0945
Phalera bucephala|MM00982|670[0n]bp|Finland.South Karelia|BOLD:AAC0945
Phalera bucephala|TLMF Lep 07826|658[0n]bp|Austria.Vorarlberg|BOLD:AAC0945
Phalera bucephala|MM00122|609[0n]bp|Finland.Uusimaa|BOLD:AAC0945
Phalera bucephala|MM00983|658[0n]bp|Finland.South Karelia|BOLD:AAC0945
Brachmia blandella|MM08656|658[0n]bp|Finland.Aland Islands|BOLD:AAD2457
Brachmia blandella|TLMF Lep 08466|658[0n]bp|Austria.Vorarlberg|BOLD:AAD2457
Brachmia blandella|MM17582|658[0n]bp|Finland|BOLD:AAD2457
Brachmia blandella|MM18253|658[0n]bp|Finland|BOLD:AAD2457
Brachmia blandella|TLMF Lep 08203|658[0n]bp|Austria.Vorarlberg|BOLD:AAD2457
Brachmia inornatella|MM17279|658[0n]bp|Finland|BOLD:AAI8301
Brachmia inornatella|TLMF Lep 12493|618[0n]bp|Austria.Vorarlberg|BOLD:AAI8301
Brachmia inornatella|MM15649|658[0n]bp|Finland|BOLD:AAI8301
Brachmia inornatella|TLMF Lep 12492|658[0n]bp|Austria.Vorarlberg|BOLD:AAI8301
Brachmia inornatella|MM17581|658[0n]bp|Finland|BOLD:AAI8301
Brachmia inornatella|MM17580|658[0n]bp|Finland|BOLD:AAI8301
Rivula sericealis|MM11634|658[0n]bp|Finland|BOLD:ABX5628
Rivula sericealis|MM01404|670[0n]bp|Finland.South Karelia|BOLD:ABX5628
Rivula sericealis|MM02905|658[0n]bp|Finland|BOLD:AAB4777
Rivula sericealis|MM05176|658[0n]bp|Finland.Finland Proper|BOLD:AAB4777
Rivula sericealis|MM00324|615[0n]bp|Finland.Uusimaa|BOLD:AAB4777
Rivula sericealis|MM01409|658[0n]bp|Finland.South Karelia|BOLD:AAB4777
Rivula sericealis|MM07817|634[0n]bp|Finland|BOLD:AAB4777
Rivula sericealis|MM02904|658[0n]bp|Finland.Northern Ostrobothnia|BOLD:AAB4777
Rivula sericealis|TLMF Lep 07898|658[0n]bp|Austria.Vorarlberg|BOLD:AAB4777
Acompsia cinerella|MM05048|658[0n]bp|Finland.Finland Proper|BOLD:AAD0078
Acompsia cinerella|MM13521|658[0n]bp|Finland|BOLD:AAD0078
Acompsia cinerella|MM02545|658[0n]bp|Finland.South Karelia|BOLD:AAD0078
Acompsia cinerella|TLMF Lep 08103|658[0n]bp|Austria.Vorarlberg|BOLD:AAD0078
Acompsia cinerella|TLMF Lep 00903|658[0n]bp|Austria.Vorarlberg|BOLD:AAD0078
Acompsia cinerella|TLMF Lep 00907|658[0n]bp|Austria.Vorarlberg|BOLD:AAD0078
Stauropus fagi|MM00981|670[0n]bp|Finland.South Karelia|BOLD:AAD0646
Stauropus fagi|MM00851|658[0n]bp|Finland.Aland Islands|BOLD:AAD0646
Stauropus fagi|MM08373|658[0n]bp|Finland|BOLD:AAD0646
Stauropus fagi|MM04544|658[0n]bp|Finland.Finland Proper|BOLD:AAD0646
Stauropus fagi|TLMF Lep 04656|658[0n]bp|Austria.Vorarlberg|BOLD:AAD0646
Falcaria lacertinaria|MM01258|658[0n]bp|Finland.South Karelia|BOLD:AAA6343
Falcaria lacertinaria|MM01257|658[0n]bp|Finland.South Karelia|BOLD:AAA6343
Falcaria lacertinaria|TLMF Lep 06178|658[0n]bp|Austria.Vorarlberg|BOLD:AAA6343
Falcaria lacertinaria|TLMF Lep 09224|658[0n]bp|Austria.Vorarlberg|BOLD:AAA6343
Falcaria lacertinaria|MM08167|658[0n]bp|Finland|BOLD:AAA6343
Falcaria lacertinaria|MM01366|658[0n]bp|Finland.South Karelia|BOLD:AAA6343
Nola confusalis|MM03773|658[0n]bp|Finland.Aland Islands|BOLD:AAB5563
Nola confusalis|MM13887|658[0n]bp|Finland|BOLD:AAB5563
Nola confusalis|MM07911|658[0n]bp|Finland|BOLD:AAB5563
Nola confusalis|TLMF Lep 04644|658[0n]bp|Austria.Vorarlberg|BOLD:AAB5563
Arctornis l-nigrum|MM15923|658[0n]bp|Finland|BOLD:AAD9740
Arctornis l-nigrum|MM15922|632[1n]bp|Finland|BOLD:AAD9740
Arctornis l-nigrum|TLMF Lep 06115|658[0n]bp|Austria.Vorarlberg|BOLD:AAD9740
Dicallomera fascelina|MM15848|658[0n]bp|Finland|BOLD:AAE7512
Dicallomera fascelina|MM06746|658[0n]bp|Finland.Aland Islands|BOLD:AAE7512
Dicallomera fascelina|TLMF Lep 06103|658[0n]bp|Austria.Vorarlberg|BOLD:AAE7512
Dicallomera fascelina|MM08578|658[0n]bp|Finland|BOLD:AAE7512
Euclidia mi|TLMF Lep 09846|634[0n]bp|Austria.Vorarlberg|BOLD:AAD6424
Euclidia mi|MM00025|637[0n]bp|Finland.South Karelia|BOLD:AAD6424
Euclidia mi|MM00361|658[0n]bp|Finland|BOLD:AAD6424
Euclidia mi|MM05469|670[0n]bp|Finland.Northern Ostrobothnia|BOLD:AAD6424
Euclidia mi|MM00026|658[0n]bp|Finland.South Karelia|BOLD:AAD6424
Orgyia antiqua|MM09339|658[0n]bp|Finland|BOLD:AAA6432
Orgyia antiqua|MM04770|658[0n]bp|Finland.Finland Proper|BOLD:AAA6432

Euclidia mi|MM00026|658[0n]bp|Finland.South Karelia|BOLD:AAD6424
Orgyia antiqua|MM09339|658[0n]bp|Finland|BOLD:AAA6432
Orgyia antiqua|MM04770|658[0n]bp|Finland.Finland Proper|BOLD:AAA6432
Orgyia antiqua|TLMF Lep 12460|658[0n]bp|Austria.Vorarlberg|BOLD:AAA6432
Orgyia antiqua|MM12217|658[0n]bp|Finland|BOLD:AAA6432
Calliteara pudibunda|MM01045|633[0n]bp|Finland.South Karelia|BOLD:AAB5204
Calliteara pudibunda|MM00859|658[0n]bp|Finland.Aland Islands|BOLD:AAB5204
Calliteara pudibunda|MM01046|658[0n]bp|Finland.South Karelia|BOLD:AAB5204
Calliteara pudibunda|TLMF Lep 07827|658[0n]bp|Austria.Vorarlberg|BOLD:AAB5204
Pterostoma palpina|TLMF Lep 07830|658[0n]bp|Austria.Vorarlberg|BOLD:AAB5417
Pterostoma palpina|MM08247|658[0n]bp|Finland|BOLD:AAB5417
Pterostoma palpina|MM14081|658[0n]bp|Finland|BOLD:AAB5417
Pterostoma palpina|MM02794|658[0n]bp|Finland.Northern Ostrobothnia|BOLD:AAB5417
Pterostoma palpina|MM00991|658[0n]bp|Finland.South Karelia|BOLD:AAB5417
Pterostoma palpina|MM00663|658[0n]bp|Finland|BOLD:AAB5417
Pterostoma palpina|MM00990|658[0n]bp|Finland.South Karelia|BOLD:AAB5417
Pterostoma palpina|MM10850|658[0n]bp|Finland|BOLD:AAB5417
Pterostoma palpina|MM04598|658[0n]bp|Finland.Finland Proper|BOLD:AAB5417
Pterostoma palpina|MM12580|658[0n]bp|Finland|BOLD:AAB5417
Pterostoma palpina|MM05423|658[0n]bp|Finland.Aland Islands|BOLD:AAB5417
Odontosia carmelita|TLMF Lep 04625|658[0n]bp|Austria.Vorarlberg|BOLD:AAD7238
Odontosia carmelita|MM00987|658[0n]bp|Finland.South Karelia|BOLD:AAD7238
Odontosia carmelita|MM04501|658[0n]bp|Finland.Finland Proper|BOLD:AAD7238
Odontosia carmelita|MM00988|658[0n]bp|Finland.South Karelia|BOLD:AAD7238
Pheosia gnoma|MM00997|658[0n]bp|Finland.South Karelia|BOLD:AAC5101
Pheosia gnoma|MM05106|658[0n]bp|Finland.Finland Proper|BOLD:AAC5101
Pheosia gnoma|MM09913|658[0n]bp|Finland|BOLD:AAC5101
Pheosia gnoma|MM00996|658[0n]bp|Finland.South Karelia|BOLD:AAC5101
Pheosia gnoma|TLMF Lep 08485|658[0n]bp|Austria.Vorarlberg|BOLD:AAC5101
Pheosia tremula|MM00995|658[0n]bp|Finland.South Karelia|BOLD:AAB5913
Pheosia tremula|MM00994|658[0n]bp|Finland.South Karelia|BOLD:AAB5913
Pheosia tremula|MM08333|658[0n]bp|Finland|BOLD:AAB5913
Pheosia tremula|TLMF Lep 08790|658[0n]bp|Austria.Vorarlberg|BOLD:AAB5913
Ptilodon capucina|TLMF Lep 07879|658[0n]bp|Austria.Vorarlberg|BOLD:AAC0728
Ptilodon capucina|MM02792|658[0n]bp|Finland.Northern Ostrobothnia|BOLD:AAC0728
Ptilodon capucina|MM00992|658[0n]bp|Finland.South Karelia|BOLD:AAC0728
Ptilodon capucina|MM00993|658[0n]bp|Finland.South Karelia|BOLD:AAC0728
Saturnia pavonia|MM12537|658[0n]bp|Finland|BOLD:AAA8149
Saturnia pavonia|MM00002|670[0n]bp|Finland.Northern Ostrobothnia|BOLD:AAA8149
Saturnia pavonia|TLMF Lep 04435|658[0n]bp|Austria.Vorarlberg|BOLD:AAA8149
Saturnia pavonia|TLMF Lep 04436|658[0n]bp|Austria.Vorarlberg|BOLD:AAA8149
Catocala fraxini|MM02788|658[0n]bp|Finland.Northern Ostrobothnia|BOLD:AAC6489
Catocala fraxini|MM01839|633[0n]bp|Finland.South Karelia|BOLD:AAC6489
Catocala fraxini|TLMF Lep 12607|658[0n]bp|Austria.Vorarlberg|BOLD:AAC6489
Catocala fraxini|MM01840|658[0n]bp|Finland.South Karelia|BOLD:AAC6489
Catocala nupta|MM15851|658[0n]bp|Finland|BOLD:ACE8723
Catocala nupta|TLMF Lep 09855|658[0n]bp|Austria.Vorarlberg|BOLD:ACE8723
Catocala nupta|MM04216|658[0n]bp|Finland.Aland Islands|BOLD:ACE8723
Catocala nupta|MM04750|658[0n]bp|Finland.Finland Proper|BOLD:ACE8723
Stathmopoda pedella|CNCLEP00020448|658[0n]bp|Finland.Paijanne Tavastia|BOLD:AAD4282
Stathmopoda pedella|MM02178|609[0n]bp|Finland.South Karelia|BOLD:AAD4282
Stathmopoda pedella|MM13872|658[0n]bp|Finland.Lapland|BOLD:AAD4282
Stathmopoda pedella|MM02351|670[0n]bp|Finland.South Karelia|BOLD:AAD4282
Stathmopoda pedella|TLMF Lep 08229|658[0n]bp|Austria.Vorarlberg|BOLD:AAD4282
Lymantria monacha|MM00138|599[0n]bp|Finland|BOLD:AAA5537
Lymantria monacha|MM01050|658[0n]bp|Finland.South Karelia|BOLD:AAA5537
Lymantria monacha|MM01048|670[0n]bp|Finland.South Karelia|BOLD:AAA5537
Lymantria monacha|MM12596|658[0n]bp|Finland|BOLD:AAA5537
Lymantria monacha|TLMF Lep 08507|658[0n]bp|Austria.Vorarlberg|BOLD:AAA5537
Lymantria monacha|MM03651|658[0n]bp|Finland.Uusimaa|BOLD:AAA5537
Meganola strigula|TLMF Lep 08558|658[0n]bp|Austria.Vorarlberg|BOLD:AAD8314
Meganola strigula|MM04673|658[0n]bp|Finland.Finland Proper|BOLD:AAD8314
Meganola strigula|MM06723|658[0n]bp|Finland.Aland Islands|BOLD:AAD8314
Meganola strigula|MM09551|658[0n]bp|Finland.Aland Islands|BOLD:AAD8314
Nycteola degenerana|MM00135|670[0n]bp|Finland.Uusimaa|BOLD:AAE2641
Nycteola degenerana|TLMF Lep 04645|658[0n]bp|Austria.Vorarlberg|BOLD:AAE2641
Nycteola degenerana|MM01835|658[0n]bp|Finland.South Karelia|BOLD:AAE2641
Nycteola degenerana|MM04623|658[0n]bp|Finland.Finland Proper|BOLD:AAE2641
Nycteola degenerana|MM01834|658[0n]bp|Finland.South Karelia|BOLD:AAE2641
Nycteola degenerana|BIOUG04490-E10|623[0n]bp|Finland.Northern Ostrobothnia|BOLD:AAE2641
Nycteola degenerana|MM02930|658[0n]bp|Finland.Northern Ostrobothnia|BOLD:AAE2641
Nycteola revayana|MM05998|658[0n]bp|Finland.Aland Islands|BOLD:AAB8993
Nycteola revayana|TLMF Lep 07908|658[0n]bp|Austria.Vorarlberg|BOLD:AAB8993
Nycteola revayana|MM18660|658[0n]bp|Finland|BOLD:AAB8993
Nycteola revayana|MM14490|658[0n]bp|Finland|BOLD:AAB8993
Nycteola revayana|MM05407|636[0n]bp|Finland.Finland Proper|BOLD:AAB8993
Herminia tarsicrinalis|TLMF Lep 08125|658[0n]bp|Austria.Vorarlberg|BOLD:AAC1537
Herminia tarsicrinalis|MM11056|658[0n]bp|Finland|BOLD:AAC1537
Herminia tarsicrinalis|MM17220|658[0n]bp|Finland|BOLD:AAC1537
Herminia grisealis|MM01421|643[0n]bp|Finland.South Karelia|BOLD:AAC3337
Herminia grisealis|MM01422|621[0n]bp|Finland.South Karelia|BOLD:AAC3337
Herminia grisealis|MM04679|639[0n]bp|Finland.Finland Proper|BOLD:AAC3337
Herminia grisealis|TLMF Lep 08166|658[0n]bp|Austria.Vorarlberg|BOLD:AAC3337
Herminia grisealis|TLMF Lep 04646|658[0n]bp|Austria.Vorarlberg|BOLD:AAC3337
Herminia tarsipennalis|MM18520|658[0n]bp|Finland|BOLD:AAC1538
Herminia tarsipennalis|MM04587|658[0n]bp|Finland.Finland Proper|BOLD:AAC1538
Herminia tarsipennalis|MM01418|635[0n]bp|Finland.South Karelia|BOLD:AAC1538
Herminia tarsipennalis|TLMF Lep 08124|658[0n]bp|Austria.Vorarlberg|BOLD:AAC1538
Hypena crassalis|MM04576|630[0n]bp|Finland.Finland Proper|BOLD:AAD1366
Hypena crassalis|MM01708|647[0n]bp|Finland.South Karelia|BOLD:AAD1366
Hypena crassalis|MM01709|658[1n]bp|Finland.South Karelia|BOLD:AAD1366
Hypena crassalis|MM17458|658[0n]bp|Finland|BOLD:AAD1366
Hypena crassalis|TLMF Lep 07578|658[0n]bp|Austria.Vorarlberg|BOLD:AAD1366
Hypena proboscidalis|MM01545|670[0n]bp|Finland.South Karelia|BOLD:AAB6485
Hypena proboscidalis|MM02769|658[0n]bp|Finland.Northern Ostrobothnia|BOLD:AAB6485
Hypena proboscidalis|MM01830|658[0n]bp|Finland.South Karelia|BOLD:AAB6485
Hypena proboscidalis|TLMF Lep 08116|658[0n]bp|Austria.Vorarlberg|BOLD:AAB6485
Hypena proboscidalis|MM01546|658[0n]bp|Finland.South Karelia|BOLD:AAB6485
Lithosia quadra|MM14423|658[0n]bp|Finland|BOLD:AAB8723
Lithosia quadra|MM17198|658[0n]bp|Finland|BOLD:AAB8723
Lithosia quadra|MM14422|658[0n]bp|Finland|BOLD:AAB8723
Lithosia quadra|TLMF Lep 08513|658[0n]bp|Austria.Vorarlberg|BOLD:AAB8723
Cybosia mesomella|MM01030|658[0n]bp|Finland.South Karelia|BOLD:AAD1825

[illegible]
Lithosia quadra|MM14422|658[0n]bp|Finland|BOLD:AAB8723
Lithosia quadra|TLMF Lep 08513|658[0n]bp|Austria.Vorarlberg|BOLD:AAB8723
Cybosia mesomella|MM01030|658[0n]bp|Finland.South Karelia|BOLD:AAD1825
Cybosia mesomella|MM02909|658[0n]bp|Finland.Northern Ostrobothnia|BOLD:AAD1825
Cybosia mesomella|MM01029|658[0n]bp|Finland.South Karelia|BOLD:AAD1825
Cybosia mesomella|TLMF Lep 06114|658[0n]bp|Austria.Vorarlberg|BOLD:AAD1825
Atolmis rubricollis|TLMF Lep 07851|658[0n]bp|Austria.Vorarlberg|BOLD:AAD2377
Atolmis rubricollis|MM01018|658[0n]bp|Finland.South Karelia|BOLD:AAD2377
Atolmis rubricollis|MM01017|658[0n]bp|Finland.South Karelia|BOLD:AAD2377
Atolmis rubricollis|MM04554|658[0n]bp|Finland.Finland Proper|BOLD:AAD2377
Eilema sororcula|TLMF Lep 07862|658[0n]bp|Austria.Vorarlberg|BOLD:AAL5773
Eilema sororcula|MM07930|658[0n]bp|Finland|BOLD:AAC1077
Eilema sororcula|MM01831|658[0n]bp|Finland.South Karelia|BOLD:AAC1077
Eilema sororcula|MM03862|658[0n]bp|Finland.South Karelia|BOLD:AAC1077
Eilema depressa|TLMF Lep 08554|658[0n]bp|Austria.Vorarlberg|BOLD:AAB6834
Eilema depressa|MM01032|658[0n]bp|Finland.South Karelia|BOLD:AAB6834
Eilema depressa|MM04890|658[0n]bp|Finland.Finland Proper|BOLD:AAB6834
Eilema depressa|MM01031|658[0n]bp|Finland.South Karelia|BOLD:AAB6834
Eilema depressa|MM04891|658[0n]bp|Finland.Finland Proper|BOLD:AAB6834
Eilema griseola|TLMF Lep 08539|658[0n]bp|Austria.Vorarlberg|BOLD:AAC1074
Eilema griseola|TLMF Lep 08794|658[0n]bp|Austria.Vorarlberg|BOLD:AAC1074
Eilema griseola|MM11628|658[0n]bp|Finland|BOLD:AAC1074
Eilema griseola|MM01164|658[0n]bp|Finland.South Karelia|BOLD:AAC1074
Eilema griseola|MM01026|658[0n]bp|Finland.South Karelia|BOLD:AAC1074
Eilema complana|MM01025|658[0n]bp|Finland.South Karelia|BOLD:AAB6846
Eilema complana|MM01024|658[0n]bp|Finland.South Karelia|BOLD:AAB6846
Eilema complana|MM04867|658[0n]bp|Finland.Finland Proper|BOLD:AAB6846
Eilema complana|TLMF Lep 06113|658[0n]bp|Austria.Vorarlberg|BOLD:AAB6846
Eilema lurideola|MM17199|658[0n]bp|Finland|BOLD:AAB6833
Eilema lurideola|TLMF Lep 08198|658[0n]bp|Austria.Vorarlberg|BOLD:AAB6833
Eilema lurideola|MM01022|658[0n]bp|Finland.South Karelia|BOLD:AAB6833
Eilema lurideola|MM01023|658[0n]bp|Finland.South Karelia|BOLD:AAB6833
Eilema lurideola|MM20534|658[0n]bp|Finland.Uusimaa|BOLD:AAB6833
Setema cereola|TLMF Lep 06120|658[0n]bp|Austria.Vorarlberg|BOLD:AAJ7543
Setema cereola|TLMF Lep 00294|658[0n]bp|Austria.Vorarlberg|BOLD:AAJ7543
Setema cereola|MM17452|658[0n]bp|Finland|BOLD:AAJ7543
Setema cereola|MM19914|658[0n]bp|Finland.Southern Savonia|BOLD:AAJ7543
Setema cereola|MM15929|658[0n]bp|Finland|BOLD:AAJ7543
Setema cereola|MM21031|658[0n]bp|Finland|BOLD:AAJ7543
Setina irrorella|TLMF Lep 00306|658[0n]bp|Austria.Vorarlberg|BOLD:ACF4655
Setina irrorella|TLMF Lep 06119|658[0n]bp|Austria.Vorarlberg|BOLD:ACF4655
Setina irrorella|MM17197|658[0n]bp|Finland|BOLD:ABZ4612
Setina irrorella|MM05904|649[0n]bp|Finland.Lapland|BOLD:ABZ4612
Setina irrorella|MM10589|658[0n]bp|Finland|BOLD:ABZ4612
Arctia caja|TLMF Lep 06116|658[0n]bp|Austria.Vorarlberg|BOLD:AAA8530
Arctia caja|MM09741|658[0n]bp|Finland.Aland Islands|BOLD:AAA8530
Arctia caja|MM04921|658[0n]bp|Finland.Finland Proper|BOLD:AAA8530
Arctia caja|MM07925|658[0n]bp|Finland|BOLD:AAA8530
Arctia caja|MM20723|658[1n]bp|Finland.North Karelia|BOLD:AAA8530
Parasemia plantaginis|MM03197|658[0n]bp|Finland.Northern Ostrobothnia|BOLD:AAB6883
Parasemia plantaginis|TLMF Lep 09845|658[0n]bp|Austria.Vorarlberg|BOLD:AAB6883
Parasemia plantaginis|MM10075|658[0n]bp|Finland|BOLD:AAB6883
Parasemia plantaginis|MM10138|655[0n]bp|Finland|BOLD:AAB6883
Diacrisia sannio|TLMF Lep 07550|658[0n]bp|Austria.Vorarlberg|BOLD:AAB8660
Diacrisia sannio|MM01044|658[0n]bp|Finland.South Karelia|BOLD:AAB8660
Diacrisia sannio|MM01043|658[0n]bp|Finland.South Karelia|BOLD:AAB8660
Diacrisia sannio|MM05163|632[0n]bp|Finland.Finland Proper|BOLD:AAB8660
Rhyparia purpurata|TLMF Lep 05623|658[0n]bp|Austria.Vorarlberg|BOLD:AAD5293
Rhyparia purpurata|MM08355|658[0n]bp|Finland|BOLD:AAD5293
Rhyparia purpurata|MM00323|658[0n]bp|Finland|BOLD:AAD5293
Rhyparia purpurata|MM08354|658[0n]bp|Finland|BOLD:AAD5293
Phragmatobia fuliginosa|MM04920|658[0n]bp|Finland.Finland Proper|BOLD:AAA6178
Phragmatobia fuliginosa|TLMF Lep 07848|658[0n]bp|Austria.Vorarlberg|BOLD:AAA6178
Phragmatobia fuliginosa|MM01042|658[0n]bp|Finland.South Karelia|BOLD:AAA6178
Phragmatobia fuliginosa|MM01041|658[0n]bp|Finland.South Karelia|BOLD:AAA6178
Phragmatobia fuliginosa|MM06360|658[0n]bp|Finland|BOLD:AAA6178
Phragmatobia fuliginosa|MM00672|658[0n]bp|Finland.Lapland|BOLD:AAA6178
Spilosoma lubricipeda|MM00858|670[0n]bp|Finland.Aland Islands|BOLD:AAB2233
Spilosoma lubricipeda|MM01040|658[0n]bp|Finland.South Karelia|BOLD:AAB2233
Spilosoma lubricipeda|MM04659|658[0n]bp|Finland.Finland Proper|BOLD:AAB2233
Spilosoma lubricipeda|MM01039|658[0n]bp|Finland.South Karelia|BOLD:AAB2233
Spilosoma lubricipeda|TLMF Lep 06159|658[0n]bp|Austria.Vorarlberg|BOLD:AAB2233
Spilosoma lutea|TLMF Lep 08280|658[0n]bp|Austria.Vorarlberg|BOLD:AAC0134
Spilosoma lutea|MM01036|658[0n]bp|Finland.South Karelia|BOLD:AAC0134
Spilosoma lutea|MM05171|658[0n]bp|Finland.Finland Proper|BOLD:AAC0134
Spilosoma lutea|MM01035|658[0n]bp|Finland.South Karelia|BOLD:AAC0134
Scoliopteryx libatrix|MM08201|658[0n]bp|Finland|BOLD:ACE7197
Scoliopteryx libatrix|MM00407|670[0n]bp|Finland|BOLD:ACE7197
Scoliopteryx libatrix|TLMF Lep 08183|658[0n]bp|Austria.Vorarlberg|BOLD:ACE7197
Scoliopteryx libatrix|MM14733|658[0n]bp|Finland.Aland Islands|BOLD:ACE7197
Scoliopteryx libatrix|MM14732|658[0n]bp|Finland.Aland Islands|BOLD:ACE7197
Scoliopteryx libatrix|TLMF Lep 04639|658[0n]bp|Austria.Vorarlberg|BOLD:ACE7197
Callimorpha dominula|MM17454|658[0n]bp|Finland|BOLD:AAD5343
Callimorpha dominula|TLMF Lep 00722|658[0n]bp|Austria.Vorarlberg|BOLD:AAD5343
Callimorpha dominula|MM17453|658[0n]bp|Finland|BOLD:AAD5343
Tyria jacobaeae|MM10597|658[0n]bp|Finland|BOLD:AAB5189
Tyria jacobaeae|TLMF Lep 04129|658[0n]bp|Austria.Vorarlberg|BOLD:AAB5189
Tyria jacobaeae|TLMF Lep 04128|658[0n]bp|Austria.Vorarlberg|BOLD:AAB5189
Heliothis nubigera|TLMF Lep 04620|658[0n]bp|Austria.Vorarlberg|BOLD:AAI6741
Heliothis nubigera|MM11113|658[0n]bp|Finland|BOLD:AAI6741
Heliothis nubigera|TLMF Lep 06174|658[0n]bp|Austria.Vorarlberg|BOLD:AAI6741
Heliothis peltigera|TLMF Lep 04621|658[0n]bp|Austria.Vorarlberg|BOLD:AAC6990
Heliothis peltigera|MM15938|648[0n]bp|Finland|BOLD:AAC6990
Earias clorana|MM06650|670[0n]bp|Finland.Uusimaa|BOLD:AAC7224
Earias clorana|MM14434|658[0n]bp|Finland|BOLD:AAC7224
Earias clorana|MM00114|658[0n]bp|Finland.Uusimaa|BOLD:AAC7224
Earias clorana|TLMF Lep 08542|658[0n]bp|Austria.Vorarlberg|BOLD:AAC7224
Acronicta leporina|MM00856|658[0n]bp|Finland.Aland Islands|BOLD:AAC2384
Acronicta leporina|MM10910|658[0n]bp|Finland|BOLD:AAC2384
Acronicta leporina|TLMF Lep 04664|658[0n]bp|Austria.Vorarlberg|BOLD:AAC2384
Acronicta leporina|MM01525|658[0n]bp|Finland.South Karelia|BOLD:AAC2384
Acronicta leporina|MM02754|658[0n]bp|Finland.Northern Ostrobothnia|BOLD:AAC2384
[illegible]

Acronicta leporina|TLMF Lep 04664|658[0n]bp|Austria.Vorarlberg|BOLD:AAC2384
Acronicta leporina|MM01525|658[0n]bp|Finland.South Karelia|BOLD:AAC2384
Acronicta leporina|MM02754|658[0n]bp|Finland.Northern Ostrobothnia|BOLD:AAC2384
Acronicta alni|MM22911|658[0n]bp|Finland|BOLD:AAC3196
Acronicta alni|MM23151|658[0n]bp|Finland|BOLD:AAC3196
Acronicta alni|MM23153|658[0n]bp|Finland|BOLD:AAC3196
Acronicta alni|TLMF Lep 04629|658[0n]bp|Austria.Vorarlberg|BOLD:AAC3196
Acronicta alni|MM22914|658[0n]bp|Finland|BOLD:AAC3196
Acronicta alni|MM22913|658[0n]bp|Finland|BOLD:AAC3196
Acronicta alni|MM22912|658[0n]bp|Finland|BOLD:AAC3196
Acronicta alni|MM22910|658[0n]bp|Finland|BOLD:AAC3196
Acronicta alni|MM22909|658[0n]bp|Finland|BOLD:AAC3196
Acronicta alni|MM23152|658[0n]bp|Finland|BOLD:AAC3196
Acronicta alni|MM18529|658[0n]bp|Finland.North Karelia|BOLD:AAC3196
Acronicta alni|MM23150|658[0n]bp|Finland|BOLD:AAC3196
Acronicta alni|MM11171|658[0n]bp|Finland|BOLD:AAC3196
Acronicta alni|MM04232|658[0n]bp|Finland.South Karelia|BOLD:AAC3196
Acronicta auricoma|MM04363|658[0n]bp|Finland.Finland Proper|BOLD:ACF2281
Acronicta auricoma|MM06306|658[0n]bp|Finland|BOLD:ACF2281
Acronicta auricoma|MM08162|658[0n]bp|Finland|BOLD:ACF2281
Acronicta auricoma|TLMF Lep 04628|658[0n]bp|Austria.Vorarlberg|BOLD:ACF2281
Acronicta euphorbiae|MM09126|658[2n]bp|Finland.Aland Islands|BOLD:AAC6993
Acronicta euphorbiae|MM09125|658[2n]bp|Finland.Aland Islands|BOLD:AAC6993
Acronicta euphorbiae|TLMF Lep 07576|658[0n]bp|Austria.Vorarlberg|BOLD:AAC6993
Acronicta cuspis|MM07390|658[0n]bp|Finland|BOLD:AAF6047
Acronicta cuspis|MM18530|658[0n]bp|Finland.Aland Islands|BOLD:AAF6047
Acronicta cuspis|TLMF Lep 10000|658[0n]bp|Austria.Vorarlberg|BOLD:AAF6047
Acronicta cuspis|MM06741|658[0n]bp|Finland.Aland Islands|BOLD:AAF6047
Acronicta psi|MM12604|658[0n]bp|Finland|BOLD:AAB3300
Acronicta psi|MM03858|658[0n]bp|Finland.Aland Islands|BOLD:AAB3300
Acronicta psi|MM01544|658[0n]bp|Finland.South Karelia|BOLD:AAB3300
Acronicta psi|MM01526|658[0n]bp|Finland.South Karelia|BOLD:AAB3300
Acronicta psi|TLMF Lep 04665|658[0n]bp|Austria.Vorarlberg|BOLD:AAB3300
Acronicta psi|MM04539|658[0n]bp|Finland.Finland Proper|BOLD:AAB3300
Acronicta rumicis|TLMF Lep 07855|658[0n]bp|Austria.Vorarlberg|BOLD:AAC2556
Acronicta rumicis|MM04657|658[0n]bp|Finland.Finland Proper|BOLD:AAC2556
Acronicta rumicis|MM01535|658[0n]bp|Finland.South Karelia|BOLD:AAC2556
Acronicta rumicis|MM03386|658[0n]bp|Finland.Aland Islands|BOLD:AAC2556
Acronicta rumicis|MM01529|670[0n]bp|Finland.South Karelia|BOLD:AAC2556
Allophyes oxyacanthae|TLMF Lep 06141|658[0n]bp|Austria.Vorarlberg|BOLD:AAC3170
Allophyes oxyacanthae|MM04213|658[0n]bp|Finland.Aland Islands|BOLD:AAC3170
Allophyes oxyacanthae|MM17378|658[0n]bp|Finland|BOLD:AAC3170
Allophyes oxyacanthae|MM01744|658[0n]bp|Finland.South Karelia|BOLD:AAC3170
Panemeria tenebrata|MM00005|670[0n]bp|Finland.South Karelia|BOLD:AAD0566
Panemeria tenebrata|MM00007|658[0n]bp|Finland.South Karelia|BOLD:AAD0566
Panemeria tenebrata|MM09978|658[0n]bp|Finland|BOLD:AAD0566
Panemeria tenebrata|TLMF Lep 09860|658[0n]bp|Austria.Vorarlberg|BOLD:AAD0566
Panemeria tenebrata|MM00006|658[0n]bp|Finland.South Karelia|BOLD:AAD0566
Abrostola triplasia|MM05143|658[0n]bp|Finland.Finland Proper|BOLD:AAC8312
Abrostola triplasia|MM01244|658[0n]bp|Finland.South Karelia|BOLD:AAC8312
Abrostola triplasia|TLMF Lep 08199|658[0n]bp|Austria.Vorarlberg|BOLD:AAC8312
Abrostola triplasia|MM01245|658[0n]bp|Finland.South Karelia|BOLD:ACE9950
Euchalcia variabilis|TLMF Lep 00307|658[0n]bp|Austria.Vorarlberg|BOLD:AAE1616
Euchalcia variabilis|MM15933|570[0n]bp|Finland|BOLD:AAP7449
Euchalcia variabilis|MM15934|658[0n]bp|Finland|BOLD:AAP7449
Lamprotes c-aureum|TLMF Lep 10025|658[0n]bp|Austria.Vorarlberg|BOLD:AAE6100
Lamprotes c-aureum|MM11063|651[0n]bp|Finland|BOLD:AAE6100
Lamprotes c-aureum|MM00018|658[0n]bp|Finland.North Karelia|BOLD:AAE6100
Lamprotes c-aureum|MM00019|658[0n]bp|Finland.North Karelia|BOLD:AAE6100
Lamprotes c-aureum|MM00301|658[0n]bp|Finland.North Karelia|BOLD:AAE6100
Lamprotes c-aureum|MM03795|647[0n]bp|Finland.North Karelia|BOLD:AAE6100
Polychrysia moneta|MM01123|658[0n]bp|Finland.South Karelia|BOLD:AAD3729
Polychrysia moneta|MM01166|658[0n]bp|Finland.South Karelia|BOLD:AAD3729
Polychrysia moneta|TLMF Lep 04615|658[0n]bp|Austria.Vorarlberg|BOLD:AAD3729
Polychrysia moneta|MM01122|658[0n]bp|Finland.South Karelia|BOLD:AAD3729
Diachrysia chrysitis|MM01113|622[0n]bp|Finland.South Karelia|BOLD:AAA6511
Diachrysia chrysitis|MM01090|622[0n]bp|Finland.South Karelia|BOLD:AAA6511
Diachrysia chrysitis|MM01092|658[0n]bp|Finland.South Karelia|BOLD:AAA6511
Diachrysia chrysitis|MM01115|622[0n]bp|Finland.South Karelia|BOLD:AAA6511
Diachrysia chrysitis|MM01089|658[0n]bp|Finland.South Karelia|BOLD:AAA6511
Diachrysia chrysitis|MM10772|658[0n]bp|Finland|BOLD:AAA6511
Diachrysia chrysitis|MM02748|658[0n]bp|Finland.Northern Ostrobothnia|BOLD:AAA6511
Diachrysia chrysitis|MM10774|658[0n]bp|Finland|BOLD:AAA6511
Diachrysia chrysitis|MM07385|658[0n]bp|Finland|BOLD:AAA6511
Diachrysia chrysitis|MM04597|658[0n]bp|Finland.Finland Proper|BOLD:AAA6511
Diachrysia chrysitis|MM04736|658[0n]bp|Finland.Finland Proper|BOLD:AAA6511
Diachrysia chrysitis|MM07384|658[0n]bp|Finland|BOLD:AAA6511
Diachrysia chrysitis|MM04595|658[0n]bp|Finland.Finland Proper|BOLD:AAA6511
Diachrysia chrysitis|MM07316|658[0n]bp|Finland|BOLD:AAA6511
Diachrysia chrysitis|MM07314|658[0n]bp|Finland|BOLD:AAA6511
Diachrysia chrysitis|MM07315|658[0n]bp|Finland|BOLD:AAA6511
Diachrysia chrysitis|MM07312|658[0n]bp|Finland|BOLD:AAA6511
Diachrysia chrysitis|MM07383|658[0n]bp|Finland|BOLD:AAA6511
Diachrysia chrysitis|MM04594|658[0n]bp|Finland.Finland Proper|BOLD:AAA6511
Diachrysia chrysitis|MM02749|658[0n]bp|Finland.Northern Ostrobothnia|BOLD:AAA6511
Diachrysia chrysitis|TLMF Lep 08151|658[0n]bp|Austria.Vorarlberg|BOLD:AAA6511
Diachrysia chrysitis|MM10773|658[0n]bp|Finland|BOLD:AAA6511
Diachrysia chrysitis|MM01104|658[0n]bp|Finland.South Karelia|BOLD:AAA6511
Diachrysia chrysitis|MM01105|658[0n]bp|Finland.South Karelia|BOLD:AAA6511
Diachrysia chrysitis|MM07313|658[0n]bp|Finland|BOLD:AAA6511
Diachrysia chrysitis|MM04592|658[0n]bp|Finland.Finland Proper|BOLD:AAA6511
Diachrysia chrysitis|MM04591|658[0n]bp|Finland.Finland Proper|BOLD:AAA6511
Diachrysia chrysitis|MM04590|658[0n]bp|Finland.Finland Proper|BOLD:AAA6511
Diachrysia chrysitis|MM04571|658[0n]bp|Finland.Finland Proper|BOLD:AAA6511
Diachrysia chrysitis|MM01112|658[0n]bp|Finland.South Karelia|BOLD:AAA6511
Diachrysia chrysitis|MM01111|658[0n]bp|Finland.South Karelia|BOLD:AAA6511
Diachrysia chrysitis|MM04547|658[0n]bp|Finland.Finland Proper|BOLD:AAA6511
Diachrysia chrysitis|MM04546|658[0n]bp|Finland.Finland Proper|BOLD:AAA6511
Diachrysia chrysitis|MM04545|658[0n]bp|Finland.Finland Proper|BOLD:AAA6511
Diachrysia chrysitis|MM01091|658[0n]bp|Finland.South Karelia|BOLD:AAA6511
Diachrysia chrysitis|MM06639|657[0n]bp|Finland|BOLD:AAA6511
Diachrysia chrysitis|MM01088|658[0n]bp|Finland.South Karelia|BOLD:AAA6511
Diachrysia chrysitis|MM12628|658[0n]bp|Finland|BOLD:AAA6511

Diachrysia chrysitis|MM06639|657[0n]bp|Finland|BOLD:AAA6511
Diachrysia chrysitis|MM01088|658[0n]bp|Finland.South Karelia|BOLD:AAA6511
Diachrysia chrysitis|MM12628|658[0n]bp|Finland|BOLD:AAA6511
Diachrysia chrysitis|MM12629|658[0n]bp|Finland|BOLD:AAA6511
Diachrysia chrysitis|MM01165|621[0n]bp|Finland.South Karelia|BOLD:AAA6511
Diachrysia chrysitis|MM01103|636[0n]bp|Finland.South Karelia|BOLD:AAA6511
Diachrysia chrysitis|MM06977|657[0n]bp|Finland|BOLD:AAA6511
Diachrysia chrysitis|MM04596|658[0n]bp|Finland.Finland Proper|BOLD:AAA6511
Diachrysia chrysitis|MM10771|658[0n]bp|Finland|BOLD:AAA6511
Diachrysia chrysitis|MM10770|658[0n]bp|Finland|BOLD:AAA6511
Diachrysia chrysitis|MM01114|658[0n]bp|Finland.South Karelia|BOLD:AAA6511
Diachrysia chrysitis|MM04593|658[0n]bp|Finland.Finland Proper|BOLD:AAA6511
Diachrysia chrysitis|MM01641|658[0n]bp|Finland.South Karelia|BOLD:AAA6511
Macdunnoughia confusa|MM12215|658[0n]bp|Finland|BOLD:AAD5865
Macdunnoughia confusa|TLMF Lep 08195|658[0n]bp|Austria.Vorarlberg|BOLD:AAD5865
Macdunnoughia confusa|MM18525|658[0n]bp|Finland|BOLD:AAD5865
Macdunnoughia confusa|MM12657|658[0n]bp|Finland|BOLD:AAD5865
Autographa gamma|TLMF Lep 08167|658[0n]bp|Austria.Vorarlberg|BOLD:AAB4345
Autographa gamma|MM00328|609[0n]bp|Finland.Uusimaa|BOLD:AAB4345
Autographa gamma|MM04361|658[0n]bp|Finland.Finland Proper|BOLD:AAB4345
Autographa gamma|MM10744|658[0n]bp|Finland|BOLD:AAB4345
Autographa gamma|MM04637|658[0n]bp|Finland.Finland Proper|BOLD:AAB4345
Autographa gamma|MM12593|658[0n]bp|Finland|BOLD:AAB4345
Autographa bractea|MM01233|658[0n]bp|Finland.South Karelia|BOLD:AAD2827
Autographa bractea|MM01234|658[0n]bp|Finland.South Karelia|BOLD:AAD2827
Autographa bractea|TLMF Lep 06206|658[0n]bp|Austria.Vorarlberg|BOLD:AAD2827
Autographa bractea|MM09746|658[0n]bp|Finland.Aland Islands|BOLD:AAD2827
Autographa bractea|MM01232|658[0n]bp|Finland.South Karelia|BOLD:AAD2827
Autographa jota|MM09788|658[0n]bp|Finland.Aland Islands|BOLD:AAA3991
Autographa jota|TLMF Lep 07565|658[0n]bp|Austria.Vorarlberg|BOLD:AAA3991
Autographa jota|MM12511|658[0n]bp|Finland|BOLD:AAA3991
Autographa jota|MM09789|658[0n]bp|Finland.Aland Islands|BOLD:AAA3991
Autographa jota|MM04527|658[0n]bp|Finland.Finland Proper|BOLD:AAA3991
Autographa jota|MM04528|658[0n]bp|Finland.Finland Proper|BOLD:AAA3991
Autographa jota|MM10734|658[0n]bp|Finland|BOLD:AAA3991
Autographa jota|MM04529|658[0n]bp|Finland.Finland Proper|BOLD:AAA3991
Autographa jota|MM04526|658[0n]bp|Finland.Finland Proper|BOLD:AAA3991
Autographa jota|MM01183|658[0n]bp|Finland.South Karelia|BOLD:AAA3991
Autographa pulchrina|MM08447|658[0n]bp|Finland|BOLD:AAA3990
Autographa pulchrina|MM08326|658[0n]bp|Finland|BOLD:AAA3990
Autographa pulchrina|MM14084|658[0n]bp|Finland|BOLD:AAA3990
Autographa pulchrina|MM04525|658[0n]bp|Finland.Finland Proper|BOLD:AAA3990
Autographa pulchrina|MM09786|658[0n]bp|Finland.Aland Islands|BOLD:AAA3990
Autographa pulchrina|MM01202|658[0n]bp|Finland.South Karelia|BOLD:AAA3990
Autographa pulchrina|MM04522|658[0n]bp|Finland.Finland Proper|BOLD:AAA3990
Autographa pulchrina|MM02750|658[0n]bp|Finland.Northern Ostrobothnia|BOLD:AAA3990
Autographa pulchrina|MM08448|658[0n]bp|Finland|BOLD:AAA3990
Autographa pulchrina|MM08325|653[0n]bp|Finland|BOLD:AAA3990
Autographa pulchrina|MM08303|574[0n]bp|Finland|BOLD:AAA3990
Autographa pulchrina|MM13862|658[0n]bp|Finland.Lapland|BOLD:AAA3990
Autographa pulchrina|MM02751|658[0n]bp|Finland.Northern Ostrobothnia|BOLD:AAA3990
Autographa pulchrina|MM13876|658[0n]bp|Finland.Lapland|BOLD:AAA3990
Autographa pulchrina|MM13877|658[0n]bp|Finland.Lapland|BOLD:AAA3990
Autographa pulchrina|MM01197|658[0n]bp|Finland.South Karelia|BOLD:AAA3990
Autographa pulchrina|MM01194|633[0n]bp|Finland.South Karelia|BOLD:AAA3990
Autographa pulchrina|MM12595|658[0n]bp|Finland|BOLD:AAA3990
Autographa pulchrina|MM10719|567[0n]bp|Finland|BOLD:AAA3990
Autographa pulchrina|MM01200|654[0n]bp|Finland.South Karelia|BOLD:AAA3990
Autographa pulchrina|MM10720|655[0n]bp|Finland|BOLD:AAA3990
Autographa pulchrina|MM06978|658[0n]bp|Finland|BOLD:AAA3990
Autographa pulchrina|MM07759|658[0n]bp|Finland|BOLD:AAA3990
Autographa pulchrina|MM09787|658[0n]bp|Finland.Aland Islands|BOLD:AAA3990
Autographa pulchrina|MM06684|658[0n]bp|Finland.Aland Islands|BOLD:AAA3990
Autographa pulchrina|MM04519|658[0n]bp|Finland.Finland Proper|BOLD:AAA3990
Autographa pulchrina|MM01199|655[0n]bp|Finland.South Karelia|BOLD:AAA3990
Autographa pulchrina|MM07381|658[0n]bp|Finland|BOLD:AAA3990
Autographa pulchrina|MM09739|658[0n]bp|Finland.Aland Islands|BOLD:AAA3990
Autographa pulchrina|MM09738|658[0n]bp|Finland.Aland Islands|BOLD:AAA3990
Autographa pulchrina|MM05524|658[0n]bp|Finland.Ostrobothnia|BOLD:AAA3990
Autographa pulchrina|MM01191|658[0n]bp|Finland.South Karelia|BOLD:AAA3990
Autographa pulchrina|MM05503|658[0n]bp|Finland.Ostrobothnia|BOLD:AAA3990
Autographa pulchrina|MM04524|658[0n]bp|Finland.Finland Proper|BOLD:AAA3990
Autographa pulchrina|MM04523|658[0n]bp|Finland.Finland Proper|BOLD:AAA3990
Autographa pulchrina|MM01201|658[0n]bp|Finland.South Karelia|BOLD:AAA3990
Autographa pulchrina|MM01186|658[0n]bp|Finland.South Karelia|BOLD:AAA3990
Autographa pulchrina|MM01196|658[0n]bp|Finland.South Karelia|BOLD:AAA3990
Autographa pulchrina|MM01195|658[0n]bp|Finland.South Karelia|BOLD:AAA3990
Autographa pulchrina|MM10133|658[0n]bp|Finland|BOLD:AAA3990
Autographa pulchrina|MM10127|658[0n]bp|Finland|BOLD:AAA3990
Autographa pulchrina|MM14492|658[0n]bp|Finland|BOLD:AAA3990
Autographa pulchrina|MM14481|658[0n]bp|Finland|BOLD:AAA3990
Autographa pulchrina|MM01188|658[0n]bp|Finland.South Karelia|BOLD:AAA3990
Autographa pulchrina|MM04516|658[0n]bp|Finland.Finland Proper|BOLD:AAA3990
Autographa pulchrina|MM01189|658[0n]bp|Finland.South Karelia|BOLD:AAA3990
Autographa pulchrina|MM04521|626[0n]bp|Finland.Finland Proper|BOLD:AAA3990
Autographa pulchrina|MM07382|623[0n]bp|Finland|BOLD:AAA3990
Autographa pulchrina|MM10717|641[0n]bp|Finland|BOLD:AAA3990
Autographa pulchrina|MM10724|641[0n]bp|Finland|BOLD:AAA3990
Autographa pulchrina|MM01198|633[0n]bp|Finland.South Karelia|BOLD:AAA3990
Autographa pulchrina|MM01193|631[0n]bp|Finland.South Karelia|BOLD:AAA3990
Autographa pulchrina|MM01192|621[0n]bp|Finland.South Karelia|BOLD:AAA3990
Autographa pulchrina|MM07947|658[0n]bp|Finland|BOLD:AAA3990
Autographa pulchrina|MM04518|658[0n]bp|Finland.Finland Proper|BOLD:AAA3990
Autographa pulchrina|MM10721|658[0n]bp|Finland|BOLD:AAA3990
Autographa pulchrina|TLMF Lep 04433|658[0n]bp|Austria.Vorarlberg|BOLD:AAA3990
Autographa pulchrina|MM12614|658[0n]bp|Finland|BOLD:AAA3990
Autographa pulchrina|MM04561|658[0n]bp|Finland.Finland Proper|BOLD:AAA3990
Autographa pulchrina|MM12594|658[0n]bp|Finland|BOLD:AAA3990
Autographa pulchrina|MM04517|658[0n]bp|Finland.Finland Proper|BOLD:AAA3990
Autographa pulchrina|MM04515|658[0n]bp|Finland.Finland Proper|BOLD:AAA3990
Autographa pulchrina|TLMF Lep 00295|658[0n]bp|Austria.Vorarlberg|BOLD:AAA3990
Autographa pulchrina|MM06685|658[0n]bp|Finland.Aland Islands|BOLD:AAA3990
Autographa pulchrina|MM09480|658[0n]bp|Finland.Aland Islands|BOLD:AAA3990

Autographa pulchrina|MM04315|658[0n]bp|Finland.Finland Proper|BOLD:AAA3990
Autographa pulchrina|TLMF Lep 00295|658[0n]bp|Austria.Vorarlberg|BOLD:AAA3990
Autographa pulchrina|MM06685|658[0n]bp|Finland.Aland Islands|BOLD:AAA3990
Autographa pulchrina|MM09480|658[0n]bp|Finland.Aland Islands|BOLD:AAA3990
Autographa pulchrina|TLMF Lep 00744|658[0n]bp|Austria.Vorarlberg|BOLD:AAA3990
Syngrapha interrogationis|MM07253|658[0n]bp|Finland|BOLD:AAB3481
Syngrapha interrogationis|TLMF Lep 00297|632[0n]bp|Austria.Vorarlberg|BOLD:AAB3481
Syngrapha interrogationis|TLMF Lep 08499|658[0n]bp|Austria.Vorarlberg|BOLD:AAB3481
Syngrapha interrogationis|MM04885|658[0n]bp|Finland.Finland Proper|BOLD:AAB3481
Syngrapha interrogationis|MM02752|658[0n]bp|Finland.Northern Ostrobothnia|BOLD:AAB3481
Syngrapha interrogationis|TLMF Lep 01138|658[0n]bp|Austria.Vorarlberg|BOLD:AAB3481
Craniophora ligustri|MM06745|670[5n]bp|Finland.Aland Islands|BOLD:AAB6108
Craniophora ligustri|MM06802|658[0n]bp|Finland.Aland Islands|BOLD:AAB6108
Craniophora ligustri|MM09713|658[0n]bp|Finland.Aland Islands|BOLD:AAB6108
Craniophora ligustri|MM00137|658[0n]bp|Finland|BOLD:AAB6108
Craniophora ligustri|TLMF Lep 07838|658[0n]bp|Austria.Vorarlberg|BOLD:AAB6108
Trachea atriplicis|TLMF Lep 08281|658[0n]bp|Austria.Vorarlberg|BOLD:AAE0836
Trachea atriplicis|MM00344|658[0n]bp|Finland|BOLD:AAE0836
Trachea atriplicis|MM00855|658[0n]bp|Finland.Aland Islands|BOLD:AAE0836
Trachea atriplicis|TLMF Lep 08508|658[0n]bp|Austria.Vorarlberg|BOLD:AAE0836
Trachea atriplicis|MM01161|630[0n]bp|Finland.South Karelia|BOLD:AAE0836
Lygephila craccae|MM18522|658[0n]bp|Finland|BOLD:AAD9537
Lygephila craccae|MM12512|658[0n]bp|Finland|BOLD:AAD9537
Lygephila craccae|MM12561|658[0n]bp|Finland|BOLD:AAD9537
Lygephila craccae|TLMF Lep 08512|658[0n]bp|Austria.Vorarlberg|BOLD:AAD9538
Lygephila viciae|MM17460|658[0n]bp|Finland|BOLD:AAK6149
Lygephila viciae|MM18665|658[0n]bp|Finland|BOLD:AAK6149
Lygephila viciae|TLMF Lep 08113|658[0n]bp|Austria.Vorarlberg|BOLD:AAK6149
Lygephila viciae|MM15931|658[0n]bp|Finland|BOLD:AAK6149
Lygephila viciae|MM17459|658[0n]bp|Finland|BOLD:AAK6149
Lygephila viciae|TLMF Lep 04650|658[0n]bp|Austria.Vorarlberg|BOLD:AAK6149
Lygephila viciae|TLMF Lep 06189|658[0n]bp|Austria.Vorarlberg|BOLD:AAK6149
Phytometra viridaria|TLMF Lep 00300|658[0n]bp|Austria.Vorarlberg|BOLD:AAD4078
Phytometra viridaria|MM18718|658[0n]bp|Finland.Aland Islands|BOLD:AAD4078
Phytometra viridaria|MM18719|658[0n]bp|Finland.Aland Islands|BOLD:AAD4078
Phytometra viridaria|MM21078|658[0n]bp|Finland|BOLD:AAD4078
Laspeyria flexula|MM05088|658[0n]bp|Finland.Finland Proper|BOLD:AAC1014
Laspeyria flexula|TLMF Lep 07888|658[0n]bp|Austria.Vorarlberg|BOLD:AAC1014
Laspeyria flexula|MM01415|658[0n]bp|Finland.South Karelia|BOLD:AAC1014
Laspeyria flexula|MM01416|658[0n]bp|Finland.South Karelia|BOLD:AAC1014
Trisateles emortualis|MM04877|670[1n]bp|Finland.Finland Proper|BOLD:AAC2587
Trisateles emortualis|MM01513|658[0n]bp|Finland.South Karelia|BOLD:AAC2587
Trisateles emortualis|TLMF Lep 07978|658[0n]bp|Austria.Vorarlberg|BOLD:AAC2587
Trisateles emortualis|MM01419|658[0n]bp|Finland.South Karelia|BOLD:AAC2587
Trisateles emortualis|MM01514|619[0n]bp|Finland.South Karelia|BOLD:AAC2587
Cucullia asteris|TLMF Lep 06109|658[0n]bp|Austria.Vorarlberg|BOLD:ACF3254
Cucullia asteris|MM18536|658[0n]bp|Finland|BOLD:ACF3254
Cucullia asteris|MM09220|658[0n]bp|Finland|BOLD:ACF3254
Cucullia asteris|MM18537|640[1n]bp|Finland|BOLD:ACF3254
Cucullia lucifuga|MM19915|658[0n]bp|Finland.Uusimaa|BOLD:ABY6309
Cucullia lucifuga|MM17486|658[0n]bp|Finland|BOLD:ABY6309
Cucullia lucifuga|MM18723|658[0n]bp|Finland|BOLD:ABY6309
Cucullia lucifuga|MM17487|658[0n]bp|Finland|BOLD:ABY6309
Cucullia lucifuga|TLMF Lep 04626|658[0n]bp|Austria.Vorarlberg|BOLD:ABY6309
Cucullia umbratica|MM14097|658[0n]bp|Finland|BOLD:AAC0849
Cucullia umbratica|MM06742|658[0n]bp|Finland.Aland Islands|BOLD:AAC0849
Cucullia umbratica|MM04543|670[2n]bp|Finland.Finland Proper|BOLD:AAC0849
Cucullia umbratica|MM04914|658[0n]bp|Finland.Finland Proper|BOLD:AAC0849
Cucullia umbratica|TLMF Lep 08120|658[0n]bp|Austria.Vorarlberg|BOLD:AAC0849
Brachionycha nubeculosa|MM01614|658[0n]bp|Finland.South Karelia|BOLD:AAE0860
Brachionycha nubeculosa|MM01543|658[0n]bp|Finland.South Karelia|BOLD:AAE0860
Brachionycha nubeculosa|MM02767|658[0n]bp|Finland.Northern Ostrobothnia|BOLD:AAE0860
Brachionycha nubeculosa|MM01542|670[0n]bp|Finland.South Karelia|BOLD:AAE0860
Brachionycha nubeculosa|TLMF Lep 09769|658[0n]bp|Austria.Vorarlberg|BOLD:AAE0860
Euclidia glyphica|MM03193|658[0n]bp|Finland.Northern Ostrobothnia|BOLD:AAD0810
Euclidia glyphica|MM14858|658[0n]bp|Finland|BOLD:AAD0810
Euclidia glyphica|TLMF Lep 08115|658[0n]bp|Austria.Vorarlberg|BOLD:AAD0810
Euclidia glyphica|MM02785|658[0n]bp|Finland.Northern Ostrobothnia|BOLD:AAD0810
Pyrrhia umbra|MM05115|658[0n]bp|Finland.Finland Proper|BOLD:AAC7527
Pyrrhia umbra|MM05114|670[0n]bp|Finland.Finland Proper|BOLD:AAC7527
Pyrrhia umbra|MM01125|658[0n]bp|Finland.South Karelia|BOLD:AAC7527
Pyrrhia umbra|TLMF Lep 04661|658[0n]bp|Austria.Vorarlberg|BOLD:AAC7527
Pyrrhia umbra|MM01126|658[0n]bp|Finland.South Karelia|BOLD:AAC7527
Moma alpium|MM03763|658[0n]bp|Finland.Aland Islands|BOLD:AAD7323
Moma alpium|TLMF Lep 07834|658[0n]bp|Austria.Vorarlberg|BOLD:AAD7323
Moma alpium|MM01536|642[0n]bp|Finland.South Karelia|BOLD:AAD7323
Moma alpium|MM01537|639[0n]bp|Finland.South Karelia|BOLD:AAD7323
Deltote pygarga|MM04676|658[0n]bp|Finland.Finland Proper|BOLD:AAB9793
Deltote pygarga|MM01427|650[0n]bp|Finland.South Karelia|BOLD:AAB9793
Deltote pygarga|MM01426|646[0n]bp|Finland.South Karelia|BOLD:AAB9793
Deltote pygarga|TLMF Lep 07861|658[0n]bp|Austria.Vorarlberg|BOLD:AAB9793
Deltote pygarga|MM01512|658[0n]bp|Finland.South Karelia|BOLD:AAB9793
Deltote bankiana|TLMF Lep 05625|658[0n]bp|Austria.Vorarlberg|BOLD:AAD6191
Deltote bankiana|MM18721|658[0n]bp|Finland|BOLD:AAD6191
Deltote bankiana|MM18720|658[0n]bp|Finland|BOLD:AAD6191
Deltote bankiana|MM01751|658[0n]bp|Finland.South Karelia|BOLD:AAD6191
Deltote uncula|TLMF Lep 10003|658[0n]bp|Austria.Vorarlberg|BOLD:AAF4745
Deltote uncula|MM02925|658[0n]bp|Finland.Northern Ostrobothnia|BOLD:AAF4745
Deltote uncula|MM04601|670[4n]bp|Finland.Finland Proper|BOLD:AAF4745
Deltote uncula|MM18523|658[1n]bp|Finland|BOLD:AAF4745
Colocasia coryli|MM08417|658[0n]bp|Finland|BOLD:AAC2611
Colocasia coryli|MM01523|658[0n]bp|Finland.South Karelia|BOLD:AAC2611
Colocasia coryli|MM01524|658[0n]bp|Finland.South Karelia|BOLD:AAC2611
Colocasia coryli|TLMF Lep 04638|658[0n]bp|Austria.Vorarlberg|BOLD:AAC2611
Panthea coenobita|MM04583|615[0n]bp|Finland.Finland Proper|BOLD:AAE7030
Panthea coenobita|MM18528|658[0n]bp|Finland|BOLD:AAE7030
Panthea coenobita|TLMF Lep 06190|658[0n]bp|Austria.Vorarlberg|BOLD:AAE7030
Panthea coenobita|TLMF Lep 08126|658[0n]bp|Austria.Vorarlberg|BOLD:AAE7030
Panthea coenobita|MM05913|658[0n]bp|Finland|BOLD:AAE7030
Amphipyra perflua|MM04353|658[0n]bp|Finland.Finland Proper|BOLD:AAD2299
Amphipyra perflua|TLMF Lep 08504|658[0n]bp|Austria.Vorarlberg|BOLD:AAD2299
Amphipyra perflua|MM01162|665[0n]bp|Finland.South Karelia|BOLD:AAD2299
Amphipyra perflua|MM01163|638[0n]bp|Finland.South Karelia|BOLD:AAD2299

Amphipyra perflua|TLMF Lep 08504|658[0n]bp|Austria.Vorarlberg|BOLD:AAD2299
Amphipyra perflua|MM01162|665[0n]bp|Finland.South Karelia|BOLD:AAD2299
Amphipyra perflua|MM01163|638[0n]bp|Finland.South Karelia|BOLD:AAD2299
Amphipyra perflua|MM01548|639[0n]bp|Finland.South Karelia|BOLD:AAD2299
Amphipyra perflua|MM12544|658[0n]bp|Finland|BOLD:AAD2299
Amphipyra berbera|MM04850|658[0n]bp|Finland.Finland Proper|BOLD:AAB4108
Amphipyra berbera|MM14418|658[0n]bp|Finland|BOLD:AAB4108
Amphipyra berbera|MM04849|658[0n]bp|Finland.Finland Proper|BOLD:AAB4108
Amphipyra berbera|TLMF Lep 07580|658[0n]bp|Austria.Vorarlberg|BOLD:AAB4108
Amphipyra pyramidea|MM04847|658[0n]bp|Finland.Finland Proper|BOLD:AAB4107
Amphipyra pyramidea|MM18532|658[0n]bp|Finland.Finland Proper|BOLD:AAB4107
Amphipyra pyramidea|MM14419|658[0n]bp|Finland|BOLD:AAB4107
Amphipyra pyramidea|TLMF Lep 00293|658[0n]bp|Austria.Vorarlberg|BOLD:AAB4107
Amphipyra tragopoginis|TLMF Lep 08550|658[0n]bp|Austria.Vorarlberg|BOLD:AAB3277
Amphipyra tragopoginis|MM01730|658[0n]bp|Finland.South Karelia|BOLD:AAB3277
Amphipyra tragopoginis|MM01731|658[0n]bp|Finland.South Karelia|BOLD:AAB3277
Amphipyra tragopoginis|MM04766|658[0n]bp|Finland.Finland Proper|BOLD:AAB3277
Diloba caeruleocephala|TLMF Lep 08789|658[0n]bp|Austria.Vorarlberg|BOLD:AAE8447
Diloba caeruleocephala|MM15926|658[0n]bp|Finland|BOLD:AAE8447
Diloba caeruleocephala|MM21049|658[0n]bp|Finland|BOLD:AAE8447
Diloba caeruleocephala|MM21050|658[0n]bp|Finland|BOLD:AAE8447
Tholera cespitis|MM01741|633[0n]bp|Finland.South Karelia|BOLD:ACE5542
Tholera cespitis|TLMF Lep 12547|658[0n]bp|Austria.Vorarlberg|BOLD:AAD2239
Tholera cespitis|MM07351|658[0n]bp|Finland|BOLD:AAD2239
Tholera cespitis|MM04754|658[0n]bp|Finland.Finland Proper|BOLD:AAD2239
Cerapteryx graminis|MM00812|636[0n]bp|Finland.Northern Ostrobothnia|BOLD:AAB4284
Cerapteryx graminis|MM01612|658[0n]bp|Finland.South Karelia|BOLD:AAB4284
Cerapteryx graminis|MM08079|658[0n]bp|Finland|BOLD:AAB4284
Cerapteryx graminis|MM01613|637[0n]bp|Finland.South Karelia|BOLD:AAB4284
Cerapteryx graminis|TLMF Lep 04617|634[0n]bp|Austria.Vorarlberg|BOLD:AAB4284
Tholera decimalis|TLMF Lep 08758|658[0n]bp|Austria.Vorarlberg|BOLD:AAC9682
Tholera decimalis|MM01729|658[1n]bp|Finland.South Karelia|BOLD:AAC9682
Tholera decimalis|MM01728|658[1n]bp|Finland.South Karelia|BOLD:AAC9682
Tholera decimalis|MM04795|658[0n]bp|Finland.Finland Proper|BOLD:AAC9682
Leucania comma|TLMF Lep 09226|629[0n]bp|Austria.Vorarlberg|BOLD:AAA3150
Leucania comma|MM09493|658[0n]bp|Finland.Aland Islands|BOLD:AAA3150
Leucania comma|MM01602|639[0n]bp|Finland.South Karelia|BOLD:AAA3150
Leucania comma|MM01601|643[0n]bp|Finland.South Karelia|BOLD:AAA3150
Leucania obsoleta|MM00662|658[0n]bp|Finland|BOLD:AAD5689
Leucania obsoleta|TLMF Lep 04452|638[0n]bp|Austria.Vorarlberg|BOLD:AAD5689
Leucania obsoleta|MM04572|648[0n]bp|Finland.Finland Proper|BOLD:AAD5689
Leucania obsoleta|MM11514|520[0n]bp|Finland|BOLD:AAD5689
Mythimna ferrago|TLMF Lep 08160|658[0n]bp|Austria.Vorarlberg|BOLD:AAC2354
Mythimna ferrago|TLMF Lep 06127|658[0n]bp|Austria.Vorarlberg|BOLD:AAC2354
Mythimna ferrago|MM21045|658[0n]bp|Finland|BOLD:AAC2354
Mythimna ferrago|MM07263|658[0n]bp|Finland|BOLD:AAC2354
Mythimna ferrago|MM01630|643[0n]bp|Finland.South Karelia|BOLD:AAC2354
Mythimna ferrago|MM12241|658[0n]bp|Finland|BOLD:AAC2354
Mythimna ferrago|MM01631|658[0n]bp|Finland.South Karelia|BOLD:AAC2354
Mythimna ferrago|MM10961|658[0n]bp|Finland|BOLD:AAC2355
Mythimna ferrago|MM12661|658[0n]bp|Finland|BOLD:AAC2355
Mythimna l-album|MM15955|658[0n]bp|Finland|BOLD:AAD5882
Mythimna l-album|TLMF Lep 08111|658[0n]bp|Austria.Vorarlberg|BOLD:AAD5882
Mythimna conigera|MM01607|658[0n]bp|Finland.South Karelia|BOLD:AAC6915
Mythimna conigera|MM01608|642[0n]bp|Finland.South Karelia|BOLD:AAC6915
Mythimna conigera|TLMF Lep 08108|658[0n]bp|Austria.Vorarlberg|BOLD:AAC6915
Mythimna conigera|MM04874|658[0n]bp|Finland.Finland Proper|BOLD:AAC6915
Mythimna impura|MM01606|658[0n]bp|Finland.South Karelia|BOLD:AAB9151
Mythimna impura|MM01605|658[0n]bp|Finland.South Karelia|BOLD:AAB9151
Mythimna impura|TLMF Lep 05614|658[0n]bp|Austria.Vorarlberg|BOLD:AAB9151
Mythimna impura|MM05124|658[0n]bp|Finland.Finland Proper|BOLD:AAB9151
Mythimna straminea|MM05155|658[0n]bp|Finland.Finland Proper|BOLD:AAC9826
Mythimna straminea|MM10923|658[0n]bp|Finland|BOLD:AAC9826
Mythimna straminea|MM17380|658[0n]bp|Finland|BOLD:AAC9826
Mythimna straminea|TLMF Lep 08185|658[0n]bp|Austria.Vorarlberg|BOLD:AAC9826
Mythimna pudorina|TLMF Lep 05626|658[0n]bp|Austria.Vorarlberg|BOLD:AAD6230
Mythimna pudorina|MM10930|658[0n]bp|Finland|BOLD:AAD6230
Mythimna pudorina|MM18740|658[0n]bp|Finland|BOLD:AAD6230
Mythimna pudorina|MM18016|658[0n]bp|Finland|BOLD:AAD6230
Mythimna turca|MM17514|658[0n]bp|Finland|BOLD:AAF8311
Mythimna turca|TLMF Lep 08145|658[0n]bp|Austria.Vorarlberg|BOLD:AAF8311
Mythimna turca|MM18907|658[0n]bp|Finland|BOLD:AAF8311
Mythimna turca|MM11083|658[2n]bp|Finland|BOLD:AAF8311
Orthosia cerasi|TLMF Lep 07871|658[0n]bp|Austria.Vorarlberg|BOLD:AAC3426
Orthosia cerasi|MM04612|658[0n]bp|Finland.Finland Proper|BOLD:AAC3426
Orthosia cerasi|MM12106|658[0n]bp|Finland|BOLD:AAC3426
Orthosia cerasi|MM04613|658[0n]bp|Finland.Finland Proper|BOLD:AAC3426
Orthosia cruda|TLMF Lep 08808|658[0n]bp|Austria.Vorarlberg|BOLD:AAD9024
Orthosia cruda|MM04616|658[0n]bp|Finland.Finland Proper|BOLD:AAD9024
Orthosia cruda|MM04617|630[0n]bp|Finland.Finland Proper|BOLD:AAD9024
Orthosia cruda|MM04618|658[0n]bp|Finland.Finland Proper|BOLD:AAD9024
Orthosia incerta|BIOUG04118-A04|633[0n]bp|Finland.Northern Ostrobothnia|BOLD:ABY5277
Orthosia incerta|MM01563|658[0n]bp|Finland.South Karelia|BOLD:ABY5277
Orthosia incerta|MM02743|658[0n]bp|Finland.Northern Ostrobothnia|BOLD:ABY5277
Orthosia incerta|TLMF Lep 08856|658[0n]bp|Austria.Vorarlberg|BOLD:ABY5277
Orthosia incerta|MM01564|644[0n]bp|Finland.South Karelia|BOLD:ABY5277
Orthosia gracilis|MM01566|647[0n]bp|Finland.South Karelia|BOLD:AAC2300
Orthosia gracilis|MM01567|658[0n]bp|Finland.South Karelia|BOLD:AAC2300
Orthosia gracilis|MM04504|658[0n]bp|Finland.Finland Proper|BOLD:AAC2300
Orthosia gracilis|TLMF Lep 08809|658[0n]bp|Austria.Vorarlberg|BOLD:AAC2300
Orthosia opima|TLMF Lep 08853|658[0n]bp|Austria.Vorarlberg|BOLD:ACE8591
Orthosia opima|MM01562|642[0n]bp|Finland.South Karelia|BOLD:ACE8591
Orthosia opima|MM00415|658[0n]bp|Finland.Northern Ostrobothnia|BOLD:ACE8591
Orthosia opima|MM01561|658[0n]bp|Finland.South Karelia|BOLD:ACE8591
Anorthoa munda|MM04339|658[0n]bp|Finland.Finland Proper|BOLD:AAD6193
Anorthoa munda|MM04340|658[0n]bp|Finland.Finland Proper|BOLD:AAD6193
Anorthoa munda|TLMF Lep 08854|658[0n]bp|Austria.Vorarlberg|BOLD:AAD6193
Anorthoa munda|MM04341|658[0n]bp|Finland.Finland Proper|BOLD:AAD6193
Orthosia gothica|MM01574|642[3n]bp|Finland.South Karelia|BOLD:AAB6211
Orthosia gothica|MM08419|658[0n]bp|Finland|BOLD:AAB6211
Orthosia gothica|TLMF Lep 08852|658[0n]bp|Austria.Vorarlberg|BOLD:AAB6211
Orthosia gothica|MM01573|658[0n]bp|Finland.South Karelia|BOLD:AAB6211
Orthosia gothica|MM02733|658[0n]bp|Finland.Northern Ostrobothnia|BOLD:AAB6211

Orthosia gothica|TLMF Lep 08852|658[0n]bp|Austria.Vorarlberg|BOLD:AAB6211
Orthosia gothica|MM01573|658[0n]bp|Finland.South Karelia|BOLD:AAB6211
Orthosia gothica|MM02733|658[0n]bp|Finland.Northern Ostrobothnia|BOLD:AAB6211
Orthosia populeti|MM01570|658[0n]bp|Finland.South Karelia|BOLD:AAD3183
Orthosia populeti|MM01569|658[0n]bp|Finland.South Karelia|BOLD:AAD3183
Orthosia populeti|TLMF Lep 08855|658[0n]bp|Austria.Vorarlberg|BOLD:AAD3183
Orthosia populeti|MM00453|658[0n]bp|Finland.South Karelia|BOLD:AAD3183
Orthosia populeti|MM13860|658[0n]bp|Finland.Lapland|BOLD:AAD3183
Panolis flammea|MM00397|658[0n]bp|Finland.Northern Ostrobothnia|BOLD:AAC5406
Panolis flammea|MM01583|638[0n]bp|Finland.South Karelia|BOLD:AAC5406
Panolis flammea|MM01582|642[0n]bp|Finland.South Karelia|BOLD:AAC5406
Panolis flammea|TLMF Lep 07881|658[0n]bp|Austria.Vorarlberg|BOLD:AAC5406
Pachetra sagittigera|TLMF Lep 07832|658[0n]bp|Austria.Vorarlberg|BOLD:AAC1169
Pachetra sagittigera|MM18909|658[0n]bp|Finland.Aland Islands|BOLD:ACF4123
Pachetra sagittigera|MM15957|658[1n]bp|Finland|BOLD:ACF4123
Polia bombycina|MM12540|658[0n]bp|Finland|BOLD:AAD1839
Polia bombycina|MM01750|633[1n]bp|Finland.South Karelia|BOLD:AAD1839
Polia bombycina|TLMF Lep 09227|658[0n]bp|Austria.Vorarlberg|BOLD:AAD1839
Polia bombycina|MM12542|658[0n]bp|Finland|BOLD:AAD1839
Polia bombycina|MM05120|658[0n]bp|Finland.Finland Proper|BOLD:AAD1839
Polia hepatica|TLMF Lep 09999|658[0n]bp|Austria.Vorarlberg|BOLD:AAE1370
Polia hepatica|MM17917|658[0n]bp|Finland|BOLD:AAE1370
Polia hepatica|MM17918|658[0n]bp|Finland|BOLD:AAE1370
Polia hepatica|MM17916|639[0n]bp|Finland|BOLD:AAE1370
Polia nebulosa|MM10808|658[0n]bp|Finland|BOLD:AAE1369
Polia nebulosa|MM01749|632[1n]bp|Finland.South Karelia|BOLD:AAE1369
Polia nebulosa|TLMF Lep 08106|658[0n]bp|Austria.Vorarlberg|BOLD:AAE1369
Polia nebulosa|MM12549|658[0n]bp|Finland|BOLD:AAE1369
Polia nebulosa|MM05090|556[0n]bp|Finland.Finland Proper|BOLD:AAE1369
Polia nebulosa|TLMF Lep 08174|658[0n]bp|Austria.Vorarlberg|BOLD:AAE1369
Polia nebulosa|TLMF Lep 08173|658[0n]bp|Austria.Vorarlberg|BOLD:AAE1369
Charanyca ferruginea|MM05144|658[0n]bp|Finland.Finland Proper|BOLD:AAB9497
Charanyca ferruginea|TLMF Lep 06160|658[0n]bp|Austria.Vorarlberg|BOLD:AAB9497
Charanyca ferruginea|MM01538|658[0n]bp|Finland.South Karelia|BOLD:AAB9497
Charanyca ferruginea|MM01539|658[0n]bp|Finland.South Karelia|BOLD:AAB9497
Charanyca ferruginea|TLMF Lep 08138|658[0n]bp|Austria.Vorarlberg|BOLD:AAB9497
Euxoa nigricans|MM04904|658[0n]bp|Finland.Finland Proper|BOLD:ABZ9438
Euxoa nigricans|MM18655|658[0n]bp|Finland|BOLD:ABZ9438
Euxoa nigricans|TLMF Lep 06188|658[0n]bp|Austria.Vorarlberg|BOLD:ABZ9438
Euxoa nigricans|MM14694|658[0n]bp|Finland|BOLD:ABZ9438
Euxoa nigricans|MM14690|658[0n]bp|Finland|BOLD:ABZ9438
Euxoa nigricans|MM04375|658[0n]bp|Finland.Finland Proper|BOLD:ABZ9438
Euxoa nigricans|MM18674|658[0n]bp|Finland|BOLD:ABZ9438
Euxoa nigricans|MM06955|658[0n]bp|Finland|BOLD:ABZ9438
Euxoa nigricans|MM14691|658[0n]bp|Finland|BOLD:ABZ9438
Euxoa nigricans|MM03617|658[0n]bp|Finland.Uusimaa|BOLD:ABZ9438
Euxoa nigricans|MM06979|658[0n]bp|Finland|BOLD:ABZ9438
Euxoa recussa|MM00731|658[0n]bp|Finland|BOLD:ACE9579
Euxoa recussa|MM00730|657[0n]bp|Finland|BOLD:ACE9579
Euxoa recussa|MM18001|658[0n]bp|Finland|BOLD:ACE9579
Euxoa recussa|TLMF Lep 06097|658[0n]bp|Austria.Vorarlberg|BOLD:ACE9579
Charanyca trigrammica|TLMF Lep 07913|658[0n]bp|Austria.Vorarlberg|BOLD:AAB4764
Charanyca trigrammica|MM14288|658[0n]bp|Finland.Aland Islands|BOLD:AAB4764
Charanyca trigrammica|MM12562|658[0n]bp|Finland|BOLD:AAB4764
Charanyca trigrammica|MM14315|658[0n]bp|Finland.Aland Islands|BOLD:AAB4764
Hoplodrina blanda|MM17365|658[0n]bp|Finland|BOLD:AAC0362
Hoplodrina blanda|TLMF Lep 08510|658[0n]bp|Austria.Vorarlberg|BOLD:AAC0362
Hoplodrina blanda|MM05130|658[0n]bp|Finland.Finland Proper|BOLD:AAC0362
Hoplodrina blanda|MM18046|658[0n]bp|Finland|BOLD:AAC0362
Hoplodrina blanda|MM18045|658[0n]bp|Finland|BOLD:AAC0362
Hoplodrina octogenaria|TLMF Lep 05615|658[0n]bp|Austria.Vorarlberg|BOLD:AAB4763
Hoplodrina octogenaria|MM01651|670[0n]bp|Finland.South Karelia|BOLD:AAB4763
Hoplodrina octogenaria|MM07463|658[0n]bp|Finland|BOLD:AAB4763
Hoplodrina octogenaria|MM17366|658[0n]bp|Finland|BOLD:AAB4763
Hoplodrina octogenaria|MM17367|658[0n]bp|Finland|BOLD:AAB4763
Hoplodrina octogenaria|MM18047|658[0n]bp|Finland|BOLD:AAB4763
Hoplodrina octogenaria|TLMF Lep 08140|658[0n]bp|Austria.Vorarlberg|BOLD:AAB4763
Hoplodrina octogenaria|MM12720|658[0n]bp|Finland|BOLD:AAB4763
Hoplodrina octogenaria|MM01746|637[1n]bp|Finland.South Karelia|BOLD:AAB4763
Hoplodrina octogenaria|MM04900|624[0n]bp|Finland.Finland Proper|BOLD:AAB4763
Hoplodrina octogenaria|MM01652|639[0n]bp|Finland.South Karelia|BOLD:AAB4763
Athetis pallustris|MM01522|658[0n]bp|Finland.South Karelia|BOLD:AAF5119
Athetis pallustris|MM01521|658[0n]bp|Finland.South Karelia|BOLD:AAF5119
Athetis pallustris|TLMF Lep 04676|658[0n]bp|Austria.Vorarlberg|BOLD:AAF5119
Athetis pallustris|MM02778|658[0n]bp|Finland.Northern Ostrobothnia|BOLD:AAF5119
Lasionycta imbecilla|MM01610|658[0n]bp|Finland.South Karelia|BOLD:AAE1902
Lasionycta imbecilla|MM06604|658[0n]bp|Finland|BOLD:AAE1902
Lasionycta imbecilla|MM01609|658[0n]bp|Finland.South Karelia|BOLD:AAE1902
Lasionycta imbecilla|TLMF Lep 06108|658[0n]bp|Austria.Vorarlberg|BOLD:AAE1902
Lasionycta imbecilla|TLMF Lep 08547|658[0n]bp|Austria.Vorarlberg|BOLD:AAE1902
Lasionycta imbecilla|TLMF Lep 04675|658[0n]bp|Austria.Vorarlberg|BOLD:AAE1902
Lasionycta proxima|MM15891|658[0n]bp|Finland|BOLD:AAD3486
Lasionycta proxima|MM09917|636[1n]bp|Finland|BOLD:AAD3486
Lasionycta proxima|TLMF Lep 08777|658[0n]bp|Austria.Vorarlberg|BOLD:AAD3486
Lasionycta proxima|MM15890|658[0n]bp|Finland|BOLD:AAD3486
Hecatera bicolorata|TLMF Lep 04607|658[0n]bp|Austria.Vorarlberg|BOLD:AAD3287
Hecatera bicolorata|MM10058|654[0n]bp|Finland|BOLD:AAD3287
Hecatera bicolorata|MM02382|658[0n]bp|Finland.South Karelia|BOLD:AAD3287
Hecatera bicolorata|MM14290|658[0n]bp|Finland.Aland Islands|BOLD:AAD3287
Mamestra brassicae|TLMF Lep 08778|658[0n]bp|Austria.Vorarlberg|BOLD:AAB7858
Mamestra brassicae|MM04911|658[0n]bp|Finland.Finland Proper|BOLD:AAB7858
Mamestra brassicae|MM04910|658[0n]bp|Finland.Finland Proper|BOLD:AAB7858
Mamestra brassicae|TLMF Lep 07904|658[0n]bp|Austria.Vorarlberg|BOLD:AAB7858
Mamestra brassicae|MM12599|658[0n]bp|Finland|BOLD:AAB7858
Lacanobia contigua|MM04584|658[0n]bp|Finland.Finland Proper|BOLD:AAD8512
Lacanobia contigua|TLMF Lep 08147|658[0n]bp|Austria.Vorarlberg|BOLD:AAD8512
Lacanobia contigua|TLMF Lep 04627|658[0n]bp|Austria.Vorarlberg|BOLD:AAD8512
Lacanobia contigua|MM01633|639[0n]bp|Finland.South Karelia|BOLD:AAD8512
Lacanobia contigua|MM01632|633[0n]bp|Finland.South Karelia|BOLD:AAD8512
Lacanobia contigua|MM18736|658[0n]bp|Finland|BOLD:AAD8512
Lacanobia oleracea|TLMF Lep 08182|658[0n]bp|Austria.Vorarlberg|BOLD:ABY4614
Lacanobia oleracea|MM21086|658[1n]bp|Finland|BOLD:ABY4614
Lacanobia oleracea|MM01618|658[0n]bp|Finland.South Karelia|BOLD:ABY4614

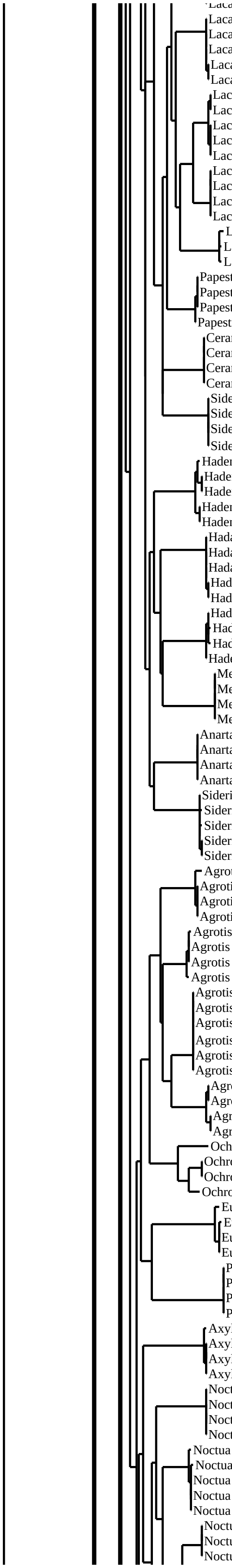

Lacanobia contigua|MM18730|658[0n]bp|Finland|BOLD:AAD8512
Lacanobia oleracea|TLMF Lep 08182|658[0n]bp|Austria.Vorarlberg|BOLD:ABY4614
Lacanobia oleracea|MM21086|658[1n]bp|Finland|BOLD:ABY4614
Lacanobia oleracea|MM01618|658[0n]bp|Finland.South Karelia|BOLD:ABY4614
Lacanobia oleracea|MM01617|658[0n]bp|Finland.South Karelia|BOLD:ABY4614
Lacanobia oleracea|MM05111|635[0n]bp|Finland.Finland Proper|BOLD:ABY4614
Lacanobia suasa|TLMF Lep 07880|658[0n]bp|Austria.Vorarlberg|BOLD:ACF5197
Lacanobia suasa|MM12654|658[0n]bp|Finland|BOLD:ACF5197
Lacanobia suasa|MM17285|658[0n]bp|Finland|BOLD:ACF5197
Lacanobia suasa|MM05146|639[0n]bp|Finland.Finland Proper|BOLD:ACF5197
Lacanobia suasa|MM05145|655[0n]bp|Finland.Finland Proper|BOLD:ACF5197
Lacanobia thalassina|MM01589|638[0n]bp|Finland.South Karelia|BOLD:ABY4615
Lacanobia thalassina|MM01588|658[0n]bp|Finland.South Karelia|BOLD:ABY4615
Lacanobia thalassina|MM00608|658[0n]bp|Finland.Northern Ostrobothnia|BOLD:ABY4615
Lacanobia thalassina|TLMF Lep 08144|658[0n]bp|Austria.Vorarlberg|BOLD:ABY4615
Lacanobia w-latinum|MM15953|658[0n]bp|Finland|BOLD:AAC6463
Lacanobia w-latinum|TLMF Lep 08107|658[0n]bp|Austria.Vorarlberg|BOLD:AAC6463
Lacanobia w-latinum|TLMF Lep 04609|658[0n]bp|Austria.Vorarlberg|BOLD:AAC6463
Papestra biren|MM14830|658[0n]bp|Finland|BOLD:AAA9849
Papestra biren|MM04101|632[0n]bp|Finland.Lapland|BOLD:AAA9849
Papestra biren|MM12519|658[0n]bp|Finland|BOLD:AAA9849
Papestra biren|TLMF Lep 04652|658[0n]bp|Austria.Vorarlberg|BOLD:AAA9849
Ceramica pisi|MM10885|658[0n]bp|Finland|BOLD:AAB0758
Ceramica pisi|TLMF Lep 07835|658[0n]bp|Austria.Vorarlberg|BOLD:AAB0758
Ceramica pisi|MM04552|658[0n]bp|Finland.Finland Proper|BOLD:AAB0758
Ceramica pisi|MM00826|658[0n]bp|Finland.Northern Ostrobothnia|BOLD:AAB0758
Sideridis reticulata|MM01593|632[0n]bp|Finland.South Karelia|BOLD:AAD4204
Sideridis reticulata|MM12607|658[0n]bp|Finland|BOLD:AAD4204
Sideridis reticulata|MM01592|643[0n]bp|Finland.South Karelia|BOLD:AAD4204
Sideridis reticulata|TLMF Lep 07581|658[0n]bp|Austria.Vorarlberg|BOLD:AAD4204
Hadena perplexa|MM12635|658[0n]bp|Finland|BOLD:AAC7968
Hadena perplexa|MM04737|658[0n]bp|Finland.Finland Proper|BOLD:AAC7968
Hadena perplexa|MM00658|658[0n]bp|Finland|BOLD:AAC7968
Hadena perplexa|TLMF Lep 04672|658[1n]bp|Austria.Vorarlberg|BOLD:AAC7968
Hadena perplexa|TLMF Lep 01139|658[0n]bp|Austria.Vorarlberg|BOLD:AAC7968
Hada plebeja|MM04732|621[0n]bp|Finland.Finland Proper|BOLD:AAC2435
Hada plebeja|MM01587|658[0n]bp|Finland.South Karelia|BOLD:AAC2435
Hada plebeja|MM01586|658[0n]bp|Finland.South Karelia|BOLD:AAC2435
Hada plebeja|MM04098|658[0n]bp|Finland.Lapland|BOLD:AAC2435
Hada plebeja|TLMF Lep 04663|658[0n]bp|Austria.Vorarlberg|BOLD:AAC2435
Hadena albimacula|MM18021|610[1n]bp|Finland|BOLD:AAF8086
Hadena albimacula|MM00656|658[0n]bp|Finland|BOLD:AAF8086
Hadena albimacula|TLMF Lep 04674|629[0n]bp|Austria.Vorarlberg|BOLD:AAF8086
Hadena albimacula|MM15889|658[0n]bp|Finland|BOLD:AAF8086
Melanchra persicariae|MM01597|658[0n]bp|Finland.South Karelia|BOLD:AAC7429
Melanchra persicariae|TLMF Lep 08129|658[0n]bp|Austria.Vorarlberg|BOLD:AAC7429
Melanchra persicariae|MM04556|658[0n]bp|Finland.Finland Proper|BOLD:AAC7429
Melanchra persicariae|MM01596|637[0n]bp|Finland.South Karelia|BOLD:AAC7429
Anarta myrtilli|MM18022|658[0n]bp|Finland|BOLD:AAE1452
Anarta myrtilli|MM15888|625[0n]bp|Finland|BOLD:AAE1452
Anarta myrtilli|MM15887|636[3n]bp|Finland|BOLD:AAE1452
Anarta myrtilli|TLMF Lep 09859|658[0n]bp|Austria.Vorarlberg|BOLD:AAE1452
Sideridis rivularis|MM01619|643[0n]bp|Finland.South Karelia|BOLD:AAC8418
Sideridis rivularis|MM14101|658[0n]bp|Finland|BOLD:AAC8418
Sideridis rivularis|MM04758|658[0n]bp|Finland.Finland Proper|BOLD:AAC8418
Sideridis rivularis|TLMF Lep 08186|658[0n]bp|Austria.Vorarlberg|BOLD:AAC8418
Sideridis rivularis|TLMF Lep 08807|658[0n]bp|Austria.Vorarlberg|BOLD:AAC8418
Agrotis ipsilon|MM17212|658[0n]bp|Finland|BOLD:AAA3364
Agrotis ipsilon|MM18000|658[0n]bp|Finland|BOLD:AAA3364
Agrotis ipsilon|MM04210|651[0n]bp|Finland.Aland Islands|BOLD:AAA3364
Agrotis ipsilon|TLMF Lep 07833|658[0n]bp|Austria.Vorarlberg|BOLD:AAA3364
Agrotis clavis|MM09495|658[0n]bp|Finland.Aland Islands|BOLD:AAC2793
Agrotis clavis|MM01656|658[0n]bp|Finland.South Karelia|BOLD:AAC2793
Agrotis clavis|MM04575|658[0n]bp|Finland.Finland Proper|BOLD:AAC2793
Agrotis clavis|TLMF Lep 08490|658[0n]bp|Austria.Vorarlberg|BOLD:AAC2793
Agrotis exclamationis|MM18832|658[0n]bp|Finland|BOLD:AAB9113
Agrotis exclamationis|MM01657|643[0n]bp|Finland.South Karelia|BOLD:AAB9113
Agrotis exclamationis|TLMF Lep 07903|658[0n]bp|Austria.Vorarlberg|BOLD:AAB9113
Agrotis exclamationis|MM06642|658[0n]bp|Finland|BOLD:AAB9113
Agrotis exclamationis|MM04568|658[0n]bp|Finland.Finland Proper|BOLD:AAB9113
Agrotis exclamationis|TLMF Lep 08146|658[0n]bp|Austria.Vorarlberg|BOLD:AAB9113
Agrotis segetum|MM11111|658[0n]bp|Finland|BOLD:AAC3884
Agrotis segetum|TLMF Lep 09862|658[0n]bp|Austria.Vorarlberg|BOLD:AAC3884
Agrotis segetum|MM15905|658[0n]bp|Finland|BOLD:AAC3884
Agrotis segetum|MM15904|658[0n]bp|Finland|BOLD:AAC3884
Ochropleura plecta|MM22894|658[0n]bp|Finland|BOLD:ABY0189
Ochropleura plecta|MM01585|658[0n]bp|Finland.South Karelia|BOLD:AAA3074
Ochropleura plecta|MM01584|637[0n]bp|Finland.South Karelia|BOLD:AAA3074
Ochropleura plecta|TLMF Lep 07859|658[0n]bp|Austria.Vorarlberg|BOLD:ACE7640
Euplexia lucipara|TLMF Lep 08135|658[0n]bp|Austria.Vorarlberg|BOLD:AAC2054
Euplexia lucipara|MM01540|658[0n]bp|Finland.South Karelia|BOLD:AAC2054
Euplexia lucipara|MM04733|658[0n]bp|Finland.Finland Proper|BOLD:AAC2054
Euplexia lucipara|MM01541|658[0n]bp|Finland.South Karelia|BOLD:AAC2054
Phlogophora meticulosa|TLMF Lep 08806|658[0n]bp|Austria.Vorarlberg|BOLD:AAB7358
Phlogophora meticulosa|MM17288|658[0n]bp|Finland|BOLD:AAB7358
Phlogophora meticulosa|MM14436|658[0n]bp|Finland|BOLD:AAB7358
Phlogophora meticulosa|MM14737|658[0n]bp|Finland.Aland Islands|BOLD:AAB7358
Axylia putris|TLMF Lep 07882|658[0n]bp|Austria.Vorarlberg|BOLD:AAB6626
Axylia putris|MM05142|658[0n]bp|Finland.Finland Proper|BOLD:AAB6626
Axylia putris|MM01629|643[0n]bp|Finland.South Karelia|BOLD:AAB6626
Axylia putris|MM01628|643[0n]bp|Finland.South Karelia|BOLD:AAB6626
Noctua comes|TLMF Lep 08761|658[0n]bp|Austria.Vorarlberg|BOLD:AAA2633
Noctua comes|MM11092|658[0n]bp|Finland.Aland Islands|BOLD:AAA2633
Noctua comes|MM12550|658[0n]bp|Finland|BOLD:AAA2633
Noctua comes|MM12551|658[0n]bp|Finland|BOLD:AAA2633
Noctua fimbriata|TLMF Lep 08553|658[0n]bp|Austria.Vorarlberg|BOLD:AAA6454
Noctua fimbriata|MM04752|670[4n]bp|Finland.Finland Proper|BOLD:AAA6454
Noctua fimbriata|MM12578|658[0n]bp|Finland|BOLD:AAA6454
Noctua fimbriata|MM10816|658[0n]bp|Finland|BOLD:AAA6454
Noctua fimbriata|MM04753|658[0n]bp|Finland.Finland Proper|BOLD:AAA6454
Noctua janthe|TLMF Lep 07722|658[0n]bp|Austria|BOLD:AAA7574
Noctua janthe|MM19920|658[0n]bp|Finland.Finland Proper|BOLD:AAA7574
Noctua janthe|TLMF Lep 07723|658[0n]bp|Austria|BOLD:AAA7574

Noctua janthe|TLMF Lep 07722|658[0n]bp|Austria|BOLD:AAA7574
Noctua janthe|MM19920|658[0n]bp|Finland.Finland Proper|BOLD:AAA7574
Noctua janthe|TLMF Lep 07723|658[0n]bp|Austria|BOLD:AAA7574
Noctua janthina|TLMF Lep 00292|658[1n]bp|Austria.Vorarlberg|BOLD:ABZ6181
Noctua janthina|MM17492|658[0n]bp|Finland|BOLD:ABZ6181
Noctua janthina|MM18742|658[0n]bp|Finland|BOLD:ABZ6181
Noctua janthina|MM15961|658[0n]bp|Finland|BOLD:ABZ6181
Noctua janthina|MM19919|658[0n]bp|Finland.Finland Proper|BOLD:ABZ6181
Noctua pronuba|MM01686|658[0n]bp|Finland.South Karelia|BOLD:AAA2632
Noctua pronuba|MM04912|658[0n]bp|Finland.Finland Proper|BOLD:AAA2632
Noctua pronuba|MM01685|658[0n]bp|Finland.South Karelia|BOLD:AAA2632
Noctua pronuba|TLMF Lep 08154|658[0n]bp|Austria.Vorarlberg|BOLD:AAA2632
Chersotis cuprea|MM01721|630[0n]bp|Finland.South Karelia|BOLD:AAD0415
Chersotis cuprea|TLMF Lep 04616|658[0n]bp|Austria.Vorarlberg|BOLD:AAD0415
Chersotis cuprea|MM01720|658[0n]bp|Finland.South Karelia|BOLD:AAD0415
Chersotis cuprea|MM02759|658[0n]bp|Finland.Northern Ostrobothnia|BOLD:AAD0415
Naenia typica|MM09740|658[0n]bp|Finland.Aland Islands|BOLD:AAC9760
Naenia typica|MM04557|658[0n]bp|Finland.Finland Proper|BOLD:AAC9760
Naenia typica|MM04897|658[0n]bp|Finland.Finland Proper|BOLD:AAC9760
Naenia typica|TLMF Lep 08184|658[0n]bp|Austria.Vorarlberg|BOLD:AAC9760
Diarsia brunnea|MM01654|637[0n]bp|Finland.South Karelia|BOLD:AAD6686
Diarsia brunnea|MM02773|615[0n]bp|Finland.Northern Ostrobothnia|BOLD:AAD6686
Diarsia brunnea|TLMF Lep 08142|658[0n]bp|Austria.Vorarlberg|BOLD:AAD6686
Diarsia brunnea|MM01653|658[0n]bp|Finland.South Karelia|BOLD:AAD6686
Diarsia brunnea|MM18010|658[0n]bp|Finland|BOLD:AAD6686
Diarsia mendica|MM05112|658[0n]bp|Finland|BOLD:ABZ6600
Diarsia mendica|TLMF Lep 06107|658[0n]bp|Austria.Vorarlberg|BOLD:ABZ6600
Diarsia mendica|MM10960|633[0n]bp|Finland|BOLD:ABZ6600
Diarsia mendica|MM02777|658[0n]bp|Finland|BOLD:ABZ6600
Diarsia mendica|MM18009|658[0n]bp|Finland|BOLD:ABZ6600
Diarsia mendica|MM04091|658[0n]bp|Finland.Lapland|BOLD:ABZ6600
Diarsia mendica|MM22780|658[0n]bp|Finland|BOLD:AAB0038
Diarsia mendica|MM08075|658[0n]bp|Finland|BOLD:AAB0038
Diarsia mendica|MM17915|658[0n]bp|Finland|BOLD:AAB0038
Diarsia mendica|MM18008|658[0n]bp|Finland|BOLD:AAB0038
Diarsia mendica|MM02776|658[0n]bp|Finland|BOLD:AAB0038
Diarsia mendica|MM01646|658[0n]bp|Finland.South Karelia|BOLD:AAB0038
Diarsia mendica|MM01647|658[0n]bp|Finland.South Karelia|BOLD:AAB0038
Diarsia mendica|MM09918|658[0n]bp|Finland|BOLD:AAB0038
Diarsia mendica|MM08462|658[0n]bp|Finland|BOLD:AAB0038
Diarsia mendica|MM08461|658[0n]bp|Finland|BOLD:AAB0038
Diarsia rubi|MM05103|622[0n]bp|Finland.Finland Proper|BOLD:ACE8687
Diarsia rubi|MM10959|658[0n]bp|Finland|BOLD:ACE8687
Diarsia rubi|MM01644|658[0n]bp|Finland.South Karelia|BOLD:ACE8687
Diarsia rubi|MM05102|658[0n]bp|Finland.Finland Proper|BOLD:ACE8687
Diarsia rubi|TLMF Lep 05621|658[0n]bp|Austria.Vorarlberg|BOLD:ACE8687
Diarsia rubi|MM01642|658[0n]bp|Finland.South Karelia|BOLD:ACE8687
Diarsia rubi|MM01643|658[0n]bp|Finland.South Karelia|BOLD:ACE8687
Diarsia rubi|MM15958|658[0n]bp|Finland|BOLD:ACE8687
Diarsia rubi|MM01645|658[0n]bp|Finland.South Karelia|BOLD:ACE8687
Diarsia rubi|MM12689|658[0n]bp|Finland|BOLD:ACE8687
Lycophotia porphyrea|MM10908|658[0n]bp|Finland|BOLD:AAC8982
Lycophotia porphyrea|MM05151|658[0n]bp|Finland.Finland Proper|BOLD:AAC8982
Lycophotia porphyrea|TLMF Lep 10023|658[0n]bp|Austria.Vorarlberg|BOLD:AAC8982
Lycophotia porphyrea|MM02760|658[0n]bp|Finland.Northern Ostrobothnia|BOLD:AAC8982
Cerastis leucographa|TLMF Lep 04649|628[0n]bp|Austria.Vorarlberg|BOLD:AAD9079
Cerastis leucographa|MM04038|658[0n]bp|Finland.Uusimaa|BOLD:AAD9079
Cerastis leucographa|MM01579|633[0n]bp|Finland.South Karelia|BOLD:AAD9079
Cerastis leucographa|MM01578|635[0n]bp|Finland.South Karelia|BOLD:AAD9079
Cerastis rubricosa|MM01575|658[0n]bp|Finland.South Karelia|BOLD:AAD2637
Cerastis rubricosa|MM01576|658[0n]bp|Finland.South Karelia|BOLD:AAD2637
Cerastis rubricosa|MM00410|658[0n]bp|Finland.Northern Ostrobothnia|BOLD:AAD2637
Cerastis rubricosa|TLMF Lep 04622|658[0n]bp|Austria.Vorarlberg|BOLD:AAD2637
Anaplectoides prasina|TLMF Lep 08136|658[0n]bp|Austria.Vorarlberg|BOLD:AAA2948
Anaplectoides prasina|MM01639|658[0n]bp|Finland.South Karelia|BOLD:AAA2948
Anaplectoides prasina|MM01638|658[0n]bp|Finland.South Karelia|BOLD:AAA2948
Anaplectoides prasina|MM12547|658[0n]bp|Finland|BOLD:AAA2948
Anaplectoides prasina|MM05089|658[0n]bp|Finland.Finland Proper|BOLD:AAA2948
Eurois occulta|MM02786|658[0n]bp|Finland.Northern Ostrobothnia|BOLD:AAA3312
Eurois occulta|TLMF Lep 00291|658[0n]bp|Austria.Vorarlberg|BOLD:AAA3312
Eurois|BIOUG04118-F08|630[0n]bp|Finland.Northern Ostrobothnia|BOLD:AAA3312
Eurois occulta|MM01687|612[0n]bp|Finland.South Karelia|BOLD:AAA3312
Eurois occulta|MM01688|613[0n]bp|Finland.South Karelia|BOLD:AAA3312
Graphiphora augur|MM08074|658[0n]bp|Finland|BOLD:ACF0935
Graphiphora augur|MM04870|658[0n]bp|Finland.Finland Proper|BOLD:ACF0935
Graphiphora augur|MM02757|658[0n]bp|Finland.Northern Ostrobothnia|BOLD:ACF0935
Graphiphora augur|TLMF Lep 06100|658[0n]bp|Austria.Vorarlberg|BOLD:ACF0935
Eugraphe sigma|MM19921|658[0n]bp|Finland.Uusimaa|BOLD:AAF8517
Eugraphe sigma|TLMF Lep 10024|658[0n]bp|Austria.Vorarlberg|BOLD:AAF8517
Eugraphe sigma|MM15963|658[1n]bp|Finland|BOLD:AAF8517
Epipsilia grisescens|MM15893|658[0n]bp|Finland|BOLD:AAF4729
Epipsilia grisescens|MM15892|658[0n]bp|Finland.Aland Islands|BOLD:AAF4729
Epipsilia grisescens|MM03657|658[0n]bp|Finland.Uusimaa|BOLD:AAF4729
Epipsilia grisescens|TLMF Lep 06099|658[0n]bp|Austria.Vorarlberg|BOLD:AAF4729
Standfussiana lucernea|TLMF Lep 06098|658[0n]bp|Austria.Vorarlberg|BOLD:AAE9254
Standfussiana lucernea|MM17499|658[0n]bp|Finland|BOLD:AAE9254
Standfussiana lucernea|MM17500|658[0n]bp|Finland|BOLD:AAE9254
Standfussiana lucernea|MM12509|658[0n]bp|Finland|BOLD:AAE9254
Standfussiana lucernea|MM17221|658[0n]bp|Finland|BOLD:AAE9254
Xestia alpicola|TLMF Lep 06101|658[0n]bp|Austria.Vorarlberg|BOLD:ABZ1718
Xestia alpicola|LN-BD0177|658[0n]bp|Finland.Northern Ostrobothnia|BOLD:ABZ1718
Xestia alpicola|MM12102|658[0n]bp|Finland|BOLD:ABZ1718
Xestia alpicola|MM08068|658[0n]bp|Finland|BOLD:ABZ1718
Xestia alpicola|MM18749|658[0n]bp|Finland|BOLD:ABZ1718
Xestia alpicola|MM08067|658[0n]bp|Finland|BOLD:ABZ1718
Xestia baja|MM00828|658[0n]bp|Finland.Northern Ostrobothnia|BOLD:AAA2590
Xestia baja|TLMF Lep 08549|658[0n]bp|Austria.Vorarlberg|BOLD:AAA2590
Xestia baja|TLMF Lep 08793|658[0n]bp|Austria.Vorarlberg|BOLD:AAA2590
Xestia baja|MM01658|643[0n]bp|Finland.South Karelia|BOLD:AAA2590
Xestia baja|MM01659|641[0n]bp|Finland.South Karelia|BOLD:AAA2590
Tholera cespitis|TLMF Lep 12549|658[0n]bp|Austria.Vorarlberg|BOLD:AAA6806
Xestia collina|TLMF Lep 06177|658[0n]bp|Austria.Vorarlberg|BOLD:AAE1853
Xestia collina|MM01634|648[0n]bp|Finland.South Karelia|BOLD:AAE1853

[illegible]
Tholera cespitis|TLMF Lep 12549|658[0n]bp|Austria.Vorarlberg|BOLD:AAA6806
Xestia collina|TLMF Lep 06177|658[0n]bp|Austria.Vorarlberg|BOLD:AAE1853
Xestia collina|MM01634|648[0n]bp|Finland.South Karelia|BOLD:AAE1853
Xestia collina|MM07403|658[0n]bp|Finland|BOLD:AAE1853
Xestia collina|MM01635|658[0n]bp|Finland.South Karelia|BOLD:AAE1853
Xestia speciosa|MM00840|658[0n]bp|Finland.Northern Ostrobothnia|BOLD:ACE4666
Xestia speciosa|MM08073|658[0n]bp|Finland|BOLD:ACE4666
Xestia speciosa|MM18760|658[0n]bp|Finland|BOLD:ACE4666
Xestia speciosa|MM02739|658[0n]bp|Finland.Northern Ostrobothnia|BOLD:ACE4666
Xestia speciosa|MM08072|658[0n]bp|Finland|BOLD:ACE4666
Xestia speciosa|MM06113|658[0n]bp|Finland|BOLD:ACE4666
Xestia speciosa|MM18762|658[0n]bp|Finland|BOLD:ACE4666
Xestia speciosa|TLMF Lep 00183|590[0n]bp|Austria.Vorarlberg|BOLD:ACF2698
Xestia speciosa|TLMF Lep 04431|658[0n]bp|Austria.Vorarlberg|BOLD:ACF2698
Xestia speciosa|TLMF Lep 00182|658[0n]bp|Austria.Vorarlberg|BOLD:ACF2698
Xestia speciosa|TLMF Lep 04432|658[0n]bp|Austria.Vorarlberg|BOLD:ACF2698
Xestia speciosa|TLMF Lep 08488|658[0n]bp|Austria.Vorarlberg|BOLD:ACF2698
Eugnorisma depuncta|MM12668|658[0n]bp|Finland|BOLD:AAE4805
Eugnorisma depuncta|TLMF Lep 08767|658[0n]bp|Austria.Vorarlberg|BOLD:AAE4806
Eugnorisma depuncta|MM04364|658[0n]bp|Finland.Finland Proper|BOLD:AAE4806
Eugnorisma depuncta|MM04892|658[0n]bp|Finland.Finland Proper|BOLD:AAE4806
Protolampra sobrina|MM01719|631[0n]bp|Finland.South Karelia|BOLD:AAE8181
Protolampra sobrina|TLMF Lep 08769|658[0n]bp|Austria.Vorarlberg|BOLD:AAE8181
Protolampra sobrina|MM01718|658[0n]bp|Finland.South Karelia|BOLD:AAE8181
Protolampra sobrina|MM02737|658[0n]bp|Finland.Northern Ostrobothnia|BOLD:AAE8181
Xestia ashworthii|TLMF Lep 00313|650[0n]bp|Austria.Vorarlberg|BOLD:ABY8432
Xestia ashworthii|MM17510|560[0n]bp|Finland|BOLD:ABY8432
Xestia ashworthii|MM21062|658[0n]bp|Finland|BOLD:ABY8432
Xestia ashworthii|MM17509|658[0n]bp|Finland|BOLD:ABY8432
Xestia ashworthii|MM21061|658[0n]bp|Finland|BOLD:ABY8432
Xestia c-nigrum|MM07709|614[0n]bp|Finland|BOLD:AAA2144
Xestia c-nigrum|MM06870|658[0n]bp|Finland.Aland Islands|BOLD:AAA2144
Xestia c-nigrum|TLMF Lep 07836|658[0n]bp|Austria.Vorarlberg|BOLD:AAA2144
Xestia c-nigrum|MM04640|658[0n]bp|Finland.Finland Proper|BOLD:AAA2144
Xestia c-nigrum|MM17284|658[0n]bp|Finland|BOLD:AAA2144
Xestia triangulum|MM05110|658[0n]bp|Finland.Finland Proper|BOLD:AAB8843
Xestia triangulum|MM01599|658[0n]bp|Finland.South Karelia|BOLD:AAB8843
Xestia triangulum|MM01600|634[0n]bp|Finland.South Karelia|BOLD:AAB8843
Xestia triangulum|TLMF Lep 08155|658[0n]bp|Austria.Vorarlberg|BOLD:AAB8843
Xestia triangulum|MM18833|658[0n]bp|Finland|BOLD:AAB8843
Elaphria venustula|MM18915|658[0n]bp|Finland|BOLD:AAE0188
Elaphria venustula|MM11069|658[0n]bp|Finland|BOLD:AAE0188
Elaphria venustula|MM06618|658[0n]bp|Finland|BOLD:AAE0188
Elaphria venustula|TLMF Lep 08158|658[0n]bp|Austria.Vorarlberg|BOLD:AAE0188
Agrochola macilenta|MM03183|658[0n]bp|Finland.Aland Islands|BOLD:AAC1555
Agrochola macilenta|TLMF Lep 06145|658[0n]bp|Austria.Vorarlberg|BOLD:AAC1555
Agrochola macilenta|MM04642|658[0n]bp|Finland.Finland Proper|BOLD:AAC1555
Agrochola macilenta|MM03182|658[0n]bp|Finland.Aland Islands|BOLD:AAC1555
Mormo maura|MM15942|658[0n]bp|Finland|BOLD:AAD8730
Mormo maura|TLMF Lep 09854|658[0n]bp|Austria.Vorarlberg|BOLD:AAD8730
Agrochola circellaris|MM06994|658[0n]bp|Finland|BOLD:AAC7613
Agrochola circellaris|MM04842|658[0n]bp|Finland.Finland Proper|BOLD:AAC7613
Agrochola circellaris|TLMF Lep 06144|658[0n]bp|Austria.Vorarlberg|BOLD:AAC7613
Agrochola circellaris|MM18040|637[0n]bp|Finland|BOLD:AAC7613
Hyppa rectilinea|TLMF Lep 00298|656[0n]bp|Austria.Vorarlberg|BOLD:ABZ5210
Hyppa rectilinea|TLMF Lep 08117|658[0n]bp|Austria.Vorarlberg|BOLD:ABZ5210
Hyppa rectilinea|MM00646|658[0n]bp|Finland.Northern Ostrobothnia|BOLD:ABZ5210
Hyppa rectilinea|MM14384|658[0n]bp|Finland.Aland Islands|BOLD:ABZ5210
Hyppa rectilinea|MM01655|638[0n]bp|Finland.South Karelia|BOLD:ABZ5210
Ipimorpha retusa|MM01702|658[0n]bp|Finland.South Karelia|BOLD:AAD8507
Ipimorpha retusa|MM04846|658[0n]bp|Finland.Finland Proper|BOLD:AAD8507
Ipimorpha retusa|TLMF Lep 08544|658[0n]bp|Austria.Vorarlberg|BOLD:AAD8507
Ipimorpha retusa|MM01701|658[1n]bp|Finland.South Karelia|BOLD:AAD8507
Brachylomia viminalis|MM00835|658[0n]bp|Finland.Northern Ostrobothnia|BOLD:AAC7236
Brachylomia viminalis|TLMF Lep 06124|658[0n]bp|Austria.Vorarlberg|BOLD:AAC7236
Brachylomia viminalis|MM01742|658[1n]bp|Finland.South Karelia|BOLD:AAC7236
Brachylomia viminalis|MM04876|632[0n]bp|Finland.Finland Proper|BOLD:AAC7236
Agrochola nitida|TLMF Lep 08759|658[0n]bp|Austria.Vorarlberg|BOLD:AAJ1527
Agrochola nitida|MM18905|658[0n]bp|Finland.Aland Islands|BOLD:AAJ1527
Agrochola nitida|MM18906|658[0n]bp|Finland.Aland Islands|BOLD:AAJ1527
Agrochola nitida|MM15950|658[0n]bp|Finland|BOLD:AAJ1527
Denticucullus pygmina|TLMF Lep 08763|658[0n]bp|Austria.Vorarlberg|BOLD:AAE9000
Denticucullus pygmina|MM04644|658[0n]bp|Finland.Finland Proper|BOLD:AAE9000
Denticucullus pygmina|MM02703|658[0n]bp|Finland.South Karelia|BOLD:AAE9000
Denticucullus pygmina|MM00830|658[0n]bp|Finland.Northern Ostrobothnia|BOLD:AAE9000
Ipimorpha subtusa|TLMF Lep 00305|658[0n]bp|Austria.Vorarlberg|BOLD:AAC2458
Ipimorpha subtusa|MM18044|658[0n]bp|Finland|BOLD:AAC2458
Ipimorpha subtusa|TLMF Lep 08543|658[0n]bp|Austria.Vorarlberg|BOLD:AAC2458
Ipimorpha subtusa|MM01698|658[3n]bp|Finland.South Karelia|BOLD:AAC2458
Ipimorpha subtusa|MM01699|658[3n]bp|Finland.South Karelia|BOLD:AAC2458
Mesoligia furuncula|MM01726|631[0n]bp|Finland.South Karelia|BOLD:AAC2820
Mesoligia furuncula|MM04869|658[0n]bp|Finland.Finland Proper|BOLD:AAC2820
Mesoligia furuncula|TLMF Lep 08559|658[0n]bp|Austria.Vorarlberg|BOLD:AAC2820
Mesoligia furuncula|MM01727|658[0n]bp|Finland.South Karelia|BOLD:AAC2820
Agrochola helvola|MM12649|658[0n]bp|Finland|BOLD:AAD6457
Agrochola helvola|MM08205|658[0n]bp|Finland|BOLD:AAD6457
Agrochola helvola|MM07738|658[0n]bp|Finland|BOLD:AAD6457
Agrochola helvola|TLMF Lep 09863|658[0n]bp|Austria.Vorarlberg|BOLD:AAD6457
Xanthia icteritia|TLMF Lep 08795|658[0n]bp|Austria.Vorarlberg|BOLD:AAC9311
Xanthia icteritia|MM01706|658[1n]bp|Finland.South Karelia|BOLD:AAC9311
Xanthia icteritia|MM23222|658[0n]bp|Finland|BOLD:AAC9311
Xanthia icteritia|MM01707|632[1n]bp|Finland.South Karelia|BOLD:AAC9311
Xanthia icteritia|MM08182|658[0n]bp|Finland|BOLD:AAC9311
Cosmia affinis|TLMF Lep 08555|658[0n]bp|Austria.Vorarlberg|BOLD:AAE9788
Cosmia affinis|MM09284|658[0n]bp|Finland|BOLD:AAE9788
Cosmia affinis|MM15945|658[0n]bp|Finland|BOLD:AAE9788
Cosmia trapezina|MM01697|658[1n]bp|Finland.South Karelia|BOLD:AAB9038
Cosmia trapezina|MM01696|658[0n]bp|Finland.South Karelia|BOLD:AAB9038
Cosmia trapezina|TLMF Lep 08162|658[0n]bp|Austria.Vorarlberg|BOLD:AAB9038
Cosmia trapezina|MM04883|658[0n]bp|Finland.Finland Proper|BOLD:AAB9038
Cosmia pyralina|TLMF Lep 08179|658[0n]bp|Austria.Vorarlberg|BOLD:AAE9789
Cosmia pyralina|MM18727|658[0n]bp|Finland|BOLD:AAE9789
[illegible]

Cosmia trapezina|MM04883|658[0n]bp|Finland.Finland Proper|BOLD:AAB9038
Cosmia pyralina|TLMF Lep 08179|658[0n]bp|Austria.Vorarlberg|BOLD:AAE9789
Cosmia pyralina|MM18727|658[0n]bp|Finland|BOLD:AAE9789
Cosmia pyralina|MM15946|658[0n]bp|Finland|BOLD:AAE9789
Cosmia pyralina|MM21084|658[0n]bp|Finland|BOLD:AAE9789
Cosmia pyralina|MM21165|658[0n]bp|Finland|BOLD:AAE9789
Apterogenum ypsillon|TLMF Lep 10022|658[0n]bp|Austria.Vorarlberg|BOLD:AAD1478
Apterogenum ypsillon|MM03517|634[0n]bp|Finland.Kymenlaakso|BOLD:AAD1478
Apterogenum ypsillon|MM12658|658[0n]bp|Finland|BOLD:AAD1478
Apterogenum ypsillon|MM03656|658[0n]bp|Finland.Uusimaa|BOLD:AAD1478
Enargia paleacea|MM01714|658[0n]bp|Finland.South Karelia|BOLD:AAA7455
Enargia paleacea|MM02745|658[0n]bp|Finland.Northern Ostrobothnia|BOLD:AAA7455
Enargia paleacea|TLMF Lep 12462|658[0n]bp|Austria.Vorarlberg|BOLD:AAA7455
Enargia paleacea|MM12553|658[0n]bp|Finland|BOLD:AAA7455
Enargia paleacea|MM01715|658[1n]bp|Finland.South Karelia|BOLD:AAA7455
Apamea crenata|MM01172|658[0n]bp|Finland.South Karelia|BOLD:AAC0154
Apamea crenata|MM01173|658[0n]bp|Finland.South Karelia|BOLD:AAC0154
Apamea crenata|TLMF Lep 07857|658[0n]bp|Austria.Vorarlberg|BOLD:AAC0154
Apamea crenata|MM01170|670[0n]bp|Finland.South Karelia|BOLD:AAC0154
Apamea crenata|MM04727|658[0n]bp|Finland.Finland Proper|BOLD:AAC0154
Apamea lateritia|TLMF Lep 08489|658[0n]bp|Austria.Vorarlberg|BOLD:ACF5481
Apamea lateritia|MM09935|658[0n]bp|Finland|BOLD:ACF5481
Apamea lateritia|MM01734|658[0n]bp|Finland.South Karelia|BOLD:ACF5481
Apamea lateritia|MM01735|658[0n]bp|Finland.South Karelia|BOLD:ACF5481
Apamea rubrirena|TLMF Lep 00311|658[0n]bp|Austria.Vorarlberg|BOLD:ACE5999
Apamea rubrirena|MM18032|658[0n]bp|Finland|BOLD:ACE5999
Apamea rubrirena|MM06042|658[0n]bp|Finland|BOLD:ACE5999
Apamea rubrirena|MM12235|658[0n]bp|Finland|BOLD:ACE5999
Apamea illyria|TLMF Lep 06187|658[0n]bp|Austria.Vorarlberg|BOLD:AAE6985
Apamea illyria|MM12610|658[0n]bp|Finland|BOLD:AAE6985
Apamea illyria|MM01594|639[0n]bp|Finland.South Karelia|BOLD:AAE6985
Apamea illyria|MM01595|638[0n]bp|Finland.South Karelia|BOLD:AAE6985
Apamea furva|TLMF Lep 08770|658[0n]bp|Austria.Vorarlberg|BOLD:AAC7157
Apamea furva|MM10783|658[0n]bp|Finland|BOLD:AAC7157
Apamea furva|MM18033|658[0n]bp|Finland|BOLD:AAC7157
Apamea furva|MM04908|658[0n]bp|Finland.Finland Proper|BOLD:AAC7157
Apamea unanimis|MM02396|658[0n]bp|Finland.South Karelia|BOLD:AAA8789
Apamea unanimis|MM01648|643[0n]bp|Finland.South Karelia|BOLD:AAA8789
Apamea unanimis|TLMF Lep 05617|658[0n]bp|Austria.Vorarlberg|BOLD:AAA8789
Apamea unanimis|MM18031|658[0n]bp|Finland|BOLD:AAA8789
Apamea monoglypha|TLMF Lep 08119|658[0n]bp|Austria.Vorarlberg|BOLD:AAB1551
Apamea monoglypha|MM04354|658[0n]bp|Finland.Finland Proper|BOLD:AAB1551
Apamea monoglypha|MM12653|658[0n]bp|Finland|BOLD:ACF3527
Apamea monoglypha|MM18034|658[0n]bp|Finland|BOLD:ACF3527
Apamea sublustris|MM14369|658[1n]bp|Finland.Aland Islands|BOLD:AAD3064
Apamea sublustris|MM17471|658[0n]bp|Finland|BOLD:AAD3064
Apamea sublustris|MM15877|658[0n]bp|Finland|BOLD:AAD3064
Apamea sublustris|TLMF Lep 08143|658[0n]bp|Austria.Vorarlberg|BOLD:AAD3064
Nonagria typhae|TLMF Lep 08863|658[0n]bp|Austria.Vorarlberg|BOLD:AAD0414
Nonagria typhae|MM10213|658[0n]bp|Finland|BOLD:AAD0414
Nonagria typhae|MM18026|658[0n]bp|Finland|BOLD:AAD0414
Nonagria typhae|MM18025|658[0n]bp|Finland|BOLD:AAD0414
Helotropha leucostigma|MM01743|658[0n]bp|Finland.South Karelia|BOLD:ACE3288
Helotropha leucostigma|TLMF Lep 08536|658[0n]bp|Austria.Vorarlberg|BOLD:ACE3288
Helotropha leucostigma|MM07767|612[0n]bp|Finland|BOLD:ACE3288
Helotropha leucostigma|MM04651|658[0n]bp|Finland.Finland Proper|BOLD:ACE3288
Apamea scolopacina|MM04905|658[0n]bp|Finland.Finland Proper|BOLD:AAC0150
Apamea scolopacina|TLMF Lep 08188|658[0n]bp|Austria.Vorarlberg|BOLD:AAC0150
Apamea scolopacina|MM01669|658[0n]bp|Finland.South Karelia|BOLD:AAC0150
Apamea scolopacina|MM01668|658[0n]bp|Finland.South Karelia|BOLD:AAC0150
Hydraecia micacea|MM01723|658[0n]bp|Finland.South Karelia|BOLD:AAB1631
Hydraecia micacea|MM01722|658[0n]bp|Finland.South Karelia|BOLD:AAB1631
Hydraecia micacea|MM12715|658[0n]bp|Finland|BOLD:AAB1631
Hydraecia micacea|MM00824|658[0n]bp|Finland.Northern Ostrobothnia|BOLD:AAB1631
Hydraecia micacea|TLMF Lep 08533|658[0n]bp|Austria.Vorarlberg|BOLD:AAB1631
Hydraecia micacea|MM04352|658[0n]bp|Finland.Finland Proper|BOLD:AAB1631
Hydraecia micacea|MM07283|658[0n]bp|Finland|BOLD:AAB1631
Hydraecia micacea|MM08012|658[0n]bp|Finland|BOLD:AAB1631
Hydraecia micacea|MM22089|658[0n]bp|Finland|BOLD:AAB1631
Crypsedra gemmea|MM01740|658[1n]bp|Finland.South Karelia|BOLD:AAF1443
Crypsedra gemmea|MM04845|658[0n]bp|Finland.Finland Proper|BOLD:AAF1443
Crypsedra gemmea|MM00832|658[0n]bp|Finland.Northern Ostrobothnia|BOLD:AAF1443
Crypsedra gemmea|TLMF Lep 08766|658[0n]bp|Austria.Vorarlberg|BOLD:AAF1443
Oligia strigilis|MM01667|658[0n]bp|Finland.South Karelia|BOLD:AAB4833
Oligia strigilis|TLMF Lep 07887|658[0n]bp|Austria.Vorarlberg|BOLD:AAB4833
Oligia strigilis|MM01666|658[0n]bp|Finland.South Karelia|BOLD:AAB4833
Oligia strigilis|TLMF Lep 07860|658[0n]bp|Austria.Vorarlberg|BOLD:AAB4833
Oligia strigilis|MM09474|658[0n]bp|Finland.Aland Islands|BOLD:AAB4833
Oligia strigilis|MM03663|658[0n]bp|Finland.Uusimaa|BOLD:AAB4833
Amphipoea fucosa|MM01672|633[0n]bp|Finland.South Karelia|BOLD:AAB5368
Amphipoea fucosa|MM11508|623[0n]bp|Finland|BOLD:AAB5368
Amphipoea fucosa|MM12702|658[0n]bp|Finland|BOLD:AAB5368
Amphipoea fucosa|MM08706|658[0n]bp|Finland|BOLD:AAB5368
Amphipoea fucosa|MM01676|658[0n]bp|Finland.South Karelia|BOLD:AAB5368
Amphipoea fucosa|MM04880|658[0n]bp|Finland.Finland Proper|BOLD:AAB5368
Amphipoea fucosa|MM08708|658[0n]bp|Finland|BOLD:AAB5368
Amphipoea fucosa|MM12703|658[0n]bp|Finland|BOLD:AAB5368
Amphipoea fucosa|MM13878|658[0n]bp|Finland.Lapland|BOLD:AAB5368
Amphipoea fucosa|MM04879|658[0n]bp|Finland.Finland Proper|BOLD:AAB5368
Amphipoea fucosa|MM06948|658[0n]bp|Finland|BOLD:AAB5368
Amphipoea fucosa|MM01675|658[0n]bp|Finland.South Karelia|BOLD:AAB5368
Amphipoea fucosa|MM01673|633[0n]bp|Finland.South Karelia|BOLD:AAB5368
Amphipoea fucosa|MM07394|638[0n]bp|Finland|BOLD:AAB5368
Amphipoea fucosa|MM06980|650[0n]bp|Finland|BOLD:AAB5368
Amphipoea fucosa|MM08707|658[0n]bp|Finland|BOLD:AAB5368
Amphipoea fucosa|MM12701|658[0n]bp|Finland|BOLD:AAB5368
Amphipoea fucosa|MM14736|658[0n]bp|Finland.Aland Islands|BOLD:AAB5368
Amphipoea fucosa|MM04878|658[0n]bp|Finland.Finland Proper|BOLD:AAB5368
Amphipoea fucosa|TLMF Lep 08535|658[0n]bp|Austria.Vorarlberg|BOLD:AAB5368
Amphipoea fucosa|MM01674|658[0n]bp|Finland.South Karelia|BOLD:AAB5368
Amphipoea fucosa|MM11509|658[0n]bp|Finland|BOLD:AAB5368
Amphipoea fucosa|MM07396|658[0n]bp|Finland|BOLD:AAB5368
Amphipoea fucosa|TLMF Lep 08180|658[0n]bp|Austria.Vorarlberg|BOLD:AAB5368

Amphipoea fucosa|MM11509|658[0n]bp|Finland|BOLD:AAB5368
Amphipoea fucosa|MM07396|658[0n]bp|Finland|BOLD:AAB5368
Amphipoea fucosa|TLMF Lep 08180|658[0n]bp|Austria.Vorarlberg|BOLD:AAB5368
Amphipoea fucosa|TLMF Lep 08181|658[0n]bp|Austria.Vorarlberg|BOLD:AAB5368
Amphipoea fucosa|MM06993|658[0n]bp|Finland|BOLD:AAB5368
Amphipoea oculea|MM01671|644[0n]bp|Finland.South Karelia|BOLD:AAC7752
Amphipoea oculea|TLMF Lep 04673|658[0n]bp|Austria.Vorarlberg|BOLD:AAC7752
Amphipoea oculea|MM01670|658[0n]bp|Finland.South Karelia|BOLD:AAC7752
Amphipoea oculea|MM00819|658[0n]bp|Finland.Northern Ostrobothnia|BOLD:AAC7752
Amphipoea oculea|MM08324|658[0n]bp|Finland|BOLD:AAC7752
Amphipoea oculea|MM04804|658[0n]bp|Finland.Finland Proper|BOLD:AAC7752
Lateroligia ophiogramma|TLMF Lep 08194|658[0n]bp|Austria.Vorarlberg|BOLD:AAB0872
Lateroligia ophiogramma|MM12687|658[0n]bp|Finland|BOLD:AAB0872
Lateroligia ophiogramma|MM10788|658[0n]bp|Finland|BOLD:AAB0872
Lateroligia ophiogramma|MM04871|658[0n]bp|Finland.Finland Proper|BOLD:AAB0872
Mesapamea secalella|MM07085|613[0n]bp|Finland|BOLD:AAB2749
Mesapamea secalella|TLMF Lep 09225|658[0n]bp|Austria.Vorarlberg|BOLD:AAB2749
Mesapamea secalella|MM07075|614[0n]bp|Finland|BOLD:AAB2749
Mesapamea secalella|MM07077|613[0n]bp|Finland|BOLD:AAB2749
Mesapamea secalella|MM07074|613[0n]bp|Finland|BOLD:AAB2749
Mesapamea secalella|MM07073|613[0n]bp|Finland|BOLD:AAB2749
Mesapamea secalella|MM07076|613[0n]bp|Finland|BOLD:AAB2749
Mesapamea secalella|MM07078|613[0n]bp|Finland|BOLD:AAB2749
Photedes fluxa|TLMF Lep 04454|648[0n]bp|Austria.Vorarlberg|BOLD:AAD8063
Photedes fluxa|MM01626|658[0n]bp|Finland.South Karelia|BOLD:AAD8063
Photedes fluxa|MM04907|658[0n]bp|Finland.Finland Proper|BOLD:AAD8063
Photedes fluxa|MM01627|658[0n]bp|Finland.South Karelia|BOLD:AAD8063
Griposia aprilina|MM18037|658[0n]bp|Finland|BOLD:AAC3647
Griposia aprilina|MM04757|658[0n]bp|Finland.Finland Proper|BOLD:AAC3647
Griposia aprilina|TLMF Lep 08784|658[0n]bp|Austria.Vorarlberg|BOLD:AAC3647
Griposia aprilina|MM04803|658[0n]bp|Finland.Finland Proper|BOLD:AAC3647
Mniotype adusta|MM00575|658[0n]bp|Finland.Northern Ostrobothnia|BOLD:AAD5982
Mniotype adusta|MM15875|658[0n]bp|Finland|BOLD:AAD5982
Mniotype adusta|MM15876|658[0n]bp|Finland|BOLD:AAD5982
Mniotype adusta|TLMF Lep 08776|658[0n]bp|Austria.Vorarlberg|BOLD:AAD5982
Mniotype adusta|MM17469|658[0n]bp|Finland|BOLD:AAD5982
Tiliacea aurago|TLMF Lep 06193|658[0n]bp|Austria.Vorarlberg|BOLD:AAD4895
Tiliacea aurago|MM18042|658[0n]bp|Finland|BOLD:AAD4895
Tiliacea aurago|MM03179|658[0n]bp|Finland.Aland Islands|BOLD:AAD4895
Tiliacea aurago|MM04792|658[0n]bp|Finland.Finland Proper|BOLD:AAD4895
Tiliacea citrago|TLMF Lep 08762|658[0n]bp|Austria.Vorarlberg|BOLD:AAF7502
Tiliacea citrago|MM18043|658[0n]bp|Finland|BOLD:AAF7502
Tiliacea citrago|MM06120|658[0n]bp|Finland|BOLD:AAF7502
Tiliacea citrago|MM15868|658[0n]bp|Finland|BOLD:AAF7502
Agrochola litura|MM04843|658[0n]bp|Finland.Finland Proper|BOLD:AAC8167
Agrochola litura|MM07739|658[0n]bp|Finland|BOLD:AAC8167
Agrochola litura|MM12647|658[0n]bp|Finland|BOLD:AAC8167
Agrochola litura|TLMF Lep 06196|658[0n]bp|Austria.Vorarlberg|BOLD:AAC8167
Xanthia togata|TLMF Lep 08768|658[0n]bp|Austria.Vorarlberg|BOLD:AAC9312
Xanthia togata|MM00814|658[0n]bp|Finland.Northern Ostrobothnia|BOLD:AAC9312
Xanthia togata|MM08183|658[0n]bp|Finland|BOLD:AAC9312
Xanthia togata|MM04794|658[0n]bp|Finland.Finland Proper|BOLD:AAC9312
Conistra rubiginea|MM04625|658[0n]bp|Finland.Finland Proper|BOLD:ACE5168
Conistra rubiginea|MM10790|658[0n]bp|Finland|BOLD:ACE5168
Conistra rubiginea|TLMF Lep 08810|658[0n]bp|Austria.Vorarlberg|BOLD:AAD3682
Conistra rubiginea|MM12191|658[0n]bp|Finland|BOLD:AAD3682
Conistra rubiginosa|MM14439|658[0n]bp|Finland|BOLD:AAF0863
Conistra rubiginosa|MM18039|658[0n]bp|Finland|BOLD:AAF0863
Conistra rubiginosa|MM14440|658[0n]bp|Finland|BOLD:AAF0863
Conistra rubiginosa|TLMF Lep 08785|658[0n]bp|Austria.Vorarlberg|BOLD:AAF0863
Conistra vaccinii|MM01580|638[0n]bp|Finland.South Karelia|BOLD:AAB7880
Conistra vaccinii|TLMF Lep 06194|635[0n]bp|Austria.Vorarlberg|BOLD:AAB7880
Conistra vaccinii|MM01581|658[0n]bp|Finland.South Karelia|BOLD:AAB7880
Conistra vaccinii|MM04615|658[0n]bp|Finland.Finland Proper|BOLD:AAB7880
Conistra vaccinii|MM00416|658[0n]bp|Finland.Northern Ostrobothnia|BOLD:AAB7880
Lithophane consocia|MM02763|658[0n]bp|Finland.Northern Ostrobothnia|BOLD:AAD9908
Lithophane consocia|MM01556|644[0n]bp|Finland.South Karelia|BOLD:AAD9908
Lithophane consocia|MM01555|658[0n]bp|Finland.South Karelia|BOLD:AAD9908
Lithophane consocia|TLMF Lep 08757|658[0n]bp|Austria.Vorarlberg|BOLD:AAD9908
Lithophane furcifera|TLMF Lep 09515|658[0n]bp|Austria.Vorarlberg|BOLD:AAJ2397
Lithophane furcifera|MM18038|658[0n]bp|Finland|BOLD:AAJ2397
Lithophane furcifera|MM10812|658[0n]bp|Finland|BOLD:AAJ2397
Lithophane furcifera|MM12670|658[0n]bp|Finland|BOLD:AAJ2397
Eupsilia transversa|MM04621|658[0n]bp|Finland.Finland Proper|BOLD:AAC7414
Eupsilia transversa|MM15867|658[0n]bp|Finland|BOLD:AAC7414
Eupsilia transversa|TLMF Lep 06142|658[0n]bp|Austria.Vorarlberg|BOLD:AAC7414
Eupsilia transversa|MM15866|629[0n]bp|Finland|BOLD:AAC7414
Eupsilia transversa|TLMF Lep 04653|658[0n]bp|Austria.Vorarlberg|BOLD:AAC7414
Eupsilia transversa|TLMF Lep 06195|658[0n]bp|Austria.Vorarlberg|BOLD:AAC7414
Mesogona oxalina|TLMF Lep 12466|658[0n]bp|Austria.Vorarlberg|BOLD:AAF2131
Mesogona oxalina|MM18726|658[0n]bp|Finland|BOLD:AAF2131
Mesogona oxalina|MM14776|557[0n]bp|Finland|BOLD:AAF2131
Mesogona oxalina|MM18913|658[0n]bp|Finland|BOLD:AAF2131
Agrochola lota|MM03181|658[0n]bp|Finland.Aland Islands|BOLD:AAC0283
Agrochola lota|MM02694|658[0n]bp|Finland.South Karelia|BOLD:AAC0283
Agrochola lota|MM18041|658[0n]bp|Finland|BOLD:AAC0283
Agrochola lota|TLMF Lep 06143|658[0n]bp|Austria.Vorarlberg|BOLD:AAC0283
Parastichtis suspecta|TLMF Lep 10026|658[0n]bp|Austria.Vorarlberg|BOLD:AAB4551
Parastichtis suspecta|MM02764|658[0n]bp|Finland.Northern Ostrobothnia|BOLD:AAB4551
Parastichtis suspecta|MM01717|621[1n]bp|Finland.South Karelia|BOLD:AAB4551
Parastichtis suspecta|MM01716|632[2n]bp|Finland.South Karelia|BOLD:AAB4551
Xylena vetusta|MM04503|658[0n]bp|Finland.Finland Proper|BOLD:AAC2612
Xylena vetusta|TLMF Lep 05820|658[0n]bp|Austria.Vorarlberg|BOLD:AAC2612
Xylena vetusta|MM01560|658[0n]bp|Finland.South Karelia|BOLD:AAC2612
Xylena vetusta|MM01559|638[0n]bp|Finland.South Karelia|BOLD:AAC2612
Borkhausenia fuscescens|TLMF Lep 08299|658[0n]bp|Austria.Vorarlberg|BOLD:AAD4407
Borkhausenia fuscescens|MM22786|658[0n]bp|Finland|BOLD:AAD4407
Borkhausenia fuscescens|TLMF Lep 09893|658[0n]bp|Austria.Vorarlberg|BOLD:AAD4407
Borkhausenia fuscescens|MM00132|670[0n]bp|Finland.Uusimaa|BOLD:AAD4407
Borkhausenia fuscescens|MM10400|658[0n]bp|Finland.Aland Islands|BOLD:AAD4407
Borkhausenia fuscescens|MM02424|658[0n]bp|Finland.South Karelia|BOLD:AAD4407
Borkhausenia fuscescens|MM09329|658[0n]bp|Finland|BOLD:AAD4407
Papilio machaon|TLMF Lep 09113|658[0n]bp|Austria.Vorarlberg|BOLD:AAA5810

Borkhausenia fuscescens|MM10400|658[0n]bp|Finland.Aland Islands|BOLD:AAD4407
Borkhausenia fuscescens|MM02424|658[0n]bp|Finland.South Karelia|BOLD:AAD4407
Borkhausenia fuscescens|MM09329|658[0n]bp|Finland|BOLD:AAD4407
Papilio machaon|TLMF Lep 09113|658[0n]bp|Austria.Vorarlberg|BOLD:AAA5810
Papilio machaon|MM05702|658[0n]bp|Finland.North Karelia|BOLD:AAA5810
Papilio machaon|MM00727|658[0n]bp|Finland|BOLD:AAA5810
Papilio machaon|MM05450|658[0n]bp|Finland.Northern Ostrobothnia|BOLD:AAA5810
Ethmia bipunctella|MM12518|658[0n]bp|Finland|BOLD:AAB2343
Ethmia bipunctella|MM13188|658[0n]bp|Finland|BOLD:AAB2343
Ethmia bipunctella|TLMF Lep 09932|658[0n]bp|Austria.Vorarlberg|BOLD:AAB2343
Ethmia bipunctella|MM05918|658[0n]bp|Finland|BOLD:AAB2343
Ethmia pusiella|MM00042|670[0n]bp|Finland.Northern Ostrobothnia|BOLD:AAE4537
Ethmia pusiella|MM05432|658[0n]bp|Finland.Uusimaa|BOLD:AAE4537
Ethmia pusiella|MM01224|658[0n]bp|Finland.South Karelia|BOLD:AAE4537
Ethmia pusiella|TLMF Lep 08402|658[0n]bp|Austria.Vorarlberg|BOLD:AAE4537
Ethmia pusiella|MM01223|658[0n]bp|Finland.South Karelia|BOLD:AAE4537
Hofmannophila pseudospretella|MM10373|658[0n]bp|Finland.Aland Islands|BOLD:AAB0831
Hofmannophila pseudospretella|MM04345|630[0n]bp|Finland.Finland Proper|BOLD:AAB0831
Hofmannophila pseudospretella|TLMF Lep 07525|658[0n]bp|Austria.Vorarlberg|BOLD:AAB0831
Hofmannophila pseudospretella|TLMF Lep 08231|658[0n]bp|Austria.Vorarlberg|BOLD:AAB0831
Hofmannophila pseudospretella|MM10372|658[0n]bp|Finland.Aland Islands|BOLD:AAB0831
Lomographa bimaculata|TLMF Lep 07864|658[0n]bp|Austria.Vorarlberg|BOLD:AAB9407
Lomographa bimaculata|MM01311|658[0n]bp|Finland.South Karelia|BOLD:AAB9407
Lomographa bimaculata|MM01310|658[0n]bp|Finland.South Karelia|BOLD:AAB9407
Lomographa bimaculata|MM03704|658[0n]bp|Finland.Aland Islands|BOLD:AAB9407
Bucculatrix frangutella|TLMF Lep 08063|658[0n]bp|Austria.Vorarlberg|BOLD:AAI8491
Bucculatrix frangutella|MM18082|658[0n]bp|Finland|BOLD:AAI8491
Bucculatrix frangutella|MM18081|658[0n]bp|Finland|BOLD:AAI8491
Bucculatrix frangutella|MM06179|658[0n]bp|Finland|BOLD:AAI8491
Geometra papilionaria|MM01146|670[0n]bp|Finland.South Karelia|BOLD:AAB2012
Geometra papilionaria|MM09837|658[0n]bp|Finland.Aland Islands|BOLD:AAB2012
Geometra papilionaria|MM01147|658[0n]bp|Finland.South Karelia|BOLD:AAB2012
Geometra papilionaria|MM02809|632[0n]bp|Finland.Northern Ostrobothnia|BOLD:AAB2012
Geometra papilionaria|TLMF Lep 08775|658[0n]bp|Austria.Vorarlberg|BOLD:AAB2012
Paranthrene tabaniformis|TLMF Lep 09089|615[0n]bp|Austria.Vorarlberg|BOLD:ACF0532
Paranthrene tabaniformis|MM15756|658[0n]bp|Finland|BOLD:ACF0531
Paranthrene tabaniformis|MM08396|658[0n]bp|Finland|BOLD:ACF0531
Paranthrene tabaniformis|MM08395|670[0n]bp|Finland.Northern Ostrobothnia|BOLD:ACF0531
Notodonta dromedarius|MM04512|658[0n]bp|Finland.Finland Proper|BOLD:AAC1146
Notodonta dromedarius|MM10751|633[0n]bp|Finland|BOLD:AAC1146
Notodonta dromedarius|MM07685|658[0n]bp|Finland|BOLD:AAC1146
Notodonta dromedarius|MM00121|658[0n]bp|Finland.Uusimaa|BOLD:AAC1146
Notodonta dromedarius|MM00998|670[0n]bp|Finland.South Karelia|BOLD:AAC1146
Notodonta dromedarius|TLMF Lep 08532|658[0n]bp|Austria.Vorarlberg|BOLD:AAC1145
Notodonta dromedarius|TLMF Lep 04662|658[0n]bp|Austria.Vorarlberg|BOLD:AAC1145
Notodonta dromedarius|TLMF Lep 07872|658[0n]bp|Austria.Vorarlberg|BOLD:AAC1145
Notodonta dromedarius|TLMF Lep 08511|645[0n]bp|Austria.Vorarlberg|BOLD:AAC1145
Notodonta dromedarius|TLMF Lep 08141|658[0n]bp|Austria.Vorarlberg|BOLD:AAC1145
Notodonta ziczac|MM01002|658[0n]bp|Finland.South Karelia|BOLD:ABY9866
Notodonta ziczac|TLMF Lep 07828|658[0n]bp|Austria.Vorarlberg|BOLD:ABY9866
Notodonta ziczac|MM04923|658[0n]bp|Finland.Finland Proper|BOLD:ABY9866
Notodonta ziczac|MM01001|658[0n]bp|Finland.South Karelia|BOLD:ABY9866
Malacosoma neustria|TLMF Lep 09500|658[0n]bp|Austria.Vorarlberg|BOLD:AAD1119
Malacosoma neustria|MM17218|658[0n]bp|Finland|BOLD:AAD1119
Malacosoma neustria|MM17217|658[0n]bp|Finland|BOLD:AAD1119
Tischeria ekebladella|MM00943|666[0n]bp|Finland.Aland Islands|BOLD:AAF8247
Tischeria ekebladella|MM00945|658[0n]bp|Finland.Aland Islands|BOLD:AAF8247
Tischeria ekebladella|TLMF Lep 08062|658[0n]bp|Austria.Vorarlberg|BOLD:AAF8247
Tischeria ekebladella|MM19896|658[0n]bp|Finland.Finland Proper|BOLD:AAF8247
Tischeria ekebladella|MM00033|642[0n]bp|Finland.Aland Islands|BOLD:AAF8247
Tischeria ekebladella|MM05331|608[0n]bp|Finland.Finland Proper|BOLD:AAF8247
Idaea biselata|MM02918|632[0n]bp|Finland.Northern Ostrobothnia|BOLD:AAB2239
Idaea biselata|MM12904|658[0n]bp|Finland|BOLD:AAB2239
Idaea biselata|TLMF Lep 08189|658[0n]bp|Austria.Vorarlberg|BOLD:AAB2239
Idaea biselata|MM01508|658[0n]bp|Finland.South Karelia|BOLD:AAB2239
Idaea seriata|TLMF Lep 09896|658[0n]bp|Austria.Vorarlberg|BOLD:AAA9645
Idaea seriata|MM06892|658[0n]bp|Finland.Aland Islands|BOLD:ACF4900
Idaea seriata|MM18414|658[0n]bp|Finland|BOLD:ACF4900
Idaea seriata|TLMF Lep 08191|658[0n]bp|Austria.Vorarlberg|BOLD:ACF4900
Idaea seriata|MM10367|658[0n]bp|Finland.Aland Islands|BOLD:ACF4900
Idaea muricata|MM09756|658[0n]bp|Finland.Aland Islands|BOLD:AAD4604
Idaea muricata|MM06826|658[0n]bp|Finland.Aland Islands|BOLD:AAD4604
Idaea muricata|TLMF Lep 08538|658[0n]bp|Austria.Vorarlberg|BOLD:AAD4604
Idaea muricata|MM09467|658[0n]bp|Finland.Aland Islands|BOLD:AAD4604
Idaea serpentata|TLMF Lep 08282|658[0n]bp|Austria.Vorarlberg|BOLD:AAA9647
Idaea serpentata|MM06505|658[0n]bp|Finland|BOLD:AAA9647
Idaea serpentata|MM03203|658[0n]bp|Finland.Northern Ostrobothnia|BOLD:AAA9647
Idaea serpentata|MM10355|658[0n]bp|Finland|BOLD:AAA9647
Batrachedra pinicolella|MM21054|658[0n]bp|Finland|BOLD:AAF0078
Batrachedra pinicolella|MM21052|658[0n]bp|Finland|BOLD:AAF0078
Batrachedra pinicolella|MM06705|658[0n]bp|Finland.Aland Islands|BOLD:AAF0078
Batrachedra pinicolella|MM16052|616[0n]bp|Finland|BOLD:AAF0077
Batrachedra pinicolella|MM22067|658[0n]bp|Finland|BOLD:AAF0077
Batrachedra pinicolella|MM09830|658[0n]bp|Finland.Aland Islands|BOLD:AAF0077
Batrachedra pinicolella|MM16054|658[0n]bp|Finland|BOLD:AAF0077
Batrachedra pinicolella|MM16053|658[0n]bp|Finland|BOLD:AAF0077
Batrachedra pinicolella|MM21053|658[0n]bp|Finland|BOLD:AAF0077
Batrachedra pinicolella|MM22068|658[0n]bp|Finland|BOLD:AAF0077
Batrachedra pinicolella|MM21051|658[0n]bp|Finland|BOLD:AAF0077
Batrachedra pinicolella|MM22065|658[0n]bp|Finland|BOLD:AAF0077
Batrachedra pinicolella|MM06674|658[0n]bp|Finland|BOLD:AAF0077
Batrachedra pinicolella|MM17247|658[0n]bp|Finland|BOLD:AAF0077
Batrachedra pinicolella|TLMF Lep 08072|658[0n]bp|Austria.Vorarlberg|BOLD:AAF0077
Batrachedra pinicolella|MM22066|658[0n]bp|Finland|BOLD:AAF0077
Batrachedra pinicolella|MM21056|658[0n]bp|Finland|BOLD:AAF0077
Batrachedra pinicolella|MM21055|658[0n]bp|Finland|BOLD:AAF0077
Batrachedra praeangusta|TLMF Lep 12479|532[0n]bp|Austria.Vorarlberg|BOLD:ABZ6897
Batrachedra praeangusta|MM02328|670[0n]bp|Finland.South Karelia|BOLD:ABZ6897
Batrachedra praeangusta|MM10275|658[0n]bp|Finland|BOLD:ABZ6897
Batrachedra praeangusta|MM05053|658[0n]bp|Finland.Finland Proper|BOLD:ABZ6897
Scopula caricaria|TLMF Lep 08526|658[0n]bp|Austria.Vorarlberg|BOLD:AAF0636
Scopula caricaria|MM17860|658[0n]bp|Finland|BOLD:AAF0636
Scopula caricaria|MM17859|658[0n]bp|Finland|BOLD:AAF0636
[illegible]

Scopula caricaria|TLMF Lep 08526|658[0n]bp|Austria.Vorarlberg|BOLD:AAF0636
Scopula caricaria|MM17860|658[0n]bp|Finland|BOLD:AAF0636
Scopula caricaria|MM17859|658[0n]bp|Finland|BOLD:AAF0636
Scopula caricaria|MM10462|658[0n]bp|Finland|BOLD:AAF0636
Cleora cinctaria|MM01299|658[0n]bp|Finland.South Karelia|BOLD:AAC4786
Cleora cinctaria|MM00450|658[0n]bp|Finland.South Karelia|BOLD:AAC4786
Cleora cinctaria|MM04057|638[0n]bp|Finland.Northern Ostrobothnia|BOLD:AAC4786
Cleora cinctaria|MM04632|658[0n]bp|Finland.Finland Proper|BOLD:AAC4786
Cleora cinctaria|TLMF Lep 04630|658[0n]bp|Austria.Vorarlberg|BOLD:AAC4786
Endromis versicolora|MM12175|658[0n]bp|Finland|BOLD:AAD4912
Endromis versicolora|MM02802|634[0n]bp|Finland.Northern Ostrobothnia|BOLD:AAD4912
Endromis versicolora|MM00394|670[0n]bp|Finland.Northern Ostrobothnia|BOLD:AAD4912
Endromis versicolora|MM07742|656[0n]bp|Finland|BOLD:AAD4912
Endromis versicolora|TLMF Lep 09774|658[0n]bp|Austria.Vorarlberg|BOLD:AAD4912
Gnophos obfuscata|MM12883|658[0n]bp|Finland|BOLD:ABZ5475
Gnophos obfuscata|MM15843|658[0n]bp|Finland|BOLD:ABZ5475
Gnophos obfuscata|MM15842|658[0n]bp|Finland|BOLD:ABZ5475
Gnophos obfuscata|TLMF Lep 00310|658[0n]bp|Austria.Vorarlberg|BOLD:ABZ5475
Ammoconia caecimacula|TLMF Lep 08760|658[0n]bp|Austria.Vorarlberg|BOLD:AAE6007
Ammoconia caecimacula|MM18036|658[0n]bp|Finland|BOLD:AAE6007
Ammoconia caecimacula|MM15871|658[0n]bp|Finland|BOLD:AAE6007
Ammoconia caecimacula|MM15872|658[0n]bp|Finland|BOLD:AAE6007
Antitype chi|MM04796|658[0n]bp|Finland.Finland Proper|BOLD:AAE7040
Antitype chi|MM02699|658[0n]bp|Finland.South Karelia|BOLD:AAE7040
Antitype chi|MM12719|658[0n]bp|Finland|BOLD:AAE7040
Antitype chi|TLMF Lep 06192|658[0n]bp|Austria.Vorarlberg|BOLD:AAE7040
Mniotype satura|TLMF Lep 08792|658[0n]bp|Austria.Vorarlberg|BOLD:AAD0227
Mniotype satura|MM07372|658[0n]bp|Finland|BOLD:AAD0227
Mniotype satura|MM01736|658[1n]bp|Finland.South Karelia|BOLD:AAD0227
Mniotype satura|MM01737|658[0n]bp|Finland.South Karelia|BOLD:AAD0227
Spodoptera exigua|MM15939|658[0n]bp|Finland|BOLD:AAA6644
Spodoptera exigua|TLMF Lep 06197|658[0n]bp|Austria.Vorarlberg|BOLD:AAA6644
Spodoptera exigua|MM15940|658[0n]bp|Finland|BOLD:AAA6644
Spodoptera exigua|MM21046|627[0n]bp|Finland|BOLD:AAA6644
Apeira syringaria|TLMF Lep 10020|658[0n]bp|Austria.Vorarlberg|BOLD:AAD4663
Apeira syringaria|MM01503|658[0n]bp|Finland.South Karelia|BOLD:AAD4663
Apeira syringaria|MM02397|649[0n]bp|Finland.South Karelia|BOLD:AAD4663
Cabera exanthemata|MM05166|627[0n]bp|Finland.Finland Proper|BOLD:AAA6653
Cabera exanthemata|MM00640|658[0n]bp|Finland.Northern Ostrobothnia|BOLD:AAA6653
Cabera exanthemata|TLMF Lep 07909|658[0n]bp|Austria.Vorarlberg|BOLD:AAA6653
Cabera exanthemata|MM01306|658[0n]bp|Finland.South Karelia|BOLD:AAA6653
Cabera pusaria|TLMF Lep 08127|658[0n]bp|Austria.Vorarlberg|BOLD:AAA9589
Cabera pusaria|TLMF Lep 04658|658[0n]bp|Austria.Vorarlberg|BOLD:AAA9589
Cabera pusaria|BIOUG04118-E03|621[0n]bp|Finland.Northern Ostrobothnia|BOLD:AAA9589
Cabera pusaria|BIOUG04118-E04|620[0n]bp|Finland.Northern Ostrobothnia|BOLD:AAA9589
Cabera pusaria|BIOUG04116-C07|630[0n]bp|Finland.Northern Ostrobothnia|BOLD:AAA9589
Cabera pusaria|BIOUG04118-B03|630[0n]bp|Finland.Northern Ostrobothnia|BOLD:AAA9589
Cabera pusaria|BIOUG04116-C06|614[0n]bp|Finland.Northern Ostrobothnia|BOLD:AAA9589
Cabera pusaria|MM00589|658[0n]bp|Finland.Northern Ostrobothnia|BOLD:AAA9589
Cabera pusaria|BIOUG04116-F02|630[0n]bp|Finland.Northern Ostrobothnia|BOLD:AAA9589
Cabera pusaria|MM01304|658[0n]bp|Finland.South Karelia|BOLD:AAA9589
Cabera pusaria|MM01305|658[0n]bp|Finland.South Karelia|BOLD:AAA9589
Cabera pusaria|BIOUG04116-C08|614[0n]bp|Finland.Northern Ostrobothnia|BOLD:AAA9589
Plagodis dolabraria|TLMF Lep 04634|658[0n]bp|Austria.Vorarlberg|BOLD:AAB8023
Plagodis dolabraria|MM01253|631[0n]bp|Finland.South Karelia|BOLD:AAB8023
Plagodis dolabraria|MM01252|658[0n]bp|Finland.South Karelia|BOLD:AAB8023
Plagodis dolabraria|MM04669|658[0n]bp|Finland.Finland Proper|BOLD:AAB8023
Plagodis pulveraria|MM00503|658[0n]bp|Finland.Lapland|BOLD:AAA6014
Plagodis|BIOUG04490-E08|621[0n]bp|Finland.Northern Ostrobothnia|BOLD:AAA6014
Plagodis|BIOUG04118-A05|627[0n]bp|Finland.Northern Ostrobothnia|BOLD:AAA6014
Plagodis pulveraria|MM01261|658[0n]bp|Finland.South Karelia|BOLD:AAA6014
Plagodis pulveraria|MM01262|658[0n]bp|Finland.South Karelia|BOLD:AAA6014
Plagodis pulveraria|TLMF Lep 04667|658[1n]bp|Austria.Vorarlberg|BOLD:AAA6014
Colotois pennaria|TLMF Lep 06122|658[0n]bp|Austria.Vorarlberg|BOLD:AAB0886
Colotois pennaria|MM02693|658[0n]bp|Finland.South Karelia|BOLD:AAB0887
Colotois pennaria|MM12218|658[0n]bp|Finland|BOLD:AAB0887
Colotois pennaria|MM18507|658[0n]bp|Finland|BOLD:AAB0887
Lomographa temerata|TLMF Lep 07865|658[0n]bp|Austria.Vorarlberg|BOLD:AAB5203
Lomographa temerata|MM01309|658[0n]bp|Finland.South Karelia|BOLD:AAB5203
Lomographa temerata|MM03703|658[0n]bp|Finland.Aland Islands|BOLD:AAB5203
Lomographa temerata|MM01308|658[2n]bp|Finland.South Karelia|BOLD:AAB5203
Abraxas sylvata|MM01459|658[0n]bp|Finland.South Karelia|BOLD:AAC2560
Abraxas sylvata|TLMF Lep 08122|658[0n]bp|Austria.Vorarlberg|BOLD:AAC2560
Abraxas sylvata|MM10848|658[0n]bp|Finland|BOLD:AAC2560
Abraxas sylvata|MM01282|658[0n]bp|Finland.South Karelia|BOLD:AAC2560
Abraxas sylvata|MM01460|658[0n]bp|Finland.South Karelia|BOLD:AAC2560
Abraxas sylvata|MM01283|658[0n]bp|Finland.South Karelia|BOLD:AAC2560
Paradarisa consonaria|MM01515|658[0n]bp|Finland.South Karelia|BOLD:AAC3271
Paradarisa consonaria|MM01294|658[0n]bp|Finland.South Karelia|BOLD:AAC3271
Paradarisa consonaria|MM03807|636[0n]bp|Finland.Uusimaa|BOLD:AAC3271
Paradarisa consonaria|TLMF Lep 04660|658[0n]bp|Austria.Vorarlberg|BOLD:AAC3271
Paradarisa consonaria|MM01292|658[0n]bp|Finland.South Karelia|BOLD:AAC3271
Hypoxystis pluviaria|MM17991|658[0n]bp|Finland|BOLD:AAD1512
Hypoxystis pluviaria|MM15837|633[0n]bp|Finland|BOLD:AAD1512
Hypoxystis pluviaria|MM12189|658[0n]bp|Finland|BOLD:AAD1512
Hypoxystis pluviaria|MM01752|634[0n]bp|Finland.South Karelia|BOLD:AAD1512
Hypoxystis pluviaria|TLMF Lep 10010|658[0n]bp|Austria.Vorarlberg|BOLD:AAD1512
Jodis lactearia|MM01504|658[0n]bp|Finland.South Karelia|BOLD:AAD4811
Jodis lactearia|MM14292|658[0n]bp|Finland.Aland Islands|BOLD:AAD4811
Jodis lactearia|TLMF Lep 07976|658[0n]bp|Austria.Vorarlberg|BOLD:AAD4811
Jodis lactearia|MM01505|645[1n]bp|Finland.South Karelia|BOLD:AAD4811
Jodis putata|MM01506|658[0n]bp|Finland.South Karelia|BOLD:ABZ4040
Jodis putata|MM08170|639[0n]bp|Finland|BOLD:ABZ4040
Jodis putata|TLMF Lep 10004|658[0n]bp|Austria.Vorarlberg|BOLD:ABZ4040
Jodis putata|MM01507|658[0n]bp|Finland.South Karelia|BOLD:ABZ4040
Selenia lunularia|TLMF Lep 07840|658[0n]bp|Austria.Vorarlberg|BOLD:ABY7097
Selenia lunularia|MM03711|658[0n]bp|Finland.Aland Islands|BOLD:AAB1585
Selenia lunularia|MM03764|658[0n]bp|Finland.Aland Islands|BOLD:AAB1585
Selenia lunularia|MM03387|658[0n]bp|Finland.Aland Islands|BOLD:AAB1585
Selenia dentaria|MM08423|658[0n]bp|Finland|BOLD:AAB7343
Selenia dentaria|MM01267|658[0n]bp|Finland.South Karelia|BOLD:AAB7343
Selenia dentaria|MM00475|648[0n]bp|Finland.Northern Ostrobothnia|BOLD:AAB7343
Selenia dentaria|TLMF Lep 08525|658[0n]bp|Austria.Vorarlberg|BOLD:AAB7343

Selenia dentaria|MM01267|658[0n]bp|Finland.South Karelia|BOLD:AAB7343
Selenia dentaria|MM00475|648[0n]bp|Finland.Northern Ostrobothnia|BOLD:AAB7343
Selenia dentaria|TLMF Lep 08525|658[0n]bp|Austria.Vorarlberg|BOLD:AAB7343
Selenia dentaria|MM01268|658[0n]bp|Finland.South Karelia|BOLD:AAB7343
Selenia tetralunaria|MM01360|658[0n]bp|Finland.South Karelia|BOLD:AAB7329
Selenia tetralunaria|MM01353|648[0n]bp|Finland.South Karelia|BOLD:AAB7329
Selenia tetralunaria|MM08421|658[0n]bp|Finland|BOLD:AAB7329
Selenia tetralunaria|TLMF Lep 07843|658[0n]bp|Austria.Vorarlberg|BOLD:AAB7329
Selenia tetralunaria|MM01266|658[0n]bp|Finland.South Karelia|BOLD:AAB7329
Selenia tetralunaria|MM10853|658[0n]bp|Finland|BOLD:AAB7329
Biston betularia|MM02799|658[0n]bp|Finland.Northern Ostrobothnia|BOLD:ABY9367
Biston betularia|MM01227|637[0n]bp|Finland.South Karelia|BOLD:ABY9367
Biston betularia|MM01127|598[0n]bp|Finland.South Karelia|BOLD:ABY9367
Biston betularia|MM01128|610[0n]bp|Finland.South Karelia|BOLD:ABY9367
Biston betularia|TLMF Lep 07841|658[0n]bp|Austria.Vorarlberg|BOLD:ABY9367
Biston betularia|MM05118|658[0n]bp|Finland.Finland Proper|BOLD:ABY9367
Biston strataria|TLMF Lep 08814|658[0n]bp|Austria.Vorarlberg|BOLD:AAB4693
Biston strataria|MM04608|658[0n]bp|Finland.Finland Proper|BOLD:AAB4693
Biston strataria|MM02367|658[0n]bp|Finland.South Karelia|BOLD:AAB4693
Biston strataria|MM12173|658[0n]bp|Finland|BOLD:AAB4693
Biston strataria|MM04607|658[0n]bp|Finland.Finland Proper|BOLD:AAB4693
Petrophora chlorosata|MM04506|658[0n]bp|Finland.Finland Proper|BOLD:AAC0420
Petrophora chlorosata|TLMF Lep 07973|658[0n]bp|Austria.Vorarlberg|BOLD:AAC0420
Petrophora chlorosata|MM17371|658[0n]bp|Finland|BOLD:AAC0420
Petrophora chlorosata|MM05420|658[0n]bp|Finland.Aland Islands|BOLD:AAC0420
Ectropis crepuscularia|TLMF Lep 08176|658[0n]bp|Austria.Vorarlberg|BOLD:ACE6053
Ectropis crepuscularia|MM04502|658[0n]bp|Finland.Finland Proper|BOLD:ACE6053
Ectropis crepuscularia|MM01293|658[0n]bp|Finland.South Karelia|BOLD:ACE6053
Ectropis crepuscularia|MM07791|658[0n]bp|Finland|BOLD:ACE6053
Ectropis crepuscularia|MM07792|658[0n]bp|Finland|BOLD:ACE6053
Ectropis crepuscularia|MM01428|658[0n]bp|Finland.South Karelia|BOLD:ACE6053
Ectropis crepuscularia|MM18511|658[4n]bp|Finland|BOLD:ACE6053
Ectropis crepuscularia|MM17370|658[0n]bp|Finland|BOLD:AAA2076
Ectropis crepuscularia|MM00643|658[0n]bp|Finland.Northern Ostrobothnia|BOLD:AAA2076
Ectropis crepuscularia|MM14382|656[0n]bp|Finland.Aland Islands|BOLD:AAA2076
Ectropis crepuscularia|MM11593|658[0n]bp|Finland|BOLD:AAA2076
Ectropis crepuscularia|MM00472|658[0n]bp|Finland.Northern Ostrobothnia|BOLD:AAA2076
Ectropis crepuscularia|MM18512|658[0n]bp|Finland|BOLD:AAA2076
Ectropis crepuscularia|MM14383|658[0n]bp|Finland.Aland Islands|BOLD:AAA2076
Ectropis crepuscularia|MM22781|658[0n]bp|Finland|BOLD:AAA2076
Ectropis crepuscularia|MM12856|658[0n]bp|Finland|BOLD:AAA2076
Ectropis crepuscularia|MM11592|658[0n]bp|Finland|BOLD:AAA2076
Ectropis crepuscularia|MM06210|658[0n]bp|Finland|BOLD:AAA2076
Ectropis crepuscularia|MM17219|658[0n]bp|Finland|BOLD:AAA2076
Ectropis crepuscularia|MM17992|658[0n]bp|Finland|BOLD:AAA2076
Deileptenia ribeata|TLMF Lep 08187|658[0n]bp|Austria.Vorarlberg|BOLD:AAC3800
Deileptenia ribeata|MM06692|658[0n]bp|Finland.Aland Islands|BOLD:AAC3800
Deileptenia ribeata|MM06638|658[0n]bp|Finland|BOLD:AAC3800
Deileptenia ribeata|MM04588|658[0n]bp|Finland.Finland Proper|BOLD:AAC3800
Opisthograptis luteolata|MM01350|629[0n]bp|Finland.South Karelia|BOLD:AAA9865
Opisthograptis luteolata|MM04672|658[0n]bp|Finland.Finland Proper|BOLD:AAA9865
Opisthograptis luteolata|TLMF Lep 04632|658[0n]bp|Austria.Vorarlberg|BOLD:AAA9865
Opisthograptis luteolata|MM12768|658[0n]bp|Finland|BOLD:AAA9865
Campaea margaritaria|MM09755|639[0n]bp|Finland.Aland Islands|BOLD:AAB5336
Campaea margaritaria|MM06747|658[0n]bp|Finland.Aland Islands|BOLD:AAB5336
Campaea margaritaria|TLMF Lep 07907|658[0n]bp|Austria.Vorarlberg|BOLD:AAB5336
Campaea margaritaria|MM09754|658[0n]bp|Finland.Aland Islands|BOLD:AAB5336
Odontopera bidentata|MM00648|658[0n]bp|Finland.Northern Ostrobothnia|BOLD:AAB6560
Odontopera bidentata|MM04666|658[0n]bp|Finland.Finland Proper|BOLD:AAB6560
Odontopera bidentata|TLMF Lep 04659|658[0n]bp|Austria.Vorarlberg|BOLD:AAB6560
Odontopera bidentata|MM01486|658[0n]bp|Finland.South Karelia|BOLD:AAB6560
Angerona prunaria|MM01271|658[0n]bp|Finland.South Karelia|BOLD:AAB7137
Angerona prunaria|MM06691|658[0n]bp|Finland.Aland Islands|BOLD:AAB7137
Angerona prunaria|MM01270|658[0n]bp|Finland.South Karelia|BOLD:AAB7137
Angerona prunaria|TLMF Lep 08153|658[0n]bp|Austria.Vorarlberg|BOLD:AAB7137
Angerona prunaria|MM01477|658[0n]bp|Finland.South Karelia|BOLD:AAB7137
Hylaea fasciaria|MM06506|658[0n]bp|Finland|BOLD:AAB6675
Hylaea fasciaria|MM01385|633[0n]bp|Finland.South Karelia|BOLD:AAB6675
Hylaea fasciaria|MM01386|634[0n]bp|Finland.South Karelia|BOLD:AAB6675
Hylaea fasciaria|TLMF Lep 08132|658[0n]bp|Austria.Vorarlberg|BOLD:AAB6675
Ourapteryx sambucaria|TLMF Lep 08156|658[0n]bp|Austria.Vorarlberg|BOLD:AAB4472
Ourapteryx sambucaria|MM09715|658[0n]bp|Finland.Aland Islands|BOLD:AAB4472
Ourapteryx sambucaria|MM01150|658[0n]bp|Finland.South Karelia|BOLD:AAB4472
Ourapteryx sambucaria|MM01149|658[0n]bp|Finland.South Karelia|BOLD:AAB4472
Ourapteryx sambucaria|MM09772|658[0n]bp|Finland.Aland Islands|BOLD:AAB4472
Agriopis marginaria|TLMF Lep 08857|658[0n]bp|Austria.Vorarlberg|BOLD:AAC0355
Agriopis marginaria|MM07217|658[0n]bp|Finland|BOLD:AAC0355
Agriopis marginaria|MM17858|658[0n]bp|Finland|BOLD:AAC0355
Peribatodes secundaria|MM06806|658[0n]bp|Finland.Aland Islands|BOLD:AAC2923
Peribatodes secundaria|TLMF Lep 08551|658[0n]bp|Austria.Vorarlberg|BOLD:AAC2923
Peribatodes secundaria|MM06807|658[0n]bp|Finland.Aland Islands|BOLD:AAC2923
Peribatodes secundaria|MM09579|658[0n]bp|Finland.Aland Islands|BOLD:AAC2923
Cepphis advenaria|MM11594|658[0n]bp|Finland|BOLD:AAC7380
Cepphis advenaria|MM01324|658[0n]bp|Finland.South Karelia|BOLD:AAC7380
Cepphis advenaria|MM01335|658[0n]bp|Finland.South Karelia|BOLD:AAC7380
Cepphis advenaria|MM01323|658[0n]bp|Finland.South Karelia|BOLD:AAC7380
Cepphis advenaria|TLMF Lep 08152|658[0n]bp|Austria.Vorarlberg|BOLD:AAC7380
Phigalia pilosaria|MM07736|658[0n]bp|Finland|BOLD:AAD0877
Phigalia pilosaria|MM04605|609[0n]bp|Finland.Finland Proper|BOLD:AAD0877
Phigalia pilosaria|TLMF Lep 09775|658[0n]bp|Austria.Vorarlberg|BOLD:AAD0877
Phigalia pilosaria|MM01482|658[0n]bp|Finland.South Karelia|BOLD:AAD0877
Ematurga atomaria|TLMF Lep 10012|658[0n]bp|Austria.Vorarlberg|BOLD:AAC1877
Ematurga atomaria|MM00501|658[0n]bp|Finland.Northern Ostrobothnia|BOLD:AAC1877
Ematurga atomaria|MM18510|658[0n]bp|Finland|BOLD:AAC1877
Ematurga atomaria|MM00562|658[0n]bp|Finland.Northern Ostrobothnia|BOLD:AAC1877
Hypomecis punctinalis|MM01281|658[0n]bp|Finland.South Karelia|BOLD:AAB1058
Hypomecis punctinalis|MM01280|658[0n]bp|Finland.South Karelia|BOLD:AAB1058
Hypomecis punctinalis|MM08369|658[0n]bp|Finland|BOLD:AAB1058
Hypomecis punctinalis|TLMF Lep 07886|658[0n]bp|Austria.Vorarlberg|BOLD:ACA2461
Chiasmia clathrata|BIOUG04490-F09|633[0n]bp|Finland.Northern Ostrobothnia|BOLD:AAB0547
Chiasmia clathrata|TLMF Lep 07842|658[0n]bp|Austria.Vorarlberg|BOLD:AAB0547
Chiasmia clathrata|MM01278|658[0n]bp|Finland.South Karelia|BOLD:AAB0547
[illegible]

Chiasmia clathrata|[illegible]|Finland.Northern Ostrobothnia|BOLD:AAB0547
Chiasmia clathrata|TLMF Lep 07842|658[0n]bp|Austria.Vorarlberg|BOLD:AAB0547
Chiasmia clathrata|MM01278|658[0n]bp|Finland.South Karelia|BOLD:AAB0547
Chiasmia clathrata|MM01279|658[0n]bp|Finland.South Karelia|BOLD:AAB0547
Chiasmia clathrata|MM13945|658[0n]bp|Finland|BOLD:AAB0547
Pseudopanthera macularia|TLMF Lep 04678|658[0n]bp|Austria.Vorarlberg|BOLD:AAC7055
Pseudopanthera macularia|MM18708|658[0n]bp|Finland|BOLD:AAC7055
Pseudopanthera macularia|MM15835|658[0n]bp|Finland|BOLD:AAC7055
Pseudopanthera macularia|MM08577|658[0n]bp|Finland|BOLD:AAC7055
Elophos vittaria|MM04136|658[0n]bp|Finland.Lapland|BOLD:AAD3009
Elophos vittaria|MM14085|658[0n]bp|Finland|BOLD:AAD3009
Elophos vittaria|MM02824|658[0n]bp|Finland.Northern Ostrobothnia|BOLD:AAD3009
Elophos vittaria|TLMF Lep 09996|658[0n]bp|Austria.Vorarlberg|BOLD:AAD3010
Ennomos alniaria|MM12770|658[0n]bp|Finland|BOLD:AAB2314
Ennomos alniaria|MM04510|658[0n]bp|Finland.Finland Proper|BOLD:AAB2314
Ennomos alniaria|TLMF Lep 08531|658[0n]bp|Austria.Vorarlberg|BOLD:AAB2314
Ennomos alniaria|MM07824|641[0n]bp|Finland|BOLD:AAB2314
Hypomecis roboraria|MM01470|658[0n]bp|Finland.South Karelia|BOLD:AAC9905
Hypomecis roboraria|TLMF Lep 08165|658[0n]bp|Austria.Vorarlberg|BOLD:AAC9905
Hypomecis roboraria|MM09834|658[0n]bp|Finland.Aland Islands|BOLD:AAC9905
Hypomecis roboraria|MM05135|658[0n]bp|Finland.Finland Proper|BOLD:AAC9905
Lycia hirtaria|MM02807|658[0n]bp|Finland.Northern Ostrobothnia|BOLD:ACF3346
Lycia hirtaria|MM01484|658[0n]bp|Finland.South Karelia|BOLD:ACF3346
Lycia hirtaria|MM01483|658[0n]bp|Finland.South Karelia|BOLD:ACF3346
Lycia hirtaria|TLMF Lep 09777|658[0n]bp|Austria.Vorarlberg|BOLD:ACF3346
Alcis repandata|TLMF Lep 08149|658[0n]bp|Austria.Vorarlberg|BOLD:AAA8484
Alcis repandata|TLMF Lep 07564|658[0n]bp|Austria.Vorarlberg|BOLD:AAA8484
Alcis repandata|MM01516|658[0n]bp|Finland.South Karelia|BOLD:AAA8484
Alcis repandata|MM01517|658[0n]bp|Finland.South Karelia|BOLD:AAA8484
Alcis repandata|MM02817|658[0n]bp|Finland.Northern Ostrobothnia|BOLD:AAA8484
Arichanna melanaria|MM01351|658[0n]bp|Finland.South Karelia|BOLD:AAC8717
Arichanna melanaria|MM08115|658[0n]bp|Finland|BOLD:AAC8717
Arichanna melanaria|TLMF Lep 02877|658[0n]bp|Austria.Vorarlberg|BOLD:AAC8717
Arichanna melanaria|MM01352|658[0n]bp|Finland.South Karelia|BOLD:AAC8717
Bupalus piniaria|MM05427|658[0n]bp|Finland.Northern Ostrobothnia|BOLD:AAC7293
Bupalus piniaria|TLMF Lep 07839|658[0n]bp|Austria.Vorarlberg|BOLD:AAC7293
Bupalus piniaria|MM12778|658[0n]bp|Finland|BOLD:AAC7293
Bupalus piniaria|MM09470|658[0n]bp|Finland.Aland Islands|BOLD:AAC7293
Erannis defoliaria|MM18515|658[0n]bp|Finland|BOLD:AAB4418
Erannis defoliaria|MM14438|658[0n]bp|Finland|BOLD:AAB4418
Erannis defoliaria|MM12237|658[0n]bp|Finland|BOLD:AAB4418
Erannis defoliaria|TLMF Lep 06800|658[0n]bp|Austria.Vorarlberg|BOLD:AAB4418
Siona lineata|MM01273|658[0n]bp|Finland.South Karelia|BOLD:AAC1903
Siona lineata|MM01272|658[0n]bp|Finland.South Karelia|BOLD:AAC1903
Siona lineata|MM04731|658[0n]bp|Finland.Finland Proper|BOLD:AAC1903
Siona lineata|TLMF Lep 09876|658[0n]bp|Austria.Vorarlberg|BOLD:AAC1903
Cosmotriche lobulina|EA1.0E6|658[0n]bp|Finland|BOLD:AAE4008
Cosmotriche lobulina|EA1.08A|658[0n]bp|Finland|BOLD:AAE4008
Cosmotriche lobulina|EA1.0EC|658[0n]bp|Finland|BOLD:AAE4008
Cosmotriche lobulina|EA1.08V|658[0n]bp|Finland|BOLD:AAE4008
Cosmotriche lobulina|EA1.099|658[0n]bp|Finland|BOLD:AAE4008
Cosmotriche lobulina|EA1.08M|658[0n]bp|Finland|BOLD:AAE4008
Cosmotriche lobulina|EA1.08K|658[0n]bp|Finland|BOLD:AAE4008
Cosmotriche lobulina|EA1.08H|658[0n]bp|Finland|BOLD:AAE4008
Cosmotriche lobulina|EA1.09I|658[0n]bp|Finland|BOLD:AAE4008
Cosmotriche lobulina|EA1.08T|658[0n]bp|Finland|BOLD:AAE4008
Cosmotriche lobulina|EA1.08Z|658[0n]bp|Finland|BOLD:AAE4008
Cosmotriche lobulina|EA1.09G|658[0n]bp|Finland|BOLD:AAE4008
Cosmotriche lobulina|EA1.090|658[0n]bp|Finland|BOLD:AAE4008
Cosmotriche lobulina|EA1.091|658[0n]bp|Finland|BOLD:AAE4008
Cosmotriche lobulina|EA1.092|658[0n]bp|Finland|BOLD:AAE4008
Cosmotriche lobulina|EA1.096|658[0n]bp|Finland|BOLD:AAE4008
Cosmotriche lobulina|EA1.098|658[0n]bp|Finland|BOLD:AAE4008
Cosmotriche lobulina|EA1.0E9|658[0n]bp|Finland|BOLD:AAE4008
Cosmotriche lobulina|EA1.08O|658[0n]bp|Finland|BOLD:AAE4008
Cosmotriche lobulina|EA1.094|658[0n]bp|Finland|BOLD:AAE4008
Cosmotriche lobulina|EA1.095|658[0n]bp|Finland|BOLD:AAE4008
Cosmotriche lobulina|EA1.09E|658[0n]bp|Finland|BOLD:AAE4008
Cosmotriche lobulina|EA1.09A|658[0n]bp|Finland|BOLD:AAE4008
Cosmotriche lobulina|EA1.0E3|658[1n]bp|Finland|BOLD:AAE4008
Cosmotriche lobulina|EA1.089|658[0n]bp|Finland|BOLD:AAE4008
Cosmotriche lobulina|EA1.086|658[0n]bp|Finland|BOLD:AAE4008
Cosmotriche lobulina|TLMF Lep 06110|658[0n]bp|Austria.Vorarlberg|BOLD:AAE4008
Cosmotriche lobulina|TLMF Lep 03077|658[0n]bp|Austria.Vorarlberg|BOLD:AAE4008
Cosmotriche lobulina|TLMF Lep 03076|658[0n]bp|Austria.Vorarlberg|BOLD:AAE4008
Cosmotriche lobulina|EA1.08D|658[0n]bp|Finland|BOLD:AAE4008
Cosmotriche lobulina|EA1.08B|658[0n]bp|Finland|BOLD:AAE4008
Cosmotriche lobulina|EA1.09D|658[0n]bp|Finland|BOLD:AAE4008
Cosmotriche lobulina|EA1.0E8|658[0n]bp|Finland|BOLD:AAE4008
Cosmotriche lobulina|EA1.0E7|658[0n]bp|Finland|BOLD:AAE4008
Cosmotriche lobulina|EA1.08P|658[0n]bp|Finland|BOLD:AAE4008
Cosmotriche lobulina|EA1.08N|658[0n]bp|Finland|BOLD:AAE4008
Cosmotriche lobulina|EA1.08G|658[0n]bp|Finland|BOLD:AAE4008
Cosmotriche lobulina|EA1.08F|658[0n]bp|Finland|BOLD:AAE4008
Cosmotriche lobulina|EA1.08C|658[0n]bp|Finland|BOLD:AAE4008
Cosmotriche lobulina|EA1.0ED|658[0n]bp|Finland|BOLD:AAE4008
Cosmotriche lobulina|EA1.084|658[0n]bp|Finland|BOLD:AAE4008
Cosmotriche lobulina|EA1.093|658[0n]bp|Finland|BOLD:AAE4008
Cosmotriche lobulina|EA1.09H|658[0n]bp|Finland|BOLD:AAE4008
Cosmotriche lobulina|EA1.09F|658[0n]bp|Finland|BOLD:AAE4008
Cosmotriche lobulina|EA1.08Y|658[0n]bp|Finland|BOLD:AAE4008
Cosmotriche lobulina|EA1.08X|658[0n]bp|Finland|BOLD:AAE4008
Cosmotriche lobulina|EA1.08W|658[0n]bp|Finland|BOLD:AAE4008
Cosmotriche lobulina|EA1.08R|658[0n]bp|Finland|BOLD:AAE4008
Cosmotriche lobulina|EA1.08Q|658[0n]bp|Finland|BOLD:AAE4008
Cosmotriche lobulina|EA1.0E5|658[0n]bp|Finland|BOLD:AAE4008
Cosmotriche lobulina|EA1.0E4|658[0n]bp|Finland|BOLD:AAE4008
Cosmotriche lobulina|MM18678|658[0n]bp|Finland|BOLD:AAE4008
Cosmotriche lobulina|MM07392|658[0n]bp|Finland|BOLD:AAE4008
Cosmotriche lobulina|EA1.085|658[0n]bp|Finland|BOLD:AAE4008
Euthrix potatoria|MM01057|658[0n]bp|Finland.South Karelia|BOLD:AAC1584
Euthrix potatoria|MM01056|658[0n]bp|Finland.South Karelia|BOLD:AAC1584
Euthrix potatoria|MM07310|658[0n]bp|Finland|BOLD:AAC1584

Euthrix potatoria|MM01057|658[0n]bp|Finland.South Karelia|BOLD:AAC1584
Euthrix potatoria|MM01056|658[0n]bp|Finland.South Karelia|BOLD:AAC1584
Euthrix potatoria|MM07310|658[0n]bp|Finland|BOLD:AAC1584
Euthrix potatoria|TLMF Lep 08528|658[0n]bp|Austria.Vorarlberg|BOLD:AAC1584
Gagitodes sagittata|TLMF Lep 09415|658[0n]bp|Austria.Vorarlberg|BOLD:AAD8985
Gagitodes sagittata|MM11603|658[0n]bp|Finland|BOLD:AAD8985
Gagitodes sagittata|MM06808|658[0n]bp|Finland.Aland Islands|BOLD:AAD8985
Gagitodes sagittata|MM06750|658[0n]bp|Finland.Aland Islands|BOLD:AAD8985
Lasiocampa quercus|MM00380|658[0n]bp|Finland.Northern Ostrobothnia|BOLD:AAC5660
Lasiocampa quercus|MM06634|658[0n]bp|Finland|BOLD:AAC5660
Lasiocampa quercus|MM12528|658[0n]bp|Finland|BOLD:AAC5660
Lasiocampa quercus|MM03714|670[1n]bp|Finland.Northern Ostrobothnia|BOLD:AAC5660
Lasiocampa quercus|MM14213|658[0n]bp|Finland.Aland Islands|BOLD:AAC5660
Lasiocampa quercus|MM10716|658[0n]bp|Finland|BOLD:AAC5660
Lasiocampa quercus|MM15183|658[0n]bp|Finland|BOLD:AAC5660
Lasiocampa quercus|MM11191|658[0n]bp|Finland|BOLD:AAC5660
Lasiocampa quercus|MM17998|658[0n]bp|Finland|BOLD:AAC5660
Lasiocampa quercus|MM06739|658[0n]bp|Finland.Aland Islands|BOLD:AAC5660
Lasiocampa quercus|MM10251|658[0n]bp|Finland|BOLD:AAC5660
Lasiocampa quercus|TLMF Lep 08503|658[0n]bp|Austria.Vorarlberg|BOLD:AAC5660
Hemithea aestivaria|MM06098|658[0n]bp|Finland|BOLD:AAA4522
Hemithea aestivaria|MM10875|637[0n]bp|Finland|BOLD:AAA4522
Hemithea aestivaria|TLMF Lep 08192|658[0n]bp|Austria.Vorarlberg|BOLD:AAA4522
Hemithea aestivaria|MM10874|658[0n]bp|Finland|BOLD:AAA4522
Lomaspilis marginata|TLMF Lep 04618|658[0n]bp|Austria.Vorarlberg|BOLD:AAB5300
Lomaspilis marginata|MM01247|670[0n]bp|Finland.South Karelia|BOLD:AAB5300
Lomaspilis marginata|MM01248|632[0n]bp|Finland.South Karelia|BOLD:AAB5300
Lomaspilis marginata|MM01249|658[0n]bp|Finland.South Karelia|BOLD:AAB5300
Lomaspilis marginata|MM00572|658[0n]bp|Finland.Northern Ostrobothnia|BOLD:AAB5300
Dendrolimus pini|MM08302|658[0n]bp|Finland|BOLD:AAB6845
Dendrolimus pini|MM12533|658[0n]bp|Finland|BOLD:AAB6845
Dendrolimus pini|TLMF Lep 06156|658[0n]bp|Austria.Vorarlberg|BOLD:AAB6845
Dendrolimus pini|MM01054|658[0n]bp|Finland.South Karelia|BOLD:AAB6845
Macrothylacia rubi|TLMF Lep 07824|658[0n]bp|Austria.Vorarlberg|BOLD:AAD0839
Macrothylacia rubi|MM01059|658[0n]bp|Finland.South Karelia|BOLD:AAD0839
Macrothylacia rubi|MM01060|658[0n]bp|Finland.South Karelia|BOLD:AAD0839
Macrothylacia rubi|MM10712|658[0n]bp|Finland|BOLD:AAD0839
Poecilocampa populi|TLMF Lep 06215|658[0n]bp|Austria.Vorarlberg|BOLD:AAC8994
Poecilocampa populi|MM02696|670[0n]bp|Finland.South Karelia|BOLD:AAC8994
Poecilocampa populi|MM08339|658[0n]bp|Finland|BOLD:AAC8994
Poecilocampa populi|MM02715|658[0n]bp|Finland.Northern Ostrobothnia|BOLD:AAC8994
Poecilocampa populi|MM08223|658[0n]bp|Finland|BOLD:AAC8994
Trichiura crataegi|TLMF Lep 08491|658[0n]bp|Austria.Vorarlberg|BOLD:AAB4489
Trichiura crataegi|MM12574|658[0n]bp|Finland|BOLD:AAB4489
Trichiura crataegi|MM01051|658[0n]bp|Finland.South Karelia|BOLD:AAB4489
Trichiura crataegi|MM13870|658[0n]bp|Finland.Lapland|BOLD:AAB4489
Trichiura crataegi|MM07389|658[0n]bp|Finland|BOLD:AAB4489
Trichiura crataegi|MM08292|658[0n]bp|Finland|BOLD:AAB4489
Trichiura crataegi|MM02797|658[0n]bp|Finland.Northern Ostrobothnia|BOLD:AAB4489
Trichiura crataegi|MM00836|658[0n]bp|Finland.Northern Ostrobothnia|BOLD:AAB4489
Trichiura crataegi|MM13871|658[0n]bp|Finland.Lapland|BOLD:AAB4489
Trichiura crataegi|MM01052|658[0n]bp|Finland.South Karelia|BOLD:AAB4489
Trichiura crataegi|MM03616|651[0n]bp|Finland.Uusimaa|BOLD:AAB4489
Trichiura crataegi|MM08104|636[0n]bp|Finland|BOLD:AAB4489
Scopula ornata|TLMF Lep 08148|658[0n]bp|Austria.Vorarlberg|BOLD:AAB1550
Scopula ornata|MM07059|646[0n]bp|Finland|BOLD:AAB1550
Scopula ornata|MM10461|658[0n]bp|Finland|BOLD:AAB1550
Scopula incanata|TLMF Lep 08498|658[0n]bp|Austria.Vorarlberg|BOLD:AAC9956
Scopula incanata|MM18413|658[0n]bp|Finland|BOLD:AAC9956
Scopula incanata|MM12852|658[0n]bp|Finland|BOLD:AAC9956
Scopula incanata|MM04558|658[0n]bp|Finland.Finland Proper|BOLD:AAC9956
Scopula floslactata|MM01339|654[0n]bp|Finland.South Karelia|BOLD:AAC5048
Scopula floslactata|MM01338|658[0n]bp|Finland.South Karelia|BOLD:AAC5048
Scopula floslactata|TLMF Lep 07974|658[0n]bp|Austria.Vorarlberg|BOLD:AAC5048
Scopula floslactata|BIOUG04490-E11|614[0n]bp|Finland.Northern Ostrobothnia|BOLD:AAC5048
Scopula floslactata|MM02862|658[0n]bp|Finland.Northern Ostrobothnia|BOLD:AAC5048
Scopula ternata|MM08463|658[0n]bp|Finland|BOLD:AAA5384
Scopula ternata|TLMF Lep 00314|658[0n]bp|Austria.Vorarlberg|BOLD:AAA5384
Scopula ternata|MM01445|658[0n]bp|Finland.South Karelia|BOLD:AAA5384
Scopula ternata|MM01444|658[0n]bp|Finland.South Karelia|BOLD:AAA5384
Scopula virgulata|TLMF Lep 08128|658[0n]bp|Austria.Vorarlberg|BOLD:AAP7443
Scopula virgulata|MM22893|658[0n]bp|Finland|BOLD:AAP7443
Scopula virgulata|MM18696|658[0n]bp|Finland|BOLD:AAP7443
Scopula virgulata|MM17429|658[0n]bp|Finland|BOLD:AAP7443
Scopula virgulata|MM17430|658[0n]bp|Finland|BOLD:AAP7443
Scopula virgulata|MM18695|658[0n]bp|Finland|BOLD:AAP7443
Aplocera praeformata|MM01468|658[0n]bp|Finland.South Karelia|BOLD:AAC3875
Aplocera praeformata|MM04928|645[0n]bp|Finland.Finland Proper|BOLD:AAC3875
Aplocera praeformata|MM12783|634[0n]bp|Finland|BOLD:AAC3875
Aplocera praeformata|TLMF Lep 08495|658[0n]bp|Austria.Vorarlberg|BOLD:AAC3875
Carsia sororiata|TLMF Lep 12550|658[0n]bp|Austria.Vorarlberg|BOLD:AAC1640
Carsia sororiata|MM02919|658[0n]bp|Finland.Northern Ostrobothnia|BOLD:AAC1640
Carsia sororiata|MM08117|658[0n]bp|Finland|BOLD:AAC1640
Carsia sororiata|MM01438|658[0n]bp|Finland.South Karelia|BOLD:AAC1640
Catarhoe cuculata|MM01358|625[0n]bp|Finland.South Karelia|BOLD:AAC2047
Catarhoe cuculata|MM01359|631[0n]bp|Finland.South Karelia|BOLD:AAC2047
Catarhoe cuculata|TLMF Lep 04641|658[0n]bp|Austria.Vorarlberg|BOLD:AAC2047
Catarhoe cuculata|TLMF Lep 08114|658[0n]bp|Austria.Vorarlberg|BOLD:AAC2047
Catarhoe cuculata|MM06718|658[0n]bp|Finland.Aland Islands|BOLD:AAC2047
Odezia atrata|MM03239|658[0n]bp|Finland.Northern Ostrobothnia|BOLD:AAD5243
Odezia atrata|MM06562|658[0n]bp|Finland|BOLD:AAD5243
Odezia atrata|MM01378|646[0n]bp|Finland.South Karelia|BOLD:AAD5243
Odezia atrata|TLMF Lep 09514|658[0n]bp|Austria.Vorarlberg|BOLD:ACG1959
Odezia atrata|TLMF Lep 09840|658[0n]bp|Austria.Vorarlberg|BOLD:AAD5242
Ecliptopera capitata|MM18426|658[1n]bp|Finland|BOLD:AAC9011
Ecliptopera capitata|TLMF Lep 07889|658[0n]bp|Austria.Vorarlberg|BOLD:AAC9011
Ecliptopera capitata|MM10468|658[0n]bp|Finland|BOLD:AAC9011
Ecliptopera capitata|MM01374|633[0n]bp|Finland.South Karelia|BOLD:AAC9011
Ecliptopera silaceata|MM08169|632[0n]bp|Finland|BOLD:AAA2586
Ecliptopera silaceata|MM01321|658[0n]bp|Finland.South Karelia|BOLD:AAA2586
Ecliptopera silaceata|TLMF Lep 07849|658[0n]bp|Austria.Vorarlberg|BOLD:AAA2586
Ecliptopera silaceata|MM01322|658[0n]bp|Finland.South Karelia|BOLD:AAA2586
Chloroclystis v-ata|MM10480|658[0n]bp|Finland|BOLD:AAB1832

[illegible]
Ecliptopera silaceata|TLMF Lep 07849|658[0n]bp|Austria.Vorarlberg|BOLD:AAA2586
Ecliptopera silaceata|MM01322|658[0n]bp|Finland.South Karelia|BOLD:AAA2586
Chloroclystis v-ata|MM10480|658[0n]bp|Finland|BOLD:AAB1832
Chloroclystis v-ata|MM10482|658[0n]bp|Finland|BOLD:AAB1832
Chloroclystis v-ata|MM17291|658[0n]bp|Finland|BOLD:AAB1832
Chloroclystis v-ata|MM10478|658[0n]bp|Finland|BOLD:AAB1832
Chloroclystis v-ata|TLMF Lep 07877|658[0n]bp|Austria.Vorarlberg|BOLD:AAB1832
Chloroclystis v-ata|MM17292|658[0n]bp|Finland|BOLD:AAB1832
Chloroclystis v-ata|MM10481|658[0n]bp|Finland|BOLD:AAB1832
Chloroclystis v-ata|MM17241|658[0n]bp|Finland|BOLD:AAB1832
Chloroclystis v-ata|MM10479|658[0n]bp|Finland|BOLD:AAB1832
Gymnoscelis rufifasciata|MM01823|658[0n]bp|Finland.South Karelia|BOLD:AAA7404
Gymnoscelis rufifasciata|MM01824|658[0n]bp|Finland.South Karelia|BOLD:AAA7404
Gymnoscelis rufifasciata|TLMF Lep 08164|658[0n]bp|Austria.Vorarlberg|BOLD:AAA7404
Gymnoscelis rufifasciata|MM12960|658[0n]bp|Finland|BOLD:AAA7404
Eulithis populata|TLMF Lep 06165|658[0n]bp|Austria.Vorarlberg|BOLD:ABZ1837
Eulithis populata|MM08084|658[0n]bp|Finland|BOLD:ABZ1837
Eulithis populata|TLMF Lep 06166|658[0n]bp|Austria.Vorarlberg|BOLD:ABZ1837
Eulithis populata|MM05173|634[0n]bp|Finland.Finland Proper|BOLD:ABZ1837
Eulithis populata|MM00797|658[0n]bp|Finland.Northern Ostrobothnia|BOLD:ABZ1837
Gandaritis pyraliata|MM03583|658[0n]bp|Finland.Uusimaa|BOLD:AAB5983
Gandaritis pyraliata|MM18427|658[0n]bp|Finland|BOLD:AAB5983
Gandaritis pyraliata|TLMF Lep 08163|658[0n]bp|Austria.Vorarlberg|BOLD:AAB5983
Gandaritis pyraliata|MM12812|658[0n]bp|Finland|BOLD:AAB5983
Lobophora halterata|TLMF Lep 04669|658[0n]bp|Austria.Vorarlberg|BOLD:AAB7562
Lobophora halterata|MM10101|658[0n]bp|Finland|BOLD:AAB7562
Lobophora halterata|TLMF Lep 08812|658[0n]bp|Austria.Vorarlberg|BOLD:AAB7562
Lobophora halterata|MM01274|658[0n]bp|Finland.South Karelia|BOLD:AAB7562
Lobophora halterata|MM01275|658[0n]bp|Finland.South Karelia|BOLD:AAB7562
Pterapherapteryx sexalata|MM01766|658[0n]bp|Finland.South Karelia|BOLD:AAD0485
Pterapherapteryx sexalata|MM01511|658[0n]bp|Finland.South Karelia|BOLD:AAD0485
Pterapherapteryx sexalata|MM04577|571[0n]bp|Finland.Finland Proper|BOLD:AAD0485
Pterapherapteryx sexalata|MM01436|658[0n]bp|Finland.South Karelia|BOLD:AAD0485
Pterapherapteryx sexalata|TLMF Lep 08803|658[0n]bp|Austria.Vorarlberg|BOLD:AAD0485
Acasis viretata|MM03876|658[0n]bp|Finland.Aland Islands|BOLD:AAC2783
Acasis viretata|MM10485|658[0n]bp|Finland|BOLD:AAC2783
Acasis viretata|MM04670|647[0n]bp|Finland.Finland Proper|BOLD:AAC2783
Acasis viretata|TLMF Lep 07885|658[0n]bp|Austria.Vorarlberg|BOLD:AAC2783
Trichopteryx carpinata|MM01142|658[0n]bp|Finland.South Karelia|BOLD:AAC7472
Trichopteryx carpinata|TLMF Lep 04668|658[0n]bp|Austria.Vorarlberg|BOLD:AAC7472
Trichopteryx carpinata|MM04633|670[0n]bp|Finland.Finland Proper|BOLD:AAC7472
Trichopteryx carpinata|MM01143|658[0n]bp|Finland.South Karelia|BOLD:AAC7472
Trichopteryx carpinata|MM08387|652[0n]bp|Finland|BOLD:AAC7472
Trichopteryx polycommata|TLMF Lep 08859|658[0n]bp|Austria.Vorarlberg|BOLD:AAC5988
Trichopteryx polycommata|MM00448|658[0n]bp|Finland.South Karelia|BOLD:AAC5988
Trichopteryx polycommata|MM07856|658[0n]bp|Finland|BOLD:AAC5988
Trichopteryx polycommata|MM04024|658[0n]bp|Finland.South Karelia|BOLD:AAC5988
Hydria cervinalis|MM04035|646[0n]bp|Finland.Uusimaa|BOLD:AAC1414
Hydria cervinalis|TLMF Lep 07884|658[0n]bp|Austria.Vorarlberg|BOLD:AAC1414
Hydria cervinalis|MM18465|658[0n]bp|Finland|BOLD:AAC1414
Hydria cervinalis|MM04034|658[0n]bp|Finland.Uusimaa|BOLD:AAC1414
Hydria undulata|MM01384|655[0n]bp|Finland.South Karelia|BOLD:AAA9056
Hydria undulata|TLMF Lep 07563|658[0n]bp|Austria.Vorarlberg|BOLD:AAA9056
Hydria undulata|MM02856|658[0n]bp|Finland.Northern Ostrobothnia|BOLD:AAA9056
Hydria undulata|MM01462|658[0n]bp|Finland.South Karelia|BOLD:AAA9056
Hydria undulata|MM01461|637[0n]bp|Finland.South Karelia|BOLD:AAA9056
Rheumaptera subhastata|MM10312|658[0n]bp|Finland|BOLD:AAA5436
Rheumaptera subhastata|MM18463|658[0n]bp|Finland|BOLD:AAA5436
Rheumaptera subhastata|MM18464|658[0n]bp|Finland|BOLD:AAA5436
Rheumaptera hastata|TLMF Lep 09874|635[0n]bp|Austria.Vorarlberg|BOLD:AAA5435
Rheumaptera hastata|MM00580|658[0n]bp|Finland.Northern Ostrobothnia|BOLD:AAA5435
Rheumaptera hastata|MM10108|658[0n]bp|Finland|BOLD:AAA5435
Rheumaptera hastata|MM18452|658[0n]bp|Finland|BOLD:AAA5435
Rheumaptera|BIOUG04490-F08|632[0n]bp|Finland.Northern Ostrobothnia|BOLD:AAA5435
Rheumaptera hastata|MM06321|609[0n]bp|Finland|BOLD:AAA5435
Rheumaptera hastata|MM18450|658[0n]bp|Finland|BOLD:AAA5435
Rheumaptera hastata|MM18451|658[0n]bp|Finland|BOLD:AAA5435
Rheumaptera subhastata|MM18462|618[0n]bp|Finland|BOLD:AAA5435
Rheumaptera subhastata|MM06308|658[0n]bp|Finland|BOLD:AAA5435
Rheumaptera subhastata|MM17987|658[0n]bp|Finland|BOLD:AAA5435
Rheumaptera subhastata|MM18460|658[2n]bp|Finland|BOLD:AAA5435
Rheumaptera subhastata|MM18454|658[0n]bp|Finland|BOLD:AAA5435
Rheumaptera subhastata|MM18456|658[0n]bp|Finland|BOLD:AAA5435
Rheumaptera subhastata|TLMF Lep 09873|658[0n]bp|Austria.Vorarlberg|BOLD:AAA5435
Rheumaptera subhastata|MM06292|658[0n]bp|Finland|BOLD:AAA5435
Rheumaptera subhastata|MM18459|581[0n]bp|Finland|BOLD:AAA5435
Rheumaptera subhastata|MM18457|598[0n]bp|Finland|BOLD:AAA5435
Rheumaptera subhastata|MM18455|658[0n]bp|Finland|BOLD:AAA5435
Rheumaptera subhastata|MM17986|658[0n]bp|Finland|BOLD:AAA5435
Philereme transversata|MM18449|658[0n]bp|Finland|BOLD:AAC5104
Philereme transversata|TLMF Lep 08193|658[0n]bp|Austria.Vorarlberg|BOLD:AAC5104
Philereme transversata|MM10411|658[0n]bp|Finland.Aland Islands|BOLD:AAC5104
Philereme vetulata|TLMF Lep 08860|658[0n]bp|Austria.Vorarlberg|BOLD:AAD2984
Philereme vetulata|MM09781|658[0n]bp|Finland.Aland Islands|BOLD:AAD2984
Philereme vetulata|MM09782|658[0n]bp|Finland.Aland Islands|BOLD:AAD2984
Philereme vetulata|MM18448|658[0n]bp|Finland|BOLD:AAD2984
Triphosa dubitata|MM18466|650[0n]bp|Finland|BOLD:AAD3995
Triphosa dubitata|MM15815|658[0n]bp|Finland|BOLD:AAD3995
Triphosa dubitata|MM04218|658[0n]bp|Finland.Aland Islands|BOLD:AAD3995
Triphosa dubitata|TLMF Lep 06121|658[0n]bp|Austria.Vorarlberg|BOLD:AAD3995
Cosmorhoe ocellata|TLMF Lep 07975|658[0n]bp|Austria.Vorarlberg|BOLD:AAB4250
Cosmorhoe ocellata|MM11590|505[0n]bp|Finland|BOLD:AAB4250
Cosmorhoe ocellata|MM01250|658[0n]bp|Finland.South Karelia|BOLD:AAB4250
Cosmorhoe ocellata|MM01251|658[0n]bp|Finland.South Karelia|BOLD:AAB4250
Pennithera firmata|TLMF Lep 08765|658[0n]bp|Austria.Vorarlberg|BOLD:AAC6743
Pennithera firmata|MM15807|658[0n]bp|Finland|BOLD:AAC6743
Pennithera firmata|MM04805|658[0n]bp|Finland.Finland Proper|BOLD:AAC6743
Pennithera firmata|MM12819|658[0n]bp|Finland|BOLD:AAC6743
Pennithera firmata|MM15808|658[0n]bp|Finland.Aland Islands|BOLD:AAC6743
Eustroma reticulata|TLMF Lep 04614|658[0n]bp|Austria.Vorarlberg|BOLD:AAC8101
Eustroma reticulata|MM03537|658[0n]bp|Finland.Uusimaa|BOLD:AAC8101
Eustroma reticulata|MM03516|647[0n]bp|Finland.Kymenlaakso|BOLD:AAC8101
[illegible]

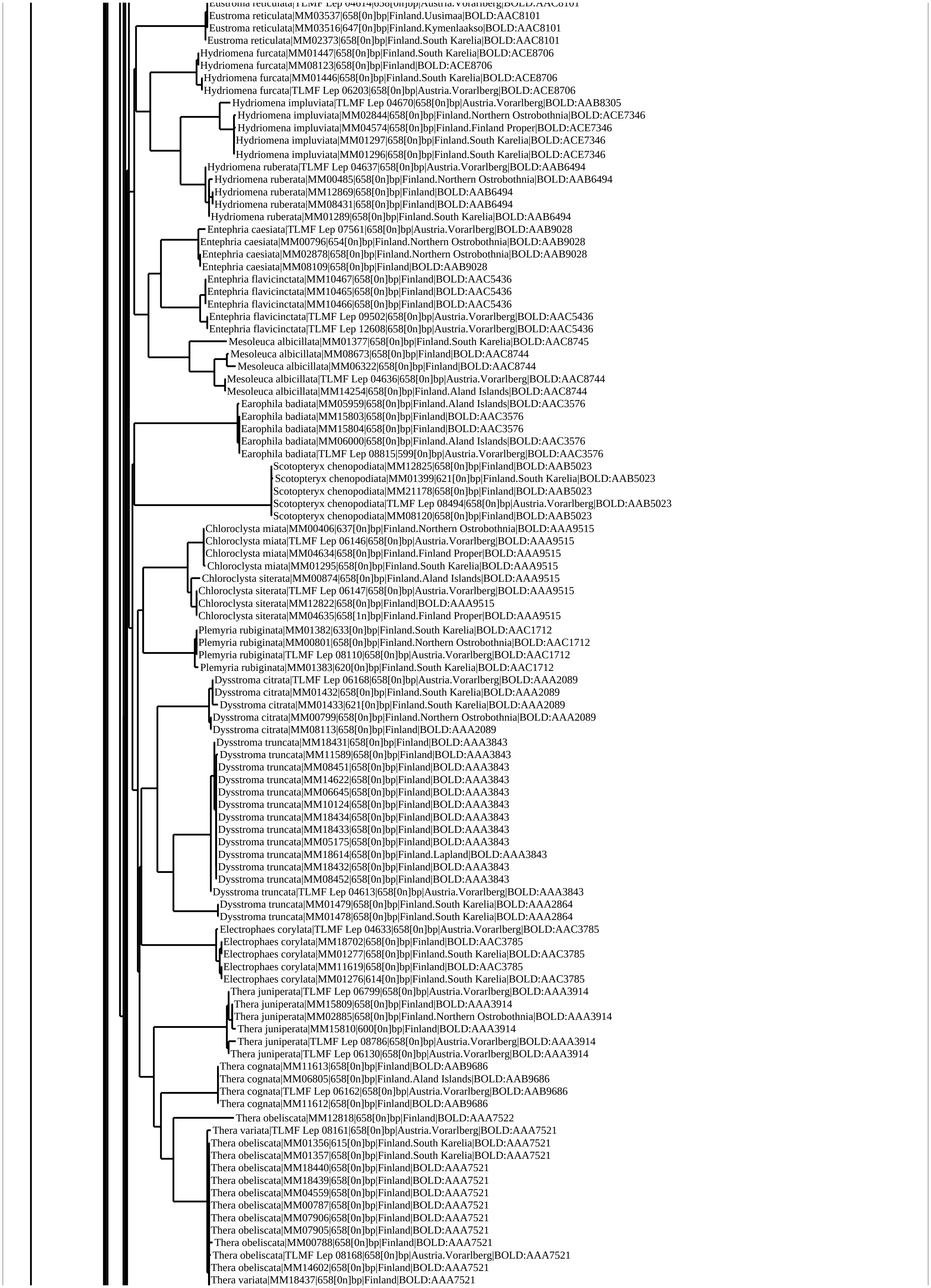
Eustroma reticulata|TLMF Lep 04614|658[0n]bp|Austria.Vorarlberg|BOLD:AAC8101
Eustroma reticulata|MM03537|658[0n]bp|Finland.Uusimaa|BOLD:AAC8101
Eustroma reticulata|MM03516|647[0n]bp|Finland.Kymenlaakso|BOLD:AAC8101
Eustroma reticulata|MM02373|658[0n]bp|Finland.South Karelia|BOLD:AAC8101
Hydriomena furcata|MM01447|658[0n]bp|Finland.South Karelia|BOLD:ACE8706
Hydriomena furcata|MM08123|658[0n]bp|Finland|BOLD:ACE8706
Hydriomena furcata|MM01446|658[0n]bp|Finland.South Karelia|BOLD:ACE8706
Hydriomena furcata|TLMF Lep 06203|658[0n]bp|Austria.Vorarlberg|BOLD:ACE8706
Hydriomena impluviata|TLMF Lep 04670|658[0n]bp|Austria.Vorarlberg|BOLD:AAB8305
Hydriomena impluviata|MM02844|658[0n]bp|Finland.Northern Ostrobothnia|BOLD:ACE7346
Hydriomena impluviata|MM04574|658[0n]bp|Finland.Finland Proper|BOLD:ACE7346
Hydriomena impluviata|MM01297|658[0n]bp|Finland.South Karelia|BOLD:ACE7346
Hydriomena impluviata|MM01296|658[0n]bp|Finland.South Karelia|BOLD:ACE7346
Hydriomena ruberata|TLMF Lep 04637|658[0n]bp|Austria.Vorarlberg|BOLD:AAB6494
Hydriomena ruberata|MM00485|658[0n]bp|Finland.Northern Ostrobothnia|BOLD:AAB6494
Hydriomena ruberata|MM12869|658[0n]bp|Finland|BOLD:AAB6494
Hydriomena ruberata|MM08431|658[0n]bp|Finland|BOLD:AAB6494
Hydriomena ruberata|MM01289|658[0n]bp|Finland.South Karelia|BOLD:AAB6494
Entephria caesiata|TLMF Lep 07561|658[0n]bp|Austria.Vorarlberg|BOLD:AAB9028
Entephria caesiata|MM00796|654[0n]bp|Finland.Northern Ostrobothnia|BOLD:AAB9028
Entephria caesiata|MM02878|658[0n]bp|Finland.Northern Ostrobothnia|BOLD:AAB9028
Entephria caesiata|MM08109|658[0n]bp|Finland|BOLD:AAB9028
Entephria flavicinctata|MM10467|658[0n]bp|Finland|BOLD:AAC5436
Entephria flavicinctata|MM10465|658[0n]bp|Finland|BOLD:AAC5436
Entephria flavicinctata|MM10466|658[0n]bp|Finland|BOLD:AAC5436
Entephria flavicinctata|TLMF Lep 09502|658[0n]bp|Austria.Vorarlberg|BOLD:AAC5436
Entephria flavicinctata|TLMF Lep 12608|658[0n]bp|Austria.Vorarlberg|BOLD:AAC5436
Mesoleuca albicillata|MM01377|658[0n]bp|Finland.South Karelia|BOLD:AAC8745
Mesoleuca albicillata|MM08673|658[0n]bp|Finland|BOLD:AAC8744
Mesoleuca albicillata|MM06322|658[0n]bp|Finland|BOLD:AAC8744
Mesoleuca albicillata|TLMF Lep 04636|658[0n]bp|Austria.Vorarlberg|BOLD:AAC8744
Mesoleuca albicillata|MM14254|658[0n]bp|Finland.Aland Islands|BOLD:AAC8744
Earophila badiata|MM05959|658[0n]bp|Finland.Aland Islands|BOLD:AAC3576
Earophila badiata|MM15803|658[0n]bp|Finland|BOLD:AAC3576
Earophila badiata|MM15804|658[0n]bp|Finland|BOLD:AAC3576
Earophila badiata|MM06000|658[0n]bp|Finland.Aland Islands|BOLD:AAC3576
Earophila badiata|TLMF Lep 08815|599[0n]bp|Austria.Vorarlberg|BOLD:AAC3576
Scotopteryx chenopodiata|MM12825|658[0n]bp|Finland|BOLD:AAB5023
Scotopteryx chenopodiata|MM01399|621[0n]bp|Finland.South Karelia|BOLD:AAB5023
Scotopteryx chenopodiata|MM21178|658[0n]bp|Finland|BOLD:AAB5023
Scotopteryx chenopodiata|TLMF Lep 08494|658[0n]bp|Austria.Vorarlberg|BOLD:AAB5023
Scotopteryx chenopodiata|MM08120|658[0n]bp|Finland|BOLD:AAB5023
Chloroclysta miata|MM00406|637[0n]bp|Finland.Northern Ostrobothnia|BOLD:AAA9515
Chloroclysta miata|TLMF Lep 06146|658[0n]bp|Austria.Vorarlberg|BOLD:AAA9515
Chloroclysta miata|MM04634|658[0n]bp|Finland.Finland Proper|BOLD:AAA9515
Chloroclysta miata|MM01295|658[0n]bp|Finland.South Karelia|BOLD:AAA9515
Chloroclysta siterata|MM00874|658[0n]bp|Finland.Aland Islands|BOLD:AAA9515
Chloroclysta siterata|TLMF Lep 06147|658[0n]bp|Austria.Vorarlberg|BOLD:AAA9515
Chloroclysta siterata|MM12822|658[0n]bp|Finland|BOLD:AAA9515
Chloroclysta siterata|MM04635|658[1n]bp|Finland.Finland Proper|BOLD:AAA9515
Plemyria rubiginata|MM01382|633[0n]bp|Finland.South Karelia|BOLD:AAC1712
Plemyria rubiginata|MM00801|658[0n]bp|Finland.Northern Ostrobothnia|BOLD:AAC1712
Plemyria rubiginata|TLMF Lep 08110|658[0n]bp|Austria.Vorarlberg|BOLD:AAC1712
Plemyria rubiginata|MM01383|620[0n]bp|Finland.South Karelia|BOLD:AAC1712
Dysstroma citrata|TLMF Lep 06168|658[0n]bp|Austria.Vorarlberg|BOLD:AAA2089
Dysstroma citrata|MM01432|658[0n]bp|Finland.South Karelia|BOLD:AAA2089
Dysstroma citrata|MM01433|621[0n]bp|Finland.South Karelia|BOLD:AAA2089
Dysstroma citrata|MM00799|658[0n]bp|Finland.Northern Ostrobothnia|BOLD:AAA2089
Dysstroma citrata|MM08113|658[0n]bp|Finland|BOLD:AAA2089
Dysstroma truncata|MM18431|658[0n]bp|Finland|BOLD:AAA3843
Dysstroma truncata|MM11589|658[0n]bp|Finland|BOLD:AAA3843
Dysstroma truncata|MM08451|658[0n]bp|Finland|BOLD:AAA3843
Dysstroma truncata|MM14622|658[0n]bp|Finland|BOLD:AAA3843
Dysstroma truncata|MM06645|658[0n]bp|Finland|BOLD:AAA3843
Dysstroma truncata|MM10124|658[0n]bp|Finland|BOLD:AAA3843
Dysstroma truncata|MM18434|658[0n]bp|Finland|BOLD:AAA3843
Dysstroma truncata|MM18433|658[0n]bp|Finland|BOLD:AAA3843
Dysstroma truncata|MM05175|658[0n]bp|Finland|BOLD:AAA3843
Dysstroma truncata|MM18614|658[0n]bp|Finland.Lapland|BOLD:AAA3843
Dysstroma truncata|MM18432|658[0n]bp|Finland|BOLD:AAA3843
Dysstroma truncata|MM08452|658[0n]bp|Finland|BOLD:AAA3843
Dysstroma truncata|TLMF Lep 04613|658[0n]bp|Austria.Vorarlberg|BOLD:AAA3843
Dysstroma truncata|MM01479|658[0n]bp|Finland.South Karelia|BOLD:AAA2864
Dysstroma truncata|MM01478|658[0n]bp|Finland.South Karelia|BOLD:AAA2864
Electrophaes corylata|TLMF Lep 04633|658[0n]bp|Austria.Vorarlberg|BOLD:AAC3785
Electrophaes corylata|MM18702|658[0n]bp|Finland|BOLD:AAC3785
Electrophaes corylata|MM01277|658[0n]bp|Finland.South Karelia|BOLD:AAC3785
Electrophaes corylata|MM11619|658[0n]bp|Finland|BOLD:AAC3785
Electrophaes corylata|MM01276|614[0n]bp|Finland.South Karelia|BOLD:AAC3785
Thera juniperata|TLMF Lep 06799|658[0n]bp|Austria.Vorarlberg|BOLD:AAA3914
Thera juniperata|MM15809|658[0n]bp|Finland|BOLD:AAA3914
Thera juniperata|MM02885|658[0n]bp|Finland.Northern Ostrobothnia|BOLD:AAA3914
Thera juniperata|MM15810|600[0n]bp|Finland|BOLD:AAA3914
Thera juniperata|TLMF Lep 08786|658[0n]bp|Austria.Vorarlberg|BOLD:AAA3914
Thera juniperata|TLMF Lep 06130|658[0n]bp|Austria.Vorarlberg|BOLD:AAA3914
Thera cognata|MM11613|658[0n]bp|Finland|BOLD:AAB9686
Thera cognata|MM06805|658[0n]bp|Finland.Aland Islands|BOLD:AAB9686
Thera cognata|TLMF Lep 06162|658[0n]bp|Austria.Vorarlberg|BOLD:AAB9686
Thera cognata|MM11612|658[0n]bp|Finland|BOLD:AAB9686
Thera obeliscata|MM12818|658[0n]bp|Finland|BOLD:AAA7522
Thera variata|TLMF Lep 08161|658[0n]bp|Austria.Vorarlberg|BOLD:AAA7521
Thera obeliscata|MM01356|615[0n]bp|Finland.South Karelia|BOLD:AAA7521
Thera obeliscata|MM01357|658[0n]bp|Finland.South Karelia|BOLD:AAA7521
Thera obeliscata|MM18440|658[0n]bp|Finland|BOLD:AAA7521
Thera obeliscata|MM18439|658[0n]bp|Finland|BOLD:AAA7521
Thera obeliscata|MM04559|658[0n]bp|Finland|BOLD:AAA7521
Thera obeliscata|MM00787|658[0n]bp|Finland|BOLD:AAA7521
Thera obeliscata|MM07906|658[0n]bp|Finland|BOLD:AAA7521
Thera obeliscata|MM07905|658[0n]bp|Finland|BOLD:AAA7521
Thera obeliscata|MM00788|658[0n]bp|Finland|BOLD:AAA7521
Thera obeliscata|TLMF Lep 08168|658[0n]bp|Austria.Vorarlberg|BOLD:AAA7521
Thera obeliscata|MM14602|658[0n]bp|Finland|BOLD:AAA7521
Thera variata|MM18437|658[0n]bp|Finland|BOLD:AAA7521

Thera obeliscata|TLMF Lep 08168|658[0n]bp|Austria.Vorarlberg|BOLD:AAA7521
Thera obeliscata|MM14602|658[0n]bp|Finland|BOLD:AAA7521
Thera variata|MM18437|658[0n]bp|Finland|BOLD:AAA7521
Thera variata|MM17989|658[0n]bp|Finland|BOLD:AAA7521
Thera variata|MM00718|658[0n]bp|Finland.Lapland|BOLD:AAA7521
Thera variata|TLMF Lep 06185|658[0n]bp|Austria.Vorarlberg|BOLD:AAA7521
Thera variata|MM17988|658[0n]bp|Finland|BOLD:AAA7521
Thera variata|MM03322|658[0n]bp|Finland.Northern Ostrobothnia|BOLD:AAA7521
Thera variata|MM17990|658[0n]bp|Finland|BOLD:AAA7521
Thera variata|TLMF Lep 06161|658[0n]bp|Austria.Vorarlberg|BOLD:AAA7521
Thera variata|MM18435|658[0n]bp|Finland|BOLD:AAA7521
Thera variata|MM17369|658[0n]bp|Finland|BOLD:AAA7521
Thera variata|MM18436|658[0n]bp|Finland|BOLD:AAA7521
Thera variata|MM18438|658[0n]bp|Finland|BOLD:AAA7521
Eupithecia lariciata|TLMF Lep 07874|658[0n]bp|Austria.Vorarlberg|BOLD:AAA4421
Eupithecia lariciata|MM07976|658[0n]bp|Finland|BOLD:AAA4421
Eupithecia lariciata|MM18504|658[0n]bp|Finland|BOLD:AAA4421
Eupithecia lariciata|MM18505|658[0n]bp|Finland|BOLD:AAA4421
Eupithecia virgaureata|MM23001|658[0n]bp|Finland|BOLD:ACE4093
Eupithecia virgaureata|MM01797|658[0n]bp|Finland.South Karelia|BOLD:ACE4093
Eupithecia virgaureata|MM23002|658[0n]bp|Finland|BOLD:ACE4093
Eupithecia virgaureata|MM23004|658[0n]bp|Finland|BOLD:ACE4093
Eupithecia virgaureata|MM06414|658[0n]bp|Finland|BOLD:ACE4093
Eupithecia virgaureata|MM23009|583[0n]bp|Finland|BOLD:ACE4093
Eupithecia virgaureata|MM10109|658[0n]bp|Finland|BOLD:ACE4093
Eupithecia virgaureata|MM23000|634[0n]bp|Finland|BOLD:ACE4093
Eupithecia virgaureata|MM22998|632[0n]bp|Finland|BOLD:ACE4093
Eupithecia virgaureata|MM23005|658[0n]bp|Finland|BOLD:ACE4093
Eupithecia virgaureata|MM23007|658[0n]bp|Finland|BOLD:ACE4093
Eupithecia virgaureata|MM23008|658[0n]bp|Finland|BOLD:ACE4093
Eupithecia virgaureata|MM22999|658[0n]bp|Finland|BOLD:ACE4093
Eupithecia virgaureata|TLMF Lep 08802|658[0n]bp|Austria.Vorarlberg|BOLD:ACE4093
Eupithecia virgaureata|TLMF Lep 08801|658[0n]bp|Austria.Vorarlberg|BOLD:ACE4093
Eupithecia virgaureata|MM12957|658[0n]bp|Finland|BOLD:ACE4093
Eupithecia virgaureata|MM23003|658[0n]bp|Finland|BOLD:ACE4093
Eupithecia virgaureata|MM23006|658[0n]bp|Finland|BOLD:ACE4093
Eupithecia virgaureata|MM06293|658[0n]bp|Finland|BOLD:ACE4093
Eupithecia tantillaria|MM07961|658[0n]bp|Finland|BOLD:AAB3785
Eupithecia|BIOUG04116-B06|614[0n]bp|Finland.Northern Ostrobothnia|BOLD:AAB3785
Eupithecia|BIOUG04490-E09|599[0n]bp|Finland.Northern Ostrobothnia|BOLD:AAB3785
Eupithecia tantillaria|MM03848|658[0n]bp|Finland.Aland Islands|BOLD:AAB3785
Eupithecia tantillaria|TLMF Lep 04606|658[0n]bp|Austria.Vorarlberg|BOLD:AAB3785
Eupithecia tantillaria|MM12961|627[0n]bp|Finland|BOLD:AAB3785
Eupithecia tantillaria|MM09961|658[0n]bp|Finland|BOLD:AAB3785
Eupithecia venosata|MM15821|658[0n]bp|Finland|BOLD:AAC2038
Eupithecia venosata|MM18478|658[0n]bp|Finland|BOLD:AAC2038
Eupithecia venosata|MM15822|658[0n]bp|Finland|BOLD:AAC2038
Eupithecia venosata|TLMF Lep 06135|658[0n]bp|Austria.Vorarlberg|BOLD:AAC2038
Eupithecia plumbeolata|MM18472|589[0n]bp|Finland|BOLD:AAB8937
Eupithecia plumbeolata|MM14180|658[0n]bp|Finland|BOLD:AAB8937
Eupithecia plumbeolata|MM12970|658[0n]bp|Finland|BOLD:AAB8937
Eupithecia plumbeolata|MM11710|658[0n]bp|Finland|BOLD:AAB8937
Eupithecia plumbeolata|MM07959|658[0n]bp|Finland|BOLD:AAB8937
Eupithecia plumbeolata|MM06648|658[0n]bp|Finland|BOLD:AAB8937
Eupithecia plumbeolata|MM12492|658[0n]bp|Finland|BOLD:AAB8937
Eupithecia plumbeolata|MM11711|658[0n]bp|Finland|BOLD:AAB8937
Eupithecia plumbeolata|MM18473|633[0n]bp|Finland|BOLD:AAB8937
Eupithecia plumbeolata|MM18475|604[0n]bp|Finland|BOLD:AAB8937
Eupithecia plumbeolata|MM01812|658[0n]bp|Finland.South Karelia|BOLD:AAB8937
Eupithecia plumbeolata|MM18474|658[0n]bp|Finland|BOLD:ACF3745
Eupithecia plumbeolata|MM14229|658[0n]bp|Finland.Aland Islands|BOLD:AAB8936
Eupithecia plumbeolata|MM01813|633[0n]bp|Finland.South Karelia|BOLD:AAB8936
Eupithecia plumbeolata|MM06512|658[0n]bp|Finland|BOLD:AAB8936
Eupithecia plumbeolata|MM01814|658[0n]bp|Finland|BOLD:AAB8936
Eupithecia plumbeolata|TLMF Lep 09917|658[0n]bp|Austria.Vorarlberg|BOLD:AAB8936
Eupithecia plumbeolata|TLMF Lep 07575|658[0n]bp|Austria.Vorarlberg|BOLD:AAB8936
Eupithecia plumbeolata|MM01811|658[0n]bp|Finland.South Karelia|BOLD:AAB8936
Eupithecia pusillata|MM17213|658[0n]bp|Finland|BOLD:ABZ6329
Eupithecia pusillata|MM08340|658[0n]bp|Finland|BOLD:ABZ6329
Eupithecia pusillata|MM08143|658[0n]bp|Finland|BOLD:ABZ6329
Eupithecia pusillata|MM23020|630[0n]bp|Finland|BOLD:ABZ6329
Eupithecia pusillata|TLMF Lep 08772|620[0n]bp|Austria.Vorarlberg|BOLD:ABZ6329
Eupithecia pusillata|MM23011|658[0n]bp|Finland|BOLD:ABZ6329
Eupithecia pusillata|MM23012|658[0n]bp|Finland|BOLD:ABZ6329
Eupithecia pusillata|MM23013|658[0n]bp|Finland|BOLD:ABZ6329
Eupithecia pusillata|MM23016|658[0n]bp|Finland|BOLD:ABZ6329
Eupithecia pusillata|MM23017|658[0n]bp|Finland|BOLD:ABZ6329
Eupithecia pusillata|TLMF Lep 08773|658[0n]bp|Austria.Vorarlberg|BOLD:ABZ6329
Eupithecia pusillata|MM23010|624[0n]bp|Finland|BOLD:ABZ6329
Eupithecia pusillata|MM23018|658[0n]bp|Finland|BOLD:ABZ6329
Eupithecia pusillata|MM23019|658[0n]bp|Finland|BOLD:ABZ6329
Eupithecia pusillata|MM23021|658[0n]bp|Finland|BOLD:ABZ6329
Eupithecia pusillata|MM01786|627[0n]bp|Finland.South Karelia|BOLD:ABZ6329
Eupithecia pusillata|TLMF Lep 08798|621[0n]bp|Austria.Vorarlberg|BOLD:ABZ6329
Eupithecia pusillata|MM00781|627[0n]bp|Finland.Northern Ostrobothnia|BOLD:ABZ6329
Eupithecia pusillata|MM01785|658[0n]bp|Finland.South Karelia|BOLD:ABZ6329
Eupithecia tenuiata|TLMF Lep 08546|658[0n]bp|Austria.Vorarlberg|BOLD:AAC7394
Eupithecia tenuiata|TLMF Lep 08774|658[0n]bp|Austria.Vorarlberg|BOLD:AAC7394
Eupithecia tenuiata|MM12968|658[0n]bp|Finland|BOLD:AAC7394
Eupithecia tenuiata|MM01818|658[0n]bp|Finland.South Karelia|BOLD:AAC7394
Eupithecia tenuiata|MM03509|658[0n]bp|Finland.Uusimaa|BOLD:AAC7394
Eupithecia tenuiata|MM01819|658[0n]bp|Finland.South Karelia|BOLD:AAC7394
Eupithecia trisignaria|MM06716|658[0n]bp|Finland.Aland Islands|BOLD:AAD5170
Eupithecia trisignaria|TLMF Lep 06140|658[0n]bp|Austria.Vorarlberg|BOLD:AAD5170
Eupithecia trisignaria|MM09680|658[0n]bp|Finland.Aland Islands|BOLD:AAD5170
Eupithecia trisignaria|MM18492|658[0n]bp|Finland|BOLD:AAD5170
Eupithecia lanceata|TLMF Lep 08811|658[0n]bp|Austria.Vorarlberg|BOLD:AAC5856
Eupithecia lanceata|MM11647|658[0n]bp|Finland|BOLD:AAC5856
Eupithecia lanceata|MM05428|634[0n]bp|Finland.Northern Ostrobothnia|BOLD:AAC5856
Eupithecia lanceata|MM04624|629[0n]bp|Finland.Finland Proper|BOLD:AAC5856
Eupithecia lanceata|MM05445|658[0n]bp|Finland.Northern Ostrobothnia|BOLD:AAC5856
Eupithecia egenaria|TLMF Lep 07982|658[0n]bp|Austria.Vorarlberg|BOLD:AAD9932
Eupithecia egenaria|MM05657|658[0n]bp|Finland.Uusimaa|BOLD:AAD9932

[illegible]
Eupithecia lanceata|MM05445|658[0n]bp|Finland.Northern Ostrobothnia|BOLD:AAC5856
Eupithecia egenaria|TLMF Lep 07982|658[0n]bp|Austria.Vorarlberg|BOLD:AAD9932
Eupithecia egenaria|MM05657|658[0n]bp|Finland.Uusimaa|BOLD:AAD9932
Eupithecia egenaria|MM05925|647[0n]bp|Finland|BOLD:AAD9932
Eupithecia egenaria|MM18704|658[0n]bp|Finland|BOLD:AAD9932
Eupithecia subumbrata|TLMF Lep 07570|658[0n]bp|Austria.Vorarlberg|BOLD:AAC9951
Eupithecia subumbrata|MM12965|658[0n]bp|Finland|BOLD:AAC9952
Eupithecia subumbrata|MM10475|658[0n]bp|Finland|BOLD:AAC9952
Eupithecia subumbrata|MM17891|658[0n]bp|Finland|BOLD:AAC9952
Eupithecia subumbrata|MM03880|658[0n]bp|Finland.South Karelia|BOLD:AAC9952
Eupithecia subumbrata|MM17334|658[0n]bp|Finland|BOLD:AAC9952
Eupithecia subumbrata|MM03879|658[0n]bp|Finland.South Karelia|BOLD:AAC9952
Eupithecia subumbrata|MM03805|658[0n]bp|Finland.Finland Proper|BOLD:AAC9952
Eupithecia subumbrata|MM10474|658[0n]bp|Finland|BOLD:AAC9952
Eupithecia indigata|MM12909|658[0n]bp|Finland|BOLD:AAD4847
Eupithecia indigata|MM04079|644[0n]bp|Finland.Lapland|BOLD:AAD4847
Eupithecia indigata|TLMF Lep 07873|658[0n]bp|Austria.Vorarlberg|BOLD:AAD4847
Eupithecia indigata|MM00568|658[0n]bp|Finland.Northern Ostrobothnia|BOLD:AAD4847
Eupithecia absinthiata|MM17244|658[0n]bp|Finland|BOLD:ACE4737
Eupithecia absinthiata|TLMF Lep 08500|658[0n]bp|Austria.Vorarlberg|BOLD:ACE4737
Eupithecia absinthiata|MM15997|658[0n]bp|Finland|BOLD:ACE4737
Eupithecia absinthiata|TLMF Lep 10002|658[0n]bp|Austria.Vorarlberg|BOLD:ACE4737
Eupithecia absinthiata|MM01820|658[0n]bp|Finland.South Karelia|BOLD:ACE4737
Eupithecia absinthiata|MM15999|658[0n]bp|Finland|BOLD:ACE4737
Eupithecia absinthiata|MM15994|658[0n]bp|Finland|BOLD:ACE4737
Eupithecia absinthiata|MM15995|658[0n]bp|Finland|BOLD:ACE4737
Eupithecia absinthiata|MM13864|658[0n]bp|Finland.Lapland|BOLD:ACE4737
Eupithecia absinthiata|MM01821|658[0n]bp|Finland.South Karelia|BOLD:ACE4737
Eupithecia absinthiata|MM04944|658[0n]bp|Finland.Finland Proper|BOLD:ACE4737
Eupithecia absinthiata|TLMF Lep 08541|658[0n]bp|Austria.Vorarlberg|BOLD:ACE4737
Eupithecia absinthiata|MM15998|658[0n]bp|Finland|BOLD:ACE4737
Eupithecia absinthiata|MM12972|658[0n]bp|Finland|BOLD:ACE4737
Eupithecia satyrata|MM01805|658[0n]bp|Finland.South Karelia|BOLD:AAA5442
Eupithecia satyrata|MM18483|658[0n]bp|Finland|BOLD:AAA5442
Eupithecia satyrata|MM01788|645[1n]bp|Finland.South Karelia|BOLD:AAA4219
Eupithecia satyrata|MM18490|599[0n]bp|Finland|BOLD:AAA4219
Eupithecia satyrata|MM18491|599[0n]bp|Finland|BOLD:AAA4219
Eupithecia satyrata|MM22896|614[0n]bp|Finland|BOLD:AAA4219
Eupithecia satyrata|MM18489|599[0n]bp|Finland|BOLD:AAA4219
Eupithecia satyrata|MM18485|599[0n]bp|Finland|BOLD:AAA4219
Eupithecia satyrata|MM18484|599[0n]bp|Finland|BOLD:AAA4219
Eupithecia satyrata|MM00633|658[0n]bp|Finland|BOLD:AAA4219
Eupithecia satyrata|MM18488|591[0n]bp|Finland|BOLD:AAA4219
Eupithecia satyrata|MM23032|658[1n]bp|Finland|BOLD:AAA4219
Eupithecia satyrata|MM17985|658[0n]bp|Finland|BOLD:AAA4219
Eupithecia satyrata|MM04689|658[0n]bp|Finland.Finland Proper|BOLD:AAA4219
Eupithecia satyrata|MM12962|658[0n]bp|Finland|BOLD:AAA4219
Eupithecia satyrata|MM23023|512[2n]bp|Finland|BOLD:AAA4219
Eupithecia satyrata|MM05487|658[0n]bp|Finland|BOLD:AAA4219
Eupithecia satyrata|MM23028|627[1n]bp|Finland|BOLD:AAA4219
Eupithecia satyrata|MM18486|599[0n]bp|Finland|BOLD:AAA4219
Eupithecia satyrata|MM18482|599[0n]bp|Finland|BOLD:AAA4219
Eupithecia satyrata|MM18487|599[0n]bp|Finland|BOLD:AAA4219
Eupithecia satyrata|MM01789|658[0n]bp|Finland|BOLD:AAA4219
Eupithecia satyrata|MM23024|654[1n]bp|Finland|BOLD:AAA4219
Eupithecia satyrata|MM01787|645[1n]bp|Finland.South Karelia|BOLD:AAA4219
Eupithecia satyrata|TLMF Lep 09882|658[0n]bp|Austria.Vorarlberg|BOLD:AAA4219
Eupithecia satyrata|MM18797|658[0n]bp|Finland|BOLD:AAA4219
Eupithecia satyrata|MM07962|658[0n]bp|Finland|BOLD:AAA4219
Eupithecia satyrata|MM06202|658[0n]bp|Finland|BOLD:AAA4219
Eupithecia satyrata|MM21047|658[0n]bp|Finland|BOLD:AAA4219
Eupithecia satyrata|MM02923|658[0n]bp|Finland|BOLD:AAA4219
Eupithecia satyrata|MM00566|646[0n]bp|Finland.Northern Ostrobothnia|BOLD:AAA4219
Eupithecia satyrata|MM12197|658[0n]bp|Finland|BOLD:AAA4219
Eupithecia satyrata|MM00567|658[0n]bp|Finland|BOLD:AAA4219
Eupithecia satyrata|MM00606|658[0n]bp|Finland|BOLD:AAA4219
Eupithecia satyrata|MM23033|658[0n]bp|Finland|BOLD:AAA4219
Eupithecia satyrata|MM23027|658[0n]bp|Finland|BOLD:AAA4219
Eupithecia satyrata|MM01790|658[0n]bp|Finland|BOLD:AAA4219
Eupithecia intricata|TLMF Lep 09799|658[0n]bp|Austria.Vorarlberg|BOLD:ACJ7134
Eupithecia intricata|MM01806|658[0n]bp|Finland.South Karelia|BOLD:ACJ7134
Eupithecia intricata|MM07963|658[0n]bp|Finland|BOLD:ACJ7134
Eupithecia intricata|MM18480|658[0n]bp|Finland|BOLD:ACJ7134
Eupithecia subfuscata|TLMF Lep 07899|658[0n]bp|Austria.Vorarlberg|BOLD:ABY4251
Eupithecia subfuscata|TLMF Lep 06134|658[0n]bp|Austria.Vorarlberg|BOLD:ACE8007
Eupithecia subfuscata|MM06647|658[0n]bp|Finland|BOLD:ACE8007
Eupithecia subfuscata|TLMF Lep 07984|658[0n]bp|Austria.Vorarlberg|BOLD:ACE8007
Eupithecia subfuscata|TLMF Lep 07901|658[0n]bp|Austria.Vorarlberg|BOLD:ACE8007
Eupithecia subfuscata|TLMF Lep 07900|658[0n]bp|Austria.Vorarlberg|BOLD:ACE8007
Eupithecia subfuscata|MM04228|581[0n]bp|Finland.North Karelia|BOLD:ACE8007
Eupithecia subfuscata|MM07970|658[0n]bp|Finland|BOLD:ACE8007
Eupithecia subfuscata|MM01816|658[0n]bp|Finland.South Karelia|BOLD:ACE8007
Eupithecia subfuscata|MM00613|658[0n]bp|Finland.Northern Ostrobothnia|BOLD:ACE8007
Eupithecia subfuscata|BIOUG04490-E12|601[0n]bp|Finland.Northern Ostrobothnia|BOLD:ACE8007
Eupithecia subfuscata|MM01817|658[0n]bp|Finland.South Karelia|BOLD:ACE8007
Eupithecia exiguata|MM01769|658[0n]bp|Finland.South Karelia|BOLD:AAB5464
Eupithecia exiguata|MM12910|658[0n]bp|Finland|BOLD:AAB5464
Eupithecia exiguata|MM03846|658[0n]bp|Finland.Aland Islands|BOLD:AAB5464
Eupithecia exiguata|TLMF Lep 09881|658[0n]bp|Austria.Vorarlberg|BOLD:AAB5464
Eupithecia exiguata|TLMF Lep 07875|658[0n]bp|Austria.Vorarlberg|BOLD:AAB5464
Eupithecia icterata|TLMF Lep 09997|658[0n]bp|Austria.Vorarlberg|BOLD:ACE7878
Eupithecia icterata|MM04941|658[0n]bp|Finland.Finland Proper|BOLD:AAB6528
Eupithecia icterata|MM12963|658[0n]bp|Finland|BOLD:AAB6528
Eupithecia icterata|MM01798|658[0n]bp|Finland.South Karelia|BOLD:AAB6528
Eupithecia icterata|MM07966|658[0n]bp|Finland|BOLD:AAB6528
Eupithecia vulgata|MM01800|658[0n]bp|Finland.South Karelia|BOLD:AAA8708
Eupithecia vulgata|MM01799|658[0n]bp|Finland.South Karelia|BOLD:AAA8708
Eupithecia vulgata|TLMF Lep 09916|658[0n]bp|Austria.Vorarlberg|BOLD:AAA8708
Eupithecia vulgata|MM08171|658[0n]bp|Finland|BOLD:AAA8708
Eupithecia vulgata|MM00632|658[0n]bp|Finland.Northern Ostrobothnia|BOLD:AAA8708
Eupithecia vulgata|MM08172|643[0n]bp|Finland|BOLD:AAA8708
Eupithecia vulgata|MM00631|635[0n]bp|Finland.Northern Ostrobothnia|BOLD:AAA8708

Eupithecia vulgata|MM00632|658[0n]bp|Finland.Northern Ostrobothnia|BOLD:AAA8708
Eupithecia vulgata|MM08172|643[0n]bp|Finland|BOLD:AAA8708
Eupithecia vulgata|MM00631|635[0n]bp|Finland.Northern Ostrobothnia|BOLD:AAA8708
Eupithecia vulgata|MM12971|658[0n]bp|Finland|BOLD:AAA8708
Eupithecia selinata|MM01808|614[0n]bp|Finland.South Karelia|BOLD:AAD5171
Eupithecia selinata|TLMF Lep 09995|658[0n]bp|Austria.Vorarlberg|BOLD:AAD5171
Eupithecia selinata|MM01809|622[1n]bp|Finland.South Karelia|BOLD:AAD5171
Eupithecia selinata|MM18479|658[0n]bp|Finland|BOLD:AAD5171
Eupithecia selinata|MM05655|658[0n]bp|Finland.Uusimaa|BOLD:AAD5171
Orthonama obstipata|MM18698|658[0n]bp|Finland|BOLD:AAA3431
Orthonama obstipata|TLMF Lep 08787|658[0n]bp|Austria.Vorarlberg|BOLD:AAA3431
Eupithecia abietaria|TLMF Lep 07558|658[0n]bp|Austria.Vorarlberg|BOLD:AAD1084
Eupithecia abietaria|MM14283|658[0n]bp|Finland.Aland Islands|BOLD:AAD1084
Eupithecia abietaria|TLMF Lep 07979|658[0n]bp|Austria.Vorarlberg|BOLD:AAD1084
Eupithecia abietaria|MM09780|658[0n]bp|Finland.Aland Islands|BOLD:AAD1084
Eupithecia abietaria|MM06844|658[0n]bp|Finland.Aland Islands|BOLD:AAD1084
Pasiphila debiliata|TLMF Lep 10005|658[0n]bp|Austria.Vorarlberg|BOLD:AAE7106
Pasiphila debiliata|MM01829|603[0n]bp|Finland.South Karelia|BOLD:AAE7106
Pasiphila debiliata|MM01828|658[0n]bp|Finland.South Karelia|BOLD:AAE7106
Pasiphila debiliata|MM05184|649[0n]bp|Finland.Finland Proper|BOLD:AAE7106
Pasiphila debiliata|MM00715|658[0n]bp|Finland.Northern Ostrobothnia|BOLD:AAE7106
Pasiphila rectangulata|MM12977|658[0n]bp|Finland|BOLD:AAA3075
Pasiphila rectangulata|MM01825|658[0n]bp|Finland.South Karelia|BOLD:AAA3075
Pasiphila rectangulata|MM07981|658[0n]bp|Finland|BOLD:AAA3075
Pasiphila rectangulata|MM01826|658[0n]bp|Finland.South Karelia|BOLD:AAA3075
Pasiphila rectangulata|TLMF Lep 08159|658[0n]bp|Austria.Vorarlberg|BOLD:AAA3075
Pasiphila rectangulata|MM00712|658[0n]bp|Finland.Lapland|BOLD:AAA3075
Horisme tersata|TLMF Lep 08170|658[0n]bp|Austria.Vorarlberg|BOLD:AAC5135
Horisme tersata|MM12781|658[0n]bp|Finland|BOLD:AAC5135
Horisme tersata|MM03863|658[0n]bp|Finland.South Karelia|BOLD:AAC5135
Horisme tersata|MM14251|658[0n]bp|Finland.Aland Islands|BOLD:AAC5135
Baptria tibiale|TLMF Lep 09417|658[0n]bp|Austria.Vorarlberg|BOLD:AAI7598
Baptria tibiale|MM17438|658[0n]bp|Finland|BOLD:AAI7598
Baptria tibiale|MM17437|658[0n]bp|Finland|BOLD:AAI7598
Baptria tibiale|MM17436|658[0n]bp|Finland|BOLD:AAI7598
Baptria tibiale|MM10471|658[0n]bp|Finland|BOLD:AAI7598
Perizoma affinitata|MM18470|658[0n]bp|Finland|BOLD:AAC0743
Perizoma affinitata|MM00094|658[0n]bp|Finland.Lapland|BOLD:AAC0743
Perizoma affinitata|TLMF Lep 04635|658[0n]bp|Austria.Vorarlberg|BOLD:AAC0743
Perizoma affinitata|MM18469|658[0n]bp|Finland|BOLD:AAC0743
Perizoma affinitata|MM01343|658[0n]bp|Finland.South Karelia|BOLD:AAC0743
Perizoma affinitata|MM10117|634[0n]bp|Finland|BOLD:AAC0743
Perizoma affinitata|MM01344|614[0n]bp|Finland.South Karelia|BOLD:AAC0743
Perizoma hydrata|MM11666|658[0n]bp|Finland|BOLD:AAC0743
Perizoma hydrata|TLMF Lep 08496|658[0n]bp|Austria.Vorarlberg|BOLD:AAC0743
Perizoma hydrata|MM12844|658[0n]bp|Finland|BOLD:AAC0743
Perizoma hydrata|MM04686|658[0n]bp|Finland.Finland Proper|BOLD:AAC0743
Perizoma hydrata|MM18468|658[0n]bp|Finland|BOLD:AAC0743
Perizoma hydrata|MM18467|658[0n]bp|Finland|BOLD:AAC0743
Perizoma alchemillata|MM01454|658[0n]bp|Finland.South Karelia|BOLD:AAA9313
Perizoma alchemillata|MM03610|625[0n]bp|Finland.Uusimaa|BOLD:AAA9313
Perizoma alchemillata|TLMF Lep 08800|658[0n]bp|Austria.Vorarlberg|BOLD:AAA9313
Perizoma alchemillata|MM00719|658[0n]bp|Finland.Lapland|BOLD:AAA9313
Perizoma blandiata|MM01437|647[0n]bp|Finland.South Karelia|BOLD:AAC6889
Perizoma blandiata|MM01509|658[0n]bp|Finland.South Karelia|BOLD:AAC6889
Perizoma blandiata|MM07900|658[0n]bp|Finland|BOLD:AAC6889
Perizoma blandiata|MM00335|658[0n]bp|Finland.Uusimaa|BOLD:AAC6889
Perizoma blandiata|MM09825|658[0n]bp|Finland.Aland Islands|BOLD:AAC6889
Perizoma blandiata|TLMF Lep 07569|658[0n]bp|Austria.Vorarlberg|BOLD:AAC6889
Perizoma minorata|MM04107|658[0n]bp|Finland.Lapland|BOLD:AAD4210
Perizoma minorata|MM06363|658[0n]bp|Finland|BOLD:AAD4210
Perizoma minorata|MM00090|658[0n]bp|Finland.Lapland|BOLD:AAD4210
Perizoma minorata|TLMF Lep 00315|658[0n]bp|Austria.Vorarlberg|BOLD:AAD4210
Colostygia aptata|TLMF Lep 06201|658[0n]bp|Austria.Vorarlberg|BOLD:AAB9512
Colostygia aptata|TLMF Lep 08497|658[0n]bp|Austria.Vorarlberg|BOLD:AAB9512
Colostygia aptata|MM01400|658[0n]bp|Finland.South Karelia|BOLD:AAB9512
Colostygia aptata|MM04931|658[0n]bp|Finland.Finland Proper|BOLD:AAB9512
Colostygia aptata|MM01371|658[0n]bp|Finland.South Karelia|BOLD:AAB9512
Colostygia aptata|MM04930|658[0n]bp|Finland.Finland Proper|BOLD:AAB9512
Colostygia aptata|MM01370|615[0n]bp|Finland.South Karelia|BOLD:AAB9512
Colostygia olivata|MM09833|658[0n]bp|Finland.Aland Islands|BOLD:AAB9511
Colostygia olivata|MM09552|658[0n]bp|Finland.Aland Islands|BOLD:AAB9511
Colostygia olivata|MM18441|658[0n]bp|Finland|BOLD:AAB9511
Colostygia olivata|TLMF Lep 08797|658[0n]bp|Austria.Vorarlberg|BOLD:AAB9509
Colostygia turbata|TLMF Lep 07562|658[0n]bp|Austria.Vorarlberg|BOLD:AAC0785
Colostygia turbata|MM15814|658[0n]bp|Finland|BOLD:AAC0784
Colostygia turbata|MM18442|647[0n]bp|Finland|BOLD:AAC0784
Colostygia turbata|MM10469|658[0n]bp|Finland|BOLD:AAC0784
Colostygia turbata|MM18443|658[0n]bp|Finland|BOLD:AAC0784
Anticlea derivata|TLMF Lep 07863|658[0n]bp|Austria.Vorarlberg|BOLD:AAD2706
Anticlea derivata|MM13886|658[0n]bp|Finland|BOLD:AAD2706
Anticlea derivata|MM17435|658[0n]bp|Finland|BOLD:AAD2706
Colostygia pectinataria|MM01388|633[0n]bp|Finland.South Karelia|BOLD:AAB4885
Colostygia pectinataria|MM02922|658[0n]bp|Finland.Northern Ostrobothnia|BOLD:AAB4885
Colostygia pectinataria|MM01387|633[0n]bp|Finland.South Karelia|BOLD:AAB4885
Colostygia pectinataria|TLMF Lep 07977|658[0n]bp|Austria.Vorarlberg|BOLD:AAB4885
Mesotype didymata|TLMF Lep 08861|658[0n]bp|Austria.Vorarlberg|BOLD:AAE3950
Mesotype didymata|MM00783|621[0n]bp|Finland.Northern Ostrobothnia|BOLD:AAE3950
Mesotype didymata|MM01355|618[0n]bp|Finland.South Karelia|BOLD:AAE3950
Mesotype didymata|MM01354|658[0n]bp|Finland.South Karelia|BOLD:AAE3950
Operophtera brumata|MM08226|647[0n]bp|Finland|BOLD:AAA3963
Operophtera brumata|MM14726|658[0n]bp|Finland.Aland Islands|BOLD:AAA3963
Operophtera brumata|MM08270|658[0n]bp|Finland|BOLD:AAA3963
Operophtera brumata|TLMF Lep 06802|658[0n]bp|Austria.Vorarlberg|BOLD:AAA3963
Operophtera fagata|MM02698|658[0n]bp|Finland.South Karelia|BOLD:AAD0141
Operophtera fagata|TLMF Lep 08788|658[0n]bp|Austria.Vorarlberg|BOLD:AAD0141
Operophtera fagata|MM18445|658[0n]bp|Finland|BOLD:AAD0141
Operophtera fagata|MM08269|658[0n]bp|Finland|BOLD:AAD0141
Asthena albulata|MM11625|658[0n]bp|Finland|BOLD:AAC7503
Asthena albulata|TLMF Lep 08534|658[0n]bp|Austria.Vorarlberg|BOLD:AAC7503
Asthena albulata|MM06107|658[0n]bp|Finland|BOLD:AAC7503
Asthena albulata|MM11624|658[0n]bp|Finland|BOLD:AAC7503
Epirrita autumnata|TLMF Lep 06129|658[0n]bp|Austria.Vorarlberg|BOLD:AAA5907

Asthena albulata|MM06107|658[0n]bp|Finland|BOLD:AAC7503
Asthena albulata|MM11624|658[0n]bp|Finland|BOLD:AAC7503
Epirrita autumnata|TLMF Lep 06129|658[0n]bp|Austria.Vorarlberg|BOLD:AAA5907
Epirrita autumnata|TLMF Lep 09875|658[0n]bp|Austria.Vorarlberg|BOLD:AAA5906
Epirrita autumnata|MM04509|658[0n]bp|Finland.Finland Proper|BOLD:AAA5906
Epirrita autumnata|MM07901|658[0n]bp|Finland|BOLD:AAA5906
Epirrita autumnata|MM02687|658[0n]bp|Finland|BOLD:AAA5906
Epirrita autumnata|MM18447|658[0n]bp|Finland|BOLD:AAA5906
Epirrita autumnata|MM02686|658[0n]bp|Finland.South Karelia|BOLD:AAA5906
Epirrita autumnata|MM12813|658[0n]bp|Finland|BOLD:AAA5906
Epirrita autumnata|MM00808|658[0n]bp|Finland|BOLD:ACE7803
Epirrita autumnata|MM00807|658[0n]bp|Finland.Northern Ostrobothnia|BOLD:ACE7803
Epirrita christyi|MM14730|658[0n]bp|Finland.Aland Islands|BOLD:AAB0936
Epirrita christyi|MM03190|620[0n]bp|Finland.Aland Islands|BOLD:AAB0936
Epirrita christyi|MM14724|658[0n]bp|Finland.Aland Islands|BOLD:AAB0936
Epirrita christyi|MM14725|658[0n]bp|Finland.Aland Islands|BOLD:AAB0936
Epirrita christyi|TLMF Lep 06128|658[0n]bp|Austria.Vorarlberg|BOLD:AAB0936
Hydrelia flammeolaria|TLMF Lep 07868|658[0n]bp|Austria.Vorarlberg|BOLD:ACE6107
Hydrelia flammeolaria|MM04553|613[0n]bp|Finland.Finland Proper|BOLD:AAC5746
Hydrelia flammeolaria|MM01431|658[0n]bp|Finland.South Karelia|BOLD:AAC5746
Hydrelia flammeolaria|MM01430|658[0n]bp|Finland.South Karelia|BOLD:AAC5746
Lampropteryx suffumata|MM13857|658[0n]bp|Finland|BOLD:AAB2818
Lampropteryx suffumata|MM11570|658[0n]bp|Finland|BOLD:AAB2818
Lampropteryx suffumata|MM06420|658[0n]bp|Finland|BOLD:AAB2818
Lampropteryx suffumata|MM01302|658[0n]bp|Finland.South Karelia|BOLD:AAB2818
Lampropteryx suffumata|MM05419|658[0n]bp|Finland.Aland Islands|BOLD:AAB2818
Lampropteryx suffumata|MM07800|658[0n]bp|Finland|BOLD:AAB2818
Lampropteryx suffumata|TLMF Lep 07891|658[0n]bp|Austria.Vorarlberg|BOLD:AAB2818
Lampropteryx suffumata|MM01301|641[0n]bp|Finland.South Karelia|BOLD:AAB2818
Lampropteryx suffumata|MM06419|658[0n]bp|Finland|BOLD:AAB2818
Euchoeca nebulata|TLMF Lep 07895|658[0n]bp|Austria.Vorarlberg|BOLD:AAD0146
Euchoeca nebulata|MM01443|658[0n]bp|Finland.South Karelia|BOLD:AAD0146
Euchoeca nebulata|MM01316|658[0n]bp|Finland.South Karelia|BOLD:AAD0146
Euchoeca nebulata|MM01317|658[0n]bp|Finland.South Karelia|BOLD:AAD0146
Hydrelia sylvata|MM01441|658[0n]bp|Finland.South Karelia|BOLD:AAD3792
Hydrelia sylvata|MM01442|658[0n]bp|Finland.South Karelia|BOLD:AAD3792
Hydrelia sylvata|TLMF Lep 07894|644[0n]bp|Austria.Vorarlberg|BOLD:AAD3792
Hydrelia sylvata|MM08365|614[0n]bp|Finland|BOLD:AAD3792
Venusia blomeri|MM01313|658[0n]bp|Finland.South Karelia|BOLD:AAC9722
Venusia blomeri|MM01755|658[0n]bp|Finland.South Karelia|BOLD:AAC9722
Venusia blomeri|TLMF Lep 08196|658[0n]bp|Austria.Vorarlberg|BOLD:AAC9722
Venusia blomeri|MM01312|658[0n]bp|Finland.South Karelia|BOLD:AAC9722
Venusia blomeri|MM14282|658[0n]bp|Finland.Aland Islands|BOLD:AAC9722
Venusia blomeri|MM01457|658[0n]bp|Finland.South Karelia|BOLD:AAC9722
Venusia cambrica|MM15832|658[0n]bp|Finland|BOLD:AAA1586
Venusia cambrica|MM10484|658[0n]bp|Finland|BOLD:AAA1586
Venusia cambrica|MM10483|658[0n]bp|Finland|BOLD:AAA1586
Venusia cambrica|TLMF Lep 06179|658[0n]bp|Austria.Vorarlberg|BOLD:AAA1586
Venusia cambrica|MM07864|658[0n]bp|Finland|BOLD:AAA1586
Epirrhoe galiata|TLMF Lep 07866|658[0n]bp|Austria.Vorarlberg|BOLD:AAB2097
Epirrhoe galiata|MM06895|658[0n]bp|Finland.Aland Islands|BOLD:AAB2097
Epirrhoe galiata|MM00339|658[0n]bp|Finland.Uusimaa|BOLD:AAB2097
Epirrhoe galiata|MM18424|658[0n]bp|Finland|BOLD:AAB2097
Epirrhoe alternata|MM23192|658[0n]bp|Finland|BOLD:ACF4363
Epirrhoe alternata|MM11620|658[0n]bp|Finland|BOLD:ACF4363
Epirrhoe alternata|TLMF Lep 07852|658[0n]bp|Austria.Vorarlberg|BOLD:ACF4363
Epirrhoe alternata|MM00615|658[0n]bp|Finland.Northern Ostrobothnia|BOLD:ACF4363
Epirrhoe alternata|MM12244|658[0n]bp|Finland|BOLD:ACF4363
Epirrhoe alternata|MM12834|658[0n]bp|Finland|BOLD:ACF4363
Epirrhoe alternata|MM04581|658[0n]bp|Finland.Finland Proper|BOLD:ACF4363
Epirrhoe alternata|MM07844|658[0n]bp|Finland|BOLD:ACF4363
Epirrhoe alternata|MM01342|611[0n]bp|Finland.South Karelia|BOLD:ACF4363
Epirrhoe alternata|MM01341|650[0n]bp|Finland.South Karelia|BOLD:ACF4363
Epirrhoe rivata|MM05919|658[0n]bp|Finland|BOLD:AAD7207
Epirrhoe rivata|MM18699|631[0n]bp|Finland|BOLD:AAD7207
Epirrhoe rivata|MM06105|658[0n]bp|Finland|BOLD:AAD7207
Epirrhoe rivata|TLMF Lep 09880|658[0n]bp|Austria.Vorarlberg|BOLD:AAD7207
Epirrhoe rivata|MM15909|658[0n]bp|Finland|BOLD:AAD7207
Epirrhoe tristata|MM01376|633[0n]bp|Finland.South Karelia|BOLD:AAD7202
Epirrhoe tristata|MM06201|658[0n]bp|Finland|BOLD:AAD7202
Epirrhoe tristata|MM06810|658[0n]bp|Finland.Aland Islands|BOLD:AAD7202
Epirrhoe tristata|MM09475|658[0n]bp|Finland.Aland Islands|BOLD:AAD7202
Epirrhoe tristata|TLMF Lep 08548|658[0n]bp|Austria.Vorarlberg|BOLD:AAD7202
Martania taeniata|MM01448|658[0n]bp|Finland.South Karelia|BOLD:AAE9770
Martania taeniata|TLMF Lep 09993|658[0n]bp|Austria.Vorarlberg|BOLD:AAE9770
Martania taeniata|TLMF Lep 09416|658[0n]bp|Austria.Vorarlberg|BOLD:AAE9770
Martania taeniata|MM01764|647[0n]bp|Finland.South Karelia|BOLD:AAE9770
Martania taeniata|MM08468|658[0n]bp|Finland|BOLD:AAE9770
Xanthorhoe biriviata|MM15800|658[0n]bp|Finland|BOLD:AAD1977
Xanthorhoe biriviata|MM10463|658[0n]bp|Finland|BOLD:AAD1977
Xanthorhoe biriviata|TLMF Lep 06200|658[0n]bp|Austria.Vorarlberg|BOLD:AAD1977
Xanthorhoe biriviata|TLMF Lep 08171|658[0n]bp|Austria.Vorarlberg|BOLD:AAD1977
Xanthorhoe biriviata|MM15801|658[0n]bp|Finland|BOLD:AAD1977
Xanthorhoe designata|MM02858|658[0n]bp|Finland.Northern Ostrobothnia|BOLD:AAA7001
Xanthorhoe designata|MM03701|648[0n]bp|Finland.Aland Islands|BOLD:AAA7001
Xanthorhoe designata|MM10118|658[0n]bp|Finland|BOLD:AAA7001
Xanthorhoe designata|MM07830|658[0n]bp|Finland|BOLD:ABZ0894
Xanthorhoe designata|MM07831|658[0n]bp|Finland|BOLD:ABZ0894
Xanthorhoe designata|MM08341|658[0n]bp|Finland|BOLD:ABZ0894
Xanthorhoe designata|MM06362|658[0n]bp|Finland|BOLD:ABZ0894
Xanthorhoe designata|TLMF Lep 07896|658[0n]bp|Austria.Vorarlberg|BOLD:ABZ0894
Xanthorhoe designata|MM06028|658[0n]bp|Finland|BOLD:ABZ0894
Xanthorhoe designata|MM12336|658[0n]bp|Finland|BOLD:ABZ0894
Xanthorhoe fluctuata|TLMF Lep 08779|658[0n]bp|Austria.Vorarlberg|BOLD:AAA6836
Xanthorhoe fluctuata|MM00088|614[0n]bp|Finland.Lapland|BOLD:AAA6836
Xanthorhoe fluctuata|MM01326|633[0n]bp|Finland.South Karelia|BOLD:AAA6836
Xanthorhoe fluctuata|MM01369|650[0n]bp|Finland.South Karelia|BOLD:AAA6836
Xanthorhoe decoloraria|TLMF Lep 05854|658[0n]bp|Austria.Vorarlberg|BOLD:AAA5318
Xanthorhoe decoloraria|MM04106|658[0n]bp|Finland.Lapland|BOLD:AAA5318
Xanthorhoe decoloraria|MM02857|658[0n]bp|Finland.Northern Ostrobothnia|BOLD:AAA5318
Xanthorhoe decoloraria|MM06361|658[0n]bp|Finland|BOLD:AAA5318
Xanthorhoe montanata|MM18420|658[0n]bp|Finland|BOLD:AAB2524
Xanthorhoe montanata|MM02846|611[5n]bp|Finland.Northern Ostrobothnia|BOLD:AAB2524

Xanthorhoe decoloraria|[illegible]|658[0n]bp|Finland.Northern Ostrobothnia|BOLD:AAA5318
Xanthorhoe decoloraria|MM06361|658[0n]bp|Finland|BOLD:AAA5318
Xanthorhoe montanata|MM18420|658[0n]bp|Finland|BOLD:AAB2524
Xanthorhoe montanata|MM02846|611[5n]bp|Finland.Northern Ostrobothnia|BOLD:AAB2524
Xanthorhoe montanata|TLMF Lep 08557|658[0n]bp|Austria.Vorarlberg|BOLD:AAB2524
Xanthorhoe montanata|MM08453|658[0n]bp|Finland|BOLD:AAB2524
Camptogramma bilineata|MM12329|658[0n]bp|Finland|BOLD:AAA8017
Camptogramma bilineata|TLMF Lep 08131|658[0n]bp|Austria.Vorarlberg|BOLD:AAA8017
Camptogramma bilineata|MM03540|658[0n]bp|Finland.Uusimaa|BOLD:AAA8017
Camptogramma bilineata|MM03702|658[0n]bp|Finland.Aland Islands|BOLD:AAA8017
Xanthorhoe quadrifasiata|MM02826|658[0n]bp|Finland.Northern Ostrobothnia|BOLD:AAC9330
Xanthorhoe quadrifasiata|MM01473|658[0n]bp|Finland.South Karelia|BOLD:AAC9330
Xanthorhoe quadrifasiata|MM01474|658[0n]bp|Finland.South Karelia|BOLD:AAC9330
Xanthorhoe quadrifasiata|TLMF Lep 08552|658[0n]bp|Austria.Vorarlberg|BOLD:AAC9330
Xanthorhoe quadrifasiata|MM23189|658[0n]bp|Finland|BOLD:AAC9330
Xanthorhoe ferrugata|MM13943|658[0n]bp|Finland|BOLD:ACF5678
Xanthorhoe ferrugata|MM01440|658[0n]bp|Finland.South Karelia|BOLD:ACF5678
Xanthorhoe ferrugata|MM12337|658[0n]bp|Finland|BOLD:ACF5678
Xanthorhoe ferrugata|MM04839|658[0n]bp|Finland|BOLD:ACF4785
Xanthorhoe ferrugata|MM03801|658[0n]bp|Finland|BOLD:ACF4785
Xanthorhoe ferrugata|MM12838|658[0n]bp|Finland|BOLD:ACF4785
Xanthorhoe ferrugata|MM12839|658[0n]bp|Finland|BOLD:ACF4785
Xanthorhoe ferrugata|MM12873|658[0n]bp|Finland|BOLD:ACF4785
Xanthorhoe ferrugata|MM09677|658[0n]bp|Finland|BOLD:ACF4785
Xanthorhoe ferrugata|MM11618|658[0n]bp|Finland|BOLD:ACF4785
Xanthorhoe ferrugata|TLMF Lep 07971|658[0n]bp|Austria.Vorarlberg|BOLD:ACF4785
Xanthorhoe ferrugata|TLMF Lep 07897|658[0n]bp|Austria.Vorarlberg|BOLD:ACF4785
Xanthorhoe ferrugata|MM18422|658[0n]bp|Finland|BOLD:ACF4785
Xanthorhoe ferrugata|MM03872|658[0n]bp|Finland|BOLD:ACF4785
Xanthorhoe ferrugata|MM01765|658[0n]bp|Finland.South Karelia|BOLD:ACF4785
Xanthorhoe ferrugata|MM03514|658[0n]bp|Finland.Kymenlaakso|BOLD:ACF4785
Xanthorhoe ferrugata|MM18421|658[0n]bp|Finland|BOLD:ACF4785
Xanthorhoe ferrugata|MM17340|658[0n]bp|Finland|BOLD:ACF4785
Xanthorhoe ferrugata|MM01396|658[0n]bp|Finland.South Karelia|BOLD:ACF4785
Xanthorhoe spadicearia|MM01345|615[0n]bp|Finland.South Karelia|BOLD:AAB7980
Xanthorhoe spadicearia|TLMF Lep 10008|658[0n]bp|Austria.Vorarlberg|BOLD:AAB7980
Xanthorhoe spadicearia|MM04108|658[0n]bp|Finland.Lapland|BOLD:AAB7980
Xanthorhoe spadicearia|MM01346|658[0n]bp|Finland.South Karelia|BOLD:AAB7980
Coleophora albidella|MM06528|658[0n]bp|Finland|BOLD:AAB8489
Coleophora albidella|MM17555|658[0n]bp|Finland|BOLD:AAB8489
Coleophora albidella|TLMF Lep 10038|658[0n]bp|Austria.Vorarlberg|BOLD:AAB8489
Coleophora albidella|MM06702|658[0n]bp|Finland.Aland Islands|BOLD:AAB8489
Coleophora albidella|MM06765|658[0n]bp|Finland.Aland Islands|BOLD:AAB8489
Coleophora albidella|MM06529|658[0n]bp|Finland|BOLD:AAB8489
Coleophora albidella|MM02496|658[0n]bp|Finland.South Karelia|BOLD:AAB8489
Coleophora albidella|MM12409|658[0n]bp|Finland|BOLD:AAB8489
Coleophora albidella|MM12436|658[0n]bp|Finland|BOLD:AAB8489
Coleophora betulella|MM09797|658[0n]bp|Finland.Aland Islands|BOLD:AAE8825
Coleophora betulella|TLMF Lep 07529|658[0n]bp|Austria.Vorarlberg|BOLD:AAE8825
Coleophora betulella|MM06617|658[0n]bp|Finland|BOLD:AAE8825
Coleophora betulella|MM10145|655[0n]bp|Finland|BOLD:AAE8825
Coleophora betulella|MM18171|658[0n]bp|Finland|BOLD:AAE8825
Coleophora kuehnella|MM17557|631[0n]bp|Finland|BOLD:AAC6417
Coleophora kuehnella|TLMF Lep 08049|658[0n]bp|Austria.Vorarlberg|BOLD:AAC6417
Coleophora kuehnella|MM18967|658[0n]bp|Finland|BOLD:AAC6417
Coleophora kuehnella|MM18968|514[0n]bp|Finland|BOLD:AAC6417
Coleophora fuscocuprella|MM18995|620[0n]bp|Finland|BOLD:AAF3835
Coleophora fuscocuprella|MM15575|658[0n]bp|Finland.Aland Islands|BOLD:AAF3835
Coleophora fuscocuprella|TLMF Lep 09975|658[0n]bp|Austria.Vorarlberg|BOLD:AAF3835
Coleophora lusciniaepennella|TLMF Lep 10044|658[0n]bp|Austria.Vorarlberg|BOLD:AAY9622
Coleophora lusciniaepennella|MM23100|658[0n]bp|Finland|BOLD:AAY9622
Coleophora lusciniaepennella|MM23099|658[0n]bp|Finland|BOLD:AAY9622
Coleophora lusciniaepennella|MM02494|658[0n]bp|Finland.South Karelia|BOLD:AAY9622
Coleophora lusciniaepennella|MM00961|658[0n]bp|Finland|BOLD:AAY9622
Coleophora lusciniaepennella|MM09305|658[0n]bp|Finland|BOLD:AAY9622
Coleophora lusciniaepennella|MM13701|658[0n]bp|Finland|BOLD:AAY9622
Coleophora lusciniaepennella|MM08687|658[0n]bp|Finland|BOLD:AAY9622
Coleophora lusciniaepennella|MM23104|658[0n]bp|Finland|BOLD:AAY9622
Coleophora lusciniaepennella|MM23101|657[0n]bp|Finland|BOLD:AAY9622
Coleophora lusciniaepennella|MM23105|625[0n]bp|Finland|BOLD:AAY9622
Coleophora lusciniaepennella|MM23097|612[0n]bp|Finland|BOLD:AAY9622
Coleophora lusciniaepennella|MM23103|612[0n]bp|Finland|BOLD:AAY9622
Coleophora lusciniaepennella|MM23096|552[1n]bp|Finland|BOLD:AAY9622
Coleophora lusciniaepennella|MM15982|620[1n]bp|Finland|BOLD:AAY9622
Coleophora lusciniaepennella|MM23098|658[0n]bp|Finland|BOLD:AAY9622
Coleophora lusciniaepennella|MM15983|658[0n]bp|Finland|BOLD:AAY9622
Coleophora ahenella|TLMF Lep 09969|658[0n]bp|Austria.Vorarlberg|BOLD:AAD0941
Coleophora ahenella|MM14850|658[0n]bp|Finland.Aland Islands|BOLD:AAD0941
Coleophora ahenella|MM02506|631[0n]bp|Finland.South Karelia|BOLD:AAD0941
Coleophora ahenella|MM14849|658[0n]bp|Finland.Aland Islands|BOLD:AAD0941
Coleophora orbitella|MM09350|658[0n]bp|Finland|BOLD:AAF4235
Coleophora orbitella|MM09315|658[0n]bp|Finland|BOLD:AAF4235
Coleophora orbitella|MM21136|658[0n]bp|Finland|BOLD:AAF4235
Coleophora orbitella|MM09433|658[0n]bp|Finland|BOLD:AAF4235
Coleophora orbitella|MM09641|658[0n]bp|Finland.Aland Islands|BOLD:AAF4235
Coleophora orbitella|TLMF Lep 09953|658[0n]bp|Austria.Vorarlberg|BOLD:AAF4235
Coleophora mayrella|MM14468|658[0n]bp|Finland|BOLD:AAB3182
Coleophora mayrella|MM02532|658[0n]bp|Finland.South Karelia|BOLD:AAB3182
Coleophora mayrella|MM03169|658[0n]bp|Finland.Northern Ostrobothnia|BOLD:AAB3182
Coleophora mayrella|TLMF Lep 08059|658[0n]bp|Austria.Vorarlberg|BOLD:AAB3182
Coleophora mayrella|TLMF Lep 08057|658[0n]bp|Austria.Vorarlberg|BOLD:AAB3182
Coleophora milvipennis|MM08586|658[0n]bp|Finland|BOLD:AAB7467
Coleophora milvipennis|MM06475|658[0n]bp|Finland|BOLD:AAB7467
Coleophora milvipennis|MM14058|658[0n]bp|Finland|BOLD:AAB7467
Coleophora milvipennis|MM17883|658[0n]bp|Finland|BOLD:AAB7467
Coleophora milvipennis|MM02475|658[0n]bp|Finland.South Karelia|BOLD:AAB7467
Coleophora milvipennis|TLMF Lep 09963|658[0n]bp|Austria.Vorarlberg|BOLD:AAB7467
Coleophora milvipennis|MM06624|658[0n]bp|Finland|BOLD:AAB7467
Coleophora trigeminella|MM17682|658[0n]bp|Finland|BOLD:AAD9304
Coleophora trigeminella|TLMF Lep 08065|658[0n]bp|Austria.Vorarlberg|BOLD:AAD9304
Coleophora trigeminella|MM10436|658[0n]bp|Finland|BOLD:AAD9304
Coleophora gryphipennella|TLMF Lep 09203|599[0n]bp|Austria.Vorarlberg|BOLD:AAE8826
Coleophora gryphipennella|MM09601|658[0n]bp|Finland.Aland Islands|BOLD:AAE8826

Coleophora trigeminella|MM10436|658[0n]bp|Finland|BOLD:AAD9304
Coleophora gryphipennella|TLMF Lep 09203|599[0n]bp|Austria.Vorarlberg|BOLD:AAE8826
Coleophora gryphipennella|MM09601|658[0n]bp|Finland.Aland Islands|BOLD:AAE8826
Coleophora gryphipennella|MM03932|658[0n]bp|Finland.Uusimaa|BOLD:AAE8826
Coleophora gryphipennella|MM09636|658[0n]bp|Finland.Aland Islands|BOLD:AAE8826
Coleophora gryphipennella|MM13678|658[0n]bp|Finland|BOLD:AAE8826
Coleophora flavipennella|MM09776|658[1n]bp|Finland.Aland Islands|BOLD:ACF1822
Coleophora flavipennella|MM09703|658[0n]bp|Finland.Aland Islands|BOLD:ACF1822
Coleophora flavipennella|MM06704|658[0n]bp|Finland.Aland Islands|BOLD:ACF1822
Coleophora flavipennella|MM06759|658[0n]bp|Finland.Aland Islands|BOLD:ACF1822
Coleophora flavipennella|TLMF Lep 08416|658[0n]bp|Austria.Vorarlberg|BOLD:ACF1822
Coleophora flavipennella|MM09808|658[0n]bp|Finland.Aland Islands|BOLD:ACF1822
Coleophora flavipennella|TLMF Lep 08238|658[0n]bp|Austria.Vorarlberg|BOLD:ACF1822
Coleophora flavipennella|TLMF Lep 08257|658[0n]bp|Austria.Vorarlberg|BOLD:ACF1822
Coleophora flavipennella|TLMF Lep 08256|658[0n]bp|Austria.Vorarlberg|BOLD:AAC3993
Coleophora serratella|MM09574|658[0n]bp|Finland.Aland Islands|BOLD:AAB5543
Coleophora serratella|MM06761|614[0n]bp|Finland.Aland Islands|BOLD:AAB5543
Coleophora serratella|MM09708|658[0n]bp|Finland.Aland Islands|BOLD:AAB5543
Coleophora serratella|MM00068|669[0n]bp|Finland.Northern Ostrobothnia|BOLD:AAB5543
Coleophora serratella|MM09707|562[0n]bp|Finland.Aland Islands|BOLD:AAB5543
Coleophora serratella|MM00067|611[0n]bp|Finland.Northern Ostrobothnia|BOLD:AAB5543
Coleophora serratella|MM06827|654[0n]bp|Finland.Aland Islands|BOLD:AAB5543
Coleophora serratella|TLMF Lep 09968|658[0n]bp|Austria.Vorarlberg|BOLD:AAB5543
Coleophora serratella|MM00700|658[0n]bp|Finland.Lapland|BOLD:AAB5543
Coleophora serratella|MM09810|658[0n]bp|Finland.Aland Islands|BOLD:AAB5543
Coleophora serratella|MM03484|658[0n]bp|Finland.Kymenlaakso|BOLD:AAB5543
Coleophora serratella|MM02502|658[0n]bp|Finland.South Karelia|BOLD:AAB5543
Coleophora serratella|MM09799|658[0n]bp|Finland.Aland Islands|BOLD:AAB5543
Coleophora serratella|MM06726|658[0n]bp|Finland.Aland Islands|BOLD:AAB5543
Coleophora uliginosella|MM21105|658[0n]bp|Finland|BOLD:AAE9656
Coleophora uliginosella|MM20540|658[0n]bp|Finland.Uusimaa|BOLD:AAE9656
Coleophora uliginosella|MM15981|658[0n]bp|Finland|BOLD:AAE9656
Coleophora uliginosella|TLMF Lep 02846|658[0n]bp|Austria.Vorarlberg|BOLD:AAE9656
Coleophora alticolella|MM06383|658[0n]bp|Finland|BOLD:AAC6419
Coleophora alticolella|MM14166|658[0n]bp|Finland|BOLD:AAC6419
Coleophora alticolella|MM09908|658[0n]bp|Finland|BOLD:AAC6419
Coleophora alticolella|MM06554|658[0n]bp|Finland|BOLD:AAC6419
Coleophora alticolella|MM09910|658[0n]bp|Finland|BOLD:AAC6419
Coleophora alticolella|MM06483|658[0n]bp|Finland|BOLD:AAC6419
Coleophora alticolella|MM03818|637[0n]bp|Finland.Aland Islands|BOLD:AAC6419
Coleophora alticolella|MM02463|658[0n]bp|Finland.South Karelia|BOLD:AAC6419
Coleophora alticolella|MM06242|658[0n]bp|Finland|BOLD:AAC6419
Coleophora alticolella|TLMF Lep 09209|658[0n]bp|Austria.Vorarlberg|BOLD:AAC6419
Coleophora alticolella|TLMF Lep 04305|658[0n]bp|Austria.Vorarlberg|BOLD:AAC6419
Coleophora alticolella|TLMF Lep 02845|658[0n]bp|Austria.Vorarlberg|BOLD:AAC6419
Coleophora alticolella|TLMF Lep 09213|658[0n]bp|Austria.Vorarlberg|BOLD:AAC6419
Coleophora alticolella|TLMF Lep 09976|658[0n]bp|Austria.Vorarlberg|BOLD:AAC6419
Coleophora taeniipennella|MM14245|658[0n]bp|Finland.Aland Islands|BOLD:AAC8749
Coleophora taeniipennella|MM14244|658[0n]bp|Finland.Aland Islands|BOLD:AAC8749
Coleophora taeniipennella|MM02500|658[0n]bp|Finland.South Karelia|BOLD:AAC8749
Coleophora taeniipennella|MM02476|658[0n]bp|Finland.South Karelia|BOLD:AAC8749
Coleophora taeniipennella|MM09831|566[0n]bp|Finland.Aland Islands|BOLD:AAC8749
Coleophora taeniipennella|TLMF Lep 07528|658[0n]bp|Austria.Vorarlberg|BOLD:AAC8749
Coleophora taeniipennella|MM09909|658[0n]bp|Finland|BOLD:AAC8749
Coleophora taeniipennella|MM12040|658[0n]bp|Finland|BOLD:AAC8749
Coleophora caespititiella|TLMF Lep 09965|658[3n]bp|Austria.Vorarlberg|BOLD:AAE8821
Coleophora caespititiella|MM06220|658[0n]bp|Finland|BOLD:AAE8821
Coleophora caespititiella|MM06232|658[0n]bp|Finland|BOLD:AAE8821
Coleophora caespititiella|MM02473|642[0n]bp|Finland.South Karelia|BOLD:AAE8821
Coleophora caespititiella|MM02472|591[0n]bp|Finland.South Karelia|BOLD:AAE8821
Coleophora caespititiella|MM19895|612[0n]bp|Finland.Uusimaa|BOLD:AAE8821
Coleophora caespititiella|MM02471|643[0n]bp|Finland.South Karelia|BOLD:AAE8821
Coleophora caespititiella|MM06481|658[0n]bp|Finland|BOLD:AAE8821
Coleophora caespititiella|MM06477|658[0n]bp|Finland|BOLD:AAE8821
Coleophora sternipennella|TLMF Lep 08467|658[0n]bp|Austria.Vorarlberg|BOLD:AAD3945
Coleophora sternipennella|MM13680|658[0n]bp|Finland|BOLD:AAE9649
Coleophora sternipennella|MM03176|658[0n]bp|Finland.Northern Ostrobothnia|BOLD:AAE9649
Coleophora sternipennella|MM02509|658[0n]bp|Finland.South Karelia|BOLD:AAE9649
Coleophora sternipennella|MM02504|658[0n]bp|Finland.South Karelia|BOLD:AAE9649
Coleophora sternipennella|MM16717|658[0n]bp|Finland.Finland Proper|BOLD:AAE9649
Coleophora sternipennella|MM03235|658[0n]bp|Finland.Northern Ostrobothnia|BOLD:AAE9649
Coleophora sternipennella|MM06829|658[0n]bp|Finland.Aland Islands|BOLD:AAE9649
Coleophora sternipennella|MM09519|658[0n]bp|Finland.Aland Islands|BOLD:AAE9649
Coleophora sternipennella|MM10059|658[0n]bp|Finland|BOLD:AAE9649
Coleophora sternipennella|TLMF Lep 08462|658[0n]bp|Austria.Vorarlberg|BOLD:AAE9649
Coleophora sternipennella|MM09520|658[0n]bp|Finland.Aland Islands|BOLD:AAE9649
Coleophora sternipennella|MM16715|658[0n]bp|Finland.Uusimaa|BOLD:AAE9649
Coleophora virgaureae|MM03173|658[0n]bp|Finland.Northern Ostrobothnia|BOLD:AAB3183
Coleophora virgaureae|MM03174|658[0n]bp|Finland.Northern Ostrobothnia|BOLD:AAB3183
Coleophora virgaureae|MM17961|658[0n]bp|Finland|BOLD:AAB3183
Coleophora virgaureae|MM10165|658[0n]bp|Finland|BOLD:AAB3183
Coleophora virgaureae|MM18194|658[0n]bp|Finland|BOLD:AAB3183
Coleophora virgaureae|MM03175|658[0n]bp|Finland.Northern Ostrobothnia|BOLD:AAB3183
Coleophora virgaureae|MM04170|658[0n]bp|Finland.Lapland|BOLD:AAB3183
Coleophora virgaureae|TLMF Lep 09188|658[0n]bp|Austria.Vorarlberg|BOLD:AAB3183
Coleophora virgaureae|MM03172|658[0n]bp|Finland.Northern Ostrobothnia|BOLD:AAB3183
Coleophora virgaureae|MM04169|658[0n]bp|Finland.Lapland|BOLD:AAB3183
Coleophora virgaureae|MM02512|658[0n]bp|Finland.South Karelia|BOLD:AAB3183
Coleophora virgaureae|MM10164|649[0n]bp|Finland|BOLD:AAB3183
Coleophora virgaureae|MM09827|569[0n]bp|Finland.Aland Islands|BOLD:AAB3183
Coleophora virgaureae|MM02493|658[0n]bp|Finland.South Karelia|BOLD:AAB3183
Coleophora virgaureae|MM02492|658[0n]bp|Finland.South Karelia|BOLD:AAB3183
Miltochrista miniata|MM11636|658[0n]bp|Finland|BOLD:AAC4781
Miltochrista miniata|MM01027|658[0n]bp|Finland.South Karelia|BOLD:AAC4781
Miltochrista miniata|TLMF Lep 08112|658[0n]bp|Austria.Vorarlberg|BOLD:AAC4781
Miltochrista miniata|MM01028|658[0n]bp|Finland.South Karelia|BOLD:AAC4781
Anacampsis blattariella|MM02324|658[0n]bp|Finland.South Karelia|BOLD:AAC9810
Anacampsis blattariella|MM08998|658[0n]bp|Finland|BOLD:AAC9810
Anacampsis blattariella|BIOUG04490-F06|619[0n]bp|Finland.Northern Ostrobothnia|BOLD:AAC9810
Anacampsis blattariella|MM03074|621[0n]bp|Finland.Northern Ostrobothnia|BOLD:AAC9810
Anacampsis blattariella|BIOUG04490-G01|638[0n]bp|Finland.Northern Ostrobothnia|BOLD:AAC9810
Anacampsis blattariella|MM05036|658[0n]bp|Finland.Finland Proper|BOLD:AAC9810
Anacampsis blattariella|MM08997|658[0n]bp|Finland|BOLD:AAC9810

Anacampsis blattariella|BIOUG04490-G01|638[0n]bp|Finland.Northern Ostrobothnia|BOLD:AAC9810
Anacampsis blattariella|MM05036|658[0n]bp|Finland.Finland Proper|BOLD:AAC9810
Anacampsis blattariella|MM08997|658[0n]bp|Finland|BOLD:AAC9810
Anacampsis populella|MM03073|670[0n]bp|Finland.Northern Ostrobothnia|BOLD:AAD3256
Anacampsis populella|TLMF Lep 08201|631[0n]bp|Austria.Vorarlberg|BOLD:AAD3256
Anacampsis populella|MM03644|658[0n]bp|Finland.Uusimaa|BOLD:AAD3256
Anacampsis blattariella|TLMF Lep 08438|658[0n]bp|Austria.Vorarlberg|BOLD:AAD3256
Anacampsis populella|MM13400|629[0n]bp|Finland|BOLD:AAD3256
Mompha conturbatella|MM09001|658[0n]bp|Finland|BOLD:AAB7570
Mompha conturbatella|MM08028|658[0n]bp|Finland|BOLD:AAB7570
Mompha conturbatella|MM00062|610[0n]bp|Finland.Northern Ostrobothnia|BOLD:AAB7570
Mompha conturbatella|MM02306|670[0n]bp|Finland.South Karelia|BOLD:AAB7570
Mompha conturbatella|TLMF Lep 09392|658[0n]bp|Austria.Vorarlberg|BOLD:AAB7570
Mompha lacteella|MM06972|658[0n]bp|Finland|BOLD:AAB9184
Mompha lacteella|MM17941|658[0n]bp|Finland|BOLD:AAB9184
Mompha lacteella|MM06971|658[0n]bp|Finland|BOLD:AAB9184
Mompha lacteella|TLMF Lep 09170|658[0n]bp|Austria.Vorarlberg|BOLD:AAB9184
Mompha subbistrigella|MM15601|658[1n]bp|Finland|BOLD:AAD0702
Mompha subbistrigella|TLMF Lep 09182|614[0n]bp|Austria.Vorarlberg|BOLD:AAD0702
Mompha subbistrigella|MM23198|658[0n]bp|Finland|BOLD:AAD0702
Mompha subbistrigella|TLMF Lep 08051|658[0n]bp|Austria.Vorarlberg|BOLD:AAD0702
Mompha subbistrigella|MM15600|658[1n]bp|Finland|BOLD:AAD0702
Mompha subbistrigella|MM17563|658[0n]bp|Finland|BOLD:AAD0702
Mompha subbistrigella|MM21101|658[0n]bp|Finland|BOLD:AAD0702
Dichomeris alacella|TLMF Lep 08444|658[0n]bp|Austria.Vorarlberg|BOLD:AAF1347
Dichomeris alacella|MM04336|658[0n]bp|Finland|BOLD:AAF1347
Dichomeris alacella|MM06878|658[0n]bp|Finland.Aland Islands|BOLD:AAF1347
Dichomeris alacella|MM02572|639[0n]bp|Finland.South Karelia|BOLD:AAF1347
Dichomeris latipennella|MM09839|658[0n]bp|Finland|BOLD:AAF1370
Dichomeris latipennella|MM18252|658[0n]bp|Finland|BOLD:AAF1370
Dichomeris latipennella|MM09840|658[0n]bp|Finland|BOLD:AAF1370
Dichomeris latipennella|TLMF Lep 09961|658[0n]bp|Austria.Vorarlberg|BOLD:AAF1370
Scythris laminella|TLMF Lep 12528|658[0n]bp|Austria.Vorarlberg|BOLD:AAJ7286
Scythris laminella|MM15608|658[0n]bp|Finland|BOLD:AAJ7286
Scythris laminella|MM17301|658[0n]bp|Finland|BOLD:AAJ7286
Scythris laminella|MM17302|658[0n]bp|Finland|BOLD:AAJ7286
Emmelina monodactyla|TLMF Lep 08009|658[0n]bp|Austria.Vorarlberg|BOLD:ACE4862
Emmelina monodactyla|TLMF Lep 08421|658[0n]bp|Austria.Vorarlberg|BOLD:ACE4862
Emmelina monodactyla|MM09764|658[0n]bp|Finland.Aland Islands|BOLD:ACE4862
Emmelina monodactyla|MM04982|658[0n]bp|Finland.Finland Proper|BOLD:ACE4862
Emmelina monodactyla|MM13389|658[0n]bp|Finland|BOLD:ACE4862
Recurvaria leucatella|TLMF Lep 08296|658[0n]bp|Austria.Vorarlberg|BOLD:AAD6088
Recurvaria leucatella|TLMF Lep 08220|658[1n]bp|Austria.Vorarlberg|BOLD:AAD6088
Recurvaria leucatella|MM05493|658[0n]bp|Finland.Aland Islands|BOLD:AAD6088
Recurvaria leucatella|MM05044|632[0n]bp|Finland.Finland Proper|BOLD:AAD6088
Recurvaria leucatella|MM02317|658[0n]bp|Finland.South Karelia|BOLD:AAD6088
Teleiodes flavimaculella|MM09223|658[0n]bp|Finland|BOLD:AAH9847
Teleiodes flavimaculella|MM17574|658[0n]bp|Finland|BOLD:AAH9847
Teleiodes flavimaculella|MM10491|658[0n]bp|Finland|BOLD:AAH9847
Teleiodes flavimaculella|TLMF Lep 07946|658[0n]bp|Austria.Vorarlberg|BOLD:AAH9847
Teleiodes luculella|MM09775|658[0n]bp|Finland.Aland Islands|BOLD:AAD2632
Teleiodes luculella|TLMF Lep 08050|658[0n]bp|Austria.Vorarlberg|BOLD:AAD2632
Teleiodes luculella|MM18229|658[0n]bp|Finland|BOLD:AAD2632
Teleiodes luculella|MM09792|658[0n]bp|Finland.Aland Islands|BOLD:AAD2632
Psoricoptera gibbosella|TLMF Lep 08236|615[0n]bp|Austria.Vorarlberg|BOLD:AAD0608
Psoricoptera gibbosella|MM05059|658[0n]bp|Finland.Finland Proper|BOLD:AAD0608
Psoricoptera gibbosella|MM05057|658[0n]bp|Finland.Finland Proper|BOLD:AAD0608
Psoricoptera gibbosella|MM05056|568[0n]bp|Finland.Finland Proper|BOLD:AAD0608
Psoricoptera gibbosella|MM05058|658[0n]bp|Finland.Finland Proper|BOLD:AAD0608
Teleiodes vulgella|TLMF Lep 08053|658[0n]bp|Austria.Vorarlberg|BOLD:AAE9855
Teleiodes vulgella|MM12470|658[0n]bp|Finland|BOLD:AAE9855
Teleiodes vulgella|MM13610|658[0n]bp|Finland|BOLD:AAE9855
Teleiodes vulgella|MM10490|658[0n]bp|Finland|BOLD:AAE9855
Carpatolechia alburnella|MM03556|658[0n]bp|Finland.Kymenlaakso|BOLD:AAF1850
Carpatolechia alburnella|TLMF Lep 12468|658[0n]bp|Austria.Vorarlberg|BOLD:AAF1850
Carpatolechia alburnella|MM12437|658[0n]bp|Finland|BOLD:AAF1850
Carpatolechia alburnella|MM09805|658[0n]bp|Finland.Aland Islands|BOLD:AAF1850
Carpatolechia fugitivella|TLMF Lep 08076|658[0n]bp|Austria.Vorarlberg|BOLD:AAA7652
Carpatolechia fugitivella|MM15625|658[0n]bp|Finland|BOLD:AAA7652
Carpatolechia fugitivella|TLMF Lep 08298|658[0n]bp|Austria.Vorarlberg|BOLD:AAA7652
Carpatolechia fugitivella|MM15624|658[0n]bp|Finland|BOLD:AAA7652
Carpatolechia fugitivella|MM12021|658[0n]bp|Finland|BOLD:AAA7652
Teleiodes wagae|TLMF Lep 08061|658[0n]bp|Austria.Vorarlberg|BOLD:AAV7088
Teleiodes wagae|MM17710|658[0n]bp|Finland|BOLD:AAV7088
Teleiodes wagae|MM17711|584[0n]bp|Finland|BOLD:AAV7088
Mompha langiella|TLMF Lep 12494|658[0n]bp|Austria.Vorarlberg|BOLD:AAF0121
Mompha langiella|MM17395|658[0n]bp|Finland|BOLD:AAF0121
Mompha langiella|MM05970|658[0n]bp|Finland.Aland Islands|BOLD:AAF0121
Mompha langiella|MM04343|658[0n]bp|Finland.Finland Proper|BOLD:AAF0121
Mompha langiella|MM04342|649[0n]bp|Finland.Finland Proper|BOLD:AAF0121
Pseudatemelia josephinae|BIOUG04116-A05|632[0n]bp|Finland.Northern Ostrobothnia|BOLD:AAC3200
Pseudatemelia josephinae|MM09816|658[0n]bp|Finland.Aland Islands|BOLD:AAC3200
Pseudatemelia josephinae|BIOUG04118-E06|630[0n]bp|Finland.Northern Ostrobothnia|BOLD:AAC3200
Pseudatemelia josephinae|MM13333|636[0n]bp|Finland|BOLD:AAC3200
Pseudatemelia josephinae|MM11940|658[0n]bp|Finland|BOLD:AAC3200
Pseudatemelia josephinae|MM05190|658[0n]bp|Finland.Finland Proper|BOLD:AAC3200
Pseudatemelia josephinae|MM05031|658[0n]bp|Finland.Finland Proper|BOLD:AAC3200
Pseudatemelia josephinae|MM08032|658[0n]bp|Finland|BOLD:AAC3200
Pseudatemelia josephinae|MM09249|658[0n]bp|Finland|BOLD:AAC3200
Pseudatemelia josephinae|MM03049|658[0n]bp|Finland.Northern Ostrobothnia|BOLD:AAC3200
Pseudatemelia josephinae|MM00066|670[0n]bp|Finland.Northern Ostrobothnia|BOLD:AAC3200
Pseudatemelia josephinae|MM03048|648[0n]bp|Finland.Northern Ostrobothnia|BOLD:AAC3200
Pseudatemelia josephinae|MM00037|609[0n]bp|Finland.Northern Ostrobothnia|BOLD:AAC3200
Pseudatemelia josephinae|BIOUG04116-A04|637[0n]bp|Finland.Northern Ostrobothnia|BOLD:AAC3200
Pseudatemelia josephinae|MM02514|658[0n]bp|Finland.South Karelia|BOLD:AAC3200
Pseudatemelia josephinae|MM02519|658[0n]bp|Finland.South Karelia|BOLD:AAC3200
Pseudatemelia josephinae|MM02513|658[0n]bp|Finland.South Karelia|BOLD:AAC3200
Pseudatemelia josephinae|MM14391|658[0n]bp|Finland.Aland Islands|BOLD:AAC3200
Pseudatemelia josephinae|MM06757|658[0n]bp|Finland.Aland Islands|BOLD:AAC3200
Pseudatemelia josephinae|TLMF Lep 08253|658[0n]bp|Austria.Vorarlberg|BOLD:AAC3200
Limnaecia phragmitella|MM00333|670[0n]bp|Finland.Uusimaa|BOLD:AAA7368
Limnaecia phragmitella|TLMF Lep 12467|658[0n]bp|Austria.Vorarlberg|BOLD:AAA7368
[illegible]

Pseudatemelia josephinae|TLMF Lep 08253|658[0n]bp|Austria.Vorarlberg|BOLD:AAC3200
Limnaecia phragmitella|MM00333|670[0n]bp|Finland.Uusimaa|BOLD:AAA7368
Limnaecia phragmitella|TLMF Lep 12467|658[0n]bp|Austria.Vorarlberg|BOLD:AAA7368
Limnaecia phragmitella|MM08567|658[0n]bp|Finland|BOLD:AAA7368
Limnaecia phragmitella|MM13549|658[0n]bp|Finland|BOLD:AAA7368
Limnaecia phragmitella|MM06831|658[0n]bp|Finland.Aland Islands|BOLD:AAA7368
Pleurota bicostella|TLMF Lep 09927|658[0n]bp|Austria.Vorarlberg|BOLD:AAD4880
Pleurota bicostella|MM00059|670[0n]bp|Finland.Northern Ostrobothnia|BOLD:AAD4880
Pleurota bicostella|MM00060|658[0n]bp|Finland.Northern Ostrobothnia|BOLD:AAD4880
Pleurota bicostella|MM00061|658[0n]bp|Finland.Northern Ostrobothnia|BOLD:AAD4880
Athrips mouffetella|TLMF Lep 10045|658[0n]bp|Austria.Vorarlberg|BOLD:AAC9993
Athrips mouffetella|MM09836|658[0n]bp|Finland.Aland Islands|BOLD:AAC9993
Athrips mouffetella|MM05061|658[0n]bp|Finland.Finland Proper|BOLD:AAC9993
Athrips mouffetella|MM09777|658[0n]bp|Finland.Aland Islands|BOLD:AAC9993
Zeuzera pyrina|MM18950|658[0n]bp|Finland.Aland Islands|BOLD:AAD8444
Zeuzera pyrina|TLMF Lep 08133|658[0n]bp|Austria.Vorarlberg|BOLD:AAD8444
Elachista albidella|MM16213|658[0n]bp|Finland.Paijanne Tavastia|BOLD:ACF5411
Elachista albidella|MM16209|655[0n]bp|Finland.Paijanne Tavastia|BOLD:ACF5411
Elachista albidella|MM03912|658[0n]bp|Finland.Northern Ostrobothnia|BOLD:ABX6815
Elachista albidella|MM06596|658[0n]bp|Finland|BOLD:ABX6815
Elachista albidella|MM06597|658[0n]bp|Finland|BOLD:ABX6815
Elachista albidella|TLMF Lep 09966|658[0n]bp|Austria.Vorarlberg|BOLD:AAE9972
Elachista albidella|MM15572|658[1n]bp|Finland|BOLD:AAE9972
Elachista albidella|MM15571|656[1n]bp|Finland|BOLD:AAE9972
Elachista albidella|MM03911|658[0n]bp|Finland.Northern Ostrobothnia|BOLD:AAE9972
Elachista albidella|MM17964|658[0n]bp|Finland|BOLD:AAE9972
Elachista albidella|MM10432|658[0n]bp|Finland|BOLD:AAE9972
Elachista albidella|MM16211|658[0n]bp|Finland.Paijanne Tavastia|BOLD:AAE9972
Elachista albidella|MM17554|652[0n]bp|Finland|BOLD:AAE9972
Elachista albidella|MM03237|632[0n]bp|Finland.Northern Ostrobothnia|BOLD:AAE9972
Macaria brunneata|MM08082|658[0n]bp|Finland|BOLD:ABY9522
Macaria brunneata|TLMF Lep 06170|658[0n]bp|Austria.Vorarlberg|BOLD:ABY9522
Macaria brunneata|MM01455|658[0n]bp|Finland.South Karelia|BOLD:ABY9522
Macaria brunneata|MM01456|658[0n]bp|Finland.South Karelia|BOLD:ABY9522
Macaria fusca|TLMF Lep 12551|658[0n]bp|Austria.Vorarlberg|BOLD:AAC7749
Macaria fusca|TLMF Lep 09503|658[0n]bp|Austria.Vorarlberg|BOLD:AAC7749
Macaria fusca|MM14587|658[0n]bp|Finland|BOLD:AAC7749
Macaria fusca|MM03420|658[0n]bp|Finland.Lapland|BOLD:AAC7749
Macaria fusca|MM18508|658[0n]bp|Finland|BOLD:AAC7749
Macaria alternata|TLMF Lep 08540|658[0n]bp|Austria.Vorarlberg|BOLD:AAA1496
Macaria alternata|MM01334|658[0n]bp|Finland.South Karelia|BOLD:AAA1496
Macaria alternata|MM11578|633[0n]bp|Finland|BOLD:AAA1496
Macaria alternata|MM01333|632[0n]bp|Finland.South Karelia|BOLD:AAA1496
Macaria liturata|MM01481|658[0n]bp|Finland.South Karelia|BOLD:ACF0383
Macaria liturata|MM01480|622[0n]bp|Finland.South Karelia|BOLD:ACF4721
Macaria liturata|MM04865|658[0n]bp|Finland.Finland Proper|BOLD:ABX6221
Macaria liturata|TLMF Lep 07869|658[0n]bp|Austria.Vorarlberg|BOLD:ABX6221
Macaria signaria|TLMF Lep 04657|658[0n]bp|Austria.Vorarlberg|BOLD:AAA1255
Macaria signaria|MM06029|658[0n]bp|Finland|BOLD:AAA1255
Macaria signaria|MM14253|658[0n]bp|Finland.Aland Islands|BOLD:AAA1255
Macaria signaria|MM14051|658[0n]bp|Finland|BOLD:AAA1255
Crocallis elinguaria|MM02805|651[0n]bp|Finland.Northern Ostrobothnia|BOLD:AAB0677
Crocallis elinguaria|MM01367|658[0n]bp|Finland.South Karelia|BOLD:AAB0677
Crocallis elinguaria|MM01368|658[0n]bp|Finland.South Karelia|BOLD:AAB0677
Crocallis elinguaria|TLMF Lep 06123|658[0n]bp|Austria.Vorarlberg|BOLD:AAB0677
Cryptoblabes bistriga|MM10529|658[0n]bp|Finland|BOLD:AAD6231
Cryptoblabes bistriga|TLMF Lep 08085|658[0n]bp|Austria.Vorarlberg|BOLD:AAD6231
Cryptoblabes bistriga|MM15769|658[0n]bp|Finland|BOLD:AAD6231
Cryptoblabes bistriga|TLMF Lep 09924|658[0n]bp|Austria.Vorarlberg|BOLD:AAD6231
Cryptoblabes bistriga|MM15770|658[0n]bp|Finland|BOLD:AAD6231
Cossus cossus|TLMF Lep 07551|658[0n]bp|Austria.Vorarlberg|BOLD:AAE4069
Cossus cossus|MM00116|670[0n]bp|Finland.Uusimaa|BOLD:ACE8991
Cossus cossus|MM06801|658[0n]bp|Finland.Aland Islands|BOLD:ACE8991
Cossus cossus|MM00354|658[0n]bp|Finland.Lapland|BOLD:ACE8991
Epermenia chaerophyllella|MM14007|658[0n]bp|Finland|BOLD:AAC9729
Epermenia chaerophyllella|MM09752|658[0n]bp|Finland|BOLD:AAC9729
Epermenia chaerophyllella|TLMF Lep 08211|615[0n]bp|Austria.Vorarlberg|BOLD:AAC9729
Epermenia chaerophyllella|MM09795|658[0n]bp|Finland.Aland Islands|BOLD:AAC9729
Epermenia chaerophyllella|MM00443|658[0n]bp|Finland|BOLD:AAC9729
Phaulernis fulviguttella|MM18904|658[0n]bp|Finland.Aland Islands|BOLD:AAI4589
Phaulernis fulviguttella|MM14645|658[0n]bp|Finland|BOLD:AAI4589
Phaulernis fulviguttella|MM15751|658[0n]bp|Finland|BOLD:AAI4589
Phaulernis fulviguttella|MM11049|637[0n]bp|Finland|BOLD:AAI4589
Phaulernis fulviguttella|TLMF Lep 04306|658[0n]bp|Austria.Vorarlberg|BOLD:AAI4589
Adaina microdactyla|MM05682|658[0n]bp|Finland.Aland Islands|BOLD:AAD2831
Adaina microdactyla|TLMF Lep 08451|658[0n]bp|Austria.Vorarlberg|BOLD:AAD2831
Adaina microdactyla|MM11165|658[0n]bp|Finland.Aland Islands|BOLD:AAD2831
Adaina microdactyla|TLMF Lep 09198|658[0n]bp|Austria.Vorarlberg|BOLD:AAD2831
Hellinsia osteodactylus|TLMF Lep 04287|658[0n]bp|Austria.Vorarlberg|BOLD:AAD2930
Hellinsia osteodactylus|MM13383|589[0n]bp|Finland|BOLD:AAD2930
Hellinsia osteodactylus|MM08472|632[3n]bp|Finland|BOLD:AAD2930
Hellinsia osteodactylus|MM06357|658[0n]bp|Finland|BOLD:AAD2930
Hellinsia osteodactylus|MM03105|658[0n]bp|Finland.Northern Ostrobothnia|BOLD:AAD2930
Merrifieldia leucodactyla|TLMF Lep 00910|658[0n]bp|Austria.Vorarlberg|BOLD:AAE1533
Merrifieldia leucodactyla|MM10051|658[0n]bp|Finland|BOLD:AAE1533
Merrifieldia leucodactyla|MM06254|658[0n]bp|Finland|BOLD:AAE1533
Merrifieldia leucodactyla|MM17337|658[0n]bp|Finland|BOLD:AAE1533
Oidaematophorus lithodactyla|MM10393|658[0n]bp|Finland.Aland Islands|BOLD:AAD8626
Oidaematophorus lithodactyla|MM09587|658[0n]bp|Finland.Aland Islands|BOLD:AAD8626
Oidaematophorus lithodactyla|MM09588|658[0n]bp|Finland.Aland Islands|BOLD:AAD8626
Oidaematophorus lithodactyla|TLMF Lep 07513|658[0n]bp|Austria.Vorarlberg|BOLD:AAD8626
Hellinsia tephradactyla|MM03104|658[0n]bp|Finland.Northern Ostrobothnia|BOLD:AAE6943
Hellinsia tephradactyla|BIOUG04116-B08|632[0n]bp|Finland.Northern Ostrobothnia|BOLD:AAE6943
Hellinsia tephradactyla|TLMF Lep 08396|658[0n]bp|Austria.Vorarlberg|BOLD:AAE6943
Hellinsia tephradactyla|MM18377|658[0n]bp|Finland|BOLD:AAE6943
Hellinsia tephradactyla|MM03103|658[0n]bp|Finland.Northern Ostrobothnia|BOLD:AAE6943
Oidaematophorus rogenhoferi|MM06393|658[0n]bp|Finland|BOLD:AAE5830
Oidaematophorus rogenhoferi|MM06583|658[0n]bp|Finland|BOLD:AAE5830
Oidaematophorus rogenhoferi|MM06582|658[0n]bp|Finland|BOLD:AAE5830
Oidaematophorus rogenhoferi|TLMF Lep 08747|658[0n]bp|Austria.Vorarlberg|BOLD:AAE5830
Marasmarcha lunaedactyla|TLMF Lep 08008|658[0n]bp|Austria.Vorarlberg|BOLD:ABX6234
Marasmarcha lunaedactyla|MM11051|658[0n]bp|Finland|BOLD:AAL6978
Marasmarcha lunaedactyla|MM11052|658[0n]bp|Finland|BOLD:AAL6978

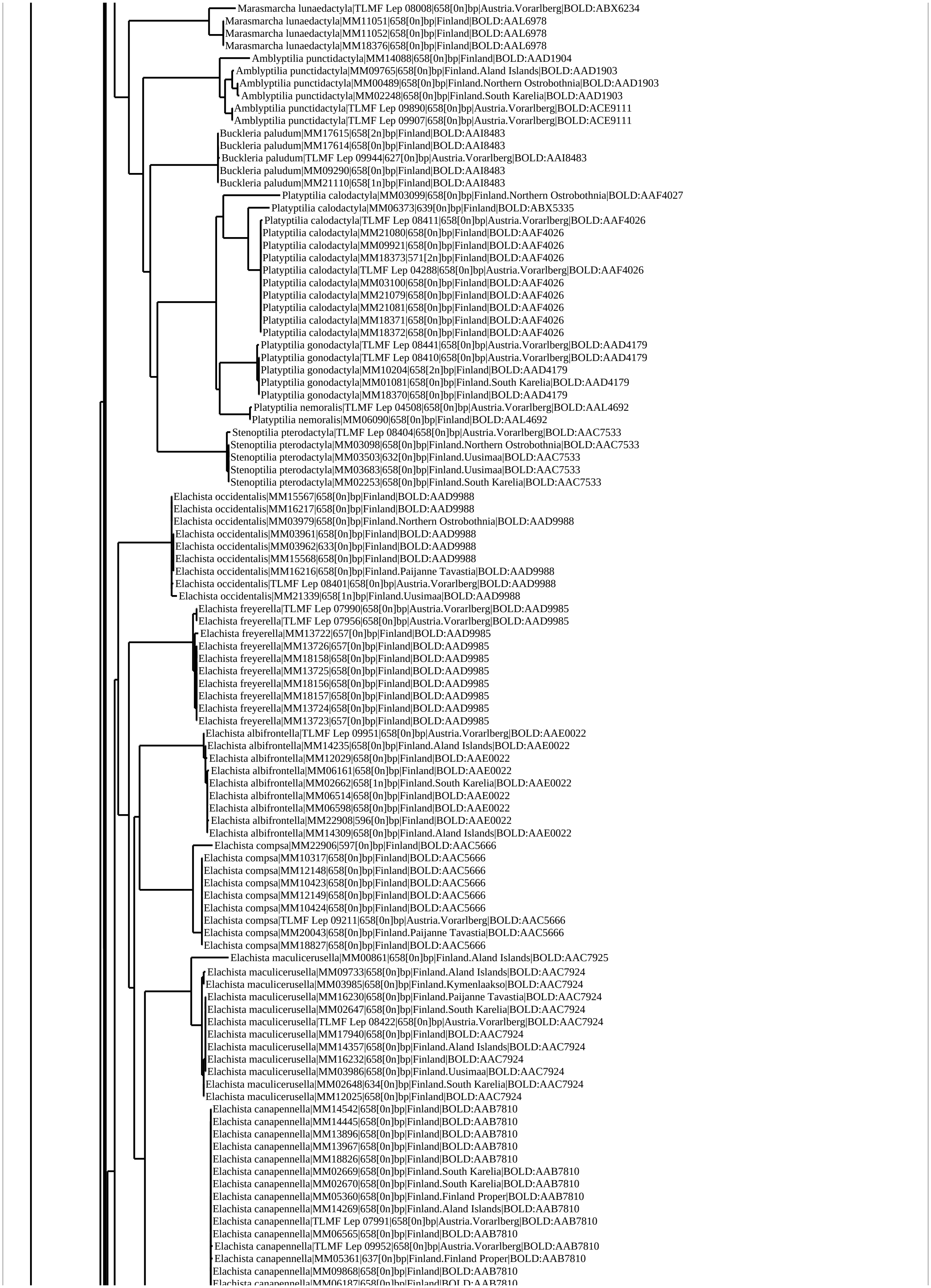
Marasmarcha lunaedactyla|TLMF Lep 08008|658[0n]bp|Austria.Vorarlberg|BOLD:ABX6234
Marasmarcha lunaedactyla|MM11051|658[0n]bp|Finland|BOLD:AAL6978
Marasmarcha lunaedactyla|MM11052|658[0n]bp|Finland|BOLD:AAL6978
Marasmarcha lunaedactyla|MM18376|658[0n]bp|Finland|BOLD:AAL6978
Amblyptilia punctidactyla|MM14088|658[0n]bp|Finland|BOLD:AAD1904
Amblyptilia punctidactyla|MM09765|658[0n]bp|Finland.Aland Islands|BOLD:AAD1903
Amblyptilia punctidactyla|MM00489|658[0n]bp|Finland.Northern Ostrobothnia|BOLD:AAD1903
Amblyptilia punctidactyla|MM02248|658[0n]bp|Finland.South Karelia|BOLD:AAD1903
Amblyptilia punctidactyla|TLMF Lep 09890|658[0n]bp|Austria.Vorarlberg|BOLD:ACE9111
Amblyptilia punctidactyla|TLMF Lep 09907|658[0n]bp|Austria.Vorarlberg|BOLD:ACE9111
Buckleria paludum|MM17615|658[2n]bp|Finland|BOLD:AAI8483
Buckleria paludum|MM17614|658[0n]bp|Finland|BOLD:AAI8483
Buckleria paludum|TLMF Lep 09944|627[0n]bp|Austria.Vorarlberg|BOLD:AAI8483
Buckleria paludum|MM09290|658[0n]bp|Finland|BOLD:AAI8483
Buckleria paludum|MM21110|658[1n]bp|Finland|BOLD:AAI8483
Platyptilia calodactyla|MM03099|658[0n]bp|Finland.Northern Ostrobothnia|BOLD:AAF4027
Platyptilia calodactyla|MM06373|639[0n]bp|Finland|BOLD:ABX5335
Platyptilia calodactyla|TLMF Lep 08411|658[0n]bp|Austria.Vorarlberg|BOLD:AAF4026
Platyptilia calodactyla|MM21080|658[0n]bp|Finland|BOLD:AAF4026
Platyptilia calodactyla|MM09921|658[0n]bp|Finland|BOLD:AAF4026
Platyptilia calodactyla|MM18373|571[2n]bp|Finland|BOLD:AAF4026
Platyptilia calodactyla|TLMF Lep 04288|658[0n]bp|Austria.Vorarlberg|BOLD:AAF4026
Platyptilia calodactyla|MM03100|658[0n]bp|Finland|BOLD:AAF4026
Platyptilia calodactyla|MM21079|658[0n]bp|Finland|BOLD:AAF4026
Platyptilia calodactyla|MM21081|658[0n]bp|Finland|BOLD:AAF4026
Platyptilia calodactyla|MM18371|658[0n]bp|Finland|BOLD:AAF4026
Platyptilia calodactyla|MM18372|658[0n]bp|Finland|BOLD:AAF4026
Platyptilia gonodactyla|TLMF Lep 08441|658[0n]bp|Austria.Vorarlberg|BOLD:AAD4179
Platyptilia gonodactyla|TLMF Lep 08410|658[0n]bp|Austria.Vorarlberg|BOLD:AAD4179
Platyptilia gonodactyla|MM10204|658[2n]bp|Finland|BOLD:AAD4179
Platyptilia gonodactyla|MM01081|658[0n]bp|Finland.South Karelia|BOLD:AAD4179
Platyptilia gonodactyla|MM18370|658[0n]bp|Finland|BOLD:AAD4179
Platyptilia nemoralis|TLMF Lep 04508|658[0n]bp|Austria.Vorarlberg|BOLD:AAL4692
Platyptilia nemoralis|MM06090|658[0n]bp|Finland|BOLD:AAL4692
Stenoptilia pterodactyla|TLMF Lep 08404|658[0n]bp|Austria.Vorarlberg|BOLD:AAC7533
Stenoptilia pterodactyla|MM03098|658[0n]bp|Finland.Northern Ostrobothnia|BOLD:AAC7533
Stenoptilia pterodactyla|MM03503|632[0n]bp|Finland.Uusimaa|BOLD:AAC7533
Stenoptilia pterodactyla|MM03683|658[0n]bp|Finland.Uusimaa|BOLD:AAC7533
Stenoptilia pterodactyla|MM02253|658[0n]bp|Finland.South Karelia|BOLD:AAC7533
Elachista occidentalis|MM15567|658[0n]bp|Finland|BOLD:AAD9988
Elachista occidentalis|MM16217|658[0n]bp|Finland|BOLD:AAD9988
Elachista occidentalis|MM03979|658[0n]bp|Finland.Northern Ostrobothnia|BOLD:AAD9988
Elachista occidentalis|MM03961|658[0n]bp|Finland|BOLD:AAD9988
Elachista occidentalis|MM03962|633[0n]bp|Finland|BOLD:AAD9988
Elachista occidentalis|MM15568|658[0n]bp|Finland|BOLD:AAD9988
Elachista occidentalis|MM16216|658[0n]bp|Finland.Paijanne Tavastia|BOLD:AAD9988
Elachista occidentalis|TLMF Lep 08401|658[0n]bp|Austria.Vorarlberg|BOLD:AAD9988
Elachista occidentalis|MM21339|658[1n]bp|Finland.Uusimaa|BOLD:AAD9988
Elachista freyerella|TLMF Lep 07990|658[0n]bp|Austria.Vorarlberg|BOLD:AAD9985
Elachista freyerella|TLMF Lep 07956|658[0n]bp|Austria.Vorarlberg|BOLD:AAD9985
Elachista freyerella|MM13722|657[0n]bp|Finland|BOLD:AAD9985
Elachista freyerella|MM13726|657[0n]bp|Finland|BOLD:AAD9985
Elachista freyerella|MM18158|658[0n]bp|Finland|BOLD:AAD9985
Elachista freyerella|MM13725|658[0n]bp|Finland|BOLD:AAD9985
Elachista freyerella|MM18156|658[0n]bp|Finland|BOLD:AAD9985
Elachista freyerella|MM18157|658[0n]bp|Finland|BOLD:AAD9985
Elachista freyerella|MM13724|658[0n]bp|Finland|BOLD:AAD9985
Elachista freyerella|MM13723|657[0n]bp|Finland|BOLD:AAD9985
Elachista albifrontella|TLMF Lep 09951|658[0n]bp|Austria.Vorarlberg|BOLD:AAE0022
Elachista albifrontella|MM14235|658[0n]bp|Finland.Aland Islands|BOLD:AAE0022
Elachista albifrontella|MM12029|658[0n]bp|Finland|BOLD:AAE0022
Elachista albifrontella|MM06161|658[0n]bp|Finland|BOLD:AAE0022
Elachista albifrontella|MM02662|658[1n]bp|Finland.South Karelia|BOLD:AAE0022
Elachista albifrontella|MM06514|658[0n]bp|Finland|BOLD:AAE0022
Elachista albifrontella|MM06598|658[0n]bp|Finland|BOLD:AAE0022
Elachista albifrontella|MM22908|596[0n]bp|Finland|BOLD:AAE0022
Elachista albifrontella|MM14309|658[0n]bp|Finland.Aland Islands|BOLD:AAE0022
Elachista compsa|MM22906|597[0n]bp|Finland|BOLD:AAC5666
Elachista compsa|MM10317|658[0n]bp|Finland|BOLD:AAC5666
Elachista compsa|MM12148|658[0n]bp|Finland|BOLD:AAC5666
Elachista compsa|MM10423|658[0n]bp|Finland|BOLD:AAC5666
Elachista compsa|MM12149|658[0n]bp|Finland|BOLD:AAC5666
Elachista compsa|MM10424|658[0n]bp|Finland|BOLD:AAC5666
Elachista compsa|TLMF Lep 09211|658[0n]bp|Austria.Vorarlberg|BOLD:AAC5666
Elachista compsa|MM20043|658[0n]bp|Finland.Paijanne Tavastia|BOLD:AAC5666
Elachista compsa|MM18827|658[0n]bp|Finland|BOLD:AAC5666
Elachista maculicerusella|MM00861|658[0n]bp|Finland.Aland Islands|BOLD:AAC7925
Elachista maculicerusella|MM09733|658[0n]bp|Finland.Aland Islands|BOLD:AAC7924
Elachista maculicerusella|MM03985|658[0n]bp|Finland.Kymenlaakso|BOLD:AAC7924
Elachista maculicerusella|MM16230|658[0n]bp|Finland.Paijanne Tavastia|BOLD:AAC7924
Elachista maculicerusella|MM02647|658[0n]bp|Finland.South Karelia|BOLD:AAC7924
Elachista maculicerusella|TLMF Lep 08422|658[0n]bp|Austria.Vorarlberg|BOLD:AAC7924
Elachista maculicerusella|MM17940|658[0n]bp|Finland|BOLD:AAC7924
Elachista maculicerusella|MM14357|658[0n]bp|Finland.Aland Islands|BOLD:AAC7924
Elachista maculicerusella|MM16232|658[0n]bp|Finland|BOLD:AAC7924
Elachista maculicerusella|MM03986|658[0n]bp|Finland.Uusimaa|BOLD:AAC7924
Elachista maculicerusella|MM02648|634[0n]bp|Finland.South Karelia|BOLD:AAC7924
Elachista maculicerusella|MM12025|658[0n]bp|Finland|BOLD:AAC7924
Elachista canapennella|MM14542|658[0n]bp|Finland|BOLD:AAB7810
Elachista canapennella|MM14445|658[0n]bp|Finland|BOLD:AAB7810
Elachista canapennella|MM13896|658[0n]bp|Finland|BOLD:AAB7810
Elachista canapennella|MM13967|658[0n]bp|Finland|BOLD:AAB7810
Elachista canapennella|MM18826|658[0n]bp|Finland|BOLD:AAB7810
Elachista canapennella|MM02669|658[0n]bp|Finland.South Karelia|BOLD:AAB7810
Elachista canapennella|MM02670|658[0n]bp|Finland.South Karelia|BOLD:AAB7810
Elachista canapennella|MM05360|658[0n]bp|Finland.Finland Proper|BOLD:AAB7810
Elachista canapennella|MM14269|658[0n]bp|Finland.Aland Islands|BOLD:AAB7810
Elachista canapennella|TLMF Lep 07991|658[0n]bp|Austria.Vorarlberg|BOLD:AAB7810
Elachista canapennella|MM06565|658[0n]bp|Finland|BOLD:AAB7810
Elachista canapennella|TLMF Lep 09952|658[0n]bp|Austria.Vorarlberg|BOLD:AAB7810
Elachista canapennella|MM05361|637[0n]bp|Finland.Finland Proper|BOLD:AAB7810
Elachista canapennella|MM09868|658[0n]bp|Finland|BOLD:AAB7810
Elachista canapennella|MM06187|658[0n]bp|Finland|BOLD:AAB7810

[illegible]
Elachista canapennella|MM05361|637[0n]bp|Finland.Finland Proper|BOLD:AAB7810
Elachista canapennella|MM09868|658[0n]bp|Finland|BOLD:AAB7810
Elachista canapennella|MM06187|658[0n]bp|Finland|BOLD:AAB7810
Elachista canapennella|MM21092|658[0n]bp|Finland|BOLD:AAB7810
Elachista canapennella|MM06302|658[0n]bp|Finland|BOLD:AAB7810
Elachista canapennella|MM06523|658[0n]bp|Finland|BOLD:AAB7810
Elachista zernyi|MM17654|658[0n]bp|Finland|BOLD:AAP5631
Elachista zernyi|TLMF Lep 10052|658[0n]bp|Austria.Vorarlberg|BOLD:AAP5631
Elachista zernyi|MM22606|658[0n]bp|Finland|BOLD:AAP5631
Elachista adscitella|TLMF Lep 08412|658[0n]bp|Austria.Vorarlberg|BOLD:AAC3846
Elachista adscitella|MM10399|628[0n]bp|Finland.Aland Islands|BOLD:AAC3846
Elachista adscitella|MM02658|658[0n]bp|Finland.South Karelia|BOLD:AAC3846
Elachista adscitella|MM09261|658[0n]bp|Finland|BOLD:AAC3846
Elachista adscitella|MM09737|658[0n]bp|Finland.Aland Islands|BOLD:AAC3846
Elachista adscitella|MM13721|658[0n]bp|Finland|BOLD:AAC3846
Elachista adscitella|MM20018|658[0n]bp|Finland|BOLD:AAC3846
Elachista adscitella|MM12026|658[0n]bp|Finland|BOLD:AAC3846
Elachista adscitella|MM09260|658[0n]bp|Finland|BOLD:AAC3846
Elachista adscitella|MM06727|658[0n]bp|Finland.Aland Islands|BOLD:AAC3846
Elachista adscitella|MM02657|638[0n]bp|Finland.South Karelia|BOLD:AAC3846
Elachista adscitella|MM12027|658[0n]bp|Finland|BOLD:AAC3846
Elachista adscitella|MM20017|658[0n]bp|Finland.Paijanne Tavastia|BOLD:AAC3846
Elachista subalbidella|TLMF Lep 02843|658[0n]bp|Austria.Vorarlberg|BOLD:AAC7905
Elachista subalbidella|TLMF Lep 02844|658[0n]bp|Austria.Vorarlberg|BOLD:AAC7905
Elachista subalbidella|MM05332|572[0n]bp|Finland.Finland Proper|BOLD:AAC7905
Elachista subalbidella|BIOUG04116-A02|609[0n]bp|Finland.Northern Ostrobothnia|BOLD:AAC7905
Elachista subalbidella|MM09037|658[0n]bp|Finland|BOLD:AAC7905
Elachista subalbidella|MM05490|658[0n]bp|Finland.Northern Ostrobothnia|BOLD:AAC7905
Elachista subalbidella|MM16735|658[0n]bp|Finland.Pirkanmaa|BOLD:AAC7905
Elachista subalbidella|MM08055|658[0n]bp|Finland|BOLD:AAC7905
Elachista subalbidella|MM03154|658[0n]bp|Finland.Northern Ostrobothnia|BOLD:AAC7905
Elachista subalbidella|MM20040|640[0n]bp|Finland.Paijanne Tavastia|BOLD:AAC7905
Elachista subalbidella|MM18154|658[0n]bp|Finland|BOLD:AAC7905
Elachista subalbidella|MM02650|658[0n]bp|Finland.South Karelia|BOLD:AAC7905
Elachista subalbidella|MM13727|658[0n]bp|Finland|BOLD:AAC7905
Elachista subalbidella|MM02651|658[0n]bp|Finland.South Karelia|BOLD:AAC7905
Elachista subalbidella|MM06221|658[0n]bp|Finland|BOLD:AAC7905
Elachista subalbidella|MM14064|658[0n]bp|Finland|BOLD:AAC7905
Elachista gleichenella|TLMF Lep 09181|658[0n]bp|Austria.Vorarlberg|BOLD:ACF5537
Elachista gleichenella|TLMF Lep 10047|658[0n]bp|Austria.Vorarlberg|BOLD:AAD8163
Elachista gleichenella|MM06595|658[0n]bp|Finland|BOLD:AAD8163
Elachista gleichenella|MM09727|658[0n]bp|Finland.Aland Islands|BOLD:AAD8163
Elachista gleichenella|MM03998|527[0n]bp|Finland|BOLD:AAD8163
Elachista gleichenella|MM06164|658[0n]bp|Finland|BOLD:AAD8163
Elachista gleichenella|MM09265|658[0n]bp|Finland|BOLD:AAD8163
Elachista gleichenella|MM03999|658[0n]bp|Finland.Aland Islands|BOLD:AAD8163
Perittia farinella|MM06229|658[0n]bp|Finland|BOLD:AAL2425
Perittia farinella|MM06230|658[0n]bp|Finland|BOLD:AAL2425
Perittia farinella|MM08383|658[0n]bp|Finland|BOLD:AAL2425
Perittia farinella|TLMF Lep 07468|658[0n]bp|Austria.Vorarlberg|BOLD:AAL2425
Cochylidia subroseana|TLMF Lep 09187|658[1n]bp|Austria.Vorarlberg|BOLD:AAD2823
Cochylidia subroseana|MM02558|658[0n]bp|Finland.South Karelia|BOLD:AAD2823
Cochylidia subroseana|MM14118|658[0n]bp|Finland|BOLD:AAD2823
Cochylidia subroseana|MM02991|658[0n]bp|Finland.Northern Ostrobothnia|BOLD:AAD2823
Cochylidia subroseana|TLMF Lep 00869|658[0n]bp|Austria.Vorarlberg|BOLD:AAD2823
Cochylis flaviciliana|TLMF Lep 07701|557[1n]bp|Austria|BOLD:ABA2672
Cochylis flaviciliana|MM06608|658[0n]bp|Finland|BOLD:AAE8888
Cochylis flaviciliana|MM06758|658[0n]bp|Finland.Aland Islands|BOLD:AAE8888
Cochylis flaviciliana|MM06891|658[0n]bp|Finland.Aland Islands|BOLD:AAE8888
Cochylis dubitana|MM04171|658[0n]bp|Finland.Lapland|BOLD:AAC3780
Cochylis dubitana|TLMF Lep 09169|658[0n]bp|Austria.Vorarlberg|BOLD:AAC3780
Cochylis dubitana|MM10346|658[0n]bp|Finland|BOLD:AAC3780
Cochylis dubitana|MM04172|658[1n]bp|Finland.Lapland|BOLD:AAC3780
Cochylis dubitana|MM13286|658[0n]bp|Finland|BOLD:AAC3780
Cochylis dubitana|MM03037|504[5n]bp|Finland.Northern Ostrobothnia|BOLD:AAC3780
Cochylis dubitana|MM11845|504[1n]bp|Finland|BOLD:AAC3780
Cochylis dubitana|MM06126|658[0n]bp|Finland|BOLD:AAC3780
Cochylis dubitana|MM02551|658[0n]bp|Finland.South Karelia|BOLD:AAC3780
Cochylis nana|TLMF Lep 09954|658[0n]bp|Austria.Vorarlberg|BOLD:ABY5998
Cochylis nana|MM14110|658[0n]bp|Finland|BOLD:ABY5998
Cochylis nana|MM03201|658[0n]bp|Finland.Northern Ostrobothnia|BOLD:ABY5998
Cochylis nana|MM05291|641[0n]bp|Finland.Finland Proper|BOLD:ABY5998
Cochylis pallidana|MM06249|658[0n]bp|Finland|BOLD:AAL6146
Cochylis pallidana|MM06762|658[0n]bp|Finland.Aland Islands|BOLD:AAE1297
Cochylis pallidana|MM13289|658[0n]bp|Finland|BOLD:AAE1297
Cochylis pallidana|MM06248|658[0n]bp|Finland|BOLD:AAE1297
Cochylis pallidana|MM17588|658[0n]bp|Finland|BOLD:AAE1297
Cochylis pallidana|TLMF Lep 07482|658[0n]bp|Austria.Vorarlberg|BOLD:AAE1297
Cochylis pallidana|MM02553|658[0n]bp|Finland.South Karelia|BOLD:AAE1297
Cochylis pallidana|TLMF Lep 09155|658[0n]bp|Austria.Vorarlberg|BOLD:AAE1297
Cochylis pallidana|TLMF Lep 08428|658[0n]bp|Austria.Vorarlberg|BOLD:AAE1297
Falseuncaria ruficiliana|TLMF Lep 07514|658[2n]bp|Austria.Vorarlberg|BOLD:AAD2822
Falseuncaria ruficiliana|TLMF Lep 08046|658[0n]bp|Austria.Vorarlberg|BOLD:AAD2822
Falseuncaria ruficiliana|MM13300|658[0n]bp|Finland|BOLD:AAD2822
Falseuncaria ruficiliana|MM09521|658[0n]bp|Finland.Aland Islands|BOLD:AAD2822
Falseuncaria ruficiliana|MM18278|658[0n]bp|Finland|BOLD:AAD2822
Gynnidomorpha alismana|TLMF Lep 08448|658[0n]bp|Austria.Vorarlberg|BOLD:ABY4645
Gynnidomorpha alismana|MM17336|658[0n]bp|Finland|BOLD:AAF7913
Gynnidomorpha alismana|MM18632|658[0n]bp|Finland|BOLD:AAF7913
Gynnidomorpha alismana|MM19266|658[0n]bp|Finland|BOLD:AAF7913
Gynnidomorpha alismana|MM19265|658[0n]bp|Finland|BOLD:AAF7913
Aethes rutilana|MM14379|658[0n]bp|Finland.Aland Islands|BOLD:AAC1488
Aethes rutilana|MM11838|658[0n]bp|Finland|BOLD:AAC1488
Aethes rutilana|TLMF Lep 10043|658[0n]bp|Austria.Vorarlberg|BOLD:AAC1488
Aethes rutilana|MM11837|658[0n]bp|Finland|BOLD:AAC1488
Aethes rutilana|MM18275|658[0n]bp|Finland|BOLD:AAC1488
Aethes rutilana|MM09745|658[0n]bp|Finland.Aland Islands|BOLD:AAC1488
Aethes rutilana|MM08053|658[0n]bp|Finland|BOLD:AAC1488
Aethes rutilana|MM18276|658[0n]bp|Finland|BOLD:AAC1488
Aethes rutilana|MM14588|658[0n]bp|Finland|BOLD:AAC1488
Aethes rutilana|MM03430|658[0n]bp|Finland.Lapland|BOLD:AAC1488
Aethes rutilana|MM00075|658[0n]bp|Finland.Lapland|BOLD:AAC1488
[illegible]

Aethes rutilana|MM14588|658[0n]bp|Finland|BOLD:AAC1488
Aethes rutilana|MM03430|658[0n]bp|Finland.Lapland|BOLD:AAC1488
Aethes rutilana|MM00075|658[0n]bp|Finland.Lapland|BOLD:AAC1488
Aethes hartmanniana|MM06142|658[0n]bp|Finland|BOLD:AAC6718
Aethes hartmanniana|MM17278|635[0n]bp|Finland|BOLD:AAC6718
Aethes hartmanniana|MM05974|658[0n]bp|Finland|BOLD:AAC6718
Aethes hartmanniana|TLMF Lep 08081|658[0n]bp|Austria.Vorarlberg|BOLD:AAC6718
Aethes hartmanniana|MM14121|658[0n]bp|Finland|BOLD:AAC6718
Aethes rubigana|MM06813|658[0n]bp|Finland.Aland Islands|BOLD:AAC2885
Aethes cnicana|MM18274|658[0n]bp|Finland|BOLD:AAC2885
Aethes cnicana|MM02986|658[0n]bp|Finland.Northern Ostrobothnia|BOLD:AAC2885
Aethes cnicana|MM14221|658[0n]bp|Finland.Aland Islands|BOLD:AAC2885
Aethes cnicana|MM01068|670[0n]bp|Finland.South Karelia|BOLD:AAC2885
Aethes cnicana|TLMF Lep 07683|658[0n]bp|Austria|BOLD:AAC2885
Aethes cnicana|TLMF Lep 07684|658[0n]bp|Austria|BOLD:AAC2885
Aethes cnicana|MM01072|658[0n]bp|Finland.South Karelia|BOLD:AAC2885
Aethes cnicana|MM18273|658[0n]bp|Finland|BOLD:AAC2885
Aethes cnicana|MM21121|658[0n]bp|Finland|BOLD:AAC2885
Aethes cnicana|MM01069|658[0n]bp|Finland|BOLD:AAC2885
Aethes cnicana|MM05509|658[0n]bp|Finland|BOLD:AAC2885
Aethes cnicana|MM02987|658[0n]bp|Finland.Northern Ostrobothnia|BOLD:AAC2885
Aethes cnicana|MM01070|658[0n]bp|Finland|BOLD:AAC2885
Aethes cnicana|MM02988|658[0n]bp|Finland|BOLD:AAC2885
Aethes cnicana|MM06710|658[0n]bp|Finland.Aland Islands|BOLD:AAC2885
Aethes cnicana|TLMF Lep 07720|621[0n]bp|Austria|BOLD:AAC2885
Aethes rubigana|MM05199|658[0n]bp|Finland.Finland Proper|BOLD:AAC2885
Aethes rubigana|MM08971|658[0n]bp|Finland|BOLD:AAC2885
Aethes rubigana|TLMF Lep 07682|658[0n]bp|Austria|BOLD:AAC2885
Aethes rubigana|MM12324|658[0n]bp|Finland|BOLD:AAC2885
Aethes rubigana|MM18272|658[0n]bp|Finland|BOLD:AAC2885
Aethes rubigana|MM05200|658[0n]bp|Finland|BOLD:AAC2885
Aethes rubigana|MM18271|658[0n]bp|Finland|BOLD:AAC2885
Aethes rubigana|MM08969|636[0n]bp|Finland|BOLD:AAC2885
Aethes rubigana|MM18270|637[0n]bp|Finland|BOLD:AAC2885
Aethes rubigana|MM08970|658[0n]bp|Finland|BOLD:AAC2885
Aethes smeathmanniana|MM02094|615[0n]bp|Finland.South Karelia|BOLD:AAB1945
Aethes smeathmanniana|TLMF Lep 09981|658[0n]bp|Austria.Vorarlberg|BOLD:AAB1945
Aethes smeathmanniana|MM13141|658[0n]bp|Finland|BOLD:AAB1945
Aethes smeathmanniana|MM13297|658[0n]bp|Finland|BOLD:AAB1945
Aethes smeathmanniana|MM06319|658[0n]bp|Finland|BOLD:AAB1945
Phalonidia gilvicomana|MM19244|519[0n]bp|Finland.Aland Islands|BOLD:AAQ3489
Phalonidia gilvicomana|MM19245|601[3n]bp|Finland.Aland Islands|BOLD:AAQ3489
Phalonidia gilvicomana|TLMF Lep 08226|658[0n]bp|Austria.Vorarlberg|BOLD:AAQ3489
Agapeta hamana|MM06820|658[0n]bp|Finland.Aland Islands|BOLD:AAB9919
Agapeta hamana|TLMF Lep 09163|658[0n]bp|Austria.Vorarlberg|BOLD:AAB9919
Agapeta hamana|MM11780|658[0n]bp|Finland|BOLD:AAB9919
Agapeta zoegana|TLMF Lep 08455|658[0n]bp|Austria.Vorarlberg|BOLD:AAA6575
Agapeta zoegana|MM13171|658[0n]bp|Finland|BOLD:AAA6573
Agapeta zoegana|MM18268|658[0n]bp|Finland|BOLD:AAA6573
Agapeta zoegana|MM09584|634[1n]bp|Finland.Aland Islands|BOLD:AAA6573
Agapeta zoegana|MM06900|658[0n]bp|Finland.Aland Islands|BOLD:AAA6573
Agapeta zoegana|MM12372|658[0n]bp|Finland|BOLD:AAA6573
Eupoecilia ambiguella|MM15673|658[0n]bp|Finland|BOLD:AAD8039
Eupoecilia ambiguella|MM06468|658[0n]bp|Finland|BOLD:AAD8039
Eupoecilia ambiguella|TLMF Lep 07989|658[0n]bp|Austria.Vorarlberg|BOLD:AAD8039
Eupoecilia ambiguella|MM15672|658[0n]bp|Finland|BOLD:AAD8039
Eupoecilia angustana|MM02556|573[1n]bp|Finland.South Karelia|BOLD:AAC8058
Eupoecilia angustana|MM09005|630[0n]bp|Finland|BOLD:AAC8058
Eupoecilia angustana|TLMF Lep 09174|650[0n]bp|Austria.Vorarlberg|BOLD:AAC8058
Eupoecilia angustana|MM11846|658[0n]bp|Finland|BOLD:AAC8058
Phtheochroa inopiana|MM02117|658[0n]bp|Finland.South Karelia|BOLD:ABZ3448
Phtheochroa inopiana|MM02118|658[0n]bp|Finland.South Karelia|BOLD:ABZ3448
Phtheochroa inopiana|MM15670|658[0n]bp|Finland|BOLD:ABZ3448
Phtheochroa inopiana|TLMF Lep 08084|658[0n]bp|Austria.Vorarlberg|BOLD:ABZ3448
Adoxophyes orana|MM02007|658[0n]bp|Finland.South Karelia|BOLD:AAD8062
Adoxophyes orana|TLMF Lep 08012|658[0n]bp|Austria.Vorarlberg|BOLD:AAD8062
Adoxophyes orana|MM02980|658[0n]bp|Finland.Northern Ostrobothnia|BOLD:AAD8062
Adoxophyes orana|BIOUG04118-B11|601[0n]bp|Finland.Northern Ostrobothnia|BOLD:AAD8062
Adoxophyes orana|MM14376|656[0n]bp|Finland.Aland Islands|BOLD:AAD8062
Adoxophyes orana|BIOUG04118-F07|627[0n]bp|Finland.Northern Ostrobothnia|BOLD:AAD8062
Adoxophyes orana|BIOUG04118-C07|591[0n]bp|Finland.Northern Ostrobothnia|BOLD:AAD8062
Adoxophyes orana|BIOUG04118-E07|604[0n]bp|Finland.Northern Ostrobothnia|BOLD:AAD8062
Adoxophyes orana|BIOUG04118-B10|630[0n]bp|Finland.Northern Ostrobothnia|BOLD:AAD8062
Adoxophyes orana|BIOUG04118-F06|633[0n]bp|Finland.Northern Ostrobothnia|BOLD:AAD8062
Adoxophyes orana|BIOUG04118-E08|633[0n]bp|Finland.Northern Ostrobothnia|BOLD:AAD8062
Ancylis diminutana|TLMF Lep 09950|658[0n]bp|Austria.Vorarlberg|BOLD:AAB6876
Ancylis diminutana|MM10031|658[0n]bp|Finland|BOLD:AAB6876
Ancylis diminutana|MM14123|658[0n]bp|Finland|BOLD:AAB6876
Ancylis diminutana|TLMF Lep 08457|658[0n]bp|Austria.Vorarlberg|BOLD:AAB6876
Ancylis diminutana|MM06536|658[0n]bp|Finland|BOLD:AAB6876
Ancylis diminutana|MM02105|658[0n]bp|Finland.South Karelia|BOLD:AAB6876
Ancylis diminutana|MM02106|639[0n]bp|Finland.South Karelia|BOLD:AAB6876
Ancylis laetana|MM13337|575[0n]bp|Finland|BOLD:AAE1220
Ancylis laetana|MM05480|634[0n]bp|Finland.Northern Ostrobothnia|BOLD:AAE1220
Ancylis laetana|TLMF Lep 09939|658[0n]bp|Austria.Vorarlberg|BOLD:AAE1220
Ancylis laetana|MM20721|658[0n]bp|Finland.North Karelia|BOLD:AAE1220
Ancylis laetana|MM00532|658[0n]bp|Finland.Northern Ostrobothnia|BOLD:AAE1220
Ancylis mitterbacheriana|TLMF Lep 09943|658[0n]bp|Austria.Vorarlberg|BOLD:AAB7371
Ancylis mitterbacheriana|TLMF Lep 07934|658[0n]bp|Austria.Vorarlberg|BOLD:AAB7371
Ancylis mitterbacheriana|TLMF Lep 07469|658[0n]bp|Austria.Vorarlberg|BOLD:AAB7371
Ancylis mitterbacheriana|MM14293|643[0n]bp|Finland.Aland Islands|BOLD:AAB7371
Ancylis mitterbacheriana|MM11193|658[0n]bp|Finland.Aland Islands|BOLD:AAB7371
Ancylis mitterbacheriana|MM03844|658[0n]bp|Finland.Aland Islands|BOLD:AAB7371
Pandemis cerasana|TLMF Lep 08018|658[0n]bp|Austria.Vorarlberg|BOLD:AAA3660
Pandemis cerasana|MM02012|658[0n]bp|Finland.South Karelia|BOLD:AAA3660
Pandemis cerasana|MM13194|658[0n]bp|Finland|BOLD:AAA3660
Pandemis cerasana|MM03256|658[0n]bp|Finland.Northern Ostrobothnia|BOLD:AAA3660
Pandemis cinnamomeana|MM03365|658[0n]bp|Finland.Northern Ostrobothnia|BOLD:AAD0575
Pandemis cinnamomeana|TLMF Lep 08017|658[0n]bp|Austria.Vorarlberg|BOLD:AAD0575
Pandemis cinnamomeana|MM13198|658[0n]bp|Finland|BOLD:AAD0575
Pandemis cinnamomeana|MM01076|658[0n]bp|Finland.South Karelia|BOLD:AAD0575
Pandemis cinnamomeana|MM01075|670[0n]bp|Finland.South Karelia|BOLD:AAD0575
Pandemis dumetana|TLMF Lep 08419|658[0n]bp|Austria.Vorarlberg|BOLD:AAD2733

Pandemis cinnamomeana|MM01076|658[0n]bp|Finland.South Karelia|BOLD:AAD0575
Pandemis cinnamomeana|MM01075|670[0n]bp|Finland.South Karelia|BOLD:AAD0575
Pandemis dumetana|TLMF Lep 08419|658[0n]bp|Austria.Vorarlberg|BOLD:AAD2733
Pandemis dumetana|MM03507|658[0n]bp|Finland.Uusimaa|BOLD:AAD2733
Pandemis dumetana|MM04989|658[0n]bp|Finland.Finland Proper|BOLD:AAD2733
Pandemis dumetana|MM03574|639[0n]bp|Finland.Kymenlaakso|BOLD:AAD2733
Argyrotaenia ljungiana|MM06423|658[0n]bp|Finland|BOLD:AAA2955
Argyrotaenia ljungiana|MM06287|658[0n]bp|Finland|BOLD:AAA2955
Argyrotaenia ljungiana|MM05462|658[0n]bp|Finland.Northern Ostrobothnia|BOLD:AAA2955
Argyrotaenia ljungiana|TLMF Lep 04304|658[0n]bp|Austria.Vorarlberg|BOLD:AAA2955
Argyrotaenia ljungiana|MM21107|638[0n]bp|Finland|BOLD:AAA2955
Spatalistis bifasciana|MM15664|621[0n]bp|Finland|BOLD:AAF4512
Spatalistis bifasciana|MM11009|658[0n]bp|Finland|BOLD:AAF4512
Spatalistis bifasciana|TLMF Lep 09957|658[0n]bp|Austria.Vorarlberg|BOLD:AAF4512
Archips rosana|MM18256|645[0n]bp|Finland|BOLD:ACE6948
Archips rosana|MM10167|634[0n]bp|Finland|BOLD:ACE6948
Archips rosana|MM02016|658[0n]bp|Finland.South Karelia|BOLD:ACE6948
Archips rosana|TLMF Lep 08262|658[0n]bp|Austria.Vorarlberg|BOLD:AAB9404
Archips rosana|TLMF Lep 08029|658[0n]bp|Austria.Vorarlberg|BOLD:AAB9404
Archips xylosteana|MM09757|658[0n]bp|Finland.Aland Islands|BOLD:AAC0366
Archips xylosteana|MM04987|658[0n]bp|Finland.Finland Proper|BOLD:AAC0366
Archips xylosteana|MM09490|658[0n]bp|Finland.Aland Islands|BOLD:AAC0366
Archips xylosteana|TLMF Lep 08013|658[0n]bp|Austria.Vorarlberg|BOLD:AAC0366
Archips oporana|MM08821|658[0n]bp|Finland|BOLD:AAD6710
Archips oporana|MM03449|658[0n]bp|Finland.Uusimaa|BOLD:AAD6710
Archips oporana|TLMF Lep 08015|658[0n]bp|Austria.Vorarlberg|BOLD:AAD6710
Archips oporana|MM02019|658[0n]bp|Finland.South Karelia|BOLD:AAD6710
Archips podana|TLMF Lep 08569|658[0n]bp|Austria.Vorarlberg|BOLD:AAB5839
Archips podana|MM15653|658[0n]bp|Finland|BOLD:AAB5839
Archips podana|MM06722|658[0n]bp|Finland.Aland Islands|BOLD:AAB5839
Archips podana|MM15190|658[0n]bp|Finland|BOLD:AAB5839
Archips podana|MM05228|658[0n]bp|Finland.Finland Proper|BOLD:AAB5839
Archips podana|MM15652|658[0n]bp|Finland.Aland Islands|BOLD:AAB5839
Aphelia paleana|MM03601|614[0n]bp|Finland.Uusimaa|BOLD:AAB6818
Aphelia paleana|MM03496|633[0n]bp|Finland.Uusimaa|BOLD:AAB6818
Aphelia paleana|MM18261|658[0n]bp|Finland|BOLD:AAB6818
Aphelia paleana|MM04997|658[0n]bp|Finland.Finland Proper|BOLD:AAB6818
Aphelia paleana|MM11775|658[0n]bp|Finland|BOLD:AAB6818
Aphelia paleana|TLMF Lep 08430|658[0n]bp|Austria.Vorarlberg|BOLD:ACE9675
Aphelia paleana|MM18260|658[0n]bp|Finland|BOLD:ACE9675
Aphelia paleana|TLMF Lep 07524|658[0n]bp|Austria.Vorarlberg|BOLD:ACE9675
Aphelia paleana|MM01902|648[0n]bp|Finland.South Karelia|BOLD:ACE9675
Aphelia paleana|MM01901|613[0n]bp|Finland.South Karelia|BOLD:ACE9675
Aphelia paleana|MM18262|658[0n]bp|Finland|BOLD:ACE9675
Aphelia paleana|MM11776|658[0n]bp|Finland|BOLD:ACE9675
Aphelia paleana|MM08875|658[0n]bp|Finland|BOLD:ACE9675
Aphelia paleana|MM13167|658[0n]bp|Finland|BOLD:ACE9674
Aphelia paleana|MM13168|658[0n]bp|Finland|BOLD:ACE9674
Aphelia paleana|MM00345|658[0n]bp|Finland|BOLD:ACE9674
Aphelia paleana|MM00118|670[0n]bp|Finland.Uusimaa|BOLD:ACE9674
Aphelia unitana|TLMF Lep 04681|658[0n]bp|Austria.Vorarlberg|BOLD:AAB6819
Aphelia unitana|TLMF Lep 00915|658[0n]bp|Austria.Vorarlberg|BOLD:AAB6819
Aphelia unitana|MM15657|658[0n]bp|Finland|BOLD:AAD2152
Aphelia unitana|MM15656|658[0n]bp|Finland|BOLD:AAD2152
Aphelia unitana|MM18259|658[0n]bp|Finland|BOLD:AAD2152
Aphelia unitana|MM18258|658[0n]bp|Finland|BOLD:AAD2152
Aphelia unitana|MM00038|636[0n]bp|Finland.Northern Ostrobothnia|BOLD:AAD2152
Aphelia unitana|MM06129|658[0n]bp|Finland|BOLD:AAD2152
Aphelia unitana|MM15655|658[0n]bp|Finland|BOLD:AAD2152
Aphelia viburniana|MM06779|657[0n]bp|Finland.Aland Islands|BOLD:ACE6890
Aphelia viburniana|MM14332|658[0n]bp|Finland.Aland Islands|BOLD:ACE6890
Aphelia viburniana|MM06502|658[0n]bp|Finland|BOLD:ACE6890
Aphelia viburniana|MM03785|658[0n]bp|Finland.Northern Ostrobothnia|BOLD:ACE6890
Aphelia viburniana|MM15990|658[0n]bp|Finland|BOLD:ACE6891
Aphelia viburniana|MM17348|658[0n]bp|Finland|BOLD:ACE6891
Aphelia viburniana|TLMF Lep 08023|658[0n]bp|Austria.Vorarlberg|BOLD:ACE6891
Dichelia histrionana|MM06817|656[0n]bp|Finland.Aland Islands|BOLD:ACF5563
Dichelia histrionana|MM06755|657[0n]bp|Finland.Aland Islands|BOLD:ACF5563
Dichelia histrionana|TLMF Lep 08083|658[0n]bp|Austria.Vorarlberg|BOLD:ACF5563
Dichelia histrionana|MM10415|658[0n]bp|Finland.Aland Islands|BOLD:ACF5563
Syndemis musculana|CNCLEP00020418|658[0n]bp|Finland.Paijanne Tavastia|BOLD:ABY7128
Syndemis musculana|TLMF Lep 07924|658[0n]bp|Austria.Vorarlberg|BOLD:ABY7128
Syndemis musculana|MM00524|658[0n]bp|Finland.Northern Ostrobothnia|BOLD:ABY7128
Syndemis musculana|MM02045|614[0n]bp|Finland.South Karelia|BOLD:ABY7128
Syndemis musculana|MM05292|638[0n]bp|Finland.Finland Proper|BOLD:ABY7128
Acleris laterana|MM00762|658[0n]bp|Finland.Northern Ostrobothnia|BOLD:AAM1977
Acleris laterana|MM13787|658[0n]bp|Finland|BOLD:AAM1977
Acleris laterana|TLMF Lep 08454|658[0n]bp|Austria.Vorarlberg|BOLD:AAM1977
Acleris laterana|MM03483|658[0n]bp|Finland.Kymenlaakso|BOLD:AAM1977
Acleris laterana|MM02029|658[0n]bp|Finland.South Karelia|BOLD:AAM1977
Acleris laterana|MM04832|658[0n]bp|Finland.Finland Proper|BOLD:AAM1977
Acleris schalleriana|MM07000|658[0n]bp|Finland.Aland Islands|BOLD:ABZ5454
Acleris schalleriana|MM06999|658[0n]bp|Finland.Aland Islands|BOLD:ABZ5454
Acleris schalleriana|MM06998|658[0n]bp|Finland.Aland Islands|BOLD:ABZ5454
Acleris schalleriana|TLMF Lep 08092|658[0n]bp|Austria.Vorarlberg|BOLD:ABZ5454
Acleris schalleriana|TLMF Lep 07931|658[0n]bp|Austria.Vorarlberg|BOLD:ABZ5454
Acleris hastiana|TLMF Lep 10031|658[0n]bp|Austria.Vorarlberg|BOLD:AAA9796
Acleris hastiana|MM00428|658[0n]bp|Finland.Northern Ostrobothnia|BOLD:AAA9796
Acleris hastiana|MM11959|658[0n]bp|Finland|BOLD:AAA9796
Acleris hastiana|MM08234|658[0n]bp|Finland|BOLD:AAA9796
Acleris hastiana|MM08206|658[0n]bp|Finland|BOLD:AAA9796
Acleris hastiana|MM00402|633[0n]bp|Finland.Northern Ostrobothnia|BOLD:AAA9796
Acleris hastiana|MM20722|658[0n]bp|Finland.North Karelia|BOLD:AAA9796
Acleris hastiana|MM00460|646[0n]bp|Finland.South Karelia|BOLD:AAA9796
Acleris hastiana|MM10283|658[0n]bp|Finland|BOLD:AAA9796
Acleris hastiana|MM19856|658[0n]bp|Finland|BOLD:AAA9796
Acleris shepherdana|MM05363|641[0n]bp|Finland.Finland Proper|BOLD:AAM2761
Acleris shepherdana|TLMF Lep 08418|658[0n]bp|Austria.Vorarlberg|BOLD:AAM2761
Acleris shepherdana|MM08976|658[0n]bp|Finland|BOLD:AAM2761
Acleris bergmanniana|MM02997|621[0n]bp|Finland.Northern Ostrobothnia|BOLD:AAE3614
Acleris bergmanniana|MM08940|658[0n]bp|Finland|BOLD:AAE3614
Acleris bergmanniana|MM02005|658[0n]bp|Finland.South Karelia|BOLD:AAE3614
Acleris bergmanniana|TLMF Lep 08398|658[0n]bp|Austria.Vorarlberg|BOLD:AAE3614

Acleris bergmanniana|MM02597|621[0n]bp|Finland.Northern Ostrobothnia|BOLD:AAE3614
Acleris bergmanniana|MM08940|658[0n]bp|Finland|BOLD:AAE3614
Acleris bergmanniana|MM02005|658[0n]bp|Finland.South Karelia|BOLD:AAE3614
Acleris bergmanniana|TLMF Lep 08398|658[0n]bp|Austria.Vorarlberg|BOLD:AAE3614
Acleris forsskaleana|TLMF Lep 08232|658[0n]bp|Austria.Vorarlberg|BOLD:AAA8796
Acleris forsskaleana|MM13267|658[0n]bp|Finland|BOLD:AAA8796
Acleris forsskaleana|MM02021|658[0n]bp|Finland.South Karelia|BOLD:AAA8796
Acleris forsskaleana|MM05001|639[0n]bp|Finland.Finland Proper|BOLD:AAA8796
Acleris ferrugana|MM04204|658[0n]bp|Finland.Aland Islands|BOLD:AAC3487
Acleris ferrugana|MM05306|641[0n]bp|Finland.Finland Proper|BOLD:AAC3487
Acleris ferrugana|TLMF Lep 07932|658[0n]bp|Austria.Vorarlberg|BOLD:AAC3487
Acleris ferrugana|MM05305|641[0n]bp|Finland.Finland Proper|BOLD:AAC3487
Acleris maccana|MM08298|658[0n]bp|Finland|BOLD:AAA8391
Acleris maccana|TLMF Lep 02971|658[0n]bp|Austria.Vorarlberg|BOLD:AAA8391
Acleris maccana|TLMF Lep 02841|658[0n]bp|Austria.Vorarlberg|BOLD:AAA8391
Acleris maccana|TLMF Lep 02842|658[0n]bp|Austria.Vorarlberg|BOLD:AAA8391
Acleris maccana|MM13882|658[0n]bp|Finland|BOLD:AAA8391
Acleris maccana|MM08197|658[0n]bp|Finland|BOLD:AAA8391
Acleris maccana|MM08762|658[0n]bp|Finland|BOLD:AAA8391
Acleris maccana|MM17981|658[0n]bp|Finland|BOLD:AAA8391
Acleris umbrana|MM08930|658[0n]bp|Finland|BOLD:AAE7430
Acleris umbrana|TLMF Lep 12532|584[0n]bp|Austria.Vorarlberg|BOLD:AAE7430
Acleris umbrana|MM02032|604[0n]bp|Finland.South Karelia|BOLD:AAE7430
Acleris umbrana|MM00624|658[0n]bp|Finland.Northern Ostrobothnia|BOLD:AAE7430
Acleris variegana|TLMF Lep 12533|658[0n]bp|Austria.Vorarlberg|BOLD:AAB2294
Acleris variegana|MM13178|658[0n]bp|Finland|BOLD:ACE3007
Acleris variegana|MM13177|658[0n]bp|Finland|BOLD:ACE3007
Aleimma loeflingiana|MM05204|639[0n]bp|Finland.Finland Proper|BOLD:AAC3136
Aleimma loeflingiana|TLMF Lep 08028|658[0n]bp|Austria.Vorarlberg|BOLD:AAC3136
Aleimma loeflingiana|MM13268|658[0n]bp|Finland|BOLD:AAC3136
Aleimma loeflingiana|MM05259|641[0n]bp|Finland.Finland Proper|BOLD:AAC3136
Tortrix viridana|MM04996|618[0n]bp|Finland.Finland Proper|BOLD:AAC2506
Tortrix viridana|MM06724|658[0n]bp|Finland.Aland Islands|BOLD:AAC2506
Tortrix viridana|TLMF Lep 08019|658[0n]bp|Austria.Vorarlberg|BOLD:AAC2506
Tortrix viridana|MM00510|670[0n]bp|Finland.Finland Proper|BOLD:AAC2506
Eucosmomorpha albersana|TLMF Lep 07936|658[0n]bp|Austria.Vorarlberg|BOLD:AAF2360
Eucosmomorpha albersana|MM09210|634[0n]bp|Finland|BOLD:AAF2360
Eucosmomorpha albersana|MM17939|658[0n]bp|Finland|BOLD:AAF2360
Eucosmomorpha albersana|MM14257|658[0n]bp|Finland.Aland Islands|BOLD:AAF2360
Lobesia reliquana|MM11878|658[0n]bp|Finland|BOLD:AAC9385
Lobesia reliquana|TLMF Lep 12503|658[0n]bp|Austria.Vorarlberg|BOLD:AAC9385
Lobesia reliquana|MM10029|626[0n]bp|Finland|BOLD:AAC9385
Lobesia reliquana|MM15710|658[0n]bp|Finland|BOLD:AAC9385
Lobesia reliquana|MM14040|658[0n]bp|Finland|BOLD:AAC9385
Lobesia reliquana|MM06146|658[0n]bp|Finland|BOLD:AAC9385
Lobesia reliquana|MM03817|631[0n]bp|Finland.Aland Islands|BOLD:AAC9385
Lobesia reliquana|MM15712|658[0n]bp|Finland|BOLD:AAC9385
Lobesia reliquana|MM15711|658[0n]bp|Finland|BOLD:AAC9385
Bactra lacteana|MM06836|658[0n]bp|Finland.Aland Islands|BOLD:AAD3405
Bactra lacteana|MM11013|658[0n]bp|Finland|BOLD:AAD3405
Bactra lacteana|MM13762|658[0n]bp|Finland|BOLD:AAD3405
Bactra lacteana|MM14258|658[0n]bp|Finland.Aland Islands|BOLD:AAD3405
Bactra lacteana|TLMF Lep 09958|599[0n]bp|Austria.Vorarlberg|BOLD:AAD3405
Bactra lacteana|MM06835|658[0n]bp|Finland.Aland Islands|BOLD:AAD3405
Bactra lancealana|MM02454|658[0n]bp|Finland.South Karelia|BOLD:AAB8686
Bactra lancealana|MM03087|631[0n]bp|Finland.Northern Ostrobothnia|BOLD:AAB8686
Bactra lancealana|MM03043|658[0n]bp|Finland.Northern Ostrobothnia|BOLD:AAB8686
Bactra lancealana|MM14172|658[0n]bp|Finland|BOLD:AAB8686
Bactra lancealana|MM06531|670[0n]bp|Finland.Northern Ostrobothnia|BOLD:AAB8686
Bactra lancealana|MM03907|658[0n]bp|Finland.Northern Ostrobothnia|BOLD:AAB8686
Bactra lancealana|MM12145|658[0n]bp|Finland|BOLD:AAB8686
Bactra lancealana|MM06532|658[0n]bp|Finland|BOLD:AAB8686
Bactra lancealana|MM06530|658[0n]bp|Finland|BOLD:AAB8686
Bactra lancealana|TLMF Lep 08031|658[0n]bp|Austria.Vorarlberg|BOLD:AAB8686
Bactra lancealana|MM21123|648[0n]bp|Finland|BOLD:AAB8686
Bactra lancealana|MM12363|646[0n]bp|Finland|BOLD:AAB8686
Bactra lancealana|MM13763|658[0n]bp|Finland|BOLD:AAB8686
Bactra lancealana|MM02453|658[0n]bp|Finland.South Karelia|BOLD:AAB8686
Bactra lancealana|MM12364|646[0n]bp|Finland|BOLD:AAB8686
Bactra lancealana|MM17778|658[0n]bp|Finland|BOLD:AAB8686
Bactra lancealana|MM09817|658[0n]bp|Finland.Aland Islands|BOLD:AAB8686
Cymolomia hartigiana|MM10356|658[0n]bp|Finland|BOLD:ACF2297
Cymolomia hartigiana|MM13316|658[0n]bp|Finland|BOLD:ACF2297
Cymolomia hartigiana|MM03506|658[0n]bp|Finland.Uusimaa|BOLD:ACF2297
Cymolomia hartigiana|TLMF Lep 08213|658[0n]bp|Austria.Vorarlberg|BOLD:AAE3063
Hedya ochroleucana|TLMF Lep 08022|658[0n]bp|Austria.Vorarlberg|BOLD:AAC0586
Hedya ochroleucana|MM05238|645[0n]bp|Finland.Finland Proper|BOLD:AAC0586
Hedya ochroleucana|MM05237|613[0n]bp|Finland.Finland Proper|BOLD:AAC0586
Hedya ochroleucana|MM05003|632[0n]bp|Finland.Finland Proper|BOLD:AAC0586
Endothenia marginana|TLMF Lep 08048|658[0n]bp|Austria.Vorarlberg|BOLD:AAC9535
Endothenia marginana|MM02152|658[0n]bp|Finland.South Karelia|BOLD:AAC9535
Endothenia marginana|MM17227|658[0n]bp|Finland|BOLD:AAC9535
Endothenia marginana|MM15705|658[0n]bp|Finland|BOLD:AAC9535
Endothenia marginana|MM02151|658[0n]bp|Finland.South Karelia|BOLD:AAC9535
Endothenia marginana|MM15702|658[0n]bp|Finland|BOLD:AAC9535
Endothenia marginana|MM15703|658[0n]bp|Finland|BOLD:AAC9535
Endothenia marginana|MM15704|658[0n]bp|Finland|BOLD:AAC9535
Endothenia marginana|MM15706|658[0n]bp|Finland|BOLD:AAC9535
Endothenia marginana|MM15707|658[0n]bp|Finland|BOLD:AAC9535
Endothenia marginana|MM15708|658[0n]bp|Finland|BOLD:AAC9535
Endothenia marginana|MM11827|658[0n]bp|Finland|BOLD:AAC9535
Endothenia marginana|TLMF Lep 07516|658[2n]bp|Austria.Vorarlberg|BOLD:AAC9535
Endothenia nigricostana|TLMF Lep 09779|658[0n]bp|Austria.Vorarlberg|BOLD:AAF0269
Endothenia nigricostana|MM08698|658[0n]bp|Finland|BOLD:AAF0269
Endothenia nigricostana|MM05924|658[0n]bp|Finland|BOLD:AAF0269
Endothenia nigricostana|MM05923|658[0n]bp|Finland|BOLD:AAF0269
Endothenia ericetana|MM15709|658[0n]bp|Finland|BOLD:AAD7173
Endothenia ericetana|MM17232|632[0n]bp|Finland|BOLD:AAD7173
Endothenia ericetana|TLMF Lep 07521|658[0n]bp|Austria.Vorarlberg|BOLD:AAD7173
Endothenia ericetana|MM13238|658[0n]bp|Finland|BOLD:AAD7173
Endothenia ericetana|MM23221|658[0n]bp|Finland|BOLD:AAD7173
Endothenia quadrimaculana|MM02113|658[0n]bp|Finland.South Karelia|BOLD:AAD4973
Endothenia quadrimaculana|MM02110|658[0n]bp|Finland.South Karelia|BOLD:AAD4973

Endothenia ericetana|MM23221|658[0n]bp|Finland|BOLD:AAD7173
Endothenia quadrimaculana|MM02113|658[0n]bp|Finland.South Karelia|BOLD:AAD4973
Endothenia quadrimaculana|MM02110|658[0n]bp|Finland.South Karelia|BOLD:AAD4973
Endothenia quadrimaculana|MM13184|658[0n]bp|Finland|BOLD:AAD4973
Endothenia quadrimaculana|TLMF Lep 08447|658[0n]bp|Austria.Vorarlberg|BOLD:AAD4973
Endothenia quadrimaculana|MM04986|658[0n]bp|Finland.Finland Proper|BOLD:AAD4973
Argyroploce noricana|TLMF Lep 00756|658[0n]bp|Austria.Vorarlberg|BOLD:AAD8744
Argyroploce noricana|MM18285|658[0n]bp|Finland|BOLD:AAD8744
Argyroploce noricana|MM07040|658[0n]bp|Finland.Lapland|BOLD:AAD8744
Argyroploce noricana|MM00083|658[0n]bp|Finland.Lapland|BOLD:AAD8744
Metendothenia atropunctana|MM06353|658[0n]bp|Finland|BOLD:ABZ7645
Metendothenia atropunctana|MM01960|658[0n]bp|Finland.South Karelia|BOLD:ABZ7645
Metendothenia atropunctana|MM13213|658[0n]bp|Finland|BOLD:ABZ7645
Metendothenia atropunctana|TLMF Lep 09202|658[0n]bp|Austria.Vorarlberg|BOLD:ABZ7645
Apotomis infida|MM18303|658[0n]bp|Finland|BOLD:ACF3687
Apotomis infida|MM02968|658[0n]bp|Finland.Northern Ostrobothnia|BOLD:ACF3687
Apotomis infida|MM01981|658[0n]bp|Finland.South Karelia|BOLD:ACF3687
Apotomis infida|TLMF Lep 09393|614[0n]bp|Austria.Vorarlberg|BOLD:ACF3687
Apotomis infida|MM01982|658[0n]bp|Finland.South Karelia|BOLD:ACF3687
Apotomis infida|MM01983|658[0n]bp|Finland.South Karelia|BOLD:ACF3687
Apotomis capreana|TLMF Lep 08079|658[0n]bp|Austria.Vorarlberg|BOLD:ABZ6958
Apotomis capreana|TLMF Lep 10030|658[0n]bp|Austria.Vorarlberg|BOLD:ABZ6958
Apotomis capreana|MM03495|632[0n]bp|Finland.Uusimaa|BOLD:ABZ6958
Apotomis capreana|MM04999|658[0n]bp|Finland.Finland Proper|BOLD:ABZ6958
Apotomis capreana|MM06708|658[0n]bp|Finland.Aland Islands|BOLD:ABZ6958
Apotomis capreana|MM08882|658[0n]bp|Finland|BOLD:ABZ6958
Apotomis capreana|MM01991|658[0n]bp|Finland.South Karelia|BOLD:ABZ6958
Apotomis capreana|MM06593|658[0n]bp|Finland|BOLD:ABZ6958
Apotomis sauciana|MM15697|658[0n]bp|Finland|BOLD:ABZ6958
Apotomis sauciana|MM15696|658[0n]bp|Finland|BOLD:ABZ6958
Apotomis sauciana|TLMF Lep 03623|658[0n]bp|Austria.Vorarlberg|BOLD:ABZ6958
Apotomis sauciana|MM08347|658[0n]bp|Finland|BOLD:ABZ6958
Apotomis sauciana|MM21065|658[0n]bp|Finland|BOLD:ABZ6958
Apotomis sauciana|MM15698|658[0n]bp|Finland|BOLD:ABZ6958
Apotomis sauciana|MM04183|658[0n]bp|Finland.Lapland|BOLD:ABZ6958
Apotomis sauciana|MM04184|658[0n]bp|Finland.Lapland|BOLD:ABZ6958
Apotomis sauciana|MM04187|658[0n]bp|Finland|BOLD:ABZ6958
Apotomis sauciana|MM18306|658[0n]bp|Finland|BOLD:ABZ6958
Apotomis sauciana|MM18308|658[0n]bp|Finland|BOLD:ABZ6958
Apotomis sauciana|MM18640|658[0n]bp|Finland|BOLD:ABZ6958
Apotomis sauciana|MM04182|658[0n]bp|Finland.Lapland|BOLD:ABZ6958
Apotomis sauciana|MM06283|658[0n]bp|Finland|BOLD:ABZ6958
Apotomis sauciana|MM06282|658[0n]bp|Finland|BOLD:ABZ6958
Apotomis sauciana|MM21063|658[0n]bp|Finland|BOLD:ABZ6958
Apotomis sauciana|MM21064|658[0n]bp|Finland|BOLD:ABZ6958
Apotomis sauciana|MM04185|658[0n]bp|Finland|BOLD:ABZ6958
Apotomis sauciana|MM06823|658[0n]bp|Finland.Aland Islands|BOLD:ABZ6958
Apotomis sauciana|MM04186|658[0n]bp|Finland|BOLD:ABZ6958
Pristerognatha penthinana|TLMF Lep 09160|658[0n]bp|Austria.Vorarlberg|BOLD:AAL5814
Pristerognatha penthinana|MM06218|658[0n]bp|Finland|BOLD:AAL5814
Pristerognatha penthinana|MM18839|658[1n]bp|Finland|BOLD:AAL5814
Celypha cespitana|MM05284|637[0n]bp|Finland.Finland Proper|BOLD:AAA9471
Celypha cespitana|MM11829|658[0n]bp|Finland|BOLD:AAA9471
Celypha cespitana|MM18830|658[0n]bp|Finland|BOLD:AAA9471
Celypha cespitana|MM13295|658[0n]bp|Finland|BOLD:AAA9471
Celypha cespitana|MM06764|658[0n]bp|Finland.Aland Islands|BOLD:AAA9471
Celypha cespitana|MM03205|630[0n]bp|Finland.Northern Ostrobothnia|BOLD:AAA9471
Celypha cespitana|MM06545|658[0n]bp|Finland|BOLD:AAA9471
Celypha cespitana|MM23196|658[0n]bp|Finland|BOLD:AAA9471
Celypha cespitana|TLMF Lep 10039|658[0n]bp|Austria.Vorarlberg|BOLD:AAA9471
Celypha rurestrana|TLMF Lep 09172|658[0n]bp|Austria.Vorarlberg|BOLD:ABX5517
Celypha rurestrana|MM05267|658[0n]bp|Finland.Finland Proper|BOLD:ABX5517
Celypha rurestrana|MM09992|658[0n]bp|Finland|BOLD:ABX5517
Celypha rurestrana|MM03011|658[0n]bp|Finland.Northern Ostrobothnia|BOLD:ABX5517
Celypha rivulana|MM13292|658[0n]bp|Finland|BOLD:AAF2534
Celypha rivulana|MM03001|658[0n]bp|Finland.Northern Ostrobothnia|BOLD:AAF2534
Celypha rivulana|TLMF Lep 08020|658[0n]bp|Austria.Vorarlberg|BOLD:AAF2534
Celypha rivulana|MM02072|658[0n]bp|Finland.South Karelia|BOLD:AAF2534
Orthotaenia undulana|MM08483|658[0n]bp|Finland|BOLD:AAB4021
Orthotaenia undulana|MM13293|658[0n]bp|Finland|BOLD:AAB4021
Orthotaenia undulana|MM18287|658[0n]bp|Finland|BOLD:AAB4021
Orthotaenia undulana|TLMF Lep 09920|658[0n]bp|Austria.Vorarlberg|BOLD:AAB4021
Orthotaenia undulana|MM11910|658[0n]bp|Finland|BOLD:AAB4021
Orthotaenia undulana|MM03014|658[0n]bp|Finland.Northern Ostrobothnia|BOLD:AAB4021
Orthotaenia undulana|BIOUG04116-A07|614[0n]bp|Finland.Northern Ostrobothnia|BOLD:AAB4021
Orthotaenia undulana|MM02068|567[0n]bp|Finland.South Karelia|BOLD:AAB4021
Orthotaenia undulana|BIOUG04490-F01|614[0n]bp|Finland.Northern Ostrobothnia|BOLD:AAB4021
Phiaris bipunctana|MM18286|658[0n]bp|Finland|BOLD:AAA6005
Phiaris bipunctana|TLMF Lep 09936|658[0n]bp|Austria.Vorarlberg|BOLD:AAA6005
Phiaris bipunctana|MM03005|658[0n]bp|Finland.Northern Ostrobothnia|BOLD:AAA6005
Phiaris bipunctana|MM08494|658[0n]bp|Finland|BOLD:AAA6005
Phiaris bipunctana|MM08291|658[0n]bp|Finland|BOLD:AAA6005
Phiaris schulziana|TLMF Lep 09147|658[0n]bp|Austria.Vorarlberg|BOLD:ACF5701
Phiaris schulziana|MM03787|658[0n]bp|Finland.Northern Ostrobothnia|BOLD:ACF5701
Phiaris schulziana|MM18291|658[0n]bp|Finland|BOLD:ACF5701
Phiaris schulziana|MM08475|658[0n]bp|Finland|BOLD:ACF5701
Phiaris schulziana|MM06286|658[0n]bp|Finland|BOLD:ACF5701
Phiaris schulziana|MM06311|658[0n]bp|Finland|BOLD:ACF5701
Phiaris schulziana|MM04120|658[0n]bp|Finland.Lapland|BOLD:ACF5701
Phiaris schulziana|MM04118|658[0n]bp|Finland.Lapland|BOLD:ACF5701
Phiaris schulziana|MM18289|658[0n]bp|Finland|BOLD:ACF5701
Phiaris schulziana|MM18290|658[0n]bp|Finland|BOLD:ACF5701
Phiaris schulziana|MM08474|658[0n]bp|Finland|BOLD:ACF5701
Phiaris schulziana|MM14581|658[0n]bp|Finland|BOLD:ACF5701
Phiaris schulziana|MM14582|658[0n]bp|Finland|BOLD:ACF5701
Phiaris schulziana|MM04119|658[0n]bp|Finland.Lapland|BOLD:ACF5701
Phiaris schulziana|MM06351|658[0n]bp|Finland|BOLD:ACF5701
Phiaris schulziana|MM06203|658[0n]bp|Finland|BOLD:ACF5701
Phiaris schulziana|MM21043|658[0n]bp|Finland|BOLD:ACF5701
Celypha lacunana|MM23074|658[0n]bp|Finland|BOLD:ACE8186
Celypha lacunana|MM13309|627[0n]bp|Finland|BOLD:ACE8186
Celypha lacunana|MM23082|658[0n]bp|Finland|BOLD:ACE8186
Celypha lacunana|MM02070|658[0n]bp|Finland.South Karelia|BOLD:AAC3531

Celypha lacunana|MM13309|627[0n]bp|Finland|BOLD:ACE8186
Celypha lacunana|MM23082|658[0n]bp|Finland|BOLD:ACE8186
Celypha lacunana|MM02070|658[0n]bp|Finland.South Karelia|BOLD:AAC3531
Celypha lacunana|TLMF Lep 07988|658[0n]bp|Austria.Vorarlberg|BOLD:AAC3531
Celypha lacunana|TLMF Lep 07930|658[0n]bp|Austria.Vorarlberg|BOLD:AAC3531
Celypha lacunana|MM03003|614[0n]bp|Finland.Northern Ostrobothnia|BOLD:AAC3531
Celypha lacunana|MM23083|658[0n]bp|Finland|BOLD:AAC3531
Celypha lacunana|MM23081|600[0n]bp|Finland|BOLD:AAC3531
Celypha lacunana|MM23079|602[0n]bp|Finland|BOLD:AAC3531
Celypha lacunana|MM23076|658[0n]bp|Finland|BOLD:AAC3531
Celypha lacunana|MM23075|658[0n]bp|Finland|BOLD:AAC3531
Celypha lacunana|MM23077|658[0n]bp|Finland|BOLD:AAC3531
Olethreutinae|BIOUG04116-E03|624[0n]bp|Finland.Northern Ostrobothnia|BOLD:AAC3531
Phiaris dissolutana|MM03007|658[0n]bp|Finland.Northern Ostrobothnia|BOLD:ABZ7390
Phiaris dissolutana|TLMF Lep 10041|658[0n]bp|Austria.Vorarlberg|BOLD:ABZ7390
Phiaris dissolutana|MM18284|658[0n]bp|Finland|BOLD:ABZ7390
Phiaris dissolutana|MM03008|658[0n]bp|Finland.Northern Ostrobothnia|BOLD:ABZ7390
Piniphila bifasciana|TLMF Lep 08069|658[0n]bp|Austria.Vorarlberg|BOLD:AAC5440
Piniphila bifasciana|MM14295|658[0n]bp|Finland.Aland Islands|BOLD:AAC5440
Piniphila bifasciana|MM15701|658[0n]bp|Finland.Aland Islands|BOLD:AAC5440
Piniphila bifasciana|MM15700|658[0n]bp|Finland.Aland Islands|BOLD:AAC5440
Olethreutes arcuella|MM09557|658[0n]bp|Finland.Aland Islands|BOLD:AAC2510
Olethreutes arcuella|MM18282|658[0n]bp|Finland|BOLD:AAC2510
Olethreutes arcuella|MM13294|658[0n]bp|Finland|BOLD:AAC2510
Olethreutes arcuella|TLMF Lep 09158|658[0n]bp|Austria|BOLD:AAC2510
Phiaris micana|TLMF Lep 10029|658[0n]bp|Austria.Vorarlberg|BOLD:ACE6219
Phiaris micana|TLMF Lep 09940|658[0n]bp|Austria.Vorarlberg|BOLD:ACE6219
Phiaris micana|MM03367|658[0n]bp|Finland.Northern Ostrobothnia|BOLD:ACE6219
Phiaris micana|MM18288|658[0n]bp|Finland|BOLD:ACE6219
Phiaris micana|MM03330|658[0n]bp|Finland.Northern Ostrobothnia|BOLD:ACE6219
Phiaris palustrana|MM03286|634[0n]bp|Finland.Northern Ostrobothnia|BOLD:AAC8201
Phiaris palustrana|MM13317|658[0n]bp|Finland|BOLD:AAC8201
Phiaris palustrana|MM02052|658[0n]bp|Finland.South Karelia|BOLD:AAC8201
Phiaris palustrana|TLMF Lep 02835|637[0n]bp|Austria.Vorarlberg|BOLD:AAC8201
Pseudohermenias abietana|MM06143|658[0n]bp|Finland|BOLD:AAD7589
Pseudohermenias abietana|MM02076|571[0n]bp|Finland.South Karelia|BOLD:AAD7589
Pseudohermenias abietana|MM14222|658[0n]bp|Finland.Aland Islands|BOLD:AAD7589
Pseudohermenias abietana|TLMF Lep 09929|603[0n]bp|Austria.Vorarlberg|BOLD:AAD7589
Hedya nubiferana|MM13244|658[0n]bp|Finland|BOLD:AAA4552
Hedya nubiferana|TLMF Lep 09926|658[0n]bp|Austria.Vorarlberg|BOLD:AAA4552
Hedya nubiferana|MM05336|658[0n]bp|Finland.Finland Proper|BOLD:AAA4552
Hedya nubiferana|MM01964|658[0n]bp|Finland.South Karelia|BOLD:AAA4552
Hedya pruniana|MM09753|658[0n]bp|Finland|BOLD:AAD0577
Hedya pruniana|TLMF Lep 09934|658[0n]bp|Austria.Vorarlberg|BOLD:AAD0577
Hedya pruniana|MM18302|658[0n]bp|Finland|BOLD:AAD0577
Hedya pruniana|MM18301|658[0n]bp|Finland|BOLD:AAD0577
Pseudosciaphila branderiana|MM01908|658[0n]bp|Finland.South Karelia|BOLD:AAD9269
Pseudosciaphila branderiana|MM13180|658[0n]bp|Finland|BOLD:AAD9269
Pseudosciaphila branderiana|MM14285|658[0n]bp|Finland.Aland Islands|BOLD:AAD9269
Pseudosciaphila branderiana|TLMF Lep 08000|658[0n]bp|Austria.Vorarlberg|BOLD:AAD9269
Capua vulgana|MM03806|658[0n]bp|Finland.Finland Proper|BOLD:AAD5387
Capua vulgana|BIOUG04116-A01|624[0n]bp|Finland.Northern Ostrobothnia|BOLD:AAD5387
Capua vulgana|MM14182|658[0n]bp|Finland|BOLD:AAD5387
Capua vulgana|MM03925|658[0n]bp|Finland.South Karelia|BOLD:AAD5387
Capua vulgana|TLMF Lep 07933|658[0n]bp|Austria.Vorarlberg|BOLD:AAD5387
Eriopsela quadrana|TLMF Lep 00868|658[0n]bp|Austria.Vorarlberg|BOLD:AAD4787
Eriopsela quadrana|MM04122|658[0n]bp|Finland.Lapland|BOLD:AAD4787
Eriopsela quadrana|MM00507|643[0n]bp|Finland.Lapland|BOLD:AAD4787
Eriopsela quadrana|MM04123|658[0n]bp|Finland.Lapland|BOLD:AAD4787
Gypsonoma dealbana|TLMF Lep 08044|658[0n]bp|Austria.Vorarlberg|BOLD:AAC8347
Gypsonoma dealbana|TLMF Lep 08251|658[0n]bp|Austria.Vorarlberg|BOLD:AAC8347
Gypsonoma dealbana|MM09819|658[0n]bp|Finland.Aland Islands|BOLD:AAB0380
Gypsonoma dealbana|MM02149|571[0n]bp|Finland.South Karelia|BOLD:AAB0380
Gypsonoma dealbana|MM03482|658[0n]bp|Finland.Kymenlaakso|BOLD:AAB0380
Gypsonoma sociana|MM02154|658[0n]bp|Finland.South Karelia|BOLD:AAA6642
Gypsonoma sociana|MM06139|658[0n]bp|Finland|BOLD:AAA6642
Gypsonoma sociana|MM09722|658[0n]bp|Finland.Aland Islands|BOLD:AAA6642
Gypsonoma sociana|MM09717|647[0n]bp|Finland.Aland Islands|BOLD:AAA6642
Gypsonoma sociana|TLMF Lep 09394|658[0n]bp|Austria.Vorarlberg|BOLD:AAA6642
Gypsonoma sociana|MM03199|658[0n]bp|Finland.Northern Ostrobothnia|BOLD:AAA6642
Rhopobota naevana|MM22962|658[0n]bp|Finland|BOLD:AAA9812
Rhopobota naevana|MM22968|658[0n]bp|Finland|BOLD:AAA9812
Rhopobota naevana|TLMF Lep 08227|658[0n]bp|Austria.Vorarlberg|BOLD:AAA9812
Rhopobota naevana|MM22967|658[0n]bp|Finland|BOLD:AAA9812
Rhopobota naevana|TLMF Lep 08098|658[0n]bp|Austria.Vorarlberg|BOLD:AAA9812
Rhopobota naevana|MM02121|631[0n]bp|Finland.South Karelia|BOLD:AAA9812
Rhopobota naevana|MM22970|622[0n]bp|Finland|BOLD:AAA9812
Rhopobota naevana|MM22964|658[0n]bp|Finland|BOLD:AAA9812
Rhopobota naevana|MM00760|658[0n]bp|Finland.Northern Ostrobothnia|BOLD:AAA9812
Rhopobota naevana|MM13304|658[0n]bp|Finland|BOLD:AAA9812
Rhopobota naevana|MM22969|658[0n]bp|Finland|BOLD:AAA9812
Rhopobota naevana|MM22966|658[0n]bp|Finland|BOLD:AAA9812
Rhopobota naevana|MM08217|647[0n]bp|Finland|BOLD:AAA9812
Rhopobota naevana|MM22961|658[0n]bp|Finland|BOLD:AAA9812
Rhopobota naevana|MM22963|658[0n]bp|Finland|BOLD:AAA9812
Rhopobota naevana|MM22965|658[0n]bp|Finland|BOLD:AAA9812
Rhopobota stagnana|MM14095|658[0n]bp|Finland|BOLD:AAF7954
Rhopobota stagnana|MM14094|658[0n]bp|Finland|BOLD:AAF7954
Rhopobota stagnana|MM11017|658[0n]bp|Finland|BOLD:AAF7954
Rhopobota stagnana|TLMF Lep 08479|658[0n]bp|Austria.Vorarlberg|BOLD:AAF7954
Cnephasia alticolana|MM08618|638[0n]bp|Finland|BOLD:ACF2531
Cnephasia alticolana|MM18636|658[0n]bp|Finland|BOLD:ACF2531
Cnephasia alticolana|MM08619|658[0n]bp|Finland|BOLD:ACF2531
Cnephasia alticolana|TLMF Lep 08397|658[0n]bp|Austria.Vorarlberg|BOLD:ACF2531
Cnephasia alticolana|MM15662|658[0n]bp|Finland|BOLD:ACF2531
Cnephasia alticolana|MM15663|658[0n]bp|Finland|BOLD:ACF2531
Cnephasia asseclana|MM06712|614[0n]bp|Finland.Aland Islands|BOLD:AAA6293
Cnephasia asseclana|MM06548|658[0n]bp|Finland|BOLD:AAA6293
Cnephasia asseclana|MM06680|658[0n]bp|Finland|BOLD:AAA6293
Cnephasia asseclana|MM09743|658[0n]bp|Finland.Aland Islands|BOLD:AAA6293
Cnephasia asseclana|MM21120|658[0n]bp|Finland|BOLD:AAA6293
Cnephasia asseclana|MM05257|658[0n]bp|Finland.Finland Proper|BOLD:AAA6293
[illegible]

Cnephasia asseclana|MM09743|658[0n]bp|Finland.Aland Islands|BOLD:AAA6293
Cnephasia asseclana|MM21120|658[0n]bp|Finland|BOLD:AAA6293
Cnephasia asseclana|MM05257|658[0n]bp|Finland.Finland Proper|BOLD:AAA6293
Cnephasia asseclana|MM05256|658[0n]bp|Finland.Finland Proper|BOLD:AAA6293
Cnephasia asseclana|TLMF Lep 09980|658[0n]bp|Austria.Vorarlberg|BOLD:AAA6293
Cnephasia asseclana|MM11849|658[0n]bp|Finland|BOLD:AAA6293
Cnephasia asseclana|MM02140|658[0n]bp|Finland.South Karelia|BOLD:AAA6293
Cnephasia asseclana|MM06681|658[0n]bp|Finland|BOLD:AAA6293
Cnephasia asseclana|MM06679|658[0n]bp|Finland|BOLD:AAA6293
Cnephasia asseclana|MM06678|658[0n]bp|Finland|BOLD:AAA6293
Cnephasia asseclana|MM03504|629[0n]bp|Finland.Uusimaa|BOLD:AAA6293
Cnephasia asseclana|MM06677|634[0n]bp|Finland|BOLD:AAA6293
Cnephasia asseclana|TLMF Lep 08252|658[0n]bp|Austria.Vorarlberg|BOLD:AAA6293
Cnephasia asseclana|MM12315|658[0n]bp|Finland|BOLD:AAA6293
Cnephasia asseclana|TLMF Lep 07985|658[0n]bp|Austria.Vorarlberg|BOLD:AAA6293
Cnephasia asseclana|MM09559|658[0n]bp|Finland.Aland Islands|BOLD:AAA6293
Cnephasia asseclana|MM13863|658[0n]bp|Finland.Lapland|BOLD:AAA6293
Cnephasia asseclana|MM14449|658[0n]bp|Finland|BOLD:AAA6293
Cnephasia asseclana|MM03463|658[0n]bp|Finland.Uusimaa|BOLD:AAA6293
Cnephasia stephensiana|TLMF Lep 08016|658[0n]bp|Austria.Vorarlberg|BOLD:AAA6292
Cnephasia stephensiana|MM05680|658[0n]bp|Finland.South Karelia|BOLD:AAA6292
Cnephasia stephensiana|MM21108|658[0n]bp|Finland|BOLD:AAA6292
Cnephasia stephensiana|MM11848|658[0n]bp|Finland|BOLD:AAA6292
Cnephasia stephensiana|MM17356|658[0n]bp|Finland|BOLD:AAA6292
Cnephasia stephensiana|MM02129|658[0n]bp|Finland.South Karelia|BOLD:AAA6292
Cnephasia stephensiana|MM13182|658[0n]bp|Finland|BOLD:AAA6292
Cnephasia stephensiana|MM13183|658[0n]bp|Finland|BOLD:AAA6292
Cnephasia stephensiana|MM17357|658[0n]bp|Finland|BOLD:AAA6292
Cnephasia stephensiana|MM05255|658[0n]bp|Finland.Finland Proper|BOLD:AAA6292
Cnephasia stephensiana|MM02128|638[0n]bp|Finland.South Karelia|BOLD:AAA6292
Cnephasia stephensiana|MM04998|639[0n]bp|Finland.Finland Proper|BOLD:AAA6292
Cnephasia stephensiana|MM03468|658[0n]bp|Finland.Uusimaa|BOLD:AAA6292
Cnephasia stephensiana|MM05203|658[0n]bp|Finland.Finland Proper|BOLD:AAA6292
Cnephasia stephensiana|MM08981|658[0n]bp|Finland|BOLD:AAA6292
Cnephasia stephensiana|TLMF Lep 08741|658[0n]bp|Austria.Vorarlberg|BOLD:AAA6292
Cnephasia stephensiana|MM03467|658[0n]bp|Finland.Uusimaa|BOLD:AAA6292
Eana argentana|MM06246|658[0n]bp|Finland|BOLD:AAA8425
Eana argentana|MM10012|634[0n]bp|Finland|BOLD:AAA8425
Eana argentana|TLMF Lep 08431|658[0n]bp|Austria.Vorarlberg|BOLD:AAA8425
Eana argentana|MM05253|658[0n]bp|Finland.Finland Proper|BOLD:AAA8425
Eana osseana|TLMF Lep 09152|658[0n]bp|Austria.Vorarlberg|BOLD:AAA6265
Eana osseana|MM10348|658[0n]bp|Finland|BOLD:AAA6265
Eana osseana|MM02993|658[0n]bp|Finland.Northern Ostrobothnia|BOLD:AAA6265
Eana osseana|MM04140|658[0n]bp|Finland.Lapland|BOLD:AAA6265
Doloploca punctulana|TLMF Lep 07917|658[0n]bp|Austria.Vorarlberg|BOLD:AAF5268
Doloploca punctulana|MM19945|608[0n]bp|Finland.Uusimaa|BOLD:AAF5268
Eana incanana|TLMF Lep 07534|658[0n]bp|Austria.Vorarlberg|BOLD:AAD7476
Eana incanana|MM05265|637[0n]bp|Finland.Finland Proper|BOLD:AAD7476
Eana incanana|MM03044|634[0n]bp|Finland.Northern Ostrobothnia|BOLD:AAD7476
Eana incanana|MM13331|542[0n]bp|Finland|BOLD:AAD7476
Eana penziana|MM08293|621[0n]bp|Finland|BOLD:AAB8228
Eana penziana|TLMF Lep 00926|658[0n]bp|Austria.Vorarlberg|BOLD:AAB8228
Eana penziana|TLMF Lep 00925|658[0n]bp|Austria.Vorarlberg|BOLD:AAB8228
Eana penziana|MM08314|658[0n]bp|Finland|BOLD:AAB8228
Eana penziana|MM03500|658[0n]bp|Finland.Uusimaa|BOLD:AAB8228
Eana penziana|MM03455|658[0n]bp|Finland.Uusimaa|BOLD:AAB8228
Eudemis porphyrana|MM05198|658[0n]bp|Finland.Finland Proper|BOLD:AAC6854
Eudemis porphyrana|MM02056|571[0n]bp|Finland.South Karelia|BOLD:AAC6854
Eudemis porphyrana|MM08923|658[0n]bp|Finland|BOLD:AAC6854
Eudemis porphyrana|TLMF Lep 08250|658[0n]bp|Austria.Vorarlberg|BOLD:AAC6854
Eulia ministrana|MM00109|658[0n]bp|Finland.Uusimaa|BOLD:AAA7315
Eulia ministrana|TLMF Lep 09144|658[0n]bp|Austria.Vorarlberg|BOLD:AAA7315
Eulia ministrana|MM06310|670[0n]bp|Finland.Lapland|BOLD:AAA7315
Eulia ministrana|MM00078|658[0n]bp|Finland.Lapland|BOLD:AAA7315
Eulia ministrana|MM00064|658[0n]bp|Finland.Northern Ostrobothnia|BOLD:AAA7315
Eulia ministrana|MM01066|658[0n]bp|Finland.South Karelia|BOLD:AAA7315
Eulia ministrana|MM01067|620[0n]bp|Finland.South Karelia|BOLD:AAA7315
Eulia ministrana|MM13206|609[0n]bp|Finland|BOLD:AAA7315
Eulia ministrana|MM03425|658[0n]bp|Finland.Lapland|BOLD:AAA7315
Pseudargyrotoza conwagana|MM14310|610[0n]bp|Finland.Aland Islands|BOLD:AAC8983
Pseudargyrotoza conwagana|TLMF Lep 07927|658[0n]bp|Austria.Vorarlberg|BOLD:AAC8983
Pseudargyrotoza conwagana|MM11917|658[0n]bp|Finland|BOLD:AAC8983
Pseudargyrotoza conwagana|MM00680|658[0n]bp|Finland.Lapland|BOLD:AAC8983
Ancylis apicella|TLMF Lep 09930|658[0n]bp|Austria.Vorarlberg|BOLD:ACE8189
Ancylis apicella|CNCLEP00020414|653[0n]bp|Finland.Paijanne Tavastia|BOLD:AAD2001
Ancylis apicella|MM03392|658[0n]bp|Finland.Aland Islands|BOLD:AAD2001
Ancylis apicella|MM08975|658[0n]bp|Finland|BOLD:AAD2001
Ancylis apicella|MM02103|615[0n]bp|Finland.South Karelia|BOLD:AAD2001
Ancylis badiana|CNCLEP00020415|658[0n]bp|Finland.Kainuu|BOLD:AAC1552
Ancylis badiana|MM02089|658[0n]bp|Finland.South Karelia|BOLD:ACJ4507
Ancylis badiana|MM02087|658[0n]bp|Finland.South Karelia|BOLD:ACJ4507
Ancylis badiana|MM02088|658[0n]bp|Finland.South Karelia|BOLD:ACJ4507
Ancylis badiana|MM08954|658[0n]bp|Finland|BOLD:ACJ4507
Ancylis badiana|TLMF Lep 07470|658[0n]bp|Austria.Vorarlberg|BOLD:ACJ4507
Ancylis badiana|MM02144|658[0n]bp|Finland.South Karelia|BOLD:ACJ4507
Ancylis badiana|MM18320|658[0n]bp|Finland|BOLD:ACJ4507
Ancylis badiana|MM08955|658[0n]bp|Finland|BOLD:ACJ4507
Ancylis badiana|MM22971|658[0n]bp|Finland|BOLD:ABY4377
Ancylis badiana|MM22975|630[0n]bp|Finland|BOLD:ABY4377
Ancylis badiana|MM22980|606[0n]bp|Finland|BOLD:ABY4377
Ancylis badiana|MM22978|606[0n]bp|Finland|BOLD:ABY4377
Ancylis badiana|MM22973|632[0n]bp|Finland|BOLD:ABY4377
Ancylis badiana|MM22977|658[0n]bp|Finland|BOLD:ABY4377
Ancylis badiana|MM22976|658[0n]bp|Finland|BOLD:ABY4377
Ancylis badiana|MM00600|626[0n]bp|Finland.Northern Ostrobothnia|BOLD:ABY4377
Ancylis badiana|MM04334|658[0n]bp|Finland.Finland Proper|BOLD:ABY4377
Ancylis badiana|MM13285|658[0n]bp|Finland|BOLD:ABY4377
Ancylis badiana|MM18319|658[0n]bp|Finland|BOLD:ABY4377
Ancylis badiana|TLMF Lep 08082|658[0n]bp|Austria.Vorarlberg|BOLD:ABY4377
Ancylis badiana|MM11871|658[0n]bp|Finland|BOLD:ABY4377
Ancylis badiana|MM22972|658[0n]bp|Finland|BOLD:ABY4377
Ancylis badiana|MM22979|658[0n]bp|Finland|BOLD:ABY4377
Ancylis myrtillana|MM00638|638[0n]bp|Finland.Northern Ostrobothnia|BOLD:ABZ7857

Ancylis badiana|MM22972|658[0n]bp|Finland|BOLD:ABY4377
Ancylis badiana|MM22979|658[0n]bp|Finland|BOLD:ABY4377
Ancylis myrtillana|MM00638|638[0n]bp|Finland.Northern Ostrobothnia|BOLD:ABZ7857
Ancylis myrtillana|TLMF Lep 09959|658[0n]bp|Austria.Vorarlberg|BOLD:ABZ7857
Ancylis myrtillana|MM04335|658[0n]bp|Finland.Finland Proper|BOLD:ABZ7857
Ancylis myrtillana|MM02085|658[0n]bp|Finland.South Karelia|BOLD:ABZ7857
Ancylis unculana|TLMF Lep 08456|658[0n]bp|Austria.Vorarlberg|BOLD:AAD3929
Ancylis unculana|MM02610|658[0n]bp|Finland.South Karelia|BOLD:AAD3930
Ancylis unculana|MM02143|621[0n]bp|Finland.South Karelia|BOLD:AAD3930
Ancylis unculana|MM06460|658[0n]bp|Finland|BOLD:AAD3930
Clepsis rurinana|MM13272|575[0n]bp|Finland|BOLD:AAF4503
Clepsis rurinana|TLMF Lep 08086|658[0n]bp|Austria.Vorarlberg|BOLD:AAF4503
Clepsis rurinana|MM06610|658[0n]bp|Finland|BOLD:AAF4503
Clepsis rurinana|MM02009|658[0n]bp|Finland.South Karelia|BOLD:AAF4503
Cydia pomonella|TLMF Lep 07926|658[0n]bp|Austria.Vorarlberg|BOLD:AAA3532
Cydia pomonella|MM03480|658[0n]bp|Finland.Kymenlaakso|BOLD:AAA3532
Cydia pomonella|MM01761|649[0n]bp|Finland.South Karelia|BOLD:AAA3532
Cydia pomonella|MM09453|658[0n]bp|Finland.Aland Islands|BOLD:AAA3532
Cydia duplicana|MM08407|658[0n]bp|Finland|BOLD:AAF7700
Cydia duplicana|MM03040|658[0n]bp|Finland.Northern Ostrobothnia|BOLD:AAF7700
Cydia duplicana|MM12137|658[0n]bp|Finland|BOLD:AAF7700
Cydia duplicana|TLMF Lep 08295|637[0n]bp|Austria.Vorarlberg|BOLD:ABZ7551
Cydia splendana|MM08633|658[0n]bp|Finland.Aland Islands|BOLD:ACJ4322
Cydia splendana|MM03565|658[0n]bp|Finland.Kymenlaakso|BOLD:ACJ4322
Cydia splendana|TLMF Lep 08032|658[0n]bp|Austria.Vorarlberg|BOLD:AAC0640
Cydia splendana|MM03458|658[0n]bp|Finland.Uusimaa|BOLD:AAC0640
Cydia splendana|MM13175|658[0n]bp|Finland|BOLD:ACJ4322
Cydia inquinatana|MM05013|658[1n]bp|Finland.Finland Proper|BOLD:AAE3029
Cydia inquinatana|TLMF Lep 08094|658[0n]bp|Austria.Vorarlberg|BOLD:AAE3029
Cydia inquinatana|MM03875|658[0n]bp|Finland.Aland Islands|BOLD:AAE3029
Cydia inquinatana|MM03843|658[0n]bp|Finland.Aland Islands|BOLD:AAE3029
Cydia illutana|MM00966|629[0n]bp|Finland.Northern Ostrobothnia|BOLD:AAD5427
Cydia illutana|MM00965|627[0n]bp|Finland.Northern Ostrobothnia|BOLD:AAD5427
Cydia illutana|TLMF Lep 09947|658[0n]bp|Austria.Vorarlberg|BOLD:AAD5427
Cydia illutana|TLMF Lep 08248|658[0n]bp|Austria.Vorarlberg|BOLD:AAD5427
Cydia illutana|TLMF Lep 09389|658[0n]bp|Austria.Vorarlberg|BOLD:AAD5427
Cydia illutana|MM15737|658[0n]bp|Finland|BOLD:AAD5427
Cydia illutana|MM00964|658[0n]bp|Finland.Northern Ostrobothnia|BOLD:AAD5427
Cydia illutana|MM18808|658[0n]bp|Finland|BOLD:AAD5427
Cydia strobilella|MM06291|658[0n]bp|Finland|BOLD:AAD5907
Cydia strobilella|MM06411|658[0n]bp|Finland|BOLD:AAD5907
Cydia strobilella|TLMF Lep 09194|658[1n]bp|Austria.Vorarlberg|BOLD:AAD5907
Cydia strobilella|MM06412|658[0n]bp|Finland|BOLD:AAD5907
Grapholita lobarzewskii|TLMF Lep 08042|658[0n]bp|Austria.Vorarlberg|BOLD:AAP8428
Grapholita lobarzewskii|MM18893|658[0n]bp|Finland.Aland Islands|BOLD:AAP8428
Grapholita tenebrosana|MM17901|629[0n]bp|Finland|BOLD:AAL3224
Grapholita tenebrosana|MM17269|658[0n]bp|Finland|BOLD:AAL3224
Grapholita tenebrosana|MM11043|658[0n]bp|Finland|BOLD:AAL3224
Grapholita tenebrosana|MM02430|658[0n]bp|Finland.South Karelia|BOLD:AAL3224
Grapholita tenebrosana|MM16013|658[0n]bp|Finland|BOLD:AAL3224
Grapholita tenebrosana|MM16012|658[0n]bp|Finland|BOLD:AAL3224
Grapholita tenebrosana|MM17899|658[0n]bp|Finland|BOLD:AAL3224
Grapholita tenebrosana|MM05511|658[0n]bp|Finland.Ostrobothnia|BOLD:AAL3224
Grapholita tenebrosana|MM11040|658[0n]bp|Finland|BOLD:AAL3224
Grapholita tenebrosana|TLMF Lep 08097|658[0n]bp|Austria.Vorarlberg|BOLD:AAD6968
Grapholita tenebrosana|MM17268|658[0n]bp|Finland|BOLD:AAD6968
Grapholita tenebrosana|MM17897|658[0n]bp|Finland|BOLD:AAD6968
Grapholita tenebrosana|MM17938|658[0n]bp|Finland|BOLD:AAD6968
Grapholita tenebrosana|MM06873|658[0n]bp|Finland.Aland Islands|BOLD:AAD6968
Grapholita tenebrosana|MM17898|658[0n]bp|Finland|BOLD:AAD6968
Grapholita tenebrosana|MM17900|658[0n]bp|Finland|BOLD:AAD6968
Grapholita tenebrosana|MM06874|658[0n]bp|Finland.Aland Islands|BOLD:AAD6968
Grapholita tenebrosana|MM06472|658[0n]bp|Finland|BOLD:AAD6968
Grapholita tenebrosana|MM18612|658[0n]bp|Finland.Aland Islands|BOLD:AAD6968
Grapholita tenebrosana|MM10360|658[0n]bp|Finland|BOLD:AAD6968
Grapholita tenebrosana|MM21170|658[0n]bp|Finland|BOLD:AAD6968
Grapholita tenebrosana|MM17323|658[0n]bp|Finland|BOLD:AAD6968
Grapholita tenebrosana|MM11042|658[0n]bp|Finland|BOLD:AAD6968
Grapholita tenebrosana|MM16014|658[0n]bp|Finland|BOLD:AAD6968
Grapholita tenebrosana|MM11041|658[0n]bp|Finland|BOLD:AAD6968
Grapholita tenebrosana|MM22900|658[0n]bp|Finland|BOLD:AAD6968
Grapholita tenebrosana|MM17896|658[0n]bp|Finland|BOLD:AAD6968
Cydia nigricana|MM05216|633[0n]bp|Finland.Finland Proper|BOLD:AAA7614
Cydia nigricana|MM17902|658[0n]bp|Finland|BOLD:AAA7614
Cydia nigricana|MM21167|658[0n]bp|Finland|BOLD:AAA7614
Cydia nigricana|TLMF Lep 04289|658[0n]bp|Austria.Vorarlberg|BOLD:AAA7614
Cydia nigricana|MM17903|658[1n]bp|Finland|BOLD:AAA7614
Cydia nigricana|MM03888|670[0n]bp|Finland.South Karelia|BOLD:AAA7614
Cydia nigricana|MM09437|658[0n]bp|Finland.Aland Islands|BOLD:AAA7614
Cydia nigricana|MM00968|658[0n]bp|Finland.Northern Ostrobothnia|BOLD:AAA7614
Cydia nigricana|MM00967|626[0n]bp|Finland.Northern Ostrobothnia|BOLD:AAA7614
Cydia nigricana|MM17895|658[0n]bp|Finland|BOLD:AAA7614
Cydia succedana|MM18349|658[0n]bp|Finland|BOLD:AAB7159
Cydia succedana|TLMF Lep 07699|658[0n]bp|Austria|BOLD:AAB7159
Cydia succedana|MM11038|658[0n]bp|Finland|BOLD:AAB7159
Cydia succedana|MM18351|658[0n]bp|Finland|BOLD:AAB7159
Cydia succedana|MM18350|658[0n]bp|Finland|BOLD:ACF3585
Cydia succedana|MM11037|658[0n]bp|Finland|BOLD:ACF3585
Lathronympha strigana|TLMF Lep 12537|658[0n]bp|Austria.Vorarlberg|BOLD:AAC1866
Lathronympha strigana|MM08966|658[0n]bp|Finland|BOLD:AAC1866
Lathronympha strigana|MM02083|658[0n]bp|Finland.South Karelia|BOLD:AAC1866
Lathronympha strigana|MM14471|658[0n]bp|Finland|BOLD:AAC1866
Lathronympha strigana|MM05266|636[0n]bp|Finland.Finland Proper|BOLD:AAC1866
Lathronympha strigana|MM09440|658[0n]bp|Finland.Aland Islands|BOLD:AAC1866
Pammene fasciana|MM09774|658[0n]bp|Finland.Aland Islands|BOLD:AAC8302
Pammene fasciana|MM03827|658[0n]bp|Finland.Aland Islands|BOLD:AAC8302
Pammene fasciana|MM09773|658[0n]bp|Finland.Aland Islands|BOLD:AAC8302
Pammene fasciana|TLMF Lep 09773|658[0n]bp|Austria.Vorarlberg|BOLD:AAC8302
Pammene ignorata|MM04310|658[0n]bp|Finland.Uusimaa|BOLD:AAF1865
Pammene ignorata|MM09253|658[0n]bp|Finland|BOLD:AAF1865
Pammene ignorata|MM17606|658[0n]bp|Finland|BOLD:AAF1865
Pammene ignorata|MM11044|658[0n]bp|Finland|BOLD:AAF1865
Pammene ignorata|MM18894|658[0n]bp|Finland.Aland Islands|BOLD:AAF1865

[illegible]
Pammene ignorata|MM17606|658[0n]bp|Finland|BOLD:AAF1865
Pammene ignorata|MM11044|658[0n]bp|Finland|BOLD:AAF1865
Pammene ignorata|MM18894|658[0n]bp|Finland.Aland Islands|BOLD:AAF1865
Pammene ignorata|MM18644|658[0n]bp|Finland|BOLD:AAF1865
Pammene ignorata|MM05217|640[0n]bp|Finland.Finland Proper|BOLD:AAF1865
Pammene ignorata|TLMF Lep 09772|630[0n]bp|Austria.Vorarlberg|BOLD:AAF1865
Pammene ochsenheimeriana|MM18359|658[0n]bp|Finland|BOLD:AAF1961
Pammene ochsenheimeriana|MM09844|658[0n]bp|Finland|BOLD:AAF1961
Pammene ochsenheimeriana|TLMF Lep 09982|654[0n]bp|Austria.Vorarlberg|BOLD:AAF1961
Pammene ochsenheimeriana|MM09843|658[1n]bp|Finland|BOLD:AAF1961
Eucosma cana|MM13186|658[0n]bp|Finland|BOLD:AAB4296
Eucosma cana|MM08961|658[0n]bp|Finland|BOLD:AAB4296
Eucosma cana|MM05258|644[0n]bp|Finland.Finland Proper|BOLD:AAB4296
Eucosma cana|MM03064|522[0n]bp|Finland.Northern Ostrobothnia|BOLD:AAB4296
Eucosma cana|MM11025|643[0n]bp|Finland.Aland Islands|BOLD:AAB4296
Eucosma cana|MM11834|658[0n]bp|Finland|BOLD:AAB4296
Eucosma cana|TLMF Lep 08460|658[0n]bp|Austria.Vorarlberg|BOLD:AAB4296
Eucosma cana|MM02107|658[0n]bp|Finland.South Karelia|BOLD:AAB4296
Eucosma cana|MM06661|658[0n]bp|Finland|BOLD:AAB4296
Eucosma cana|MM08963|658[0n]bp|Finland|BOLD:AAB4296
Eucosma conterminana|MM09536|658[0n]bp|Finland.Aland Islands|BOLD:AAC3321
Eucosma conterminana|TLMF Lep 08443|658[0n]bp|Austria.Vorarlberg|BOLD:AAC3321
Eucosma conterminana|MM22624|658[0n]bp|Finland|BOLD:AAC3321
Eucosma conterminana|MM04346|658[0n]bp|Finland.Finland Proper|BOLD:AAC3321
Eucosma conterminana|MM06887|658[0n]bp|Finland.Aland Islands|BOLD:AAC3321
Eucosma hohenwartiana|MM22621|658[0n]bp|Finland|BOLD:AAB4295
Eucosma hohenwartiana|MM22623|658[0n]bp|Finland|BOLD:AAB4295
Eucosma hohenwartiana|MM09282|658[0n]bp|Finland|BOLD:AAB4295
Eucosma hohenwartiana|MM09216|658[0n]bp|Finland|BOLD:AAB4295
Eucosma hohenwartiana|TLMF Lep 08036|658[0n]bp|Austria.Vorarlberg|BOLD:AAB4295
Eucosma hohenwartiana|MM09215|658[0n]bp|Finland|BOLD:AAB4295
Eucosma hohenwartiana|MM22622|658[1n]bp|Finland|BOLD:AAB4295
Eucosma hohenwartiana|MM09439|658[0n]bp|Finland.Aland Islands|BOLD:AAB4295
Eucosma hohenwartiana|MM14344|658[0n]bp|Finland.Aland Islands|BOLD:AAB4295
Eucosma hohenwartiana|MM09283|658[0n]bp|Finland|BOLD:AAB4295
Eucosma hohenwartiana|MM21209|658[0n]bp|Finland|BOLD:AAB4295
Eucosma hohenwartiana|MM11020|658[0n]bp|Finland|BOLD:AAB4295
Eucosma hohenwartiana|MM11022|658[0n]bp|Finland|BOLD:AAB4295
Eucosma hohenwartiana|MM06899|658[0n]bp|Finland.Aland Islands|BOLD:AAB4295
Eucosma hohenwartiana|TLMF Lep 08461|658[0n]bp|Austria.Vorarlberg|BOLD:AAB4295
Eucosma hohenwartiana|MM17240|658[0n]bp|Finland|BOLD:AAB4295
Eucosma hohenwartiana|MM12143|658[0n]bp|Finland|BOLD:AAB4295
Eucosma hohenwartiana|MM13764|658[0n]bp|Finland|BOLD:AAB4295
Eucosma hohenwartiana|MM12320|658[0n]bp|Finland|BOLD:AAB4295
Eucosma hohenwartiana|MM09451|658[0n]bp|Finland.Aland Islands|BOLD:AAB4295
Eucosma hohenwartiana|MM06631|658[0n]bp|Finland|BOLD:AAB4295
Eucosma hohenwartiana|MM08964|658[0n]bp|Finland|BOLD:AAB4295
Eucosma hohenwartiana|MM14505|658[0n]bp|Finland|BOLD:AAB4295
Eucosma hohenwartiana|MM14504|658[0n]bp|Finland|BOLD:AAB4295
Eucosma hohenwartiana|MM14503|658[0n]bp|Finland|BOLD:AAB4295
Eucosma hohenwartiana|MM14502|658[0n]bp|Finland|BOLD:AAB4295
Eucosma hohenwartiana|MM21163|658[0n]bp|Finland|BOLD:AAB4295
Eucosma hohenwartiana|MM14501|639[0n]bp|Finland|BOLD:AAB4295
Eucosma hohenwartiana|MM17239|658[0n]bp|Finland|BOLD:AAB4295
Eucosma hohenwartiana|MM06630|658[0n]bp|Finland|BOLD:AAB4295
Eucosma hohenwartiana|MM21162|658[0n]bp|Finland|BOLD:AAB4295
Eucosma hohenwartiana|MM21161|658[0n]bp|Finland|BOLD:AAB4295
Notocelia cynosbatella|MM22899|658[0n]bp|Finland|BOLD:AAC3246
Notocelia cynosbatella|MM03018|658[0n]bp|Finland.Northern Ostrobothnia|BOLD:AAC3246
Notocelia cynosbatella|MM01972|653[0n]bp|Finland.South Karelia|BOLD:AAC3246
Notocelia cynosbatella|TLMF Lep 07962|658[0n]bp|Austria.Vorarlberg|BOLD:AAC3246
Notocelia cynosbatella|MM13185|658[0n]bp|Finland|BOLD:AAC3246
Notocelia rosaecolana|MM18335|658[0n]bp|Finland|BOLD:AAC1134
Notocelia rosaecolana|TLMF Lep 10028|658[0n]bp|Austria.Vorarlberg|BOLD:AAC1134
Notocelia rosaecolana|MM08020|658[0n]bp|Finland|BOLD:AAC1134
Notocelia rosaecolana|MM08021|658[0n]bp|Finland|BOLD:AAC1134
Notocelia tetragonana|MM19240|658[0n]bp|Finland.Aland Islands|BOLD:AAC0717
Notocelia tetragonana|TLMF Lep 08090|658[0n]bp|Austria.Vorarlberg|BOLD:AAC0717
Notocelia tetragonana|MM19906|658[0n]bp|Finland|BOLD:AAC0717
Notocelia tetragonana|MM19907|658[0n]bp|Finland|BOLD:AAC0717
Epiblema scutulana|MM17266|658[0n]bp|Finland|BOLD:AAP7460
Epiblema scutulana|MM17267|658[0n]bp|Finland|BOLD:AAP7460
Epiblema scutulana|MM14105|658[0n]bp|Finland|BOLD:AAC0715
Epiblema scutulana|MM09212|658[0n]bp|Finland|BOLD:AAC0715
Epiblema scutulana|TLMF Lep 09192|658[0n]bp|Austria.Vorarlberg|BOLD:AAC0715
Epiblema scutulana|MM14107|649[0n]bp|Finland|BOLD:AAC0715
Epiblema scutulana|MM14106|658[0n]bp|Finland|BOLD:AAC0715
Epiblema scutulana|MM14104|658[0n]bp|Finland|BOLD:AAC0715
Epiblema grandaevana|TLMF Lep 08740|658[0n]bp|Austria.Vorarlberg|BOLD:AAF2217
Epiblema grandaevana|MM22090|658[0n]bp|Finland|BOLD:AAF2218
Epiblema grandaevana|MM08704|658[0n]bp|Finland|BOLD:AAF2218
Epiblema grandaevana|MM12415|658[0n]bp|Finland|BOLD:AAF2218
Epiblema grandaevana|MM14216|658[0n]bp|Finland.Aland Islands|BOLD:AAF2218
Epiblema grandaevana|MM22091|658[0n]bp|Finland|BOLD:AAF2218
Epiblema grandaevana|MM22092|658[0n]bp|Finland|BOLD:AAF2218
Epiblema sticticana|TLMF Lep 10054|658[0n]bp|Austria.Vorarlberg|BOLD:AAC0719
Epiblema sticticana|MM09989|658[0n]bp|Finland|BOLD:AAC0719
Epiblema sticticana|MM15727|658[0n]bp|Finland|BOLD:AAC0719
Epiblema sticticana|MM15726|658[0n]bp|Finland|BOLD:AAC0719
Epiblema sticticana|MM10061|658[0n]bp|Finland|BOLD:AAC0719
Pelochrista caecimaculana|MM05917|658[0n]bp|Finland|BOLD:AAE7175
Pelochrista caecimaculana|MM18336|658[0n]bp|Finland|BOLD:AAE7175
Pelochrista caecimaculana|MM18642|658[0n]bp|Finland|BOLD:AAE7175
Pelochrista caecimaculana|TLMF Lep 07509|658[0n]bp|Austria.Vorarlberg|BOLD:AAE7175
Spilonota ocellana|MM03473|658[0n]bp|Finland.Uusimaa|BOLD:ABZ4399
Spilonota ocellana|MM09512|629[0n]bp|Finland.Aland Islands|BOLD:ABZ4399
Spilonota ocellana|MM09718|658[0n]bp|Finland.Aland Islands|BOLD:ABZ4399
Spilonota ocellana|MM03620|621[0n]bp|Finland.Uusimaa|BOLD:ABZ4399
Spilonota ocellana|MM13241|658[0n]bp|Finland|BOLD:ABZ4399
Spilonota ocellana|MM05007|658[0n]bp|Finland.Finland Proper|BOLD:ABZ4399
Spilonota ocellana|MM05985|658[0n]bp|Finland|BOLD:ABZ4399
Spilonota ocellana|MM03558|635[0n]bp|Finland.Kymenlaakso|BOLD:ABZ4399
[illegible]

Spilonota ocellana|MM03007|658[0n]bp|Finland.Finland Proper|BOLD:ABZ4399
Spilonota ocellana|MM05985|658[0n]bp|Finland|BOLD:ABZ4399
Spilonota ocellana|MM03558|635[0n]bp|Finland.Kymenlaakso|BOLD:ABZ4399
Spilonota ocellana|MM02155|658[0n]bp|Finland.South Karelia|BOLD:ABZ4399
Spilonota ocellana|MM09821|658[0n]bp|Finland.Aland Islands|BOLD:ABZ4399
Spilonota ocellana|TLMF Lep 08266|658[0n]bp|Austria.Vorarlberg|BOLD:ABZ4399
Spilonota ocellana|TLMF Lep 10036|658[0n]bp|Austria.Vorarlberg|BOLD:ABZ4399
Dichrorampha alpinana|MM11045|658[0n]bp|Finland|BOLD:ACF3984
Dichrorampha alpinana|TLMF Lep 08427|658[0n]bp|Austria.Vorarlberg|BOLD:AAE7519
Dichrorampha alpinana|MM14448|658[0n]bp|Finland|BOLD:AAE7519
Dichrorampha alpinana|MM11046|658[0n]bp|Finland|BOLD:AAE7519
Dichrorampha simpliciana|MM03494|632[0n]bp|Finland.Uusimaa|BOLD:AAD3189
Dichrorampha simpliciana|MM13320|658[0n]bp|Finland|BOLD:AAD3189
Dichrorampha simpliciana|MM02165|658[0n]bp|Finland.South Karelia|BOLD:AAD3189
Dichrorampha simpliciana|TLMF Lep 07523|658[0n]bp|Austria.Vorarlberg|BOLD:AAD3189
Dichrorampha simpliciana|TLMF Lep 08733|658[0n]bp|Austria.Vorarlberg|BOLD:AAD3189
Epinotia immundana|MM05463|658[0n]bp|Finland.Northern Ostrobothnia|BOLD:AAC9104
Epinotia immundana|MM10063|658[0n]bp|Finland|BOLD:AAC9104
Epinotia immundana|MM18330|658[0n]bp|Finland|BOLD:AAC9104
Epinotia immundana|MM18329|658[0n]bp|Finland|BOLD:AAC9104
Epinotia immundana|MM21198|658[0n]bp|Finland|BOLD:AAC9104
Epinotia immundana|TLMF Lep 07481|658[1n]bp|Austria.Vorarlberg|BOLD:AAC9106
Epinotia immundana|MM02153|658[0n]bp|Finland.South Karelia|BOLD:AAC9106
Epinotia immundana|MM11881|658[0n]bp|Finland|BOLD:AAC9106
Epinotia immundana|MM11865|658[0n]bp|Finland|BOLD:AAC9106
Epinotia abbreviana|MM05009|542[0n]bp|Finland.Finland Proper|BOLD:AAE1784
Epinotia abbreviana|TLMF Lep 08093|658[0n]bp|Austria.Vorarlberg|BOLD:AAE1784
Epinotia abbreviana|MM09747|624[0n]bp|Finland.Aland Islands|BOLD:AAE1784
Epinotia abbreviana|MM09749|658[0n]bp|Finland.Aland Islands|BOLD:AAE1784
Epinotia abbreviana|MM09748|658[0n]bp|Finland.Aland Islands|BOLD:AAE1784
Epinotia subocellana|MM14025|658[0n]bp|Finland|BOLD:AAB8745
Epinotia subocellana|MM08585|658[0n]bp|Finland|BOLD:AAB8745
Epinotia subocellana|MM03832|637[0n]bp|Finland.Aland Islands|BOLD:AAB8745
Epinotia subocellana|TLMF Lep 09983|658[0n]bp|Austria.Vorarlberg|BOLD:AAB8745
Epinotia demarniana|TLMF Lep 09925|658[0n]bp|Austria.Vorarlberg|BOLD:ACJ0697
Epinotia demarniana|MM00759|658[0n]bp|Finland.Northern Ostrobothnia|BOLD:ACJ0697
Epinotia demarniana|MM02125|658[0n]bp|Finland.South Karelia|BOLD:ACJ0697
Epinotia demarniana|MM03202|658[0n]bp|Finland.Northern Ostrobothnia|BOLD:ACJ0697
Epinotia demarniana|MM14227|658[0n]bp|Finland.Aland Islands|BOLD:ACJ0697
Epinotia nanana|BIOUG04118-D10|614[0n]bp|Finland.Northern Ostrobothnia|BOLD:AAA8628
Epinotia nanana|TLMF Lep 08245|658[0n]bp|Austria.Vorarlberg|BOLD:AAA8628
Epinotia nanana|BIOUG04116-A10|614[0n]bp|Finland.Northern Ostrobothnia|BOLD:AAA8628
Epinotia nanana|MM10398|658[0n]bp|Finland.Aland Islands|BOLD:AAA8628
Epinotia nanana|MM00697|658[0n]bp|Finland.Lapland|BOLD:AAA8628
Epinotia nanana|MM10163|639[0n]bp|Finland|BOLD:AAA8628
Epinotia tetraquetrana|TLMF Lep 09201|641[0n]bp|Austria.Vorarlberg|BOLD:AAD1775
Epinotia tetraquetrana|TLMF Lep 09167|658[0n]bp|Austria.Vorarlberg|BOLD:AAD1775
Epinotia tetraquetrana|MM15993|658[0n]bp|Finland|BOLD:AAD1775
Epinotia tetraquetrana|MM00543|658[0n]bp|Finland.Northern Ostrobothnia|BOLD:AAD1775
Epinotia tetraquetrana|MM00544|658[0n]bp|Finland.Northern Ostrobothnia|BOLD:AAD1775
Epinotia tetraquetrana|MM02102|658[0n]bp|Finland.South Karelia|BOLD:AAD1775
Epinotia granitana|MM18325|632[0n]bp|Finland|BOLD:AAJ0675
Epinotia granitana|MM15719|658[0n]bp|Finland|BOLD:AAJ0675
Epinotia granitana|MM18326|658[0n]bp|Finland|BOLD:AAJ0675
Epinotia granitana|TLMF Lep 07490|658[0n]bp|Austria.Vorarlberg|BOLD:AAJ0675
Epinotia granitana|MM21060|658[0n]bp|Finland|BOLD:AAJ0675
Epinotia tedella|TLMF Lep 07938|658[0n]bp|Austria.Vorarlberg|BOLD:AAD1782
Epinotia tedella|MM02139|632[0n]bp|Finland.South Karelia|BOLD:AAD1782
Epinotia tedella|MM13338|658[0n]bp|Finland|BOLD:AAD1782
Epinotia tedella|MM05262|641[0n]bp|Finland.Finland Proper|BOLD:AAD1782
Epinotia bilunana|TLMF Lep 09931|658[0n]bp|Austria.Vorarlberg|BOLD:AAD1759
Epinotia bilunana|MM15718|658[0n]bp|Finland|BOLD:AAD1759
Epinotia bilunana|MM15717|658[0n]bp|Finland|BOLD:AAD1759
Epinotia bilunana|MM15716|658[0n]bp|Finland|BOLD:AAD1759
Epinotia cruciana|MM06544|658[0n]bp|Finland|BOLD:AAC2644
Epinotia cruciana|MM03323|658[0n]bp|Finland.Northern Ostrobothnia|BOLD:AAC2644
Epinotia cruciana|MM08349|658[0n]bp|Finland|BOLD:AAC2644
Epinotia cruciana|TLMF Lep 00798|658[0n]bp|Austria.Vorarlberg|BOLD:AAC2644
Epinotia cruciana|MM06921|658[0n]bp|Finland|BOLD:AAC2644
Epinotia mercuriana|TLMF Lep 03671|658[0n]bp|Austria.Vorarlberg|BOLD:AAE1186
Epinotia mercuriana|MM18327|658[0n]bp|Finland|BOLD:AAE1187
Epinotia mercuriana|MM06048|658[0n]bp|Finland|BOLD:AAE1187
Epinotia mercuriana|MM14635|658[0n]bp|Finland|BOLD:AAE1187
Epinotia nisella|MM12340|658[0n]bp|Finland|BOLD:AAA7530
Epinotia nisella|MM11880|658[0n]bp|Finland|BOLD:AAA7530
Epinotia nisella|MM08949|658[0n]bp|Finland|BOLD:AAA7530
Epinotia nisella|MM00758|658[0n]bp|Finland.Northern Ostrobothnia|BOLD:AAA7530
Epinotia nisella|MM08948|658[0n]bp|Finland|BOLD:AAA7530
Epinotia nisella|MM18331|658[0n]bp|Finland|BOLD:AAA7530
Epinotia nisella|MM12338|658[0n]bp|Finland|BOLD:AAA7530
Epinotia nisella|MM12339|658[0n]bp|Finland|BOLD:AAA7530
Epinotia nisella|MM13308|658[0n]bp|Finland|BOLD:AAA7530
Epinotia nisella|MM12341|658[0n]bp|Finland|BOLD:AAA7530
Epinotia nisella|TLMF Lep 08463|658[0n]bp|Austria.Vorarlberg|BOLD:AAA7530
Epinotia ramella|MM03027|658[0n]bp|Finland|BOLD:AAC6083
Epinotia ramella|MM00681|658[0n]bp|Finland.Lapland|BOLD:AAC6083
Epinotia ramella|MM08215|658[0n]bp|Finland|BOLD:AAC6083
Epinotia ramella|MM05020|658[0n]bp|Finland.Finland Proper|BOLD:AAC6083
Epinotia ramella|MM08933|658[0n]bp|Finland|BOLD:ACE7003
Epinotia ramella|TLMF Lep 08748|658[0n]bp|Austria.Vorarlberg|BOLD:ACE9771
Epinotia ramella|MM01966|658[0n]bp|Finland|BOLD:ACE9771
Epinotia trigonella|MM04647|658[0n]bp|Finland.Finland Proper|BOLD:AAB2504
Epinotia trigonella|TLMF Lep 08744|658[0n]bp|Austria.Vorarlberg|BOLD:AAB2504
Epinotia trigonella|MM18321|658[0n]bp|Finland|BOLD:AAB2504
Epinotia trigonella|MM02047|658[0n]bp|Finland.South Karelia|BOLD:AAB2504
Epinotia trigonella|MM13231|658[0n]bp|Finland|BOLD:AAB2504
Epinotia trigonella|MM12351|658[0n]bp|Finland|BOLD:AAB2504
Rhyacionia pinicolana|TLMF Lep 00923|658[0n]bp|Austria.Vorarlberg|BOLD:AAC4601
Rhyacionia pinicolana|TLMF Lep 00916|658[0n]bp|Austria.Vorarlberg|BOLD:AAC4601
Rhyacionia pinicolana|MM02096|658[0n]bp|Finland.South Karelia|BOLD:AAC4601
Rhyacionia pinicolana|TLMF Lep 08080|658[0n]bp|Austria.Vorarlberg|BOLD:AAC4601
Rhyacionia pinicolana|MM03110|658[0n]bp|Finland.Northern Ostrobothnia|BOLD:AAC4601
Rhyacionia pinicolana|MM09576|658[0n]bp|Finland.Aland Islands|BOLD:AAC4601

Rhyacionia pinicolana|TLMF Lep 08080|658[0n]bp|Austria.Vorarlberg|BOLD:AAC4601
Rhyacionia pinicolana|MM03110|658[0n]bp|Finland.Northern Ostrobothnia|BOLD:AAC4601
Rhyacionia pinicolana|MM09576|658[0n]bp|Finland.Aland Islands|BOLD:AAC4601
Rhyacionia pinivorana|TLMF Lep 07925|658[0n]bp|Austria.Vorarlberg|BOLD:AAB9878
Rhyacionia pinivorana|MM14337|658[0n]bp|Finland.Aland Islands|BOLD:AAB9878
Rhyacionia pinivorana|MM00677|658[0n]bp|Finland.Lapland|BOLD:AAB9878
Rhyacionia pinivorana|MM02147|658[0n]bp|Finland.South Karelia|BOLD:AAB9878
Rhyacionia pinivorana|MM03224|658[0n]bp|Finland.Northern Ostrobothnia|BOLD:AAB9878
Notocelia uddmanniana|MM03016|648[0n]bp|Finland.Northern Ostrobothnia|BOLD:AAC1148
Notocelia uddmanniana|MM01970|638[0n]bp|Finland.South Karelia|BOLD:AAC1148
Notocelia uddmanniana|MM13212|571[0n]bp|Finland|BOLD:AAC1148
Notocelia uddmanniana|TLMF Lep 08024|658[0n]bp|Austria.Vorarlberg|BOLD:AAC1148
Zeiraphera isertana|TLMF Lep 08047|658[0n]bp|Austria.Vorarlberg|BOLD:AAD1936
Zeiraphera isertana|MM03618|658[0n]bp|Finland.Uusimaa|BOLD:AAD1936
Zeiraphera isertana|MM10364|658[0n]bp|Finland|BOLD:AAD1936
Zeiraphera isertana|MM05011|632[0n]bp|Finland.Finland Proper|BOLD:AAD1936
Zeiraphera ratzeburgiana|TLMF Lep 08230|658[0n]bp|Austria.Vorarlberg|BOLD:AAD8502
Zeiraphera ratzeburgiana|MM00699|658[0n]bp|Finland.Lapland|BOLD:ACF5190
Zeiraphera ratzeburgiana|BIOUG04116-B11|663[0n]bp|Finland.Northern Ostrobothnia|BOLD:ACF5190
Zeiraphera ratzeburgiana|MM08972|658[0n]bp|Finland|BOLD:ACF5190
Zeiraphera ratzeburgiana|MM13314|658[0n]bp|Finland|BOLD:ACF5190
Bucculatrix nigricomella|TLMF Lep 08408|658[0n]bp|Austria.Vorarlberg|BOLD:AAE8470
Bucculatrix nigricomella|MM09948|658[0n]bp|Finland|BOLD:AAE8470
Bucculatrix nigricomella|MM06615|658[0n]bp|Finland|BOLD:AAE8470
Bucculatrix nigricomella|MM02626|658[0n]bp|Finland.South Karelia|BOLD:AAE8470
Bucculatrix bechsteinella|MM06181|658[0n]bp|Finland|BOLD:AAE8504
Bucculatrix bechsteinella|TLMF Lep 08482|658[0n]bp|Austria.Vorarlberg|BOLD:AAE8504
Bucculatrix bechsteinella|MM02642|658[0n]bp|Finland.South Karelia|BOLD:AAE8504
Bucculatrix bechsteinella|MM14829|658[0n]bp|Finland|BOLD:AAE8504
Bucculatrix bechsteinella|MM13757|658[0n]bp|Finland|BOLD:AAE8504
Bucculatrix cidarella|TLMF Lep 08233|658[0n]bp|Austria.Vorarlberg|BOLD:AAU3108
Bucculatrix cidarella|MM05732|632[0n]bp|Finland.North Karelia|BOLD:AAI3853
Bucculatrix cidarella|MM08606|658[0n]bp|Finland|BOLD:AAI3853
Bucculatrix cidarella|MM16607|658[0n]bp|Finland|BOLD:AAI3853
Bucculatrix cidarella|MM02655|658[0n]bp|Finland.South Karelia|BOLD:AAI3853
Bucculatrix cidarella|MM06180|658[0n]bp|Finland|BOLD:AAI3853
Bucculatrix demaryella|TLMF Lep 07476|658[0n]bp|Austria.Vorarlberg|BOLD:AAI8481
Bucculatrix demaryella|TLMF Lep 08074|658[0n]bp|Austria.Vorarlberg|BOLD:AAI8481
Bucculatrix demaryella|MM05482|670[0n]bp|Finland.Northern Ostrobothnia|BOLD:AAI8481
Bucculatrix demaryella|MM06182|658[0n]bp|Finland|BOLD:AAI8481
Bucculatrix demaryella|MM23175|658[0n]bp|Finland|BOLD:AAI8481
Bucculatrix demaryella|BIOUG04118-A06|621[0n]bp|Finland.Northern Ostrobothnia|BOLD:AAI8481
Bucculatrix demaryella|BIOUG04118-A01|613[0n]bp|Finland.Northern Ostrobothnia|BOLD:AAI8481
Bucculatrix demaryella|MM15169|658[0n]bp|Finland|BOLD:AAI8481
Bucculatrix thoracella|MM18084|658[0n]bp|Finland|BOLD:AAE8468
Bucculatrix thoracella|MM15537|534[0n]bp|Finland|BOLD:AAE8468
Bucculatrix thoracella|TLMF Lep 07955|658[0n]bp|Austria.Vorarlberg|BOLD:AAE8468
Bucculatrix thoracella|MM15538|658[0n]bp|Finland.Aland Islands|BOLD:AAE8468
Scythris obscurella|TLMF Lep 04510|651[0n]bp|Austria.Vorarlberg|BOLD:AAF1117
Scythris obscurella|MM18204|658[0n]bp|Finland|BOLD:AAF1117
Scythris obscurella|MM06455|658[0n]bp|Finland|BOLD:AAF1117
Scythris obscurella|MM06456|658[0n]bp|Finland|BOLD:AAF1117
Spargania luctuata|TLMF Lep 07567|658[0n]bp|Austria.Vorarlberg|BOLD:AAA2806
Spargania luctuata|MM01391|636[0n]bp|Finland.South Karelia|BOLD:AAA2806
Spargania luctuata|MM01392|606[0n]bp|Finland.South Karelia|BOLD:AAA2806
Spargania luctuata|MM08455|658[0n]bp|Finland|BOLD:AAA2806
Deilephila elpenor|MM00971|658[0n]bp|Finland.South Karelia|BOLD:AAB2861
Deilephila elpenor|MM05160|623[0n]bp|Finland.Finland Proper|BOLD:AAB2861
Deilephila elpenor|MM07300|658[0n]bp|Finland|BOLD:AAB2861
Deilephila elpenor|TLMF Lep 08527|658[0n]bp|Austria.Vorarlberg|BOLD:AAB2861
Hyles gallii|TLMF Lep 12458|658[0n]bp|Austria.Vorarlberg|BOLD:ACE4003
Hyles gallii|MM12530|658[0n]bp|Finland|BOLD:ACE4003
Hyles gallii|MM05084|670[0n]bp|Finland.Finland Proper|BOLD:ACE4003
Hyles gallii|TLMF Lep 09504|658[0n]bp|Austria.Vorarlberg|BOLD:ACE4003
Hyles gallii|MM10703|658[0n]bp|Finland|BOLD:ACE4003
Laothoe populi|MM00976|670[0n]bp|Finland.South Karelia|BOLD:AAB3599
Laothoe populi|MM12527|658[0n]bp|Finland|BOLD:AAB3599
Laothoe populi|MM02803|658[0n]bp|Finland.Northern Ostrobothnia|BOLD:AAB3599
Laothoe populi|MM00977|658[0n]bp|Finland.South Karelia|BOLD:AAB3599
Laothoe populi|TLMF Lep 08118|658[0n]bp|Austria.Vorarlberg|BOLD:AAB3599
Hemaris fuciformis|MM17191|658[0n]bp|Finland|BOLD:AAB5591
Hemaris fuciformis|MM05477|658[0n]bp|Finland|BOLD:AAB5591
Hemaris fuciformis|MM17192|625[1n]bp|Finland|BOLD:AAB5591
Hemaris fuciformis|TLMF Lep 09848|658[0n]bp|Austria.Vorarlberg|BOLD:AAB5591
Hemaris tityus|MM17190|658[0n]bp|Finland|BOLD:ACE7540
Hemaris tityus|TLMF Lep 09857|658[0n]bp|Austria.Vorarlberg|BOLD:ACE7540
Hemaris tityus|MM08732|658[0n]bp|Finland|BOLD:ACE7540
Hemaris tityus|MM08731|658[0n]bp|Finland|BOLD:ACE7540
Mimas tiliae|TLMF Lep 07822|658[0n]bp|Austria.Vorarlberg|BOLD:AAB6049
Mimas tiliae|MM00969|658[0n]bp|Finland.South Karelia|BOLD:AAB6050
Mimas tiliae|MM04540|658[0n]bp|Finland.Finland Proper|BOLD:AAB6050
Mimas tiliae|MM12516|658[0n]bp|Finland|BOLD:AAB6050
Sphinx ligustri|MM06633|658[0n]bp|Finland|BOLD:AAB6107
Sphinx ligustri|MM14214|658[0n]bp|Finland.Aland Islands|BOLD:AAB6107
Sphinx ligustri|TLMF Lep 07820|658[0n]bp|Austria.Vorarlberg|BOLD:AAB6107
Sphinx pinastri|TLMF Lep 07821|658[0n]bp|Austria.Vorarlberg|BOLD:AAA8863
Sphinx pinastri|MM00979|658[0n]bp|Finland.South Karelia|BOLD:AAA8863
Sphinx pinastri|MM00978|658[0n]bp|Finland.South Karelia|BOLD:AAA8863
Sphinx pinastri|MM13861|658[0n]bp|Finland.Lapland|BOLD:AAA8863
Glyphipterix forsterella|MM21172|658[0n]bp|Finland|BOLD:AAD7071
Glyphipterix forsterella|MM21186|658[0n]bp|Finland|BOLD:AAD7071
Glyphipterix forsterella|MM21166|658[0n]bp|Finland|BOLD:AAD7071
Glyphipterix forsterella|MM23200|658[0n]bp|Finland|BOLD:AAD7071
Glyphipterix forsterella|TLMF Lep 12491|658[0n]bp|Austria.Vorarlberg|BOLD:AAD7071
Glyphipterix forsterella|MM15546|658[0n]bp|Finland|BOLD:AAD7072
Glyphipterix forsterella|MM04000|658[0n]bp|Finland.North Karelia|BOLD:AAD7072
Glyphipterix forsterella|MM15545|658[0n]bp|Finland|BOLD:AAD7072
Anthophila fabriciana|MM02342|658[0n]bp|Finland.South Karelia|BOLD:AAC8582
Anthophila fabriciana|TLMF Lep 03622|658[0n]bp|Austria.Vorarlberg|BOLD:AAC8583
Anthophila fabriciana|MM03284|630[0n]bp|Finland.Northern Ostrobothnia|BOLD:AAC8583
Anthophila fabriciana|MM05279|650[0n]bp|Finland.Finland Proper|BOLD:AAC8583
Glyphipterix thrasonella|MM09794|658[0n]bp|Finland.Aland Islands|BOLD:AAD6735
Glyphipterix thrasonella|MM14262|658[0n]bp|Finland.Aland Islands|BOLD:AAD6735

Anthophila fabriciana|MM05284|650[0n]bp|Finland.Northern Ostrobothnia|BOLD:AAC8583
Anthophila fabriciana|MM05279|650[0n]bp|Finland.Finland Proper|BOLD:AAC8583
Glyphipterix thrasonella|MM09794|658[0n]bp|Finland.Aland Islands|BOLD:AAD6735
Glyphipterix thrasonella|MM14262|658[0n]bp|Finland.Aland Islands|BOLD:AAD6735
Glyphipterix thrasonella|MM09594|658[0n]bp|Finland.Aland Islands|BOLD:AAD6735
Glyphipterix thrasonella|TLMF Lep 09204|563[1n]bp|Austria.Vorarlberg|BOLD:AAD6735
Parachronistis albiceps|TLMF Lep 08258|658[0n]bp|Austria.Vorarlberg|BOLD:ACA9804
Parachronistis albiceps|MM06038|658[0n]bp|Finland|BOLD:ACO2214
Parachronistis albiceps|MM06876|658[0n]bp|Finland.Aland Islands|BOLD:ACO2214
Parachronistis albiceps|MM09735|658[0n]bp|Finland.Aland Islands|BOLD:ACO2214
Parachronistis albiceps|MM09445|658[0n]bp|Finland.Aland Islands|BOLD:ACO2214
Parachronistis albiceps|MM06737|658[0n]bp|Finland.Aland Islands|BOLD:ACO2214
Parachronistis albiceps|MM09224|658[0n]bp|Finland|BOLD:ACO2214
Roeslerstammia erxlebella|TLMF Lep 08240|658[0n]bp|Austria.Vorarlberg|BOLD:AAE7694
Roeslerstammia erxlebella|MM15528|658[0n]bp|Finland|BOLD:AAE7694
Roeslerstammia erxlebella|MM15529|658[0n]bp|Finland|BOLD:AAE7694
Roeslerstammia erxlebella|MM00303|609[0n]bp|Finland.Northern Ostrobothnia|BOLD:AAE7694
Roeslerstammia erxlebella|MM10170|658[0n]bp|Finland|BOLD:AAE7694
Roeslerstammia erxlebella|MM21113|658[0n]bp|Finland|BOLD:AAE7694
Psyche casta|MM06274|658[0n]bp|Finland|BOLD:ACL8669
Psyche casta|MM10019|658[0n]bp|Finland|BOLD:ACL8669
Psyche casta|MM21036|658[0n]bp|Finland|BOLD:ACL8669
Psyche casta|MM06388|631[0n]bp|Finland|BOLD:AAC5848
Psyche casta|MM00049|658[0n]bp|Finland.South Karelia|BOLD:AAC5848
Psyche casta|MM08505|658[0n]bp|Finland|BOLD:AAC5848
Psyche casta|MM08506|658[0n]bp|Finland|BOLD:AAC5848
Psyche casta|MM08593|658[0n]bp|Finland|BOLD:AAC5848
Psyche casta|MM06556|658[0n]bp|Finland|BOLD:AAC5848
Psyche casta|MM12066|658[0n]bp|Finland|BOLD:AAC5848
Psyche casta|MM14890|658[0n]bp|Finland|BOLD:AAC5848
Psyche casta|MM18619|658[0n]bp|Finland|BOLD:AAC5848
Psyche casta|MM14342|658[0n]bp|Finland.Aland Islands|BOLD:AAC5848
Psyche casta|MM21035|658[0n]bp|Finland|BOLD:AAC5848
Psyche casta|MM14341|658[0n]bp|Finland.Aland Islands|BOLD:AAC5848
Psyche casta|MM21175|658[0n]bp|Finland|BOLD:AAC5848
Psyche casta|MM21160|658[0n]bp|Finland|BOLD:AAC5848
Psyche casta|MM06387|670[0n]bp|Finland.Northern Ostrobothnia|BOLD:AAC5848
Psyche casta|MM21034|658[0n]bp|Finland|BOLD:AAC5848
Psyche casta|TLMF Lep 10014|658[0n]bp|Austria.Vorarlberg|BOLD:AAC5848
Psyche casta|TLMF Lep 09901|658[0n]bp|Austria.Vorarlberg|BOLD:AAC5848
Psyche crassiorella|MM13248|611[0n]bp|Finland|BOLD:AAC5849
Psyche crassiorella|MM12065|658[0n]bp|Finland|BOLD:AAC5849
Psyche crassiorella|MM13249|652[0n]bp|Finland|BOLD:AAC5849
Psyche crassiorella|MM13247|658[0n]bp|Finland|BOLD:AAC5849
Psyche crassiorella|MM13251|656[0n]bp|Finland|BOLD:AAC5849
Psyche crassiorella|TLMF Lep 09892|658[0n]bp|Austria.Vorarlberg|BOLD:AAD3657
Psyche crassiorella|MM13250|658[0n]bp|Finland|BOLD:AAD3657
Sterrhopterix standfussi|MM14127|658[0n]bp|Finland|BOLD:AAI0085
Sterrhopterix standfussi|MM17518|658[0n]bp|Finland|BOLD:AAI0085
Sterrhopterix standfussi|MM08501|658[0n]bp|Finland|BOLD:AAI0085
Sterrhopterix standfussi|MM08502|658[0n]bp|Finland|BOLD:AAI0085
Sterrhopterix standfussi|TLMF Lep 09505|658[0n]bp|Austria.Vorarlberg|BOLD:AAI0085
Bembecia ichneumoniformis|TLMF Lep 09865|658[0n]bp|Austria.Vorarlberg|BOLD:AAB7131
Bembecia ichneumoniformis|MM21191|658[0n]bp|Finland|BOLD:AAB7131
Bembecia ichneumoniformis|MM21231|658[0n]bp|Finland|BOLD:AAB7131
Bembecia ichneumoniformis|MM21192|658[0n]bp|Finland|BOLD:AAB7131
Synanthedon formicaeformis|TLMF Lep 09871|658[0n]bp|Austria.Vorarlberg|BOLD:AAC4578
Synanthedon formicaeformis|MM15760|658[0n]bp|Finland|BOLD:ABZ6492
Synanthedon formicaeformis|MM08402|658[0n]bp|Finland|BOLD:ABZ6492
Synanthedon formicaeformis|MM08399|658[0n]bp|Finland|BOLD:ABZ6492
Synanthedon formicaeformis|MM18794|658[0n]bp|Finland|BOLD:ABZ6492
Synanthedon formicaeformis|MM00673|633[0n]bp|Finland.Lapland|BOLD:ABZ6492
Synanthedon formicaeformis|MM18789|658[0n]bp|Finland|BOLD:ABZ6492
Synanthedon spheciformis|TLMF Lep 09872|658[0n]bp|Austria.Vorarlberg|BOLD:AAE2758
Synanthedon spheciformis|MM17408|658[0n]bp|Finland|BOLD:AAE2758
Synanthedon spheciformis|MM17878|658[0n]bp|Finland|BOLD:AAE2758
Synanthedon spheciformis|MM17877|658[0n]bp|Finland|BOLD:AAE2758
Synanthedon spheciformis|MM15758|639[0n]bp|Finland|BOLD:AAE2758
Synanthedon spheciformis|MM18790|658[0n]bp|Finland|BOLD:AAE2758
Synanthedon spheciformis|MM17407|658[0n]bp|Finland|BOLD:AAE2758
Synanthedon tipuliformis|TLMF Lep 09870|658[0n]bp|Austria.Vorarlberg|BOLD:AAC1840
Synanthedon tipuliformis|MM17410|658[0n]bp|Finland|BOLD:AAC1840
Synanthedon tipuliformis|MM00117|658[0n]bp|Finland.Uusimaa|BOLD:AAC1840
Digitivalva reticulella|TLMF Lep 04506|658[0n]bp|Austria.Vorarlberg|BOLD:AAE8385
Digitivalva reticulella|MM06606|658[0n]bp|Finland|BOLD:AAE8385
Digitivalva reticulella|MM18120|658[0n]bp|Finland|BOLD:AAE8385
Digitivalva reticulella|MM06036|658[1n]bp|Finland|BOLD:AAE8385
Callisto coffeella|TLMF Lep 00939|658[0n]bp|Austria.Vorarlberg|BOLD:AAE0738
Callisto coffeella|MM08573|658[0n]bp|Finland|BOLD:AAE0738
Callisto coffeella|MM18091|658[0n]bp|Finland|BOLD:AAE0738
Callisto coffeella|MM08523|658[0n]bp|Finland|BOLD:AAE0738
Callisto coffeella|MM08522|658[0n]bp|Finland|BOLD:AAE0738
Parornix betulae|MM17974|658[0n]bp|Finland|BOLD:AAE3418
Parornix betulae|TLMF Lep 09967|658[0n]bp|Austria.Vorarlberg|BOLD:AAE3418
Parornix betulae|MM06299|658[0n]bp|Finland|BOLD:AAE3418
Parornix betulae|MM06186|658[0n]bp|Finland|BOLD:ABZ4246
Parornix betulae|MM03970|552[0n]bp|Finland.Uusimaa|BOLD:ABZ4246
Parornix betulae|MM02603|516[0n]bp|Finland.South Karelia|BOLD:ABZ4246
Parornix devoniella|MM07010|658[0n]bp|Finland|BOLD:AAD1352
Parornix devoniella|TLMF Lep 07495|658[0n]bp|Austria.Vorarlberg|BOLD:AAD1352
Parornix devoniella|TLMF Lep 07947|658[0n]bp|Austria.Vorarlberg|BOLD:AAD1352
Parornix devoniella|MM09610|658[0n]bp|Finland.Aland Islands|BOLD:AAD1352
Calybites phasianipennella|TLMF Lep 08452|658[0n]bp|Austria.Vorarlberg|BOLD:AAD6298
Calybites phasianipennella|MM00437|658[0n]bp|Finland|BOLD:AAD6298
Calybites phasianipennella|MM10405|658[0n]bp|Finland.Aland Islands|BOLD:AAD6298
Aspilapteryx tringipennella|TLMF Lep 07467|658[0n]bp|Austria.Vorarlberg|BOLD:AAD1923
Aspilapteryx tringipennella|MM13728|658[0n]bp|Finland|BOLD:AAD1923
Aspilapteryx tringipennella|MM09218|658[0n]bp|Finland|BOLD:AAD1923
Aspilapteryx tringipennella|MM14366|658[0n]bp|Finland.Aland Islands|BOLD:AAD1923
Aspilapteryx tringipennella|TLMF Lep 04421|628[0n]bp|Austria.Vorarlberg|BOLD:AAD1923
Caloptilia elongella|TLMF Lep 07958|658[0n]bp|Austria.Vorarlberg|BOLD:AAE3456
Caloptilia elongella|MM05329|637[0n]bp|Finland.Finland Proper|BOLD:AAE3456
Caloptilia elongella|MM00447|658[0n]bp|Finland.North Karelia|BOLD:AAE3456
[illegible]

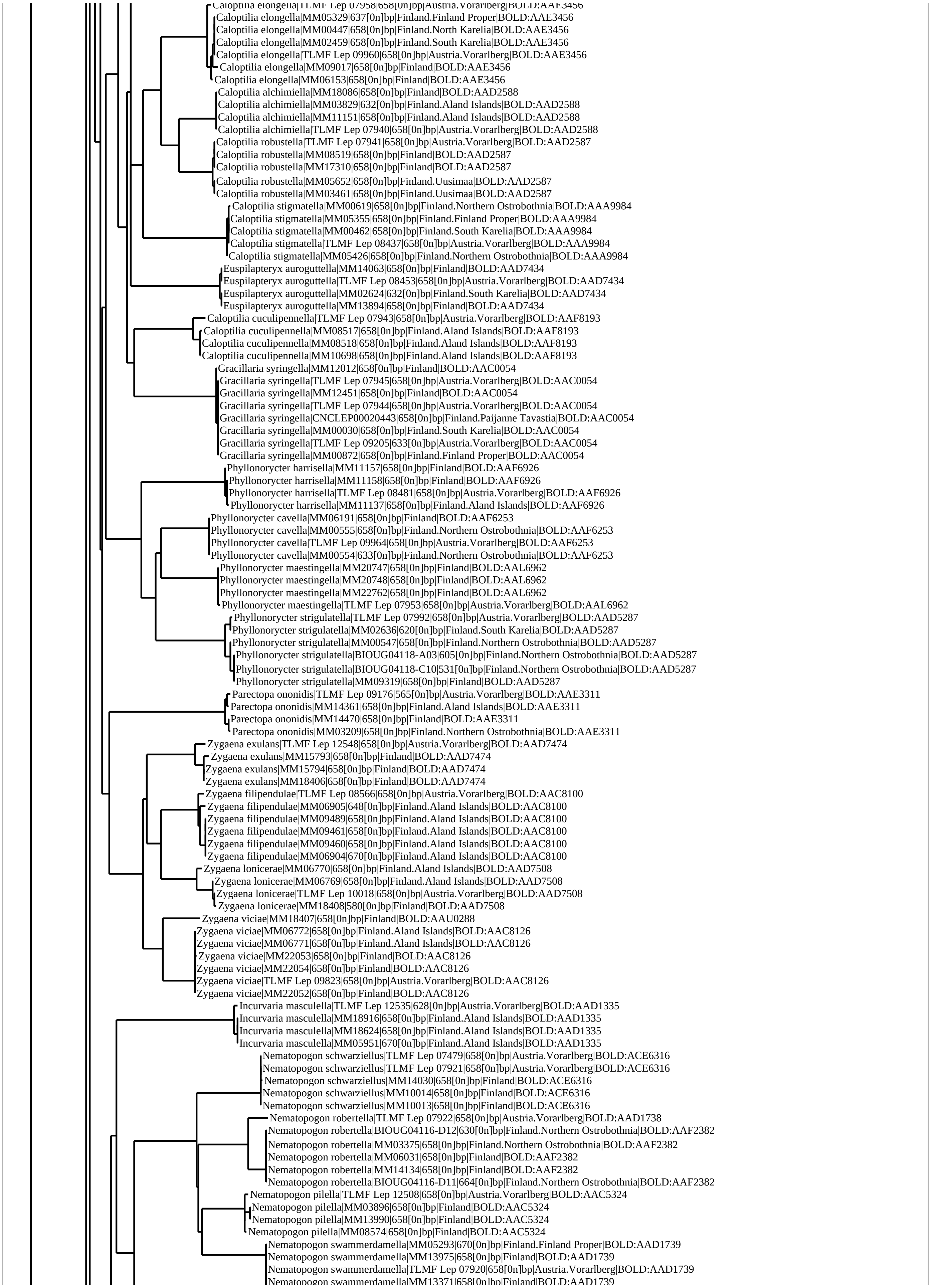
Caloptilia elongella|TLMF Lep 07958|658[0n]bp|Austria.Vorarlberg|BOLD:AAE3456
Caloptilia elongella|MM05329|637[0n]bp|Finland.Finland Proper|BOLD:AAE3456
Caloptilia elongella|MM00447|658[0n]bp|Finland.North Karelia|BOLD:AAE3456
Caloptilia elongella|MM02459|658[0n]bp|Finland.South Karelia|BOLD:AAE3456
Caloptilia elongella|TLMF Lep 09960|658[0n]bp|Austria.Vorarlberg|BOLD:AAE3456
Caloptilia elongella|MM09017|658[0n]bp|Finland|BOLD:AAE3456
Caloptilia elongella|MM06153|658[0n]bp|Finland|BOLD:AAE3456
Caloptilia alchimiella|MM18086|658[0n]bp|Finland|BOLD:AAD2588
Caloptilia alchimiella|MM03829|632[0n]bp|Finland.Aland Islands|BOLD:AAD2588
Caloptilia alchimiella|MM11151|658[0n]bp|Finland.Aland Islands|BOLD:AAD2588
Caloptilia alchimiella|TLMF Lep 07940|658[0n]bp|Austria.Vorarlberg|BOLD:AAD2588
Caloptilia robustella|TLMF Lep 07941|658[0n]bp|Austria.Vorarlberg|BOLD:AAD2587
Caloptilia robustella|MM08519|658[0n]bp|Finland|BOLD:AAD2587
Caloptilia robustella|MM17310|658[0n]bp|Finland|BOLD:AAD2587
Caloptilia robustella|MM05652|658[0n]bp|Finland.Uusimaa|BOLD:AAD2587
Caloptilia robustella|MM03461|658[0n]bp|Finland.Uusimaa|BOLD:AAD2587
Caloptilia stigmatella|MM00619|658[0n]bp|Finland.Northern Ostrobothnia|BOLD:AAA9984
Caloptilia stigmatella|MM05355|658[0n]bp|Finland.Finland Proper|BOLD:AAA9984
Caloptilia stigmatella|MM00462|658[0n]bp|Finland.South Karelia|BOLD:AAA9984
Caloptilia stigmatella|TLMF Lep 08437|658[0n]bp|Austria.Vorarlberg|BOLD:AAA9984
Caloptilia stigmatella|MM05426|658[0n]bp|Finland.Northern Ostrobothnia|BOLD:AAA9984
Euspilapteryx auroguttella|MM14063|658[0n]bp|Finland|BOLD:AAD7434
Euspilapteryx auroguttella|TLMF Lep 08453|658[0n]bp|Austria.Vorarlberg|BOLD:AAD7434
Euspilapteryx auroguttella|MM02624|632[0n]bp|Finland.South Karelia|BOLD:AAD7434
Euspilapteryx auroguttella|MM13894|658[0n]bp|Finland|BOLD:AAD7434
Caloptilia cuculipennella|TLMF Lep 07943|658[0n]bp|Austria.Vorarlberg|BOLD:AAF8193
Caloptilia cuculipennella|MM08517|658[0n]bp|Finland.Aland Islands|BOLD:AAF8193
Caloptilia cuculipennella|MM08518|658[0n]bp|Finland.Aland Islands|BOLD:AAF8193
Caloptilia cuculipennella|MM10698|658[0n]bp|Finland.Aland Islands|BOLD:AAF8193
Gracillaria syringella|MM12012|658[0n]bp|Finland|BOLD:AAC0054
Gracillaria syringella|TLMF Lep 07945|658[0n]bp|Austria.Vorarlberg|BOLD:AAC0054
Gracillaria syringella|MM12451|658[0n]bp|Finland|BOLD:AAC0054
Gracillaria syringella|TLMF Lep 07944|658[0n]bp|Austria.Vorarlberg|BOLD:AAC0054
Gracillaria syringella|CNCLEP00020443|658[0n]bp|Finland.Paijanne Tavastia|BOLD:AAC0054
Gracillaria syringella|MM00030|658[0n]bp|Finland.South Karelia|BOLD:AAC0054
Gracillaria syringella|TLMF Lep 09205|633[0n]bp|Austria.Vorarlberg|BOLD:AAC0054
Gracillaria syringella|MM00872|658[0n]bp|Finland.Finland Proper|BOLD:AAC0054
Phyllonorycter harrisella|MM11157|658[0n]bp|Finland|BOLD:AAF6926
Phyllonorycter harrisella|MM11158|658[0n]bp|Finland|BOLD:AAF6926
Phyllonorycter harrisella|TLMF Lep 08481|658[0n]bp|Austria.Vorarlberg|BOLD:AAF6926
Phyllonorycter harrisella|MM11137|658[0n]bp|Finland.Aland Islands|BOLD:AAF6926
Phyllonorycter cavella|MM06191|658[0n]bp|Finland|BOLD:AAF6253
Phyllonorycter cavella|MM00555|658[0n]bp|Finland.Northern Ostrobothnia|BOLD:AAF6253
Phyllonorycter cavella|TLMF Lep 09964|658[0n]bp|Austria.Vorarlberg|BOLD:AAF6253
Phyllonorycter cavella|MM00554|633[0n]bp|Finland.Northern Ostrobothnia|BOLD:AAF6253
Phyllonorycter maestingella|MM20747|658[0n]bp|Finland|BOLD:AAL6962
Phyllonorycter maestingella|MM20748|658[0n]bp|Finland|BOLD:AAL6962
Phyllonorycter maestingella|MM22762|658[0n]bp|Finland|BOLD:AAL6962
Phyllonorycter maestingella|TLMF Lep 07953|658[0n]bp|Austria.Vorarlberg|BOLD:AAL6962
Phyllonorycter strigulatella|TLMF Lep 07992|658[0n]bp|Austria.Vorarlberg|BOLD:AAD5287
Phyllonorycter strigulatella|MM02636|620[0n]bp|Finland.South Karelia|BOLD:AAD5287
Phyllonorycter strigulatella|MM00547|658[0n]bp|Finland.Northern Ostrobothnia|BOLD:AAD5287
Phyllonorycter strigulatella|BIOUG04118-A03|605[0n]bp|Finland.Northern Ostrobothnia|BOLD:AAD5287
Phyllonorycter strigulatella|BIOUG04118-C10|531[0n]bp|Finland.Northern Ostrobothnia|BOLD:AAD5287
Phyllonorycter strigulatella|MM09319|658[0n]bp|Finland|BOLD:AAD5287
Parectopa ononidis|TLMF Lep 09176|565[0n]bp|Austria.Vorarlberg|BOLD:AAE3311
Parectopa ononidis|MM14361|658[0n]bp|Finland.Aland Islands|BOLD:AAE3311
Parectopa ononidis|MM14470|658[0n]bp|Finland|BOLD:AAE3311
Parectopa ononidis|MM03209|658[0n]bp|Finland.Northern Ostrobothnia|BOLD:AAE3311
Zygaena exulans|TLMF Lep 12548|658[0n]bp|Austria.Vorarlberg|BOLD:AAD7474
Zygaena exulans|MM15793|658[0n]bp|Finland|BOLD:AAD7474
Zygaena exulans|MM15794|658[0n]bp|Finland|BOLD:AAD7474
Zygaena exulans|MM18406|658[0n]bp|Finland|BOLD:AAD7474
Zygaena filipendulae|TLMF Lep 08566|658[0n]bp|Austria.Vorarlberg|BOLD:AAC8100
Zygaena filipendulae|MM06905|648[0n]bp|Finland.Aland Islands|BOLD:AAC8100
Zygaena filipendulae|MM09489|658[0n]bp|Finland.Aland Islands|BOLD:AAC8100
Zygaena filipendulae|MM09461|658[0n]bp|Finland.Aland Islands|BOLD:AAC8100
Zygaena filipendulae|MM09460|658[0n]bp|Finland.Aland Islands|BOLD:AAC8100
Zygaena filipendulae|MM06904|670[0n]bp|Finland.Aland Islands|BOLD:AAC8100
Zygaena lonicerae|MM06770|658[0n]bp|Finland.Aland Islands|BOLD:AAD7508
Zygaena lonicerae|MM06769|658[0n]bp|Finland.Aland Islands|BOLD:AAD7508
Zygaena lonicerae|TLMF Lep 10018|658[0n]bp|Austria.Vorarlberg|BOLD:AAD7508
Zygaena lonicerae|MM18408|580[0n]bp|Finland|BOLD:AAD7508
Zygaena viciae|MM18407|658[0n]bp|Finland|BOLD:AAU0288
Zygaena viciae|MM06772|658[0n]bp|Finland.Aland Islands|BOLD:AAC8126
Zygaena viciae|MM06771|658[0n]bp|Finland.Aland Islands|BOLD:AAC8126
Zygaena viciae|MM22053|658[0n]bp|Finland|BOLD:AAC8126
Zygaena viciae|MM22054|658[0n]bp|Finland|BOLD:AAC8126
Zygaena viciae|TLMF Lep 09823|658[0n]bp|Austria.Vorarlberg|BOLD:AAC8126
Zygaena viciae|MM22052|658[0n]bp|Finland|BOLD:AAC8126
Incurvaria masculella|TLMF Lep 12535|628[0n]bp|Austria.Vorarlberg|BOLD:AAD1335
Incurvaria masculella|MM18916|658[0n]bp|Finland.Aland Islands|BOLD:AAD1335
Incurvaria masculella|MM18624|658[0n]bp|Finland.Aland Islands|BOLD:AAD1335
Incurvaria masculella|MM05951|670[0n]bp|Finland.Aland Islands|BOLD:AAD1335
Nematopogon schwarziellus|TLMF Lep 07479|658[0n]bp|Austria.Vorarlberg|BOLD:ACE6316
Nematopogon schwarziellus|TLMF Lep 07921|658[0n]bp|Austria.Vorarlberg|BOLD:ACE6316
Nematopogon schwarziellus|MM14030|658[0n]bp|Finland|BOLD:ACE6316
Nematopogon schwarziellus|MM10014|658[0n]bp|Finland|BOLD:ACE6316
Nematopogon schwarziellus|MM10013|658[0n]bp|Finland|BOLD:ACE6316
Nematopogon robertella|TLMF Lep 07922|658[0n]bp|Austria.Vorarlberg|BOLD:AAD1738
Nematopogon robertella|BIOUG04116-D12|630[0n]bp|Finland.Northern Ostrobothnia|BOLD:AAF2382
Nematopogon robertella|MM03375|658[0n]bp|Finland.Northern Ostrobothnia|BOLD:AAF2382
Nematopogon robertella|MM06031|658[0n]bp|Finland|BOLD:AAF2382
Nematopogon robertella|MM14134|658[0n]bp|Finland|BOLD:AAF2382
Nematopogon robertella|BIOUG04116-D11|664[0n]bp|Finland.Northern Ostrobothnia|BOLD:AAF2382
Nematopogon pilella|TLMF Lep 12508|658[0n]bp|Austria.Vorarlberg|BOLD:AAC5324
Nematopogon pilella|MM03896|658[0n]bp|Finland|BOLD:AAC5324
Nematopogon pilella|MM13990|658[0n]bp|Finland|BOLD:AAC5324
Nematopogon pilella|MM08574|658[0n]bp|Finland|BOLD:AAC5324
Nematopogon swammerdamella|MM05293|670[0n]bp|Finland.Finland Proper|BOLD:AAD1739
Nematopogon swammerdamella|MM13975|658[0n]bp|Finland|BOLD:AAD1739
Nematopogon swammerdamella|TLMF Lep 07920|658[0n]bp|Austria.Vorarlberg|BOLD:AAD1739
Nematopogon swammerdamella|MM13371|658[0n]bp|Finland|BOLD:AAD1739

Nematopogon swammerdamella|MM13975|658[0n]bp|Finland|BOLD:AAD1739
Nematopogon swammerdamella|TLMF Lep 07920|658[0n]bp|Austria.Vorarlberg|BOLD:AAD1739
Nematopogon swammerdamella|MM13371|658[0n]bp|Finland|BOLD:AAD1739
Nematopogon swammerdamella|MM05961|631[0n]bp|Finland.Aland Islands|BOLD:AAD1739
Nemophora degeerella|MM15509|650[0n]bp|Finland.Aland Islands|BOLD:AAC1892
Nemophora degeerella|MM09554|658[0n]bp|Finland.Aland Islands|BOLD:AAC1892
Nemophora degeerella|MM09760|628[0n]bp|Finland.Aland Islands|BOLD:AAC1892
Nemophora degeerella|MM15508|623[0n]bp|Finland|BOLD:AAC1892
Nemophora degeerella|TLMF Lep 09164|620[0n]bp|Austria.Vorarlberg|BOLD:AAC1892
Nemophora degeerella|MM06587|658[0n]bp|Finland|BOLD:AAC1892
Nemophora metallica|TLMF Lep 08014|658[0n]bp|Austria.Vorarlberg|BOLD:AAE5236
Nemophora metallica|MM06621|658[0n]bp|Finland|BOLD:ACF0411
Nemophora metallica|MM06622|633[0n]bp|Finland|BOLD:ACF0411
Nemophora metallica|MM06620|658[0n]bp|Finland|BOLD:ACF0411
Lampronia rupella|TLMF Lep 04291|658[0n]bp|Austria.Vorarlberg|BOLD:AAD5208
Lampronia rupella|TLMF Lep 04292|658[0n]bp|Austria.Vorarlberg|BOLD:AAD5208
Lampronia rupella|MM14304|649[0n]bp|Finland.Aland Islands|BOLD:AAD5208
Lampronia rupella|MM02270|597[0n]bp|Finland.South Karelia|BOLD:AAD5208
Lampronia rupella|MM06494|658[0n]bp|Finland|BOLD:AAD5208
Lampronia corticella|MM15520|658[0n]bp|Finland|BOLD:AAD7517
Lampronia corticella|MM15519|658[0n]bp|Finland|BOLD:AAD7517
Lampronia corticella|MM05349|571[0n]bp|Finland.Finland Proper|BOLD:AAD7517
Lampronia corticella|TLMF Lep 07690|658[0n]bp|Austria|BOLD:AAD7517
Taleporia tubulosa|MM13263|632[0n]bp|Finland|BOLD:AAC2838
Taleporia tubulosa|MM02175|670[0n]bp|Finland.South Karelia|BOLD:AAC2838
Taleporia tubulosa|MM14274|658[0n]bp|Finland.Aland Islands|BOLD:AAC2838
Taleporia tubulosa|MM05290|637[0n]bp|Finland.Finland Proper|BOLD:AAC2838
Taleporia tubulosa|MM10085|658[0n]bp|Finland|BOLD:AAC2838
Taleporia tubulosa|TLMF Lep 08242|658[0n]bp|Austria.Vorarlberg|BOLD:AAC2838
Taleporia tubulosa|MM09951|658[0n]bp|Finland|BOLD:AAC2838
Taleporia tubulosa|MM09952|658[0n]bp|Finland|BOLD:AAC2838
Taleporia tubulosa|MM02176|658[0n]bp|Finland.South Karelia|BOLD:AAC2838
Coptotriche angusticollella|MM00889|658[0n]bp|Finland.Aland Islands|BOLD:AAF8244
Coptotriche angusticollella|TLMF Lep 08483|658[0n]bp|Austria.Vorarlberg|BOLD:AAF8244
Coptotriche angusticollella|MM00891|658[0n]bp|Finland.Aland Islands|BOLD:AAF8244
Coptotriche angusticollella|MM00890|658[0n]bp|Finland.Aland Islands|BOLD:AAF8244
Coptotriche marginea|MM09666|658[0n]bp|Finland.Aland Islands|BOLD:AAF5585
Coptotriche marginea|MM21091|658[0n]bp|Finland|BOLD:AAF5585
Coptotriche marginea|MM08720|658[0n]bp|Finland|BOLD:AAF5585
Coptotriche marginea|MM09181|658[0n]bp|Finland|BOLD:AAF5585
Coptotriche marginea|TLMF Lep 08424|658[0n]bp|Austria.Vorarlberg|BOLD:AAF5585
Tinea pellionella|MM06612|658[0n]bp|Finland|BOLD:AAB9203
Tinea pellionella|MM06611|670[0n]bp|Finland.North Karelia|BOLD:AAB9203
Tinea pellionella|TLMF Lep 08474|605[0n]bp|Austria.Vorarlberg|BOLD:AAB9203
Tinea pellionella|TLMF Lep 09909|658[0n]bp|Austria.Vorarlberg|BOLD:AAB9203
Tinea pellionella|MM10377|658[0n]bp|Finland.Aland Islands|BOLD:AAB9203
Tinea pellionella|TLMF Lep 09906|658[0n]bp|Austria.Vorarlberg|BOLD:AAB9203
Tinea semifulvella|MM09751|658[0n]bp|Finland.Aland Islands|BOLD:AAC3847
Tinea semifulvella|MM02338|658[0n]bp|Finland.South Karelia|BOLD:AAC3847
Tinea semifulvella|MM03823|658[0n]bp|Finland.Aland Islands|BOLD:AAC3847
Tinea semifulvella|TLMF Lep 07968|658[0n]bp|Austria.Vorarlberg|BOLD:AAC3847
Tinea semifulvella|MM13522|658[0n]bp|Finland|BOLD:AAC3847
Tinea trinotella|MM10309|658[0n]bp|Finland|BOLD:AAD5562
Tinea trinotella|TLMF Lep 07966|658[0n]bp|Austria.Vorarlberg|BOLD:AAD5562
Tinea trinotella|TLMF Lep 07937|658[0n]bp|Austria.Vorarlberg|BOLD:AAD5562
Tinea trinotella|MM08378|658[0n]bp|Finland|BOLD:AAD5562
Ectoedemia sericopeza|TLMF Lep 07484|658[0n]bp|Austria.Vorarlberg|BOLD:AAC4337
Ectoedemia sericopeza|MM17329|658[0n]bp|Finland|BOLD:AAC4337
Ectoedemia sericopeza|MM14880|658[0n]bp|Finland|BOLD:AAC4337
Ectoedemia sericopeza|MM21131|658[0n]bp|Finland|BOLD:AAC4337
Ectoedemia sericopeza|MM21037|658[0n]bp|Finland|BOLD:AAC4337
Stigmella myrtillella|MM21040|658[0n]bp|Finland|BOLD:AAJ4189
Stigmella myrtillella|MM00936|658[0n]bp|Finland.Northern Ostrobothnia|BOLD:AAJ4189
Stigmella myrtillella|MM15174|658[0n]bp|Finland|BOLD:AAJ4189
Stigmella myrtillella|MM06330|658[0n]bp|Finland|BOLD:AAJ4189
Stigmella myrtillella|MM00935|658[0n]bp|Finland.Northern Ostrobothnia|BOLD:AAJ4189
Stigmella myrtillella|TLMF Lep 09216|658[0n]bp|Austria.Vorarlberg|BOLD:AAJ4189
Stigmella floslactella|MM07007|658[0n]bp|Finland|BOLD:AAF3363
Stigmella floslactella|MM09605|658[0n]bp|Finland.Aland Islands|BOLD:AAF3363
Stigmella floslactella|MM09606|658[0n]bp|Finland.Aland Islands|BOLD:AAF3363
Stigmella floslactella|TLMF Lep 08300|648[0n]bp|Austria.Vorarlberg|BOLD:AAF3363
Stigmella dryadella|TLMF Lep 02871|658[0n]bp|Austria.Vorarlberg|BOLD:AAV8362
Stigmella dryadella|MM23205|658[0n]bp|Finland|BOLD:ACD1518
Stigmella pretiosa|MM09642|658[0n]bp|Finland.Aland Islands|BOLD:AAI0004
Stigmella pretiosa|MM09643|658[0n]bp|Finland.Aland Islands|BOLD:AAI0004
Stigmella pretiosa|MM22761|658[0n]bp|Finland|BOLD:AAI0004
Stigmella pretiosa|TLMF Lep 02872|579[0n]bp|Austria.Vorarlberg|BOLD:AAI0004
Eriocrania semipurpurella|MM19773|658[0n]bp|Finland.Uusimaa|BOLD:AAB3768
Eriocrania semipurpurella|MM13110|658[0n]bp|Finland|BOLD:AAB3768
Eriocrania semipurpurella|MM19833|658[0n]bp|Finland.Northern Ostrobothnia|BOLD:AAB3768
Eriocrania semipurpurella|MM09028|658[0n]bp|Finland|BOLD:AAB3768
Eriocrania semipurpurella|MM16675|658[0n]bp|Finland|BOLD:AAB3768
Eriocrania semipurpurella|MM16679|658[0n]bp|Finland|BOLD:AAB3768
Eriocrania semipurpurella|MM19753|658[0n]bp|Finland.Uusimaa|BOLD:AAB3768
Eriocrania semipurpurella|MM19768|658[0n]bp|Finland.Uusimaa|BOLD:AAB3768
Eriocrania semipurpurella|MM19769|658[0n]bp|Finland.Uusimaa|BOLD:AAB3768
Eriocrania semipurpurella|MM19770|658[0n]bp|Finland.Uusimaa|BOLD:AAB3768
Eriocrania semipurpurella|MM19771|658[0n]bp|Finland.Uusimaa|BOLD:AAB3768
Eriocrania semipurpurella|MM19776|658[0n]bp|Finland.Uusimaa|BOLD:AAB3768
Eriocrania semipurpurella|MM19828|658[0n]bp|Finland.Northern Ostrobothnia|BOLD:AAB3768
Eriocrania semipurpurella|MM19830|658[0n]bp|Finland.Northern Ostrobothnia|BOLD:AAB3768
Eriocrania semipurpurella|MM19835|658[0n]bp|Finland.Northern Ostrobothnia|BOLD:AAB3768
Eriocrania semipurpurella|MM19755|658[0n]bp|Finland.Uusimaa|BOLD:AAB3768
Eriocrania semipurpurella|MM05324|639[0n]bp|Finland.Finland Proper|BOLD:AAB3768
Eriocrania semipurpurella|MM19775|635[0n]bp|Finland.Uusimaa|BOLD:AAB3768
Eriocrania sangii|MM19758|550[0n]bp|Finland.Uusimaa|
Eriocrania sangii|MM05747|658[0n]bp|Finland|BOLD:AAB3766
Eriocrania sangii|MM17929|658[0n]bp|Finland|BOLD:AAB3766
Eriocrania sangii|MM17927|658[0n]bp|Finland|BOLD:AAB3766
Eriocrania sangii|MM14777|632[0n]bp|Finland|BOLD:AAB3766
Eriocrania sangii|MM05750|658[0n]bp|Finland|BOLD:AAB3766
Eriocrania sangii|MM17912|658[0n]bp|Finland|BOLD:AAB3766
Eriocrania sangii|MM19760|658[0n]bp|Finland.Uusimaa|BOLD:AAB3766

Eriocrania sangii|MM14777|652[0n]bp|Finland|BOLD:AAB3766
Eriocrania sangii|MM05750|658[0n]bp|Finland|BOLD:AAB3766
Eriocrania sangii|MM17912|658[0n]bp|Finland|BOLD:AAB3766
Eriocrania sangii|MM19760|658[0n]bp|Finland.Uusimaa|BOLD:AAB3766
Eriocrania sangii|MM12061|658[0n]bp|Finland|BOLD:AAB3766
Eriocrania sangii|MM19761|658[0n]bp|Finland.Uusimaa|BOLD:AAB3766
Eriocrania sangii|MM17954|658[0n]bp|Finland|BOLD:AAB3766
Eriocrania sangii|MM19766|658[0n]bp|Finland.Uusimaa|BOLD:AAB3766
Eriocrania sangii|MM21223|658[0n]bp|Finland|BOLD:AAB3766
Eriocrania sangii|MM17924|658[0n]bp|Finland|BOLD:AAB3766
Eriocrania sangii|MM08799|658[0n]bp|Finland|BOLD:AAB3766
Eriocrania sangii|MM00431|658[0n]bp|Finland.Northern Ostrobothnia|BOLD:AAB3766
Eriocrania sangii|MM00391|658[0n]bp|Finland.Northern Ostrobothnia|BOLD:AAB3766
Eriocrania sangii|MM08795|658[0n]bp|Finland|BOLD:AAB3766
Eriocrania sangii|MM17926|658[0n]bp|Finland|BOLD:AAB3766
Eriocrania sangii|MM19767|658[0n]bp|Finland.Uusimaa|BOLD:AAB3766
Eriocrania sangii|MM19759|658[0n]bp|Finland.Uusimaa|BOLD:AAB3766
Eriocrania sangii|TLMF Lep 02973|658[0n]bp|Austria|BOLD:AAB3766
Eriocrania sangii|MM08793|617[0n]bp|Finland|BOLD:AAB3766
Eriocrania sangii|MM05315|545[0n]bp|Finland.Finland Proper|BOLD:AAB3766
Eriocrania sangii|MM05316|584[0n]bp|Finland.Finland Proper|BOLD:AAB3766
Eriocrania semipurpurella|MM00390|670[0n]bp|Finland.Northern Ostrobothnia|BOLD:AAB3767
Eriocrania semipurpurella|MM13130|658[0n]bp|Finland|BOLD:AAB3767
Eriocrania semipurpurella|MM14781|658[0n]bp|Finland|BOLD:AAB3767
Eriocrania semipurpurella|TLMF Lep 02954|658[0n]bp|Austria|BOLD:AAB3767
Eriocrania semipurpurella|MM19750|658[0n]bp|Finland.Uusimaa|BOLD:AAB3767
Eriocrania semipurpurella|MM19752|658[0n]bp|Finland.Uusimaa|BOLD:AAB3767
Eriocrania semipurpurella|MM19754|658[0n]bp|Finland.Uusimaa|BOLD:AAB3767
Eriocrania semipurpurella|MM19756|658[0n]bp|Finland.Uusimaa|BOLD:AAB3767
Eriocrania semipurpurella|MM19757|658[0n]bp|Finland.Uusimaa|BOLD:AAB3767
Eriocrania semipurpurella|MM19765|658[0n]bp|Finland.Uusimaa|BOLD:AAB3767
Eriocrania semipurpurella|MM19826|658[0n]bp|Finland.Northern Ostrobothnia|BOLD:AAB3767
Eriocrania semipurpurella|MM19827|658[0n]bp|Finland|BOLD:AAB3767
Eriocrania semipurpurella|MM19831|658[0n]bp|Finland.Northern Ostrobothnia|BOLD:AAB3767
Eriocrania semipurpurella|MM19837|658[0n]bp|Finland.Northern Ostrobothnia|BOLD:AAB3767
Eriocrania semipurpurella|MM19838|658[0n]bp|Finland.Northern Ostrobothnia|BOLD:AAB3767
Eriocrania semipurpurella|MM13105|658[0n]bp|Finland|BOLD:AAB3767
Eriocrania semipurpurella|MM13107|658[0n]bp|Finland|BOLD:AAB3767
Eriocrania semipurpurella|MM13109|658[0n]bp|Finland|BOLD:AAB3767
Eriocrania semipurpurella|MM13113|658[0n]bp|Finland|BOLD:AAB3767
Eriocrania semipurpurella|MM13118|658[0n]bp|Finland|BOLD:AAB3767
Eriocrania semipurpurella|MM13121|658[0n]bp|Finland|BOLD:AAB3767
Eriocrania semipurpurella|MM13122|658[0n]bp|Finland|BOLD:AAB3767
Eriocrania semipurpurella|MM13123|658[0n]bp|Finland|BOLD:AAB3767
Eriocrania semipurpurella|MM13124|658[0n]bp|Finland|BOLD:AAB3767
Eriocrania semipurpurella|MM13125|658[0n]bp|Finland|BOLD:AAB3767
Eriocrania semipurpurella|MM13126|658[0n]bp|Finland|BOLD:AAB3767
Eriocrania semipurpurella|MM13128|658[0n]bp|Finland|BOLD:AAB3767
Eriocrania semipurpurella|MM13129|658[0n]bp|Finland|BOLD:AAB3767
Eriocrania semipurpurella|MM05749|658[0n]bp|Finland|BOLD:AAB3767
Eriocrania semipurpurella|MM05742|658[0n]bp|Finland|BOLD:AAB3767
Eriocrania semipurpurella|MM05745|658[0n]bp|Finland|BOLD:AAB3767
Eriocrania semipurpurella|MM05746|658[0n]bp|Finland|BOLD:AAB3767
Eriocrania semipurpurella|MM00434|658[0n]bp|Finland.Northern Ostrobothnia|BOLD:AAB3767
Eriocrania semipurpurella|MM05321|658[0n]bp|Finland.Finland Proper|BOLD:AAB3767
Eriocrania semipurpurella|MM05320|658[0n]bp|Finland.Finland Proper|BOLD:AAB3767
Eriocrania semipurpurella|MM05319|658[0n]bp|Finland.Finland Proper|BOLD:AAB3767
Eriocrania semipurpurella|MM12060|658[0n]bp|Finland|BOLD:AAB3767
Eriocrania semipurpurella|MM08796|658[0n]bp|Finland|BOLD:AAB3767
Eriocrania semipurpurella|MM14778|658[0n]bp|Finland|BOLD:AAB3767
Eriocrania semipurpurella|MM21222|658[0n]bp|Finland|BOLD:AAB3767
Eriocrania semipurpurella|MM19764|658[0n]bp|Finland.Uusimaa|BOLD:AAB3767
Eriocrania semipurpurella|MM05323|641[0n]bp|Finland.Finland Proper|BOLD:AAB3767
Eriocrania semipurpurella|MM19751|627[0n]bp|Finland.Uusimaa|BOLD:AAB3767
Eriocrania semipurpurella|MM17922|658[0n]bp|Finland|BOLD:AAB3764
Eriocrania semipurpurella|MM16677|658[0n]bp|Finland|BOLD:AAB3764
Eriocrania semipurpurella|MM17928|658[0n]bp|Finland|BOLD:AAB3764
Eriocrania semipurpurella|MM17925|658[0n]bp|Finland|BOLD:AAB3764
Eriocrania semipurpurella|MM16676|658[0n]bp|Finland|BOLD:AAB3764
Eriocrania semipurpurella|MM13117|658[0n]bp|Finland|BOLD:AAB3764
Eriocrania semipurpurella|MM13106|632[0n]bp|Finland|BOLD:AAB3764
Eriocrania semipurpurella|MM14779|658[0n]bp|Finland|BOLD:AAB3764
Eriocrania semipurpurella|MM14780|658[0n]bp|Finland|BOLD:AAB3764
Eriocrania semipurpurella|MM13108|658[0n]bp|Finland|BOLD:AAB3764
Eriocrania semipurpurella|MM13119|658[0n]bp|Finland|BOLD:AAB3764
Eriocrania semipurpurella|MM05748|658[0n]bp|Finland|BOLD:AAB3764
Eriocrania semipurpurella|MM17909|658[0n]bp|Finland|BOLD:AAB3764
Eriocrania semipurpurella|MM00430|658[0n]bp|Finland.Northern Ostrobothnia|BOLD:AAB3764
Eriocrania semipurpurella|MM00432|658[0n]bp|Finland.Northern Ostrobothnia|BOLD:AAB3764
Eriocrania semipurpurella|MM21218|658[0n]bp|Finland|BOLD:AAB3764
Eriocrania semipurpurella|MM21219|658[0n]bp|Finland|BOLD:AAB3764
Eriocrania semipurpurella|MM21220|658[0n]bp|Finland|BOLD:AAB3764
Eriocrania semipurpurella|MM21224|658[0n]bp|Finland|BOLD:AAB3764
Eriocrania semipurpurella|MM00392|618[0n]bp|Finland.Northern Ostrobothnia|BOLD:AAB3764
Eriocrania semipurpurella|MM13127|658[0n]bp|Finland|BOLD:AAB3764
Eriocrania semipurpurella|MM17923|633[0n]bp|Finland|BOLD:AAB3764
Eriocrania semipurpurella|MM00403|647[0n]bp|Finland.Northern Ostrobothnia|BOLD:AAB3764
Eriocrania semipurpurella|MM19825|633[0n]bp|Finland.Northern Ostrobothnia|BOLD:AAB3764
Eriocrania semipurpurella|MM13116|633[0n]bp|Finland|BOLD:AAB3764
Eriocrania semipurpurella|MM00414|642[0n]bp|Finland.Northern Ostrobothnia|BOLD:AAB3764
Eriocrania semipurpurella|MM13112|602[0n]bp|Finland|BOLD:AAB3764
Eriocrania semipurpurella|MM00404|658[0n]bp|Finland.Northern Ostrobothnia|BOLD:AAB3764
Eriocrania semipurpurella|MM00429|658[0n]bp|Finland.Northern Ostrobothnia|BOLD:AAB3764
Eriocrania semipurpurella|MM00405|658[0n]bp|Finland.Northern Ostrobothnia|BOLD:AAB3764
Eriocrania semipurpurella|MM21225|658[0n]bp|Finland|BOLD:AAB3764
Eriocrania semipurpurella|MM21221|658[0n]bp|Finland|BOLD:AAB3764
Eriocrania semipurpurella|MM17955|658[0n]bp|Finland|BOLD:AAB3764
Eriocrania semipurpurella|MM13111|658[0n]bp|Finland|BOLD:AAB3764
Eriocrania semipurpurella|MM17910|658[0n]bp|Finland|BOLD:AAB3764
Eriocrania semipurpurella|MM08794|635[0n]bp|Finland|BOLD:AAB3764
Eriocrania semipurpurella|MM08797|572[0n]bp|Finland|BOLD:AAB3764
Heringocrania unimaculella|TLMF Lep 09897|658[0n]bp|Austria.Vorarlberg|BOLD:AAF3179
Heringocrania unimaculella|MM08800|658[0n]bp|Finland|BOLD:AAF3179

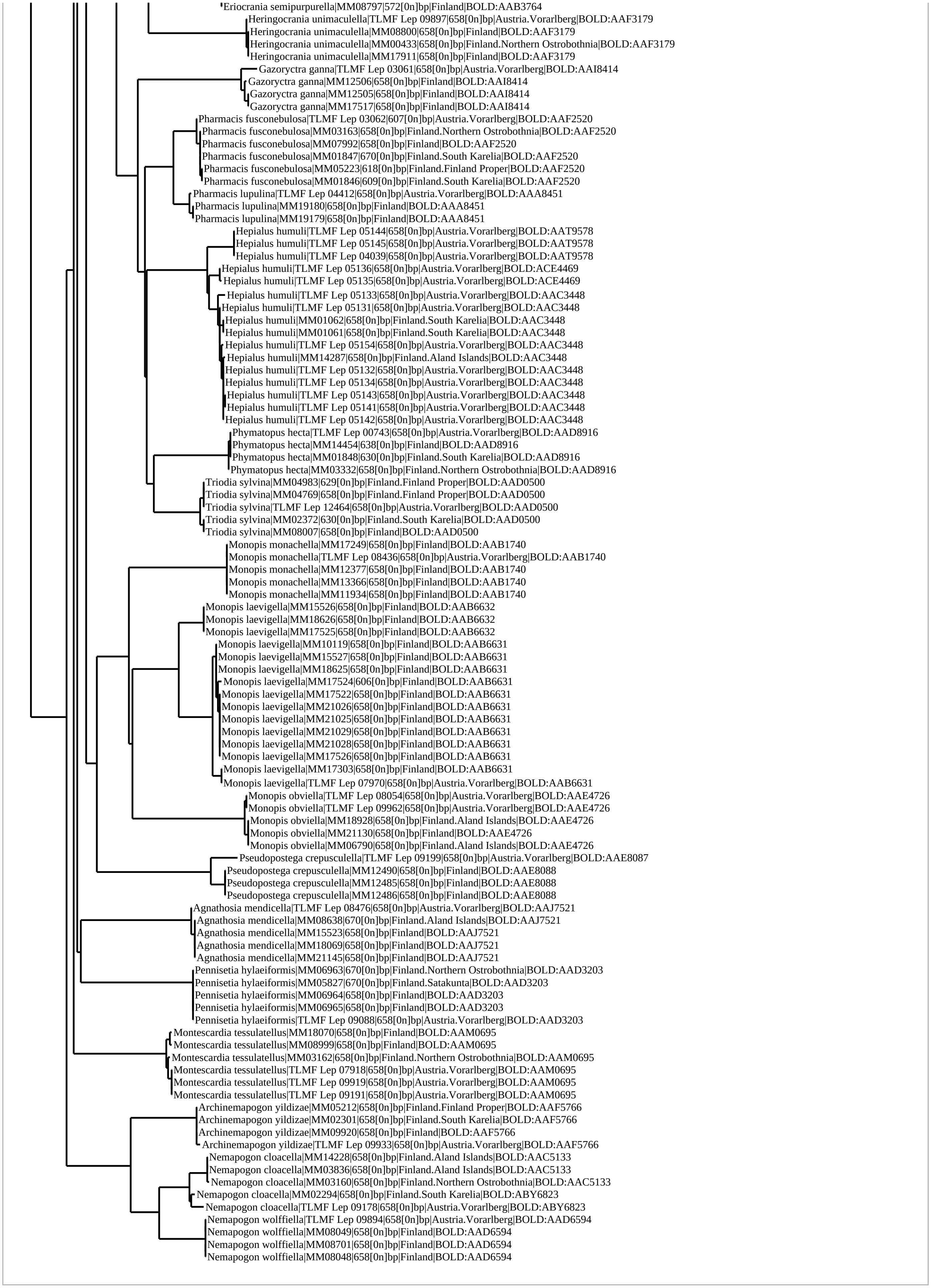
Eriocrania semipurpurella|MM08797|572[0n]bp|Finland|BOLD:AAB3764
Heringocrania unimaculella|TLMF Lep 09897|658[0n]bp|Austria.Vorarlberg|BOLD:AAF3179
Heringocrania unimaculella|MM08800|658[0n]bp|Finland|BOLD:AAF3179
Heringocrania unimaculella|MM00433|658[0n]bp|Finland.Northern Ostrobothnia|BOLD:AAF3179
Heringocrania unimaculella|MM17911|658[0n]bp|Finland|BOLD:AAF3179
Gazoryctra ganna|TLMF Lep 03061|658[0n]bp|Austria.Vorarlberg|BOLD:AAI8414
Gazoryctra ganna|MM12506|658[0n]bp|Finland|BOLD:AAI8414
Gazoryctra ganna|MM12505|658[0n]bp|Finland|BOLD:AAI8414
Gazoryctra ganna|MM17517|658[0n]bp|Finland|BOLD:AAI8414
Pharmacis fusconebulosa|TLMF Lep 03062|607[0n]bp|Austria.Vorarlberg|BOLD:AAF2520
Pharmacis fusconebulosa|MM03163|658[0n]bp|Finland.Northern Ostrobothnia|BOLD:AAF2520
Pharmacis fusconebulosa|MM07992|658[0n]bp|Finland|BOLD:AAF2520
Pharmacis fusconebulosa|MM01847|670[0n]bp|Finland.South Karelia|BOLD:AAF2520
Pharmacis fusconebulosa|MM05223|618[0n]bp|Finland.Finland Proper|BOLD:AAF2520
Pharmacis fusconebulosa|MM01846|609[0n]bp|Finland.South Karelia|BOLD:AAF2520
Pharmacis lupulina|TLMF Lep 04412|658[0n]bp|Austria.Vorarlberg|BOLD:AAA8451
Pharmacis lupulina|MM19180|658[0n]bp|Finland|BOLD:AAA8451
Pharmacis lupulina|MM19179|658[0n]bp|Finland|BOLD:AAA8451
Hepialus humuli|TLMF Lep 05144|658[0n]bp|Austria.Vorarlberg|BOLD:AAT9578
Hepialus humuli|TLMF Lep 05145|658[0n]bp|Austria.Vorarlberg|BOLD:AAT9578
Hepialus humuli|TLMF Lep 04039|658[0n]bp|Austria.Vorarlberg|BOLD:AAT9578
Hepialus humuli|TLMF Lep 05136|658[0n]bp|Austria.Vorarlberg|BOLD:ACE4469
Hepialus humuli|TLMF Lep 05135|658[0n]bp|Austria.Vorarlberg|BOLD:ACE4469
Hepialus humuli|TLMF Lep 05133|658[0n]bp|Austria.Vorarlberg|BOLD:AAC3448
Hepialus humuli|TLMF Lep 05131|658[0n]bp|Austria.Vorarlberg|BOLD:AAC3448
Hepialus humuli|MM01062|658[0n]bp|Finland.South Karelia|BOLD:AAC3448
Hepialus humuli|MM01061|658[0n]bp|Finland.South Karelia|BOLD:AAC3448
Hepialus humuli|TLMF Lep 05154|658[0n]bp|Austria.Vorarlberg|BOLD:AAC3448
Hepialus humuli|MM14287|658[0n]bp|Finland.Aland Islands|BOLD:AAC3448
Hepialus humuli|TLMF Lep 05132|658[0n]bp|Austria.Vorarlberg|BOLD:AAC3448
Hepialus humuli|TLMF Lep 05134|658[0n]bp|Austria.Vorarlberg|BOLD:AAC3448
Hepialus humuli|TLMF Lep 05143|658[0n]bp|Austria.Vorarlberg|BOLD:AAC3448
Hepialus humuli|TLMF Lep 05141|658[0n]bp|Austria.Vorarlberg|BOLD:AAC3448
Hepialus humuli|TLMF Lep 05142|658[0n]bp|Austria.Vorarlberg|BOLD:AAC3448
Phymatopus hecta|TLMF Lep 00743|658[0n]bp|Austria.Vorarlberg|BOLD:AAD8916
Phymatopus hecta|MM14454|638[0n]bp|Finland|BOLD:AAD8916
Phymatopus hecta|MM01848|630[0n]bp|Finland.South Karelia|BOLD:AAD8916
Phymatopus hecta|MM03332|658[0n]bp|Finland.Northern Ostrobothnia|BOLD:AAD8916
Triodia sylvina|MM04983|629[0n]bp|Finland.Finland Proper|BOLD:AAD0500
Triodia sylvina|MM04769|658[0n]bp|Finland.Finland Proper|BOLD:AAD0500
Triodia sylvina|TLMF Lep 12464|658[0n]bp|Austria.Vorarlberg|BOLD:AAD0500
Triodia sylvina|MM02372|630[0n]bp|Finland.South Karelia|BOLD:AAD0500
Triodia sylvina|MM08007|658[0n]bp|Finland|BOLD:AAD0500
Monopis monachella|MM17249|658[0n]bp|Finland|BOLD:AAB1740
Monopis monachella|TLMF Lep 08436|658[0n]bp|Austria.Vorarlberg|BOLD:AAB1740
Monopis monachella|MM12377|658[0n]bp|Finland|BOLD:AAB1740
Monopis monachella|MM13366|658[0n]bp|Finland|BOLD:AAB1740
Monopis monachella|MM11934|658[0n]bp|Finland|BOLD:AAB1740
Monopis laevigella|MM15526|658[0n]bp|Finland|BOLD:AAB6632
Monopis laevigella|MM18626|658[0n]bp|Finland|BOLD:AAB6632
Monopis laevigella|MM17525|658[0n]bp|Finland|BOLD:AAB6632
Monopis laevigella|MM10119|658[0n]bp|Finland|BOLD:AAB6631
Monopis laevigella|MM15527|658[0n]bp|Finland|BOLD:AAB6631
Monopis laevigella|MM18625|658[0n]bp|Finland|BOLD:AAB6631
Monopis laevigella|MM17524|606[0n]bp|Finland|BOLD:AAB6631
Monopis laevigella|MM17522|658[0n]bp|Finland|BOLD:AAB6631
Monopis laevigella|MM21026|658[0n]bp|Finland|BOLD:AAB6631
Monopis laevigella|MM21025|658[0n]bp|Finland|BOLD:AAB6631
Monopis laevigella|MM21029|658[0n]bp|Finland|BOLD:AAB6631
Monopis laevigella|MM21028|658[0n]bp|Finland|BOLD:AAB6631
Monopis laevigella|MM17526|658[0n]bp|Finland|BOLD:AAB6631
Monopis laevigella|MM17303|658[0n]bp|Finland|BOLD:AAB6631
Monopis laevigella|TLMF Lep 07970|658[0n]bp|Austria.Vorarlberg|BOLD:AAB6631
Monopis obviella|TLMF Lep 08054|658[0n]bp|Austria.Vorarlberg|BOLD:AAE4726
Monopis obviella|TLMF Lep 09962|658[0n]bp|Austria.Vorarlberg|BOLD:AAE4726
Monopis obviella|MM18928|658[0n]bp|Finland.Aland Islands|BOLD:AAE4726
Monopis obviella|MM21130|658[0n]bp|Finland|BOLD:AAE4726
Monopis obviella|MM06790|658[0n]bp|Finland.Aland Islands|BOLD:AAE4726
Pseudopostega crepusculella|TLMF Lep 09199|658[0n]bp|Austria.Vorarlberg|BOLD:AAE8087
Pseudopostega crepusculella|MM12490|658[0n]bp|Finland|BOLD:AAE8088
Pseudopostega crepusculella|MM12485|658[0n]bp|Finland|BOLD:AAE8088
Pseudopostega crepusculella|MM12486|658[0n]bp|Finland|BOLD:AAE8088
Agnathosia mendicella|TLMF Lep 08476|658[0n]bp|Austria.Vorarlberg|BOLD:AAJ7521
Agnathosia mendicella|MM08638|670[0n]bp|Finland.Aland Islands|BOLD:AAJ7521
Agnathosia mendicella|MM15523|658[0n]bp|Finland|BOLD:AAJ7521
Agnathosia mendicella|MM18069|658[0n]bp|Finland|BOLD:AAJ7521
Agnathosia mendicella|MM21145|658[0n]bp|Finland|BOLD:AAJ7521
Pennisetia hylaeiformis|MM06963|670[0n]bp|Finland.Northern Ostrobothnia|BOLD:AAD3203
Pennisetia hylaeiformis|MM05827|670[0n]bp|Finland.Satakunta|BOLD:AAD3203
Pennisetia hylaeiformis|MM06964|658[0n]bp|Finland|BOLD:AAD3203
Pennisetia hylaeiformis|MM06965|658[0n]bp|Finland|BOLD:AAD3203
Pennisetia hylaeiformis|TLMF Lep 09088|658[0n]bp|Austria.Vorarlberg|BOLD:AAD3203
Montescardia tessulatellus|MM18070|658[0n]bp|Finland|BOLD:AAM0695
Montescardia tessulatellus|MM08999|658[0n]bp|Finland|BOLD:AAM0695
Montescardia tessulatellus|MM03162|658[0n]bp|Finland.Northern Ostrobothnia|BOLD:AAM0695
Montescardia tessulatellus|TLMF Lep 07918|658[0n]bp|Austria.Vorarlberg|BOLD:AAM0695
Montescardia tessulatellus|TLMF Lep 09919|658[0n]bp|Austria.Vorarlberg|BOLD:AAM0695
Montescardia tessulatellus|TLMF Lep 09191|658[0n]bp|Austria.Vorarlberg|BOLD:AAM0695
Archinemapogon yildizae|MM05212|658[0n]bp|Finland.Finland Proper|BOLD:AAF5766
Archinemapogon yildizae|MM02301|658[0n]bp|Finland.South Karelia|BOLD:AAF5766
Archinemapogon yildizae|MM09920|658[0n]bp|Finland|BOLD:AAF5766
Archinemapogon yildizae|TLMF Lep 09933|658[0n]bp|Austria.Vorarlberg|BOLD:AAF5766
Nemapogon cloacella|MM14228|658[0n]bp|Finland.Aland Islands|BOLD:AAC5133
Nemapogon cloacella|MM03836|658[0n]bp|Finland.Aland Islands|BOLD:AAC5133
Nemapogon cloacella|MM03160|658[0n]bp|Finland.Northern Ostrobothnia|BOLD:AAC5133
Nemapogon cloacella|MM02294|658[0n]bp|Finland.South Karelia|BOLD:ABY6823
Nemapogon cloacella|TLMF Lep 09178|658[0n]bp|Austria.Vorarlberg|BOLD:ABY6823
Nemapogon wolffiella|TLMF Lep 09894|658[0n]bp|Austria.Vorarlberg|BOLD:AAD6594
Nemapogon wolffiella|MM08049|658[0n]bp|Finland|BOLD:AAD6594
Nemapogon wolffiella|MM08701|658[0n]bp|Finland|BOLD:AAD6594
Nemapogon wolffiella|MM08048|658[0n]bp|Finland|BOLD:AAD6594
